# Amino acid appended supramolecular self-associating amphiphiles demonstrate dual activity against both MRSA and ovarian cancer

**DOI:** 10.1039/d5sc03376d

**Published:** 2026-03-10

**Authors:** Precious I. A. Popoola, Thomas L. Allam, Rebecca J. Lilley, Chandni Manwani, Olivia B. Keers, Junyang Tan, Kylie Yang, Yifan Long, Ewan R. Clark, Lisa J. White, Kira L. F. Hilton, Jennifer Rankin, Jennifer Baker, Charlotte Bennett, Hollie B. Wilson, Evelyn R. Morton, Alvaro Keskküla, Bethany Martin, Christopher O'Connor, J. Mark Sutton, Charlotte K. Hind, Michelle D. Garrett, Cally J. E. Haynes, Jennifer R. Hiscock

**Affiliations:** a University of Kent Canterbury CT2 7NH UK J.R.Hiscock@Kent.ac.uk M.D.Garrett@kent.ac.uk; b School of Chemistry, University of Southampton Highfield Southampton SO17 1BJ UK; c Chemistry Department, UCL 20 Gordon Street London WC1H 0AJ UK cally.haynes@ucl.ac.uk; d School of Science, University of Greenwich Chatham Maritime Kent ME4 4TB UK; e Cancer Research Horizons, Babraham Research Campus Cambridge CB22 3AT UK; f Research and Evaluation, Porton Down, UKHSA Salisbury SP4 0JG UK Charlotte.Hind@UKHSA.gov.uk

## Abstract

Differences in the lipid composition of prokaryotic and eukaryotic cell membranes are well understood and can be exploited to produce novel antimicrobials. However, what is less well recognised is that alteration in the phospholipid composition of the cell membrane is also one of the first phenotypic changes when a cell becomes cancerous. In addition, changes in phospholipid cell membrane composition are a known cause of drug resistance in both microbial disease and cancer. Here we present a novel, next generation series of chiral, amino acid appended supramolecular self-associating amphiphiles that suggest membrane active technologies can be used to produce novel drugs which simultaneously fight against two of the greatest global health threats facing us today, antimicrobial resistant infections and cancer diseases. We demonstrate the antimicrobial and anticancer efficacy of this membrane active amphiphile technology against susceptible and resistant *Staphylococcus aureus* and ovarian cancer cells. We propose a mode of action through a combination of vesicle, NMR spectroscopy and patch clamp experiments, and provide evidence that supports the potential for this class of compound to be developed as pharmaceutical agents against these diseases through *in vitro* drug metabolism and pharmacokinetics experiments alongside *in vivo Galleria mellonella* toxicity experiments.

## Abstract 2

Novel amphiphiles target cell membranes, demonstrating biological efficacy against resistant *S. aureus* and ovarian cancer, with strong mechanistic and DMPK evidence for future therapeutic development.

## Graphical abstract

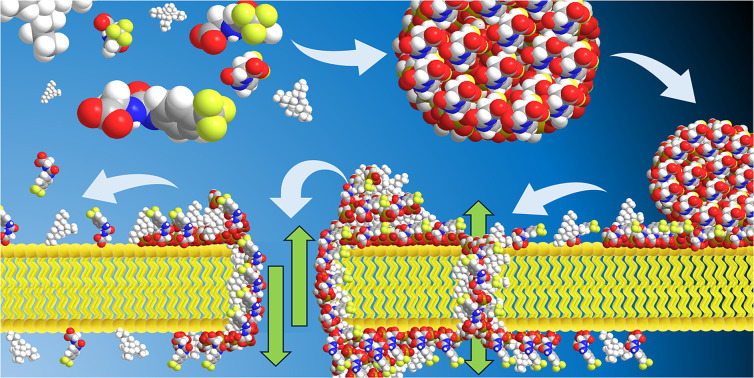

## Introduction

The rise of antimicrobial resistant (AMR) bacterial infections and cancer are two of the greatest health threats facing humanity today. A report generated in 2014 attributed 8.8 million deaths to cancer in that year alone, while simultaneously predicting that by the year 2050, ten million people per year will die from the primary effects of AMR.^1^ The present impact of AMR was confirmed by a recent study, which reported 4.95 million and 1.27 million deaths from the indirect and direct effects of AMR respectively during 2019.^2^ This means that over this time period, AMR was directly responsible for the same number, if not more deaths than those directly attributed to either HIV/AIDs or malaria.^2^ This rise in AMR has traditionally been attributed to the misuse of antimicrobial agents, including antibiotics, across multiple sectors.^3–6^ In addition, although we do not yet truly understand the impacts of the COVID-19 pandemic on increased rate of AMR infections, there was a high level of AMR bacterial co-infections confirmed within patients suffering from COVID-19 over the first 18 months of the pandemic.^7^

Given the serious health threat that AMR poses now and in the future, there has been a focused effort to develop new antimicrobial agents.^8^ This has led to the approval of 12 new antibiotics for use within the clinic (2017–2021), with a further 45 in the clinical development pipeline, 27 of which target WHO priority pathogens.^9^ However, this level of innovation still does not fulfil current or future global need.^8,9^

In 2020, there were an estimated 19.3 million new cancer cases identified and almost 10 million cancer-related deaths worldwide.^10^ There is no doubt that the burden of cancer is growing globally,^10^ due to the aging population, types of risk factors,^11–13^ and rise of resistance to commonly marketed therapeutic agents.^14^ In 2020, 313 959 new ovarian cancer cases were reported globally. The number of ovarian cancer-related deaths in this same year was 207 252 (2.1% of all cancer deaths).^10^ The high mortality figures for ovarian cancer are in part due to the fact that approximately 70% of all diagnoses occur at an advanced stage of the disease, reducing patient survival rates.^15^ The high mortality figures are also due to the common use of platinum and taxane-based chemotherapeutic agents, which often lead to complications and resistance, causing 70% of patients to develop disease recurrence with resistance to these therapeutic agents.^16–18^

Due to the expense associated with developing novel drugs, there is much interest in repurposing currently approved therapies to treat different diseases. Interestingly, a number of antimicrobial agents are reported to have off target activity for the treatment of cancer *e.g.* tetracyclines and fluoroquinolones.^19^ However, as well as providing a novel route for the low cost identification of novel cancer agents, the identification of agents with both antimicrobial and anticancer activity may have other advantages for the treatment of cancer patients.^20^ Microbial infection is a significant cause of morbidity and mortality in cancer patients, due to general immunosuppression including febrile neutropenia, caused by the effect of the cancer on the human body and/or induced by cancer drug treatment, tumour obstruction, or surgery. Of the chemotherapy regimens used to treat ovarian cancer, topotecan, docetaxel, and paclitaxel all place a patient at significant risk of febrile neutropenia.^20^ Therefore, the gold standard treatment here should be one that treats the cancer, treats infectious disease and can overcome resistance in both diseases simultaneously.

**Fig. 1:**
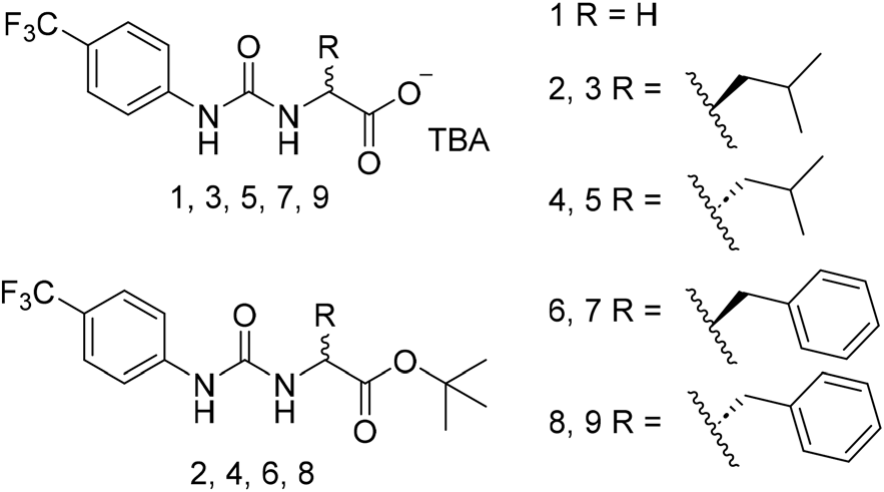
Structures of five amino acid incorporated SSAs (1, 3, 5, 7, and 9) and precursors (2, 4, 6, and 8). Each SSA was prepared as the tetrabutylammonium (TBA) salt.

Supramolecular chemists are making a considerable effort to exploit our understanding of molecular interactions in the production of next-generation approaches to the design of anticancer^21–25^ and antimicrobial technologies.^26–29^ Supramolecular self-associating amphiphiles (SSAs) are a class of amphiphilic salts (Fig. 1) and structurally related compounds that are capable of undergoing intermolecular hydrogen bonding events. These events not only influence SSA self-associative properties and any resultant material formation (hydrogels or spherical aggregate),^30–33^ but also the interaction of this class of compounds with other molecular species, such as cell surfaces,^34–36^ in particular phospholipid membranes.^37–39^ We have previously shown SSAs to demonstrate activity as both antimicrobial agents against Gram-positive methicillin resistant *Staphylococcus aureus* (MRSA) USA300 and Gram-negative *Escherichia coli*,^34^ as well as against A2780 ovarian cancer cells.^36^ In addition, we have shown that members from this family of compounds have a druggable profile when administered intravenously to mice, although Caco-2 permeability studies have suggested to date that SSAs may only be developed as therapeutic agents to be administered intravenously.^39^

Data from previous studies has led us to hypothesise SSAs arrive at the target cell surface as self-associated species, either hydrogel fibres, or more commonly, spherical self-associated aggregates, hydrodynamic diameter (*d*_H_) ≈ 100–550 nm.^30,31,33^ These spherical structures then morph into a coating that covers the surface of the target cells, permeating through the cell membrane,^31,34–36^ with selectivity provided, at least in part, through preferential interactions with polar phospholipid headgroups, present on the extracellular leaflet of the cell membrane.^37–39^ It is principally these phospholipid selective interactions, followed by critical membrane permeation and/or disruption events,^39^ due to increases in SSA concentration at the cell surface, that we believe results in the biological activity observed. Finally, we have also shown that SSAs can act as Na^+/^/K^+^ transporters across synthetic phospholipid membranes when working cooperatively with a known anionophore.^40^ This leads us to explore the possibility that SSAs may also mediate cell death through ion transport, as well as membrane disruption events, depending on the concentration of SSA present at the cell surface.

**Fig. 2:**
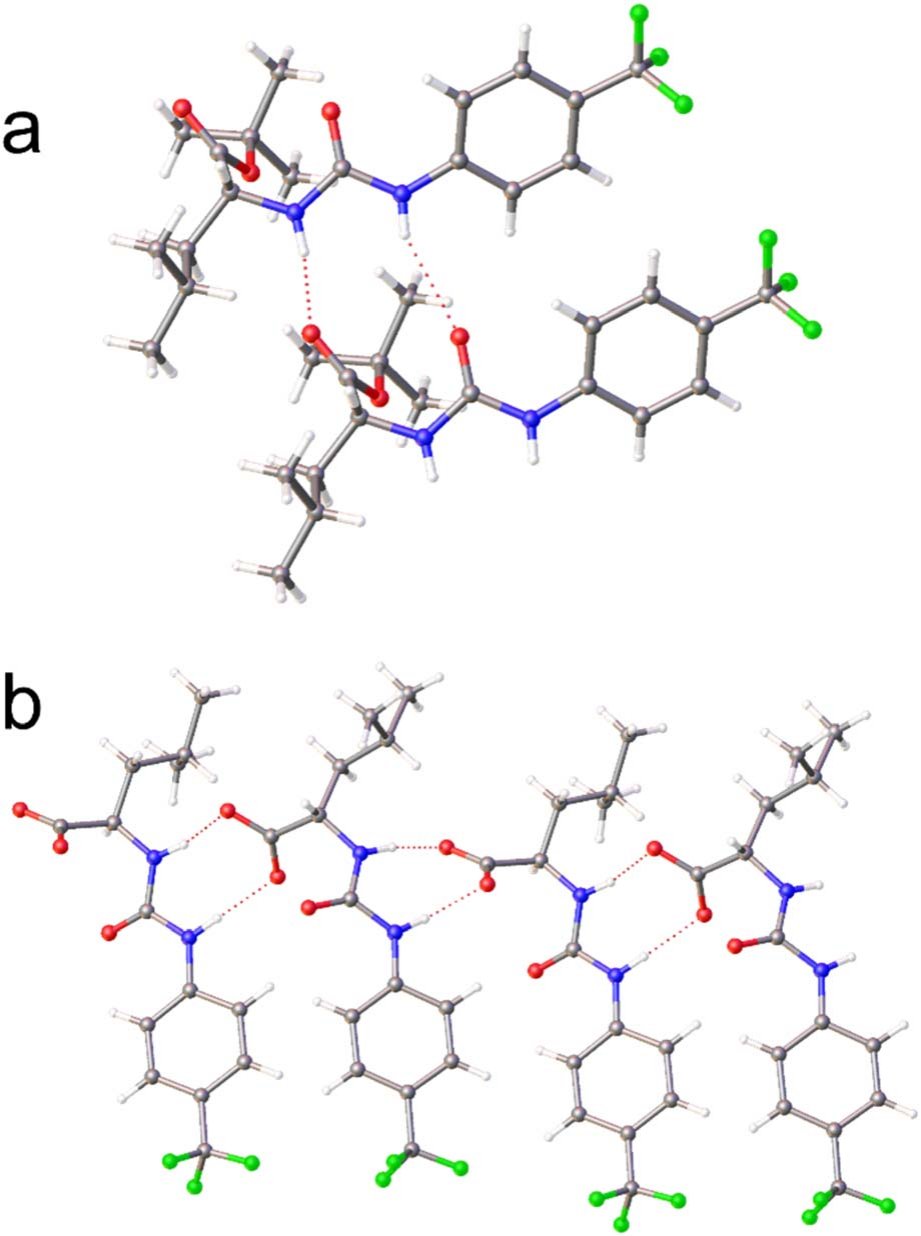
Single crystal X-ray structure of (a) 2 and (b) the 1 : 1 enantiomeric mixture of 3 and 5. The TBA counter cations have been omitted for clarity. Grey = carbon, blue = nitrogen, red = oxygen green = fluorine, white = hydrogen, red dashed lines = hydrogen bonds.††A suitable crystal was selected and mounted on a Rigaku Oxford Diffraction Supernova diffractometer. Data were collected using Cu Kα radiation at 100 K. Structures were solved with the ShelXT^67^ or ShelXS structure solution programs *via* Direct Methods and refined with ShelXL^68^ on Least Squares minimisation. Olex2 (ref. 69) was used as an interface to all ShelX programs. CCDC deposition number for the single crystal X-ray structure shown in Fig. 2a = 2205765 and Fig. 2b = 2218842.

**Fig. 3:**
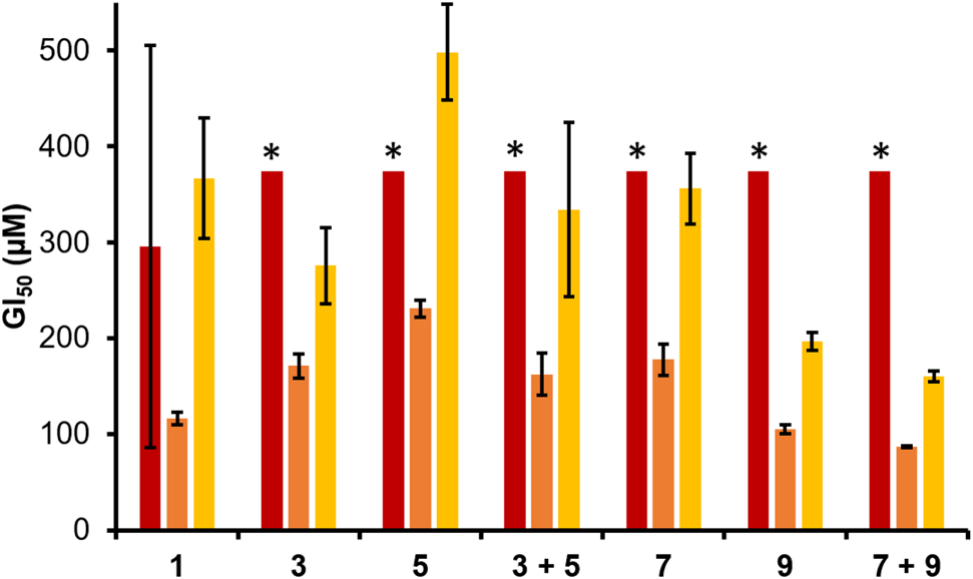
GI_50_ values determined for 1, 3, 5, 7, and 9, and 1 : 1 mixtures of 3 + 5 and 7 + 9 against: epithelial cells (RPE-1 – red); ovarian cancer cells (A2780 – orange) and; cisplatin resistant ovarian cancer cells (A2780 CisR – yellow). Where SSAs are supplied as mixtures, the concentrations reported represent the total molecular concentration. Values obtained from SRB Assays, incorporating data from three biological repeat experiments, each containing three technical repeats, after 96 hours. Please see SI Sections S3 and S19 for further experimental and processed raw data. * Indicates no impact of cell growth at the concentration reported.

Herein, we present a series of four novel next-generation amino acid appended SSAs (Fig. 1 – 3, 5, 7, and 9) designed to selectively increase the activity of this class of compound against drug susceptible and resistant, clinically relevant *S. aureus* and ovarian cancer cells. The design of these next generation SSAs: (i) builds on structure activity relationship analysis, which suggests that the presence of a carboxylate functionality within the structure of the SSA anion increases the strength of preferential intermolecular interactions;^30,34^ (ii) explores the effects of chirality on SSA (host) biological activity, as phospholipid headgroups are themselves chiral species; (iii) use the presence of a hydrophobic amino acid R-group to increase the stability of any SSA transmembrane structures within the hydrophobic portions of the phospholipid bilayer through shielding of the SSA anion hydrophilic moieties (3, 5, 7, 9), and/or introducing π–π stacking interactions (7 and 9).

## Results and discussion

### Synthesis

SSA 1, which acts as a structural control, was synthesised through previously published methods.^30^ Next-generation 3, 5, 7, and 9 were synthesised through the deprotection of 2, 4, 6, and 8, followed by the addition of tetrabutylammonium (TBA). Full details are provided in the SI.

### Physicochemical and self-associative properties

The physicochemical characterisation of SSA systems requires the use of multiple complimentary techniques.^41^ The self-associative properties of the SSAs, alongside their *tert*-butyl protected precursors (Fig. 1) were first explored in the solid state, using single crystal X-ray diffraction techniques. Here, structures obtained from the slow evaporation of solutions containing SSA precursors 2, 4, 6, and 8 demonstrate intermolecular hydrogen bond formation between the urea NH hydrogen bond donor groups to either the urea or ester C=O functionality, as shown in Fig. 2a. However, although homogeneous single crystals of 3, 5, 7, and 9 were not obtained, a single crystal of 3 and 5, supplied as a 1 : 1 enantiomeric mixture, was found (Fig. 2b) showing the anionic SSA enantiomers adopt an urea-anion hydrogen bonded alternating tape.

Moving into solution, the self-associative properties of this sub-class of amino acid appended SSAs were first explored in DMSO-*d*_6_ (Table 1), as within this polar organic solvent, SSAs have been shown to produce hydrogen bonded anionic dimers. This enables the quantification of parameters such as SSA anion dimerization constant which we have shown to correlate with critical aggregation concentration (CAC) values obtained from aqueous solutions of that same SSA.^30^

**Table 1:** Physicochemical data produced to characterise SSA self-association events in a DMSO-*d*_6_ 1% DCM (Q ^1^H NMR spectroscopy) or DMSO-*d*_6_ 0.5% H_2_O (^1^H NMR DOSY or dilution study) solution. Where the SSAs are supplied as an enantiomeric mixture, they are present in a 1 : 1 ratio. Concentrations reported represent total molecular concentration of SSA present. All Q ^1^H NMR spectroscopy experiments were conducted with a delay time (*d*_1_) of 60 s at 298 K and a concentration of 112 mM, and DCM was used as the internal standard. The values given in % represent the observed proportion of compound to become ^1^H NMR spectroscopy silent. The *d*_H_ of the SSA species present in DMSO-*d*_6_ 0.5% H_2_O were obtained through ^1^H NMR DOSY at a total molecular concentration of 112 mM (298 K). *K*_dim_ values are calculated *via*^1^H NMR spectroscopy dilution study, followed by subsequent fitting of these data to the equal *K*/dimerization self-associative binding isotherm model using Bindfit v0.5.^42^ Electrostatic surface potential energy maximum (*E*_max_) and minimum (*E*_max_) values were calculated using Spartan ‘20, while consensus log *P* values were calculated using the Swiss ADME platform^43^

SSA	Q NMR (%)		*d*_H_ (nm)	*K*_dim_ (M^−1^)	*E*_min_ (kJ mol^−1^)	*E*_max_ (kJ mol^−1^)	Consensus log *P*
	SSA anion	SSA cation					
1	0 (ref. 30)	0 (ref. 30)	1.3 (ref. 30)	41 (±1.3%)^30^	−730	−45	2.90
3	0	0	1.7	121 (±1.9%)	−728	−50	4.08
5	0	0	1.7	90 (±3.2%)	−728	−49	4.07
7	0	0	1.7	28 (±2.1%)	−714	−47	4.03
9	0	0	1.7	23 (±2.5%)	−715	−46	4.22
3 + 5	68	69	1.4^a^	a	—	—	—
7 + 9	82	78	0.9^a^	a	—	—	—

a Not calculated due to SSA heterogeneity.

Initially, quantitative (Q) ^1^H NMR spectroscopy experiments were undertaken in DMSO-*d*_6_ 1% DCM, using the DCM as an internal standard to identify the presence of either low or high order SSA self-associated aggregates. Here, low-order SSA self-associated aggregates are defined as those directly observable using traditional solution state ^1^H NMR spectroscopy such as the formation of hydrogen bonded anionic dimers. To determine the presence of low or high-order SSA aggregates, signals corresponding to the anionic or cationic component of the SSA are integrated against those of the internal DCM standard. An apparent ‘loss’ of signal indicates the presence of larger, NMR silent aggregates, which exhibit solid-like properties and are therefore no longer visible using this experimental technique. As summarised in Table 1, at concentrations ≤112 mM, SSAs 3, 5, 7, and 9 form only low order self-associated aggregates in polar organic DMSO-*d*_6_ solutions. This is supported by the results of complimentary ^1^H NMR DOSY experiments, here the *d*_H_ of the SSA anion, was calculated between 0.9-1.7 nm. However, 1 : 1 enantiomeric mixtures of 3 + 5 and 7 + 9 also show the presence of higher order aggregates, supporting the hypothesis that the interaction of SSA anion enantiomers influences molecular self-association events in DMSO-*d*_6_ 1% DCM. We hypothesise that this is driven by the preferential heterogeneous hydrogen bonded complex formation between the SSA anionic components, such as those shown in Fig. 2b. Here, the difference in amino acid side chain was also found to influence the relative proportion of the SSA to become incorporated into these higher order SSA aggregates. We can discount the strength of hydrogen bonding from influencing the increase in the proportion of SSA to become incorporated into these higher order structures at this concentration as the hydrogen bond mediated dimerization constant (*K*_dim_, Table 1) calculated for the SSA anion from homogenous DMSO-*d*_6_ 0.5% H_2_O solutions of 3, 5, 7, and 9 was found to be lower for 7 and 9 when compared to 3 and 5. Instead, we hypothesise that this increase in SSA incorporated into higher order aggregates is due to a combination of favourable π–π stacking interactions and decreased steric hindrance, afforded by the change in amino acid R-group.

Further comparative analysis of these *K*_dim_ values showed the *K*_dim_ calculated for 7 and 9 to be comparable in magnitude to the *K*_dim_ calculated for 1. We reason that this similarity in *K*_dim_ to be due to the effect that the R-group substitution has on hydrogen bond donor/acceptor spatial positioning and the comparative basicity and acidity of the carboxylate and urea functionalities respectively. It has been shown that electrostatic surface potential values, calculated by low level computational modelling programs such as Spartan,^44^ using semi-empirical PM6 molecular modelling methods,^45^ can be used to estimate the hydrogen donor/acceptor activity, and thus estimate hydrogen bonding strength. For the SSA anionic component of 1, 3, 5, 7, and 9, *E*_max_ and *E*_min_ values obtained from electrostatic potential maps were found to correlate with the urea NH and carboxylate residues respectively (Table 1, SI Section S24, Table S20). Comparison of these data reveal *E*_max_ values to remain comparable across the series of SSAs tested. When comparing the *E*_min_ values, 1, 3, and 5 were found to exhibit values similar to each other and lower in magnitude than that of 7 and 9. This difference in *E*_min_ indicates the basicity of these carboxylate groups to be enhanced in the former compared to the latter, which corresponds to the increased strength of *K*_dim_ observed for 3 and 5 over 7 and 9. However, this does not explain the difference in *K*_dim_ observed between 1 and 3 or 5. We believe that this difference is due to the increased steric bulk of 3 and 5 in comparison to 1, which may pre-organise the SSA anion of 3 and 5 towards dimer formation, resulting in increased *K*_dim_ values.

Moving from a polar organic DMSO solution to an aqueous environment, the presence of lower and higher order self-associated SSA aggregates was confirmed using quantitative ^1^H NMR spectroscopy in a D_2_O/5% EtOH solution (EtOH used as the internal standard), at a total SSA concentration of 5.56 mM, as shown in Table 2. This confirmed the presence of higher order aggregates, demonstrating solid like properties for homogenous solutions of 1, 3, 5, 7, and 9. As expected, the proportion of the SSA incorporated into higher order aggregates remained comparable for homogenous solutions of both enantiomers, with no significant difference in the proportion of SSA to be incorporated into the higher order aggregates of 3, 5, 7, and 9. However, when supplied as a 1 : 1 enantiomeric mixture, although the trend in increased incorporation of 7 + 9 over 3 + 5 into the higher order aggregate structure was retained from previous observations in polar organic solvents, the total proportion of SSA incorporated into these higher order structures was approximately 20% lower (from ≈ 50% to ≈ 30%) for the 1 : 1 enantiomeric mixture of 3 + 5 over the homogenous solution of those same SSAs. This supports the hypothesis that, as observed within DMSO-*d*_6_ 1% DCM solution (Table 1), the combination of SSA enantiomers influences SSA self-association events, causing the amino acid R-group to play a greater role in influencing higher order SSA aggregation events.

**Table 2:** Physicochemical data produced to characterise SSA self-association events in a H_2_O 5% EtOH or D_2_O 5% EtOH (^1^H NMR spectroscopy only) solution. Where the SSAs are supplied as an enantiomeric mixture, they are present in a 1 : 1 ratio. Aggregate stability and *d*_H_ were obtained *via* zeta potential and DLS measurements respectively, at a concentration of 5.56 mM and a temperature of 298 K, following an annealing process. The *d*_H_ of the aggregates listed were obtained from intensity distribution peak maxima. CAC was derived at approximately 291 K from surface tension measurements. All Q ^1^H NMR spectroscopy experiments were conducted with a delay time (*d*_1_) of 60 s at 298 K and a concentration of 5.56 mM, EtOH was used as the internal standard. The values given in % represent the observed proportion of compound to become ^1^H NMR spectroscopy silent. The slight difference in anion: cation ratio is due to increased experimental error due to decreased SSA concentration

SSA	Q NMR (%)		*d*_H_ (nm)	Error	PDI	Error	Zeta potential (mV)	CAC (mM)	Surface tension (mN m^−1^)
	SSA anion	SSA cation							
1	68 (ref. 30)	a 30	220 (ref. 30)	a 30	a 30	a 30	−37 (ref. 30)	11.2 (ref. 30)	39.33 (ref. 30)
3	50	44	266	19	0.038	0.0028	−55 (±3.64)	b	40.92^c^
5	52	48	174	13	0.033	0.0042	−58 (±1.50)	b	40.92^c^
7	51	48	167	19	0.029	0.0150	−58 (±1.64)	b	39.83^c^
9	58	53	181	53	0.026	0.0120	−66 (±0.78)	b	39.64^c^
3 + 5	31	33	238	3	0.047	0.0034	−57 (±0.65)	b	38.67^c^
7 + 9	56	51	146	14	0.019	0.0025	−65 (±0.31)	b	39.25^c^

a Data not available.

b Solubility prevented data acquisition.

c Data collected at limit of SSA solubility = 5.56 mM.

The size and stability of those self-associated structures present were confirmed through a combination of dynamic light scattering (DLS) and zeta potential measurements respectively (Table 2). The *d*_H_ of the higher order species present at 5.56 mM, calculated from intensity distributions, were found to exhibit similar properties to those SSA self-associated species previously published.^30,34^ The aggregated species produced from 3, 5, 7, 9, and their 1 : 1 enantiomeric mixtures under these experimental conditions exhibited zeta potential values between −66 to −55 mV, confirming the stability of the self-associated species present to be greater than those of 1 at the same molecular concentration. Therefore, the presence of the alkyl/aromatic R-groups within the amino acid appended SSA are confirmed to increase higher order self-associated aggregate stability.

Here, the CAC is defined as the concentration at which any additional SSA added to a solution will result in the production of high-order self-associated species. However, this does not mean that these larger self-associated aggregates do not exist in solution below the CAC; these species will instead exist in equilibria with those species present at the solution interface.^46^ To determine CAC, we used the pendant drop method to elucidate the surface tension of H_2_O 5% EtOH solutions of 1, 3, 5, 7, 9, and 1 : 1 SSA enantiomeric mixtures at 291 K (Table 2). Although the Q ^1^H NMR spectroscopy, DLS, and zeta potential measurements confirmed the presence of higher order aggregated species at 5.56 mM, and a decrease in surface tension with respect to increasing SSA concentration confirmed surfactant properties in all cases, a CAC could not be determined for solutions of 3, 5, 7, or 9 due to compound solubility.

Preliminary DFT assessment of the interaction of 7 and 9 with model phospholipids predicts that all such interactions are favourable, and significant differences in energy of interaction between enantiomers, supporting the hypothesis that chiral modification provides a route to tuning SSA activity (see SI Section S25).

### Biological activity

SSAs have been previously reported to exhibit weak antimicrobial properties against both Gram-positive clinically relevant *S. aureus* – MRSA USA300 and Gram-negative *E. coli* DH10B bacteria.^34^ Next generation SSAs 3, 5, 7, and 9 were specifically designed to increase the activity of SSAs against both drug susceptible and resistant *S. aureus* and ovarian cancer cells. Table 3 reports the MIC values obtained for previously reported 1 and novel SSAs 3, 5, 7, and 9, provided as single agents or 1 : 1 enantiomeric mixture against four different strains of *S. aureus*. These strains include: (i) USA300, a common clinically relevant, methicillin resistant strain;^47^ (ii) ATCC 9144, a fully genome sequenced, β-lactamase negative and penicillin susceptible strain;^48^ (iii) NCTC 13616, a clinically relevant methicillin resistant strain;^49^ (iv) 1199B, which over expresses the NorA efflux pump^50,51^ that has a wide range of substrates including fluoroquinolone antibiotics, antiseptics, and quaternary ammonium compounds, causing this strain to be classed as multidrug resistant.^51^

**Table 3:** MIC values (µM) determined for 1, 3, 5, 7, and 9, and 1 : 1 enantiomeric mixtures of 3 + 5 and 7 + 9 against four different *S. aureus* strains: ATCC 9144; NCTC 13616; USA300; 1199B. Where SSAs are supplied as mixtures, the concentrations reported represent the total molecular concentration. The values calculated represent absolute values, obtained from two biological repeat experiments, each containing three technical repeats, after 900 min. Please see SI Section S18 for MIC data, specifically summary Table S15. MIC of ciprofloxacin (CP) is provided in µg mL^−1^

SSA	ATCC 9144	NCTC 13616	USA300	1199B
1	1390	>2780	>2780	>2780
3	87	87	>2780	87
5	174	87	>2780	2780
7	87	87	87	174
9	174	87	87	174
3 + 5	174	87	174	174
7 + 9	87	87	87	87–349
CIP	1	128	64	8

Prior to this study, the lowest MIC_50_ (the minimum amount of a compound required to inhibit bacterial growth by 50%) reported for an SSA against MRSA USA300 was 0.25 mM.^34^ Here, 7, 9, and the 1 : 1 enantiomeric mixture of 7 and 9 exhibited an MIC (a complete absence of any detectable bacterial growth) of 87 µM, against this same strain of *S. aureus*, making these by far the most active SSA antimicrobial agents reported against MRSA USA300 to date. When compared to 1 (the glycine appended SSA where R = H), the substitution of this R-group for either an isobutane (3 and 5, leucine) or a benzyl (7 and 9, phenylalanine) derivative resulted in an overall enhancement of antimicrobial activity. This activity is hypothesised to be due to the additional hydrophobicity and/or π–π stacking interactions provided by these functionalities, increasing the membrane permeability properties of these agents, alongside alteration in the strength of molecular self-association interactions (3 and 5, Table 1), in particular the formation of hydrogen bonded SSA anion complexes as previously described.^30,33^ Interestingly, the presence of natural l-amino acids within the SSA structure (3 and 7), led to an enhanced activity against the methicillin susceptible ATCC 9144 strain, over their D-substituted counterparts (5 and 9). MIC values determined for the different SSA enantiomers support the hypothesis that these SSAs are interacting with a chiral target (*e.g.* specific phospholipid headgroups). An enhancement in activity where there is a 1 : 1 ratio of enantiomers present suggests that an alteration in SSA self-associative complex and/or high-order aggregate formation (see Fig. 2, Tables 1 and 2) also contributes to enhanced antimicrobial activity, *e.g.* through stabilisation of cell membrane permeation events. This is exemplified when comparing the antimicrobial activity of the 1 : 1 enantiomeric mixture of 3 and 5 against USA300 (MIC = 174 µM), to that of either 3 or 5 alone (MIC = 10 mM). Finally, the antimicrobial activity of 3, 7, and 9 against 1199B, provides supporting evidence towards the hypothesis that the SSA anion is integral to the activity of these SSAs, as this strain of *S. aureus* overexpresses the NorA efflux pump, which specifically targets quaternary ammonium species, such as TBA, the SSA counter cation. This hypothesis is further supported through the change in antimicrobial efficacy of 1, 3, 5, 7, and 9, which is a direct result of the modification in structure of the SSA anionic component. Attempts were made to confirm the presence of channel/pore-like structures through both TEM and SEM measurements, however results of these studies proved inclusive. This is not surprising as these structures are believed to be dynamic in nature, as is suggested by the results presented within the mode of action section of article. Examples of the data collected within the scope of SEM studies are included within Section S27.

We then moved to establish the efficacy for this series of SSAs and their 1 : 1 enantiomeric mixtures towards cisplatin sensitive and resistant ovarian cancer cells. We have previously determined the GI_50_ (the concentration of a compound needed to inhibit 50% of cellular growth) values for a series of sulfonate appended SSAs against cisplatin sensitive A2780 ovarian cancer cells.^36^ Here GI_50_ values between 45 ± 19 µM and 451 ± 38 µM were reported. As summarised in Fig. 3, the GI_50_ values recorded for this series of carboxylate SSAs range from 87 ± 1 µM (7 and 9) to 231 ± 9 µM (5), showing the carboxylate based SSAs to report similar activities against this cancer cell line as the sulfonate SSAs. Here 9, and the 1 : 1 enantiomeric mixture of 7 and 9, demonstrated the greatest activity against A2780 ovarian cancer cells. This indicates anticancer activity to be influenced by the amino acid R-group present, chirality and SSA self-associative complexation processes, as previously observed when establishing the antimicrobial activity of these same agents. These observations are retained when investigating the activity of these same SSAs against the cisplatin resistant A2780 CisR cell line. Therefore, 9 shows the greatest anticancer activity against both cisplatin susceptible and resistant cancer cell lines, with an increased efficacy demonstrated when supplying 7 and 9 as a 1 : 1 enantiomeric mixture wherein the effective concentration of 9 present is decreased by 50%. This suggests, as observed when supplying chiral SSAs as antimicrobial agents, that this increase in efficacy could be due to alteration in, or stabilisation of SSA self-association events and/or resultant aggregate structure influencing molecular membrane permeation events.

To gauge the selectivity of SSAs for ovarian cancer cells over non-cancerous human cells, we attempted to obtain GI_50_ values for 1, 3, 5, 7, 9, and 1 : 1 enantiomeric mixture of 3 + 5 and 7 + 9 against the RPE-1 non-cancerous human epithelial cell line (Fig. 3, Section S19). The RPE-1 cell line is widely used in research as a non-cancerous/non-transformed epithelial cell line. It is used here as a comparator, as the ovarian cancer cells are also of epithelial origin. Where there was no impact on cell growth at 350 µM for an SSA/SSA mixture, no effort was made to further derive a GI_50_ value as this observation confirms a ≥two-fold selectivity for the cancer cells over the RPE-1 cells for the most active SSA (9) and 1 : 1 SSA mixture (7 + 9). These data show the incorporation of the benzyl and isopropyl functionalities within chiral SSAs 3, 5, 7, and 9 to increase selectivity against cancerous over non-cancerous cell lines. Of these chiral SSAs, 9 and the 1 : 1 enantiomeric mixture of 7 and 9, containing an additional benzyl functionality, demonstrated the greatest degree of selectivity for the cancer cells over the RPE-1 cells, while the presence of the amino acid glycine within the SSA structure removed selectivity for the cancer cells over the RPE cells.

### Mechanism of action

We have previously confirmed SSAs to selectively interact, permeate and/or lyse phospholipid membranes of differing compositions, depending on the chemical composition of the anionic component of this amphiphilic salt.^37–39^ We have also shown an SSA to act as a potassium or sodium phospholipid membrane transport agent when working cooperatively with a known anionophore.^40^ These findings are further supported by a series of fluorescent live cell microscopy studies, in which intrinsically fluorescent SSAs can be observed forming spherical aggregates, which then interact with a target cell surface, morphing into a coating, and then permeating into the cell, enabling the entry of other molecules previously unable to gain cellular entry.^31,34–36^

To confirm selectivity of the SSA anionic component for the phosphate species contained within the phospholipid headgroups over other biologically relevant anionic species, a series of ^1^H NMR spectroscopy titrations were performed in DMSO-*d*_6_ 0.5% H_2_O. Here H_2_PO_4_^−^ was used to model the phosphate present in the phospholipid headgroup, while Cl^−^ was incorporated into these studies as a common biologically relevant anion. However, these data should be treated with caution, due to the strength of the SSA anion self-association events present, see Table 1. In those instances where evidence of a hydrogen bonded SSA host:guest complex could be observed, the data was fitted to a 1 : 1 binding isotherm model using Bindfit v0.5 (Section S16).^42^ Here, we obtained limited data supporting the formation of a 1 : 1 host : guest complex, with an enhanced strength of interaction noted for H_2_PO_4_^−^ over Cl^−^ (*e.g.* TBACl:7*K*_ass_ = 0 M^−1^; TBAH_2_PO_4_:7*K*_ass_ = 30 M^−1^ ± 7.5%).

**Fig. 4:**
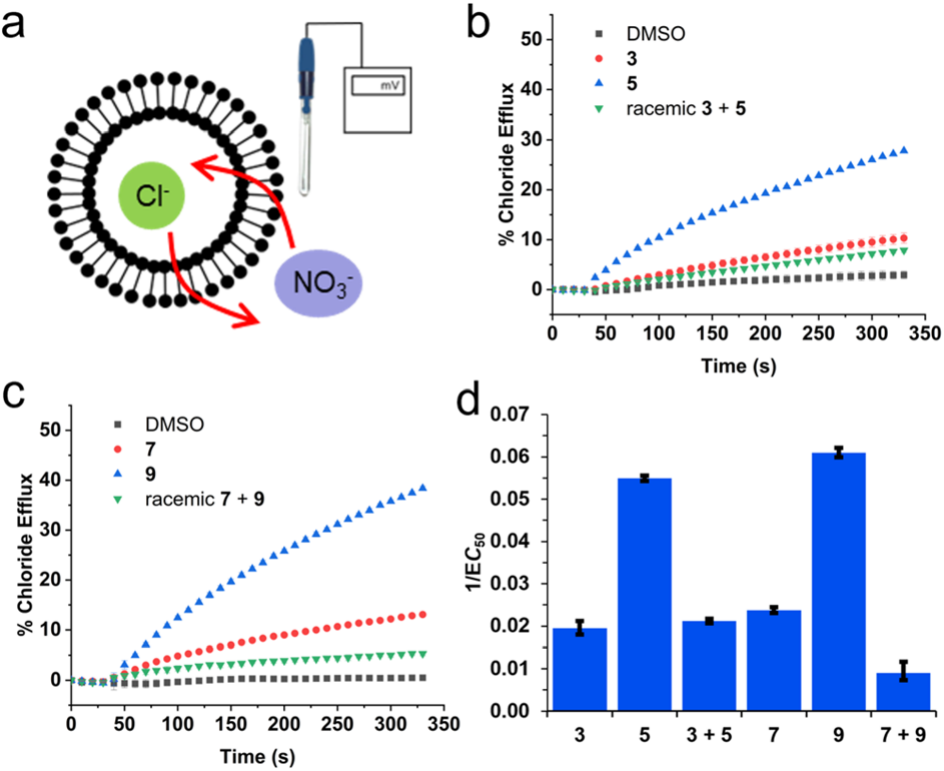
(a) Schematic representation of the Cl^−^/NO_3_^−^ antiport assay;^52^ (b) chloride efflux mediated by 3, 5, and 3 + 5 (10 mol% w.r.t. lipid); (c) chloride efflux mediated by compounds 7, 9, and 7 + 9 (10 mol% w.r.t. lipid); (d) an overview of the reciprocal of the EC_50_ values for each compound and racemic mixture to visualise a comparison of the overall efficacies. Where multiple SSAs are supplied in combination, they are supplied as a 1 : 1 enantiomeric mixture with values reported representing total SSA concentration.

Although evidence supports the hypothesis that SSAs are able to act as selective phospholipid membrane coordination and/or disruption agents,^39^ to date we have minimal evidence that verifies the role ion transport events may play in the SSA mechanism of biological activity, or the types of transmembrane SSA structures which may be involved in facilitating these events, such as ion channels or membrane pores. Therefore, to confirm the role ion transport may play in the biological activity of SSAs, we explored the ability of SSAs 1, 3, 5, 7, and 9 to mediate ion transport in phospholipid vesicles (Section S17). We initially performed a Cl^−^/NO_3_^−^ antiport assay.^52^ Large unilamellar vesicles composed of 1-palmitoyl-2-oleoyl-sn-glycero-3-phosphocholine (POPC) were prepared containing NaCl (ionic strength = 500 mM) buffered to pH 7.2 with sodium phosphate salts. The vesicles were suspended in NaNO_3_ (ionic strength = 500 mM) at pH 7.2 (Fig. 4a). On addition of the SSA as a 5 mM DMSO stock solution (which was maintained throughout these experiments to minimise any variation in aggregation state induced by changes in concentration), any resulting chloride efflux was detected using a chloride ion selective electrode (ISE). All of these molecules but compound 1 (Section S17) mediated a modest amount of chloride efflux when added at 10 mol% w.r.t. lipid (Fig. 4b and c)which is surprising given the negative charge of the SSA anion. Comparable experiments in which the external anion was replaced with SO_4_^2−^ resulted in greatly reduced chloride efflux, indicating a likely anion antiport mechanism. Introducing a pH gradient into the Cl^−^/SO_4_^2−^ vesicle system also did not result in improved chloride efflux. However, additional transport experiments using the pH sensitive probe HPTS have been communicated elsewhere indicated that these molecules can mediate an electroneutral H^+^/Cl^−^ transport mechanism; hence, the apparent Cl^−^/NO_3_^−^ antiport activity observed here could be the net result of the exchange of HCl and HNO_3_ across the bilayer.^53^ We were unable to observe significant transport activity when these compounds were added as a stock solution dissolved in water (5 mM).

Hill plot analyses were used to quantify the efficacy of Cl^−^/NO_3_^−^ antiport, generating comparable values of EC_50_ (the concentration of transporter required to mediate 50% chloride efflux after 270 s – Section S17). The reciprocal of the EC_50_ values are summarised in Fig. 4d, to visualise a comparison of the overall efficacies, with the most active compounds exhibiting the largest 1/EC_50_ values. From these data, we observe that 3, 5, 7, and 9 are modest anion antiporters (EC_50_ > 15 mol% w.r.t. lipid in each case). SSA 9 was the most active transporter within this series, and we observed two major trends: (i) in each case, the SSAs containing unnatural d-amino acids (5 and 9) are more active than their L-counterparts (3 and 7; (ii) the transport efficacy of the 1 : 1 enantiomeric mixture were significantly reduced compared to the most active enantiomer of each pair. We speculate that this reduced activity could result from differences in self-associative processes adopted by the SSA enantiomeric mixtures when compared to the homogenous aggregates produced by a single SSA as evidenced by Q ^1^H NMR spectroscopy experiment (Tables 1 and 2). Further analysis of this class of compound to act as ion transporters and phospholipid membrane lysis agents against a range of biologically relevant vesicles is included within a second article, which builds upon the results supplied herein.^53^

There is a literature president for both synthetic and naturally occurring amphiphiles to act as phospholipid membrane ion chennels^54–59^ or pores.^60–63^ Evidence to support the potential for SSAs to form ion channel or pore-like structures was obtained *via* planar bilayer patch clamp experiment, using a semi-automated Port-a-Patch system (Nanion), keeping experimental conditions as similar as possible to those used within the vesicle transport assays. Some differentiation between the methodologies was necessary to retain planar phospholipid bilayer stability. While the ionic strength was retained at 500 mM and SSAs were supplied in a DMSO solution, POPC was replaced by 1,2-diphytanoyl-sn-glycero-3-phosphocholine (DPhPC) – 10% cholesterol, the pH was maintained at 5.5, NaCl was substituted for KCl and NaNO_3_ was substituted for Na_2_SO_4_. Differences in the composition of the solutions present either side of the planar lipid bilayer were achieved through buffer exchange processes post planar bilayer formation, Section S3 and S22.

Initial experiments explored the effects of SSA concentration and change in SSA molecular structure/composition on the flow of ions across the planar bilayer. These initial experiments were performed with a holding voltage of +100 mV. Each experiment was conducted a minimum of three times to ensure experimental reproducibility and event capture. In these experiments set concentrations (0.10 mM, 0.25 mM, 0.38 mM, 0.50 mM, and 1.00 mM) of SSA, or SSA 1 : 1 enantiomeric mixture, were applied to the surface of the phospholipid bilayer at time = 0 seconds. Change in current across the phospholipid bilayer was then recorded with respect to time.

**Fig. 5:**
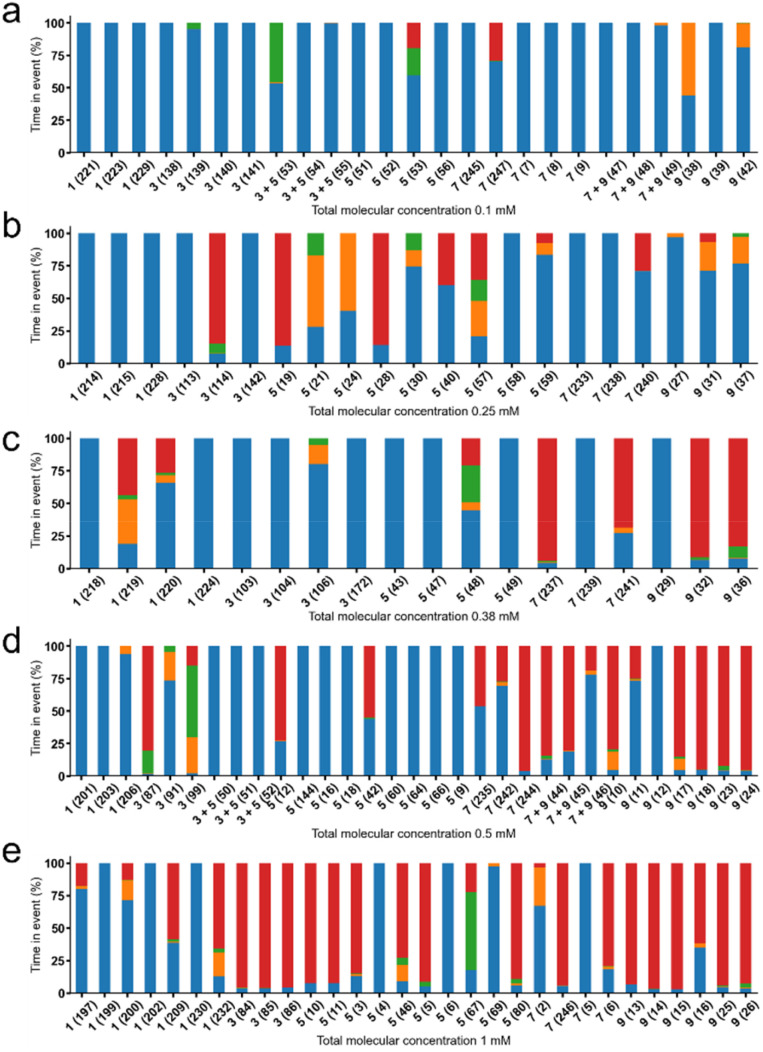
Summary of time spent in different event states upon the addition of 1, 3, 5, 7, 9, 3 + 5 (1 : 1), 7 + 9 (1 : 1) at: (a) 0.10 mM, (b) 0.25 mM, (c) 0.38 mM, (d) 0.50 mM and (e) 1.00 mM. Events were categorised as follows (current, A) 0: −∞ A ≤ Event 0 (blue) < 5.00 × 10^−11^ A, 1 : 5.00 × 10^−11^ A ≤ Event 1 (orange) < 5.00 × 10^−10^ A, 2 : 5.00 × 10^−10^ A ≤ Event 2 (green) < 4.90 × 10^−8^ A, 3 : 4.90 × 10^−8^ A ≤ Event 3 (red) < ∞ A. Experiments were conducted over a 1000 s ± 5 s unless stated in Table S19. For ease of reference to raw and partially processed data the experiment numbers are given in brackets within this figure. Planar phospholipid bilayers formed were formed from DPhPC 10% cholesterol, with Buffer A sealed below, and Buffer B accessible above the bilayer. Buffer A (KCl (489 mM), NaOAc (5 mM), pH 5.5, ionic strength = 500 mM); B (Na_2_SO_4_ (167 mM), NaOAc (5 mM), pH 5.5, ionic strength = 500 mM). Stock solutions of SSA(s) were prepared as a DMSO:buffer B 1 : 50 mixture. Control experiments confirmed the membrane to retain stability in the presence of DMSO at these concentrations. All experiments were conducted with a holding voltage +100 mV.

These experiments show a variety of membrane ion transport events to occur, which were first categorised by event type as defined by both current magnitude and time spent in that event state. Fig. 5 summarises the time (%) that each system spends in each event state over the course of the experiment. The experiment lasted approximately 1000 s unless detailed in Table S19. From the summary of these data, as the concentration of SSA is increased, the percentage of time spent in each event window shifts from Event 0, Event 1, Event 2 and through to Event 3, characterised by ampere (A). We hypothesise that increase in event magnitude and time spent in that event window are a result of increasing local SSA concentration at the surface of the planar bilayer, in addition to alterations in SSA structure and self-associative properties. As these SSAs likely exist as spherical aggregates under experimental conditions, it is likely that should an aggregate encounter the membrane, it would instantly result in a dramatic increase in local SSA concentration at the membrane surface, which influences experimental reproducibility, particularly at lower SSA concentrations.

**Fig. 6:**
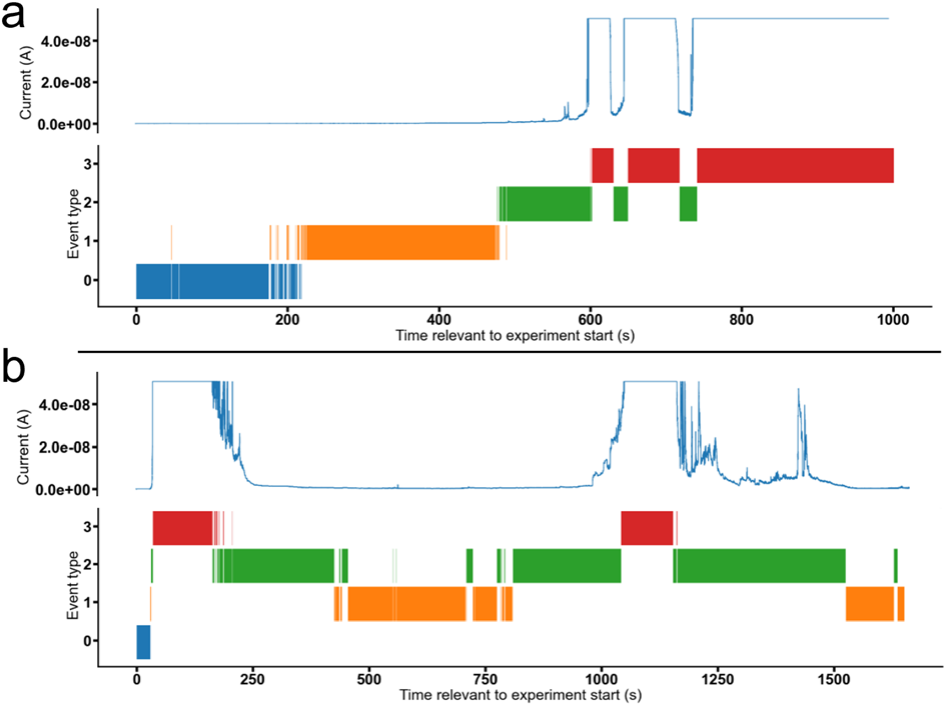
Data summary for patch clamp experiments conducted with a holding voltage of +100 mV for: (a) 5 (0.25 mM), experiment id 57; and (b) 3 (0.50 mM), experiment id 99. Events were categorised as follows (current, A) 0: −∞ A ≤ Event 0 (blue) < 5.00 × 10^−11^ A, 1 : 5.00 × 10^−11^ A ≤ Event 1 (orange) < 5.00 × 10^−10^ A, 2 : 5.00 × 10^−10^ A ≤ Event 2 (green) < 4.90 × 10^−8^ A, 3 : 4.90 × 10^−8^ A ≤ Event 3 (red) < ∞ A.

In general, 1 exhibits the weakest activity, with 7 and 9, supplied as either single agents or as a 1 : 1 enantiomeric mixture, shown to cause the greatest change in current, except at a concentration of 0.25 mM, where 3 and 5 show greater activity. The increased activity of 7 and 9 within these planar bilayer patch clamp experiments correlate with the general increased activity of these SSAs as both anticancer and antimicrobial agents. Interestingly, although there may be a time dependent, sequential movement through Event type (Fig. 6a), for most experiments conducted this is not the case, as demonstrated in Fig. 6b. Here SSA induced change in current with respect to time, is also shown to be reversable. This leads us to propose the following hypothesis: A critical concentration of a particular SSA is required to induce any change in current, due to membrane disruption events. As the local concentration of SSA at the surface of, or permeating into the membrane, increases, the degree of membrane disruption increases, resulting in an increase in current. Using this hypothesis, a decrease in current with respect to time would also indicate a time dependent decrease in local concentration of SSA present at the surface of, or within the phospholipid bilayer. These membrane disruption events, resulting in both increases and decreases in current with respect to time, may also report on the local concentration of SSA present on/in the phospholipid membrane, providing evidence for the flow of SSAs from one side of the phospholipid bilayer to the other, and then away from the bilayer into the bulk solution. This process decreases the effective concentration of an SSA at/in the phospholipid bilayer, enabling the membrane to ‘heal’, resulting in a decrease in current observed with increasing time.

**Fig. 7:**
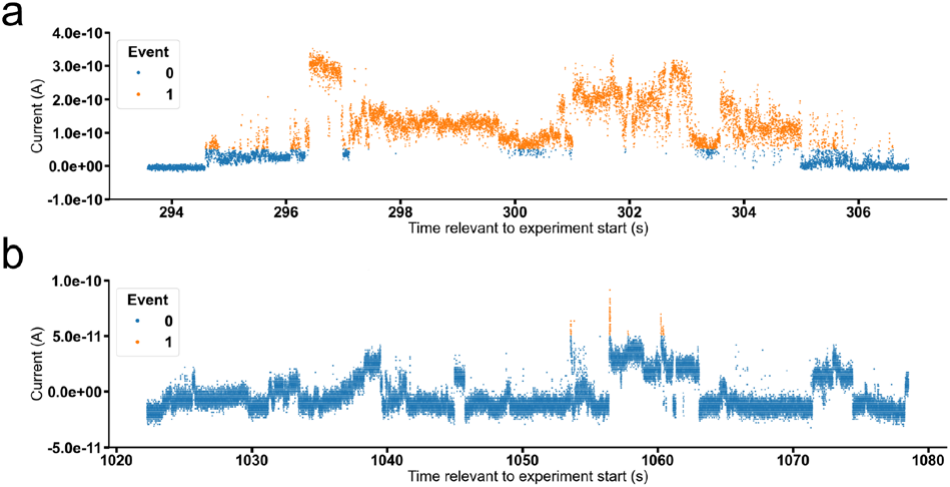
Graphs showing current (A) *vs.* time (s) for: (a) 5 (0.25 mM), experiment id 59 at a holding voltage of +100 mV and (b) 9 (0.1 mM), experiment id 42 at a holding voltage of +100 mV.

**Fig. 8:**
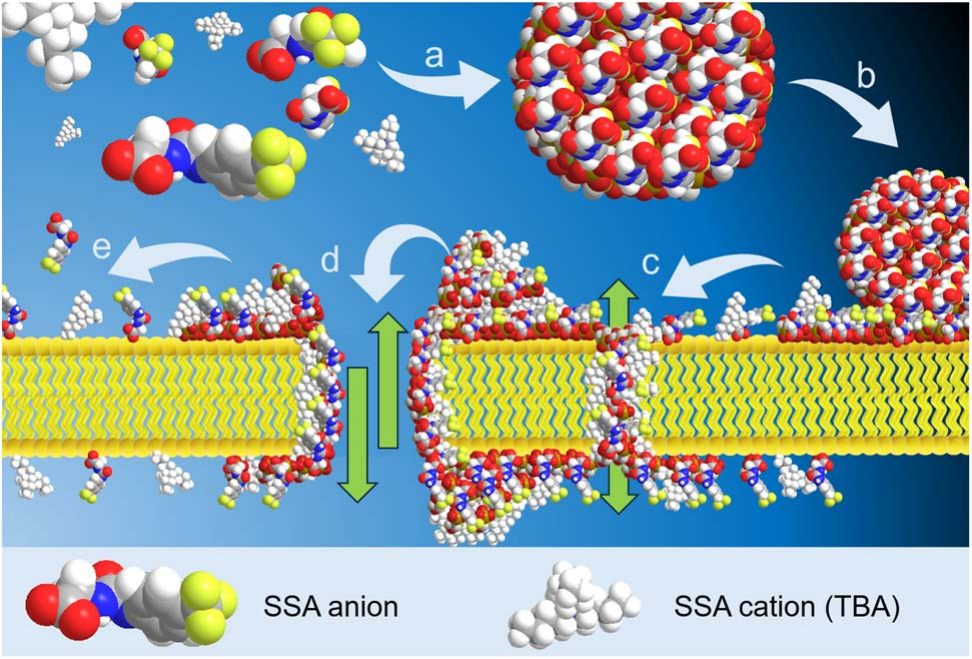
Cartoon summarising the hypothesised effect of SSAs on target phospholipid membranes. (a) The SSA self-assembles under aqueous conditions to form a spherical aggregate. (b) The spherical aggregate arrives at the surface of the target cell membrane. Upon adhesion, the SSAs begin to distribute themselves across the outer leaflet of the phospholipid bilayer. (c) Upon optimisation of preferential SSA:phospholipid headgroup interactions and increase in local concentration of the SSA, the SSA units now self-associate, producing structures that are able to permeate the phospholipid bilayer and display ‘channel/pore-like’ behaviour. However, these structures are not static as the SSA continues to diffuse through the membrane, increasing the local concentration of the SSA on the inner leaflet of the phospholipid membrane and/or diffusing away from this structure into the cell cytoplasm. (d) As the local concentration of SSA present increases still further, greater disruption to the phospholipid bilayer is observed, increasing ion flow across this structure. (e) As the local concentration of SSA present at the outer or inner leaflet of the phospholipid membrane is reduced, the SSA is no longer able to disrupt the membrane or facilitate the movement of ions, and the phospholipid membrane repairs itself.

Further analysis of these data enabled the identification of ion channel/pore-like behaviour, two examples of which are provided in Fig. 7. These data, extracted from different patch clamp experimental data sets support the hypothesis that SSAs can form organised ion channel/pore-like structures that transverse the phospholipid bilayer, facilitating the movement of ions. Although demonstrating events greater in magnitude than the majority of synthetic or naturally occurring ion channels/pores, repetitive step changes in current can clearly be identified, a typical characteristic for the opening/shutting of single ion channels/pores. Numerous examples of this ion pore/channel-like behaviour were identified in the wider SSA patch clamp data set, some of which have been further detailed within the SI (Section S22). A cartoon summarising these processes is provided in Fig. 8 and bears some similarity to the carpet model a mode of action adopted by antimicrobial peptides to permeabilize the membranes of microbial cells. Here the antimicrobial peptides ‘carpet’ the surface of the membrane, and at higher concentrations permeate through the cell membrane.^64^ This similarity is further highlighted by the results of a sister study, in which the ability of an SSA to lyse or interact with phospholipid vesicle membranes of differing compositions was found to be concentration, SSA aggregate composition and SSA structure dependent.^65^

### Drug metabolism and pharmacokinetics (DMPK)

To determine the potential for this sub-class of chiral, amino acid appended SSAs to transition into the clinic, a range of *in vitro* DMPK studies have been performed. A similar range of studies had previously been undertaken for 1,^39^ however, here we select 9 as a representative agent for the four chiral SSAs detailed herein, as this SSA demonstrated high levels of ion transport, antimicrobial and anticancer activity. Kinetic solubility studies conducted in PBS buffer at a pH of 7.4 confirmed the maximum of solubility of 9 is 271 µM (Table S25). This indicates that solubility should not become a limiting factor for the translation of this sub-class of SSAs during further optimisation into the clinic.

Following this, the metabolic stability of 9 was determined against mouse, rat, and human liver microsomes. Microsomes contain membrane bound enzymes, such as cytochrome p450, which contribute to first pass metabolism effects, to which a drug must exhibit some resilience. Within this assay the stability of the intact parent compound is monitored with respect to time, during incubation at 37 °C in the presence and absence of the co-factor NADPH – required for cytochrome p450 activity. From these data, the intrinsic clearance (CL_int_) of the compound can be calculated. The lower this value, the more stable the compound is to the actions of these membrane bound metabolic enzymes. The results of this assay showed 9 to have a CL_int_ < 7.50 µL min^−1^ g^−1^ of liver for mouse, rat and human microsomes, considered low by industrial standards (Table S26).

In addition, a human plasma protein binding (PPB) assay was performed to determine the fraction unbound (Fu) of 9. This allows an assessment of the free drug concentration available to distribute into the tissues and interact with the target. The extent to which a compound binds to the constituents of blood plasma can influence drug distribution. This assay was performed after six hours incubation time, after which 98.3% of 9 was recovered (Table S24). At this time, 99.7% of 9 was bound to the proteins present in the plasma, with 0.3% of this agent remaining free, corresponding to a very highly bound compound (Table S24).

Next, to gauge the suitability of SSAs for oral dosing, 9 was subjected to a Caco-2 permeability assay which is used to both predict human intestinal permeability and investigate drug efflux, predicting *in vivo* compound absorption. This assay allows assessment of a compound's ability to undergo transport from the apical to the basolateral (A–B) compartments of the cell monolayer and *vice versa* (B–A). Similarly to previously tested SSAs, including 1,^39^9 was confirmed to poorly permeate the monolayer in the A–B direction (permeability coefficient – *P*_app_ (A–B) = 0.17 × 10^−6^ cm s^−1^), however high permeability in the B–A direction was observed (*P*_app_ (B–A) = 16.76 × 10^−6^ cm s^−1^) (Table S28). This data suggests that the compound is intrinsically permeatable but is likely to be an efflux substrate, based on the poor permeability observed with drug transporters present. This is likely to result in poor absorption when dosed orally and therefore further development would be necessary for these agents to become suitable for oral administration.

**Fig. 9:**
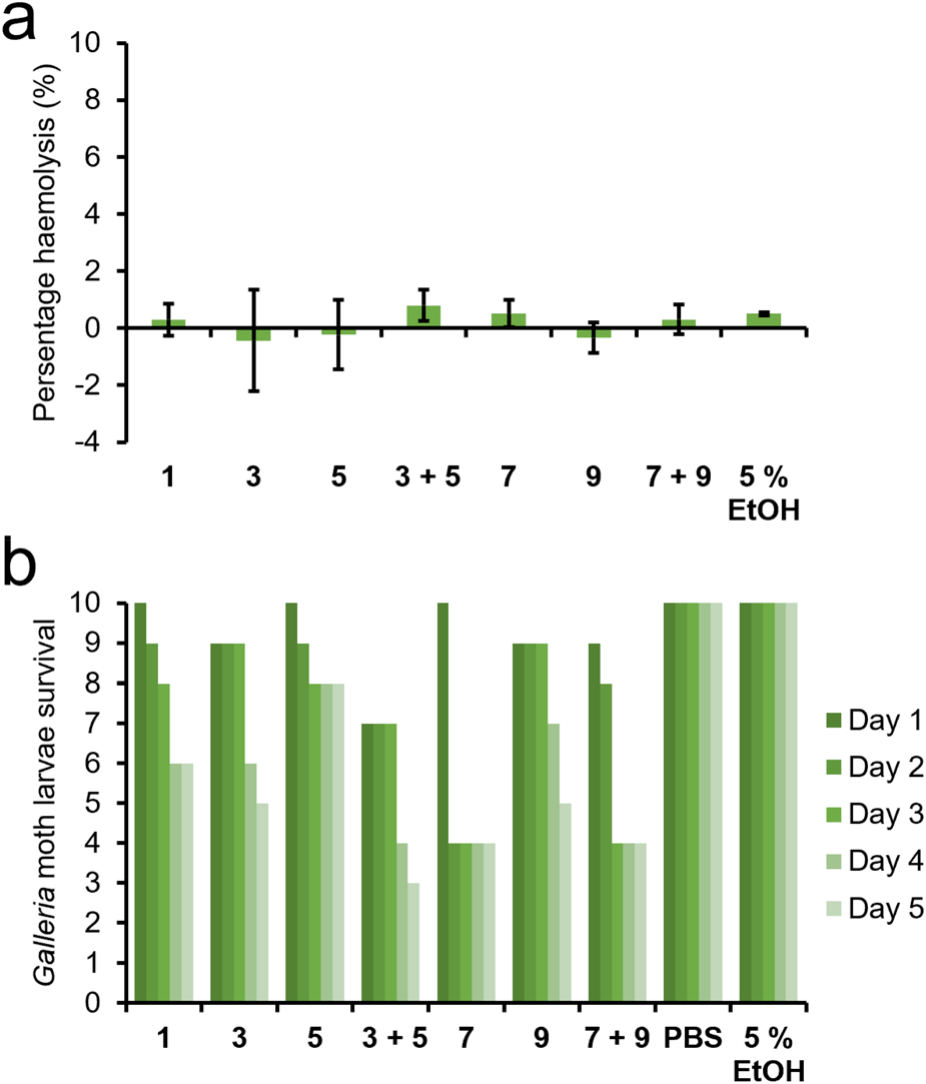
(a) The percentage haemolysis of human erythrocytes determined for 1, 3, 5, 7, 9 and 1 : 1 enantiomeric mixtures of 3 + 5 and 7 + 9 at 1.39 mM. (b) The toxicity of 1, 3, 5, 7, 9, and 1 : 1 enantiomeric mixtures of 3 + 5 and 7 + 9 determined through the observation of *G. mellonella* larvae survival rates after injection with SSA or 1 : 1 SSA enantiomeric mixture (10 µL of 5 mM distilled H_2_O/EtOH 19 : 1 solution). PBS and EtOH : H_2_O (1 : 19) were used as controls.

As our hypothesised mechanism of action includes the selective coordination and permeation of SSAs through the polar membranes of both MRSA and ovarian cancer cells, we also explored the toxicity of SSAs 1, 3, 5, 7, and 9 as either homogenous solutions or heterogenous (1 : 1) enantiomeric mixtures against human erythrocytes. In this experiment (Fig. 9a), the proportion of erythrocytes to undergo lysis in the presence of SSAs supplied at a total molecular concentration of 1.39 mM, chosen to correlate against the upper limits of SSAs supplied in both the antimicrobial and/or anticancer activity studies, Fig. 3 and 4 respectively, were recorded as the percentage haemolysis value. Here the maximum haemolysis recorded for all SSAs/SSA mixtures under these experimental conditions was <2%.

Finally, we gauged the toxicity of SSAs 1, 3, 5, 7, and 9 as either homogenous solutions or heterogenous (1 : 1) enantiomeric mixtures towards multicellular organisms which have an intrinsic immune system.^66^ Here, *Galleria mellonella* larvae were injected with 10 µL of SSA (5 mM) in a distilled H_2_O/EtOH 19 : 1 solution and then incubated at 37 °C for a maximum of five days and the number of surviving larvae recorded each day. As shown in Fig. 9b, 5 exhibited the lowest toxicity against *G. mellonella*, with 8 of 10 larvae surviving for the full five days. When comparing the toxicity of the different enantiomers, those SSAs which incorporate unnaturally occurring d-amino acids (5 and 9) are less toxic than those which incorporate the natural l-amino acids (3 and 7). When comparing the effects of R-group substitution, the leucine appended analogue was shown to exhibit a lower toxicity than the comparative phenylalanine substituted SSA. Interestingly, combining enantiomers in a 1 : 1 ratio, while keeping the total molecular concentration comparative with the single agent studies, showed the enantiomeric SSA mixtures to exhibit either a comparable or enhanced toxicity relative to those studies containing a single SSA. In summary, although SSAs are delivered as self-associated spherical aggregates, interestingly they exhibit toxicity profiles similar to small molecules.

## Conclusions

We have previously shown members from the wider SSA library to exhibit activity against Gram-positive, clinically relevant MRSA USA300 and the A2780 cisplatin susceptible ovarian cancer cell line. During these studies, data obtained from live cell fluorescence microscopy experiments showed intrinsically fluorescent SSAs arriving at the cells surface as spherical aggregates, then forming a coating over the surface of the cell, diffusing across the cell surface membranes, before becoming internalised within the cell and moving to interact with those structures within the cell itself.^34^ We had also shown SSAs to selectively interact with phospholipid membranes of differing composition, and demonstrated the potential for members of the SSA library to be developed as drugs for intravenous administration.^37,39^

Within the scope of this study, we have built on initial structure activity relationships, incorporating chirality through the presence of both natural and unnatural amino acid residues into the SSA structure. Through comparison with the control achiral, glycine substituted 1, we have provided evidence that the incorporation of hydrophobic R-groups, alongside the introduction of chirality within the SSA scaffold and control of self-associative complexation processes significantly enhances both desirable biological activity against both infectious and non-infectious disease-causing cells.

From the data presented here, we hypothesise that SSAs arrive at the surface of the membrane and increase in local concentration over time, disrupting the phospholipid bilayer enabling the flow of ions (including SSAs) from one side of the membrane to the other. As the local concentration of SSA changes, the degree of phospholipid membrane disruption increases/decreases resulting in the corresponding increase/decrease in current, supported by evidence supplied through planar bilayer patch clamp experiments. These planar bilayer patch clamp experiments also identify the presence of membrane pore or ion channel like behaviour – again supporting the hypothesis that these SSAs can form self-associated transmembrane structures, capable of facilitating function (ion flow) in a controlled manner – opening and shutting, depending on SSA concentration.

Physicochemical analysis of these SSAs performed in the solid and solution state confirm a link between strength of intermolecular hydrogen bond formation, properties of amino acid substitution and higher order self-associated aggregation events. We confirm that SSAs may be developed with activity against both drug susceptible and resistant bacterial infection and cancer; with 9 (or a 1 : 1 enantiomeric mixture of 7 + 9) showing the greatest activity against multiple strains of clinically relevant drug resistant and susceptible *S. aureus* and ovarian cancer cell lines. Finally, we show 9 to exhibit a profile competitive to that of known drugs *in vitro* screening experiments, with the results demonstrating tractable ADME properties and defined scope for optimisation. Haemolysis experiments, and GI_50_ determination against RPE-1 cell lines suggest selectivity for our target microbial and cancer cells, while studies against *G. mellonella* confirm 9 to exhibit lower toxicity when compared to 7 and the 1 : 1 antiromantic mixture of 7 + 9.

## Author contributions

PIAP, TA, ERC, LJW: Investigation; validation; writing – review & editing. RJL, JT, CM, KY, IL, AK, ERM, KLFH, OBK, JR, JB, CB, HBW, BM, CO: investigation; validation. JMS, CKH, MDG, CJEH: supervision; validation; writing – review & editing; funding acquisition. JRH: conceptualization; funding acquisition; project administration; supervision; writing – original draft, review & editing.

## Conflicts of interest

There are no conflicts to declare.

## Supplementary Material

SC-017-D5SC03376D-s001

SC-017-D5SC03376D-s002

## Data Availability

CCDC 2205765–2205768 and 2218842 contain the supplementary crystallographic data for this paper.^70*a*–*e*^ The data supporting this article have been included as part of the supplementary information (SI). The code and data analysis scripts used to process the patch clamp data are described within the SI are available for download from Github (https://github.com/). Supplementary information: this includes experimental details and computational modelling, DLS, zeta potential, tensiometry, mass spectrometry, NMR spectroscopy, circular dichroism, crystallography, molecular characterisation, patch clamp, vesicle ion transport and biological data. See DOI: https://doi.org/10.1039/d5sc03376d.
