# Supplementary material for: Amino acid appended supramolecular self-associating amphiphiles demonstrate dual activity against both MRSA and ovarian cancer
Source: Chem Sci. 2026 Mar 10;17(17):8567–79. doi: 10.1039/d5sc03376d (PMC12973313; doi:10.1039/d5sc03376d)
Supplement: SC-017-D5SC03376D-s001 [file SC-017-D5SC03376D-s001.pdf]

Supplementary Information (SI) for Chemical Science.
This journal is © The Royal Society of Chemistry 2026

Amino acid appended supramolecular self-associating amphiphiles demonstrate dual activity against both MRSA and ovarian cancer

Precious I. A. Popoola, Thomas L. Allam, Rebecca J. Lilley, Chandni Manwani, Olivia B. Keers, Junyang Tan, Kylie Yang, Yifan Long, Ewan R. Clark, Lisa J. White, Kira L. F. Hilton, Jennifer Rankin, Jennifer Baker, Charlotte Bennett, Hollie B. Wilson, Evelyn R. Morton, Alvaro Keskküla, Bethany Martin, Christoper O'Connor, J. Mark Sutton, Charlotte K. Hind,* Michelle D. Garrett,* Cally J. E. Haynes* and Jennifer R. Hiscock*

Electronic Supplementary Information (ESI)

Contents

Section S1: General remarks 4

Section S2: Physicochemical methods 4

Section S3: Biological methods 6

Ion transport studies 6

MIC assay 6

Scanning Electron Microscopy (SEM) studies 7

Haemolysis assay 7

Toxicity assay 7

GI_{50} determination assays 7

Patch clamp studies 8

In vitro Absorption, Distribution and Metabolism studies 10

The summary of *in vitro* results is shown in Tables S1-S4. 12

Section S4: Chemical structures 13

Section S5: Chemical synthesis 13

Section S6: Characterisation NMR spectra 17

Section S7: Quantitative ^{1}H NMR studies 25

Summary 31

Section S8: ^{1}H NMR DOSY studies 32

Summary 38

Section S9: ^{1}H NMR self-association data 39

Summary 42

Section S10: Dynamic Light Scattering (DLS) studies 43

DLS studies in H_2O/5 % EtOH 43

Summary 61

Section S11: Zeta potential studies 62
Zeta potential studies in H_2O/5 % EtOH 62
Summary 71
Section S12: Surface tension measurements and critical aggregate concentration (CAC) determination 72
Section S13: Single crystal X-ray structures 75
Hydrogen bonding summary 80
Section S14: Mass spectrometry spectra 80
Summary 84
Section S15: Circular dichroism studies 85
Section S16: Proton NMR spectroscopy titration studies 86
Summary 94
Section S17: Ion transport studies 95
Cl^-/NO_3^- antiport Hill plots – pH 7.2 95
Control experiments 98
Summary 99
Section S18: MIC determination assay 100
Section S19: GI_{50} determination assays 101
RPE-1 cell line 101
A2780 cell line 105
A2780 CisR cell line 110
Summary 114
Section S20: Toxicity assay 115
Section S21: Haemolysis assay data 115
Section S22: Patch clamp studies 116
Changing concentration of 1 117
Changing concentration of 3 137
Changing the concentration of 5 154
Changing holding voltage with 5 190
Changing the concentration of 3 and 5 (1:1) 204
Changing the concentration of 7 210
Changing the concentration of 9 228
Changing the concentration of 7 and 9 (1:1) 250
Control experiments 256

Magnified portions of patch clamp data sets 261
Data analysed magnified portions of patch clamp data sets 290
Section S23: Molecular modelling and simulation 313
Section S24: Low level *in silico* modelling 313
Summary 315
Calculation of LogP values using swissADME 315
Section S25: DFT SSA-Model Lipid Interaction Calculations 316
Section S26: *In vitro* Drug Metabolism and Pharmacokinetics (DMPK) studies 342
Protein Binding Measurements in Human Plasma by Using Equilibrium Dialysis 342
Kinetic Solubility Determination in PBS pH 7.4 342
Metabolic Stability in Rat and Mouse Liver Microsomes 342
Bidirectional Permeability in Caco-2 Cell Line 343
Section S27: SEM Images 344
Section S28: References 346

Section S1: General remarks

A positive pressure of nitrogen and oven dried glassware were used for all reactions. All solvents and starting materials were purchased from known chemical suppliers or available stores and used without any further purification unless specifically stipulated. The NMR spectra were obtained using a Bruker AV2 400 MHz or AVNEO 400 MHz spectrometer. The data was processed using TopSpin 4.1.4. software. NMR chemical shift values are reported in parts per million (ppm) and calibrated to the centre of the residual solvent peak set (s = singlet, br = broad, d = doublet, t = triplet, q = quartet, m = multiplet). Tensiometry measurements were undertaken using the Biolin Scientific Theta Attension optical tensiometer. The data was processed using Biolin OneAttension software. A Hamilton (309) syringe was used for these measurements. The melting point for each compound was measured using Stuart SMP10 melting point apparatus. High resolution mass spectrometry was performed using a Bruker microTOF-Q mass spectrometer and spectra recorded and processed using Bruker's Compass Data Analysis software. Infrared spectra were obtained using Shimadzu IR-Affinity-1 model Infrared spectrometer. The data was analysed in wavenumbers (cm^{-1}) using IRsolution software. DLS and Zeta Potential studies were carried out using an Anton Paar Litesizer™ 500 and processed using Kalliope™ professional software.

Section S2: Physicochemical methods

^{1}H NMR Diffusion Ordered Spectroscopy (DOSY): The hydrodynamic diameter was derived from diffusion rates obtained from ^{1}H NMR DOSY measurements using the Stokes-Einstein equation. The viscosity value used for the calculation was 0.00199 mPa (DMSO).

Quantitative ^{1}H NMR (qNMR) studies: A ^{1}H NMR spectrum was obtained with a delay (d_1 = 60 s) for compounds (112 mM) in DMSO-d_6/ 1.0 % DCM or 5.56 mM in D_2O/ 5.0 % EtOH. Through comparative integration of the anionic and cationic component signals against the internal standard signals (DCM/EtOH), the proportion of these molecular components to become 'lost' from solution, through the adoption of solid-like characteristics can be calculated.

Self-association constant calculation: Self-association constants were determined using Bindfit v0.5.[1] All the data can be accessed online using the hyperlinks provided.

Tensiometry studies: All the samples were prepared in an H_2O/5 % EtOH solution. All samples underwent an annealing process where they were heated to approximately 313 K before being left to cool to RT, enabling each sample to reach a thermodynamic minimum. All samples were prepared through serial dilution of the most concentrated sample. Three surface tension measurements were obtained for each sample at a given concentration using the pendant drop method. The average values were then used to calculate the CAC.

Mass spectrometry studies: Approximately 1 mg of each compound was dissolved in 1 mL of MeOH. This solution was further diluted 100-fold before undergoing analysis where 10 µL of each sample was injected directly into a flow of 10 mM ammonium acetate in 95 % water (flow rate = 0.02 mL/min).

Dynamic light scattering (DLS) studies: All solvents used were filtered to remove any particulates that may interfere with the results obtained. All samples underwent an annealing process, in which they were heated to 313 K before being left to cool to 298 K to allow each sample to reach a thermodynamic minimum. A series of 10 runs were recorded at 298 K.

Zeta potential studies: All solvents used were filtered to remove any particulates that may interfere with the results obtained. All samples underwent an annealing process in which they were heated to 313 K before being left to cool to 298 K to allow each sample to reach a thermodynamic minimum. The final zeta potential value given is an average of the number of experiments conducted at 298 K.

Crystal x-ray studies: Single crystals were produced through selective precipitation using chloroform and hexane. A suitable crystal was selected and mounted on a Rigaku Oxford Diffraction Supernova diffractometer. Data were collected using Cu Kα radiation at 100 K. Structures were solved with the ShelXT or ShelXS structure solution programs via Direct Methods and refined with ShelXL by least Squares minimisation. Olex2 was used as an interface to all ShelX programs (CCDC 2122929).

Circular dichroism studies: All circular dichroism experiments were performed on the JASCO J-175 spectropolarimeter using a 1 mm pathlength quartz cuvette. Far UV-spectra were obtained between 200-260 nm with an average of 4 scans at 100 nm/min, 0.5 nm step resolution, 1.0 second response and 0.5 nm bandwidth. Samples were prepared between 1 mM in 400 µL of H_2O/5 % EtOH.

Proton NMR spectroscopy titration studies: First 1.5 mL of a 0.01 M solution of receptor (SSA) was prepared. Of this solution, 0.5 mL was added to an NMR tube, which was then sealed with an airtight suba seal. The remaining 1.0 mL of the receptor solution was used to make a 0.15 M solution of the TBA salt of the anion. The anion/receptor solution was titrated into the NMR tube in small aliquots and a ^{1}H NMR spectrum taken after each addition. This allows the concentration of the anion in the NMR tube to increase whilst the concentration of receptor to remain constant. Chemical shifts for each ^{1}H NMR spectrum were recorded in ppm and calibrated to the solvent peak set. Bindfit v0.5 is then used to interpret the data to solve the binding constant(s).[1] Where SSAs are supplied as coformulations, they are present in a 1:1 molecular ratio, with the total molecular concentration equal to that of the of the experiment containing a single agent only.

Molecular modelling and simulation: Structures of the compounds were drawn in ChemDraw (version 22.0.0) and were imported into Chem3D (version 22.2.0). MM2 energy minimisations were run, giving the dipole-dipole energy and total energy in kcal/mol. The length of the compound was obtained by measuring the distance between the two atoms furthest away from each other, given in Å and converted to nm.

Low level in silico modelling: Computational calculations to identify primary hydrogen bond donating and accepting sites were conducted in line with studies reported by Hunter using Spartan 20 v1.1.4.[2] Calculations were performed using semi-empirical PM6 methods, after

energy minimisation calculations, to identify E_{max} and E_{min} values. PM6 was used over AM1 in line with research conducted by Stewart.[3]

Section S3: Biological methods

Ion transport studies

Preparation of vesicles: A stock solution of 1-palmitoyl-2-oleoyl-sn-glycero-3-phosphocholine (POPC) was prepared by dissolving 1 g of POPC in 35 mL de-acidified $CHCl_3$. The $CHCl_3$ was passed through a column of basic alumina before use. This solution was stored at -20 °C when not in use.

A lipid film of POPC was prepared by adding 1–4 mL of the above stock solution into a pre-weighed falcon tube and gently removing the $CHCl_3$ under a stream of nitrogen. The lipid film was dried under vacuum overnight. The lipid film was re-hydrated by vortexing with the desired internal buffer solution, then subjected to 9 freeze-thaw cycles. Typically, the lipids were not thawed on the last cycle and were stored at -20 °C for use at a later date.

The lipid was thawed to room temperature and allowed to stand for 30 mins, then extruded 27 times through a 200 nm polycarbonate membrane. The resulting unilamellar vesicles were dialysed against the desired external buffer solution for a minimum of 2 hours.

Transport experiments protocol: The vesicles prepared as described above were diluted to a known volume of 5 mL using the desired external buffer solution. This solution was further diluted into individual 5 mL samples for testing at a final lipid concentration of 1 mM. The transporter was added to the vesicles from a stock solution in DMSO (5 mM) after 30 s in order to initiate the transport and any resulting chloride efflux was monitored using a chloride selective electrode (ISE – Cole Parmer). At the end of the experiment, the vesicles were lysed by the addition of 40 μL Triton-X 100 (10 wt% in H_2O). The final electrode reading was taken 2 mins later and used to calibrate 100% chloride efflux. Each experiment was performed 3 times.

MIC assay

Preparation of bacterial plates: Sterile TSB agar plates were streaked using the desired bacteria [*Staphylococcus aureus* (9144, 13616, USA 300 and 1199B), *Enterococcus faecalis* (NCTC 775 and 12201) and *Enterococcus faecium* (NCTC 12204)] and incubated at 37 °C overnight.

Preparation of inoculum: An initial culture was made up by inoculating TSB (5 mL) with 3 single colonies of the desired bacteria under sterile conditions and incubated at 37 °C overnight. The optical density at 600 nm (OD_{600}) was adjusted using sterile distilled H_2O (dH_2O) to equal ~1 x 10^6 bacteria/mL.

Preparation of 96-well microplate: A solution of each compound in H_2O/5 % EtOH was made at a top concentration of 5.56 mM on the day of experiment. The compound solution (200 μL) was added to and diluted 50:50 down the plate. The bacterial suspension (100 μL) was dispensed into each well under sterile conditions. The plates were sealed using parafilm and incubated at 37 °C for 20 hours, after which the OD_{600} was measured.

Scanning Electron Microscopy (SEM) studies

Sample preparation: Bacterial samples at 1x10^8 CFU/mL were treated for 10 minutes at 37 °C with 2 x MIC of SSA. Samples were then fixed using 2 % formaldehyde (48 % stock).

A drop of broth was left to settle overnight on to poly-L-lysine coated glass coverslips. The coverslips were then secondarily fixed in osmium tetroxide and then dehydrated through a graded ethanol series. Ethanol was replaced with two changes of hexamethyldisilazane (HMDS). Once dry, the coverslips were mounted onto aluminium stubs using sticky carbon disks. Stubs were gold coated with an Atom Tech ion beam coater.

Image collection a processing: SEM images were recorded using a Zeiss Sigma 300 VP, at 3 kV accelerating voltage, with a 6 mm working distance.

Haemolysis assay

Preparation of 96-well plate: Protocol modified from Travis et al.[4] Heparinised human red blood cells collected from a volunteer and washed three times in PBS (pH 7.4). Compounds were serial diluted using PBS buffer across a 96-well V bottom Greiner plate. Negative control (100 µL PBS), and positive control (100 µL 0.1 % (v/v) Triton-X-100) were added to the plate. 100 µL of 10 % (v/v) of blood suspension added to all wells. Plates were incubated for 60 minutes at 37 °C. After incubation, plates were centrifuged for 15 minutes at 4680 rpm. The resultant supernatant was then transferred to a 96-well flat bottom plate and absorbance read at 540 nm (Fluostar Omega). Percent haemolysis was calculated using the formula as shown in Equation S2.

Equation S2 - Percentage haemolysis calculation.

$$\text{Haemolysis (\%)} = \frac{A_{sample} - A_{negative}}{A_{Triton} - A_{negative}}\ X\ 100$$

Toxicity assay

***G. mellonella* treatment assay**: *G. mellonella* larvae were purchased from Livefood UK Ltd. (Rooks Bridge, UK) and maintained on wood chips in the dark at 15 °C until use. *Galleria* larvae were injected with 10 µL of compound in H_2O/5 % EtOH, incubated at 37 °C for 5 days and the deaths counted. Groups of 10 *Galleria* were injected per compound.

GI_{50} determination assays

Cell culture: The RPE-1 retinal pigment epithelial, A2780 human ovarian carcinoma, and A2780 CisR cisplatin resistant human ovarian carcinoma cell lines were donated with thanks from the Institute of Cancer Research. The A2780 and A2780 CisR cell lines were cultured in IMDM supplemented with 10 % FBS at 37 °C and 5 % CO_2, while the RPE-1 cell line was cultured in a 1:1 mixture of Dulbecco's Modified Eagle Medium and Ham's F-12 Nutrient Mixture (DMEM/F-12) at 37 °C and 5 % CO_2.

SRB assay: Cells were seeded into 96-well plates at 800 cells per well/cpw (A2780), 1600 cpw (A2780 CisR) or 400 cpw (RPE-1) in cell culture medium and cultured for 48 hours followed by addition of each compound over an eight-point concentration range (each concentration in triplicate). Plates were then cultured for a further 96 hours, after which the cell culture

medium was removed from each well and the cells fixed with addition of 70 μL/well of 10 % (w/v) trichloroacetic acid (TCA) in distilled water followed by incubation for 30 minutes at RT. Each plate was then washed with distilled water five times before addition of 70 μL SRB dye (0.4 % (w/v) SRB dye (ThermoFisher Scientific, USA) solubilised in 1 % (v/v) acetic acid/ dH_2O) and incubated for 30 minutes at RT, followed by washing three times with 1 % (v/v) acetic acid and drying in a 37 °C oven overnight. Once dry, 100 μl of 10 mM Tris-base (ThermoFisher Scientific, USA) was added to each well and plates put on a microplate shaker for 10 minutes at 200 rpm to solubilize the dye. Absorbance values were then read at wavelength 490 nm on a Victor X4 multi-label plate reader (PerkinElmer Life Sciences, USA), data analysed using Microsoft Excel and graphs produced using GraphPad Prism 9.0 and the GI_{50} value calculated.

Patch clamp studies

Solutions and compound preparation: All solvents and starting materials were purchased from known chemical suppliers. Compound stock solutions were prepared in DMSO. Buffer A consisted of KCl (489 mM) and NaOAc (5 mM) with a pH of 5.5 and an ionic strength of 500 mM. Buffer B consisted of Na_2SO_4 (167 mM) and NaOAc (5 mM) with a pH of 5.5 and an ionic strength of 500 mM.

Vesicle formation: Giant unilamellar Vesicles (GUVS) were prepared through the electro formation method using a Nanion VesiclePrepPro. A solution of 1,2-diphytanoyl-sn-glycero-3-phosphocholine (DPhPC) (10 mM) with 10 % cholesterol in chloroform (20 μL) was deposited onto an ITO coated glass surface (surface coated glass slide) and allowed to evaporate to create a dehydrated thin lipid film. A greased O-ring was then placed around the dehydrated lipid film to create a seal. This volume was then filled with a sucrose solution (275 μL, 200 mM) and then sealed with a second ITO coated glass slide. An AC current (3 V/5 Hz) was then applied. After 2 hrs, the voltage and frequency were reduced in steps over 30 mins to 1.6 V/1 Hz and held for 1 hr before being reduced to 0 V/0 Hz over a further 30 min period.

Patch clamp measurements: Experiments were performed using a Port-a-Patch system (Nanion Technologies) with borosilicate glass chips that contain an aperture diameter of approximately 1 μm. Buffer A (5 μL) was placed on the underside of the chip over the aperture, The chip was then placed into the port-a-patch system. Buffer A (15 μL) was then added to the top of the chip and 5 μL of the GUV stock solution added. The GUVS were the positioned over the aperture in the chip by application of negative pressure (-30 mbar). When a GUV is positioned over the aperture in the chip, it bursts, leaving a planar bilayer as a seal. The negative pressure is then removed, leaving the planar bilayer in place over the aperture. The external buffer A was the exchanged with buffer B, through four simultaneous buffer exchanges (4 x 15 μL). The holding potential was set to ±50 or ±100 mV depending on the experiment. The compound in a DMSO: buffer B 1:50 mixture (5 μL) was then added at t = 30 s, to give a final experimental concentration of 0.1 – 1 mM as appropriate. The number of sweeps was set to 1000, the sweep interval to 0.00 s, sample interval to 1.00 ms and the protocol was run for a total of 5 mins. Each experiment was repeated a minimum of three time to ensure reproducibility.

Data analysis: Initially the .asc files generated from the patch clamp experiment were curated into the format – internal_molecule_id/ experimental_conditions/ experiment_id/ files_ending_experiment_id.asc.

Files were read and converted from .asc files to csv files using Python scripts. The process combined all .asc files associated with an experiment into a single csv which can be read in and manipulated using Python. The experiments had been recorded at different resolutions; therefore, time rather than number of points has been used in the analysis. Two methods were used to categorise the time series patch clamp data into discrete states for analysis, method 1(to get an understanding of the true baseline and small current changes) and method 2 (to get a macroscopic view of each experiment).

Method 1 involved finding the mean of the first 200 points for each experiment. The system was defined as being in an event state if a percentage of datapoints are above the baseline threshold (mean + standard deviation for each experiment). To do this, a convolution operation was applied to a masked version of the patch clamp current data with a vector containing only ones set to length 20. The masked version of the dataset set each datapoint 1 if the datapoint was above the baseline threshold and 0 if it was below. The convolution returned a look forward window with the number of points above and below the baseline which was converted to a percentage and checked against a threshold (set at 70 %). This set to 1 (event) if above and 0 (baseline) if below. Method 1 was used as a tool in analysis to pick and plot all events. These events were then post-processed and those events with a mean current below the standard deviation of the baseline or those that are too short-lived (less than 0.2 seconds by default) were removed.

Method 2 was a data binning approach used to classify the data into 4 event types (though these have been scaled for the variable current experiments) – 0: baseline (-inf A to 0.5e-10 A), 1: events (0.5e-10 A to 5e-10 A), 3: events where the current readings surpass the detector's measurable range (4.9e-8 A to inf A), and 2: events where current is between 5e-10 A and 4.9e-8 A. These current bins are as stated above for + 100 mV, halved for + 50 mV experiments, and made negative for -100 mV and for -50 mV. When the current is around the cutoffs, the noise causes the data to be categorised as above and below and threshold, these times are set to the minimum resolution of the experiment.

The data was processed using a PatchClampData class coded in Python which gives the ability to run these thresholding methods and generate the plots used within this paper and ESI. It has also allowed a summary table of the experiments including the total time of each experiment, the time in each event state and the bins used to threshold each experiment to be generated. The code for this project is available on GitHub - https://github.com/ta1u18/ssa_patch_clamp_data_analysis and original data, processed data and figures available as part of the supplementary information.

In vitro Absorption, Distribution and Metabolism studies

General note: These studies were performed by Contract Research Organisation (CRO) Pharmaron. The methodology provided within the ESI is published within the express permission of this organisation. The percentage of a compound recovered during the analysis process should be 100 %; any deviation from this value indicates experimental limitations such as unintended potential non-specific binding events to experimental equipment, solubility issues, etc.

Protein Binding measurements in human plasma by using equilibrium dialysis:

The fraction unbound in human plasma (P-KB, Ltd People's hospital of Shandong) was measured using 96-well Equilibrium Dialysis Plate (HTDialysis LLC, Gales Ferry, CT). Human plasma containing 5 µM test compound was dialysed against dialysis buffer (PBS, pH 7.4) with initial samples taken to allow for assessment of plasma stability and recovery before the plate was incubated for 6 hours at 37°C at 5% CO_2. Samples were removed at 6 hours, and all samples were matrix matched and quenched with acetonitrile containing internal standard. Samples were centrifuged at 3,220g for 30 minutes to precipitate protein and enable sampling of supernatant prior to appropriate dilution for liquid chromatography mass-spectrometry (LC-MS/MS) analysis.

Samples were analysed using a Shimadzu Nexera LC Binary Gradient LC-30AD (Shimadzu Inc, Kyoto), coupled to a Sciex 6500+ (AB Inc., Illinois). Separation was achieved using a XSelect HSS T3 (2.5µm, 2.1 x 50 mm) (Waters Ltd., Wilmslow) column at a temperature of 40°C and a binary mobile phase gradient at a flow rate of 0.8 mL/min. Initial LC conditions comprised of 95% solvent A (0.1% formic acid in water), 5% solvent B (0.1% formic acid in acetonitrile); this was ramped to 98% B at 0.5 min, held for 0.4 min and then immediately returned to initial conditions. Sample analysis was by electrospray ionisation in combined with multiple reaction monitoring in positive ionisation mode. The ionisation voltage was +5500 V.

Kinetic Solubility Determination in PBS pH 7.4:

The stock solutions of test and control compounds were prepared in DMSO at 10 mM. From this a 300 µM solution was prepared in duplicate in 100 mM Phosphate Buffered Saline (PBS, pH 7.4 ± 0.1). These solutions were then shaken at 1100 rpm for 2 hours at 25°C. Following incubation, the samples were filtered using a vacuum manifold. The filtrate was analysed and quantified against a standard of known concentration using liquid chromatography mass-spectrometry (LC-MS/MS).

Samples were analysed using a Shimadzu Nexera LC Binary Gradient LC-30AD (Shimadzu Inc, Kyoto), coupled to a Sciex 4500 mass spectrometer (AB Inc., Illinois). Separation of compounds was achieved using an XSelect HSS T3 column (2.5µm, 50 x 2.1 mm) (Waters Ltd., Wilmslow) at a temperature of 40°C and a binary mobile phase gradient at a flow rate of 0.8 mL/min. Initial LC conditions comprised of 95% solvent A (0.1% formic acid in water) and 5% solvent B (0.1% formic acid in acetonitrile). This was ramped to 98% solvent B at 0.5 minutes, held for 0.4 minutes and then immediately returned to initial conditions. Sample analysis was by electrospray ionisation combined with multiple reaction monitoring in positive ion mode. The ionspray voltage was 5500V.

Metabolic Stability in Rat, Mouse and Human Liver Microsomes:

The metabolic stability of compounds at a concentration of 1 µM, in rat, mouse and human liver microsomes (BD Gentest, Franklin Lakes NJ) was measured following incubation with hepatic microsomes (0.5 mg of protein / mL) in the presence and absence of cofactor (NADPH) at 37 °C. Aliquots of each incubation were removed at 0.5, 5, 15, 30 and 60 minutes. The reaction was stopped by the addition of cold acetonitrile containing analytical internal standard (IS).

Samples were then centrifuged at 3,220 g for 40 minutes. The supernatant was aliquoted and added to ultra-pure H_2O in a 1:1 ratio in preparation for LC-MS/MS analysis. Incubations were conducted in duplicate; control incubations (substituting PBS for NADPH) were conducted simultaneously. All control incubations were within acceptance criteria in mouse, rat, and human microsomes confirming stability was acceptable over 60 minutes in the absence of co-factor.

Samples were analysed using a Shimadzu Shimadzu Nexera LC Binary Gradient LC-30AD (Shimadzu Inc, Kyoto), coupled to a Sciex 5500 mass spectrometer (AB Inc., Illinois) for rat and mouse microsomes and a Sciex 6500+ for human microsomes. Separation achieved using an XSelect HSS T3 column (2.5µm, 50 x 2.1 mm) (Waters Ltd., Wilmslow) at a temperature of 40°C and a binary mobile phase gradient at a flow rate of 0.7 mL/min. Initial LC conditions comprised of 95% solvent A (0.1% formic acid in water) and 5% solvent B (0.1% formic acid in acetonitrile). This was ramped to 95% solvent B at 0.7 minutes, held for 0.5 minutes and then immediately returned to initial conditions. Sample analysis was by electrospray ionisation combined with multiple reaction monitoring in positive ion mode. The ionspray voltage was 5500V.

Bi-directional Permeability in Caco-2 Cell Line:

The bi-directional permeability in Caco-2 cell lines was determined by seeding cells onto an HTS Transwell plate. Cells were cultivated for 14-18 days in a cell culture incubator at 37°C, 5% CO_2, 95% relative humidity. Cell culture medium was replaced every other day. To assess monolayer integrity the transepithelial electrical resistance (TEER) across the monolayer was measured using Millicell Epithelial volt-ohm meter prior to assay and Lucifer yellow (LY) fluorescence measurement post-assay.

Test compound was prepared in DMSO and diluted in Hank's Balanced Saline Solution (HBSS, pH 7.4) to a final concentration of 5 µM. Compound was added to the donor compartment of the transwell plate for both apical to basolateral (A>B) and basolateral to apical (B>A) measurements, with blank HBSS being added to the receiver compartment. The transwell plate was then incubated at 37°C, 5% CO_2 for 2 hours.

Samples were taken at T=0 and T=2 hours determine the concentration of initial donor sample, and the concentration of receiver and donor wells post-incubation, respectively. The samples were quenched with solvent containing internal standard (IS). The supernatant was mixed with an appropriate volume of ultra-pure water before LC-MS/MS analysis.

Samples were analysed using a Shimadzu Nexera LC Binary Gradient LC-30AD (Shimadzu Inc, Kyoto), coupled to a Sciex 6500+ (AB Inc., Illinois). Separation of compounds was achieved using a HALO 90Å biphenyl (2.7µm,2.1x50mm) (Advanced Materials Technology, Wilmington) column at a temperature of 40°C. A binary mobile phase gradient at a flow rate of 0.7 mL/min was used. Initial LC conditions comprised of 95% solvent A (0.1% formic acid in water) and 5% solvent B (0.1% formic acid in acetonitrile). This was ramped to 95% solvent B at 0.7 minutes, held for 0.5 minutes and then immediately returned to initial conditions. Sample analysis was by electrospray ionisation combined with multiple reaction monitoring in positive ion mode. The ion spray voltage was 5500V.

In vitro Absorption, Distribution and Metabolism results

The summary of *in vitro* results is shown in Tables S1-S4.

Table S1 – Result from solubility assays performed in PBS pH 7.4 for SSA 9.

ID	Solubility (µM)
**SSA 9**	271

Table S2 – Summary of results from Caco-2 permeability assays for SSA 9. P_{app} is given as $x10^{-6}$ cm/s. Low P_{app} = < 5 $x10^{-6}$ cm/s; Moderate P_{app} = 5-10 $x10^{-6}$ cm/s; High P_{app} = > 10 $x10^{-6}$ cm/s. A = apical; B = basolateral. Recovery = % SSA recovered from the assay after 2 hours.

ID	P_{app} (A-B)	% Recovery	P_{app} (B-A)	% Recovery	Efflux Ratio
**SSA 9**	0.17	110.8	16.8	97.7	96.9

Table S3 – Summary of results from human plasma protein binding assays for SSA 9. % Bound = % of SSA bound to the plasma proteins after 6 hours incubation at 37 °C. Low % Bound = < 80 %; Moderate % Bound = 80-95 %; High % Bound = 95-99 %; Very high % Bound = > 99 %. % Recovery = % SSA recovered from the assay after 6 hours.

ID	% Bound	% Recovery	% Stability
**SSA 9**	99.7	98.3	98.9

Table S4 – Summary of results from mouse, rat and human microsomal stability assays for SSA 9. The *in vitro* half-life ($t_{1/2}$) and microsomal intrinsic clearance (CL_{int}) were calculated from the elimination rate (k, the slope of the natural log of concentration verses time curve), assuming first order kinetics, using the formulae $t_{1/2} = 0.693/\text{-}k$ and $CL_{int} = 0.693/t_{1/2}$ x microsomal protein concentration. This compound would be classed as a low clearance compound in all three species.

	Mouse		Rat		Human	
ID	*In vitro* $t_{1/2}$ (min)	CL_{int} (µL/min/mg)	*In vitro* $t_{1/2}$ (min)	CL_{int} (µL/min/mg)	*In vitro* $t_{1/2}$ (min)	CL_{int} (µL/min/mg)
**SSA 9**	> 185	< 7.5	> 185	< 7.5	> 185	< 7.5

Section S4: Chemical structures

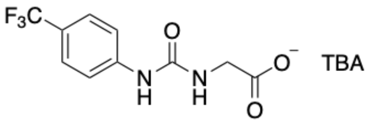

1

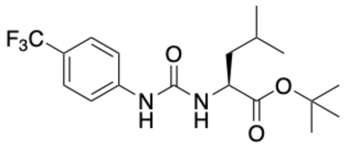

2

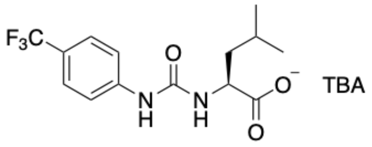

3

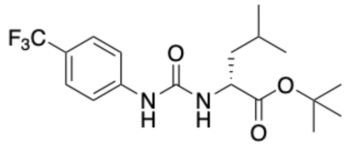

4

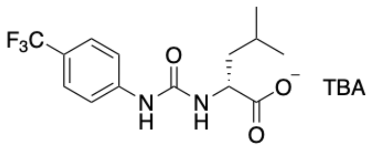

5

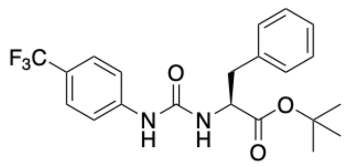

6

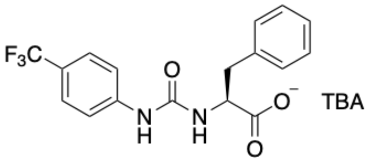

7

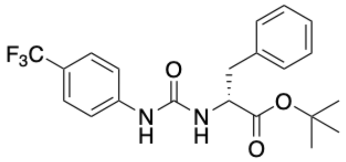

8

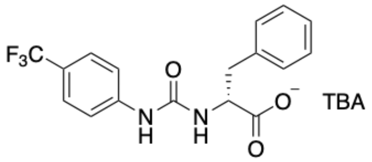

9

Figure S1 - Chemical structures of **1** - **9**. TBA = tetrabutylammonium.

Section S5: Chemical synthesis

Compound 1: This was synthesised in line with previously published methods.[5] ^{1}H NMR (400 MHz, 298 K, DMSO-d_6): δ: 10.27 (s, 1H), 7.68 (d, *J* = 8.60 Hz, 2H), 7.50 (d, *J* = 8.60 Hz, 2H), 6.75 (s, 1H), 3.18 - 3.13 (m, 8H), 1.60 - 1.52 (m, 8H), 1.34 - 1.25 (m, 8H), 0.93 (t, *J* = 14.64 Hz, 12H).

Compound 2: 1-Isocyanato-4-(trifluoromethyl) benzene (0.29 mL, 2.00 mmol) was added to L-Leucine *tert*-butyl ester hydrochloride (0.45 g, 2 mmol) in anhydrous pyridine (10 mL) and stirred at room temperature overnight. The mixture was then taken to complete dryness and redissolved in chloroform (5 mL), followed by dropwise additions of hexane (> 5 mL) resulting in precipitation. The precipitate was collected via filtration and purified via flash column chromatography with a 3:2 mixture of hexane to ethyl acetate. All fractions were monitored using thin layer chromatography and were taken to complete dryness. The pure product was identified using NMR spectroscopy and collected as a white solid with a yield of 53 % (0.40 g, 1.07 mmol); melting point: > 200 °C; ^{1}H NMR (400 MHz, 298 K, DMSO-d_6): δ: 8.96 (s, 1H), 7.57 (s, 4H), 6.55 (d, *J* = 8.12 Hz, 1H), 4.13 (q, *J* = 22.92 Hz, 1H), 1.71 – 1.62 (m, 1 H), 1.54 – 1.48 (m, 2H), 1.41 (s, 9H), 0.91 (q, *J* = 18.80 Hz, 6H); ^{13}C{^{1}H} NMR (100 MHz, 298 K, DMSO-d_6): δ: 172.8 (C=O), 154.9 (C=O), 144.3 (ArC), 126.5 (q, *J* = 3.81 Hz, ArCH), 123.7 (ArC), 121.7 (q, *J* = 31.78 Hz, CF_3), 117.7 (ArCH), 81.1 (C), 51.9 (CH), 41.3 (CH_2), 28.1 (CH), 24.9 (CH_3), 23.1 (CH_3), 22.1 (CH_3); IR (film): ν = 3338 (NH stretch), 2958, 1701, 1680, 1527, 1332, 1315, 1155, 1105, 1066, 833, 607; HRMS for the carboxylate-urea ion ($C_{14}H_{16}F_3N_2O_3^-$); HRMS

for the *tert*-butyl carboxylate-urea ($C_{18}H_{25}F_3N_2O_3$) (ESI^-): m/z: act = 374.1739 $[M]^-$, cal = 374.1817 $[M]^-$.

Compound 3: Zinc bromide (2.25 g, 10 mmol) was added to **2** (0.40 g, 1.07 mmol) in dichloromethane (5 mL) and stirred at room temperature for 24 hours. The reaction mixture was then quenched with water (20 mL) and stirred at room temperature for 4 hours, resulting in precipitation. The precipitate was collected via filtration and TBA hydroxide in methanol (0.51 mL, 1 M) was added. The mixture was taken to dryness, and the pure product was collected via flash column chromatography with 100 % ethyl acetate followed by 100 % methanol. The methanol fraction was taken to dryness to give the pure product as a brown solid with a yield of 62 % (0.25 g, 0.45 mmol); melting point: 131 °C; ^1H NMR (400 MHz, 298 K, DMSO-d_6): δ: 9.85 (s, 1H), 7.65 (d, *J* = 8.48 Hz, 2H), 7.49 (d, *J* = 8.40 Hz, 2H), 6.74 (s, 1H), 3.74 (q, *J* = 18.80 Hz, 1H), 3.19 – 3.14 (m, 8H), 1.74 – 1.67 (m, 1H), 1.61 – 1.48 (m, 9H), 1.40 – 1.24 (m, 9H), 0.96 – 0.84 (m, 18H); $^{13}C\{^1H\}$ NMR (100 MHz, 298 K, DMSO-d_6): δ: 174.9 (C=O), 155.4 (C=O), 146.3 (ArC), 125.9 (d, *J* = 3.84 Hz, ArCH), 124.0 (ArC), 119.9 (q, *J* = 31.44 Hz, CF_3), 117.2 (ArCH), 58.0 (CH_2), 54.2 (CH), 44.6 (CH_2), 25.0 (CH_3), 23.7 (CH_3), 23.5 (CH_2), 23.4 (CH_3), 19.6 (CH_2), 13.9 (CH_3); IR (film): ν = 3311 (NH stretch), 2960, 1697, 1604, 1319, 1105, 1064, 842; HRMS for the carboxylate-urea ion ($C_{14}H_{16}F_3N_2O_3^-$) (ESI^-): m/z: act = 317.1110 $[M]^-$, cal = 317.1118 $[M]^-$.

Compound 4: 1-Isocyanato-4-(trifluoromethyl) benzene (0.29 mL, 2.00 mmol) was added to D-Leucine *tert*-butyl ester hydrochloride (0.45 g, 2 mmol) in anhydrous pyridine (10 mL) and stirred at room temperature overnight. The mixture was then taken to complete dryness and redissolved in chloroform (5 mL), followed by dropwise additions of hexane (> 5 mL) resulting in precipitation. The precipitate was collected via filtration and purified via flash column chromatography with a 3:2 mixture of hexane to ethyl acetate. All fractions were monitored using thin layer chromatography and were taken to complete dryness. The pure product was identified using NMR spectroscopy and collected as a white solid with a yield of 75 % (0.56 g, 1.50 mmol); melting point: > 200 °C; ^1H NMR (400 MHz, 298 K, DMSO-d_6): δ: 8.96 (s, 1H), 7.57 (s, 4H), 6.55 (d, *J* = 8.12 Hz, 1H), 4.13 (q, *J* = 22.76 Hz, 1H), 1.70 – 1.62 (m, 1 H), 1.52 – 1.48 (m, 2H), 1.41 (s, 9H), 0.91 (q, *J* = 18.72 Hz, 6H); $^{13}C\{^1H\}$ NMR (100 MHz, 298 K, DMSO-d_6): δ: 172.8 (C=O), 154.9 (C=O), 144.3 (ArC), 126.5 (q, *J* = 3.82 Hz, ArCH), 123.7 (ArC), 121.7 (q, *J* = 31.78 Hz, CF_3), 117.7 (ArCH), 81.1 (C), 51.9 (CH), 41.3 (CH_2), 28.1 (CH_3), 24.9 (CH_3), 23.1 (CH_3), 22.1 (CH_3); IR (film): ν = 3338 (NH stretch) 2958, 1703, 1680, 1529, 1332, 1315, 1155, 1107, 1066, 833, 609; HRMS for the *tert*-butyl carboxylate-urea ($C_{18}H_{25}F_3N_2O_3$) (ESI^-): m/z: act = 374.1763 $[M]^-$, cal = 374.1817 $[M]^-$.

Compound 5: Zinc bromide (2.25 g, 10 mmol) was added to **4** (0.56 g, 1.50 mmol) in dichloromethane (5 mL) and stirred at room temperature for 24 hours. The reaction mixture was then quenched with water (20 mL) and t stirred at room temperature for 4 hours, resulting in precipitation. The precipitate was collected via filtration and TBA hydroxide in methanol (0.51 mL, 1M) was added. The mixture was taken to dryness, and the pure product was collected via flash column chromatography with 100 % ethyl acetate followed by 100 % methanol. The methanol fraction was taken to dryness to give the pure product as a brown solid with a yield of 68 % (0.38 g, 0.68 mmol); melting point: 120 °C; ^1H NMR (400

MHz, 298 K, DMSO-*d*$_6$): δ: 10.01 (s, 1H), 7.67 (d, *J* = 8.28 Hz, 2H), 7.49 (d, *J* = 8.60 Hz, 2H), 6.81 (s, 1H), 3.75 (q, *J* = 18.64 Hz, 1H), 3.19 – 3.14 (m, 8H), 1.75 – 1.66 (m, 1H), 1.61 – 1.48 (m, 9H), 1.41 – 1.24 (m, 9H), 0.96 – 0.85 (m, 18H); $^{13}C\{^1H\}$ NMR (100 MHz, 298 K, DMSO-*d*$_6$): δ: 175.0 (C=O), 155.4 (C=O), 146.3 (ArC), 125.9 (d, *J* = 3.9 Hz, ArCH), 121.7 (q, *J* = 31.48 Hz, CF_3), 117.2 (ArCH), 58.0 (CH_2), 54.2 (CH), 44.5 (CH_2), 25.0 (CH_3), 23.7 (CH_3), 23.5 (CH_2), 23.4 (CH_3), 19.6 (CH_2), 13.9 (CH_3); IR (film): ν = 3309 (NH stretch), 2960, 1697, 1606, 1319, 1107, 1066, 846; HRMS for the carboxylate-urea ion ($C_{14}H_{16}F_3N_2O_3^-$) (ESI$^-$): m/z: act = 317.1160 $[M]^-$, cal = 317.1118 $[M]^-$.

Compound 6: 1-Isocyanato-4-(trifluoromethyl) benzene (0.29 mL, 2.00 mmol) was added to a stirring solution of L-Phenylalanine *tert*-butyl ester hydrochloride (0.52 g, 2 mmol) in anhydrous pyridine (10 mL) and left at room temperature overnight. The mixture was then taken to complete dryness and redissolved in chloroform (5 mL), followed by dropwise additions of hexane (> 5 mL) resulting in precipitation. The precipitate was collected via filtration and purified via flash column chromatography with a 3:2 mixture of hexane to ethyl acetate. All fractions were monitored using thin layer chromatography and were taken to complete dryness. The pure product was identified using NMR spectroscopy and collected as a white solid with a yield of 61 % (0.50 g, 1.22 mmol); melting point: > 200 °C; ^1H NMR (400 MHz, 298 K, DMSO-*d*$_6$): δ: 9.10 (s, 1H), 7.56 (s, 4H), 7.33 - 7.20 (m, 5H), 6.52 (d, *J* = 7.52 Hz, 1H), 4.41 (q, *J* = 20.64 Hz, 1H), 2.99 (d, *J* = 5.04 Hz, 2H), 1.35 (s, 9H); $^{13}C\{^1H\}$ NMR (100 MHz, 298 K, DMSO-*d*$_6$): δ: 171.5 (C=O), 154.7 (C=O), 144.2 (ArC), 137.2 (ArC), 129.8 (ArCH), 128.7 (ArCH), 127.0 (ArCH), 126.4 (q, *J* = 3.77 Hz, ArCH), 121.5 (q, *J* = 31.75 Hz, CF_3), 123.7 (ArC), 117.7 (ArCH), 81.4 (C), 54.6 (CH), 37.9 (CH_2), 28.0 (CH_3); IR (film): ν = 3381 (NH stretch), 2987, 1681, 1535, 1327, 1315, 1234, 1159, 1107, 1066, 835, 700, 609; HRMS for the *tert*-butyl carboxylate-urea ($C_{21}H_{23}F_3N_2O_3$) (ESI$^-$): m/z: act = 408.1227 $[M]^-$, cal = 408.1661 $[M]^-$.

Compound 7: Zinc bromide (2.25 g, 10 mmol) was added to a stirring solution of **6** (0.50 g, 1.22 mmol) in dichloromethane (5 mL) and stirred at room temperature for 24 hours. The reaction mixture was then quenched with water (20 mL) and stirred at room temperature for 4 hours, resulting in precipitation. The precipitate was collected via filtration and TBA hydroxide in methanol (0.51 mL, 1M) was added. The mixture was taken to dryness, and the pure product was collected via flash column chromatography with 100 % ethyl acetate followed by 100 % methanol. The methanol fraction was taken to dryness to give the pure product as a brown solid with a yield of 54 % (0.27 g, 0.45 mmol); melting point: 160 °C; ^1H NMR (400 MHz, 298 K, DMSO-*d*$_6$): δ: 9.81 (s, 1H), 7.64 (d, *J* = 8.64 Hz, 2H), 7.49 (d, *J* = 8.64 Hz, 2H), 7.15 - 7.04 (m, 5H), 6.61 (d, *J* = 5.12 Hz, 1H), 3.96 (q, *J* = 15.92 Hz, 1H), 3.17 - 2.97 (m, 10 H), 1.60 – 1.51 (m, 8H), 1.34 – 1.25 (m, 8H), 0.92 (t, *J* = 14.64 Hz, 12H); $^{13}C\{^1H\}$ NMR (100 MHz, 298 K, DMSO-*d*$_6$): δ: 173.2 (C=O), 155.2 (C=O), 146.0 (ArC), 140.3 (ArC), 130.1 (ArCH), 128.0 (ArCH), 126.0 (q, *J* = 3.75 Hz, ArCH), 125.8 (ArCH), 124.0 (ArC), 120.2 (q, *J* = 31.55 Hz, CF_3), 117.2 (ArCH), 57.9 (t, *J* = 2.5 Hz, CH_2), 56.7 (CH), 39.1 (CH_2), 23.5 (CH_2), 19.7 (CH_2), 13.9 (CH_3);IR (film): ν = 3419 (NH stretch), 2964, 1695, 1591, 1485, 1394, 1319, 1109, 1064, 846, 705; HRMS for the carboxylate-urea ion ($C_{17}H_{14}F_3N_2O_3^-$) (ESI$^-$): m/z: act = 351.0952 $[M]^-$, cal = 351.0962 $[M]^-$.

Compound 8: 1-Isocyanato-4-(trifluoromethyl) benzene (0.29 mL, 2.00 mmol) was added to D-phenylalanine *tert*-butyl ester hydrochloride (0.52 g, 2 mmol) in anhydrous pyridine (10 mL) and stirred at room temperature overnight. The mixture was then taken to complete dryness and redissolved in chloroform (5 mL), followed by dropwise additions of hexane (> 5 mL) resulting in precipitation. The precipitate was collected via filtration and purified via flash column chromatography with a 3:2 mixture of hexane to ethyl acetate. All fractions were monitored using thin layer chromatography and were taken to complete dryness. The pure product was identified using NMR spectroscopy and collected as a white solid with a yield of 62 % (0.51 g, 1.25 mmol); melting point: 166 °C; ^{1}H NMR (400 MHz, 298 K, DMSO-d_6): δ: 9.10 (s, 1H), 7.56 (s, 4H), 7.33 – 7.21 (m, 5H), 6.53 (d, *J* = 8.12 Hz, 1H), 4.41, (q, *J* = 21.40 Hz, 1H), 3.00 (d, *J* = 6.88 Hz, 2H), 1.35 (s, 9H); ^{13}C{^{1}H} NMR (100 MHz, 298 K, DMSO-d_6): δ: 171.5 (C=O), 154.7 (C=O), 144.3 (ArC), 137.3 (ArC), 129.8 (ArCH), 128.7 (ArCH), 127.1 (ArCH), 126.5 (q, *J* = 3.79 Hz, ArCH), 121.8 (q, *J* = 31.78 Hz, CF_3), 123.7 (ArC), 117.7 (ArCH), 81.5 (C), 54.6 (CH), 38.0 (CH_2), 28.0 (CH_3); IR (film): ν = 3381 (NH stretch), 2985, 1681, 1537, 1328, 1317, 1236, 1159, 1107, 1066, 835, 700, 609; HRMS for the *tert*-butyl carboxylate-urea ($C_{21}H_{23}F_3N_2O_3$) (ESI$^-$): m/z: act: 408.1502 $[M]^-$ cal: 408.1661 $[M]^-$.

Compound 9: Zinc bromide (2.25 g, 10 mmol) was added to **8** (0.51 g, 1.25 mmol) in dichloromethane (5 mL) and stirred at room temperature for 24 hours. The reaction mixture was then quenched with water (20 mL) and stirred at room temperature for 4 hours, resulting in precipitation. The precipitate was collected via filtration and TBA hydroxide in methanol (0.51 mL, 1M) was added. The mixture was taken to dryness, and the pure product was collected via flash column chromatography with 100 % ethyl acetate followed by 100 % methanol. The methanol fraction was taken to dryness to give the pure product as a brown solid with a yield of 54 % (0.28 g, 0.47 mmol); melting point: > 200 °C; ^{1}H NMR (400 MHz, 298 K, DMSO-d_6): δ: 9.71 (s, 1H), 7.63 (d, *J* = 8.60 Hz, 2H), 7.50 (d, *J* = 8.72 Hz, 2H), 7.31 - 7.05 (m, 5H), 6.57 (s, 1H), 3.87 (q, *J* = 14.44 Hz, 1H), 3.18 – 2.98 (m, 10 H), 1.60 – 1.51 (m, 8H), 1.35 – 1.25 (m, 8H), 0.93 (t, *J* = 14.88 Hz, 12H); ^{13}C{^{1}H} NMR (100 MHz, 298 K, DMSO-d_6): δ: 173.2 (C=O), 155.2 (C=O), 146.0 (ArC), 140.2 (ArC), 130.1 (ArCH), 128.0 (ArCH), 126.0 (q, *J* = 3.62 Hz, ArCH), 125.8 (ArCH), 124.0 (ArC), 120.2 (q, *J* = 31.54 Hz, CF_3), 117.3 (ArCH), 58.0 (t, *J* = 2.46 Hz, CH_2), 56.7 (CH), 39.1 (CH_2), 23.5 (CH_2), 19.7 (CH_2), 13.9 (CH_3); IR (film): ν = 3309 (NH stretch), 2962, 1695, 1606, 1489, 1381, 1319, 1107, 1066, 846, 702; HRMS for the carboxylate-urea ion ($C_{17}H_{14}F_3N_2O_3^-$) (ESI$^-$): m/z: act: 351.0937 $[M]^-$ cal: 351.0962 $[M]^-$.

Section S6: Characterisation NMR spectra

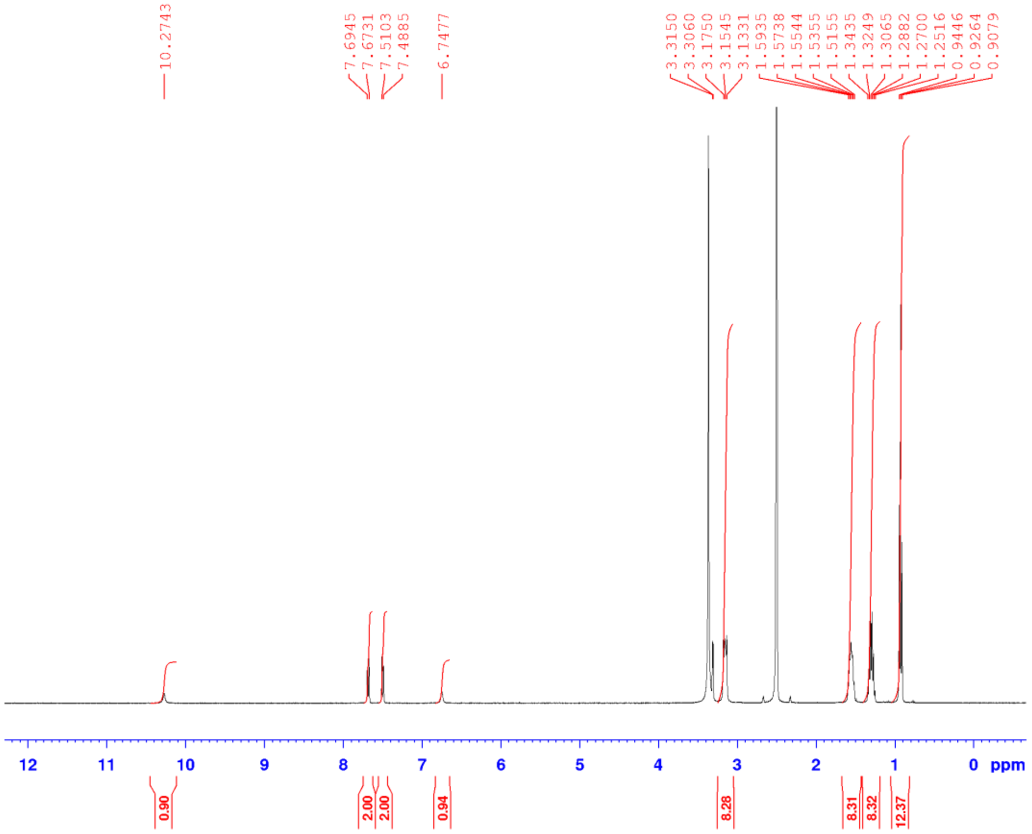

Figure S2 - ^{1}H NMR spectrum of **1** in DMSO-d_6 conducted at 298 K.

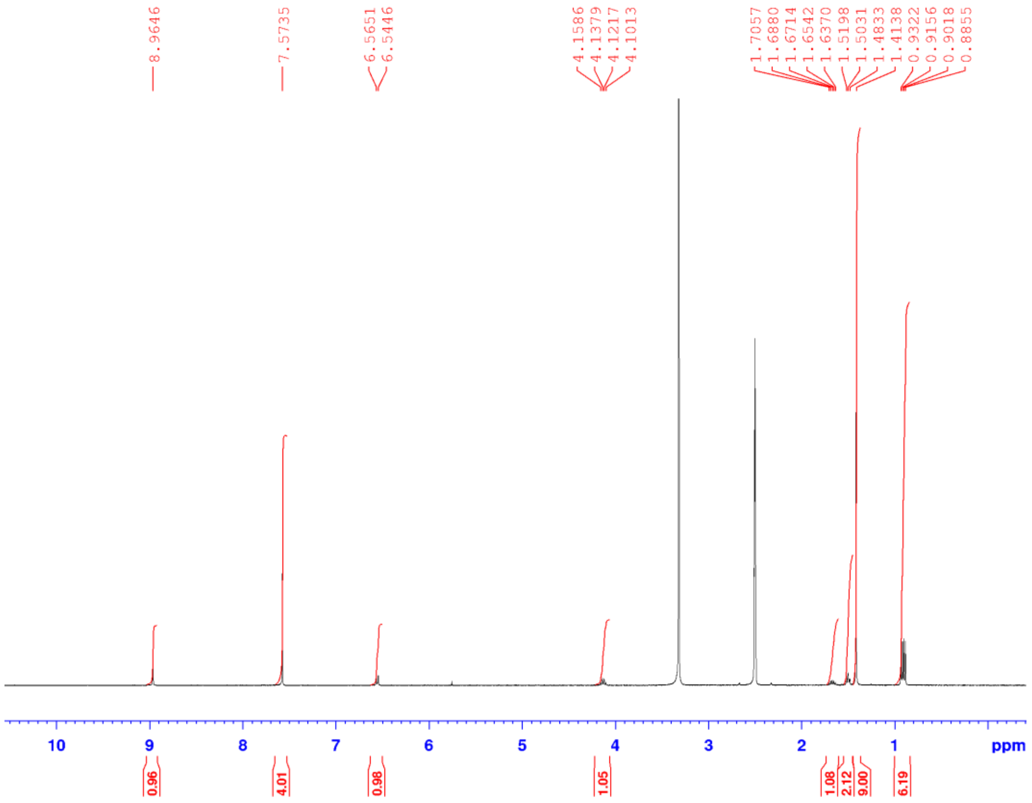

Figure S3 - ^{1}H NMR spectrum of **2** in DMSO-d_6 conducted at 298 K.

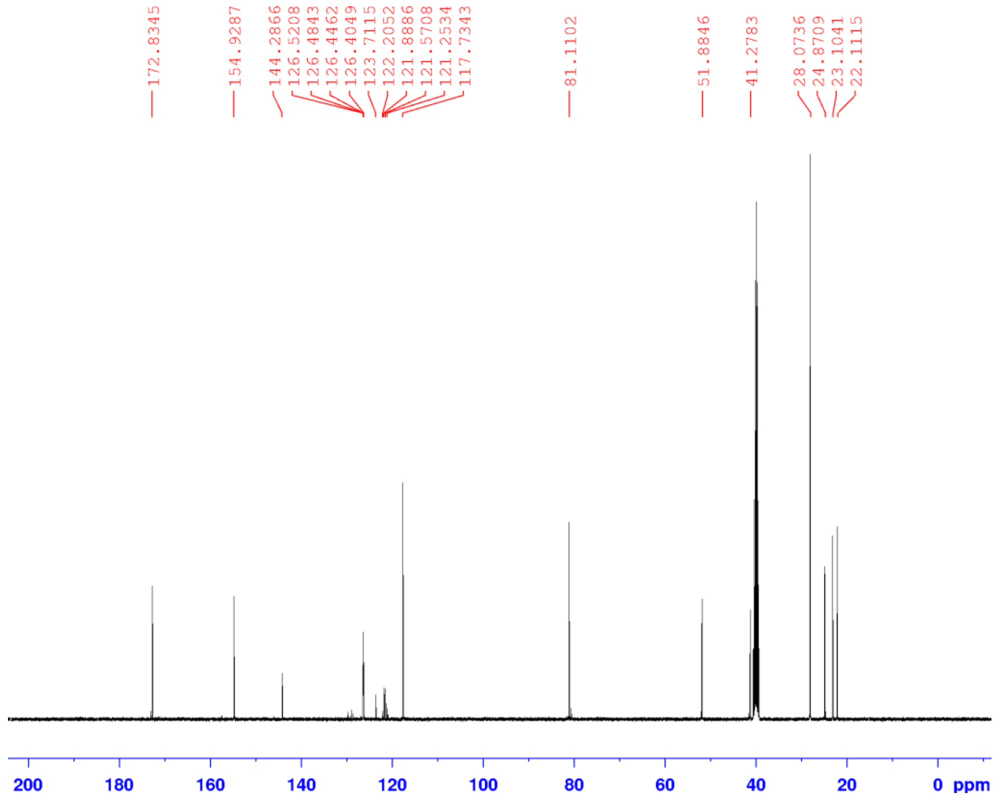

Figure S4 - $^{13}C\{^1H\}$ NMR spectrum of **2** in DMSO-d_6 conducted at 298 K.

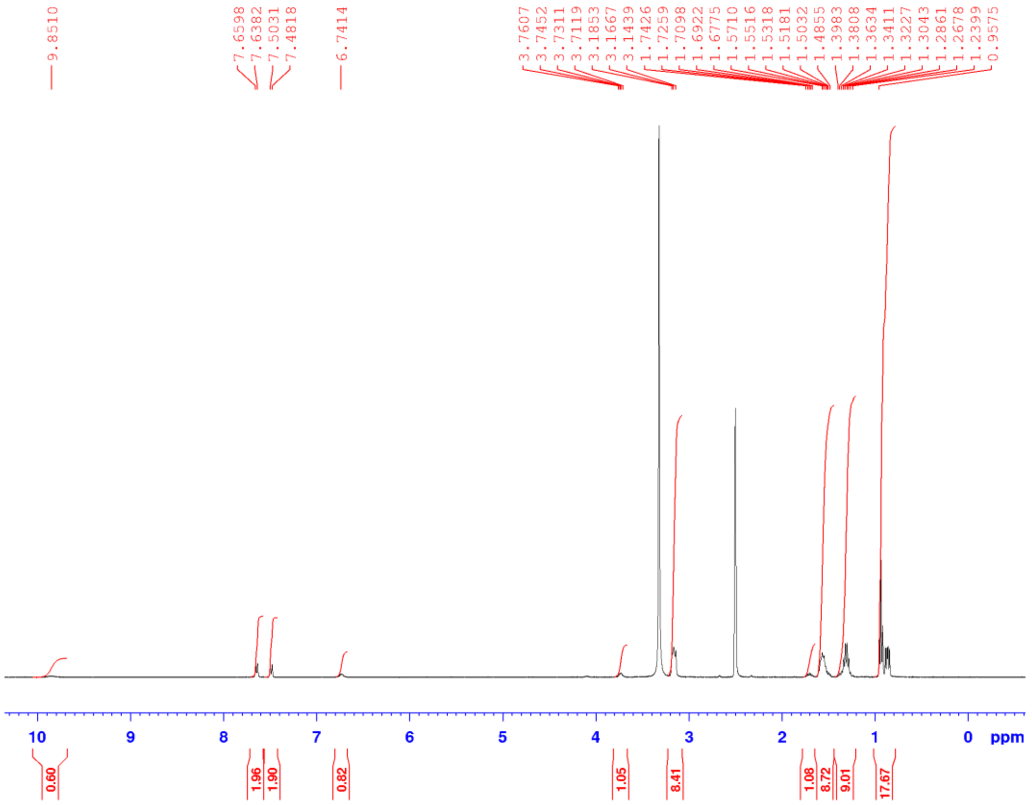

Figure S5 - ^1H NMR spectrum of **3** in DMSO-d_6 conducted at 298 K.

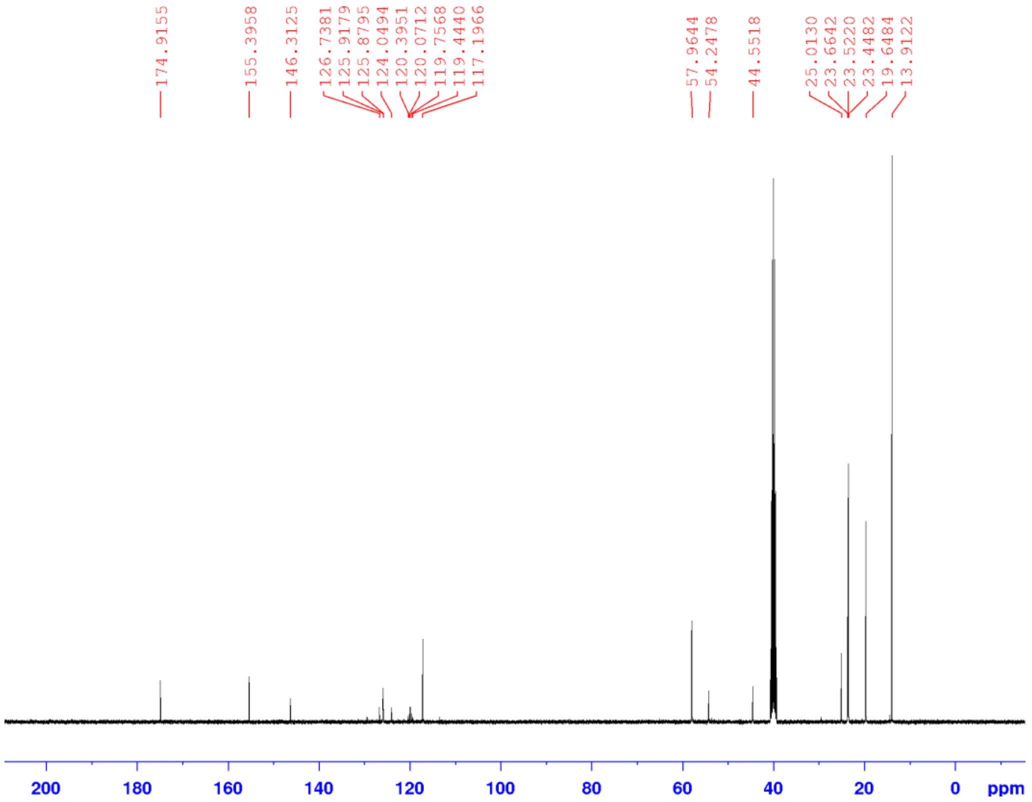

Figure S6 - $^{13}C\{^{1}H\}$ NMR spectrum of **3** in DMSO-d_6 conducted at 298 K.

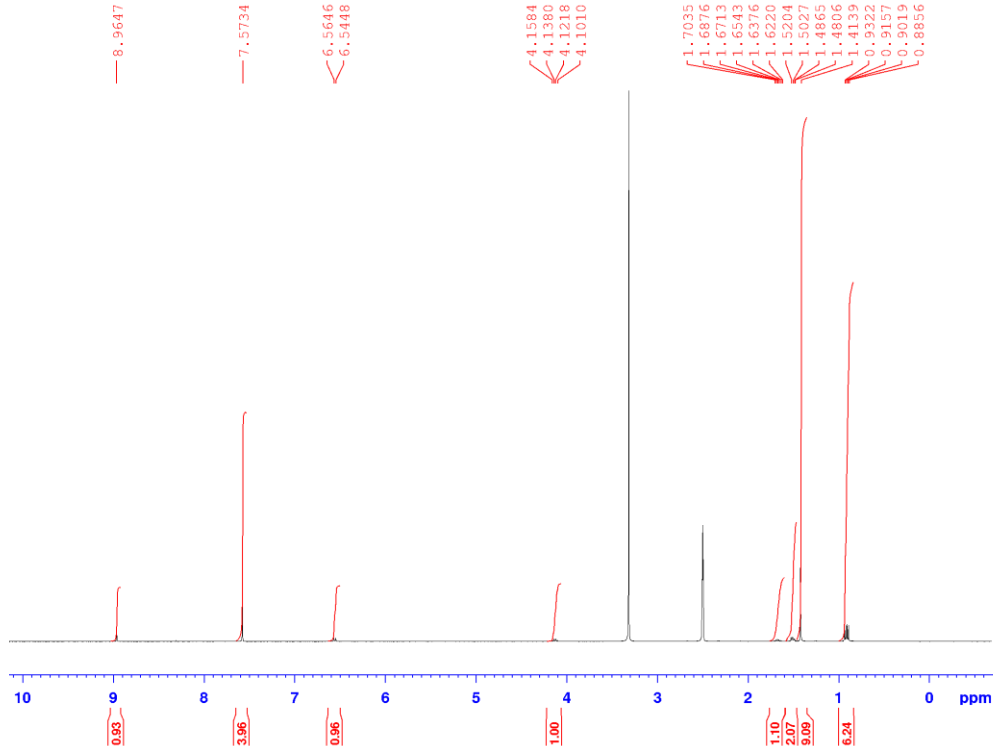

Figure S7 - ^{1}H NMR spectrum of **4** in DMSO-d_6 conducted at 298 K.

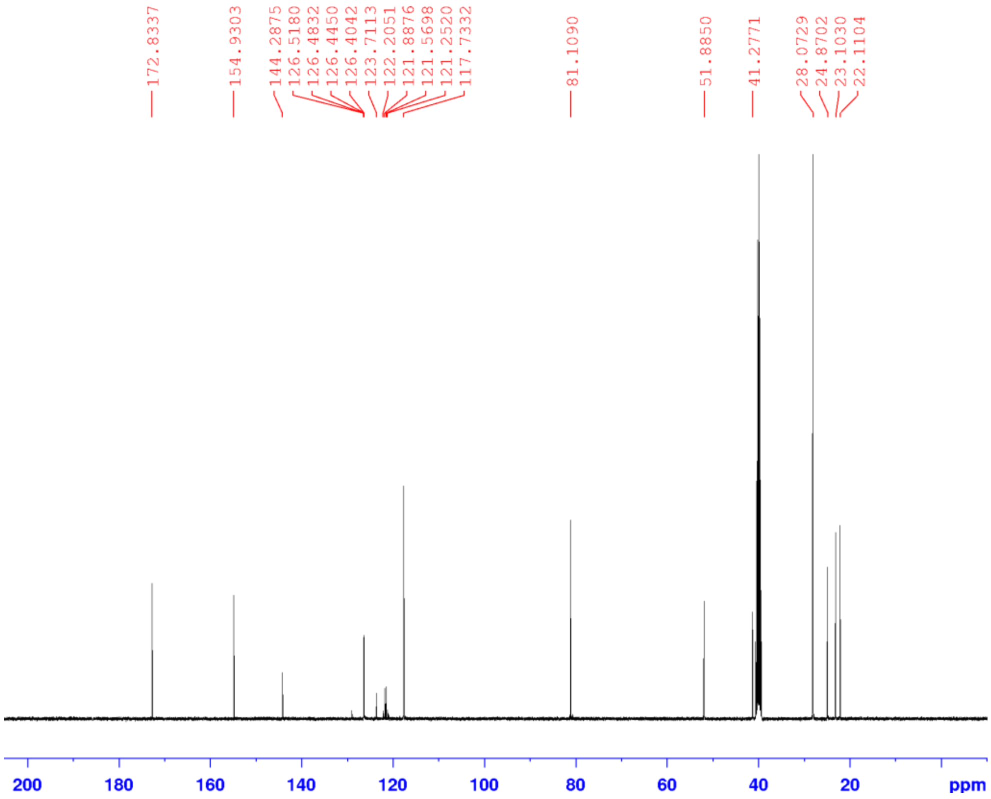

Figure S8 - $^{13}C\{^{1}H\}$ NMR spectrum of **4** in DMSO-d_6 conducted at 298 K.

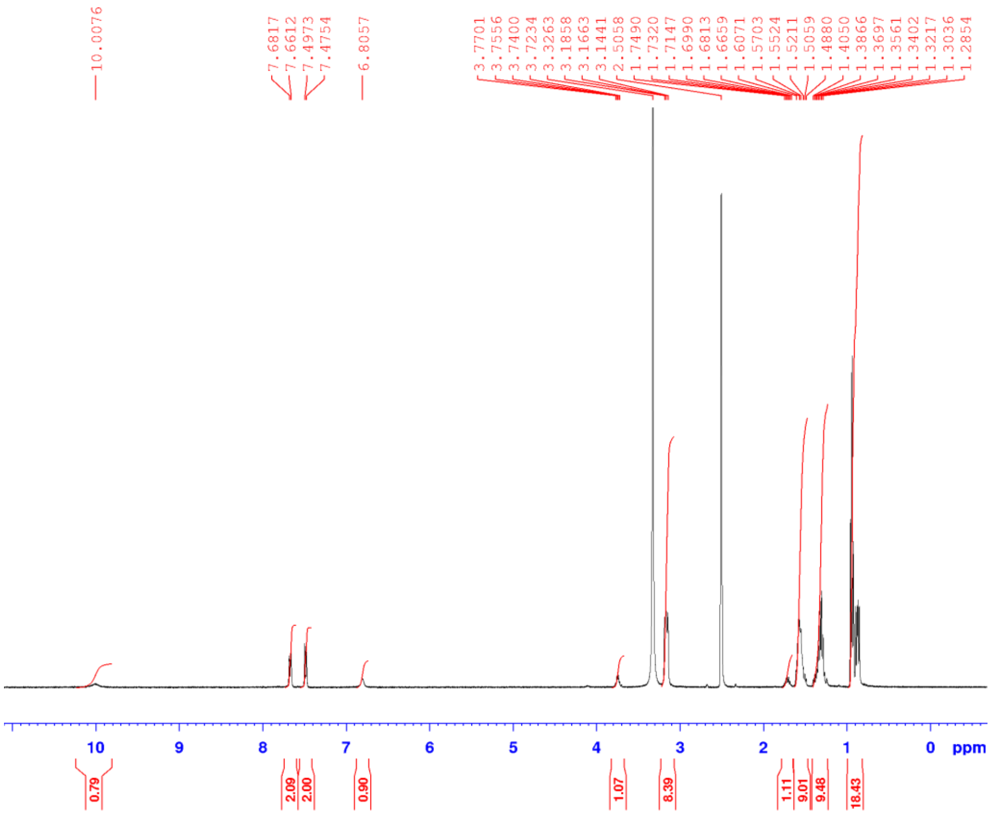

Figure S9 - ^{1}H NMR spectrum of **5** in DMSO-d_6 conducted at 298 K.

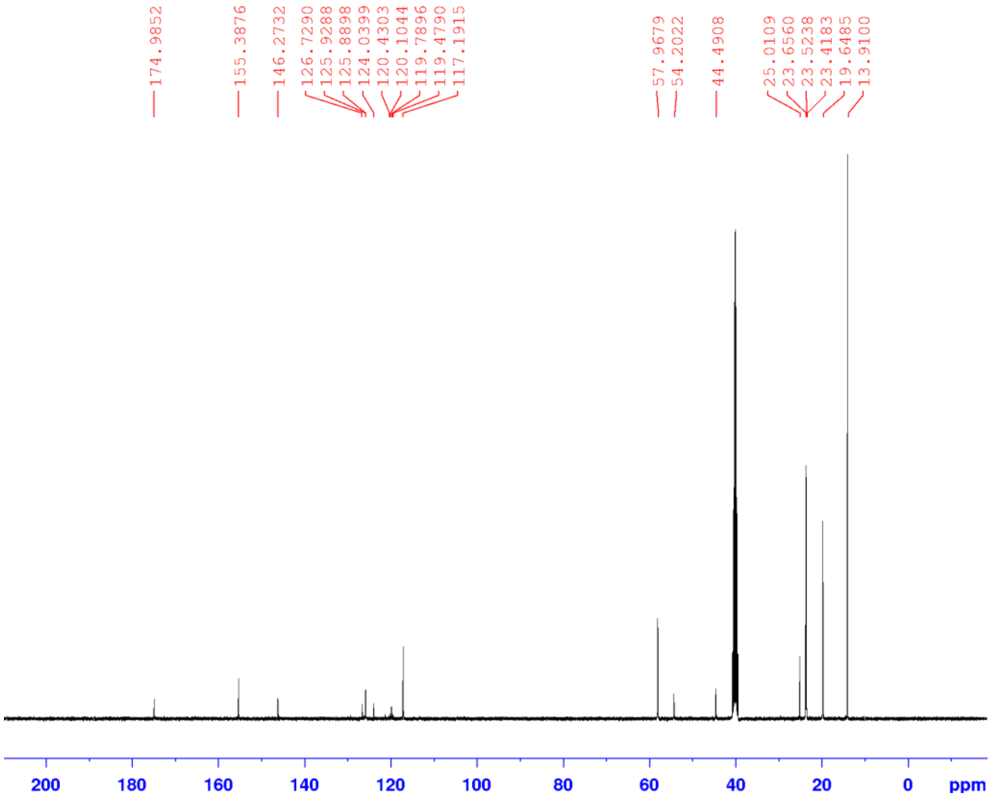

Figure S10 - $^{13}C\{^{1}H\}$ NMR spectrum of **5** in DMSO-d_6 conducted at 298 K.

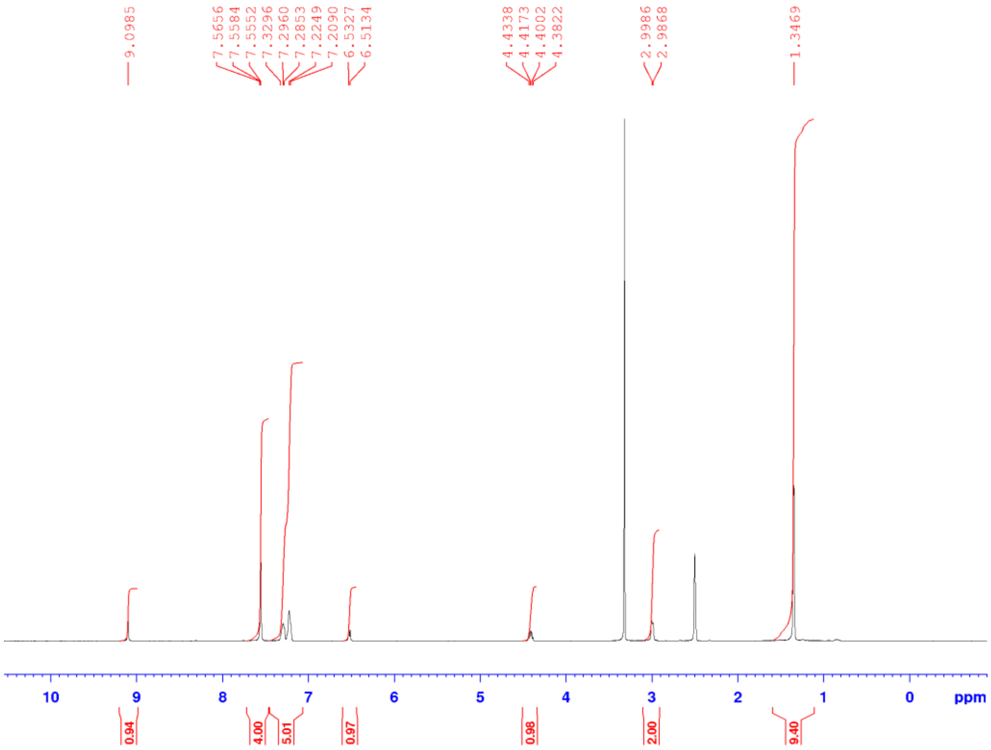

Figure S11 - ^{1}H NMR spectrum of **6** in DMSO-d_6 conducted at 298 K.

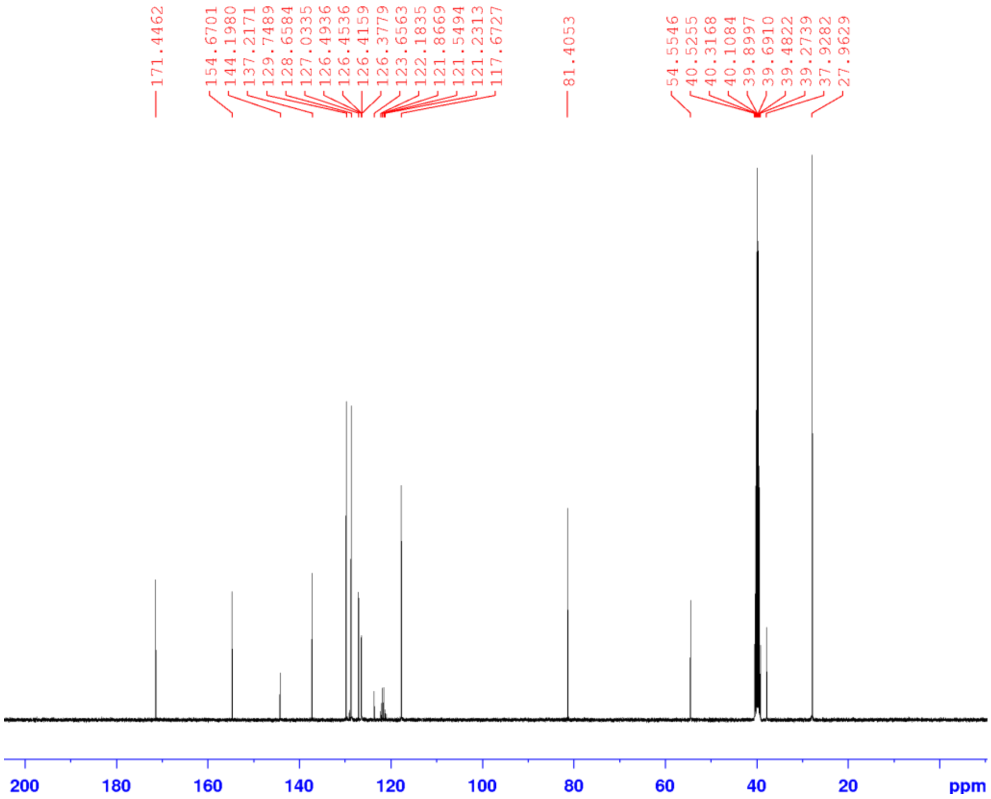

Figure S12 - $^{13}C\{^{1}H\}$ NMR spectrum of **6** in DMSO-d_6 conducted at 298 K.

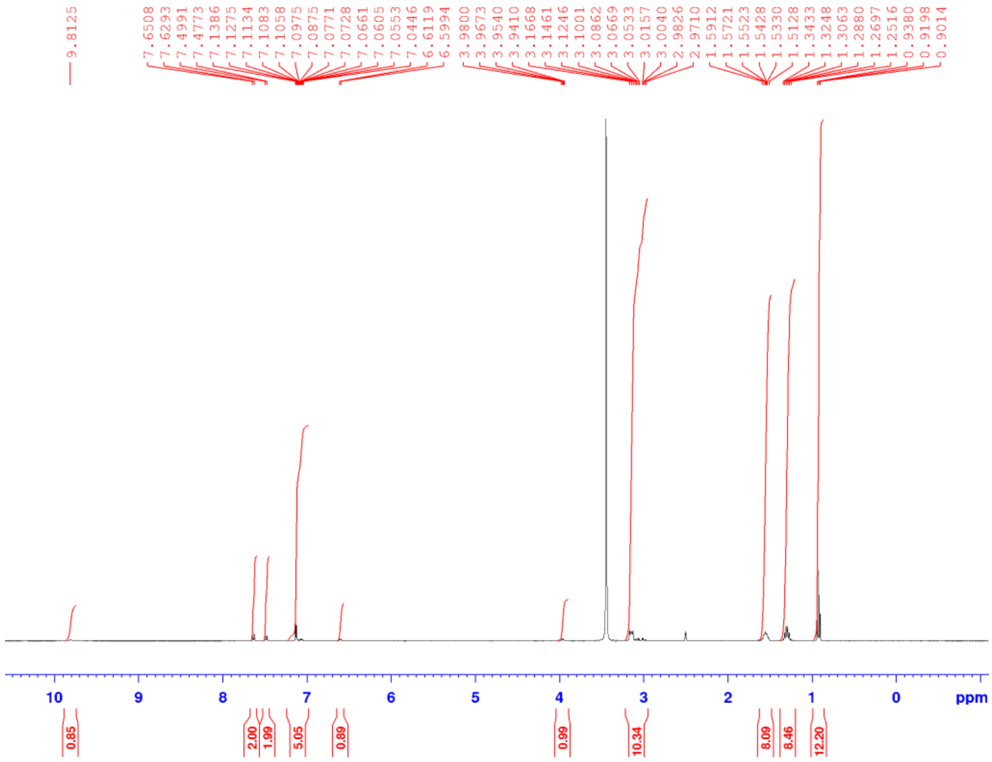

Figure S13 - ^{1}H NMR spectrum of **7** in DMSO-d_6 conducted at 298 K.

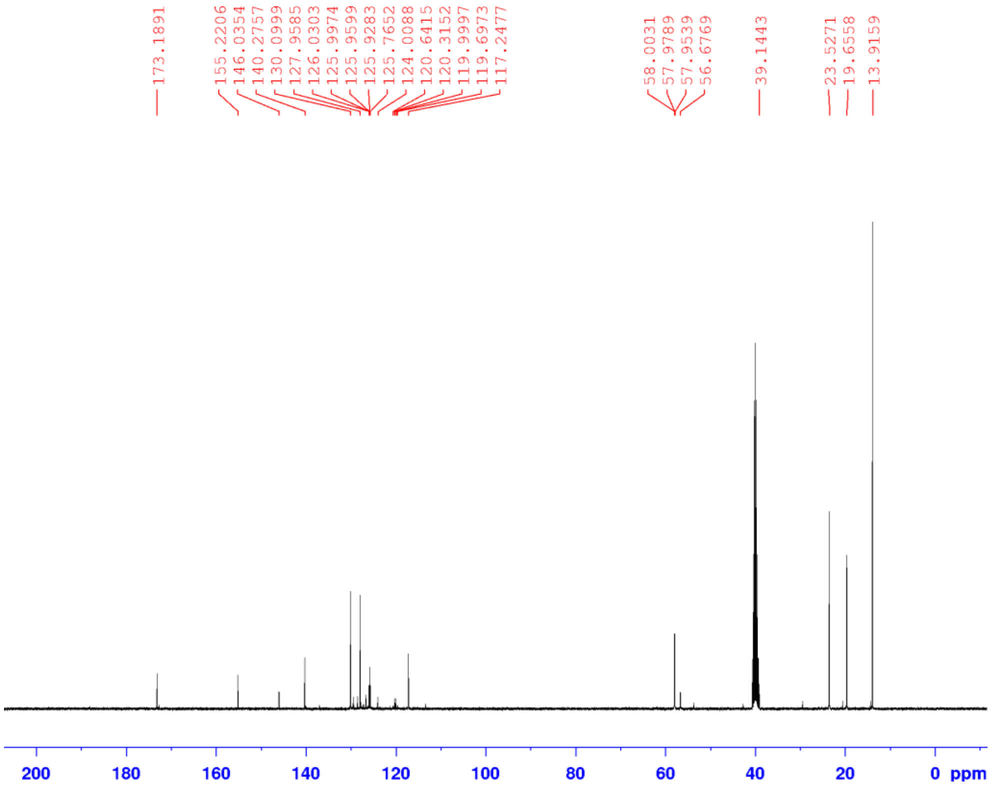

Figure S14 - $^{13}C\{^{1}H\}$ NMR spectrum of **7** in DMSO-d_6 conducted at 298 K.

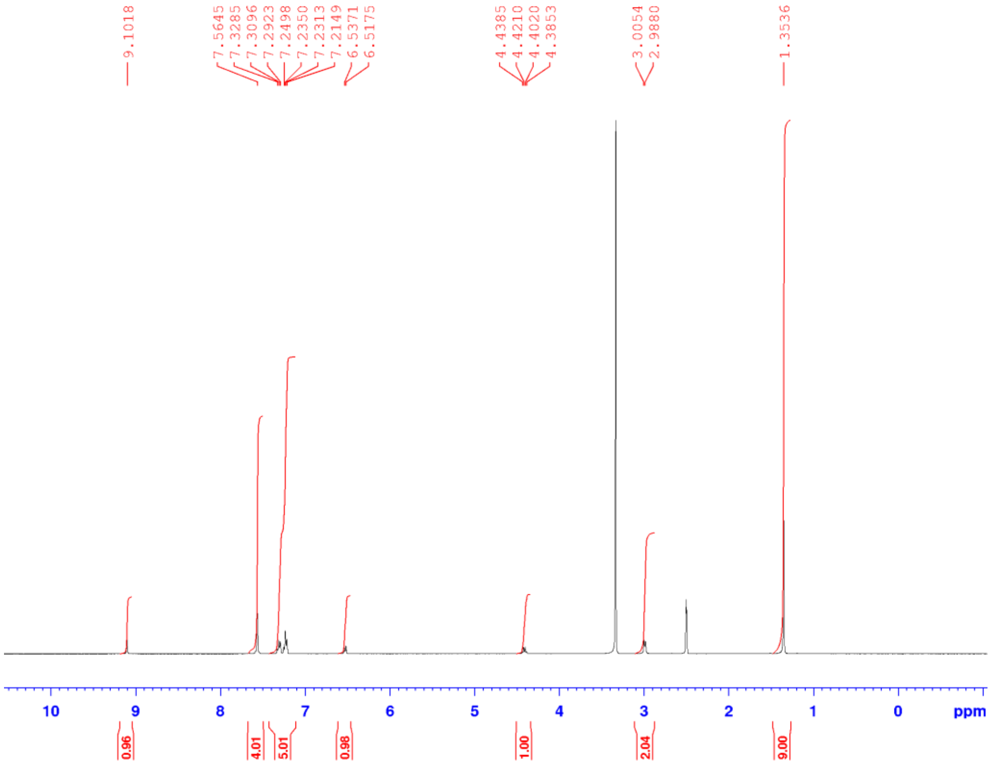

Figure S15 - ^{1}H NMR spectrum of **8** in DMSO-d_6 conducted at 298 K.

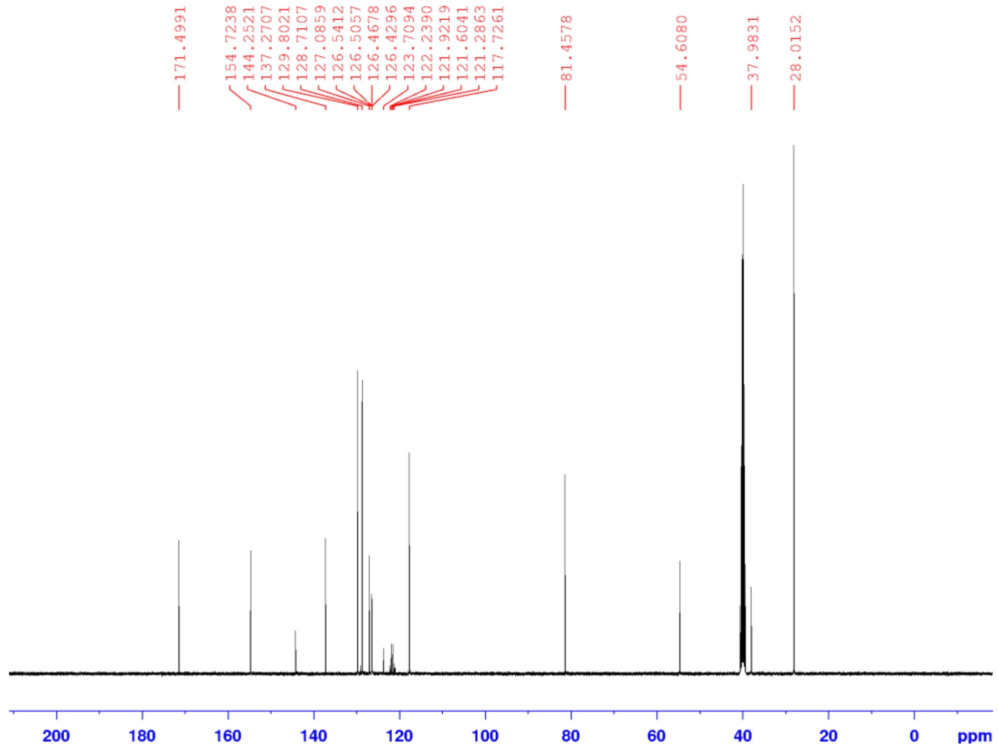

Figure S16 - $^{13}C\{^{1}H\}$ NMR spectrum of **8** in DMSO-d_6 conducted at 298 K.

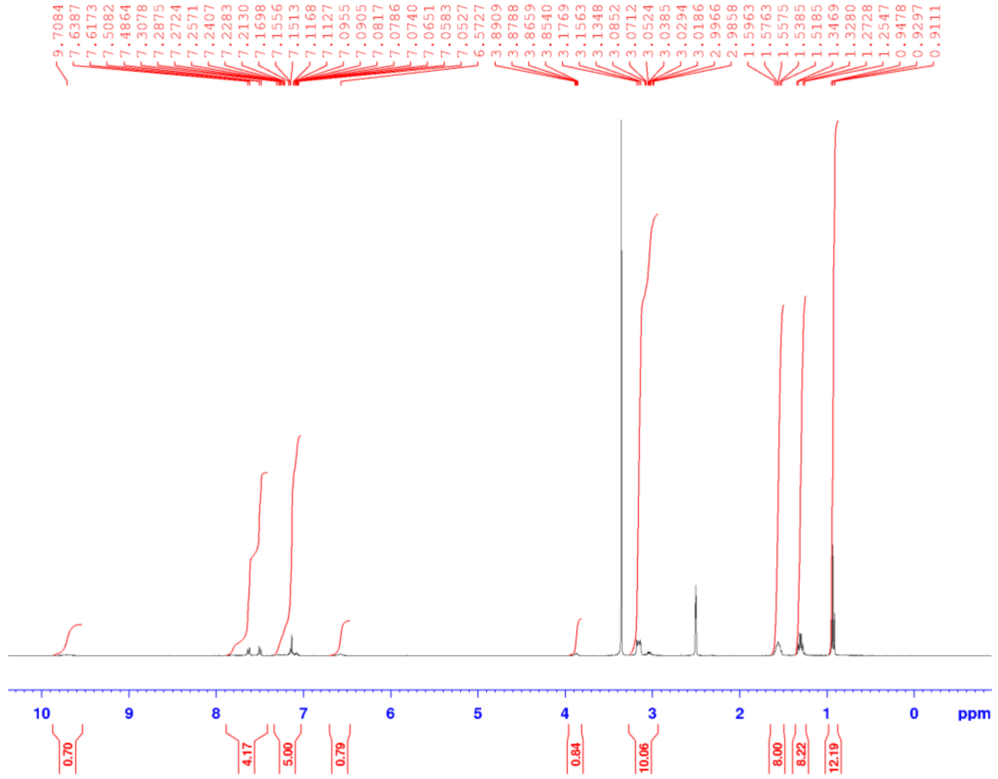

Figure S17 - ^{1}H NMR spectrum of **9** in DMSO-d_6 conducted at 298 K.

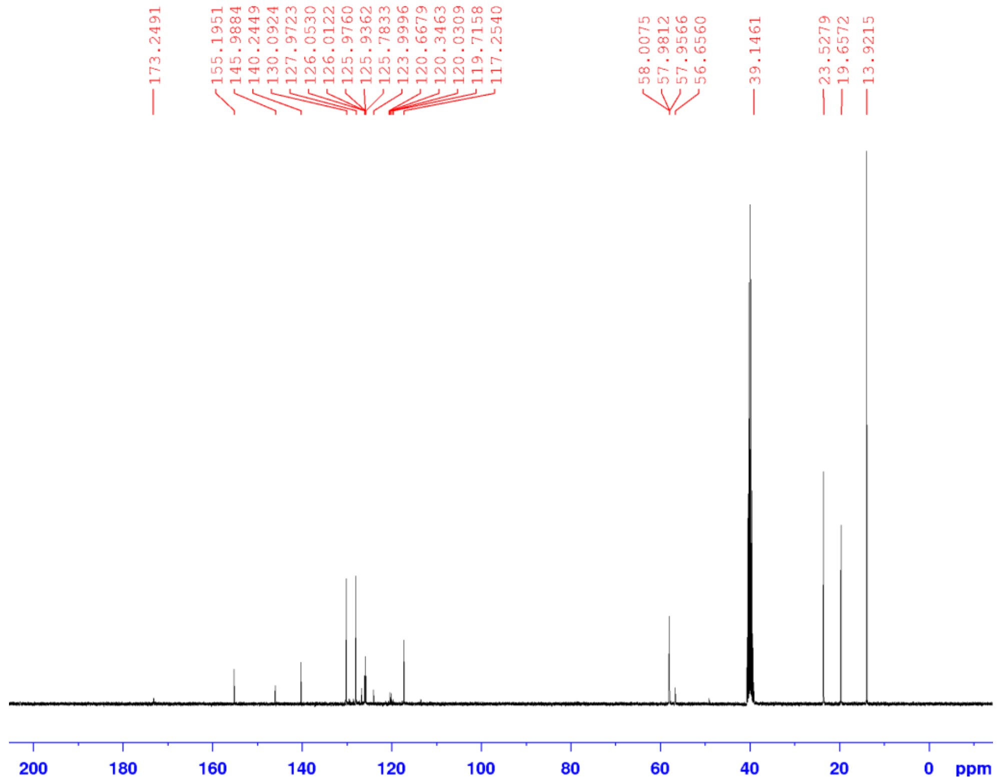

Figure S18 - $^{13}C\{^{1}H\}$ NMR spectrum of **9** in DMSO-d_6 conducted at 298 K.

Section S7: Quantitative ^{1}H NMR studies

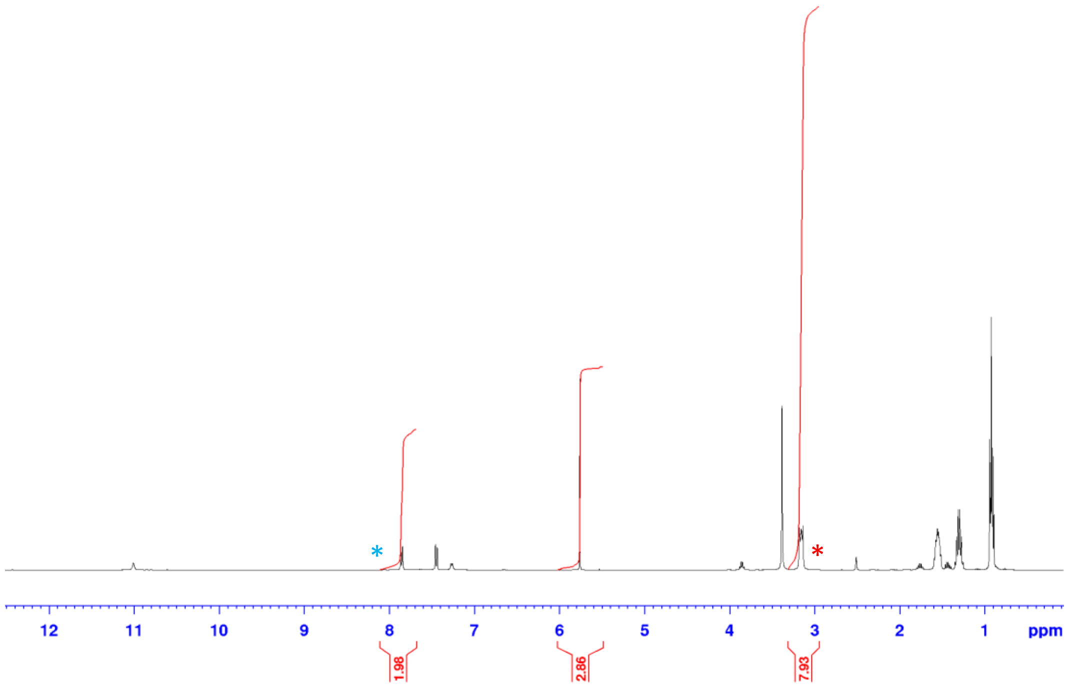

Figure S19 - ^{1}H NMR spectrum (d_1 = 60 s) of **3** (112 mM) in DMSO-d_6/ 1.0 % DCM. Comparative integration indicates 0 % of the anionic component (blue) and 0 % of the cationic component (red) of **3** has become NMR silent.

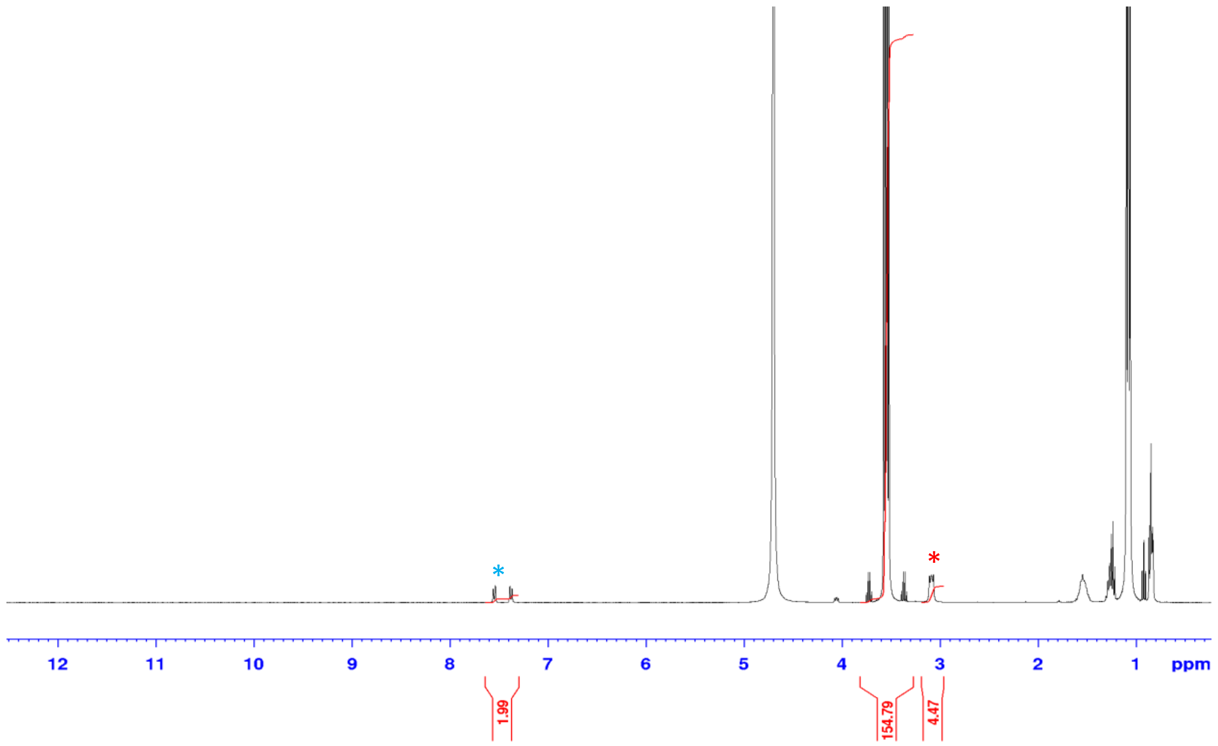

Figure S20 - ^{1}H NMR spectrum (d_1 = 60 s) of **3** (5.56 mM) in D_2O/ 5.0 % EtOH. Comparative integration indicates 50 % of the anionic component (blue) and 44 % of the cationic component (red) of **3** has become NMR silent.

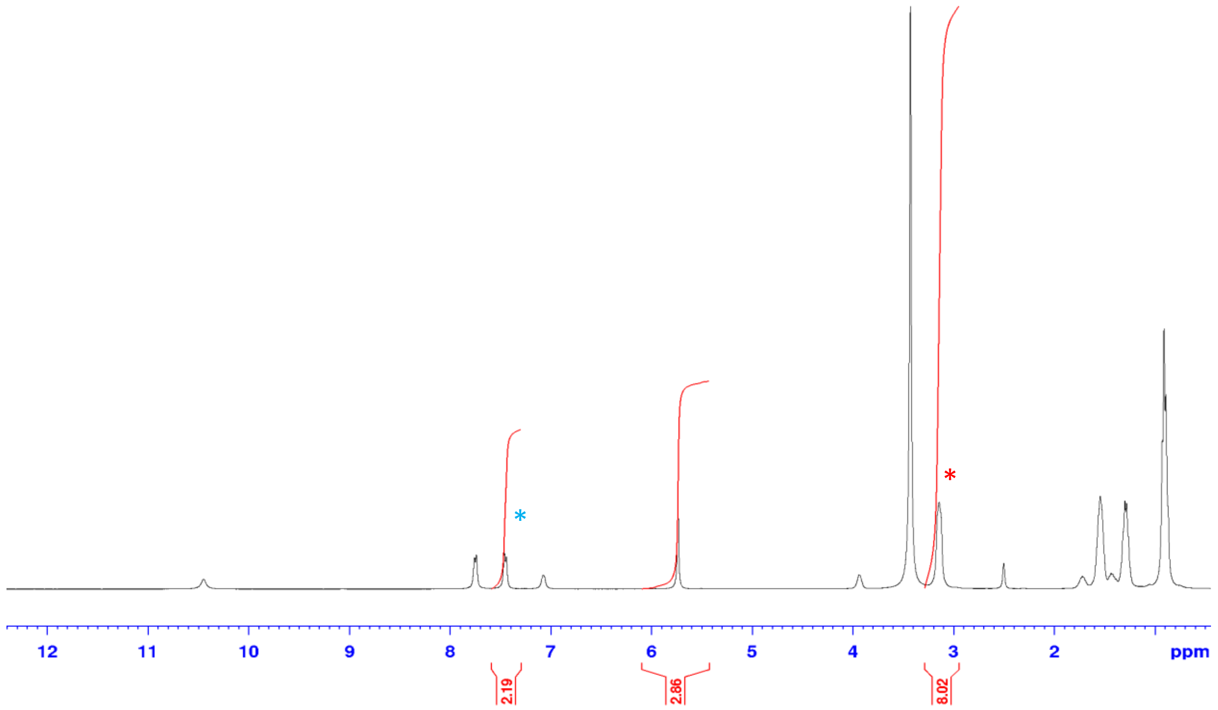

Figure S21 - ^{1}H NMR spectrum (d_1 = 60 s) of **5** (112 mM) in DMSO-d_6/ 1.0 % DCM. Comparative integration indicates 0 % of the anionic component (blue) and 0 % of the cationic component (red) of **5** has become NMR silent.

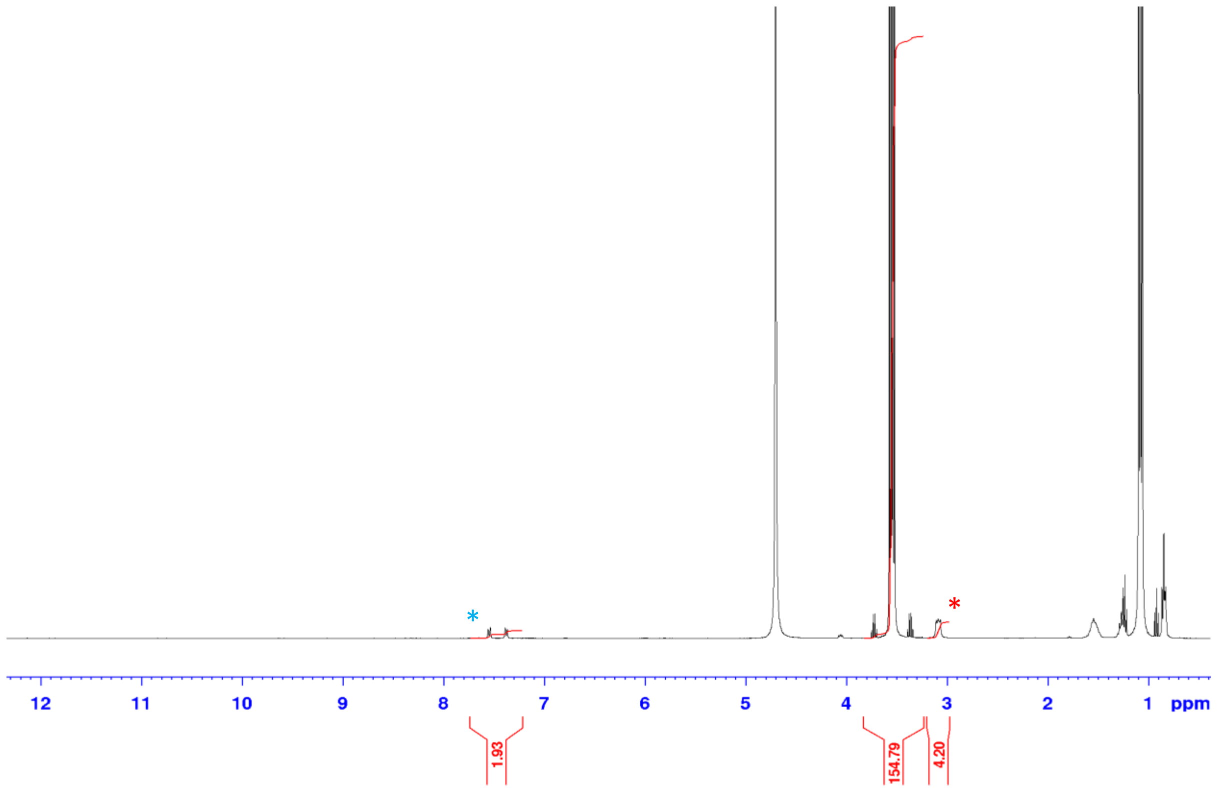

Figure S22 - ^{1}H NMR spectrum (d_1 = 60 s) of **5** (5.56 mM) in D_2O/ 5.0 % EtOH. Comparative integration indicates 52 % of the anionic component (blue) and 48 % of the cationic component (red) of **5** has become NMR silent.

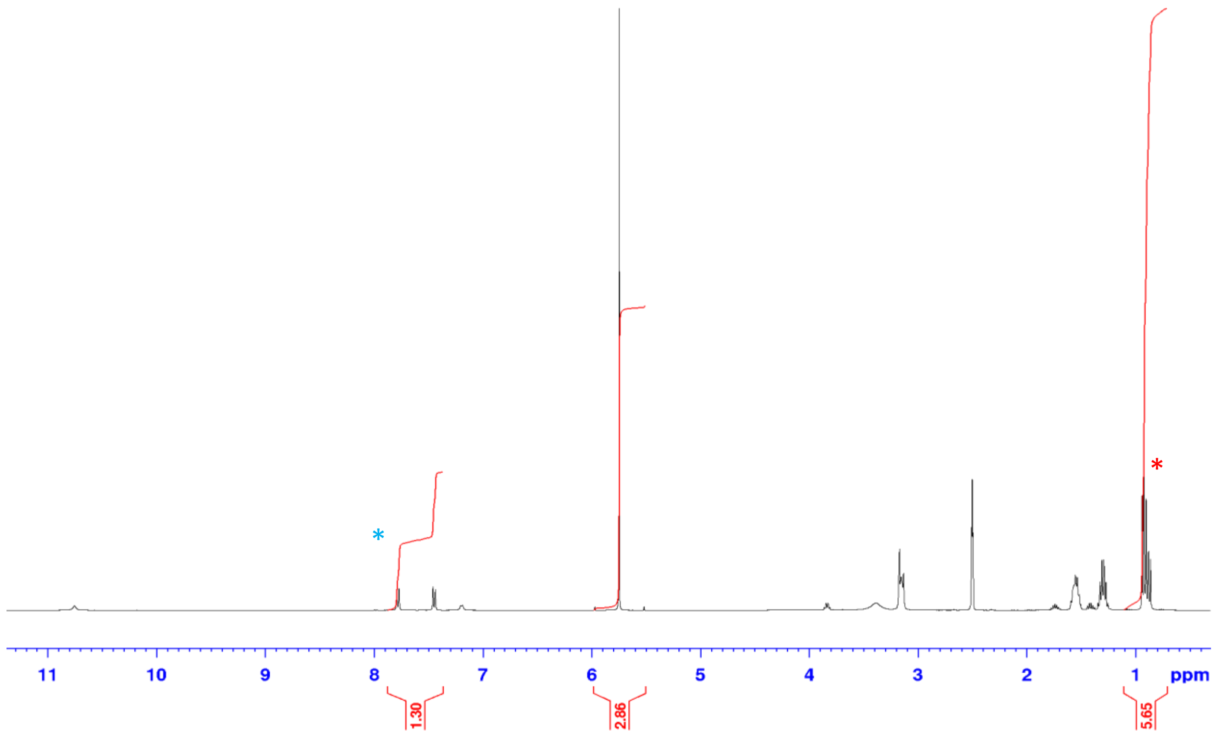

Figure S23 - ^{1}H NMR spectrum (d_1 = 60 s) of **3** + **5** (112 mM) in DMSO-*d*$_6$/ 1.0 % DCM. Comparative integration indicates 68 % of the anionic component (blue) and 69 % of the cationic component (red) has become NMR silent.

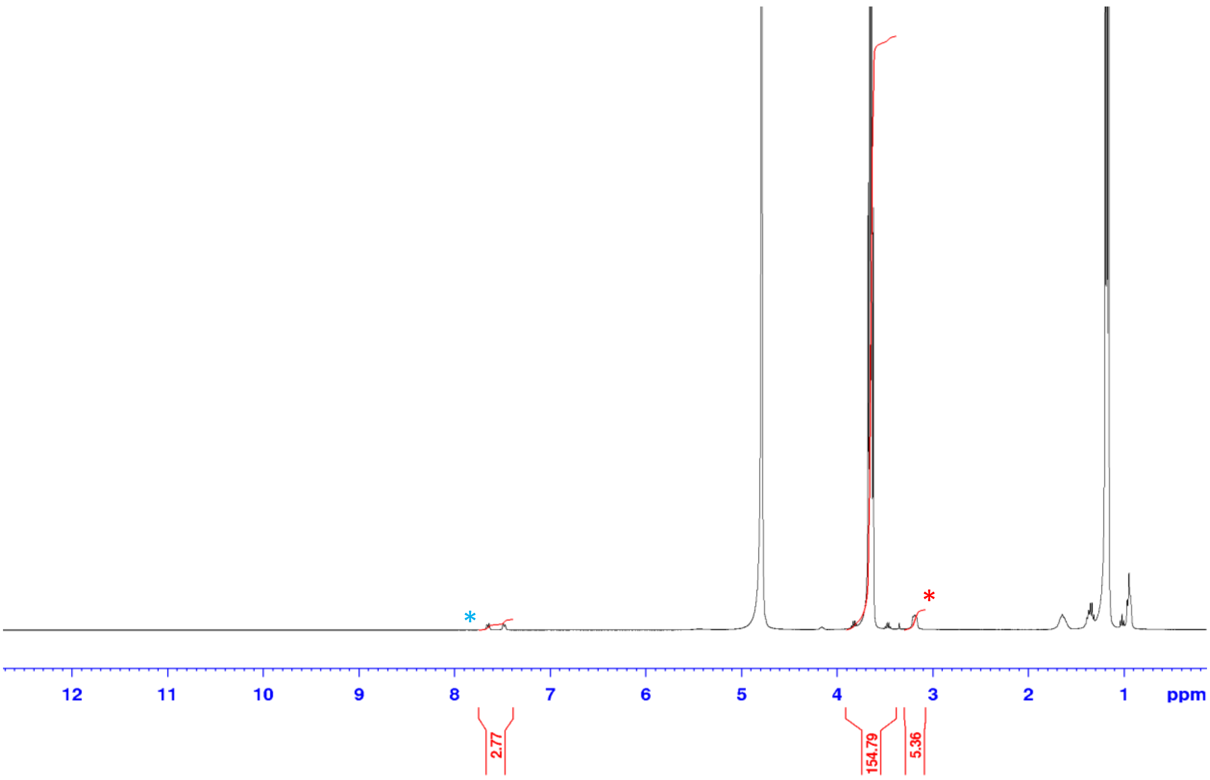

Figure S24 - ^{1}H NMR spectrum (d_1 = 60 s) of **3** + **5** (5.56 mM) in D_2O/ 5.0 % EtOH. Comparative integration indicates 31 % of the anionic component (blue) and 33 % of the cationic component (red) has become NMR silent.

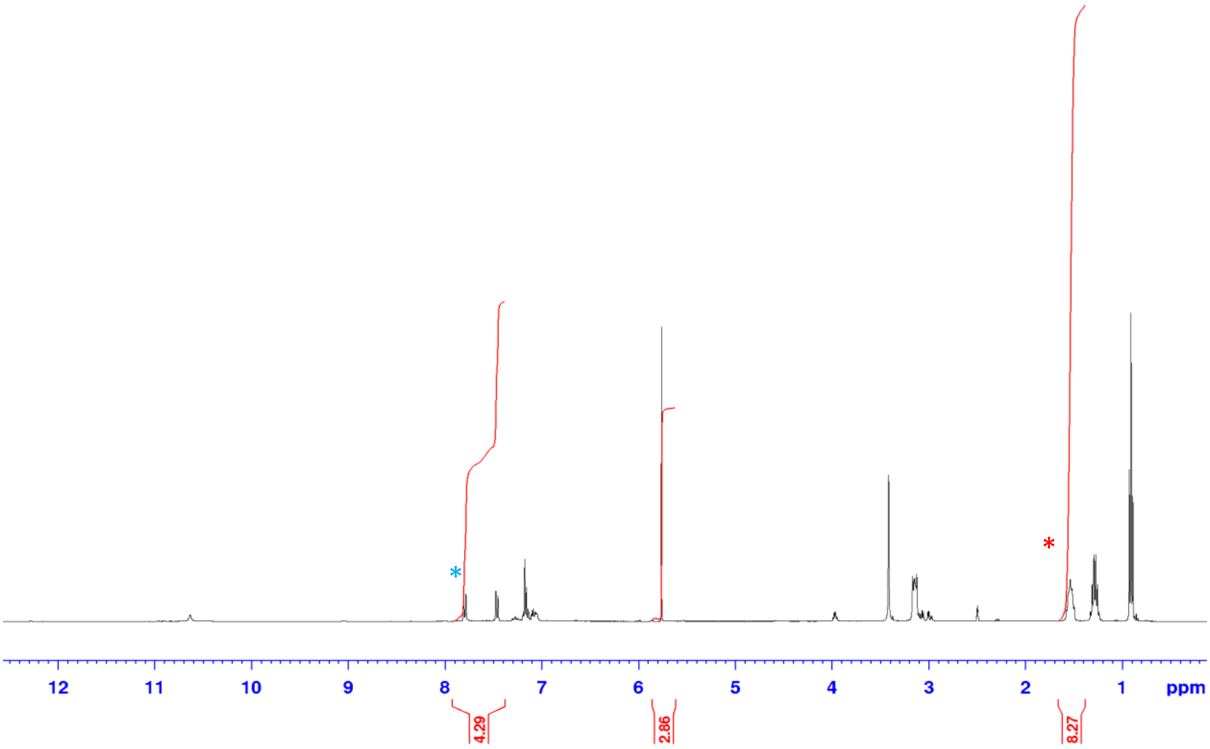

Figure S25 - ^{1}H NMR spectrum (d_1 = 60 s) of **7** (112 mM) in DMSO-d_6/1.0 % DCM. Comparative integration indicates 0 % of the anionic component (blue) and 0 % of the cationic component (red) of **7** has become NMR silent.

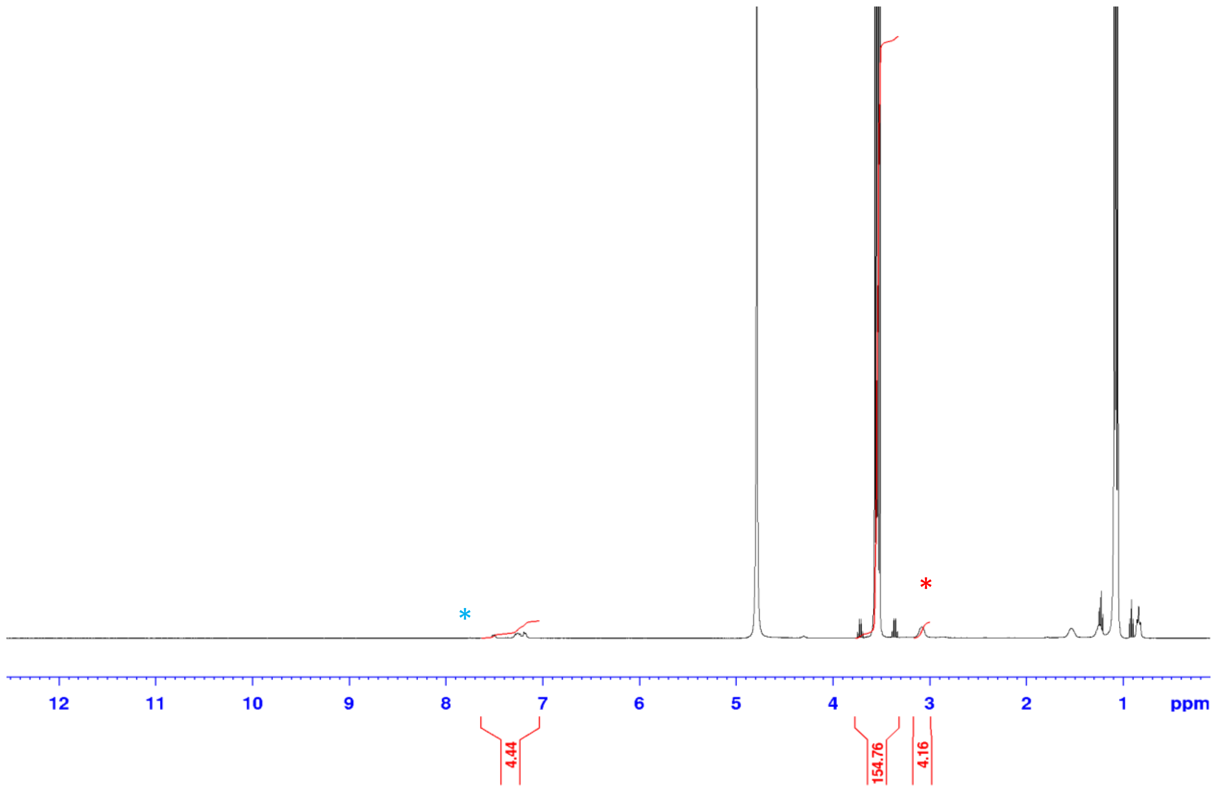

Figure S26 - ^{1}H NMR spectrum (d_1 = 60 s) of **7** (5.56 mM) in D_2O/ 5.0 % EtOH. Comparative integration indicates 51 % of the anionic component (blue) and 48 % of the cationic component (red) of **7** has become NMR silent.

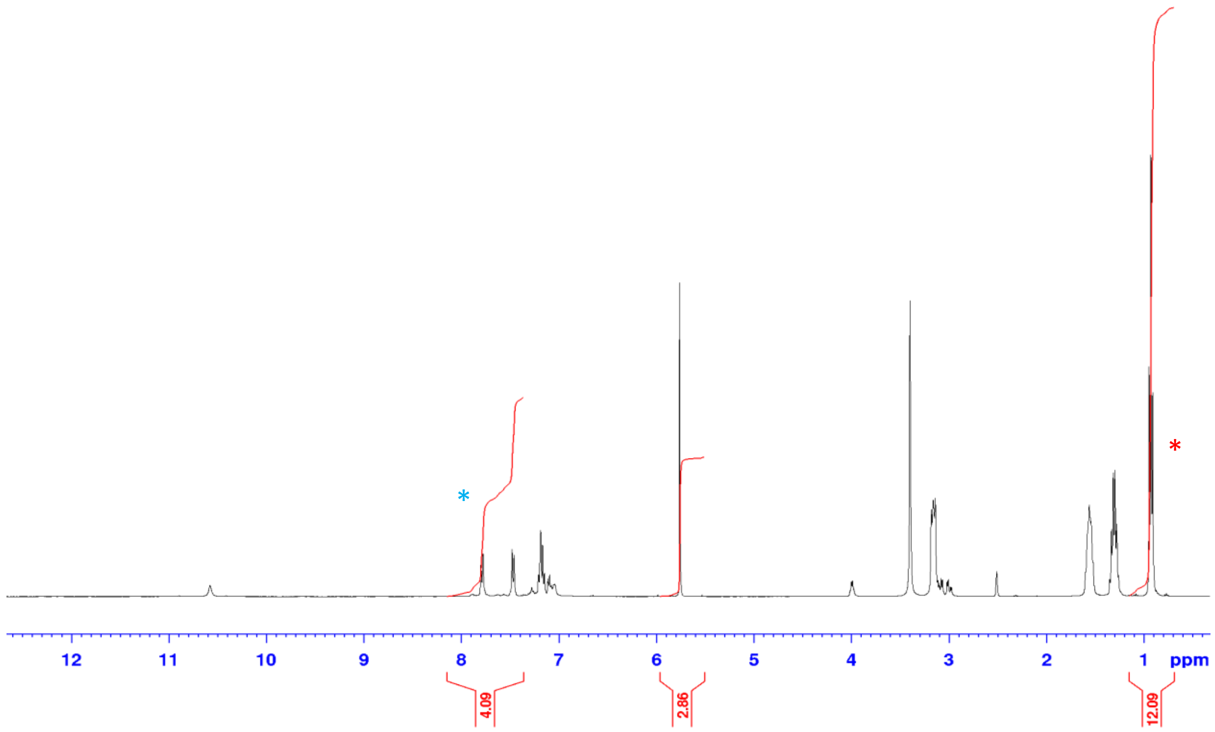

Figure S27 - ^{1}H NMR spectrum (d_1 = 60 s) of **9** (112 mM) in DMSO-d_6/ 1.0 % DCM. Comparative integration indicates 0 % of the anionic component (blue) and 0 % of the cationic component (red) of **9** has become NMR silent.

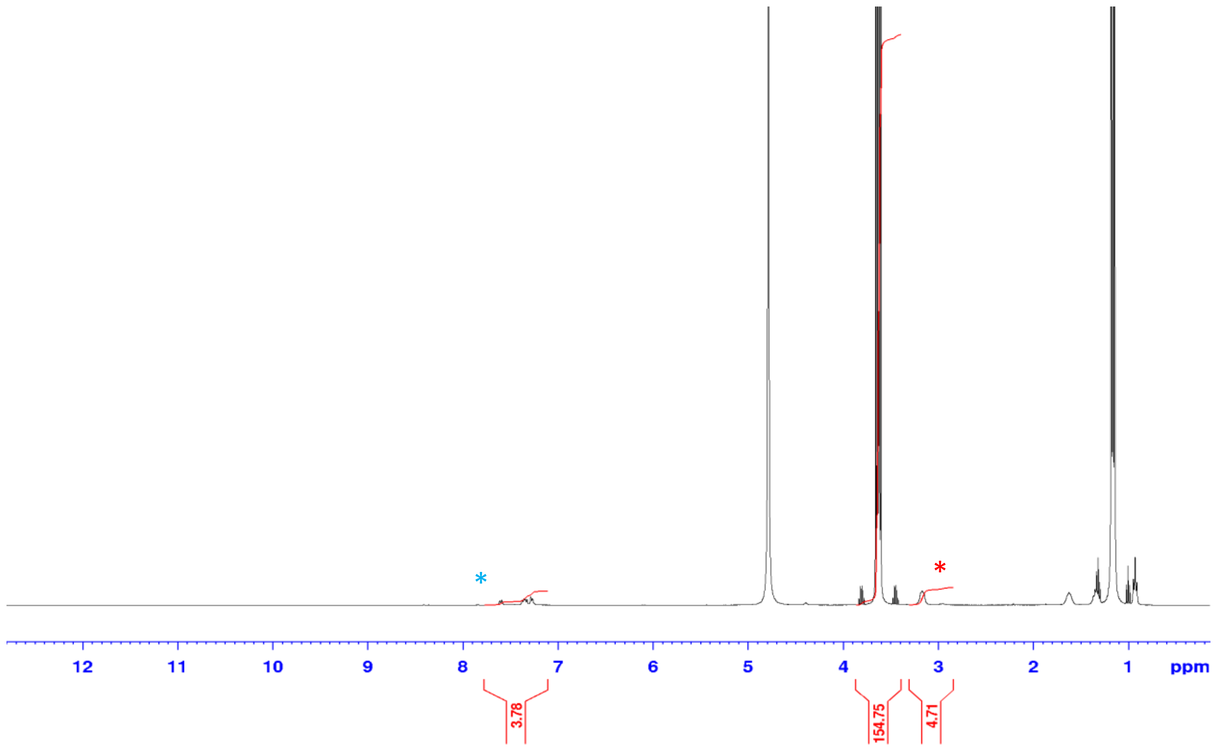

Figure S28 - ^{1}H NMR spectrum (d_1 = 60 s) of **9** (5.56 mM) in D_2O/ 5.0 % EtOH. Comparative integration indicates 58 % of the anionic component (blue) and 53 % of the cationic component (red) of **9** has become NMR silent.

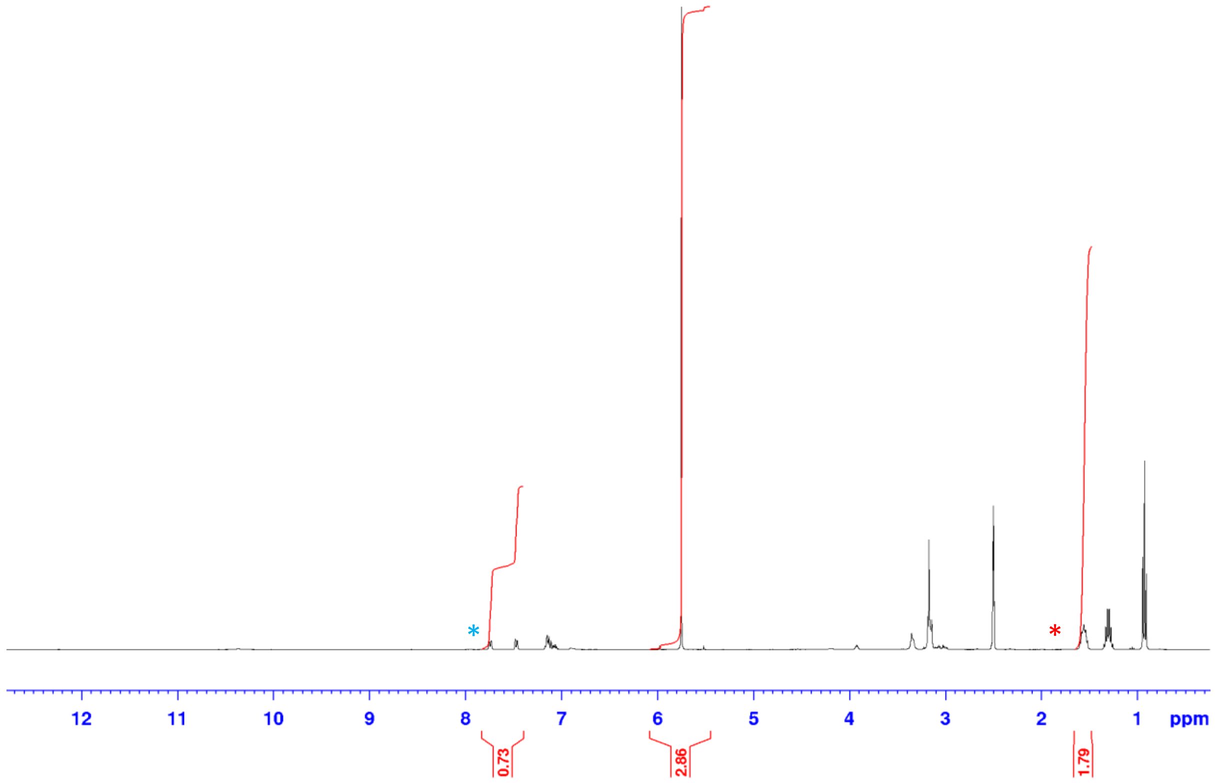

Figure S29 - ^{1}H NMR spectrum (d_1 = 60 s) of **7** and **9** (112 mM) in DMSO-d_6/ 1.0 % DCM. Comparative integration indicates 82 % of the anionic component (blue) and 78 % of the cationic component (red) has become NMR silent.

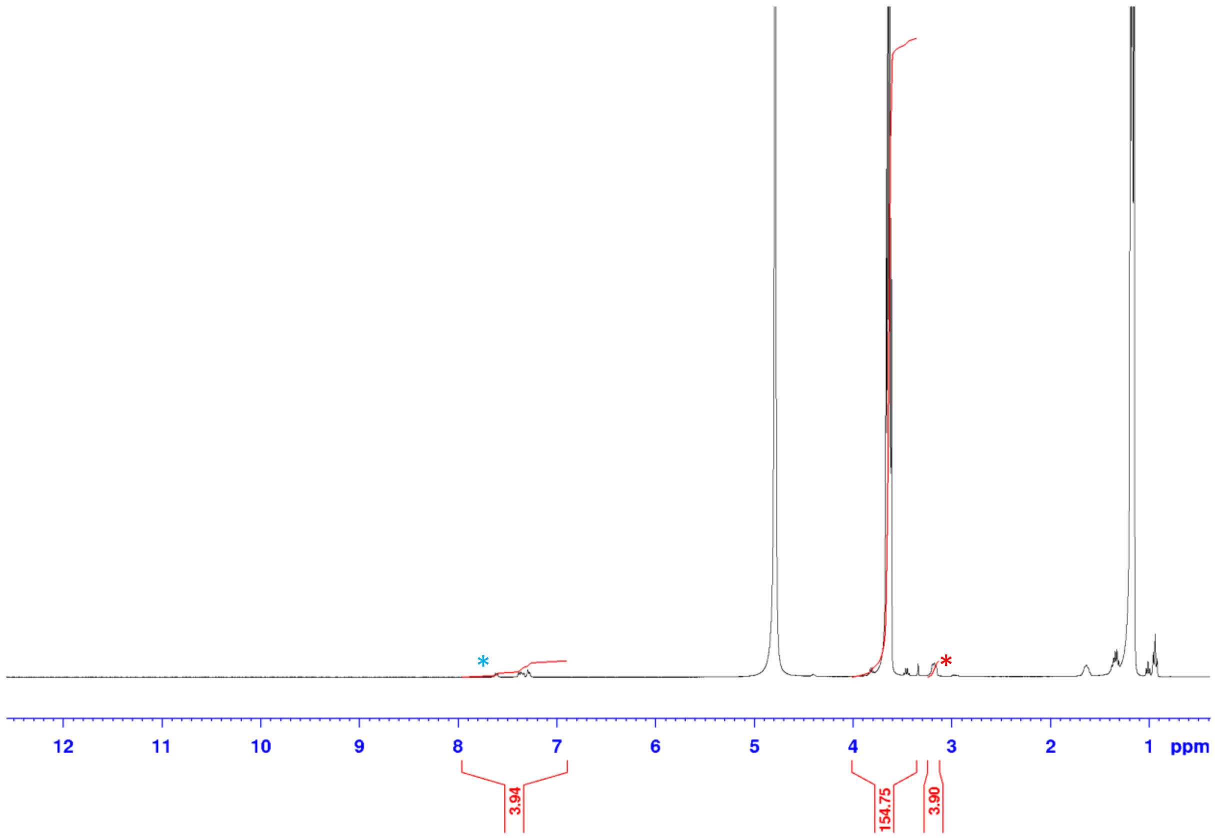

Figure S30 - ^{1}H NMR spectrum (d_1 = 60 s) of a 1:1 enantiomeric mixture of **7** + **9** (5.56 mM) in D_2O/ 5.0 % EtOH. Comparative integration indicates 56 % of the anionic component (blue) and 51 % of the cationic component (red) has become NMR silent.

Summary

Table S5 – Overview of results of the quantitative ^{1}H NMR (qNMR) studies for **3**, **5**,**7**, **9** and 1:1 enantiomeric mixtures of **3** + **5** and **7** + **9** performed at concentrations of ≈ 112 mM in DMSO-d_6/ 1.0 % DCM and 5.56 mM in D_2O/ 5.0 % EtOH. Values given in % represent the observed proportion of compound that has become NMR silent.

**Compound**	**DMSO-d_6/1.0 % DCM (%)**		**D_2O/5.0 % EtOH (%)**	
	**Anionic component**	**Cationic component**	**Anionic component**	**Cationic component**
**3**	0	0	50	44
**5**	0	0	52	48
**3** + **5** ^{a}	68	69	31	33
**7**	0	0	51	48
**9**	0	0	58	53
**7** + **9** ^{a}	82	78	56	51

a – 1:1 mixture.

Section S8: ^{1}H NMR DOSY studies

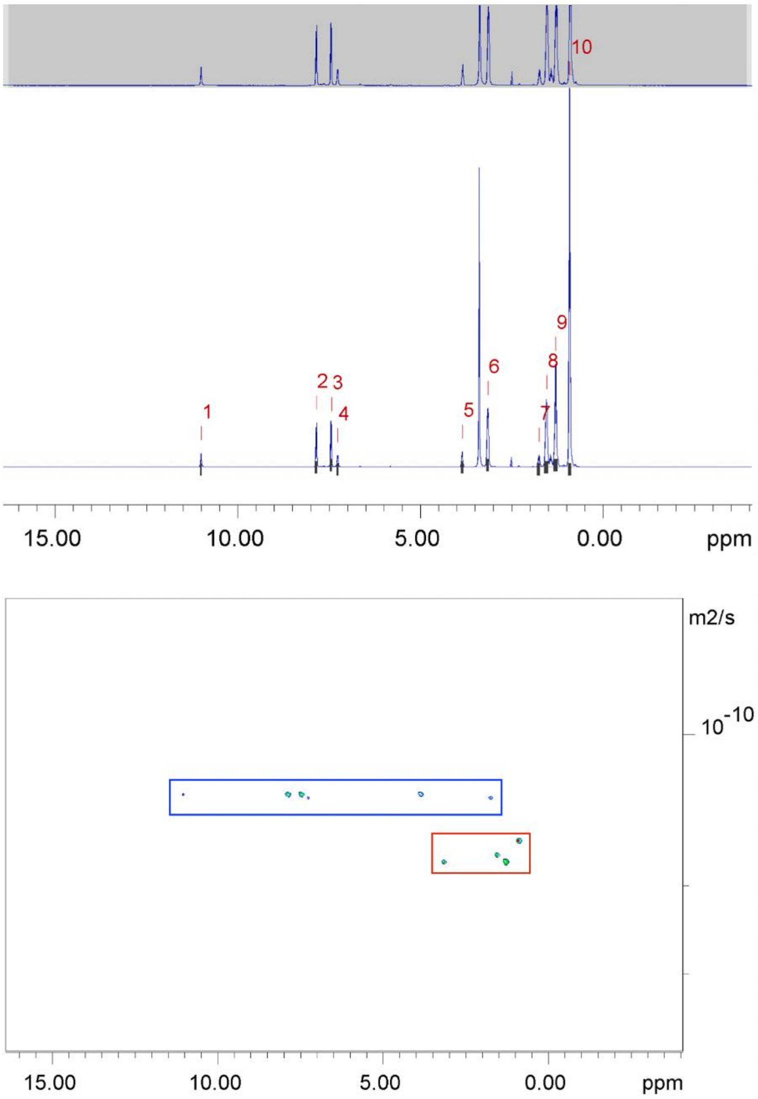

Peak name	F2 [ppm]	Io	error	D [m2/s]	error
1	11.002	1.46e+09	1.277e+05	1.31e-10	2.530e-14
2	7.850	5.20e+09	1.511e+05	1.32e-10	8.446e-15
3	7.449	5.18e+09	1.454e+05	1.31e-10	8.111e-15
4	7.268	1.81e+09	1.405e+05	1.34e-10	2.296e-14
5	3.854	2.37e+09	1.542e+05	1.31e-10	1.873e-14
6	3.154	1.25e+10	1.725e+05	1.81e-10	5.360e-15
7	1.761	2.23e+09	1.648e+05	1.33e-10	2.159e-14
8	1.552	1.70e+10	2.017e+05	1.73e-10	4.413e-15
9	1.300	2.01e+10	2.032e+05	1.80e-10	3.923e-15
10	0.915	4.64e+10	1.733e+05	1.63e-10	1.318e-15

Figure S31 - ^{1}H DOSY NMR of **3** (112 mM) in a DMSO-d_6/0.5 % H_2O solution conducted at 298 K and a table reporting the diffusion constants calculated for each peak used to determine the hydrodynamic diameter (d_H) of the anionic and cationic components. The anionic component is highlighted in blue and corresponds to peaks 1-5 and 7 (d_H = 1.66 nm), while the cationic component is highlighted in red and corresponds to peaks 6 and 8-10 (d_H = 1.26 nm).

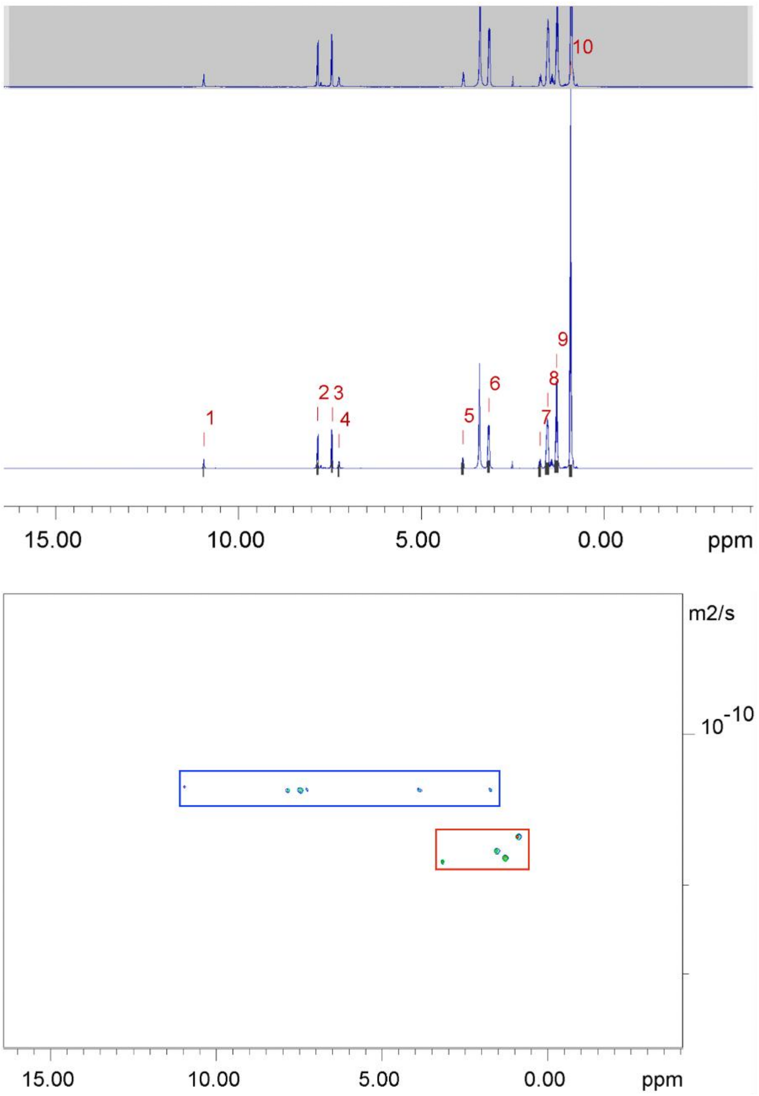

Peak name	F2 [ppm]	Io	error	D [m2/s]	error
1	10.952	6.35e+08	5.186e+04	1.28e-10	2.301e-14
2	7.838	2.45e+09	6.193e+04	1.29e-10	7.145e-15
3	7.448	2.56e+09	5.791e+04	1.28e-10	6.391e-15
4	7.255	7.27e+08	5.629e+04	1.29e-10	2.190e-14
5	3.865	1.24e+09	7.675e+04	1.29e-10	1.754e-14
6	3.159	6.81e+09	8.296e+04	1.78e-10	4.649e-15
7	1.757	1.20e+09	7.804e+04	1.29e-10	1.851e-14
8	1.552	9.23e+09	9.579e+04	1.70e-10	3.786e-15
9	1.301	1.08e+10	9.916e+04	1.77e-10	3.471e-15
10	0.915	2.49e+10	8.302e+04	1.61e-10	1.158e-15

Figure S32 - ^{1}H DOSY NMR of **5** (112 mM) in a DMSO-d_6/0.5 % H_2O solution conducted at 298 K and a table reporting the diffusion constants calculated for each peak used to determine the hydrodynamic diameter (d_H) of the anionic component. The anionic component is highlighted in blue and corresponds to peaks 1-5 and 7 (d_H = 1.70 nm), while the cationic component is highlighted in red and corresponds to peaks 6 and 8-10 (d_H = 1.28 nm).

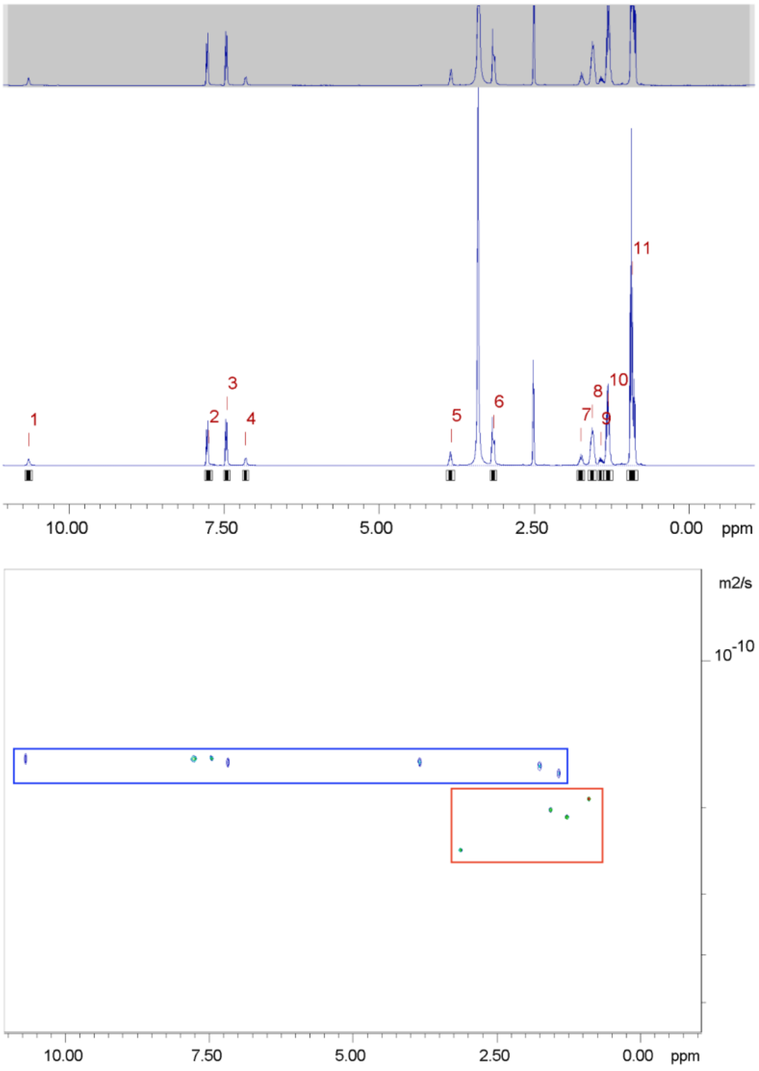

Peak name	F2 [ppm]	Io	error	D [m2/s]	error
1	10.644	1.47e+08	5.563e+04	1.58e-10	1.285e-13
2	7.748	6.63e+08	5.668e+04	1.59e-10	2.921e-14
3	7.452	6.84e+08	5.252e+04	1.59e-10	2.621e-14
4	7.150	1.72e+08	4.986e+04	1.62e-10	1.008e-13
5	3.838	3.05e+08	5.975e+04	1.60e-10	6.752e-14
6	3.150	9.00e+08	5.944e+04	2.44e-10	3.369e-14
7	1.738	3.10e+08	5.675e+04	1.63e-10	6.397e-14
8	1.553	1.31e+09	6.435e+04	2.01e-10	2.086e-14
9	1.414	2.17e+08	4.964e+04	1.69e-10	8.258e-14
10	1.293	2.04e+09	6.611e+04	2.09e-10	1.424e-14
11	0.908	5.22e+09	7.047e+04	1.93e-10	5.500e-15

Figure S33 - ^{1}H DOSY NMR of **3** + **5** (112 mM) in a DMSO-d_6/0.5 % H_2O solution conducted at 298 K and a table reporting the diffusion constants calculated for each peak used to determine the hydrodynamic diameter (d_H) of the anionic component. The anionic component is highlighted in blue and corresponds to peaks 1-5,7 and 9 (d_H = 1.36 nm), while the cationic component is highlighted in red and corresponds to peaks 6, 8, 10 and 11 (d_H = 1.04 nm).

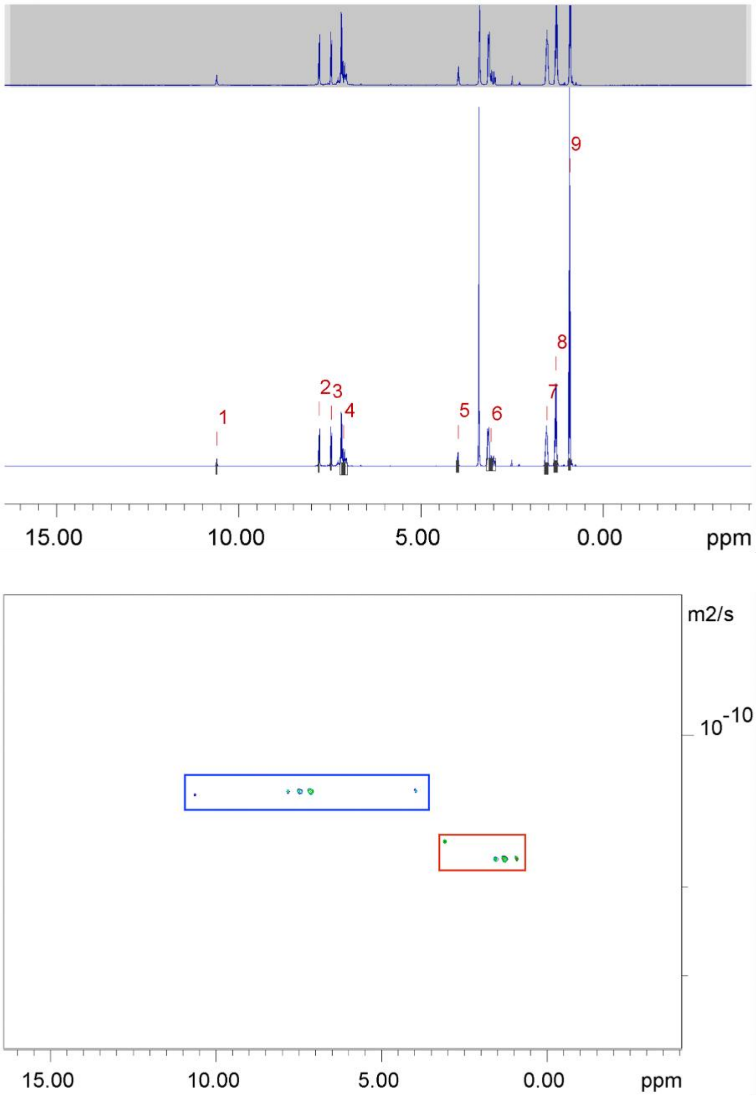

Peak name	F2 [ppm]	Io	error	D [m2/s]	error
1	10.610	7.03e+08	5.841e+04	1.30e-10	2.408e-14
2	7.797	2.75e+09	5.991e+04	1.30e-10	6.279e-15
3	7.468	2.79e+09	5.845e+04	1.29e-10	6.016e-15
4	7.124	8.56e+09	1.197e+05	1.29e-10	4.018e-15
5	3.988	1.34e+09	7.616e+04	1.29e-10	1.627e-14
6	3.078	9.81e+09	1.471e+05	1.62e-10	5.304e-15
7	1.553	8.47e+09	9.874e+04	1.75e-10	4.419e-15
8	1.301	1.14e+10	9.922e+04	1.77e-10	3.329e-15
9	0.923	1.84e+10	8.030e+04	1.75e-10	1.654e-15

Figure S34 – ^{1}H DOSY NMR of **7** (112 mM) in a DMSO-d_6/0.5 % H_2O solution conducted at 298 K and a table reporting the diffusion constants calculated for each peak used to determine the hydrodynamic diameter (d_H) of the anionic component. The anionic component is highlighted in blue and corresponds to peaks 1-5 (d_H = 1.70 nm), while the cationic component is highlighted in red and corresponds to peaks 6-9 (d_H = 1.27 nm).

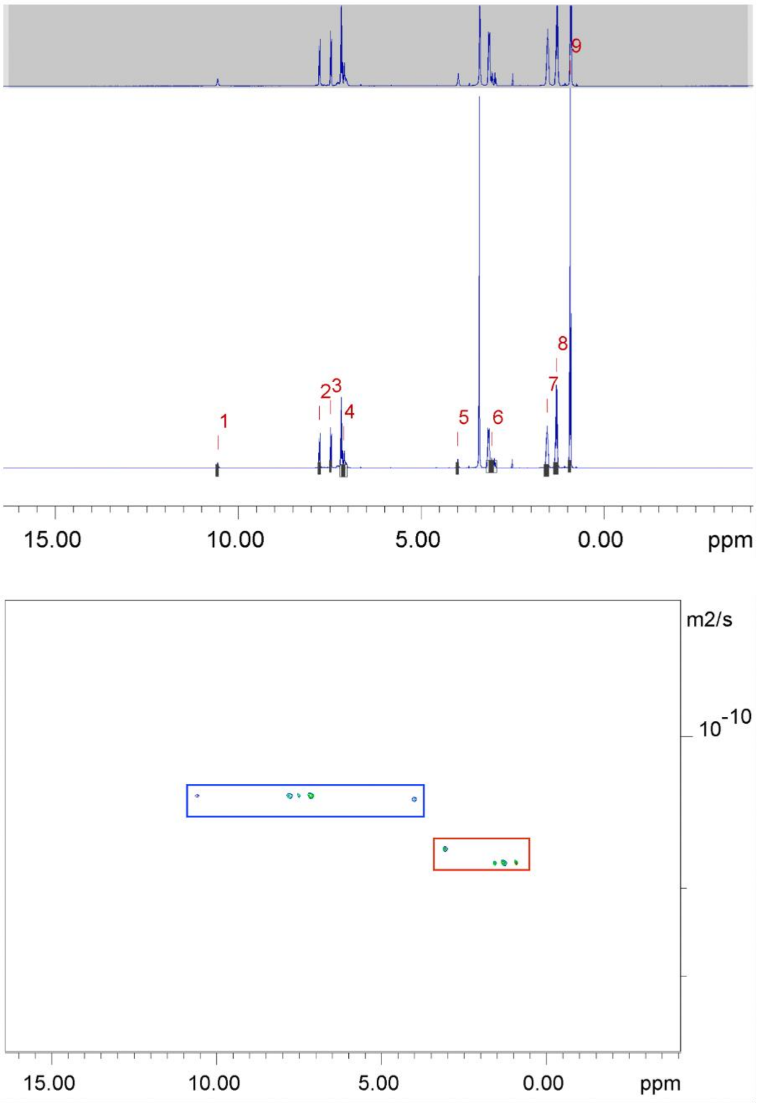

Peak name	F2 [ppm]	Io	error	D [m2/s]	error
1	10.568	6.77e+08	8.274e+04	1.31e-10	3.527e-14
2	7.780	2.67e+09	8.020e+04	1.32e-10	8.744e-15
3	7.479	2.70e+09	7.400e+04	1.31e-10	7.933e-15
4	7.126	7.56e+09	1.245e+05	1.31e-10	4.755e-15
5	4.003	1.17e+09	8.845e+04	1.33e-10	2.222e-14
6	3.077	8.99e+09	1.602e+05	1.68e-10	6.432e-15
7	1.566	7.88e+09	1.167e+05	1.79e-10	5.671e-15
8	1.308	1.03e+10	1.190e+05	1.79e-10	4.421e-15
9	0.935	1.68e+10	9.043e+04	1.79e-10	2.059e-15

Figure S35 – ^{1}H DOSY NMR of **9** (112 mM) in a DMSO-d_6/0.5 % H_2O solution conducted at 298 K and a table reporting the diffusion constants calculated for each peak used to determine the hydrodynamic diameter (d_H) of the anionic component. The anionic component is highlighted in blue and corresponds to peaks 1-5 (d_H = 1.67 nm), while the cationic component is highlighted in red and corresponds to peaks 6-9 (d_H = 1.24 nm).

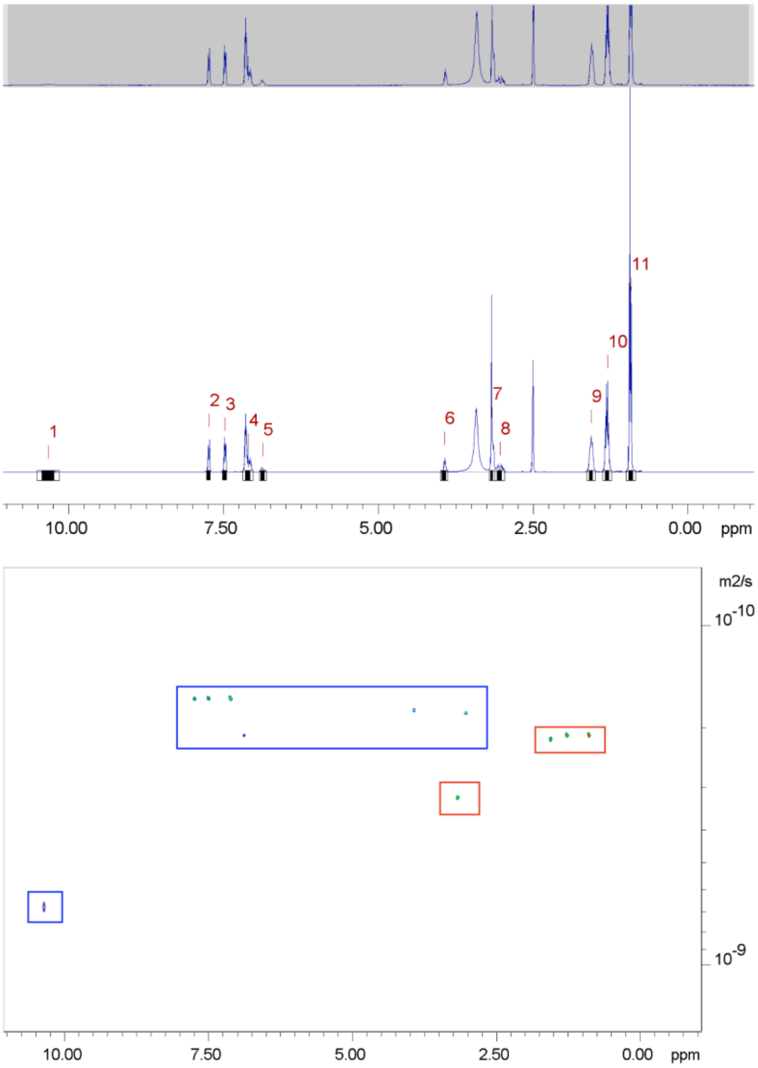

Peak name	F2 [ppm]	Io	error	D [m2/s]	error
1	10.330	5.77e+07	1.286e+05	6.68e-10	2.697e-12
2	7.741	2.74e+08	3.560e+04	1.66e-10	4.097e-14
3	7.480	2.79e+08	3.709e+04	1.66e-10	4.199e-14
4	7.104	7.02e+08	5.344e+04	1.66e-10	2.405e-14
5	6.867	7.07e+07	4.792e+04	2.12e-10	2.670e-13
6	3.941	1.41e+08	4.736e+04	1.75e-10	1.111e-13
7	3.168	7.78e+08	5.117e+04	3.19e-10	3.811e-14
8	3.041	1.91e+08	5.559e+04	1.83e-10	9.988e-14
9	1.563	6.32e+08	5.467e+04	2.13e-10	3.420e-14
10	1.296	1.09e+09	5.576e+04	2.13e-10	2.014e-14
11	0.917	1.99e+09	5.793e+04	2.13e-10	1.149e-14

Figure S36 – ^{1}H DOSY NMR of a 1:1 enantiomeric mixture of **7** + **9** (112 mM) in a DMSO-*d*$_6$/0.5 % H_2O solution conducted at 298 K and a table reporting the diffusion constants calculated for each peak used to determine the hydrodynamic diameter (d_H) of the anionic component. The anionic component is highlighted in blue and corresponds to peaks 1-6 and 8 (d_H = 0.88 nm), while the cationic component is highlighted in red and corresponds to peaks 7 and 9-11 (d_H = 0.92 nm).

Summary

Table S6 - Overview of diffusion coefficients (m^2s^{-1}) of **3**, **5**,**7**, **9**, and 1:1 enantiomeric mixtures of **3** + **5** and **7** + **9** in a DMSO-d_6/0.5 % H_2O solution at 298 K. Errors for diffusion constants are no greater than ± 1 x 10^{-13} m^2s^{-1}.

	**Diffusion coefficient (m^2s^{-1})**	
**Compound**	**Anionic component**	**Cationic component**
**3**	1.32 x 10^{-10}	1.74 x 10^{-10}
**5**	1.29 x 10^{-10}	1.72 x 10^{-10}
**3 + 5** [a]	1.61 x 10^{-10}	2.12 x 10^{-10}
**7**	1.29 x 10^{-10}	1.72 x 10^{-10}
**9**	1.32 x 10^{-10}	1.76 x 10^{-10}
**7 + 9** [a]	2.48 x 10^{-10}	2.40 x 10^{-10}

a – 1:1 mixture.

Table S7 - Overview of hydrodynamic diameters (nm) for **3**, **5**,**7**, **9**, and 1:1 enantiomeric mixture of **3** + **5** and **7** + **9** in a DMSO-d_6/0.5 % H_2O solution at 298 K.

	**Hydrodynamic diameter (nm)**	
**Compound**	**Anionic component**	**Cationic component**
**1**	1.26-1.29[5]	*b*
**3**	1.66	1.26
**5**	1.70	1.28
**3 + 5** [a]	1.36	1.04
**7**	1.70	1.27
**9**	1.67	1.24
**7 + 9** [a]	0.88	0.92

a – 1:1 mixture.

b – Data not available.

Section S9: ^{1}H NMR self-association data

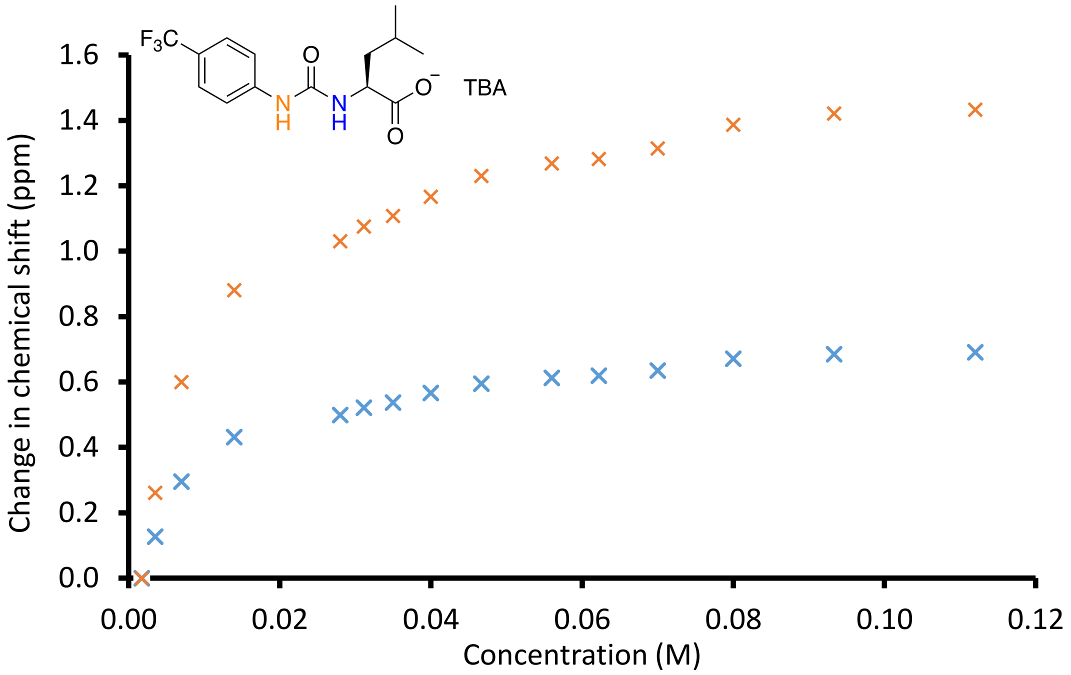

Figure S37 – Graph illustrating the ^{1}H NMR down-field change in chemical shift of urea NH resonances with increasing concentration of **3** in DMSO-d_6/0.5 % H_2O (298 K).

Self-association constant calculation: values were calculated from data gathered from both NH's and fitted to the Cooperative Equal K (CoEK) and Equal K/ dimerization binding models.

Equal K/Dimerization model (Nelder-Mead fit):

K_e = 242.72 M^{-1} ± 3.80 % K_{dim} = 121.36 M^{-1} ± 1.90 %
http://app.supramolecular.org/bindfit/view/51ce652b-7b2b-4974-8812-79d1ae73fc4f

CoEK model (Nelder-Mead fit):

K_e = 560.90 M^{-1} ± 5.21 % K_{dim} = 280.45 M^{-1} ± 2.61 % ρ = 2.29 ± 9.25 %
http://app.supramolecular.org/bindfit/view/c48548c8-ee64-4855-8686-7c76c927c103

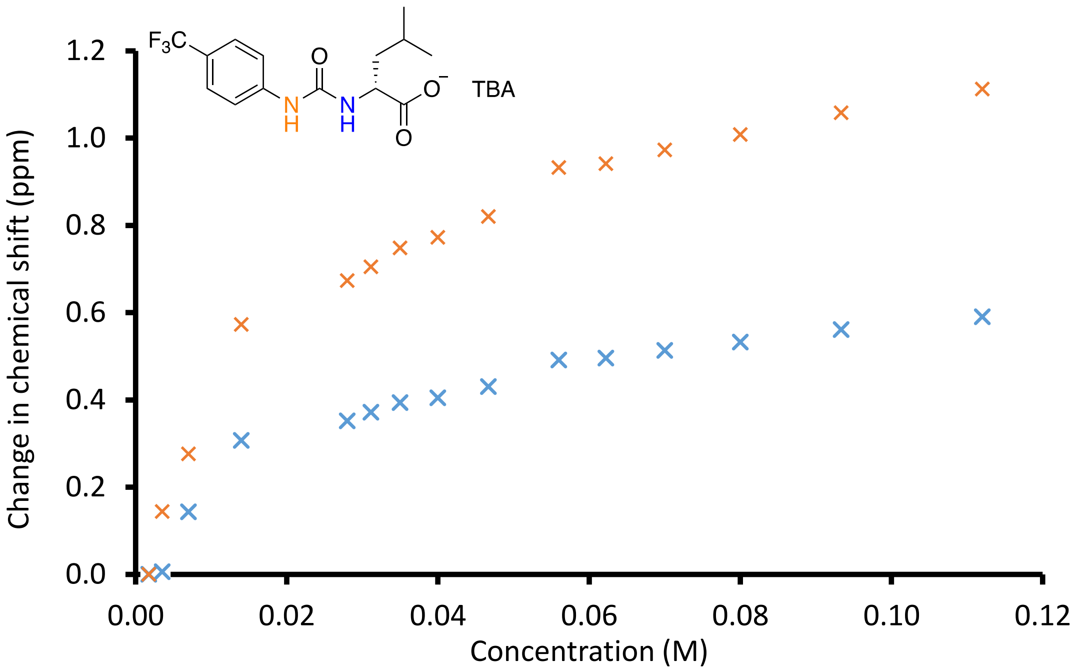

Figure S38 – Graph illustrating the ^{1}H NMR down-field change in chemical shift of urea NH resonances with increasing concentration of **5** in DMSO-d_6/0.5 % H_2O (298 K).

Self-association constant calculation: values were calculated from data gathered from both NH's and fitted to the Cooperative Equal K (CoEK) and Equal K/ dimerization binding models.

Equal K/Dimerization model:

K_e = 180.55 M^{-1} ± 6.48 % K_{dim} = 90.27 M^{-1} ± 3.24 %
http://app.supramolecular.org/bindfit/view/b8b3ad35-c95a-4b43-ab01-9424006b8cf2

CoEK model:

K_e = 351.80 M^{-1} ± 7.18 % K_{dim} = 175.90 M^{-1} ± 3.59 % ρ = 3.41 ± 12.69 %
http://app.supramolecular.org/bindfit/view/3c8e5c40-cd1e-4e18-9c93-530cc3f7c71c

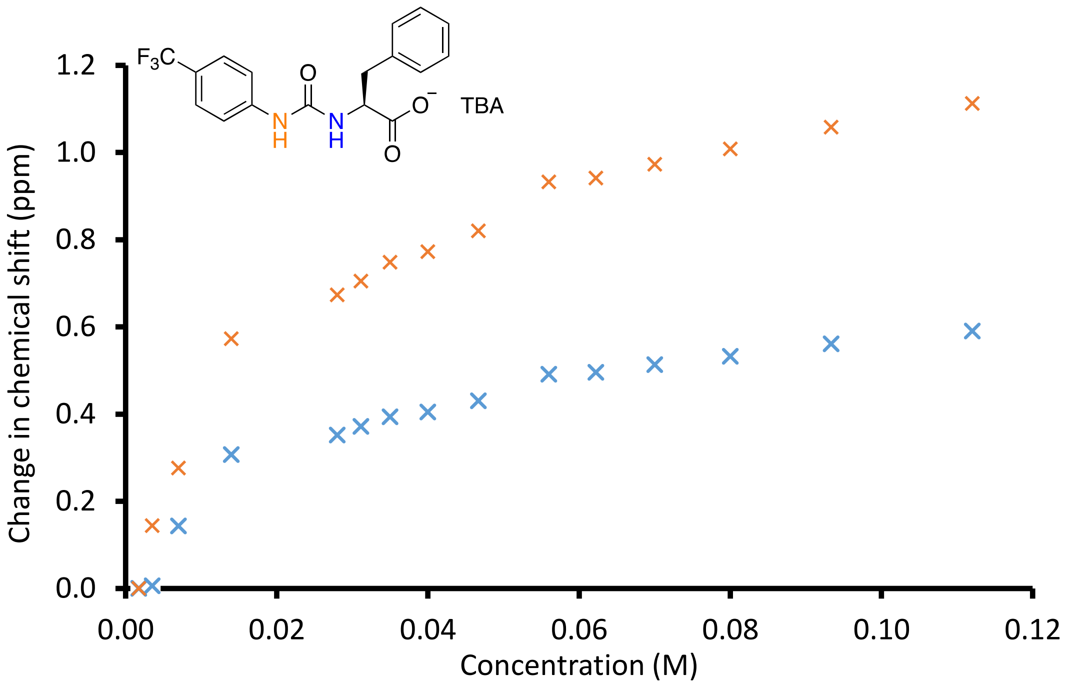

Figure S39 – Graph illustrating the ^{1}H NMR down-field change in chemical shift of urea NH resonances with increasing concentration of **7** in DMSO-d_6/0.5 % H_2O (298 K).

Self-association constant calculation: values were calculated from data gathered from both NH's and fitted to the Cooperative Equal K (CoEK) and Equal K/ dimerization binding models.

Equal K/Dimerization model:

K_e = 55.52 M^{-1} ± 4.24 % K_{dim} = 27.76 M^{-1} ± 2.12 %
http://app.supramolecular.org/bindfit/view/3bb6b512-7ffd-4fb2-b22c-3423a512b003

CoEK model:

K_e = 21.17 M^{-1} ± 6.11 % K_{dim} = 10.58 M^{-1} ± 3.06 % ρ = 2.86 ± 14.59 %
http://app.supramolecular.org/bindfit/view/0d92b6d7-4c95-488c-bc2d-3fe43e1e4cb7

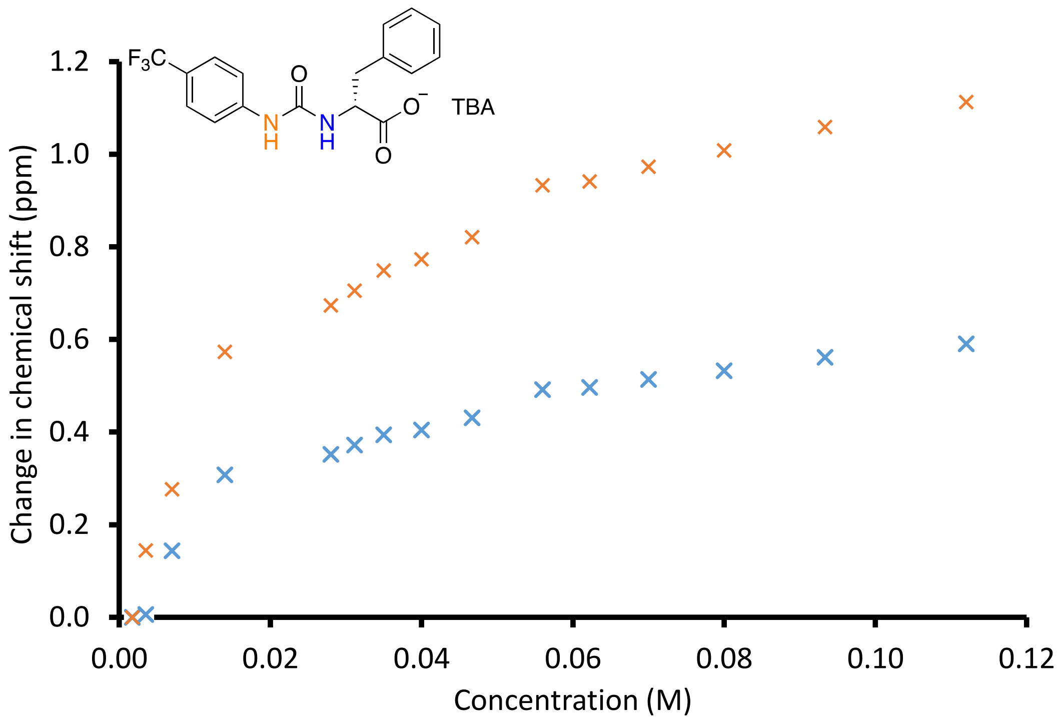

Figure S40 – Graph illustrating the ^{1}H NMR down-field change in chemical shift of urea NH resonances with increasing concentration of **9** in DMSO-d_6/0.5 % H_2O (298 K).

Self-association constant calculation: values were calculated from data gathered from both NH's and fitted to the Cooperative Equal K (CoEK) and Equal K/ dimerization binding models.

Equal K/Dimerization model:

K_e = 45.90 M^{-1} ± 5.03 % K_{dim} = 22.95 M^{-1} ± 2.52 %
http://app.supramolecular.org/bindfit/view/3abcecc0-ffc9-4e4e-917f-14958fbcf026

CoEK model:

K_e = 17.84 M^{-1} ± 8.52 % K_{dim} = 8.92 M^{-1} ± 4.26 % ρ = 2.62 ± 19.62 %
http://app.supramolecular.org/bindfit/view/91f0f17f-4882-4fec-bd09-cfce15a99d6b

Summary

Table S8- The self-association constants (K_e and K_{dim}) calculated for **3**, **5**, **7** and **9** in DMSO-d_6/ 0.5 % H_2O solution at 298 K. Constants were obtained for the EK and CoEK models by fitting ^{1}H NMR self-association data to Bindfit v0.5.

Compound	EK model (M^{-1})				CoEK model (M^{-1})					
	K_e	Error (± %)	K_{dim}	Error (± %)	K_e	Error (± %)	K_{dim}	Error (± %)	ρ	Error (± %)
**3**	242.72	3.80	121.36	1.90	560.90	5.21	280.45	2.61	2.29	9.25
**5**	180.55	6.48	90.27	3.24	351.80	7.18	175.90	3.59	3.41	12.69
**7**	55.52	4.24	27.76	2.12	21.17	6.11	10.58	3.06	2.86	14.59
**9**	45.90	5.03	22.95	2.52	17.84	8.52	8.92	4.26	2.62	19.62

Section S10: Dynamic Light Scattering (DLS) studies

DLS studies in H_2O/5 % EtOH

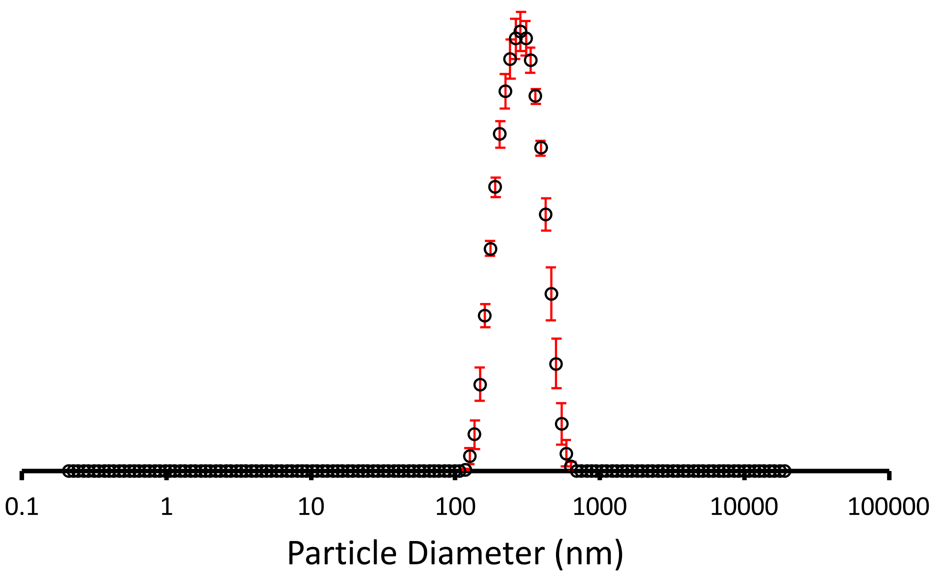

Figure S41 – The average intensity particle size distribution calculated using 10 DLS runs of **3** (5.56 mM) in H_2O/5 % EtOH at 298 K (1st repeat), with a peak maximum of 290.88 nm.

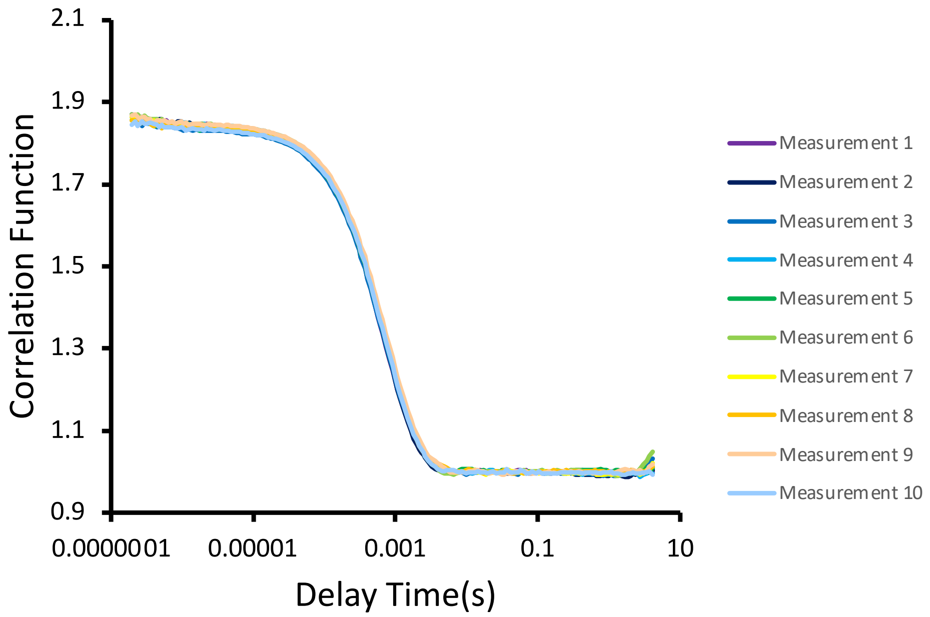

Figure S42 – The correlation function data for 10 DLS runs of **3** (5.56 mM) in H_2O/5 % EtOH at 298 K (1st repeat).

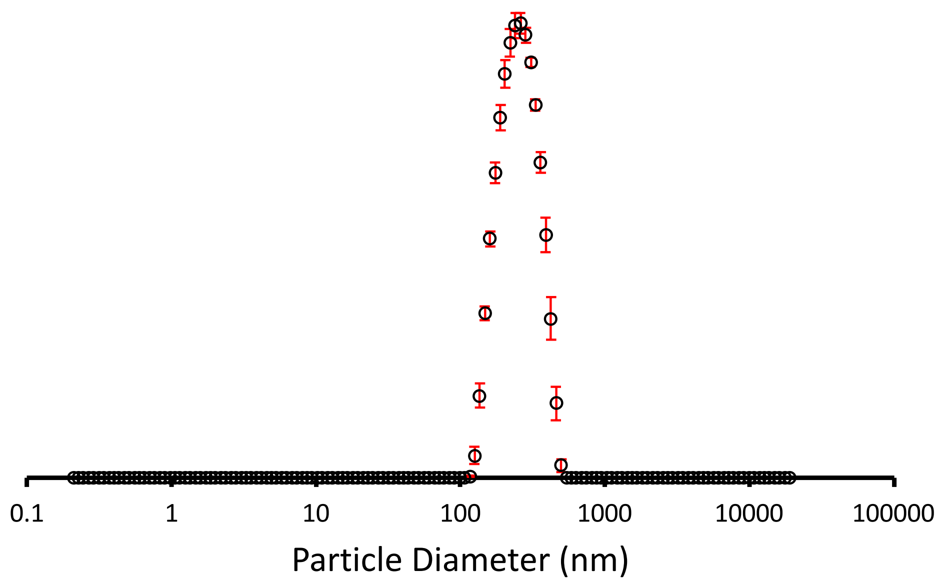

Figure S43 – The average intensity particle size distribution calculated using 10 DLS runs of **3** (5.56 mM) in H_2O/5 % EtOH at 298 K (2nd repeat), with a peak maximum of 262.00 nm.

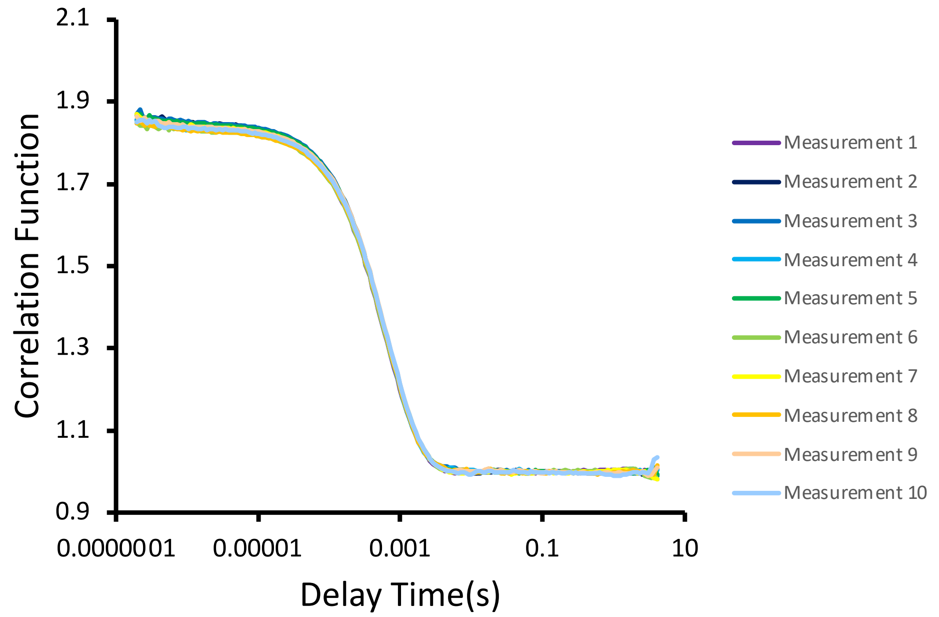

Figure S44 – The correlation function data for 10 DLS runs of **3** (5.56 mM) in H_2O/5 % EtOH at 298 K (2nd repeat).

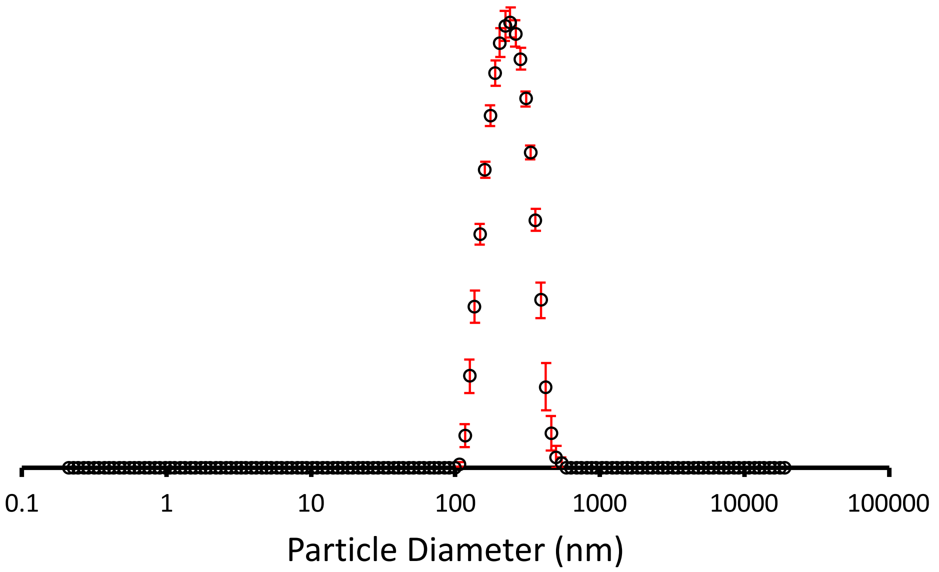

Figure S45 – The average intensity particle size distribution calculated using 10 DLS runs of **3** (5.56 mM) in H_2O/5 % EtOH at 298 K (3rd repeat), with a peak maximum of 244.06 nm.

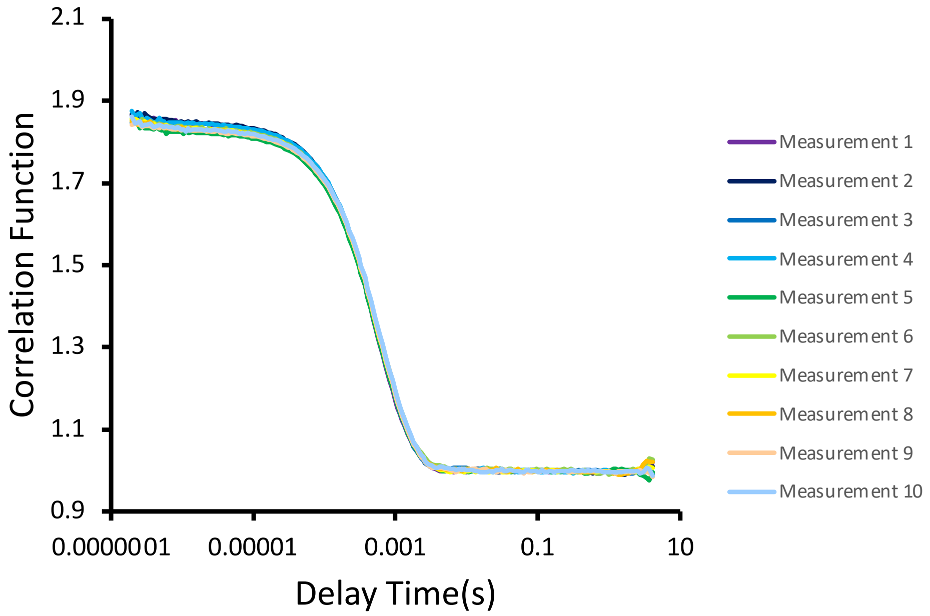

Figure S46 – The correlation function data for 10 DLS runs of **3** (5.56 mM) in H_2O/5 % EtOH at 298 K (3rd repeat).

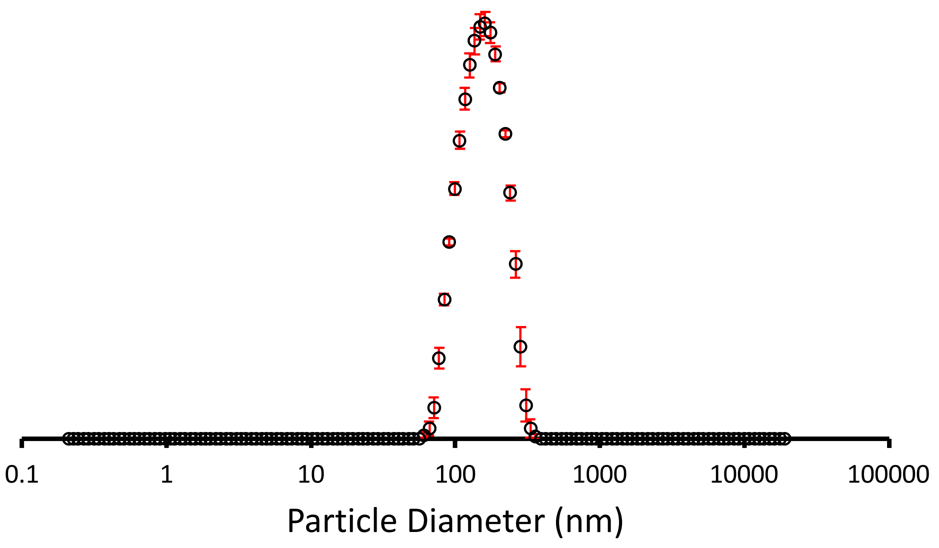

Figure S47 – The average intensity particle size distribution calculated using 10 DLS runs of **5** (5.56 mM) in H_2O/5 % EtOH at 298 K (1st repeat), with a peak maximum of 159.93 nm.

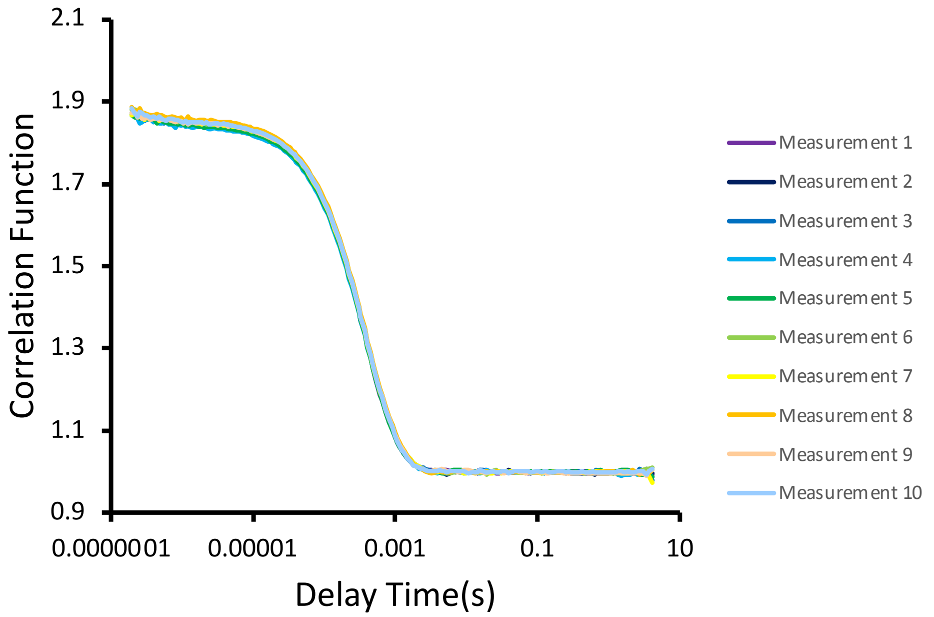

Figure S48 – The correlation function data for 10 DLS runs of **5** (5.56 mM) in H_2O/5 % EtOH at 298 K (1st repeat).

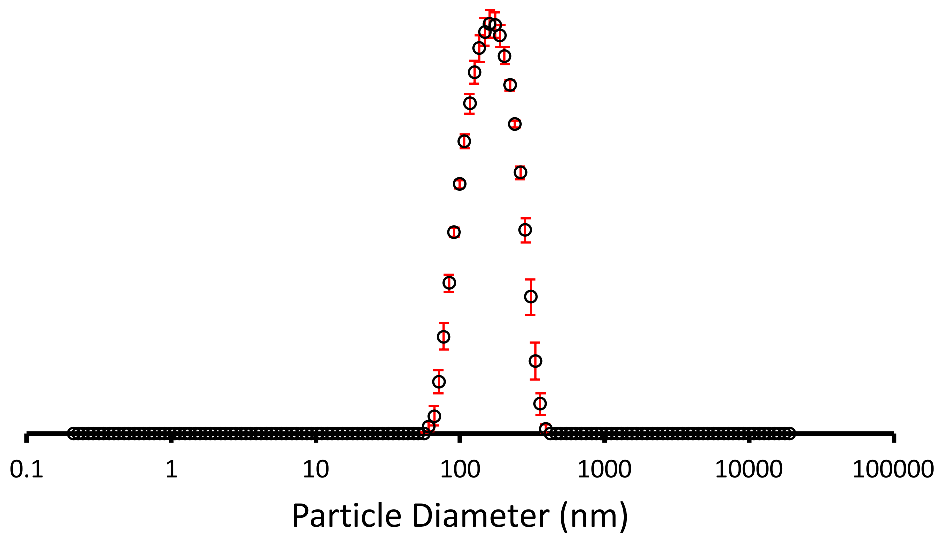

Figure S49 – The average intensity particle size distribution calculated using 10 DLS runs of **5** (5.56 mM) in H_2O/5 % EtOH at 298 K (2nd repeat), with a peak maximum of 170.91 nm.

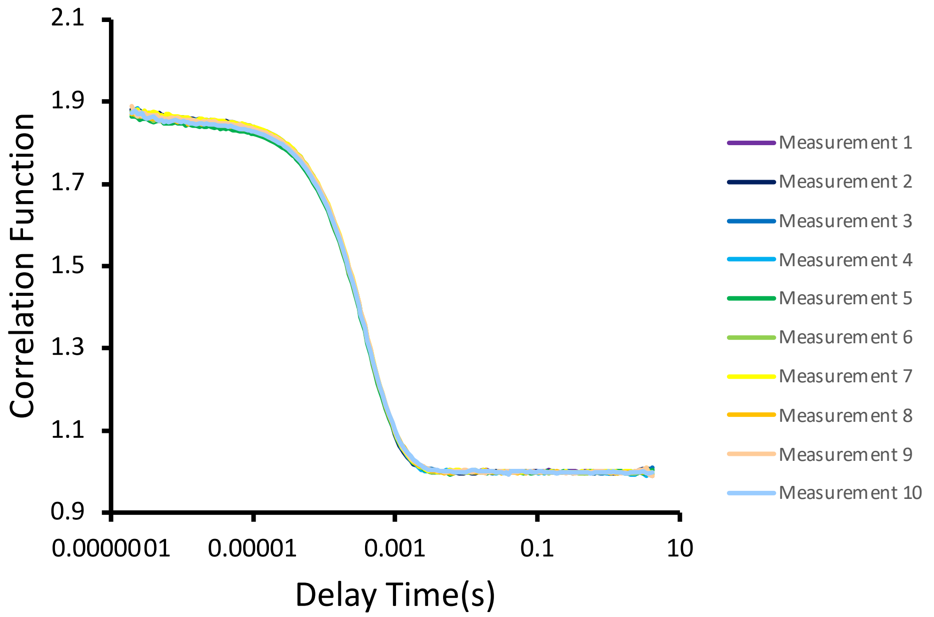

Figure S50 – The correlation function data for 10 DLS runs of **5** (5.56 mM) in H_2O/5 % EtOH at 298 K (2nd repeat).

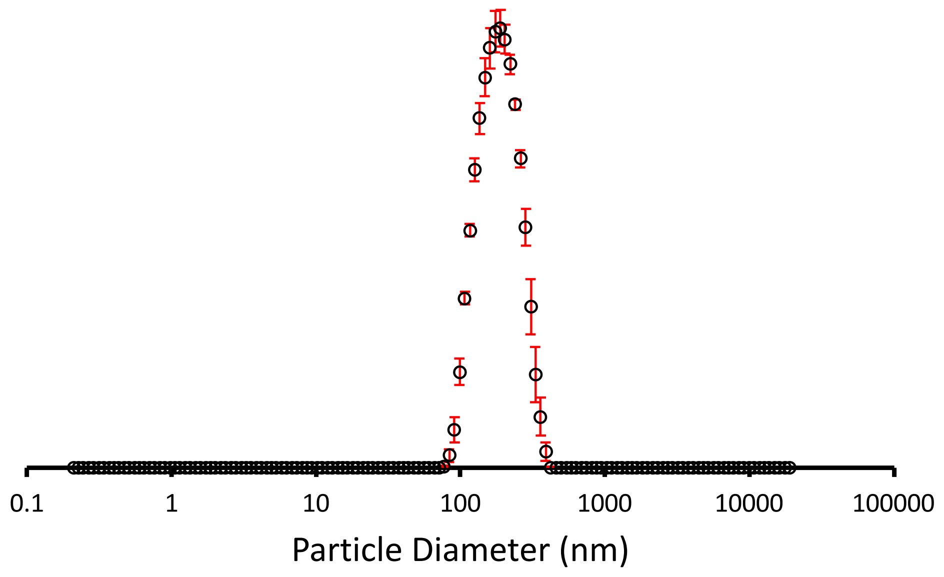

Figure S51 – The average intensity particle size distribution calculated using 10 DLS runs of **5** (5.56 mM) in H_2O/5 % EtOH at 298 K (3rd repeat), with a peak maximum of 191.31 nm.

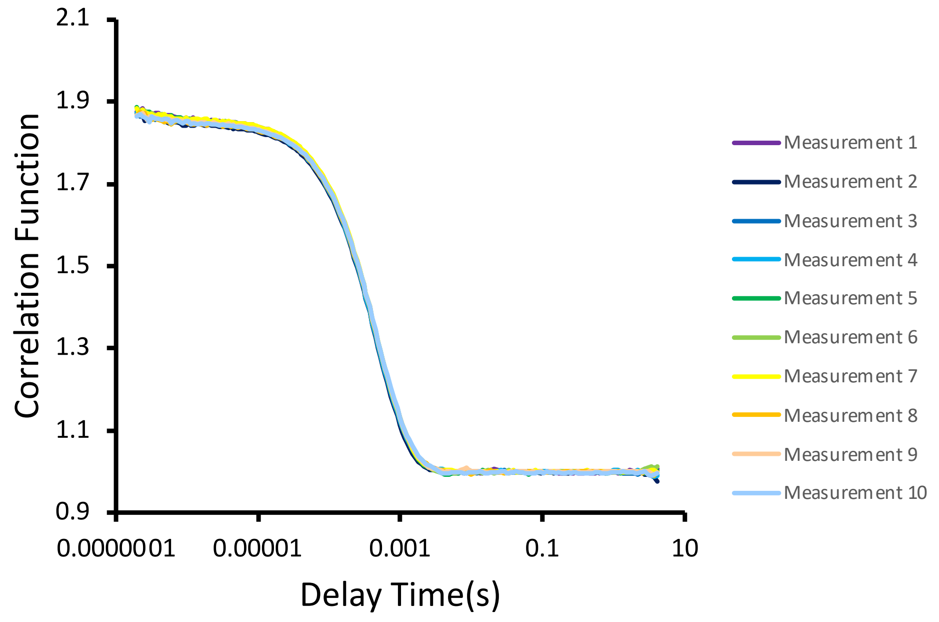

Figure S52 – The correlation function data for 10 DLS runs of **5** (5.56 mM) in H_2O/5 % EtOH at 298 K (3rd repeat).

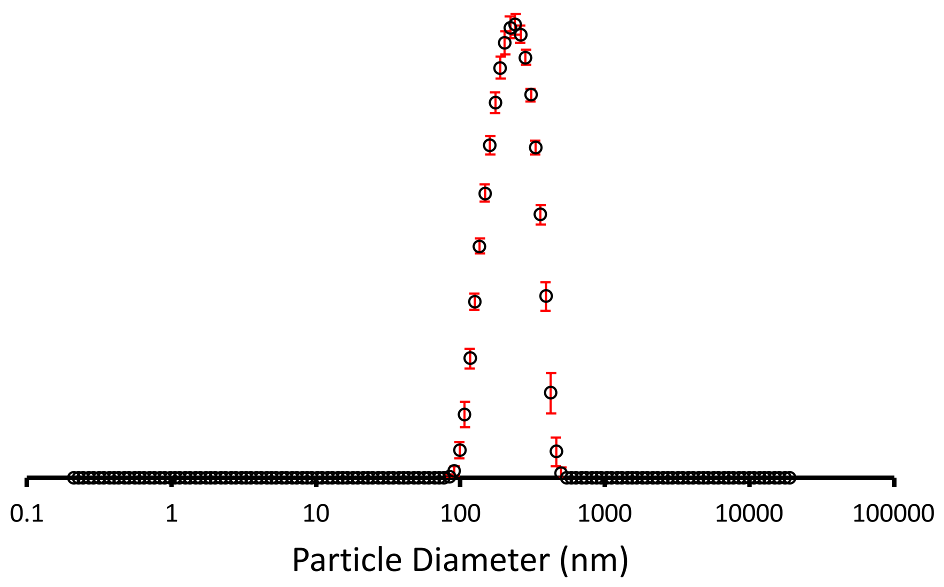

Figure S53 – The average intensity particle size distribution calculated using 10 DLS runs of a 1:1 enantiomeric mixture of **3** + **5** (5.56 mM) in H_2O/5 % EtOH at 298 K (1st repeat), with a peak maximum of 234.81 nm.

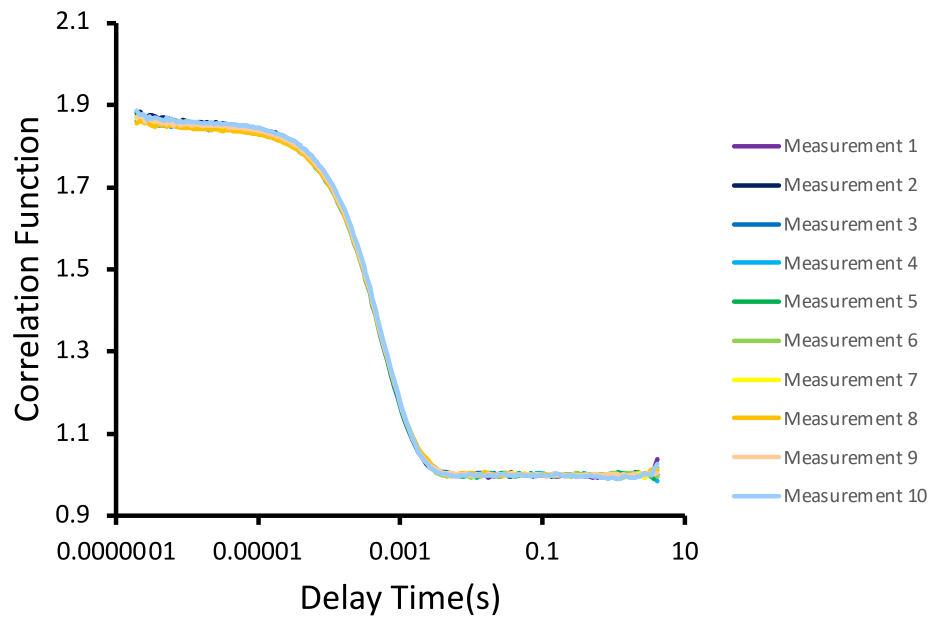

Figure S54 – The correlation function data for 10 DLS runs of a 1:1 enantiomeric mixture of **3** + **5** (5.56 mM) in H_2O/5 % EtOH at 298 K (1st repeat).

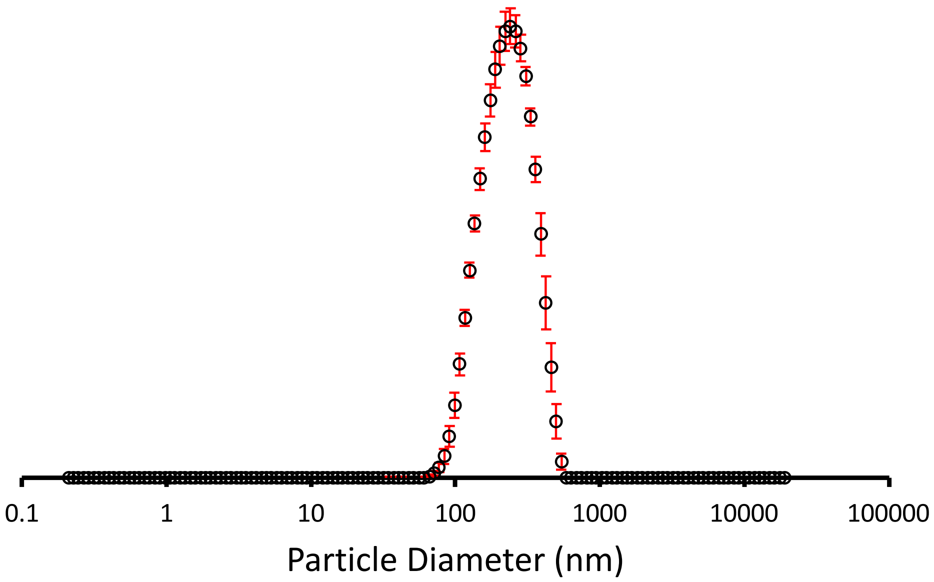

Figure S55 – The average intensity particle size distribution calculated using 10 DLS runs of a 1:1 enantiomeric mixture of **3** + **5** (5.56 mM) in H_2O/5 % EtOH at 298 K (2nd repeat), with a peak maximum of 241.14 nm.

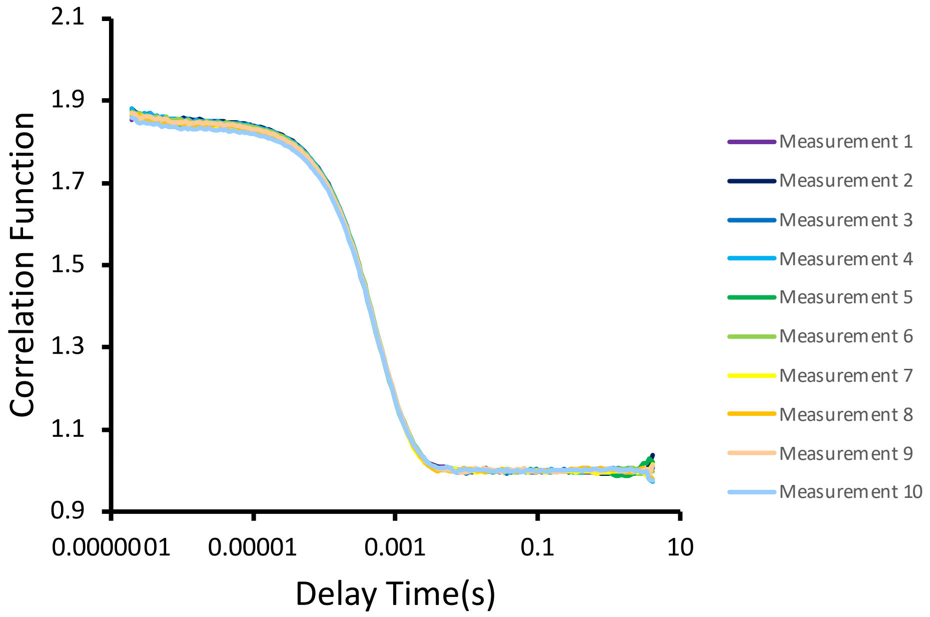

Figure S56 – The correlation function data for 10 DLS runs of a 1:1 enantiomeric mixture of **3** + **5** (5.56 mM) in H_2O/5 % EtOH at 298 K (2nd repeat).

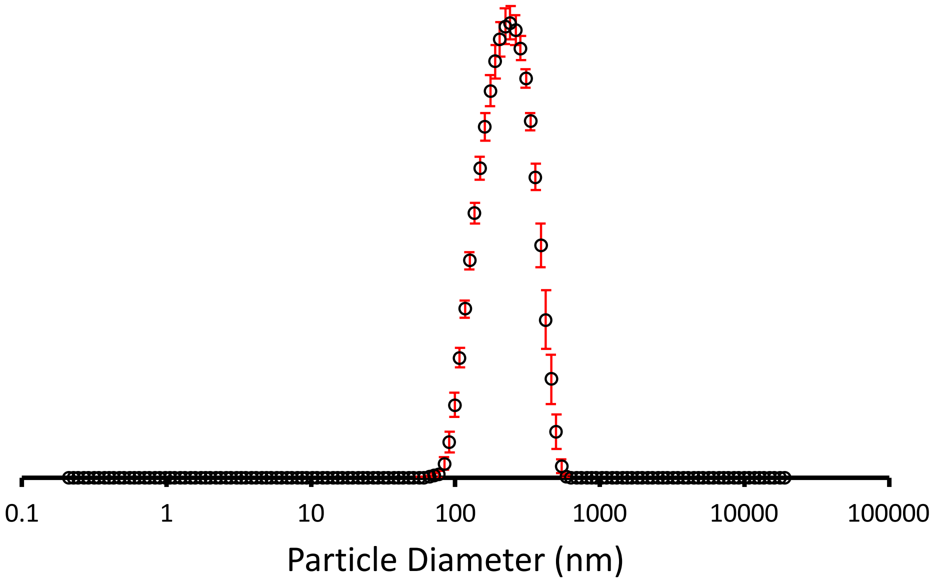

Figure S57 – The average intensity particle size distribution calculated using 10 DLS runs of a 1:1 enantiomeric mixture of **3** + **5** (5.56 mM) in H_2O/5 % EtOH at 298 K (3rd repeat), with a peak maximum of 238.58 nm.

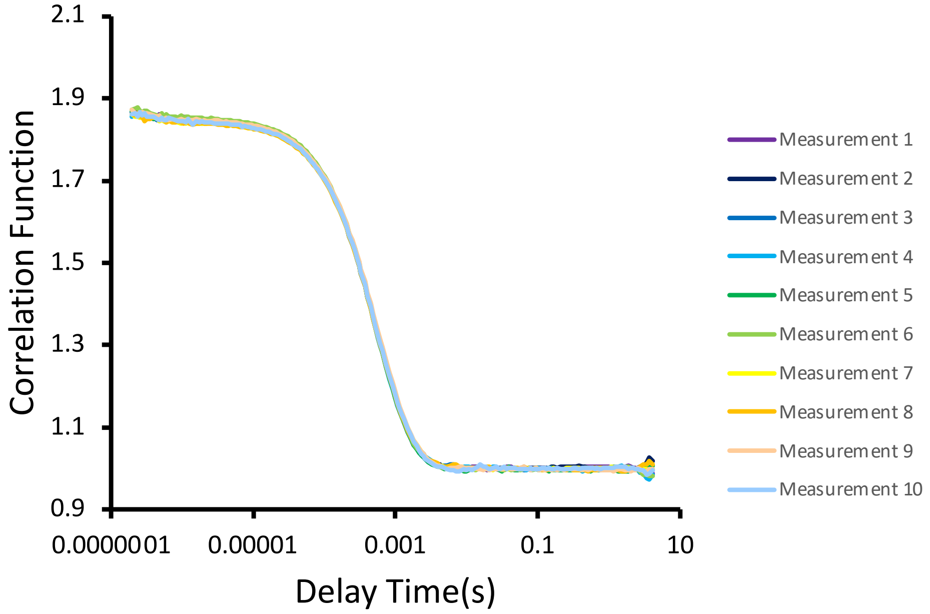

Figure S58 – The correlation function data for 10 DLS runs of a 1:1 enantiomeric mixture of **3** + **5** (5.56 mM) in H_2O/5 % EtOH at 298 K (3rd repeat).

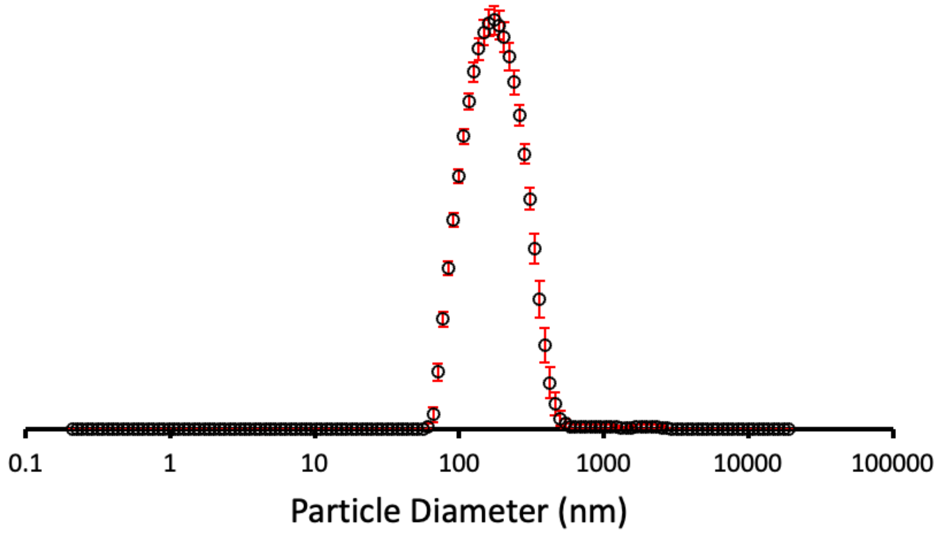

Figure S59 – The average intensity particle size distribution calculated using 10 DLS runs of **7** (5.56 mM) in H_2O/5 % EtOH at 298 K (1st repeat), with a peak maximum of 188.66 nm.

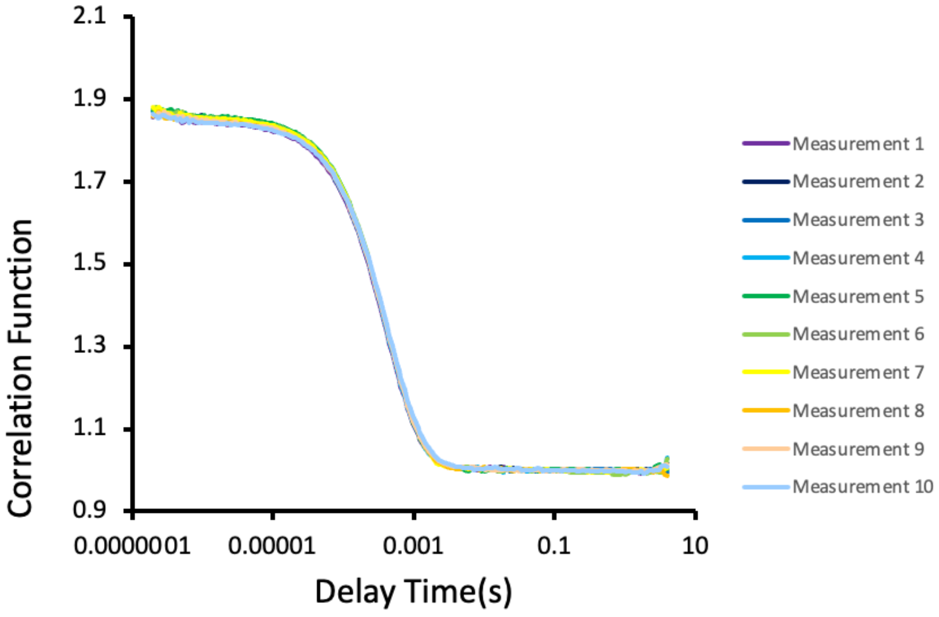

Figure S60 – The correlation function data for 10 DLS runs of **7** (5.56 mM) in H_2O/5 % EtOH at 298 K (1st repeat).

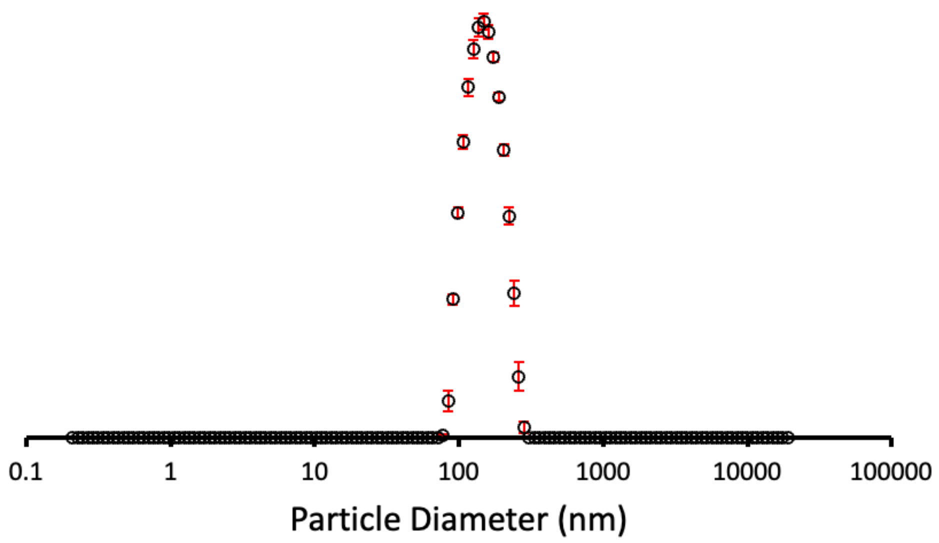

Figure S61 – The average intensity particle size distribution calculated using 10 DLS runs of **7** (5.56 mM) in H_2O/5 % EtOH at 298 K (2nd repeat), with a peak maximum of 154.17 nm.

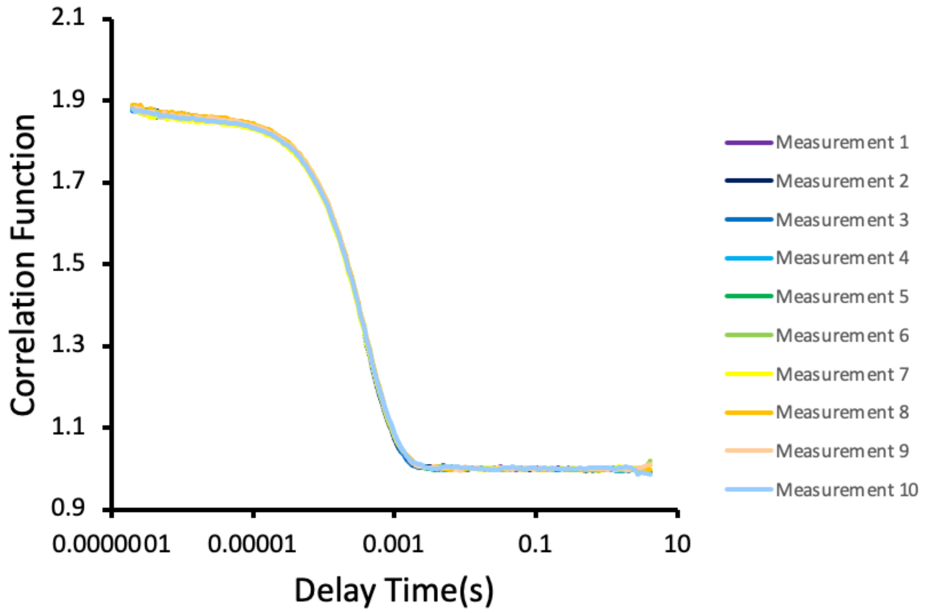

Figure S62 – The correlation function data for 10 DLS runs of **7** (5.56 mM) in H_2O/5 % EtOH at 298 K (2nd repeat).

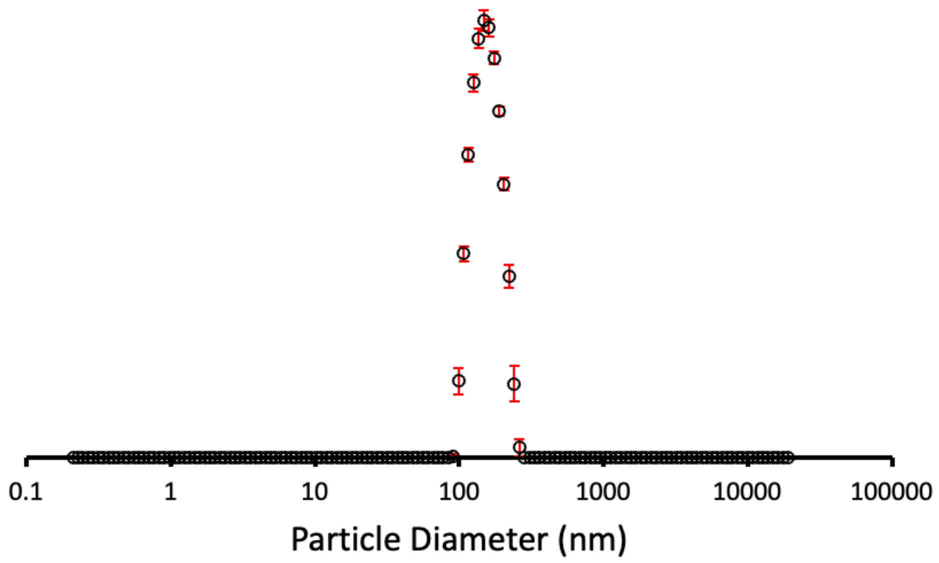

Figure S63 – The average intensity particle size distribution calculated using 10 DLS runs of **7** (5.56 mM) in H_2O/5 % EtOH at 298 K (3rd repeat), with a peak maximum of 157.03 nm.

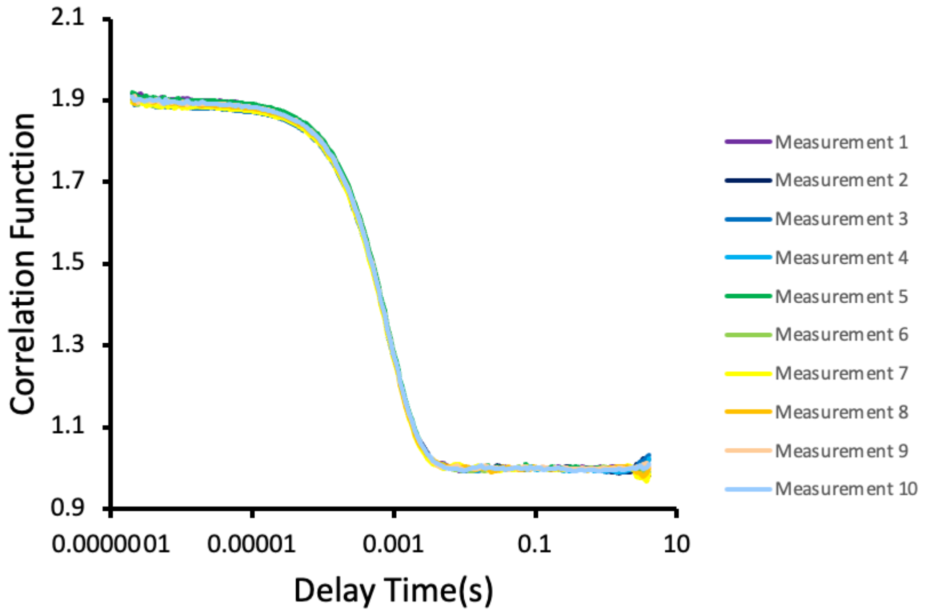

Figure S64 – The correlation function data for 10 DLS runs of **7** (5.56 mM) in H_2O/5 % EtOH at 298 K (3rd repeat).

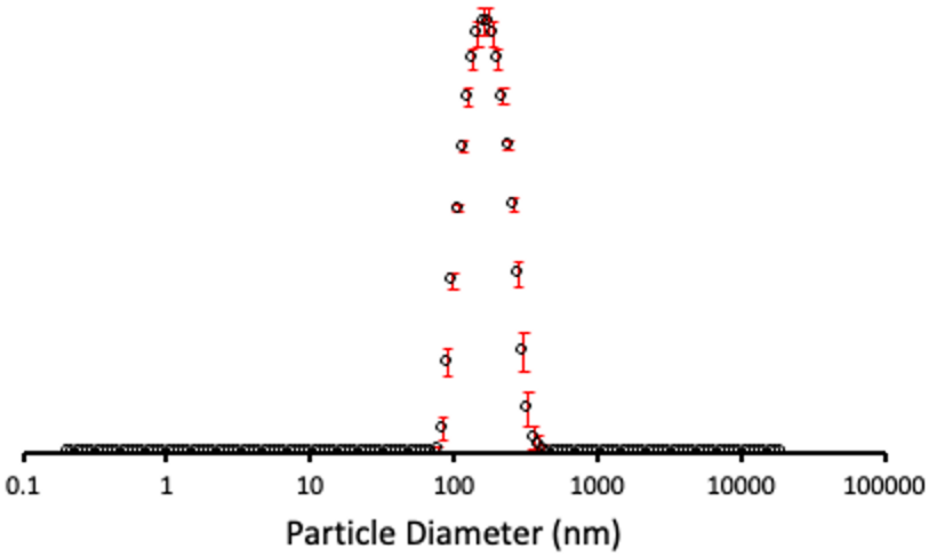

Figure S65 – The average intensity particle size distribution calculated using 10 DLS runs of **9** (5.56 mM) in H_2O/5 % EtOH at 298 K (1st repeat), with a peak maximum of 177.48 nm.

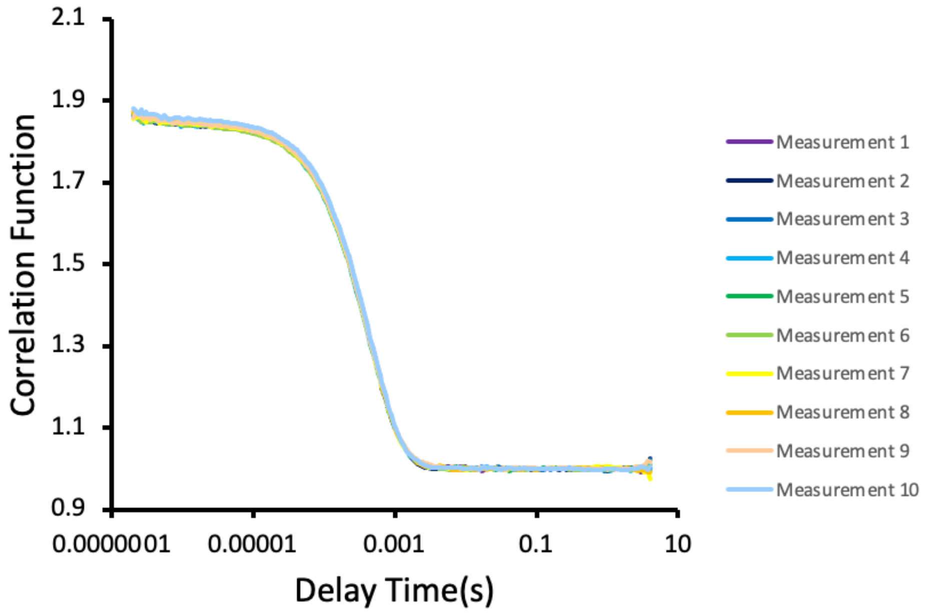

Figure S66 – The correlation function data for 10 DLS runs of **9** (5.56 mM) in H_2O/5 % EtOH at 298 K (1st repeat).

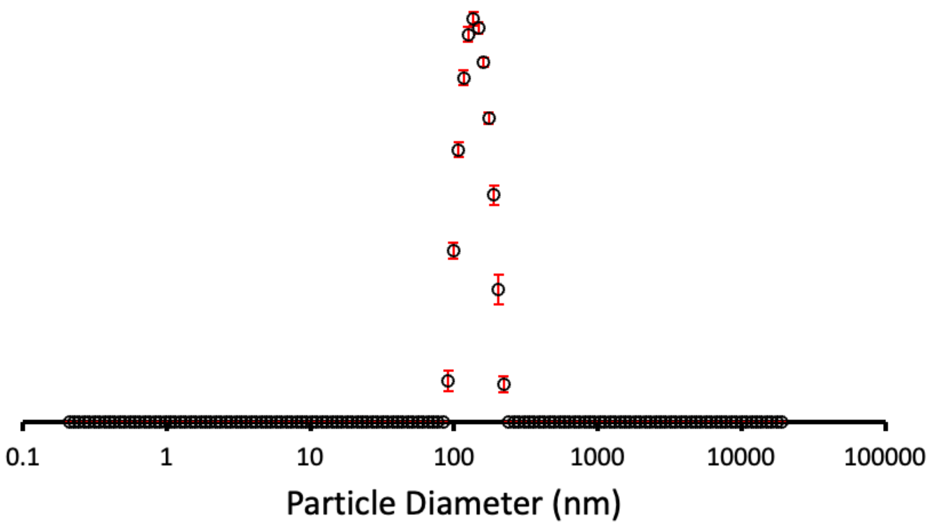

Figure S67 – The average intensity particle size distribution calculated using 10 DLS runs of **9** (5.56 mM) in H_2O/5 % EtOH at 298 K (2nd repeat), with a peak maximum of 143.43 nm.

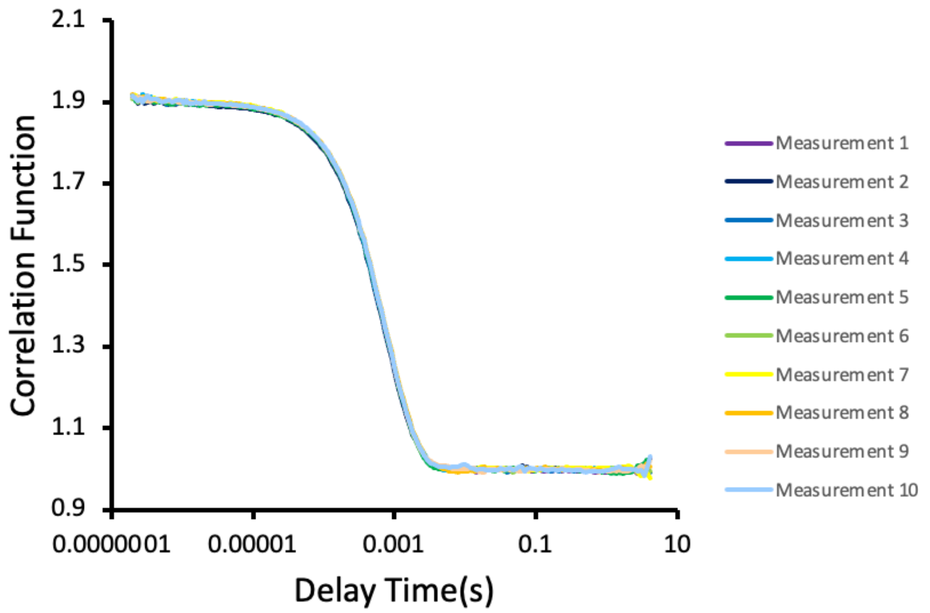

Figure S68 – The correlation function data for 10 DLS runs of **9** (5.56 mM) in H_2O/5 % EtOH at 298 K (2nd repeat).

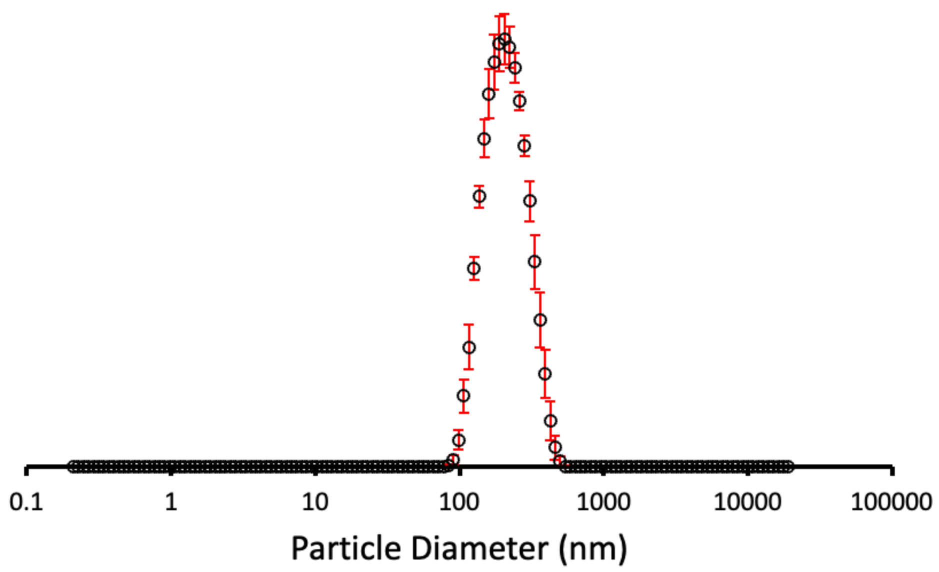

Figure S69 – The average intensity particle size distribution calculated using 10 DLS runs of **9** (5.56 mM) in H_2O/5 % EtOH at 298 K (3rd repeat), with a peak maximum of 218.56 nm.

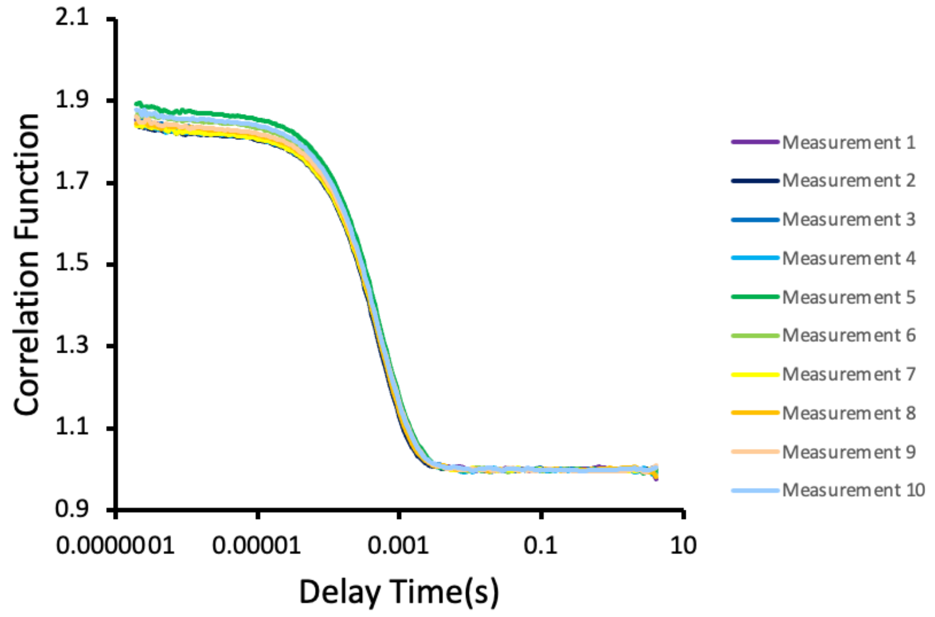

Figure S70 – The correlation function data for 10 DLS runs of **9** (5.56 mM) in H_2O/5 % EtOH at 298 K (3rd repeat).

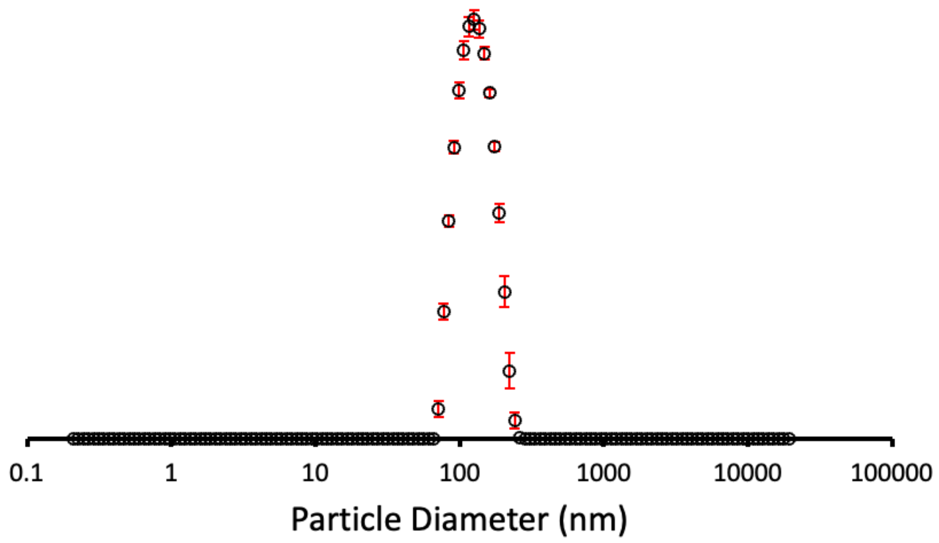

Figure S71 – The average intensity particle size distribution calculated using 10 DLS runs of a 1:1 enantiomeric mixture of **7** + **9** (5.56 mM) in H_2O/5 % EtOH at 298 K (1st repeat), with a peak maximum of 159.61 nm.

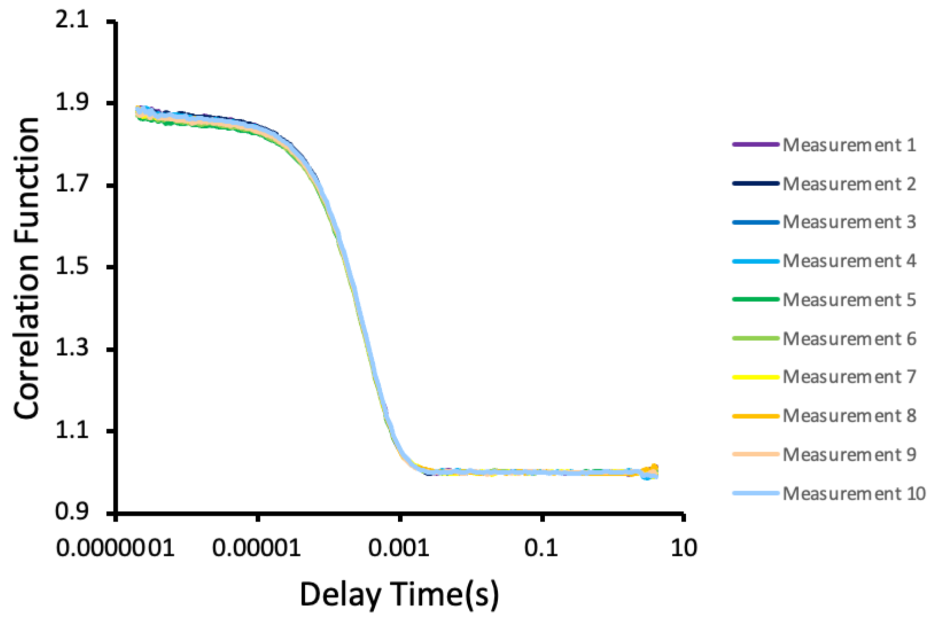

Figure S72 – The correlation function data for 10 DLS runs of a 1:1 enantiomeric mixture of **7** + **9** (5.56 mM) in H_2O/5 % EtOH at 298 K (1st repeat).

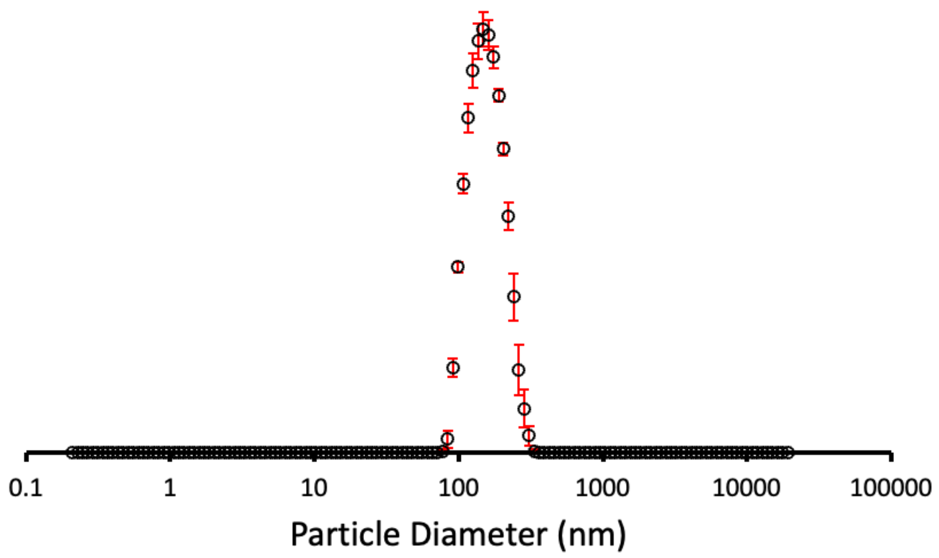

Figure S73 – The average intensity particle size distribution calculated using 10 DLS runs of a 1:1 enantiomeric mixture of **7** + **9** (5.56 mM) in H_2O/5 % EtOH at 298 K (2nd repeat), with a peak maximum of 147.14 nm.

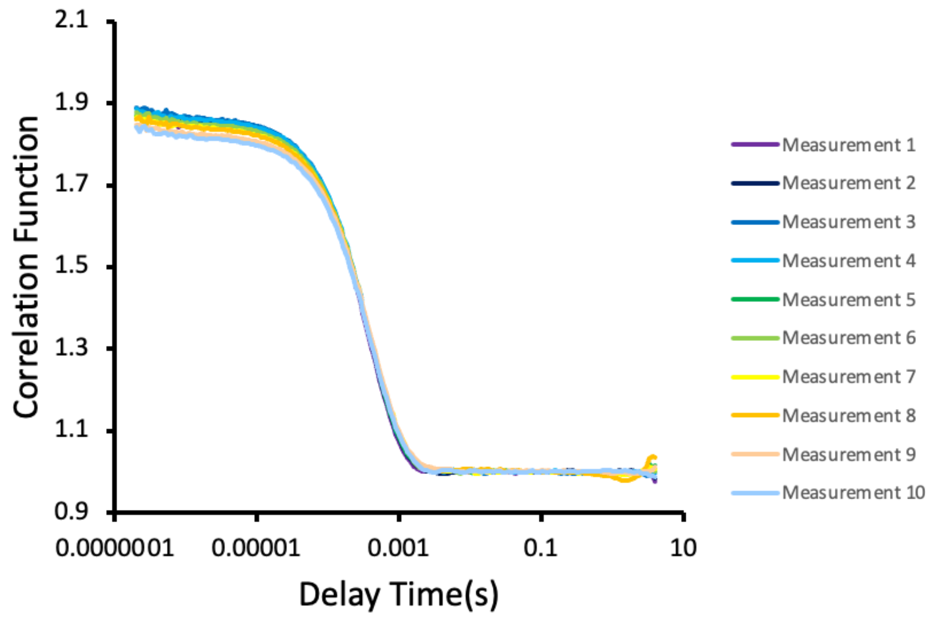

Figure S74 – The correlation function data for 10 DLS runs of a 1:1 enantiomeric mixture of **7** + **9** (5.56 mM) in H_2O/5 % EtOH at 298 K (2nd repeat).

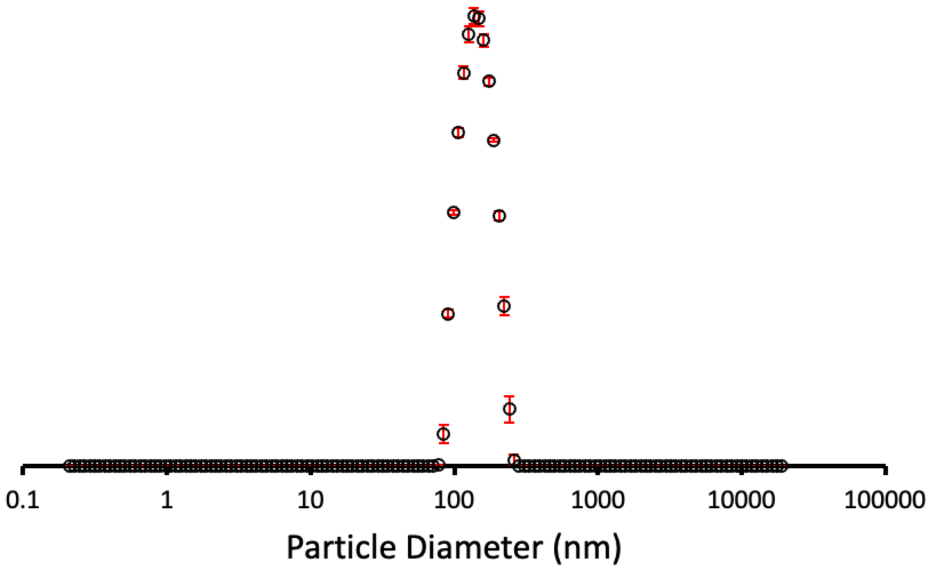

Figure S75 – The average intensity particle size distribution calculated using 10 DLS runs of a 1:1 enantiomeric mixture of **7** + **9** (5.56 mM) in H_2O/5 % EtOH at 298 K (3rd repeat), with a peak maximum of 132.09 nm.

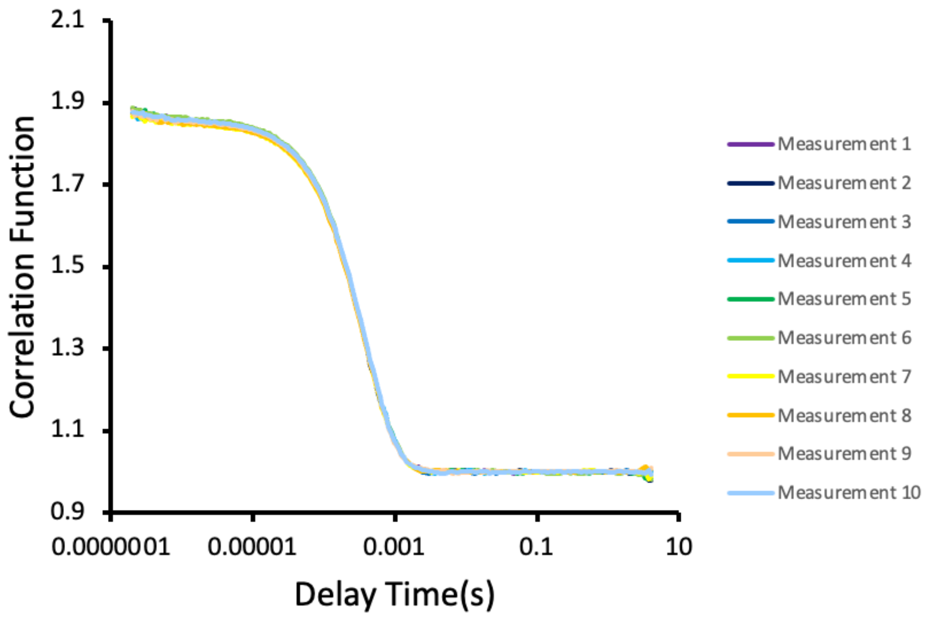

Figure S76 – The correlation function data for 10 DLS runs of a 1:1 enantiomeric mixture of **7** + **9** (5.56 mM) in H_2O/5 % EtOH at 298 K (3rd repeat).

Summary

Table S9 - The peak maxima and polydispersity indices obtained for **1**, **3**, **5**, **7**, **9**, and 1:1 enantiomeric mixtures of **3** + **5** and **7** + **9** obtained at 5.56 mM in H_2O/5 % EtOH. An annealing process was applied in which the samples were heated to approximately 313 K before being cooled down to a measurement temperature of 298 K. The errors given are the standard error of the average.

Compound	Repeat	Peak maxima (nm)	Polydispersity index
**1**	1	220	*b*
**3**	1	290.88	0.040
	2	262.00	0.040
	3	244.06	0.034
	Average	265.65 (± 19.29)	0.038 (± 0.0028)
**5**	1	159.93	0.032
	2	170.91	0.039
	3	191.31	0.029
	Average	174.05 (± 13.00)	0.033 (± 0.0042)
**3** + **5** [a]	1	234.81	0.042
	2	241.14	0.048
	3	238.58	0.050
	Average	238.18 (± 2.60)	0.047 (0.0034)
**7**	1	188.66	0.050
	2	154.17	0.019
	3	157.03	0.017
	Average	166.62 (± 19.14)	0.029 (± 0.015)
**9**	1	177.48	0.028
	2	143.43	0.011
	3	218.55	0.039
	Average	180.99 (± 53.12)	0.026 (± 0.012)
**7** + **9** [a]	1	132.08	0.020
	2	147.14	0.016
	3	159.61	0.022
	Average	146.27(± 13.78)	0.019 (±0.0025)

a – 1:1 mixture.

b – Data not available.

Section S11: Zeta potential studies

Zeta potential studies in H_2O/5 % EtOH

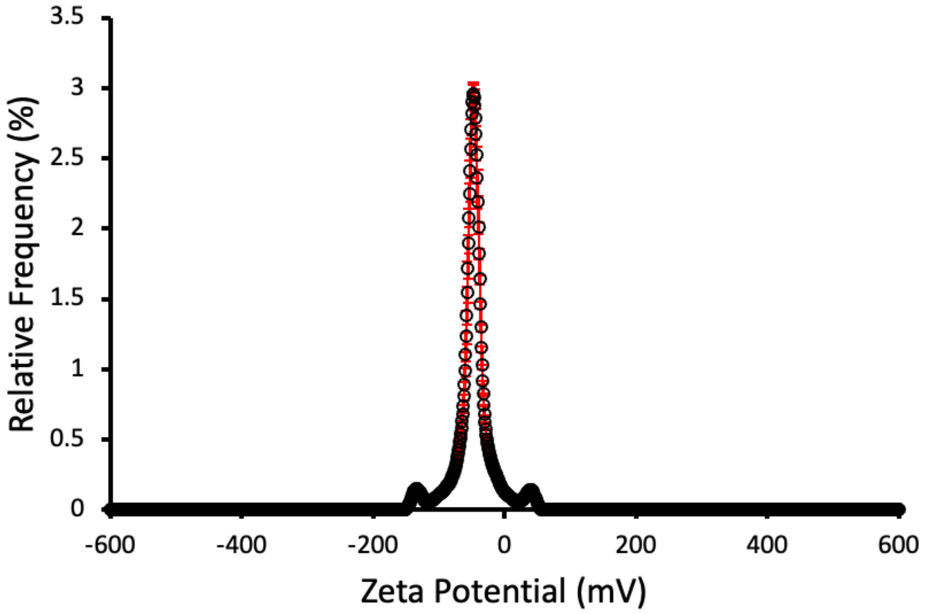

Figure S77 – The average zeta potential distribution for **3** (5.56 mM) in H_2O/5 % EtOH calculated using 10 runs at 298 K (1st repeat). Average measurement value = -48.20 mV.

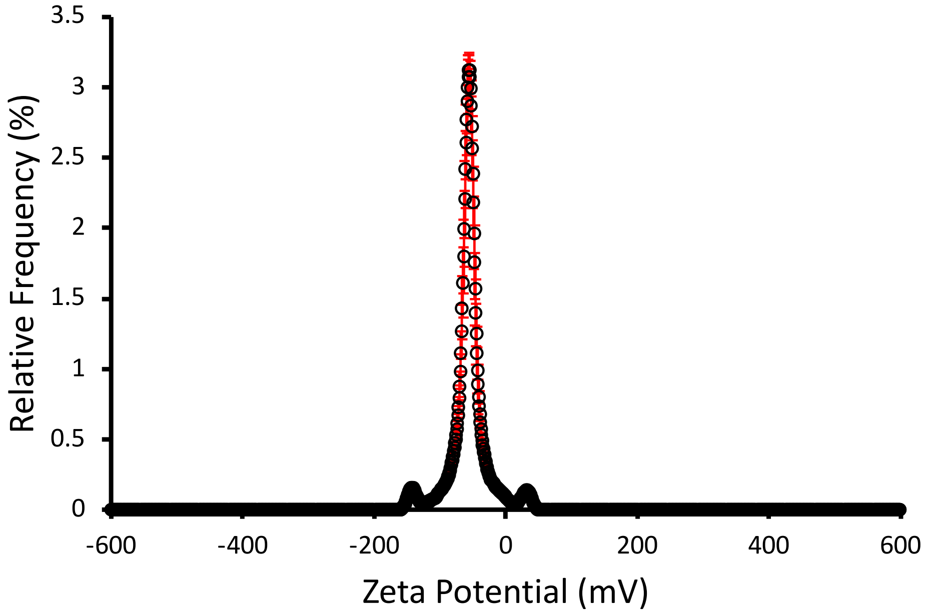

Figure S78 – The average zeta potential distribution for **3** (5.56 mM) in H_2O/5 % EtOH calculated using 10 runs at 298 K (2nd repeat). Average measurement value = -58.22 mV.

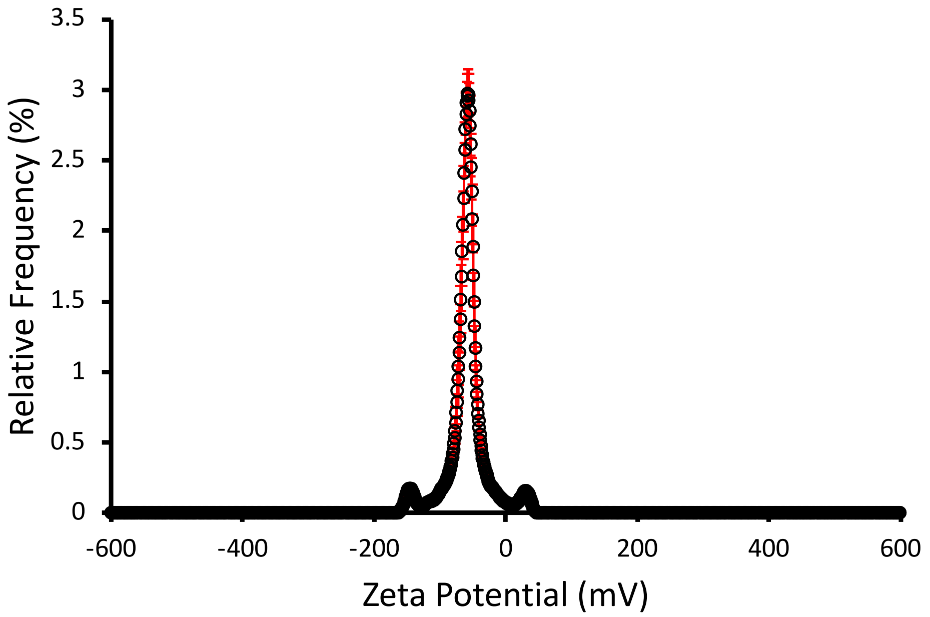

Figure S79 – The average zeta potential distribution for **3** (5.56 mM) in H_2O/5 % EtOH calculated using 10 runs at 298 K (3rd repeat). Average measurement value = -59.86 mV.

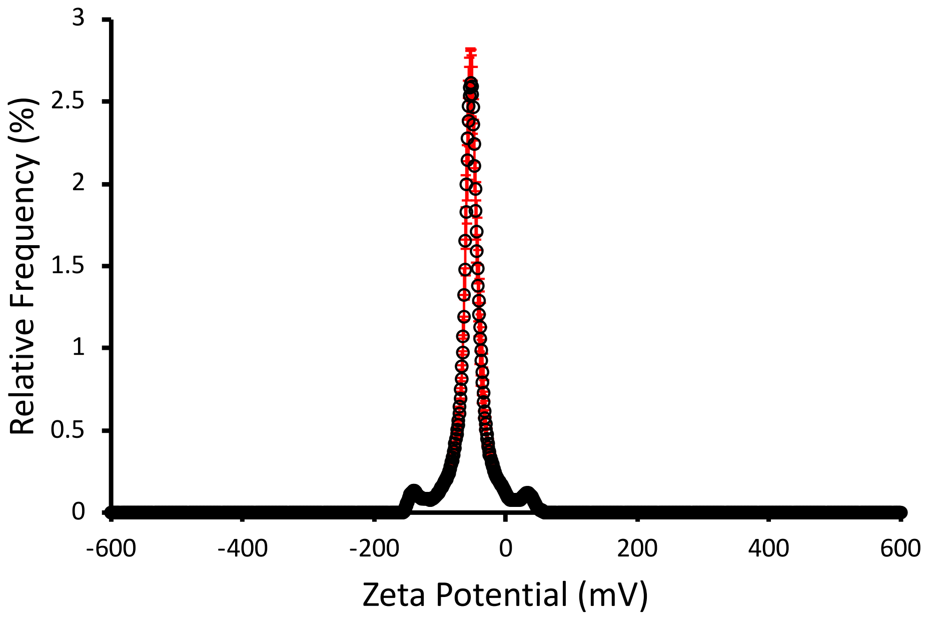

Figure S80 – The average zeta potential distribution for **5** (5.56 mM) in H_2O/5 % EtOH calculated using 10 runs at 298 K (1st repeat). Average measurement value = -56.99 mV.

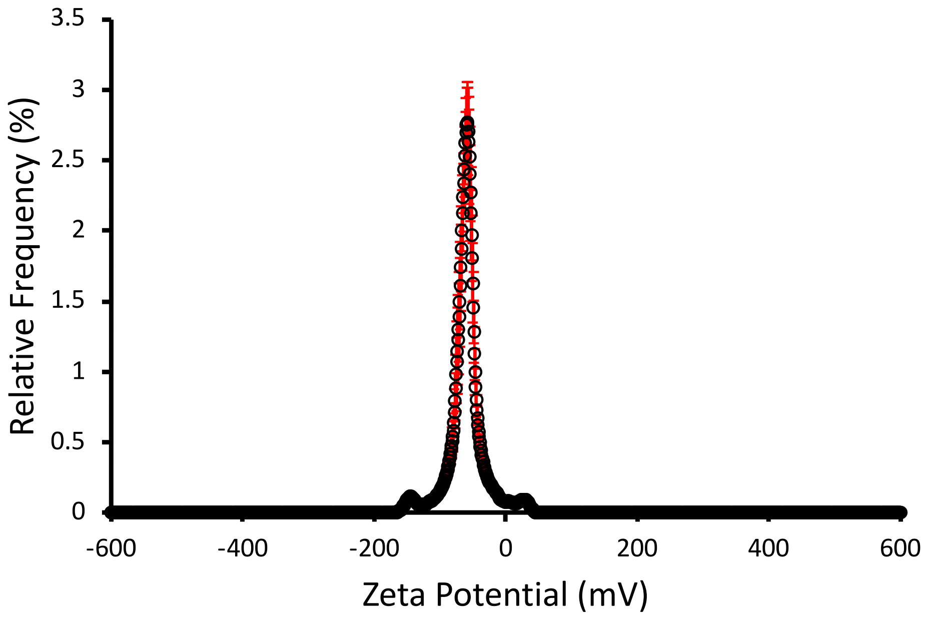

Figure S81 – The average zeta potential distribution for **5** (5.56 mM) in H_2O/5 % EtOH calculated using 10 runs at 298 K (2nd repeat). Average measurement value = -56.13 mV.

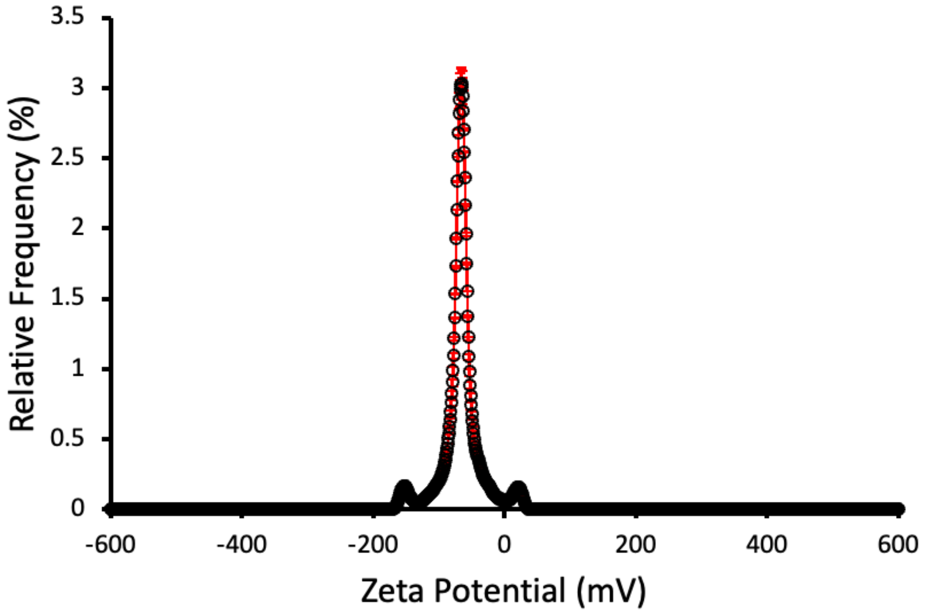

Figure S82 – The average zeta potential distribution for **5** (5.56 mM) in H_2O/5 % EtOH calculated using 10 runs at 298 K (3rd repeat). Average measurement value = -61.00 mV.

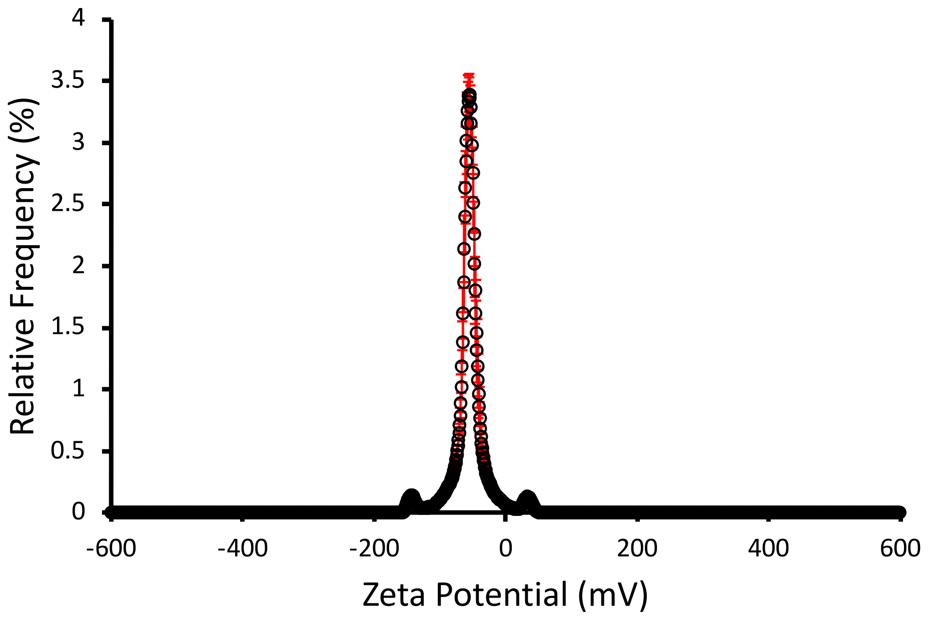

Figure S83 - The average zeta potential distribution for a 1:1 enantiomeric mixture of **3** + **5** (5.56 mM) in H_2O/5 % EtOH calculated using 10 runs at 298 K (1st repeat). Average measurement value = -58.17 mV.

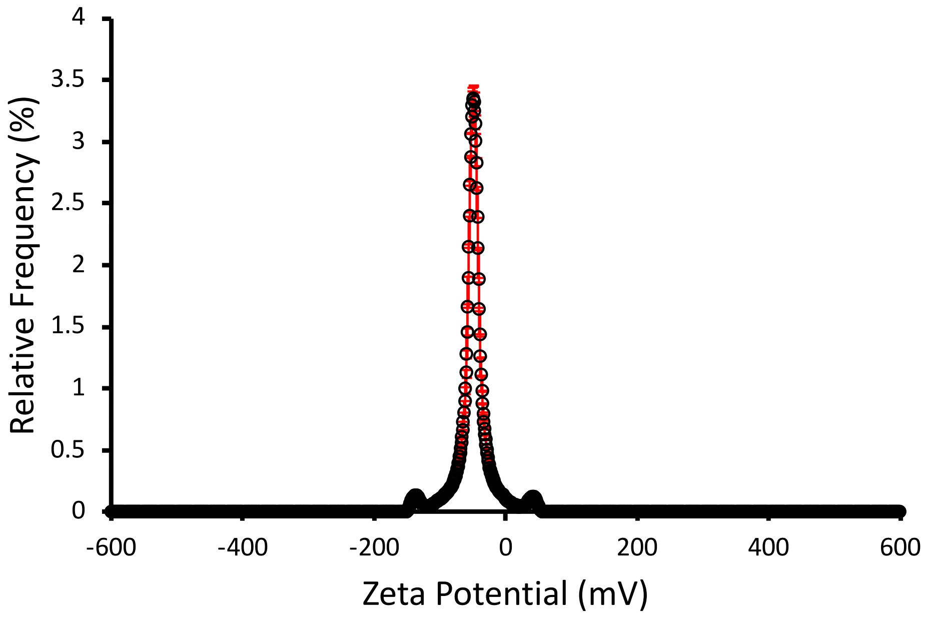

Figure S84 - The average zeta potential distribution for a 1:1 enantiomeric mixture of **3** + **5** (5.56 mM) in H_2O/5 % EtOH calculated using 10 runs at 298 K (2nd repeat). Average measurement value = -56.53 mV.

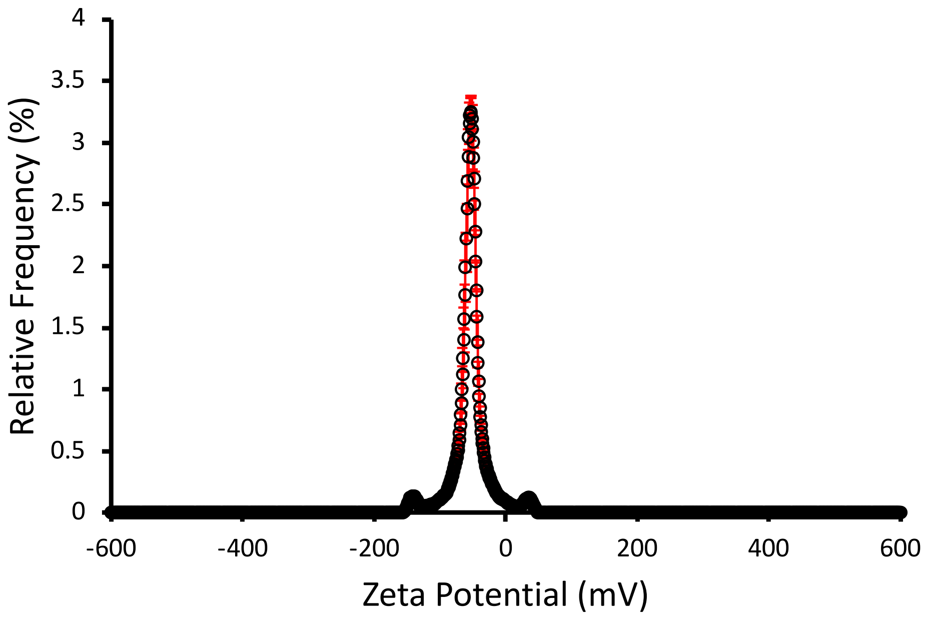

Figure S85 - The average zeta potential distribution for a 1:1 enantiomeric mixture of **3** + **5** (5.56 mM) in H_2O/5 % EtOH calculated using 10 runs at 298 K (3rd repeat). Average measurement value = -56.00 mV.

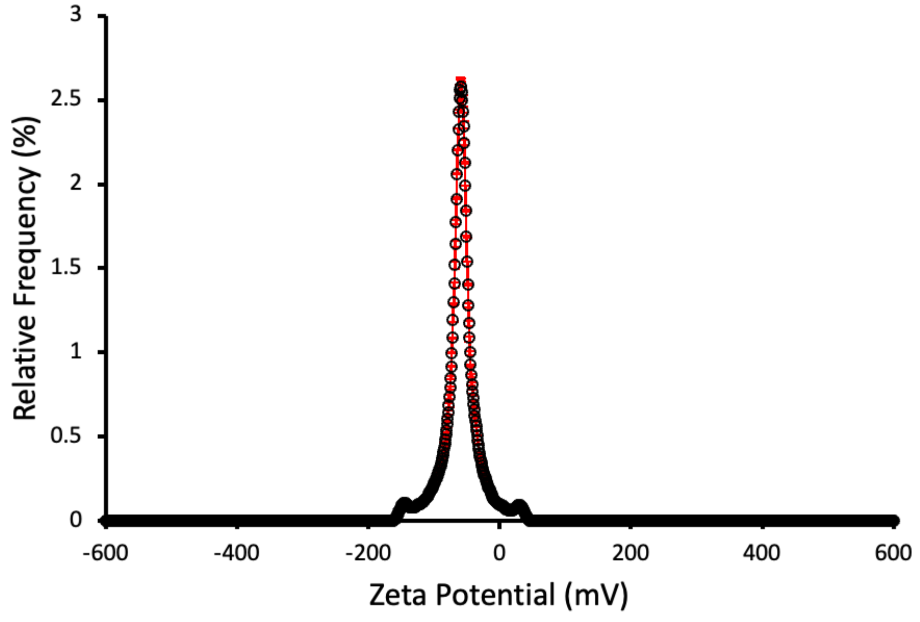

Figure S86 – The average zeta potential distribution for **7** (5.56 mM) in H_2O/5 % EtOH calculated using 10 runs at 298 K (1st repeat). Average measurement value = -59.08 mV.

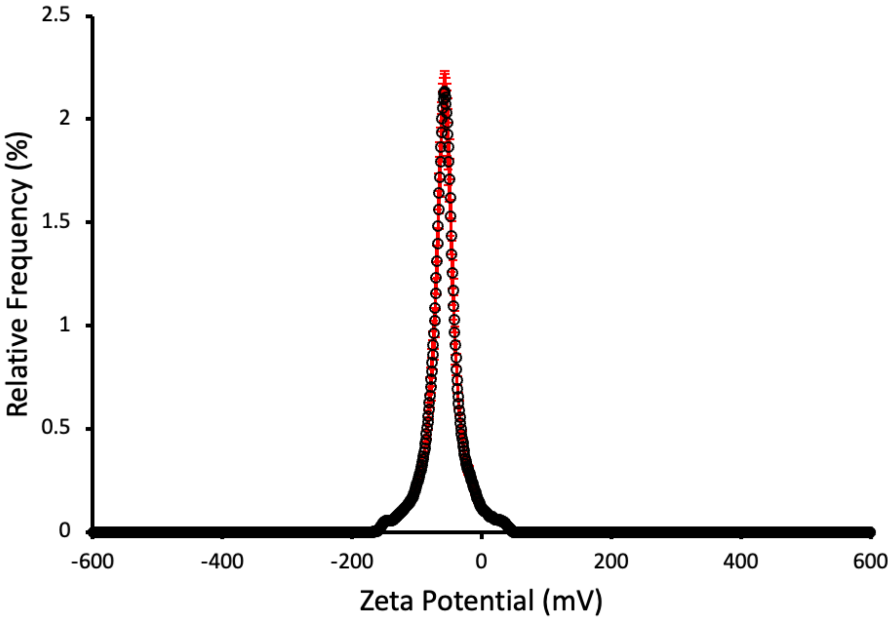

Figure S87 – The average zeta potential distribution for **7** (5.56 mM) in H_2O/5 % EtOH calculated using 10 runs at 298 K (2nd repeat). Average measurement value = -54.44 mV.

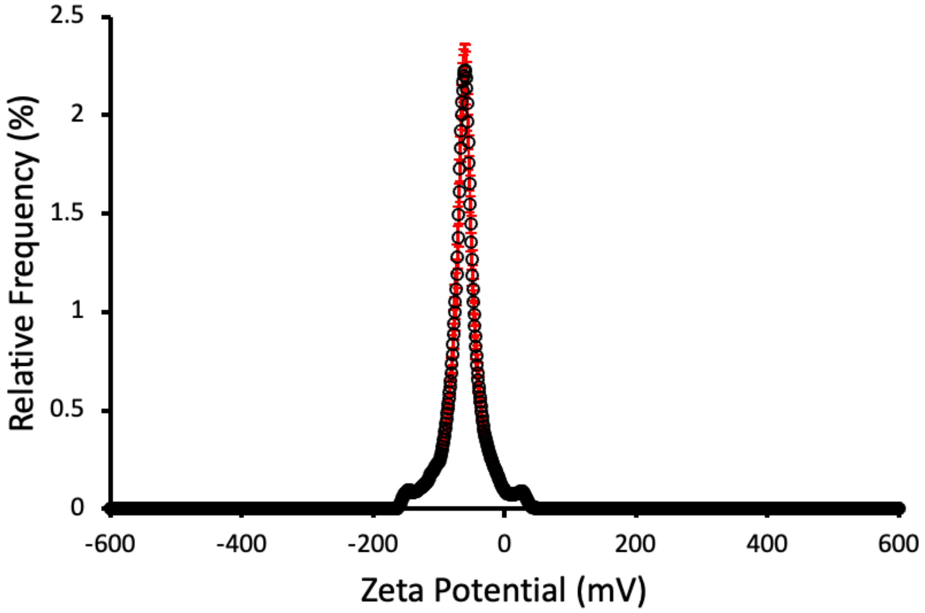

Figure S88 – The average zeta potential distribution for **7** (5.56 mM) in H_2O/5 % EtOH calculated using 10 runs at 298 K (3rd repeat). Average measurement value = -59.62 mV.

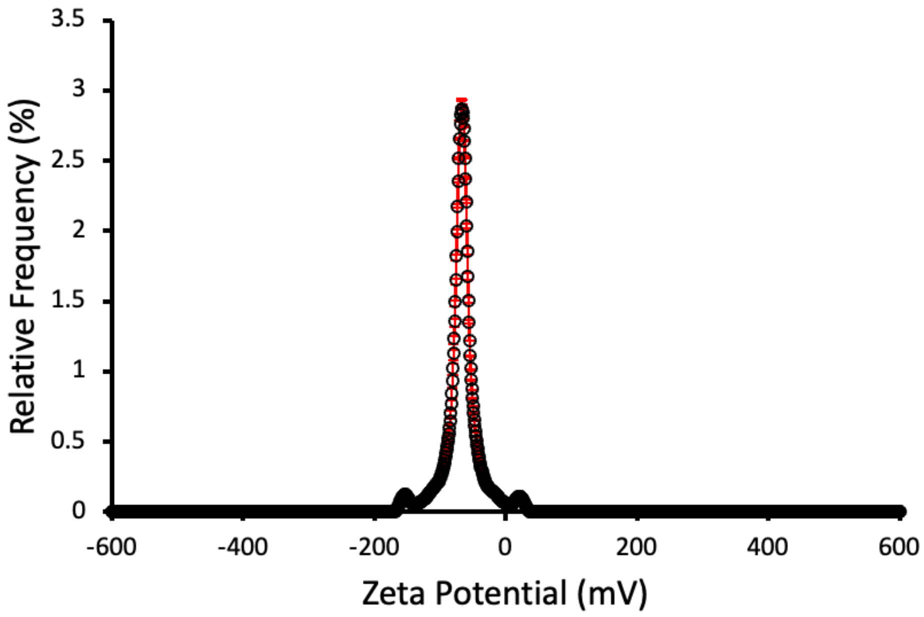

Figure S89 – The average zeta potential distribution for **9** (5.56 mM) in H_2O/5 % EtOH calculated using 10 runs at 298 K (1st repeat). Average measurement value = -66.78 mV.

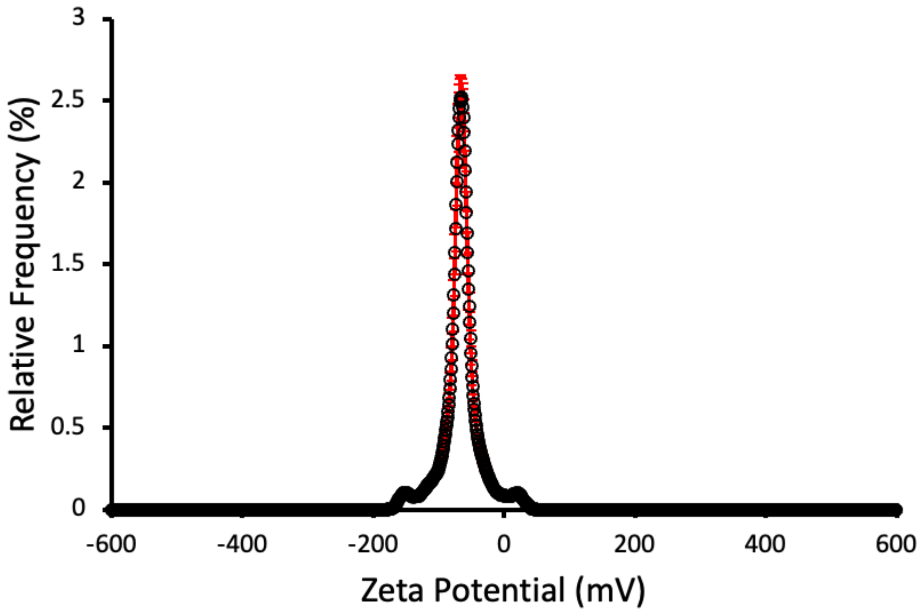

Figure S90 – The average zeta potential distribution for **9** (5.56 mM) in H_2O/5 % EtOH calculated using 10 runs at 298 K (2nd repeat). Average measurement value = -64.75 mV.

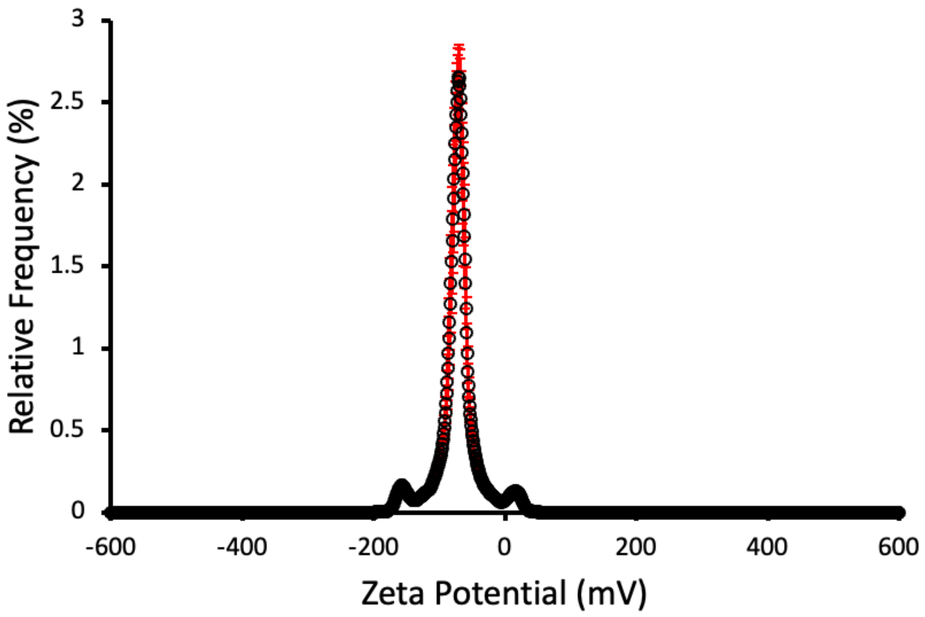

Figure S91 – The average zeta potential distribution for **9** (5.56 mM) in H_2O/5 % EtOH calculated using 10 runs at 298 K (3rd repeat). Average measurement value = -67.30 mV.

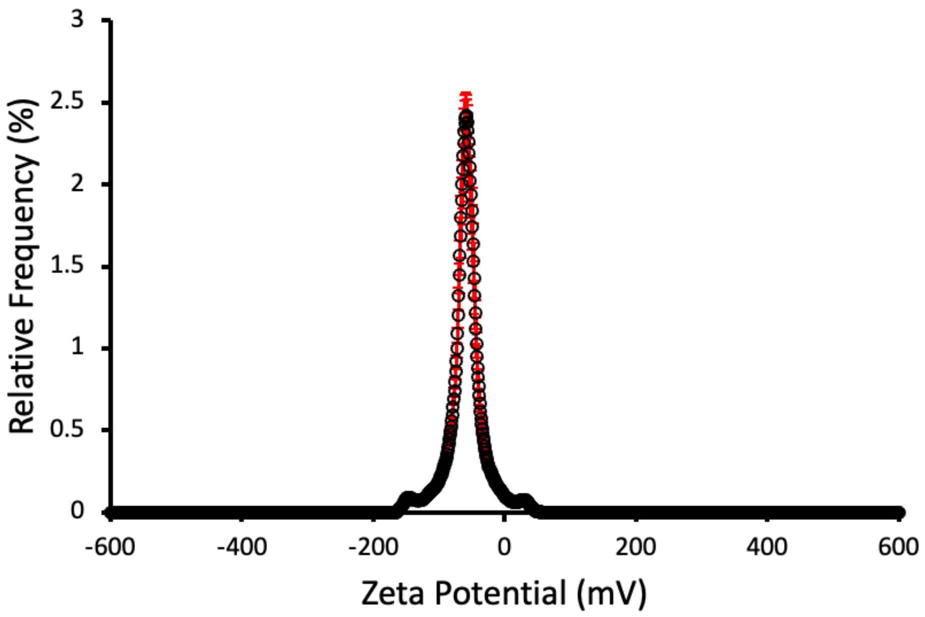

Figure S92 - The average zeta potential distribution for a 1:1 enantiomeric mixture of **7** + **9** (5.56 mM) in H_2O/5 % EtOH calculated using 10 runs at 298 K (1st repeat). Average measurement value = -65.52 mV.

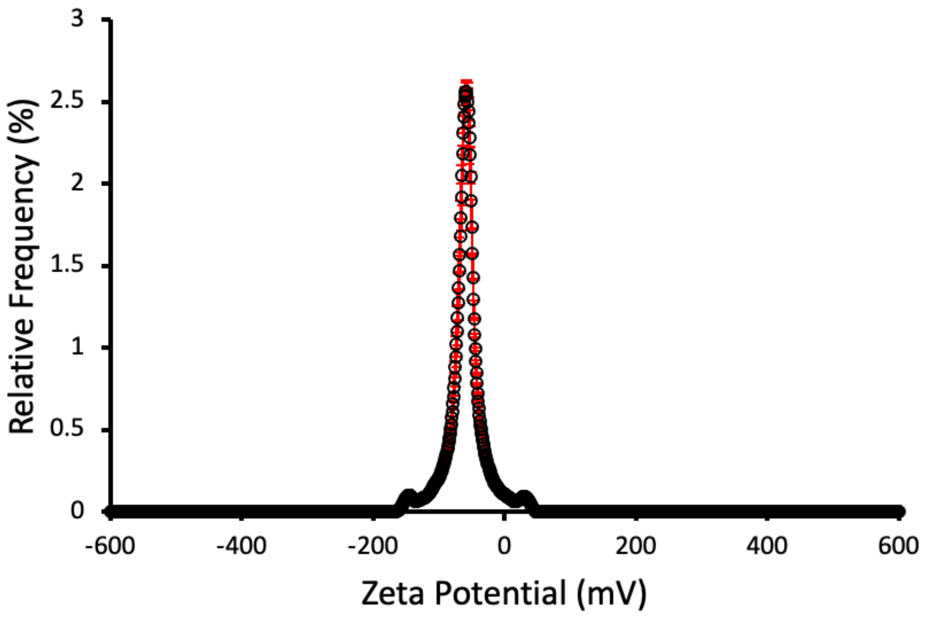

Figure S93 - The average zeta potential distribution for a 1:1 enantiomeric mixture of **7** + **9** (5.56 mM) in H_2O/5 % EtOH calculated using 10 runs at 298 K (2nd repeat). Average measurement value = -64.69 mV.

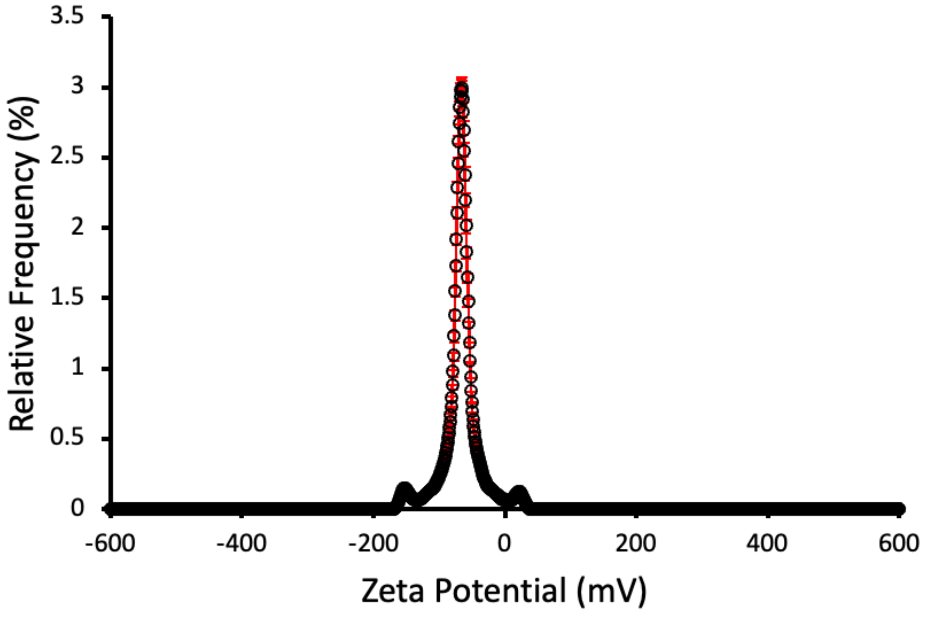

Figure S94 - The average zeta potential distribution for a 1:1 enantiomeric mixture of **7** + **9** (5.56 mM) in H_2O/5 % EtOH calculated using 10 runs at 298 K (3rd repeat). Average measurement value = -65.70 mV.

Summary

Table S10 – Summary of the average zeta potential for **1**, **3**, **5**, **7**, **9**, and 1:1 enantiomeric mixture of **3** + **5** and **7** + **9** at 5.56 mM in H_2O/5 % EtOH.

**Compound**	***n***	**Zeta potential (mV)**
**1**	1	-37
**3**	1	-48.20
	2	-58.22
	3	-59.86
	**Average**	-55.43 (± 3.64)
**5**	1	-56.99
	2	-56.13
	3	-61.00
	**Average**	-58.04 (± 1.50)
**3+5** [*a*]	1	-58.17
	2	-56.53
	3	-56.00
	**Average**	-56.90 (± 0.65)
**7**	1	-59.62
	2	-59.08
	3	-54.44
	**Average**	-57.72 (± 1.64)
**9**	1	-66.78
	2	-64.75
	3	-67.30
	**Average**	-66.28 (± 0.78)
**7+9** [*a*]	1	-65.52
	2	-64.69
	3	-65.70
	**Average**	-65.31 (± 0.31)

a – 1:1 mixture.

Error = standard error of the mean.

Section S12: Surface tension measurements and critical aggregate concentration (CAC) determination

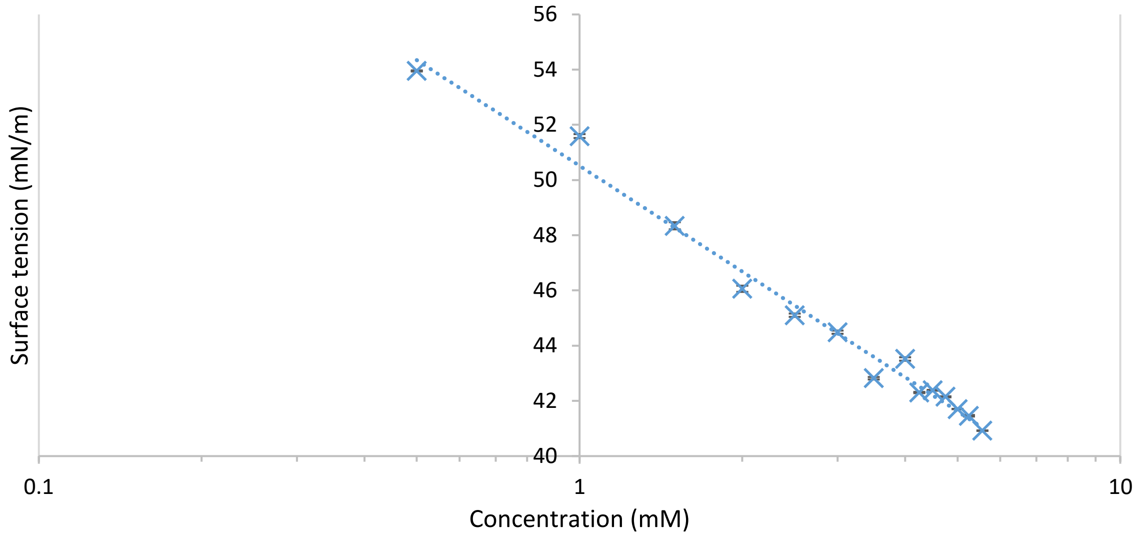

Figure S95 – Determination of the critical aggregate concentration of **3** in H_2O/5 % EtOH using surface tension measurements. The CAC is above the limit of solubility (5.56 mM), at which the surface tension is 40.92 mN/m.

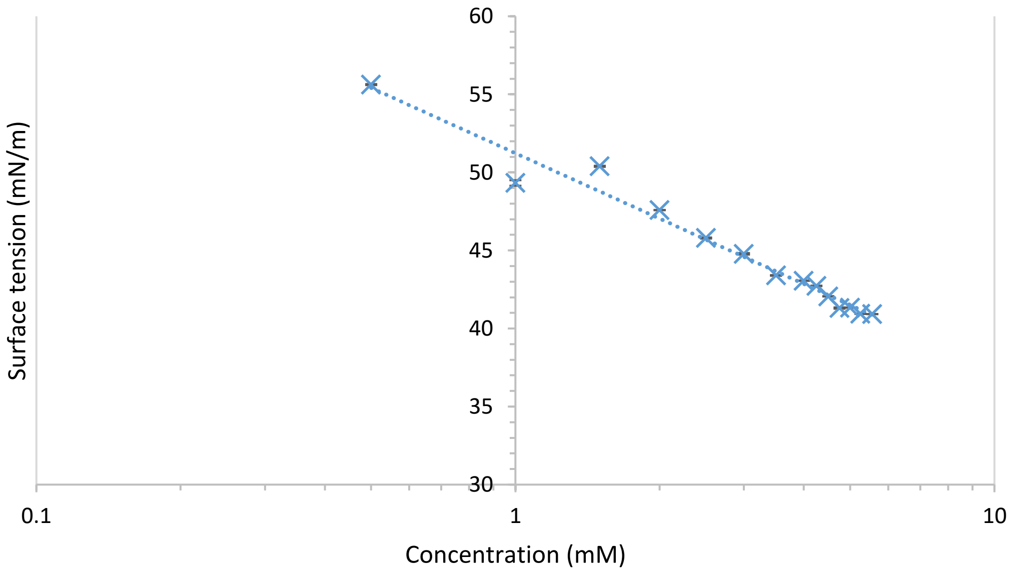

Figure S96 - Determination of the critical aggregate concentration of **5** in H_2O/5 % EtOH using surface tension measurements. The CAC is above the limit of solubility (5.56 mM), at which the surface tension is 40.92 mN/m.

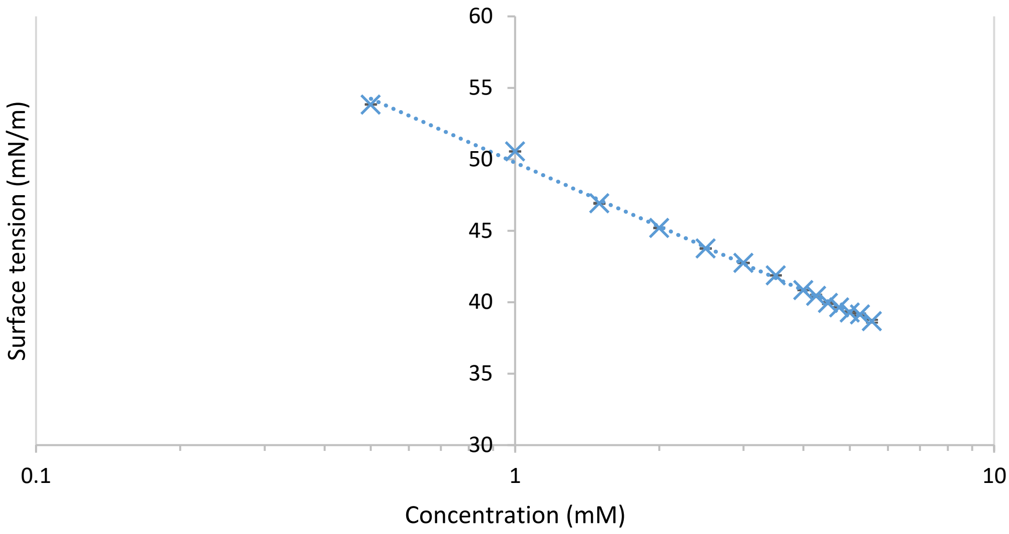

Figure S97 - Determination of the critical aggregate concentration of **3** + **5** in H_2O/5 % EtOH using surface tension measurements. The CAC is above the limit of solubility (5.56 mM), at which the surface tension is 38.67 mN/m.

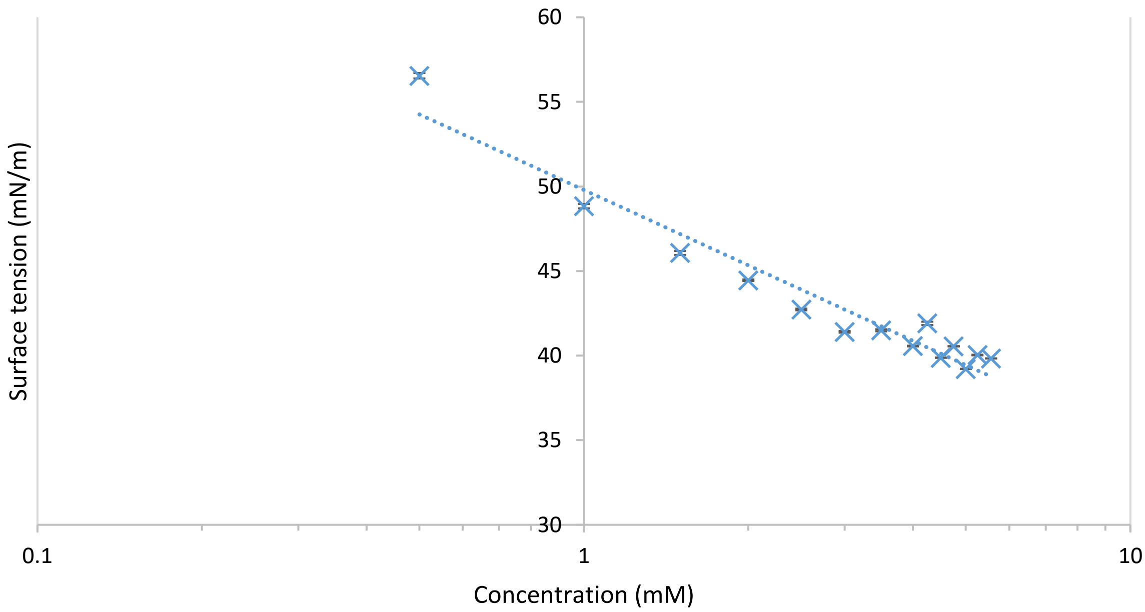

Figure S98 - Determination of the critical aggregate concentration of **7** in H_2O/5 % EtOH using surface tension measurements. The CAC is above the limit of solubility (5.56 mM), at which the surface tension is 39.83 mN/m.

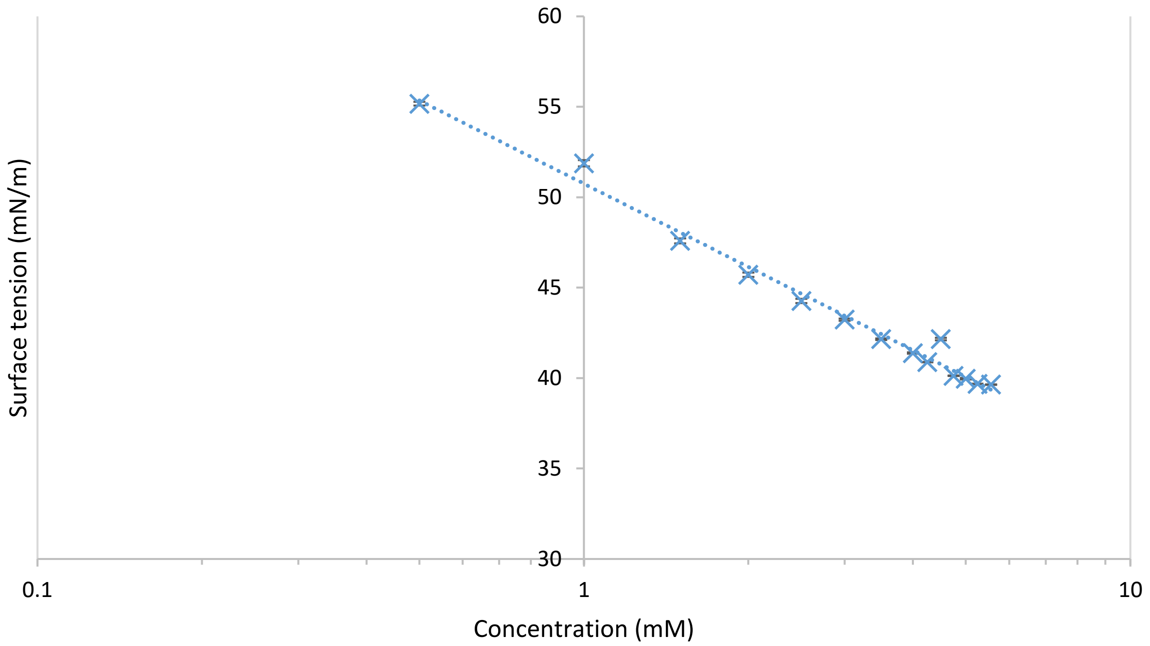

Figure S99 - Determination of the critical aggregate concentration of **9** in H_2O/5 % EtOH using surface tension measurements. The CAC is above the limit of solubility (5.56 mM), at which the surface tension is 39.64 mN/m.

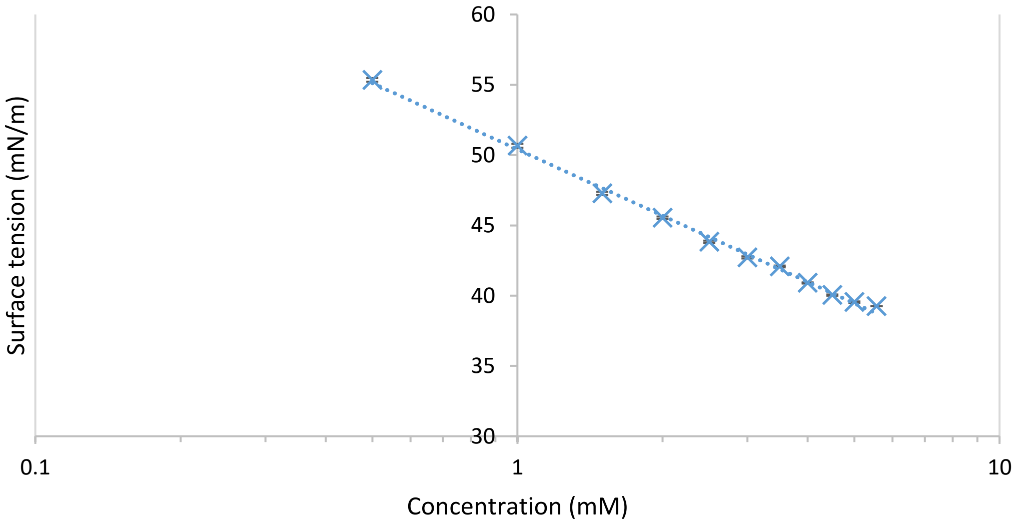

Figure S100 - Determination of the critical aggregate concentration of **7** + **9** in H_2O/5 % EtOH using surface tension measurements. The CAC is above the limit of solubility (5.56 mM), at which the surface tension is 39.25 mN/m.

Section S13: Single crystal X-ray structures

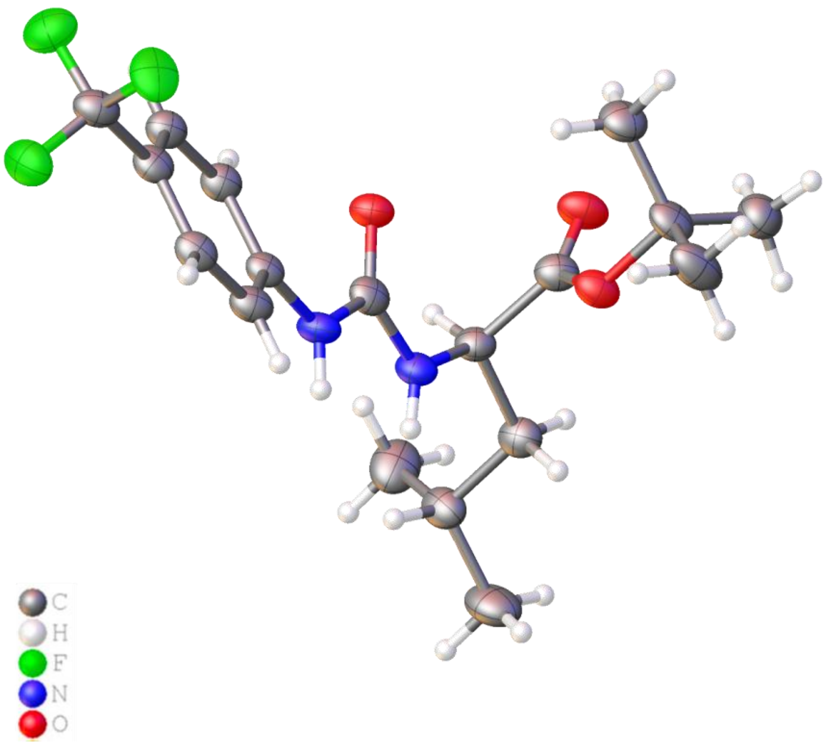

Figure S101 – Single crystal X-ray structure of **2**: red = oxygen; green = fluorine; blue = nitrogen; white = hydrogen; grey = carbon. CCDC 2205767, $C_{18}H_{25}F_3N_2O_3$ (M = 374.40): orthorhombic, space group P 21 21 21, a = 5.5098(7) Å, b = 18.427(4) Å, c = 18.838(3) Å, α = 90°, β = 90°, γ = 90°, V = 1912.6(6) $Å^3$, Z = 4, T = 100(1) K, CuK\α = 1.5418 Å, Dcalc = 1.300 g/cm^3, 12906 reflections measured (9.390 ≤ 2Θ ≤ 148.174), 3720 unique (R_{int} = 0.1367, R_{sigma} = 0.1210) which were used in all calculations. The final R_1 was 0.0591 (I > 2σ(I)) and wR_2 was 0.1361 (all data).

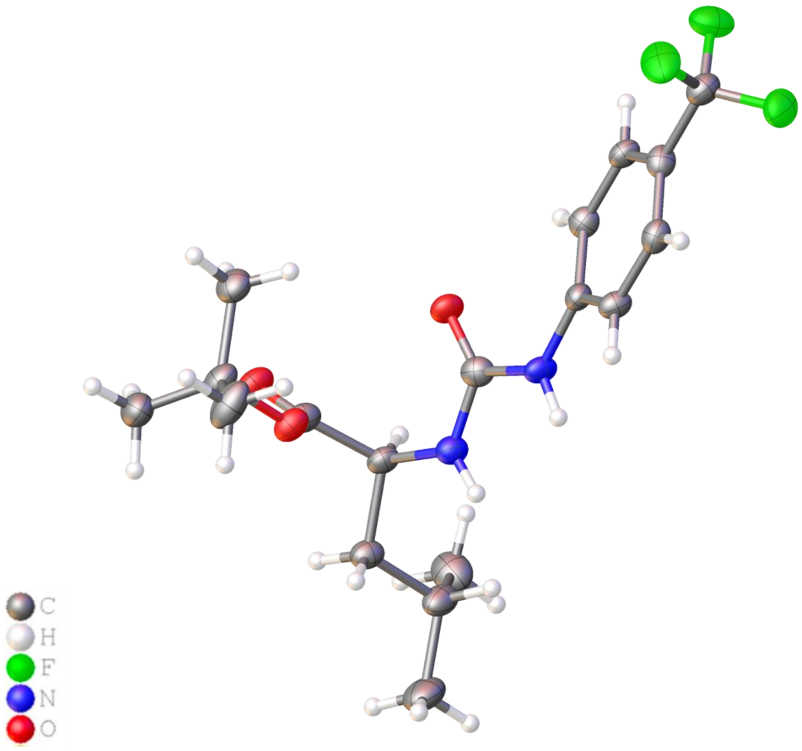

Figure S102 – Single crystal X-ray structure of **4**: red = oxygen; green = fluorine; blue = nitrogen; white = hydrogen; grey = carbon. CCDC 2205768, $C_{18}H_{25}F_3N_2O_3$ (M = 374.40): orthorhombic, space group P 21 21 21, a = 5.5068(4) Å, b = 18.3998(17) Å, c = 18.8406(14) Å, α = 90°, β = 90°, γ = 90°, V = 1909(3) $Å^3$, Z = 4, T = 100(1) K, CuK\α = 1.5418 Å, Dcalc = 1.303 g/cm^3, 12686 reflections measured (9.388 ≤ 2Θ ≤ 143.696), 3691 unique (R_{int} = 0.0935, R_{sigma} = 0.0908) which were used in all calculations. The final R_1 was 0.0663 (I > 2σ(I)) and wR_2 was 0.1531 (all data).

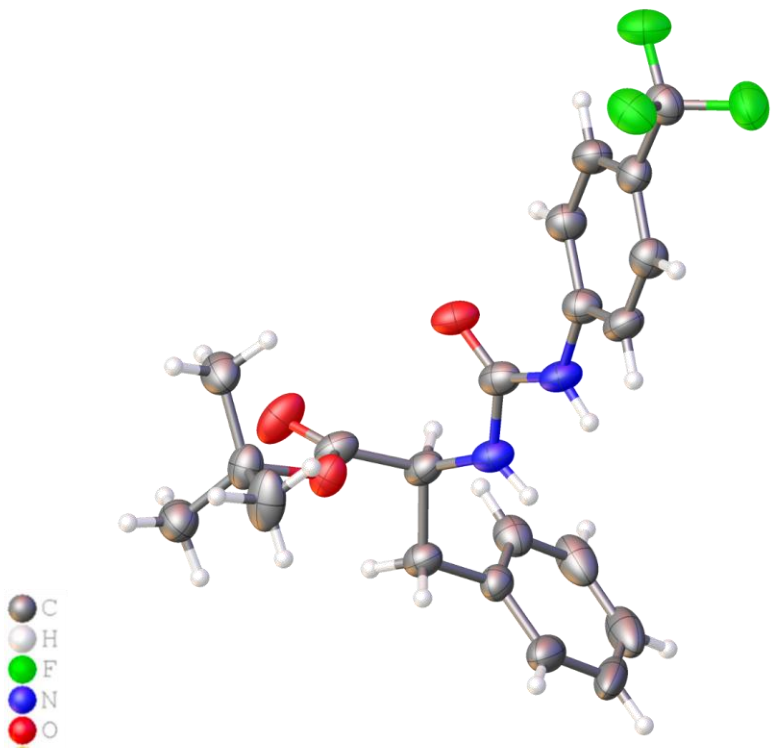

Figure S103 - Single crystal X-ray structure of **6**: red = oxygen; green = fluorine; blue = nitrogen; white = hydrogen; grey = carbon. CCDC 2205765, $C_{21}H_{23}F_3N_2O_3$ (M = 408.41): orthorhombic, space group P 21 21 21, a = 5.7353(4) Å, b = 18.3597(18) Å, c = 19.4042(15) Å, α = 90°, β = 90°, γ = 90°, V = 2043.2(3) Å^3, Z = 4, T = 100(1) K, CuK\α = 1.5418 Å, Dcalc = 1.328 g/cm^3, 13037 reflections measured (9.114 ≤ 2Θ ≤ 144.834), 3930 unique (R_{int} = 0.0790, R_{sigma} = 0.0731) which were used in all calculations. The final R_1 was 0.0717 (I > 2σ(I)) and wR_2 was 0.1927 (all data).

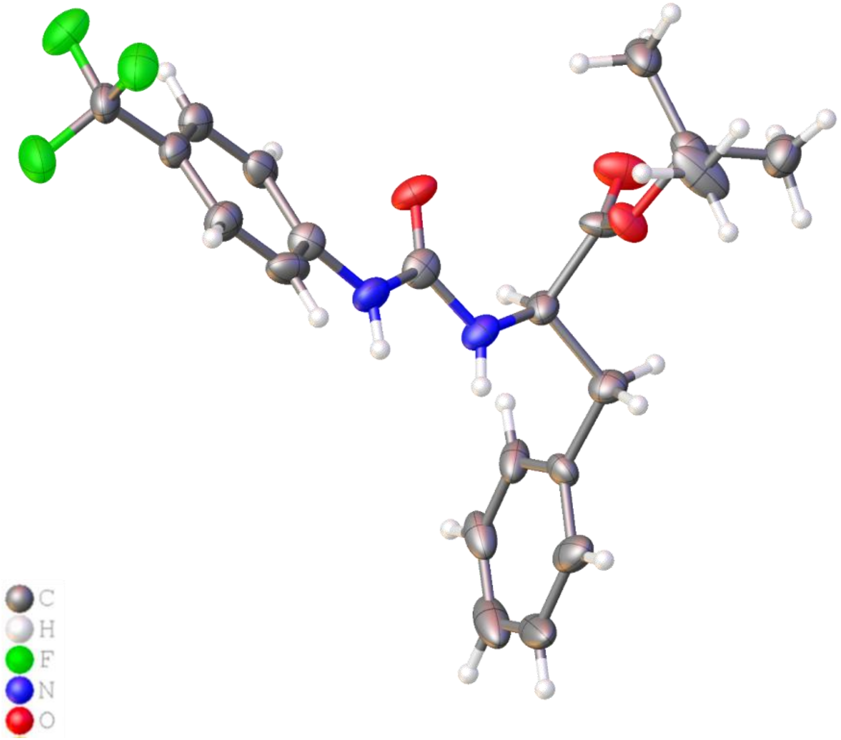

Figure S104 – Single crystal X-ray structure of **8**: red = oxygen; green = fluorine; blue = nitrogen; white = hydrogen; grey = carbon. CCDC 2205766, $C_{21}H_{23}F_3N_2O_3$ (M = 408.41): orthorhombic, space group P 21 21 21, a = 5.7453(6) Å, b = 18.227(3) Å, c = 19.3974(16) Å, α = 90°, β = 90°, γ = 90°, V = 2036.8(4) Å^3, Z = 4, T = 100(1) K, CuK\α = 1.5418 Å, Dcalc = 1.332 g/cm^3, 4808 reflections measured (9.118 ≤ 2Θ ≤ 144.298), 3301 unique (R_{int} = 0.1545, R_{sigma} = 0.1629) which were used in all calculations. The final R_1 was 0.1056 (I > 2σ(I)) and wR_2 was 0.3038 (all data).

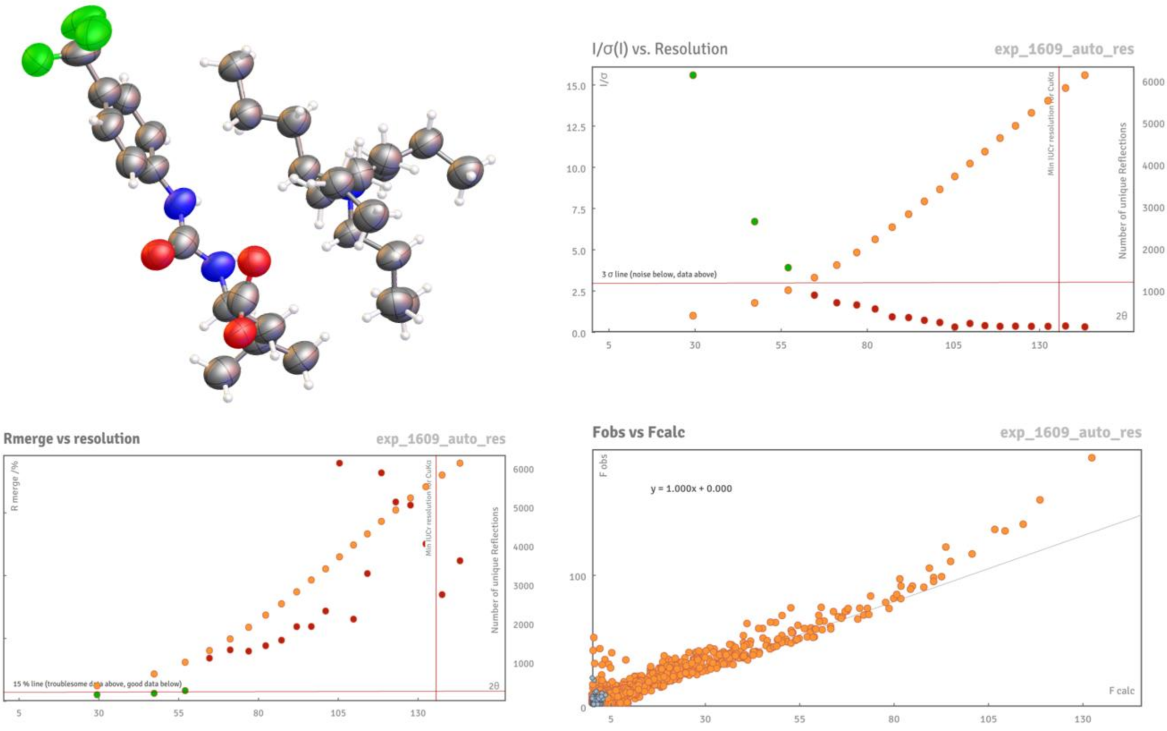

Figure S105 – Single crystal X-ray structure of **3** and **5**, obtained from a 1:1 enantiomeric mixture through slow evaporation from methanol: red = oxygen; blue = nitrogen; white = hydrogen; grey = carbon. CCDC 2218842, $C_{30}H_{52}F_3N_3O_3$ (M = 559.74): monoclinic, space group P 21/c, a = 15.470 (4) Å, b = 18.843 (6) Å, c = 10.856 (3) Å, α = 90°, β = 90.99(3)°, γ = 90°, V = 1164.2(15) Å^3, Z = 4, T = 100(1) K, CuK\α = 1.5418 Å, Dcalc = 1.175 g/cm^3, 21210 reflections measured (7.394 ≤ 2Θ ≤ 133.196), 5590 unique (R_{int} = 0.2749**,** R_{sigma} = 0.1847) which were used in all calculations. The final R_1 was 0.1757 (I > 2σ(I)) and wR_2 was 0.4984 (all data). We acknowledge the poor quality of these data. Repeated attempts to obtain a crystalline sample of this material were obtained over a 1.5-year period. This was the only sample for which sufficient data could be obtained to generate a structure and was obtained from the fracturing of a single plate. The sample did not survive further analysis attempts. These data should only be used to enable the determination of atom connectivity and to approximate atomic location.

Hydrogen bonding summary

Table S11 - Hydrogen bond distances and angles observed for **2**, **4**, **6**, **8**, and **3** + **5** in a 1:1 enantiomeric mixture calculated from the single crystal X-ray structure shown in Figure S101 – Figure S105.

Compound	Hydrogen bond donor	Hydrogen atom	Hydrogen bond acceptor	Hydrogen bond length (D•••A) (Å)	Hydrogen bond angle (D-H•••A) (°)
**2**	N1	H1	O1	3.271(6)	155.4(3)
	N2	H2	O2	2.858(5)	140.4(3)
**4**	N1	H1	O1	2.861(6)	155.8(3)
	N2	H2	O2	3.270(6)	141.1(3)
**6**	N1	H1	O1	3.482(7)	144.4(3)
	N1	H1	O2	3.372(7)	136.0(4)
	N2	H2	O2	2.824(7)	141.9(3)
**8**	N1	H1	O1	3.471(10)	171.84(11)
	N1	H1	O2	3.35(1)	135.3(5)
	N2	H2	O2	2.813(10)	143.7(5)
**3** + **5**	N1	H1	O1	2.889(11)	151.7(6)
	N2	H2	O2	2.736 (11)	172.2(6)

Section S14: Mass spectrometry spectra

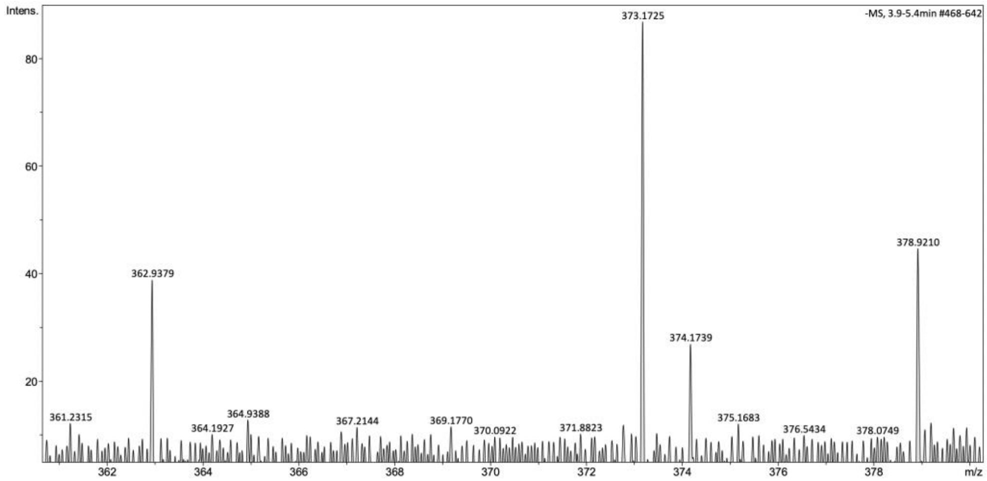

Figure S106 - A high-resolution mass spectrum (ESI$^-$) obtained for **2** in methanol, *m/z* [M]$^-$.

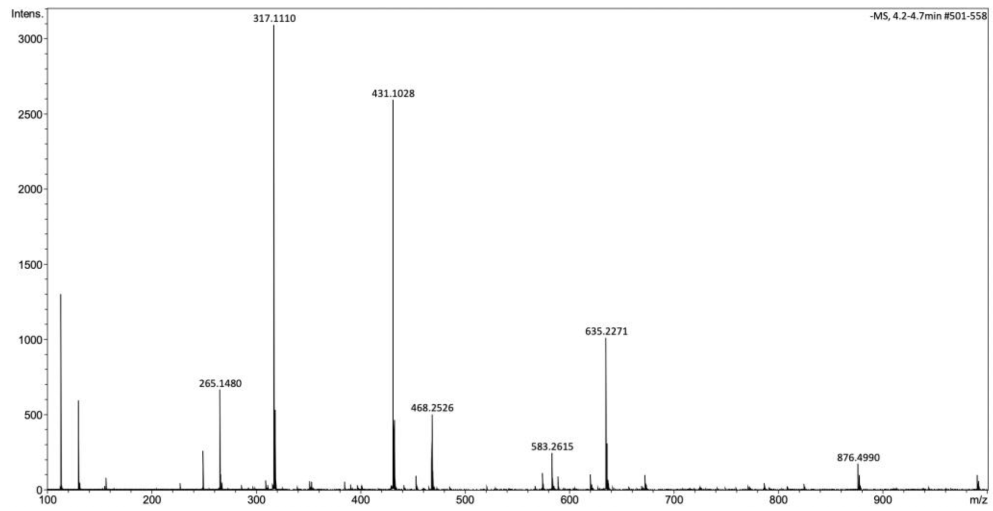

Figure S107 - A high-resolution mass spectrum (ESI$^-$) obtained for **3** in methanol, *m/z* [M]$^-$.

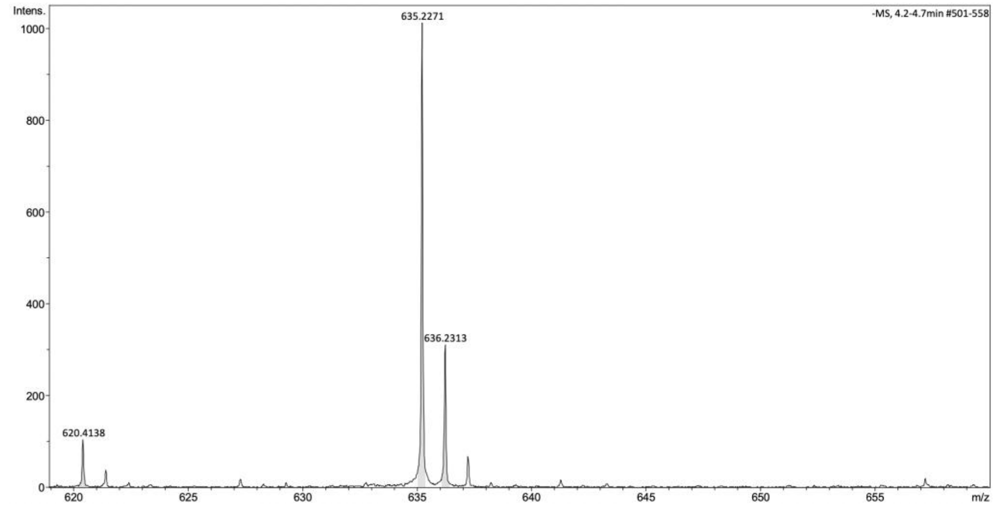

Figure S108 - A high-resolution mass spectrum (ESI$^-$) obtained for dimeric species of **3** in methanol, *m/z* [M + M + H]$^-$.

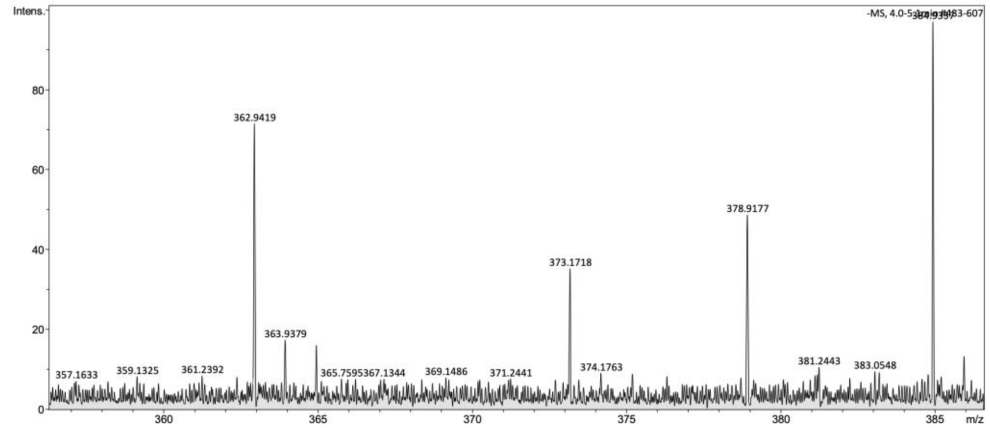

Figure S109 - A high-resolution mass spectrum (ESI$^-$) obtained for **4** in methanol, *m/z* [M]$^-$.

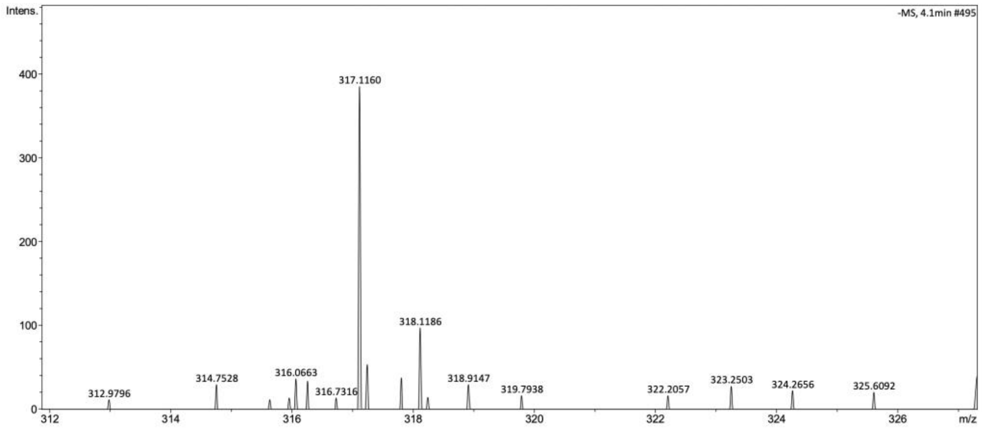

Figure S110 - A high-resolution mass spectrum (ESI$^-$) obtained for **5** in methanol, *m/z* [M]$^-$.

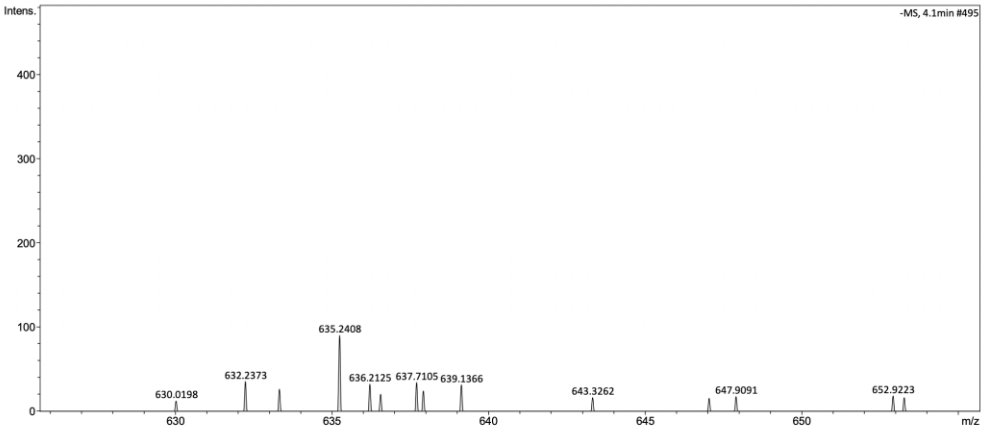

Figure S111 - A high-resolution mass spectrum (ESI$^-$) obtained for dimeric species of **5** in methanol, *m/z* [M + M + H]$^-$.

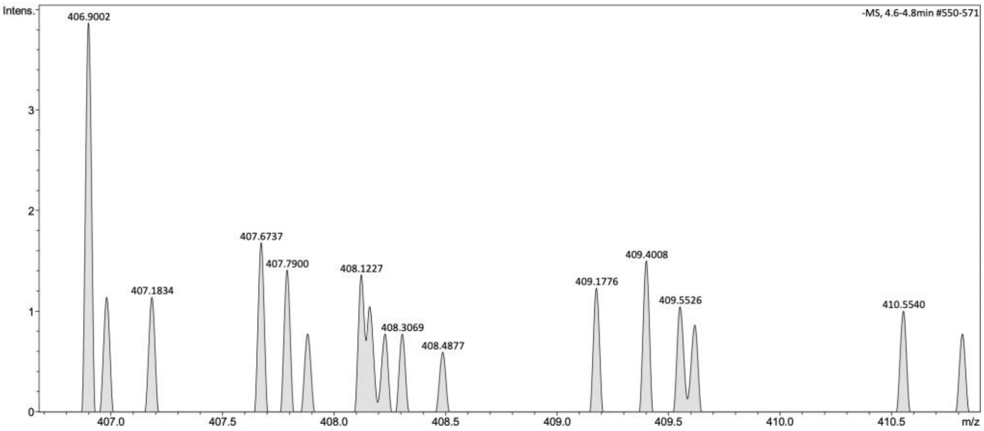

Figure S112 – A high-resolution mass spectrum (ESI$^-$) obtained for **6** in methanol, *m/z* [M]$^-$.

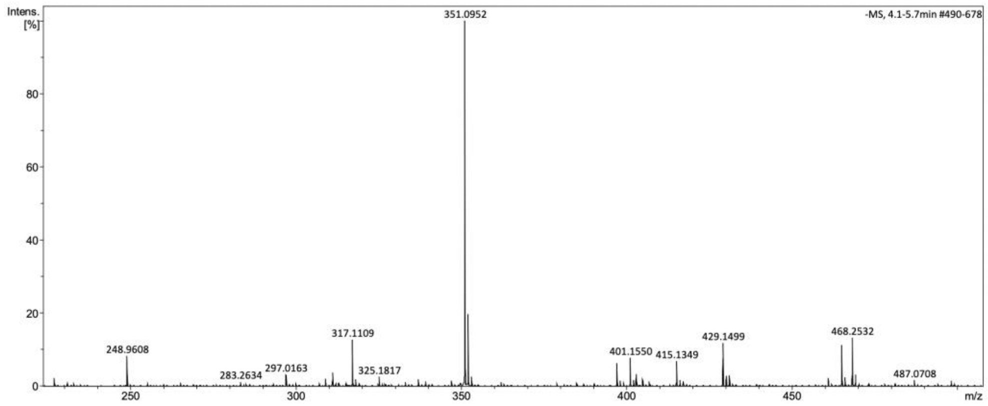

Figure S113 - A high-resolution mass spectrum (ESI$^-$) obtained for **7** in methanol, *m/z* [M]$^-$.

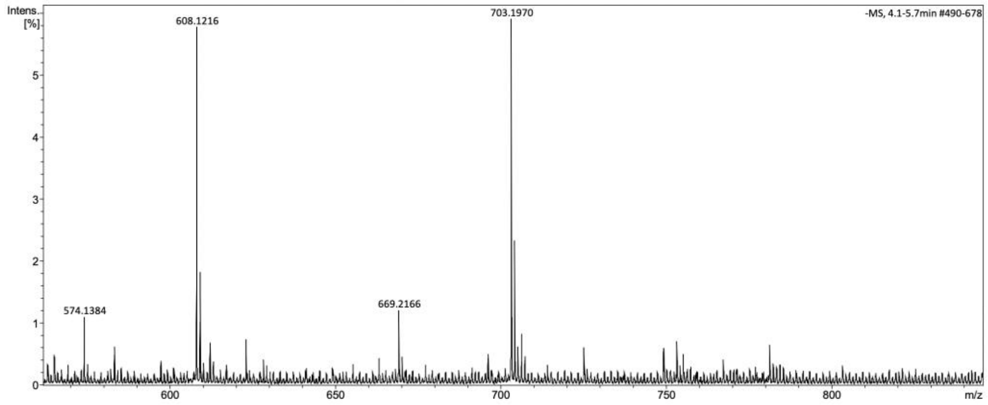

Figure S114 - A high-resolution mass spectrum (ESI$^-$) obtained for dimeric species of **7** in methanol, *m/z* [M + M + H]$^-$.

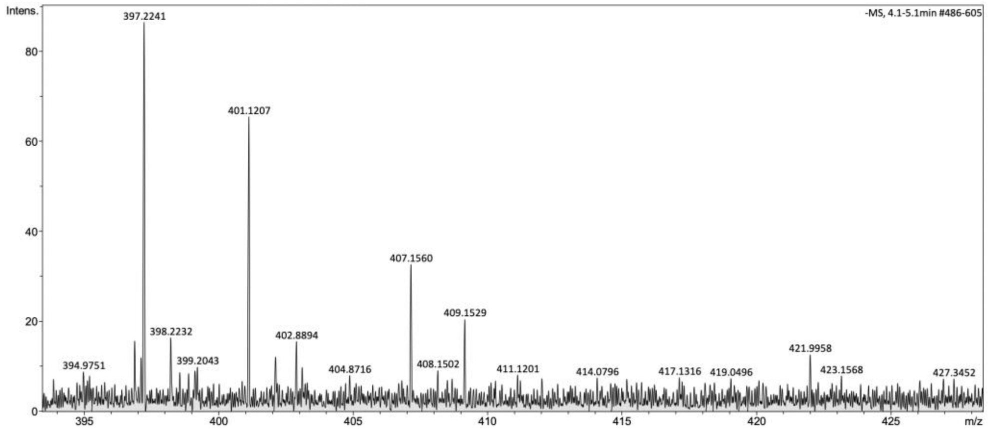

Figure S115 - A high-resolution mass spectrum (ESI$^-$) obtained for **8** in methanol, *m/z* [M]$^-$.

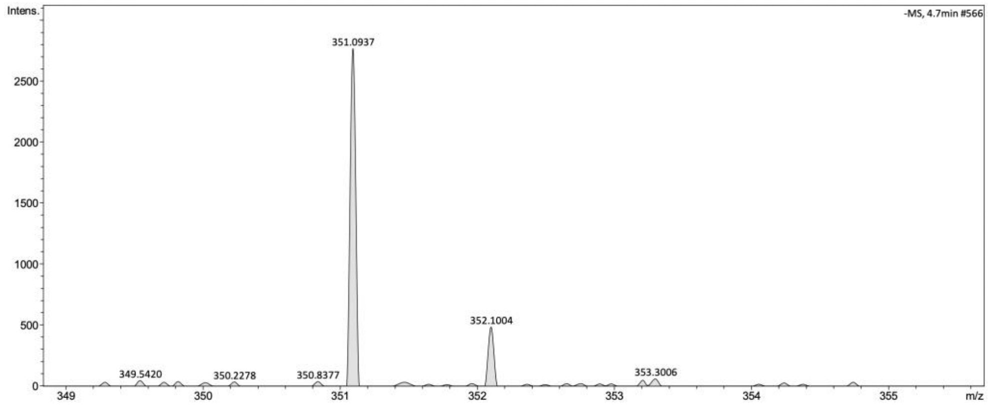

Figure S116 - A high-resolution mass spectrum (ESI$^-$) obtained for **9** in methanol, *m/z* [M]$^-$.

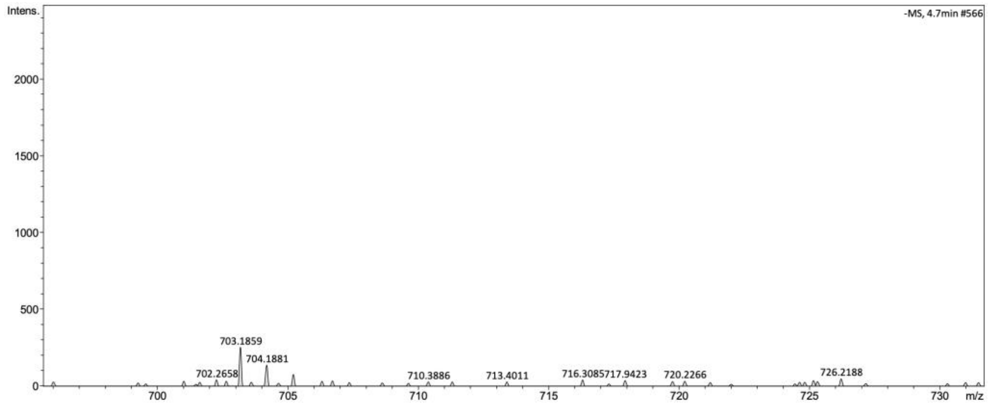

Figure S117 - A high-resolution mass spectrum (ESI$^-$) obtained for dimeric species of **9** in methanol, *m/z* [M + M + H]$^-$.

Summary

Table S12 – Summary of high-resolution electrospray ionisation mass spectrometry (ESI-MS) theoretical and experimentally derived values for compounds **2** - **9**.

	m/z [M]$^-$		m/z [M + M + H]$^-$	
**Compound**	**Theoretical**	**Actual**	**Theoretical**	**Actual**
**2**	374.1817	374.1739	*a*	*a*
**3**	317.1118	317.1110	635.2308	635.2271
**4**	374.1817	374.1763	*a*	*a*
**5**	317.1118	317.1160	635.2308	635.2408
**6**	408.1661	408.1227	*a*	*a*
**7**	351.0962	351.0952	703.1996	703.1970
**8**	408.1661	408.1502	*a*	*a*
**9**	351.0962	351.0937	703.1996	703.1859

a = not applicable.

Section S15: Circular dichroism studies

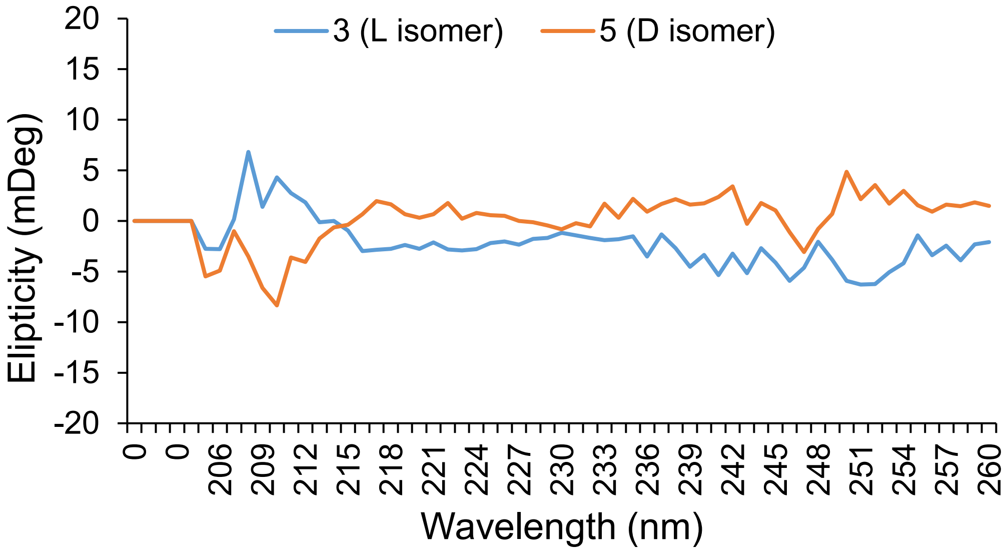

Figure S118 - The circular dichroism spectrum for **3** and **5** at 1 mM in H_2O/ 5 % EtOH.

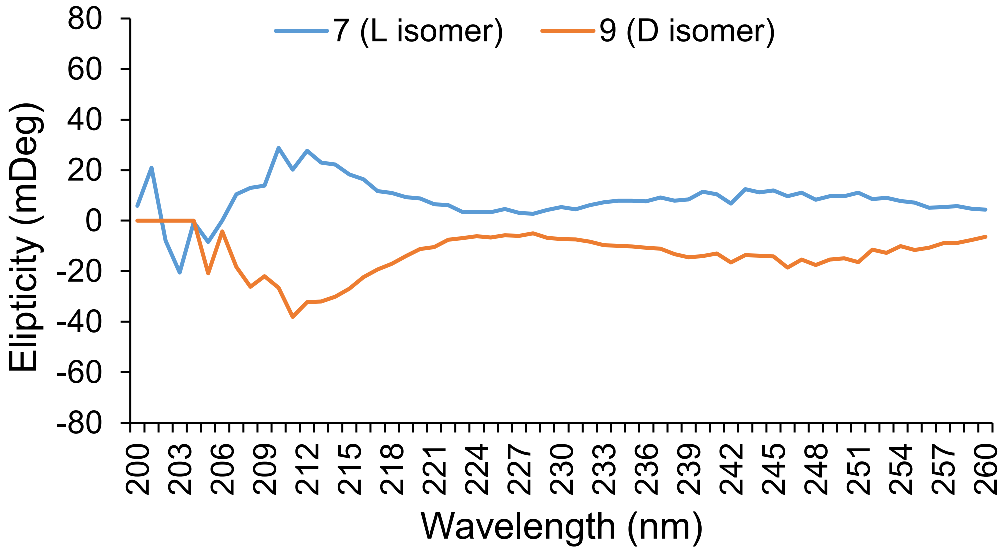

Figure S119 - The circular dichroism spectrum for **7** and **9** at 1 mM in H_2O/ 5 % EtOH.

Section S16: Proton NMR spectroscopy titration studies

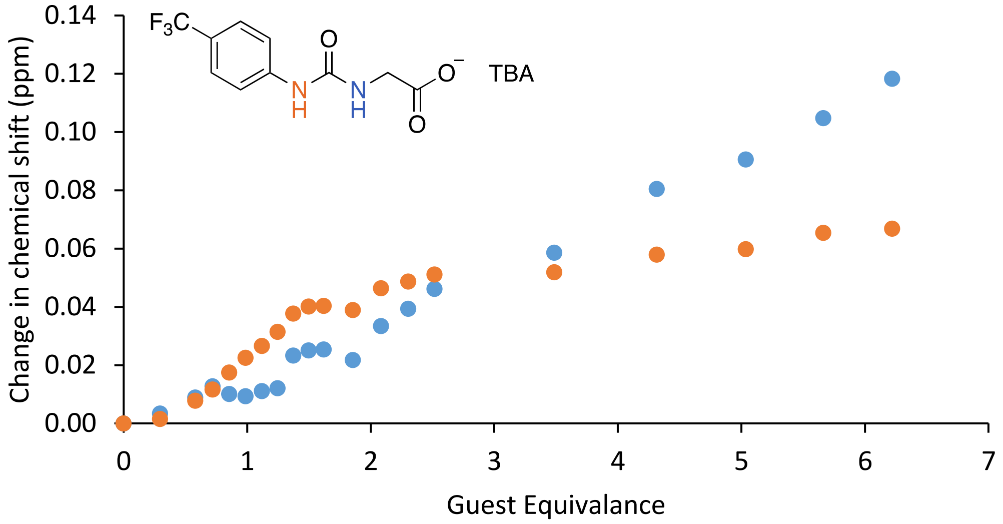

Figure S120 - Graph illustrating the ^{1}H NMR down-field change in chemical shift of urea NH resonances for **1** with increasing concentration of TBACl in DMSO-d_6/0.5 % H_2O (298 K).

Association constant calculation: values were calculated from data gathered from the urea NH resonances in **1** and fit to a 1:1 binding isotherm (Nelder-Mead fit).

K_a = 4.33 M^{-1} ± 6.19 %
http://app.supramolecular.org/bindfit/view/fa9845e4-e26e-4a1b-80d3-03ea3aeb2b5a

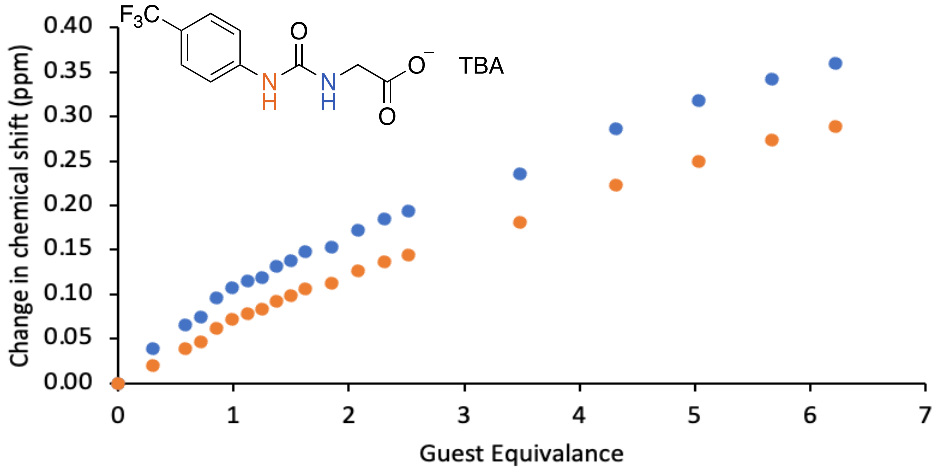

Figure S121 - Graph illustrating the ^{1}H NMR down-field change in chemical shift of urea NH resonances for **1** with increasing concentration of $TBAH_2PO_4$ in DMSO-d_6/0.5 % H_2O (298 K).

Association constant calculation: values were calculated from data gathered from the urea NH resonances in **1** and fit to a 1:1 binding isotherm (Nelder-Mead fit).
K_a = 12.59 M^{-1} ± 1.77 %
http://app.supramolecular.org/bindfit/view/812372fc-44c7-4572-a273-30c4cd88eb35

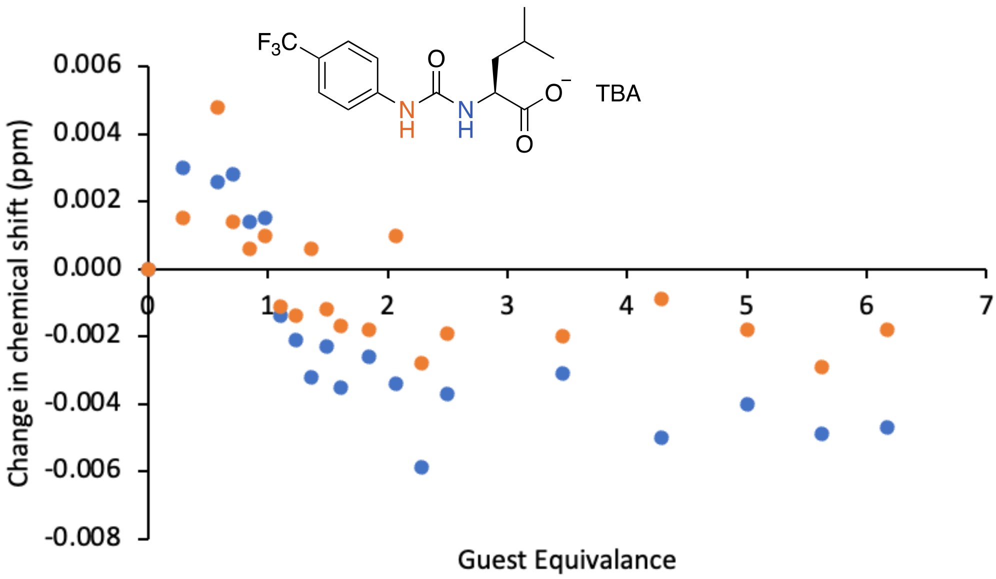

Figure S122 - Graph illustrating the ^{1}H NMR down-field change in chemical shift of urea NH resonances for **3** with increasing concentration of TBACl in DMSO-d_6/0.5 % H_2O (298 K).

Association constant calculation: values were calculated from data gathered from the urea NH resonances in **3**. The data could not be fitted as it is below the limit of detection of the NMR machine.

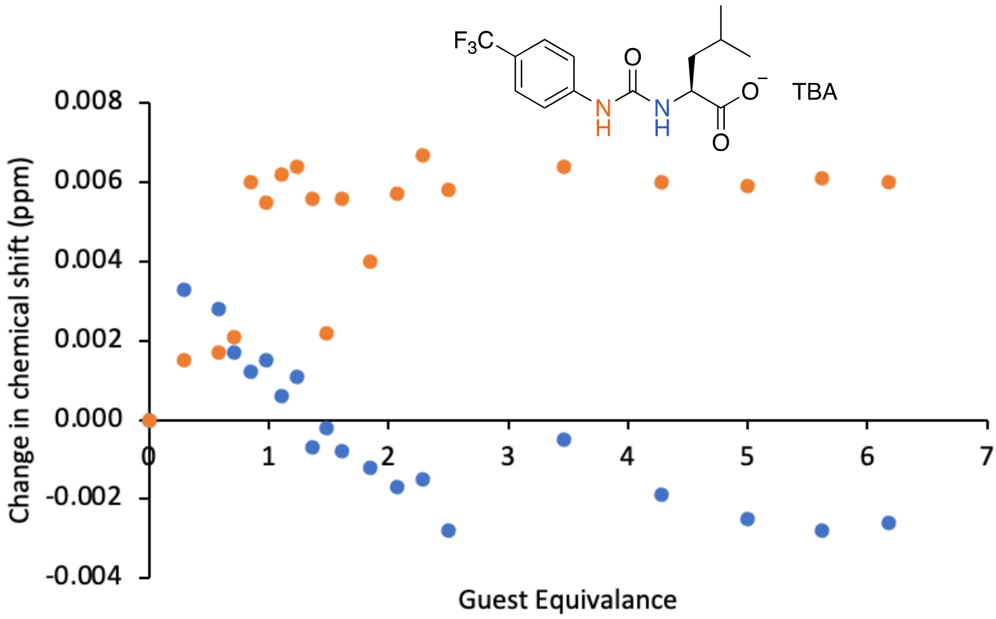

Figure S123 - Graph illustrating the ^{1}H NMR down-field change in chemical shift of urea NH resonances for **3** with increasing concentration of $TBAH_2PO_4$ in DMSO-d_6/0.5 % H_2O (298 K).

Association constant calculation: values were calculated from data gathered from the urea NH resonances in **3**. The data could not be fitted as the change in chemical shift is less than 0.01 ppm.

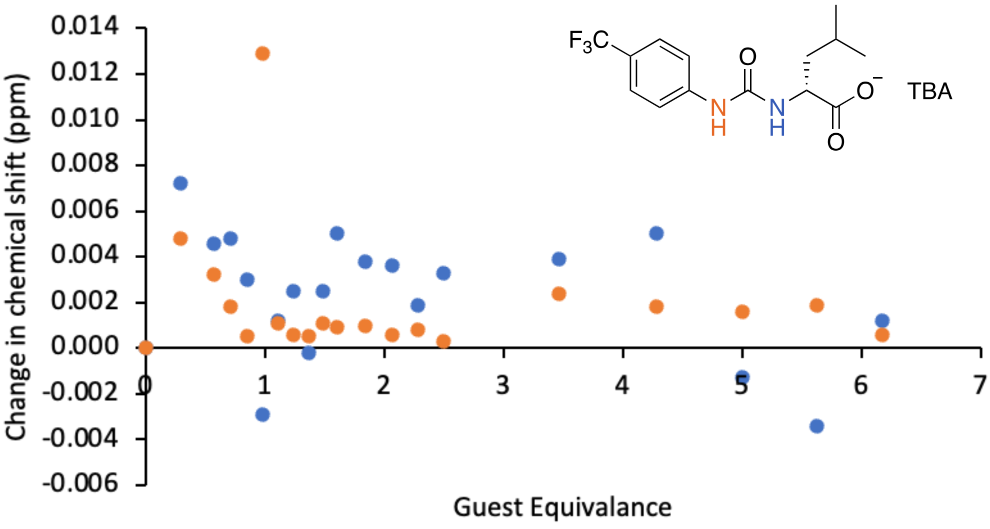

Figure S124 - Graph illustrating the ^{1}H NMR down-field change in chemical shift of urea NH resonances for **5** with increasing concentration of TBACl in DMSO-d_6/0.5 % H_2O (298 K).

Association constant calculation: values were calculated from data gathered from the urea NH resonances in **5**. The data could not be fitted as the overall change in chemical shift is less than 0.01 ppm.

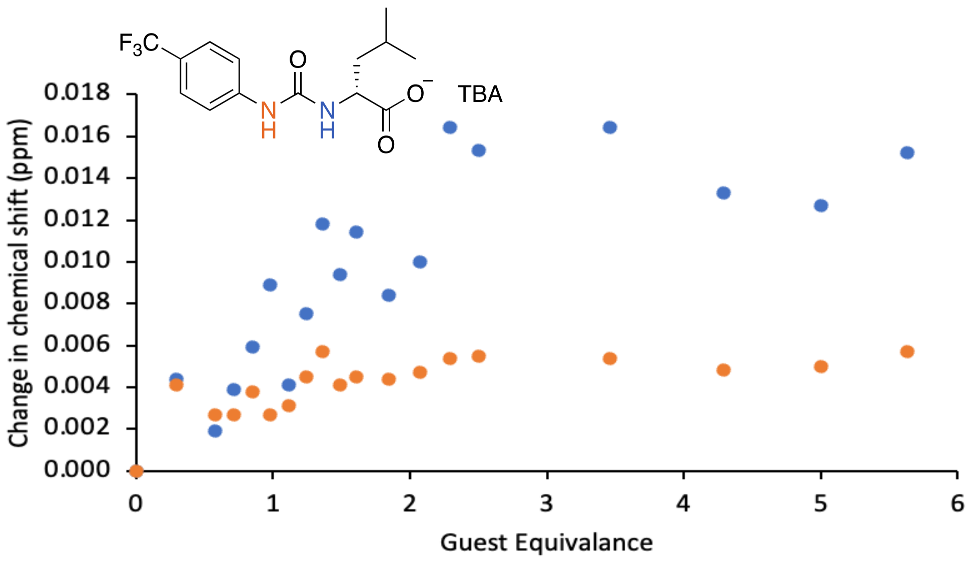

Figure S125 - Graph illustrating the ^{1}H NMR down-field change in chemical shift of urea NH resonances for **5** with increasing concentration of $TBAH_2PO_4$ in DMSO-d_6/0.5 % H_2O (298 K).

Association constant calculation: values were calculated from data gathered from only one urea NH resonance in **5**, as the other NH resonance had change in chemical shift is less than 0.01 ppm. The data was fit to a 1:1 binding isotherm (Nelder-Mead fit) but no association constant is reported for the formation of any complex due to the 40 % error calculated for the fit of these data.

http://app.supramolecular.org/bindfit/view/48194597-20be-4f60-9019-b49603589fcb

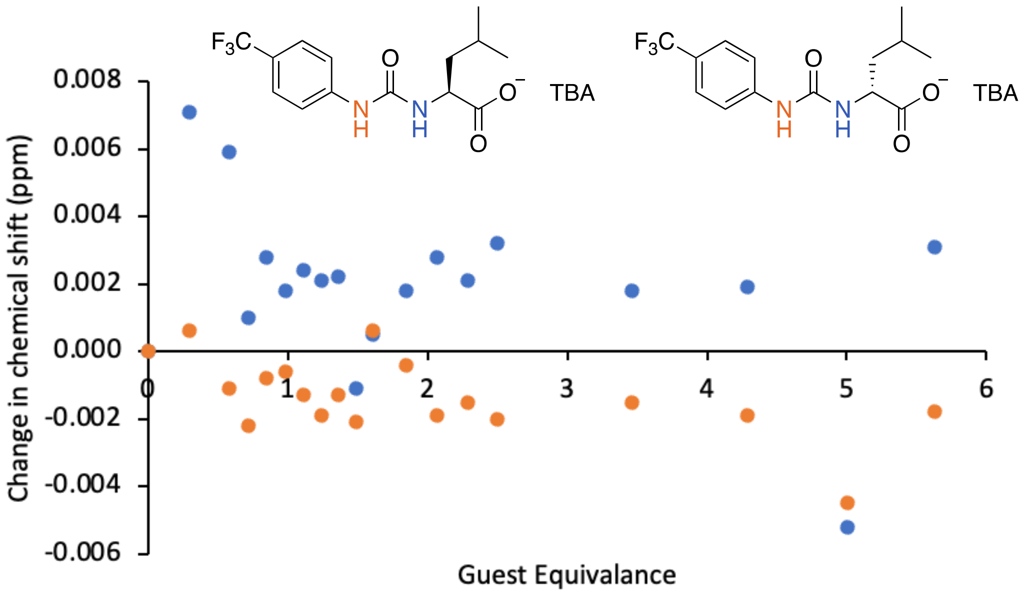

Figure S126 - Graph illustrating the ^{1}H NMR down-field change in chemical shift of urea NH resonances for a 1:1 enantiomeric mixture of **3** + **5** with increasing concentration of TBACl in DMSO-d_6/0.5 % H_2O (298 K).

Association constant calculation: values were calculated from data gathered from the urea NH resonances in **3** and **5**. The data could not be fitted as the change in chemical shift is less than 0.01 ppm.

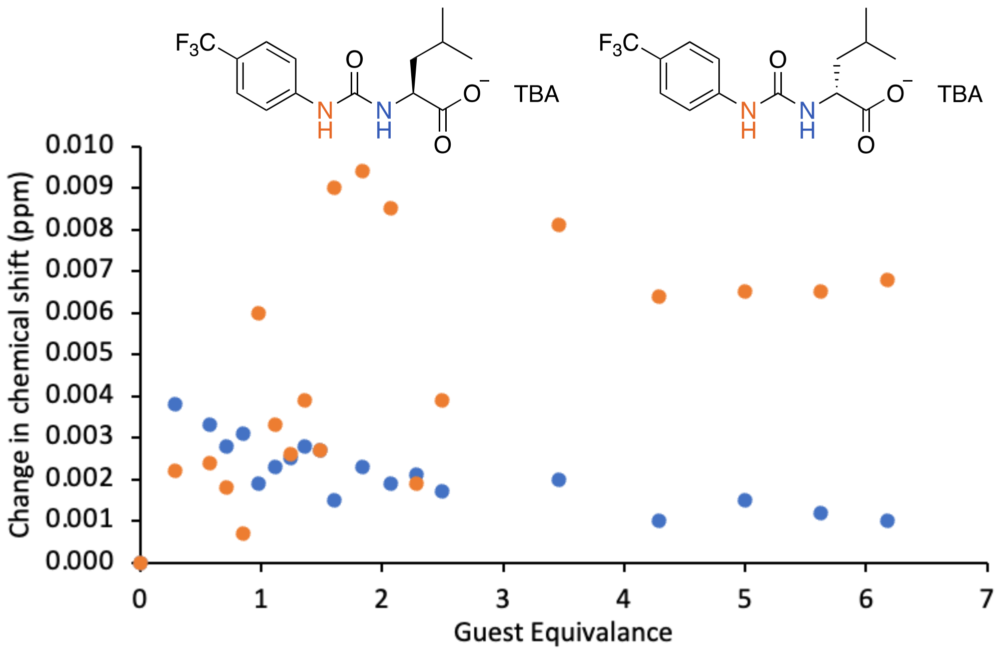

Figure S127 - Graph illustrating the ^{1}H NMR down-field change in chemical shift of urea NH resonances for a 1:1 enantiomeric mixture of **3** + **5** with increasing concentration of $TBAH_2PO_4$ in DMSO-d_6/0.5 % H_2O (298 K).

Association constant calculation: values were calculated from data gathered from the urea NH resonances in **3** and **5**. The data could not be fitted as the change in chemical shift is less than 0.01 ppm.

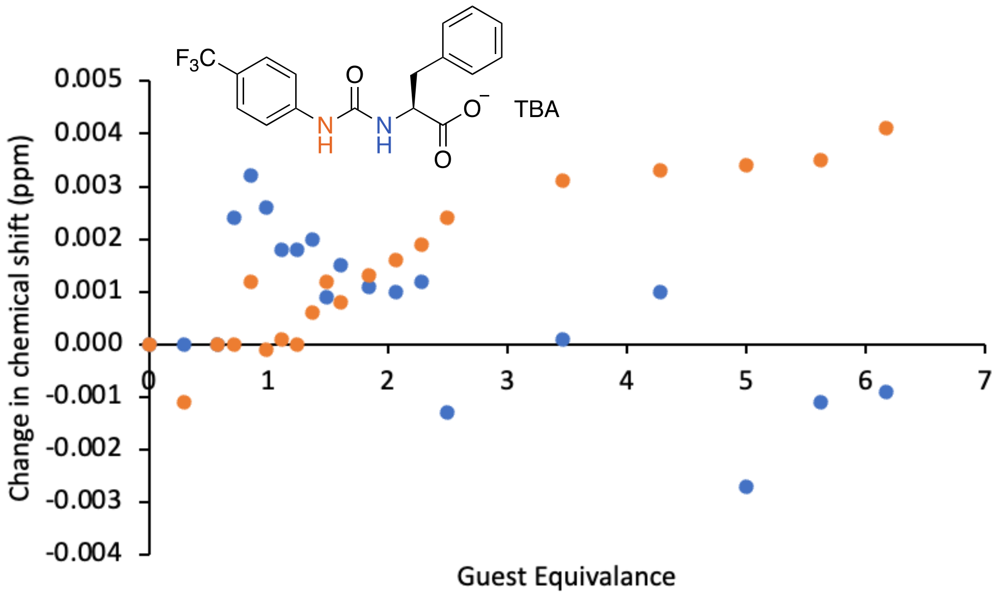

Figure S128 - Graph illustrating the ^{1}H NMR down-field change in chemical shift of urea NH resonances for **7** with increasing concentration of TBACl in DMSO-d_6/0.5 % H_2O (298 K).

Association constant calculation: values were calculated from data gathered from the urea NH resonances in **7**. The data could not be fitted as it is below the limit of detection of the NMR machine.

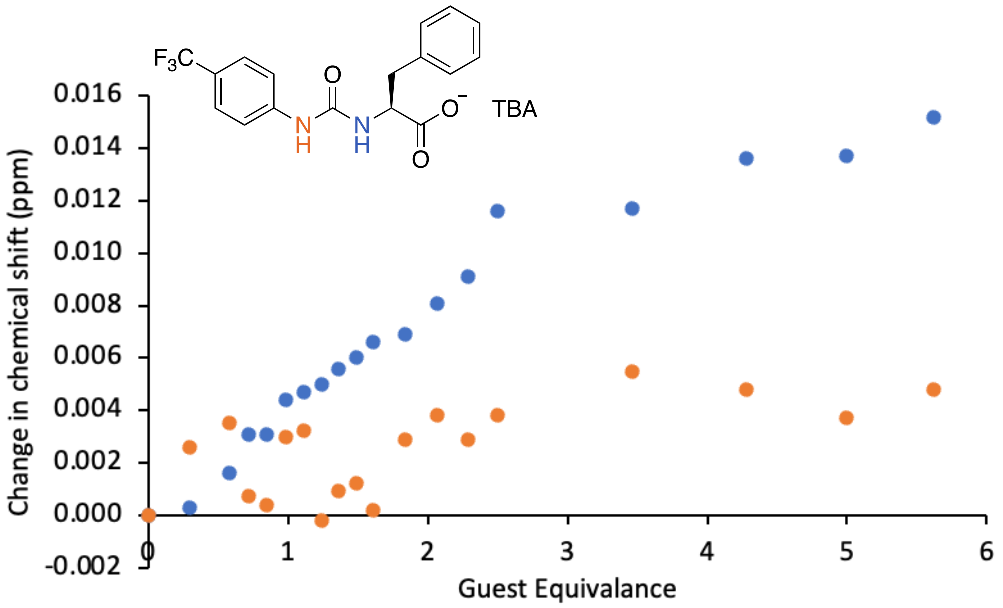

Figure S129 - Graph illustrating the ^{1}H NMR down-field change in chemical shift of urea NH resonances for **7** with increasing concentration of $TBAH_2PO_4$ in DMSO-d_6/0.5 % H_2O (298 K).

Association constant calculation: values were calculated from data gathered from only one urea NH resonance in **7**, as the other NH has change in chemical shift is less than 0.01 ppm. The data was fit to a 1:1 binding isotherm (Nelder-Mead fit):

K_a = 30.27 M^{-1} ± 7.50 %
http://app.supramolecular.org/bindfit/view/b7f02191-544b-4a16-9268-e2474ada3bdf

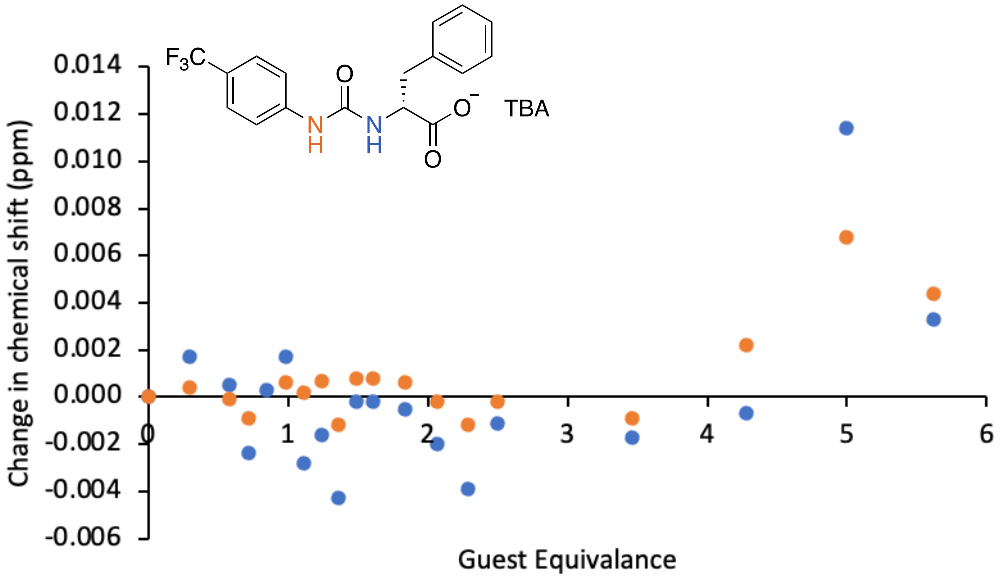

Figure S130 - Graph illustrating the ^{1}H NMR down-field change in chemical shift of urea NH resonances for **9** with increasing concentration of TBACl in DMSO-d_6/0.5 % H_2O (298 K).

Association constant calculation: values were calculated from data gathered from both urea NH resonances in **9**. The data could not be fitted as it is below the limit of detection of the NMR machine.

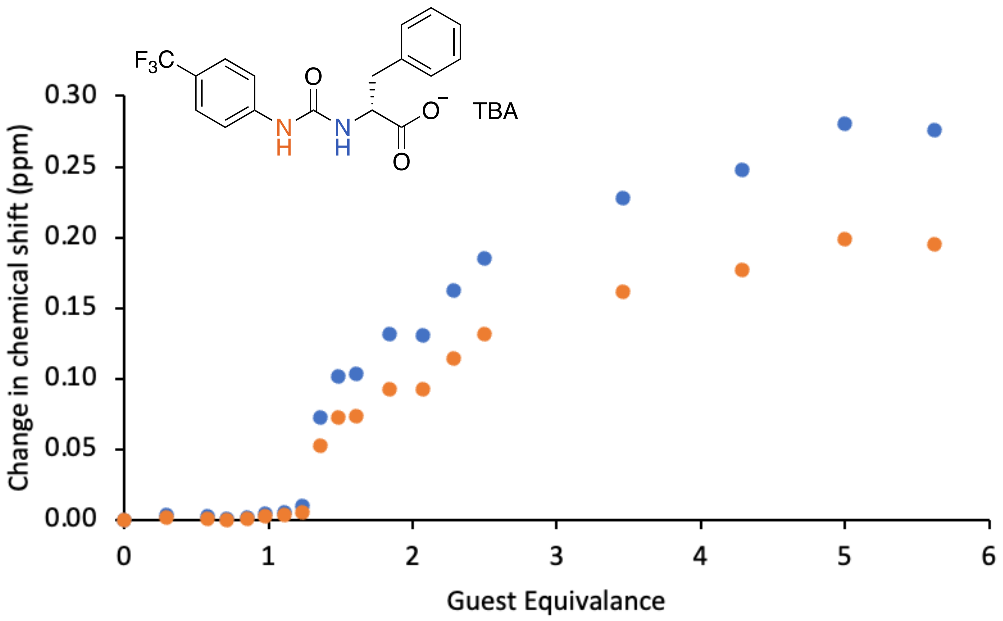

Figure S131 - Graph illustrating the ^{1}H NMR down-field change in chemical shift of urea NH resonances for **9** with increasing concentration of $TBAH_2PO_4$ in DMSO-d_6/0.5 % H_2O (298 K).

Association constant calculation: values were calculated from data gathered from both urea NH resonances in **9** and fit to a 1:1 binding isotherm (Nelder-Mead fit): http://app.supramolecular.org/bindfit/view/14610503-0cd1-4c7c-945c-da1bf465e2dd

The data was then fit to a 2:1 (guest:host) binding isotherm (Nelder-Mead fit): http://app.supramolecular.org/bindfit/view/d1a599b4-ee96-4019-b8f9-6f6c64fd1c34

No association constants are reported for the formation of any complex due to the high error calculated for the fit of these data, or the trend of these data failing to fit any ideal predicted trends.

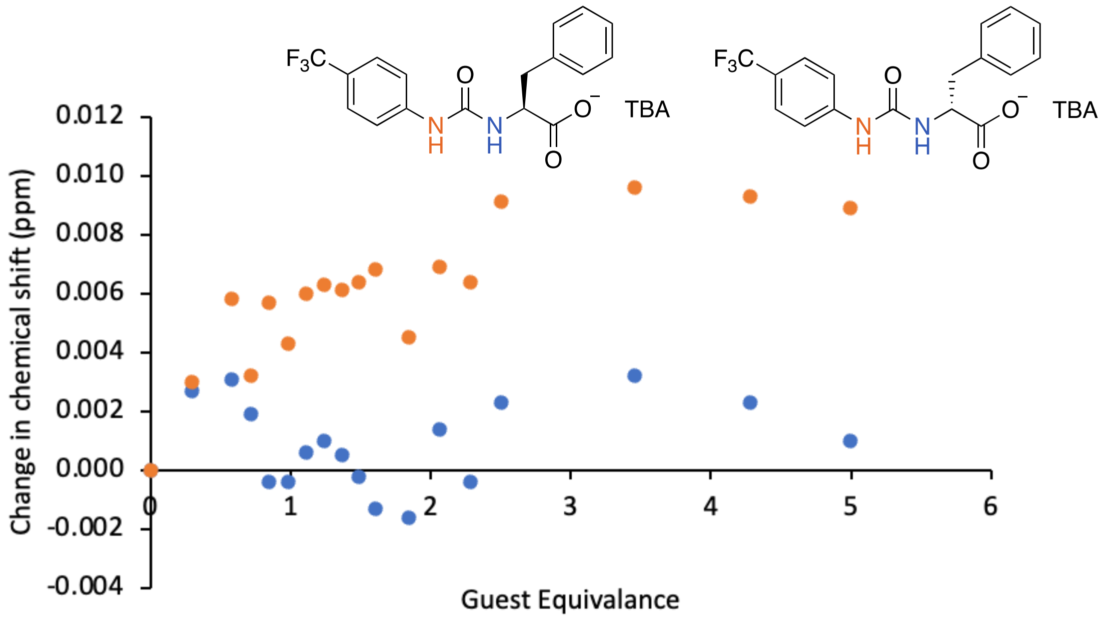

Figure S132 - Graph illustrating the ^{1}H NMR down-field change in chemical shift of urea NH resonances for a 1:1 enantiomeric mixture of **7** + **9** with increasing concentration of TBACl in DMSO-d_6/0.5 % H_2O (298 K).

Association constant calculation: values were calculated from data gathered from both urea NH resonances in **7** and **9**. The data could not be fitted as the change in chemical shift is less than 0.01 ppm.

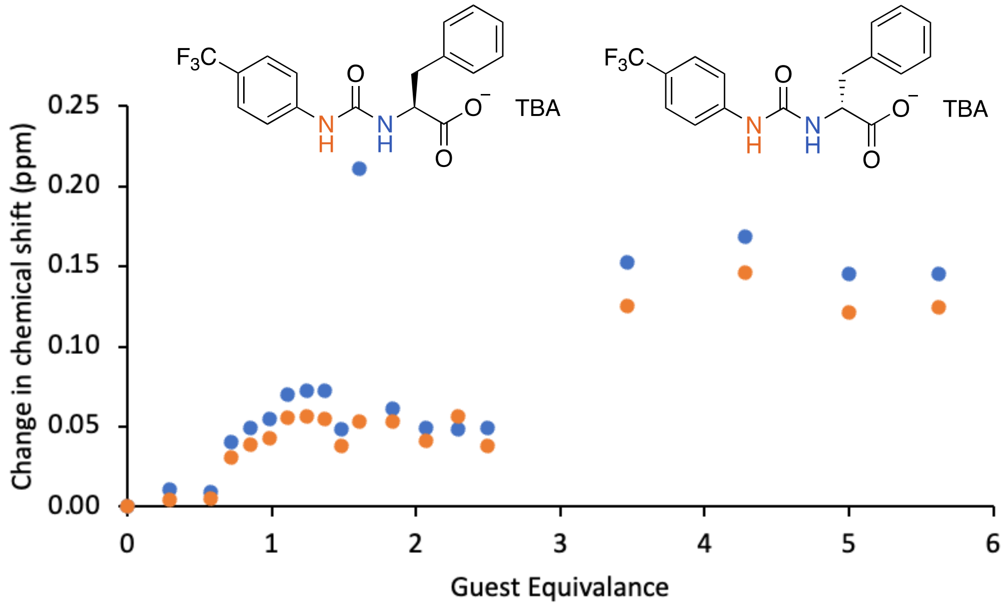

Figure S133 - Graph illustrating the ^{1}H NMR down-field change in chemical shift of urea NH resonances for a 1:1 enantiomeric mixture of **7** + **9** with increasing concentration of $TBAH_2PO_4$ in DMSO-d_6/0.5 % H_2O (298 K).

Association constant calculation: values were calculated from data gathered from both urea NH resonances in **7** and **9** and fit to a 1:1 binding isotherm (Nelder-Mead fit): http://app.supramolecular.org/bindfit/view/e8f619f2-545b-4a4d-9a97-f74c5df1a603

The data was then fit to a 2:1 (guest:host) binding isotherm (Nelder-Mead fit): http://app.supramolecular.org/bindfit/view/b8b35088-97ef-4f84-abf0-91d82174e736

No association constants are reported for the formation of any complex due to the high error calculated for the fit of these data, or the trend of these data failing to fit any ideal predicted trends.

Summary

Table S13 - Summary of association constants (K_a) calculated form ^{1}H NMR titration data, using Bindfit v0.5,[1] conducted in DMSO-d_6/0.5 % H_2O at 298 K. Anionic guest species were supplied as the TBA salt. Data was fitted to both 1:1, 1:2 and 2:1 binding isotherm. Comparative error analysis confirmed the 1:1 binding isotherm to demonstrate the highest level of confidence when fitted to these data, unless otherwise indicated.

Host	Guest	K_a (M^{-1})	Error (± %)
**1**	Cl^-	4.33	6.19
**1**	$H_2PO_4^-$	12.59	1.77
**3**	Cl^-	*b*	*b*
**3**	$H_2PO_4^-$	*b*	*b*
**5**	Cl^-	*b*	*b*
**5**	$H_2PO_4^-$	*c*	*c*
**3+5** [a]	Cl^-	*b*	*b*
**3+5** [a]	$H_2PO_4^-$	*b*	*b*
**7**	Cl^-	*b*	*b*
**7**	$H_2PO_4^-$	30.27	7.50
**9**	Cl^-	*b*	*b*
**9**	$H_2PO_4^-$	c	c
**7+9** [a]	Cl^-	*b*	*b*
**7+9** [a]	$H_2PO_4^-$	*c*	*c*

a – 1:1 mixture.

b – No evidence of host: guest complex formation detected.

c – Data could not be fitted with a high degree of confidence to any binding model (error > ± 15 %).

Section S17: Ion transport studies

Cl^-/NO_3^- antiport Hill plots – pH 7.2

Internal buffer: 500 mM NaCl buffered to pH 7.2 with 5 mM sodium phosphate salts.

External buffer: 500 mM $NaNO_3$ buffered to pH 7.2 with 5 mM sodium phosphate salts.

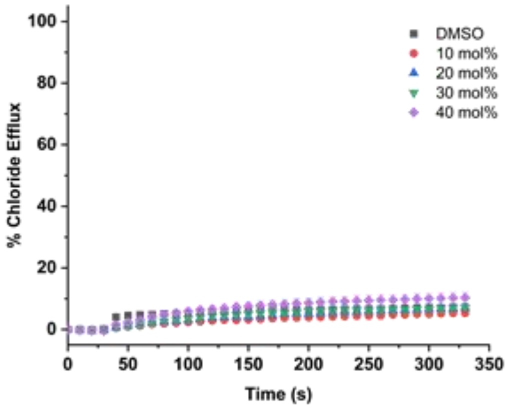

Figure S134 - Transport data for the Cl^-/NO_3^- antiport Hill plot experiment with compound **1**. Full Hill analysis was not performed due to the low observed activity of this compound.

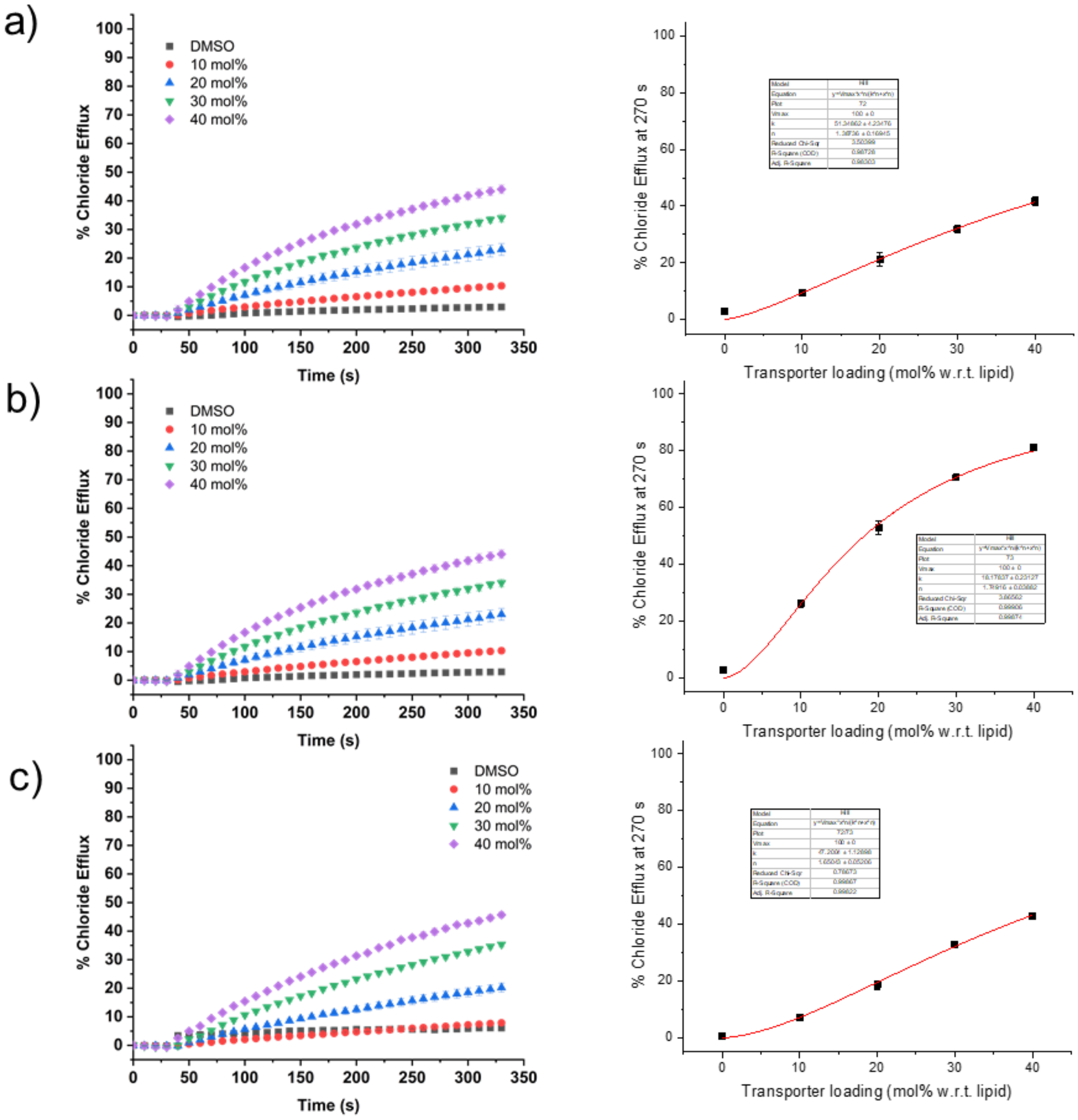

Figure S135 - Transport data and Hill analyses for the Cl^-/NO_3^- antiport experiment with compounds: a) **3**, b) **5** and c) a racemic mixture of **3** and **5**.

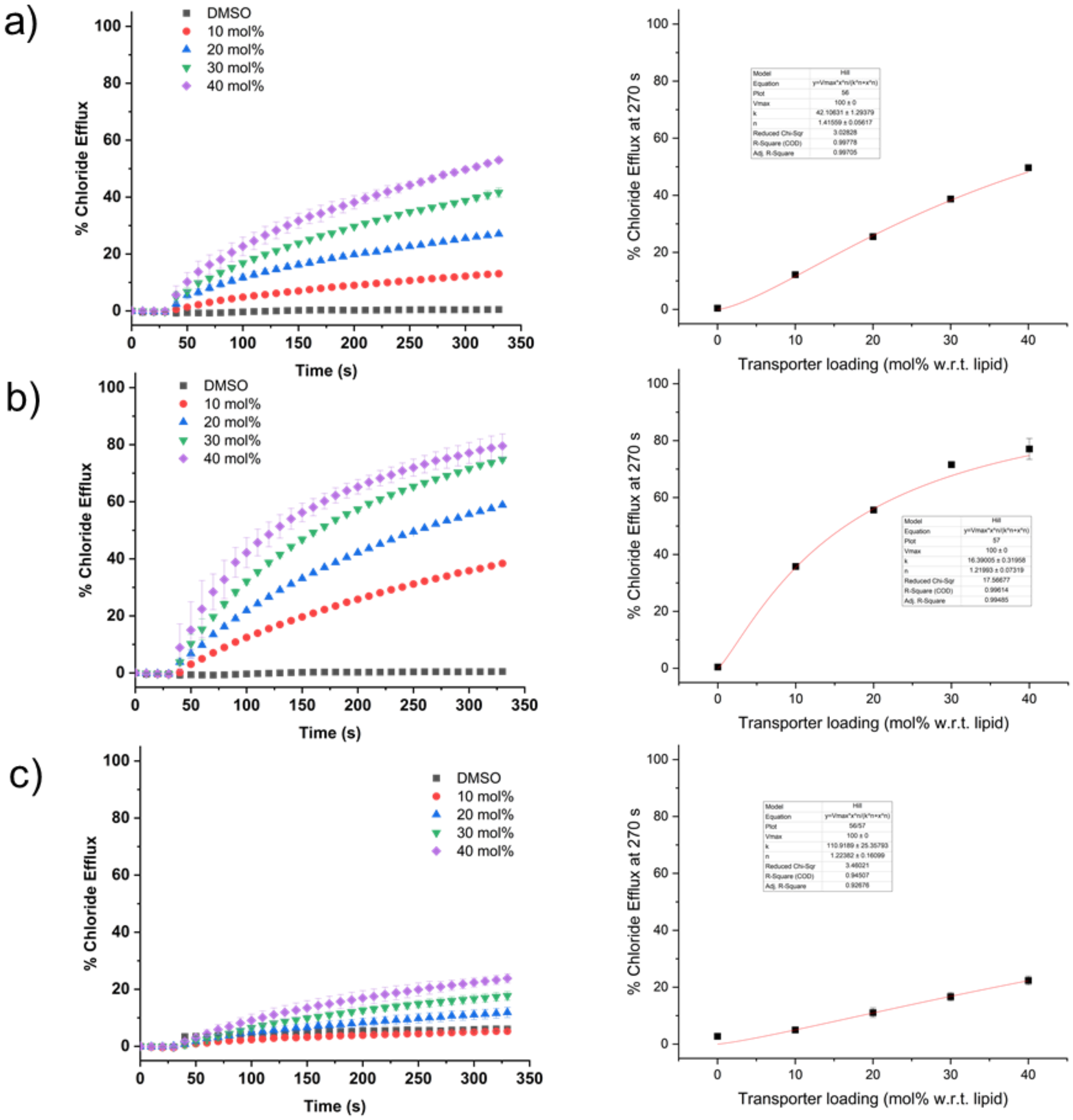

Figure S136 - Transport data and Hill analyses for the Cl^-/NO_3^- antiport experiment with compounds: a) **7**, b) **9** and c) a racemic mixture of **7** and **9**.

Control experiments

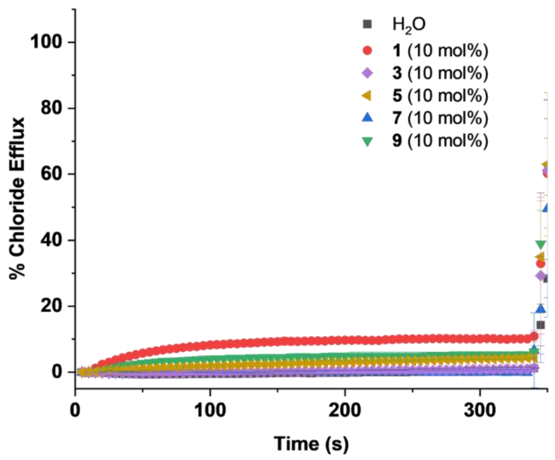

Figure S137 - Cl^-/NO_3^- antiport experiment with compounds **1**, **3**, **5**, **7** and **9** (10 mol%) added from a stock solution in H_2O (5 mM).

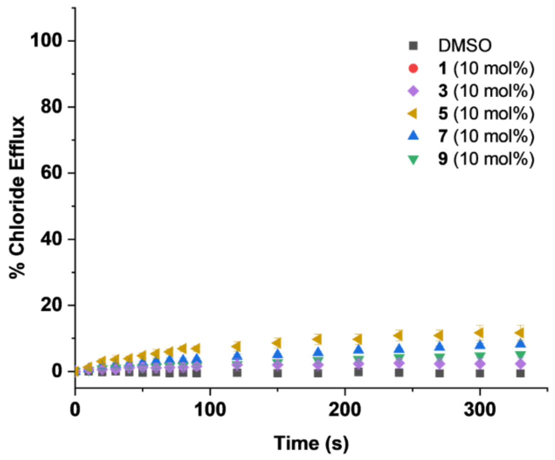

Figure S138 - Chloride efflux mediated by compounds **1**, **3**, **5**, **7** and **9** (10 mol%) from a stock solution in DMSO (5 mM). The transporter was added to vesicles containing 489 mM NaCl buffered to pH 7.2 with 5 mM sodium phosphate salts, which were suspended in 167 mM Na_2SO_4 buffered to pH 7.2 with 5 mM sodium phosphate salts.

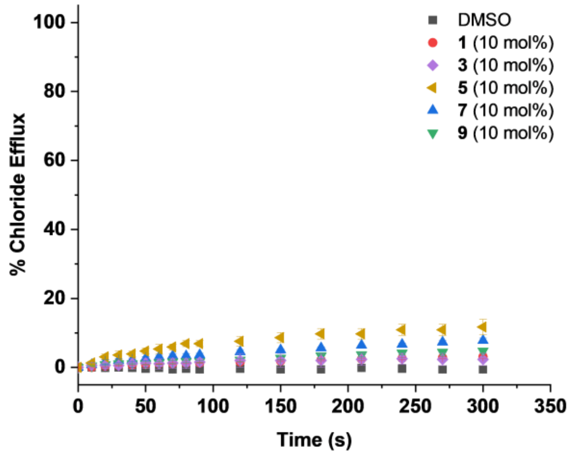

Figure S139 - Chloride efflux mediated by compounds **1**, **3**, **5**, **7** and **9** (10 mol%) from a stock solution in DMSO (5 mM). The transporter was added to vesicles containing 489 mM NaCl buffered to pH 4 with 5 mM sodium citrate salts, which were suspended in 167 mM Na_2SO_4 buffered to pH 7.2 with 5 mM sodium phosphate salts.

Summary

Table S14 – a summary of the results of the Hill analyses of compounds **1**, **3**, **5**, **7** and **9** in Cl^-/NO_3^- antiport experiments. EC_{50} is the effective concentration of transporter required to mediate 50% chloride efflux after 270 s; *n* is the Hill coefficient; n.d. = not determined due to the low observed activity of the transporter. Errors were derived from fitting the data using Origin 2019.

Compound	pH 7.2			
	EC_{50}	± Error	*n*	± Error
**1**	*b*	n.a.	*b*	n.a.
**3**	51.3	4.2	1.4	0.2
**5**	18.2	0.2	1.7	0.04
**3+5** [a]	47.2	1.1	1.7	0.05
**7**	42.1	1.3	1.4	0.06
**9**	16.4	0.3	1.3	0.07
**7+9** [a]	111	25.3	1.2	0.16

a – 1:1 mixture. *b* – not determined.

Section S18: MIC determination assay

Table S15 – The minimum inhibitory concentration (MIC) values determined for **1**, **3**, **5**, **7**, **9**, and 1:1 enantiomeric mixtures of **3** + **5** and **7** + **9** against 7 different Gram-positive *Staphylococcus aureus* (*S. aureus*), *Enterococcus faecalis* (*E. faecalis*) and *Enterococcus faecium* (*E. faecium*) bacteria strains. Here, the MIC is the lowest concentration at which no visible growth was observed.

MIC (mM)							
**Compound**	***S. aureus* 9144**	***S. aureus* 13616**	***S. aureus* USA 300**	***S. aureus* 1199B**	***E. Faecalis* NCTC 775**	***E. Faecalis* NCTC 12201**	***E. Faecium* NCTC 12204**
**1**	1.390	> 2.780	> 2.780	> 2.780	> 2.780	> 2.780	> 2.780
**3**	0.087	0.087	> 2.780	0.087	> 2.780	> 2.780	> 2.780
**5**	0.174	0.087	> 2.780	2.780	2.780	> 2.780	> 2.780
**3+5** [a]	0.174	0.087	0.174	0.174	> 2.780	> 2.780	> 2.780
**7**	0.087	0.087	0.087	0.174	> 2.780	> 2.780	> 2.780
**9**	0.174	0.087	0.087	0.174	2.780	> 2.780	> 2.780
**7+9** [a]	0.087	0.087	0.087	0.087 – 0.349	> 2.780	> 2.780	> 2.780

a – 1:1 mixture.

Section S19: GI_{50} determination assays

RPE-1 cell line

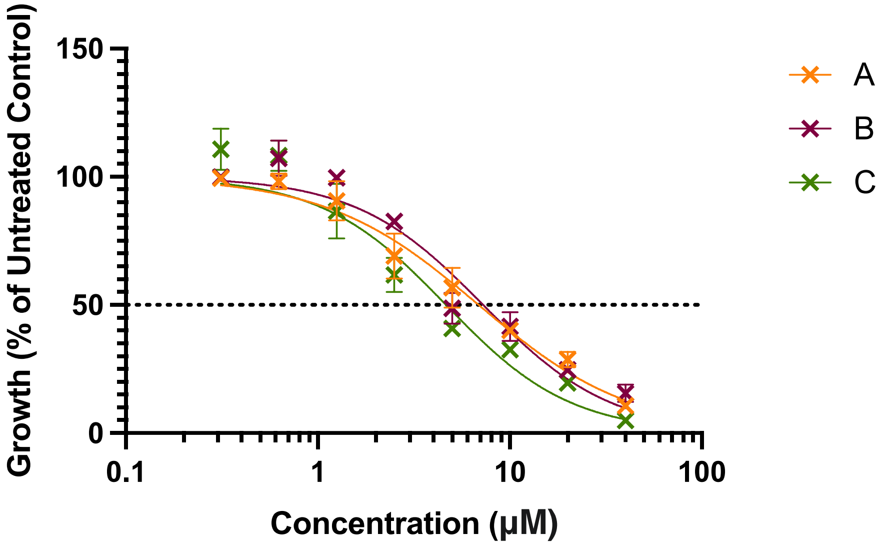

Figure S140 - Graph showing the effects of cisplatin on RPE-1 cells after a 96-hour incubation period followed by analysis as per SRB assay. The data shown represents three separate experiments: A = 14/11/2023; B = 16/11/2023; C = 21/11/2023.

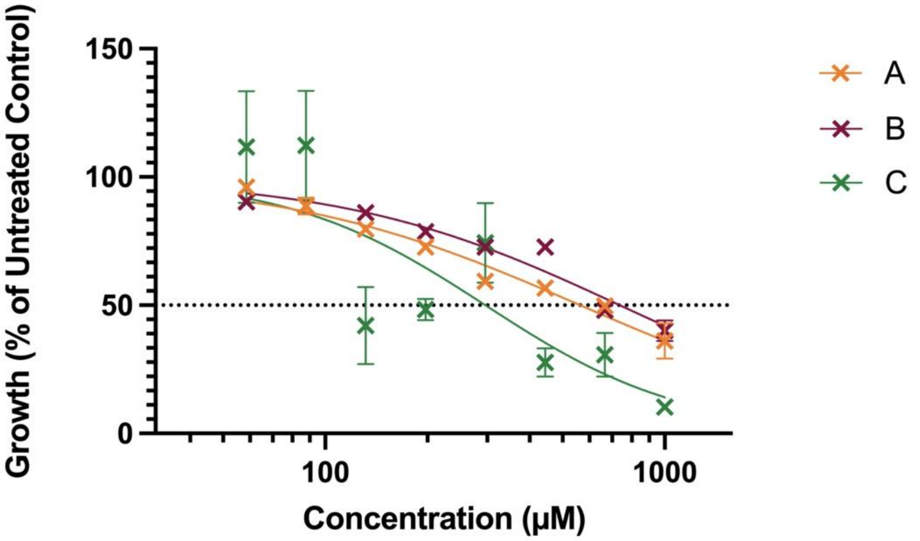

Figure S141 - Graph showing the effects of TBACl on RPE-1 cells after a 96-hour incubation period followed by analysis as per SRB assay. The data shown represents three separate experiments: A = 30/01/2022; B = 31/01/2022; C = 27/03/2024.

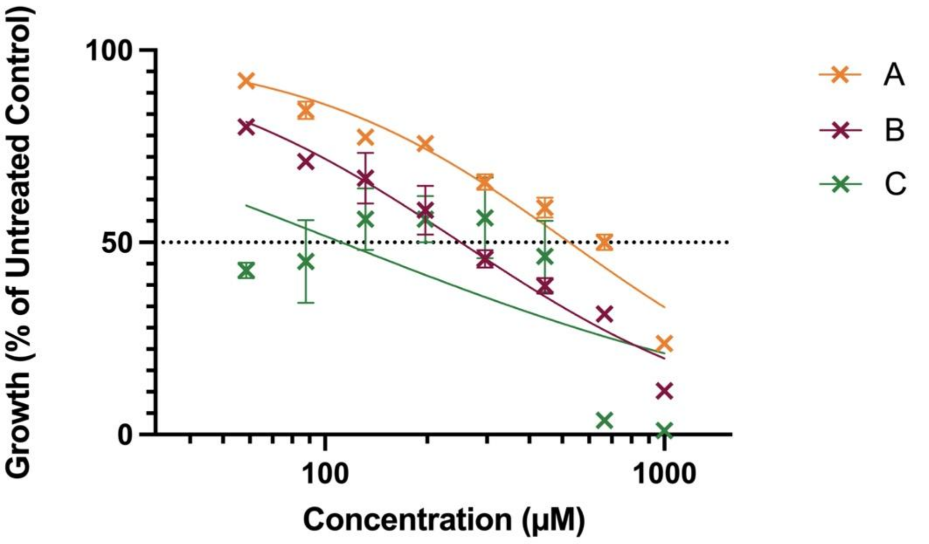

Figure S142 - Graph showing the effects of **1** on RPE-1 cells after a 96-hour incubation period followed by analysis as per SRB assay. The data shown represents three separate experiments: A = 30/01/2022; B= 31/01/2022; C = 27/03/2024.

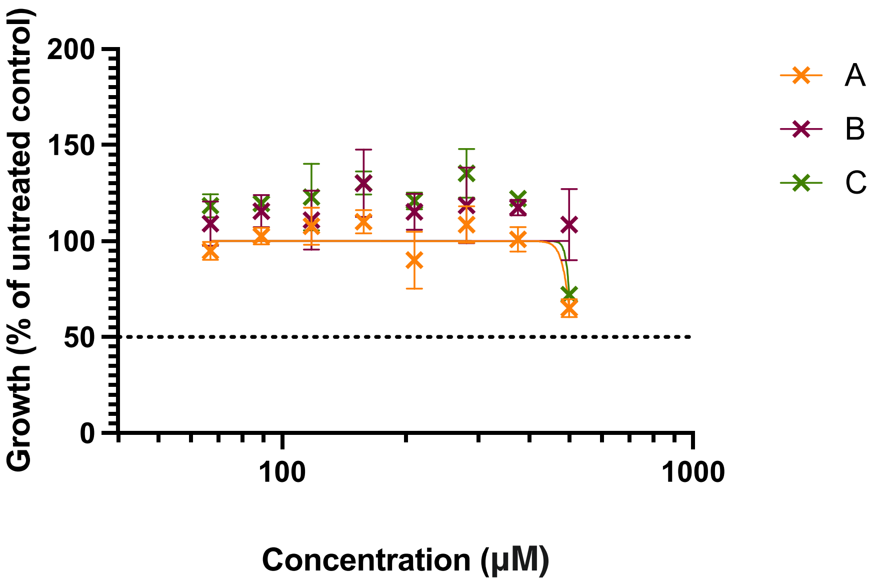

Figure S143 - Graph showing the effects of **3** on RPE-1 cells after a 96-hour incubation period followed by analysis as per SRB assay. The data shown represents three separate experiments: A = 14/11/2023; B = 16/11/2023; C = 21/11/2023.

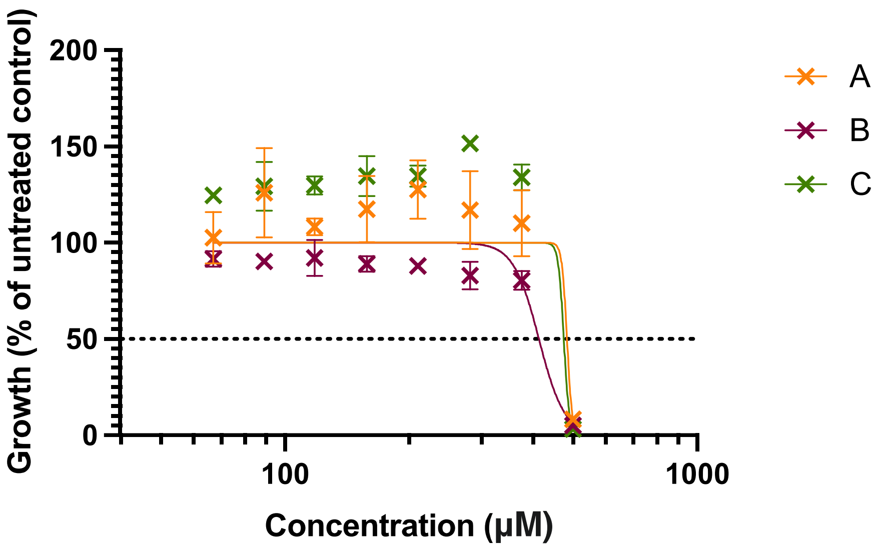

Figure S144 - Graph showing the effects of **5** on RPE-1 cells after a 96-hour incubation period followed by analysis as per SRB assay. The data shown represents three separate experiments: A = 14/11/2023; B = 16/11/2023; C = 21/11/2023.

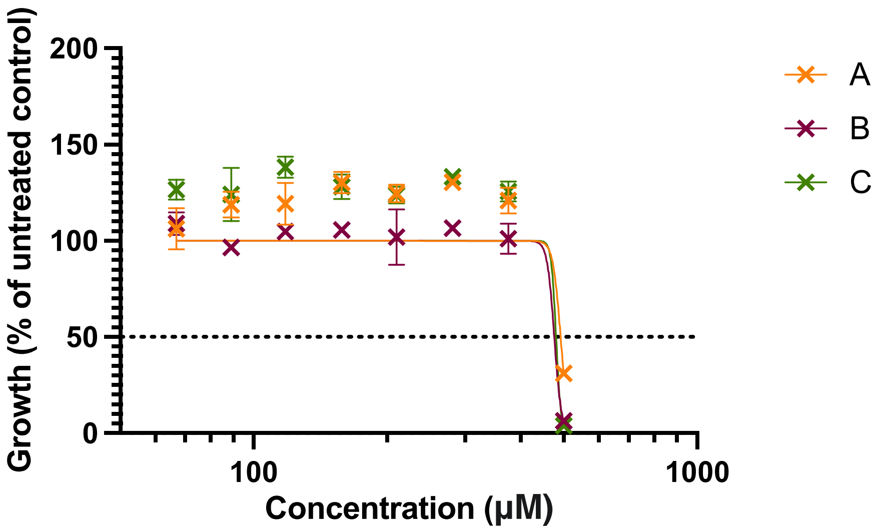

Figure S145 - Graph showing the effects of a 1:1 enantiomeric mixture of **3** + **5** on RPE-1 cells after a 96-hour incubation period followed by analysis as per SRB assay. The data shown represents three separate experiments: A = 14/11/2023; B = 16/11/2023; C = 21/11/2023.

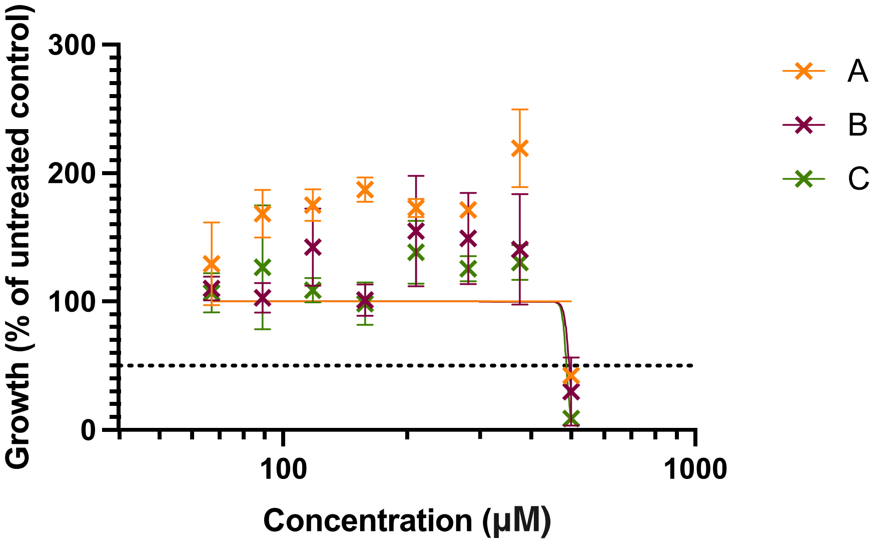

Figure S146 - Graph showing the effects of **7** on RPE-1 cells after a 96-hour incubation period followed by analysis as per SRB assay. The data shown represents three separate experiments: A = 14/11/2023; B = 16/11/2023; C = 21/11/2023.

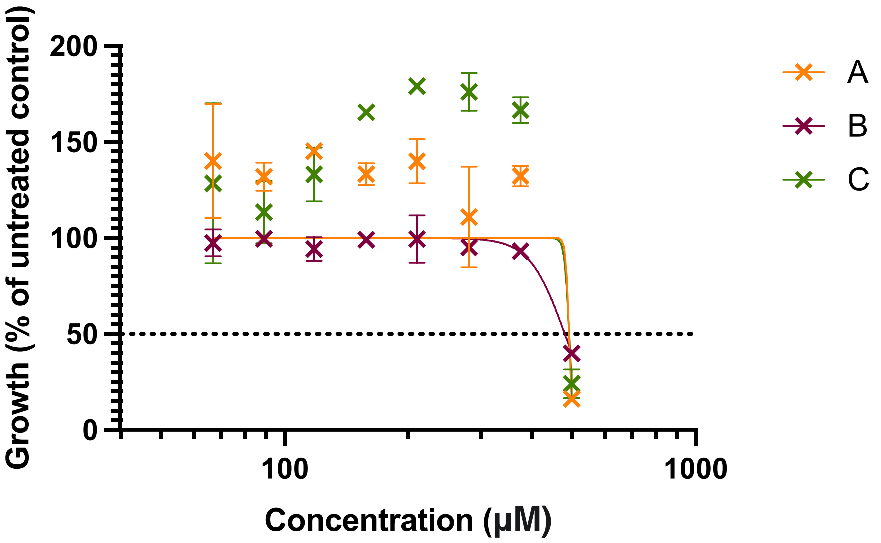

Figure S147 - Graph showing the effects of **9** on RPE-1 cells after a 96-hour incubation period followed by analysis as per SRB assay. The data shown represents three separate experiments: A = 14/11/2023; B = 16/11/2023; C = 21/11/2023.

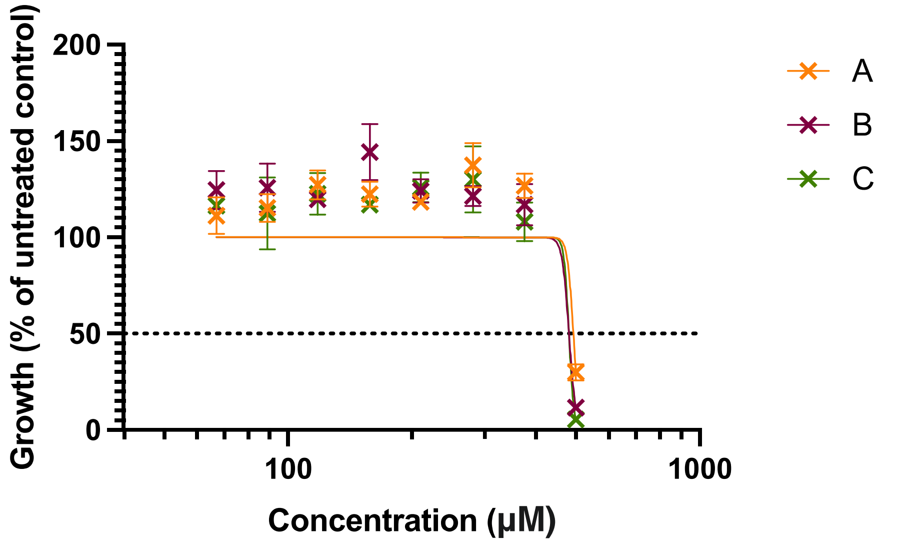

Figure S148 - Graph showing the effects of a 1:1 enantiomeric mixture of **7** + **9** on RPE-1 cells after a 96-hour incubation period followed by analysis as per SRB assay. The data shown represents three separate experiments: A = 14/11/2023; B = 16/11/2023; C = 21/11/2023.

A2780 cell line

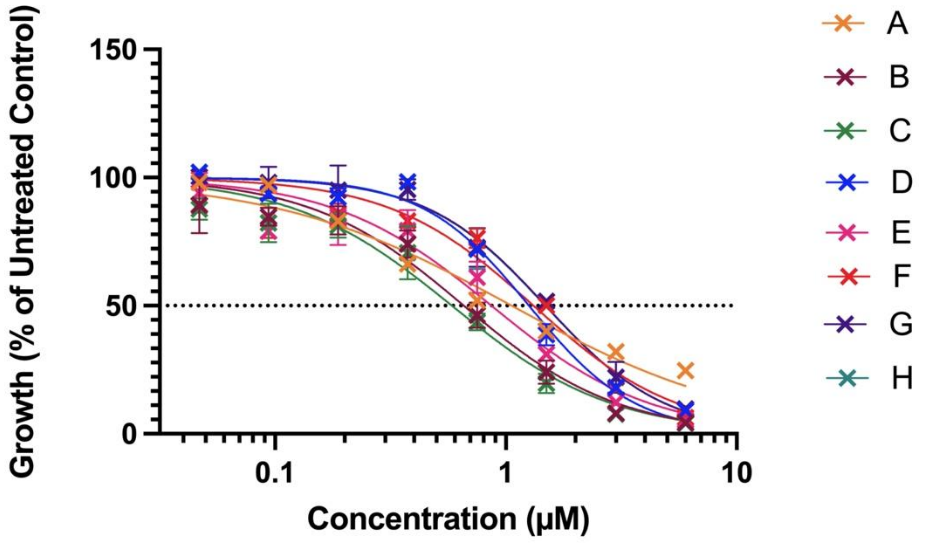

Figure S149 – Graph showing the effects of cisplatin on A2780 cells after a 96-hour incubation period followed by analysis as per SRB assay. The data shown represents eight separate experiments: A = 28/11/2021; B = 07/06/2022; C = 08/06/2022; D = 20/01/2023; E = 05/03/2023; F = 28/07/2023; G = 23/02/2024; H = 24/02/2024.

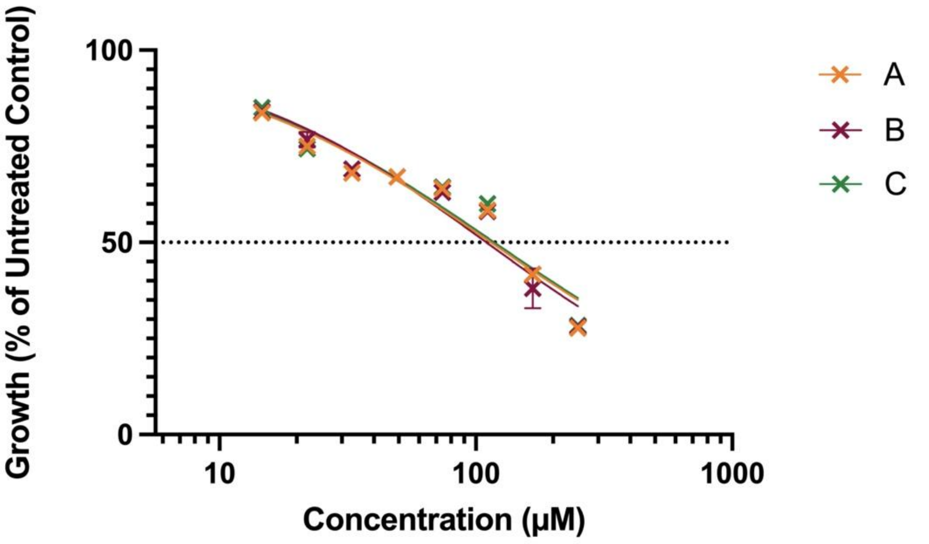

Figure S150 – Graph showing the effects of TBACl on A2780 cells after a 96-hour incubation period followed by analysis as per SRB assay. The data shown represents three separate experiments: A = 20/12/2021; B = 09/01/2022; C = 10/01/2022.

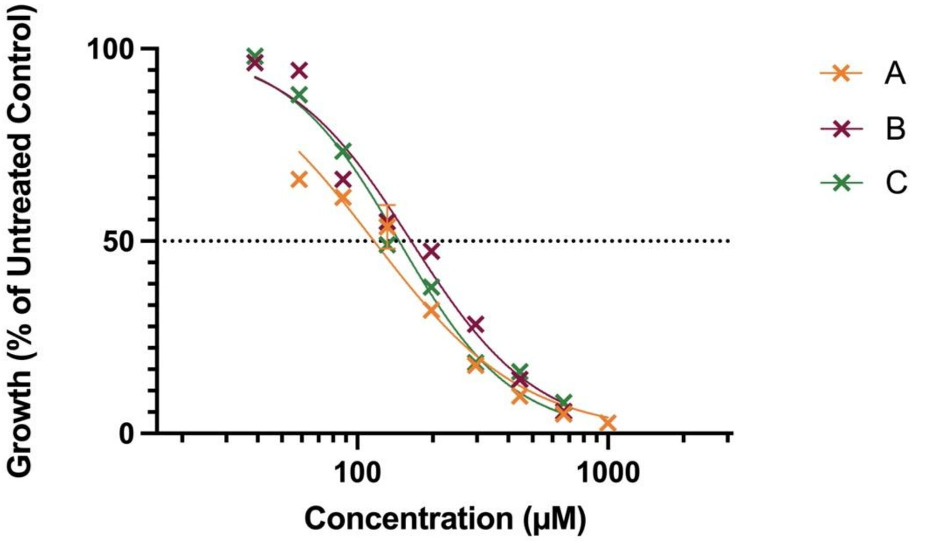

Figure S151 – Graph showing the effects of **1** on A2780 cells after a 96-hour incubation period followed by analysis as per SRB assay. The data shown represents three separate experiments: A = 21/11/2021; B = 28/11/2021; C = 30/11/2021.

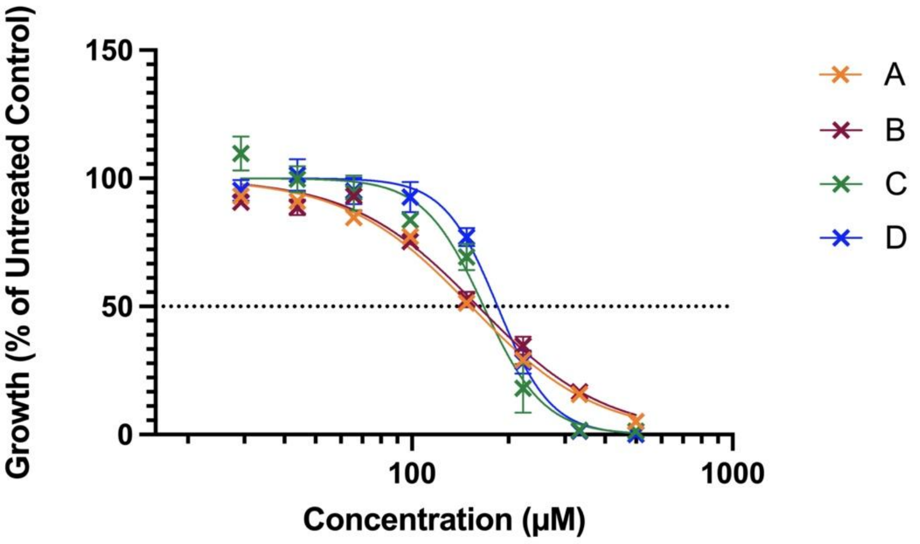

Figure S152 – Graph showing the effects of **3** on A2780 cells after a 96-hour incubation period followed by analysis as per SRB assay. The data shown represents four separate experiments: A = 21/07/2023; B = 22/07/2023; C = 23/02/2024; D = 26/02/2024.

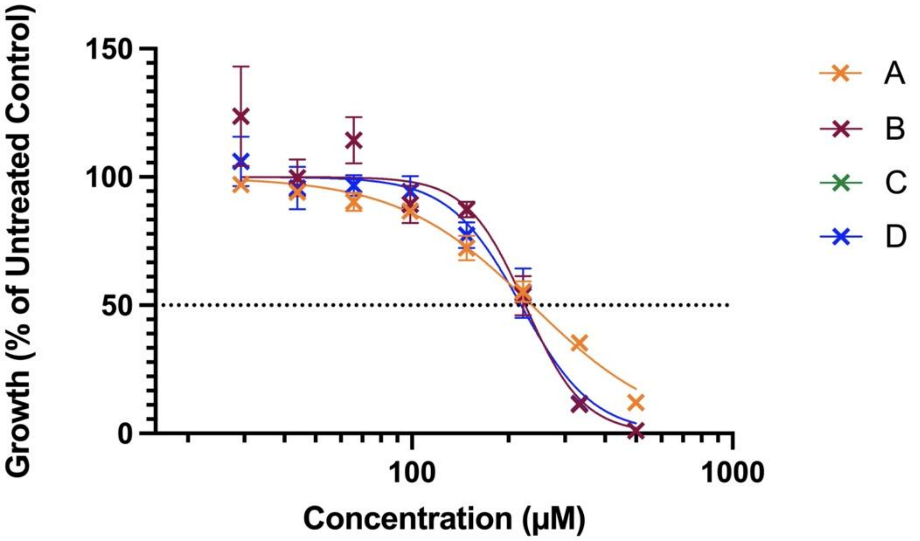

Figure S153 – Graph showing the effects of **5** on A2780 cells after a 96-hour incubation period followed by analysis as per SRB assay. The data shown represents four separate experiments: A = 21/07/2023; B = 22/07/2023; C = 23/02/2024; D = 26/02/2024.

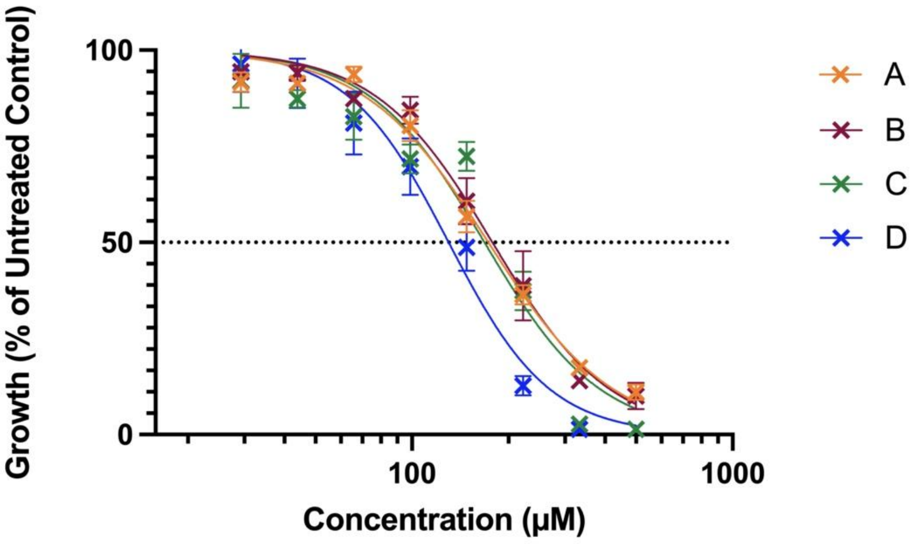

Figure S154 – Graph showing the effects of a 1:1 enantiomeric mixture of **3** + **5** on A2780 cells after a 96-hour incubation period followed by analysis as per SRB assay. The data shown represents four separate experiments: A = 21/07/2023; B = 22/07/2023; C = 23/02/2024; D = 26/02/2024.

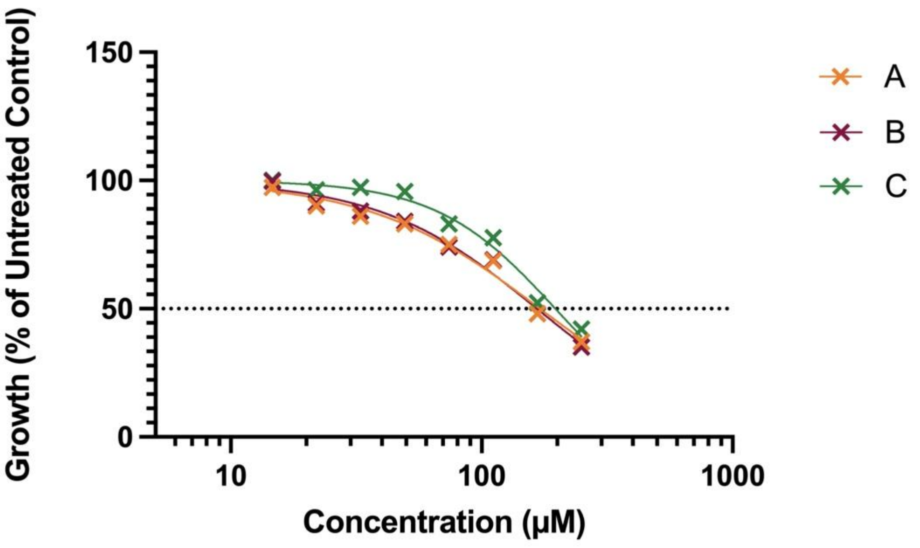

Figure S155 – Graph showing the effects of **7** on A2780 cells after a 96-hour incubation period followed by analysis as per SRB assay. The data shown represents three separate experiments: A = 20/12/2021; B = 09/01/2022; C = 10/01/2022.

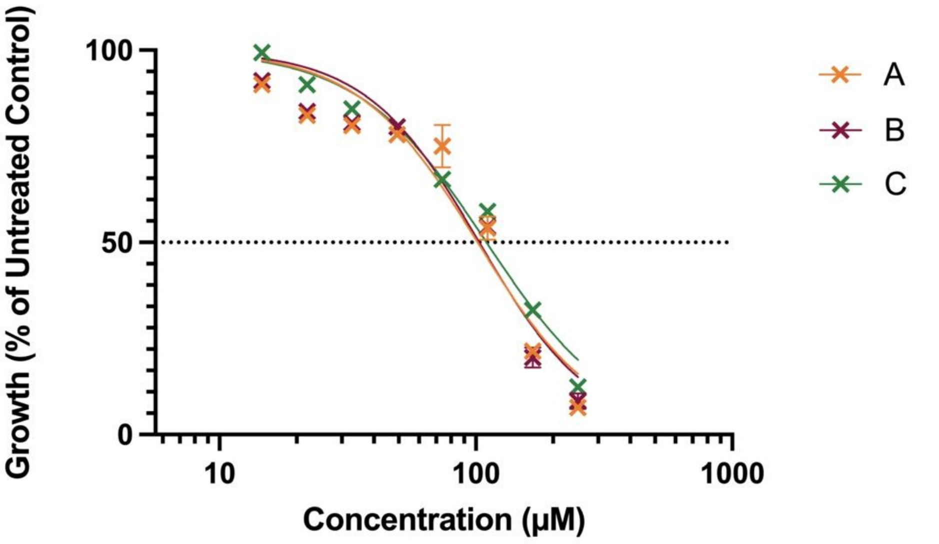

Figure S156 – Graph showing the effects of **9** on A2780 cells after a 96-hour incubation period followed by analysis as per SRB assay. The data shown represents three separate experiments: A = 19/12/2021; B = 09/01/2022; C = 10/01/2022.

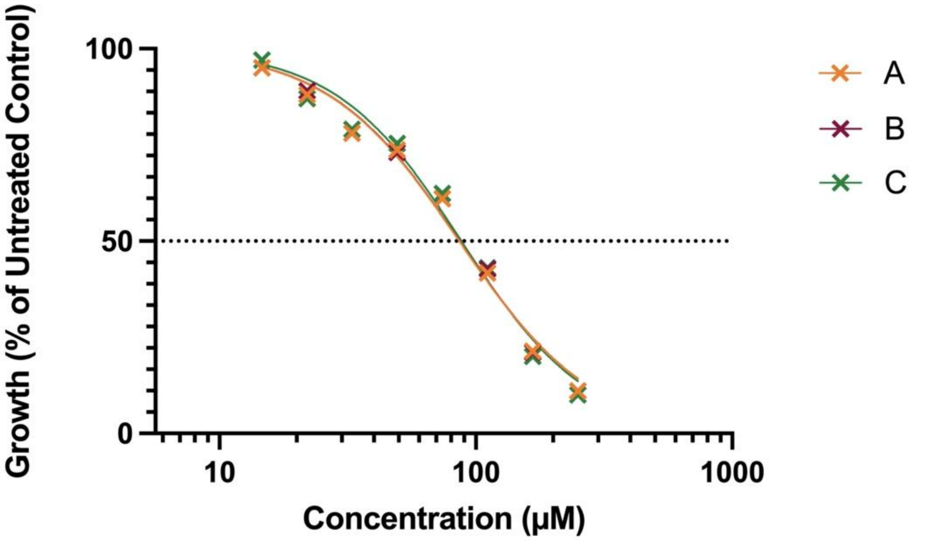

Figure S157 – Graph showing the effects of a 1:1 enantiomeric mixture of 7 + **9** on A2780 cells after a 96-hour incubation period followed by analysis as per SRB assay. The data shown represents three separate experiments: A = 19/12/2021; B = 09/01/2022; C = 10/01/2022.

A2780 CisR cell line

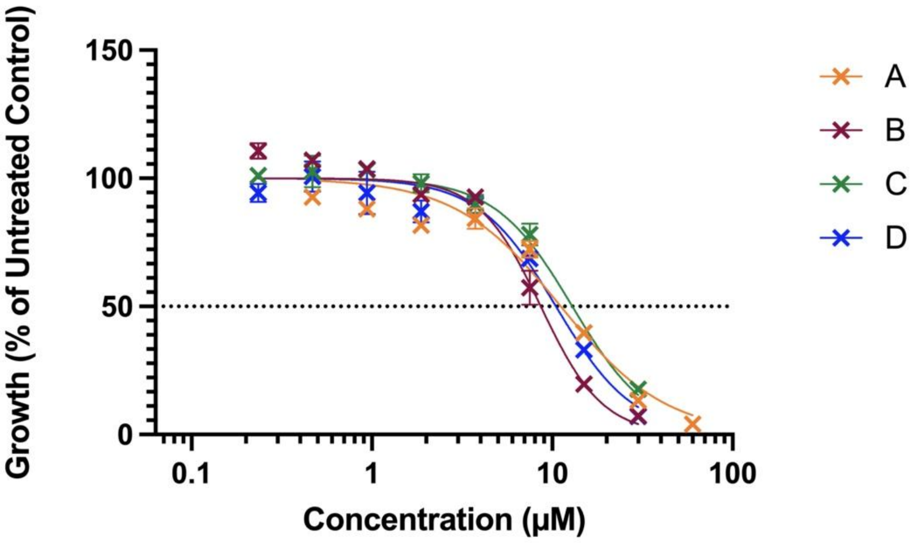

Figure S158 – Graph showing the effects of cisplatin on A2780 CISR cells after a 96-hour incubation period followed by analysis as per SRB assay. The data shown represents four separate experiments: A = 28/11/2021; B = 08/03/2023; C = 11/04/2023; D = 29/11/2023.

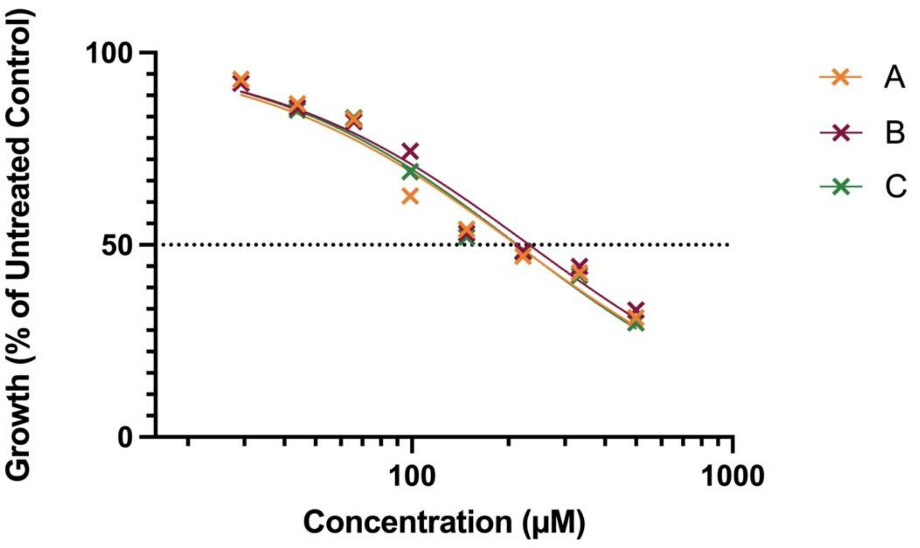

Figure S159 – Graph showing the effects of TBACl on A2780 CISR cells after a 96-hour incubation period followed by analysis as per SRB assay. The data shown represents three separate experiments: A = 19/12/2021; B = 20/12/2021; C = 09/01/2022.

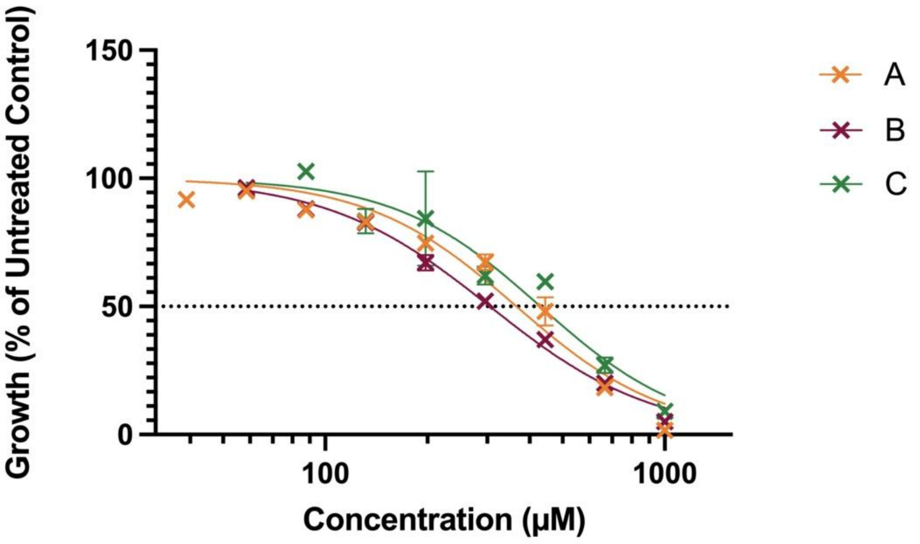

Figure S160 – Graph showing the effects of **1** on A2780 CISR cells after a 96-hour incubation period followed by analysis as per SRB assay. The data shown represents three separate experiments: A = 08/11/2021; B = 28/11/2021; C = 14/12/2021.

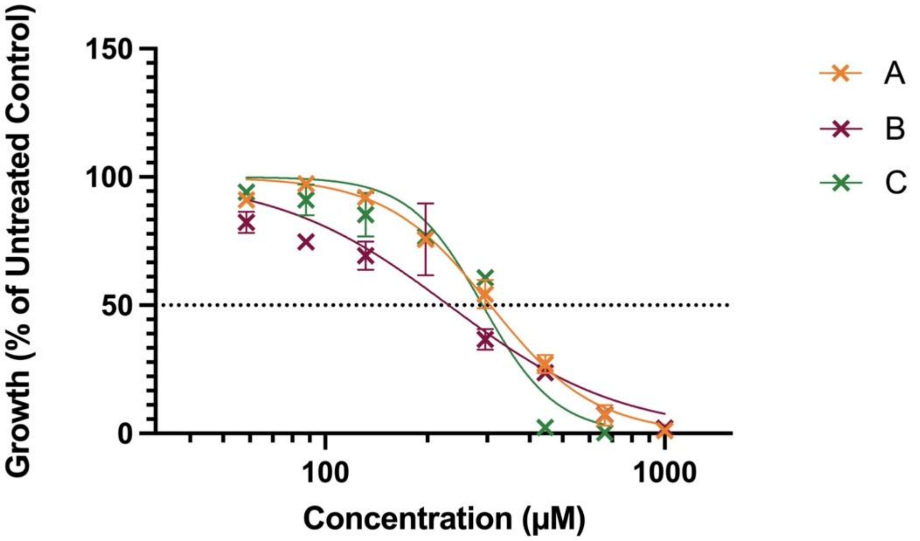

Figure S161 – Graph showing effects of **3** on A2780 CISR cells after a 96-hour incubation period followed by analysis as per SRB assay. The data shown represents three separate experiments: A = 26/11/2023; B = 29/11/2023; C = 13/02/2024.

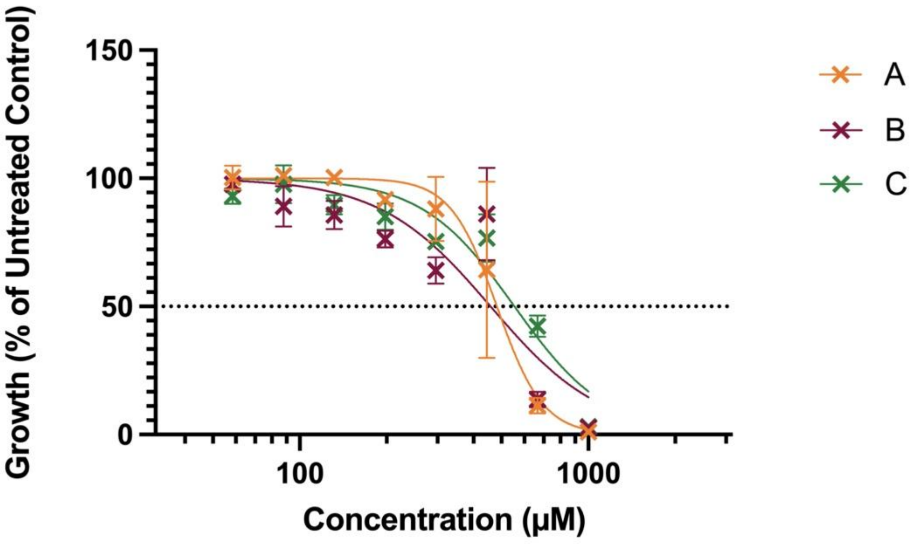

Figure S162 – Graph showing the effects of **5** on A2780 CISR cells after a 96-hour incubation period followed by analysis as per SRB assay. The data shown represents three separate experiments: A = 26/11/2023; B = 29/11/2023; C = 13/02/2024.

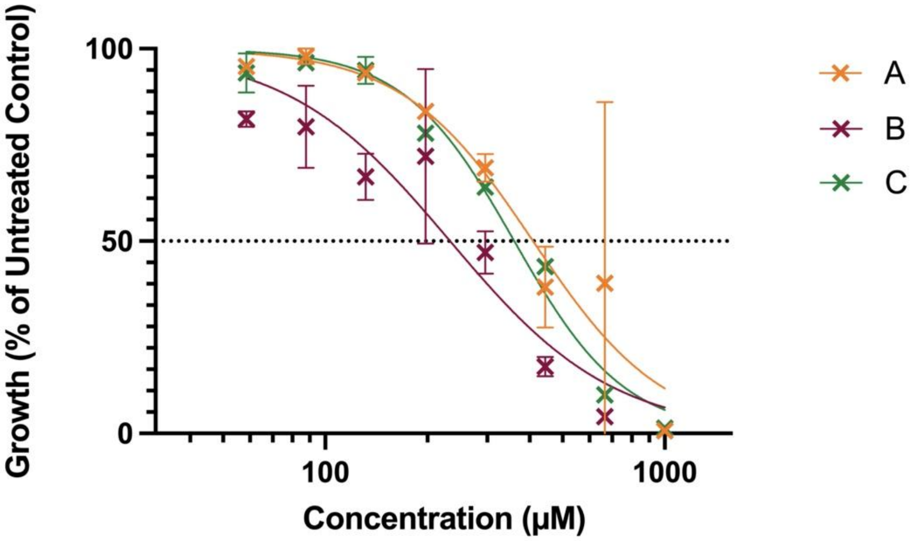

Figure S163 – Graph showing effects of a 1:1 enantiomeric mixture of **3** + **5** on A2780 CISR cells after a 96-hour incubation period followed by analysis as per SRB assay. The data shown represents three separate experiments: A = 26/11/2023; B = 29/11/2023; C = 13/02/2024.

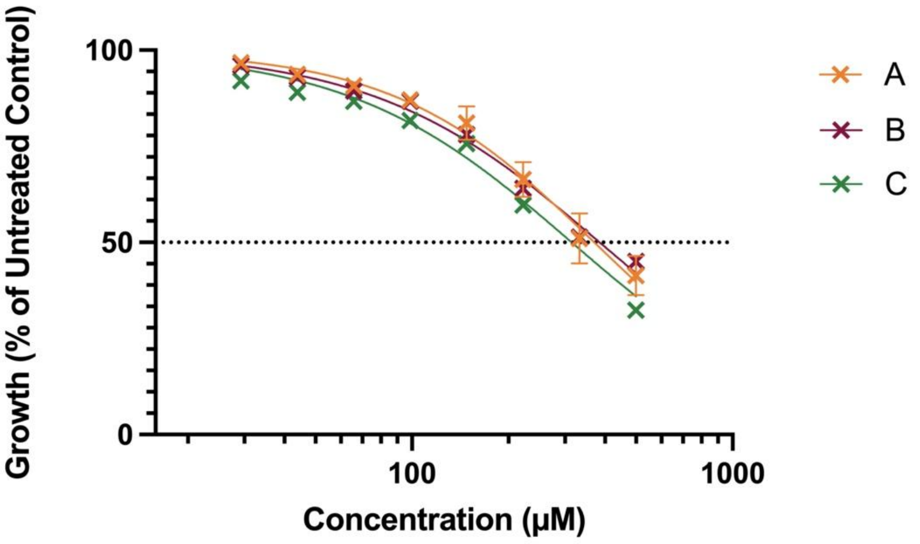

Figure S164 – Graph showing effects of **7** on A2780 CISR cells after a 96-hour incubation period followed by analysis as per SRB assay. The data shown represents three separate experiments: A = 19/12/2021; B = 20/12/2021; C = 09/01/2022.

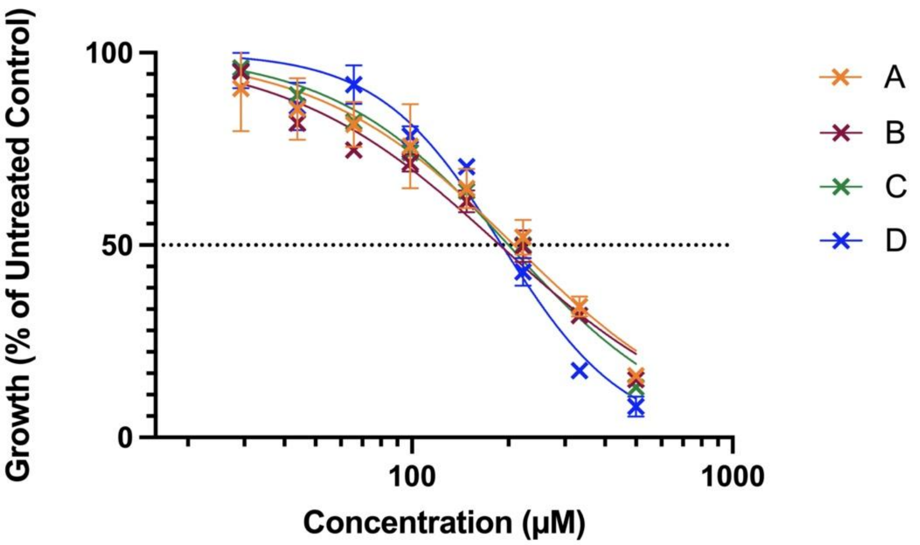

Figure S165 – Graph showing effects of **9** on A2780 CISR cells after a 96-hour incubation period followed by analysis as per SRB assay. The data shown represents four separate experiments: A = 19/12/2021; B = 20/12/2021; C = 09/01/2022; D = 10/01/2022.

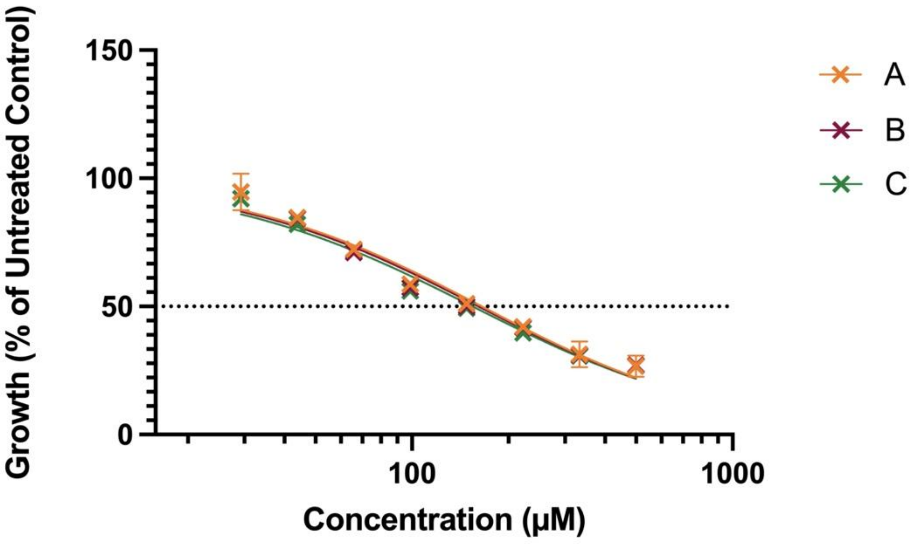

Figure S166 – Graph showing the effects of a 1:1 enantiomeric mixture of **7** + **9** on A2780 CISR cells after a 96-hour incubation period followed by analysis as per SRB assay. The data shown represents three separate experiments: A = 19/12/2021; B = 20/12/2021; C = 09/01/2022.

Summary

Table S16 - The concentration required to reduce/ inhibit cellular growth by 50 % (GI_{50}) determined for cisplatin, TBACl, **1**, **3**, **5**, **7**, **9**, and 1:1 enantiomeric mixtures of **3** + **5** and **7** + **9** against non-cancerous RPE-1 cells, ovarian cancer cells (A2780) and cisplatin resistant ovarian cancer cells (A2780 CisR).

	**RPE-1 SRB Assays**			**A2780 SRB Assays**			**A2780 CisR SRB Assays**		
**Compound**	**Mean GI_{50} (µM)**	**SD +/-**	**R^2 > 0.9 n =**	**Mean GI_{50} (µM)**	**SD +/-**	**R^2 > 0.9 n =**	**Mean GI_{50} (µM)**	**SD +/-**	**R^2 > 0.9 n =**
Cisplatin	6.25	1.44	3	1.088	0.35	8	10.804	1.77	4
TBACl	532.46	222	3	114.16	4.01	3	219.26	11.21	3
**1**	295.93	210	3	116.53	6.50	3	367.20	62.46	3
**3**	> 500	*b*	*b*	171.03	12.45	3	276.06	39.7	3
**5**	> 375	*b*	*b*	230.90	9.25	3	498.40	50.17	3
**3+5** [a]	> 375	*b*	*b*	162.40	21.90	3	334.40	91.00	3
**7**	> 375	*b*	*b*	177.76	16.47	3	356.33	36.94	3
**9**	> 375	*b*	*b*	104.93	4.44	3	196.72	9.25	4
**7+9** [a]	> 375	*b*	*b*	86.81	0.90	3	160.33	5.35	3

a – 1:1 mixture.

b = not applicable.

Section S20: Toxicity assay

Table S17 – The toxicity of **1**, **3**, **5**, **7**, **9**, and 1:1 enantiomeric mixtures of **3** + **5** and **7** + **9** determined by observing the number of *Galleria mellonella* moth larvae that survived out of 10 after injection of compound after five days.

	**Galleria moth larvae survived**				
**Compound**	**Day 1**	**Day 2**	**Day 3**	**Day 4**	**Day 5**
**PBS**	10	10	10	10	10
**H_2O/5 % EtOH**	10	10	10	10	10
**1**	10	9	8	6	6
**3**	9	9	9	6	5
**5**	10	9	8	8	8
**3+5** [a]	7	7	7	4	3
**7**	10	4	4	4	4
**9**	9	9	9	7	5
**7+9** [a]	9	8	4	4	4

a – 1:1 mixture.

Section S21: Haemolysis assay data

Table S18 - The haemolysis of red blood cells (RBCs) determined for **1**, **3**, **5**, **7**, **9**, and 1:1 enantiomeric mixtures of **3** + **5** and **7** + **9** obtained at 1.39 mM.

**Compound**	**Haemolysis at 1.39 mM (%)**	**Standard deviation**
**H_2O/ 5 % EtOH**	0.54	0.05
**1**	0.29	0.56
**3**	- 0.44	1.78
**5**	- 0.23	1.22
**3+5** [a]	0.83	0.54
**7**	0.47	0.48
**9**	- 0.34	0.54
**7+9** [a]	0.28	0.52

a – 1:1 mixture.

Section S22: Patch clamp studies

Table S19 - Total experiment time of experiments detailed in Figure 6 (main manuscript), that do not comply with a 1000 (+/- 5) second (s) timeframe.

SSA	Exp. No.	Time (s)	SSA	Exp. No.	Time (s)
**1**	215	2013	**5**	11	395
**1**	206	1045	**5**	3	601
**1**	209	1124	**5**	4	602
**3**	104	2011	**5**	5	601
**3**	87	2075	**5**	6	601
**3**	91	1918	**5**	67	2003
**3**	99	1652	**5**	69	1838
**3 + 5**	53	701	**5**	80	1064
**3 + 5**	54	701	**7**	233	2110
**3 + 5**	50	701	**7**	235	1073
**3 + 5**	51	701	**7 + 9**	47	701
**3 + 5**	52	701	**7 + 9**	48	701
**5**	53	1617	**7 + 9**	49	701
**5**	40	2004	**7 + 9**	44	706
**5**	43	1501	**7 + 9**	45	701
**5**	49	1622	**7 + 9**	46	701
**5**	12	513	**9**	42	1505
**5**	16	601	**9**	36	701
**5**	9	606	**9**	17	301
**5**	10	395			

Changing concentration of **1**

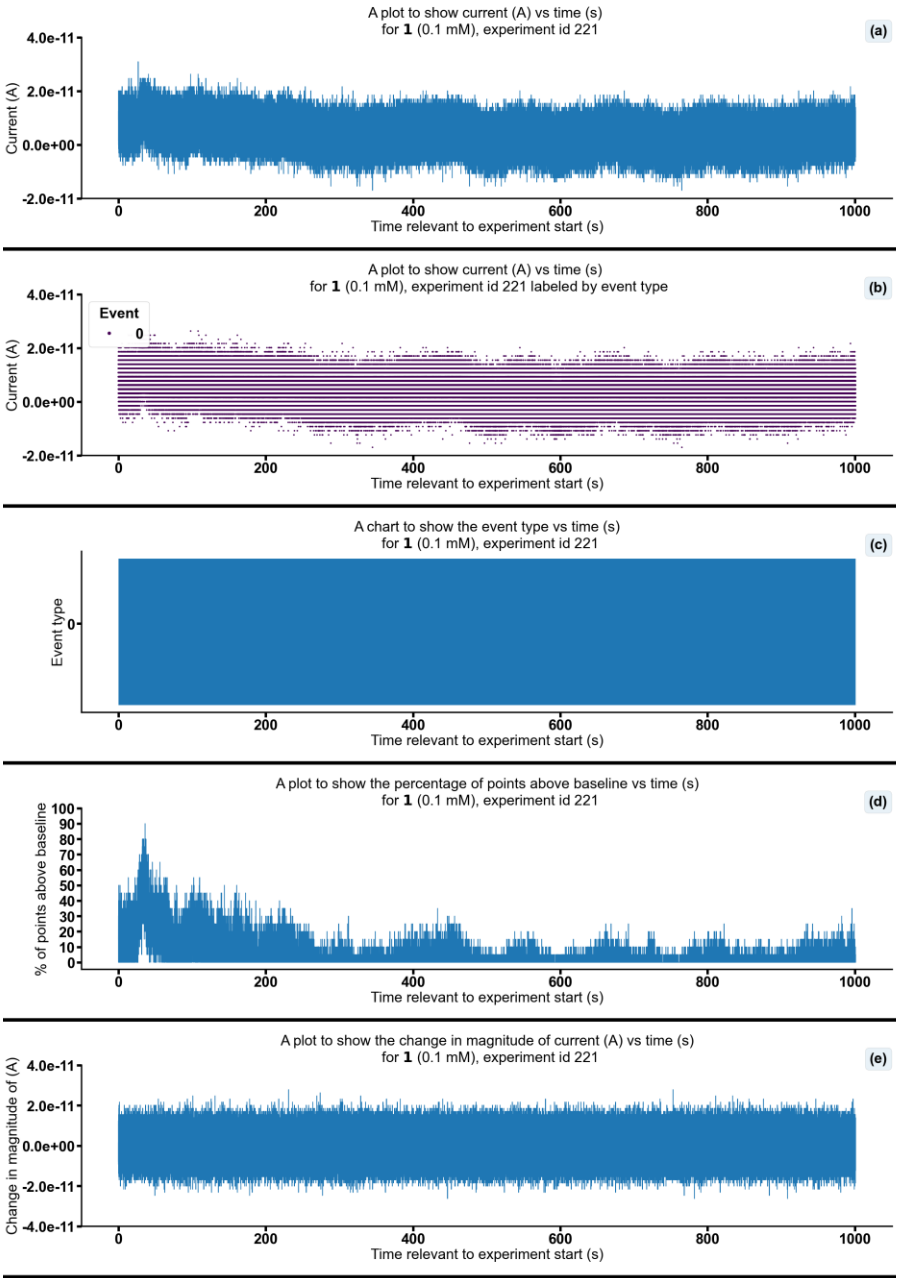

Figure S167 - Summarising the data from the patch clamp experiment for **1** (0.1 mM), experiment id 221 at +100 mV. Events were categorised into the following types: 0: -∞ A ≤ Event 0 < 5.00e-11 A, 1: 5.00e-11 A ≤ Event 1 < 5.00e-10 A, 2: 5.00e-10 A ≤ Event 2 < 4.90e-08 A, 3: 4.90e-08 A ≤ Event 3 < ∞ A. Subfigure (d) shows the percentage of points found above the threshold 1.2e-11 (A) in a rolling look ahead window of 20 points. The threshold is the mean + standard deviation of the noise of the first 200 datapoints.

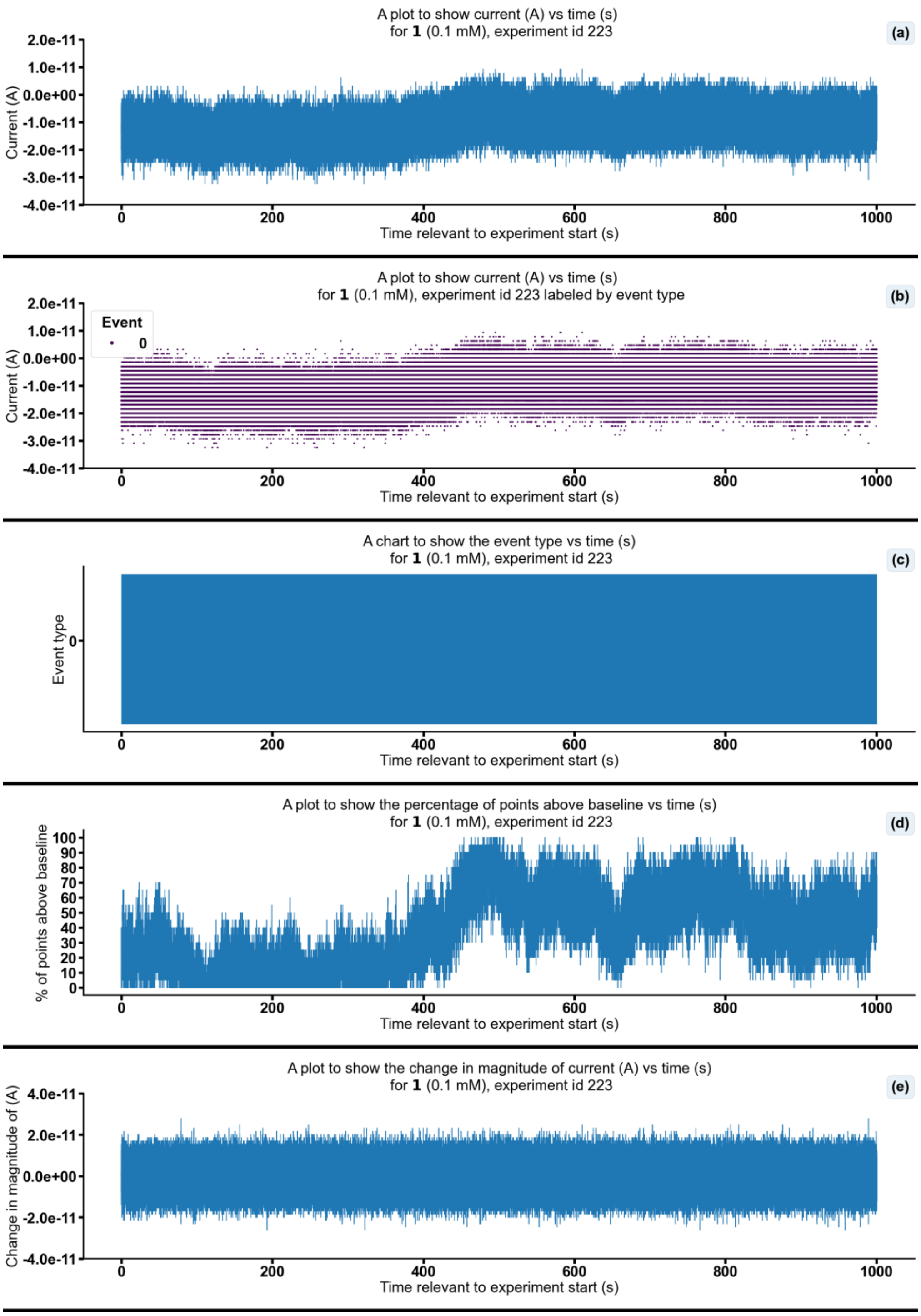

Figure S168 - Summarising the data from the patch clamp experiment for **1** (0.1 mM), experiment id 223 at +100 mV. Events were categorised into the following types: 0: -∞ A ≤ Event 0 < 5.00e-11 A, 1: 5.00e-11 A ≤ Event 1 < 5.00e-10 A, 2: 5.00e-10 A ≤ Event 2 < 4.90e-08 A, 3: 4.90e-08 A ≤ Event 3 < ∞ A. Subfigure (d) shows the percentage of points found above the threshold -9.5e-12 (A) in a rolling look ahead window of 20 points. The threshold is the mean + standard deviation of the noise of the first 200 datapoints.

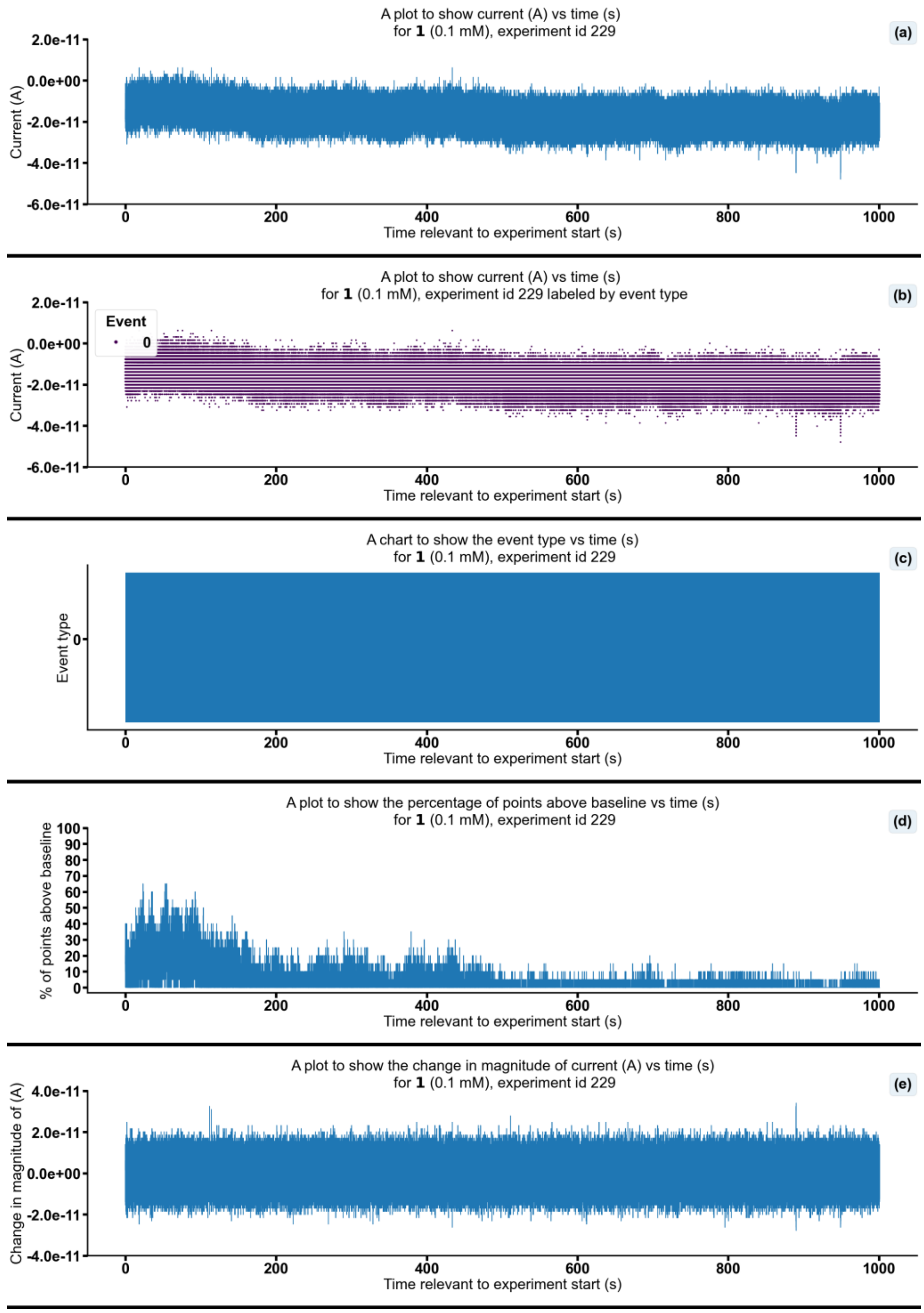

Figure S169 - Summarising the data from the patch clamp experiment for **1** (0.1 mM), experiment id 229 at +100 mV. Events were categorised into the following types: 0: -∞ A ≤ Event 0 < 5.00e-11 A, 1: 5.00e-11 A ≤ Event 1 < 5.00e-10 A, 2: 5.00e-10 A ≤ Event 2 < 4.90e-08 A, 3: 4.90e-08 A ≤ Event 3 < ∞ A. Subfigure (d) shows the percentage of points found above the threshold -8.3e-12 (A) in a rolling look ahead window of 20 points. The threshold is the mean + standard deviation of the noise of the first 200 datapoints.

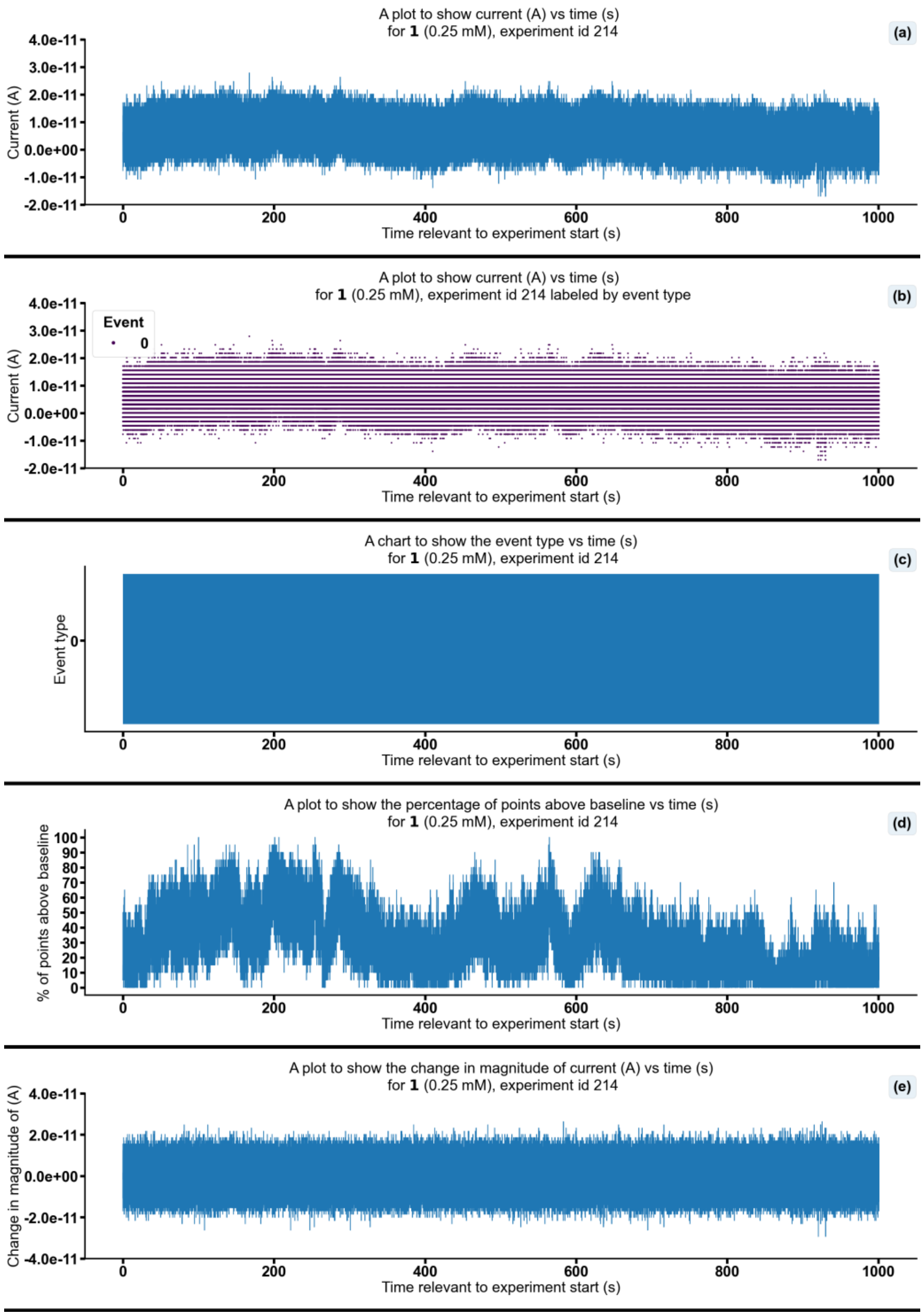

Figure S170 - Summarising the data from the patch clamp experiment for **1** (0.25 mM), experiment id 214 at +100 mV. Events were categorised into the following types: 0: -∞ A ≤ Event 0 < 5.00e-11 A, 1: 5.00e-11 A ≤ Event 1 < 5.00e-10 A, 2: 5.00e-10 A ≤ Event 2 < 4.90e-08 A, 3: 4.90e-08 A ≤ Event 3 < ∞ A. Subfigure (d) shows the percentage of points found above the threshold 8.4e-12 (A) in a rolling look ahead window of 20 points. The threshold is the mean + standard deviation of the noise of the first 200 datapoints.

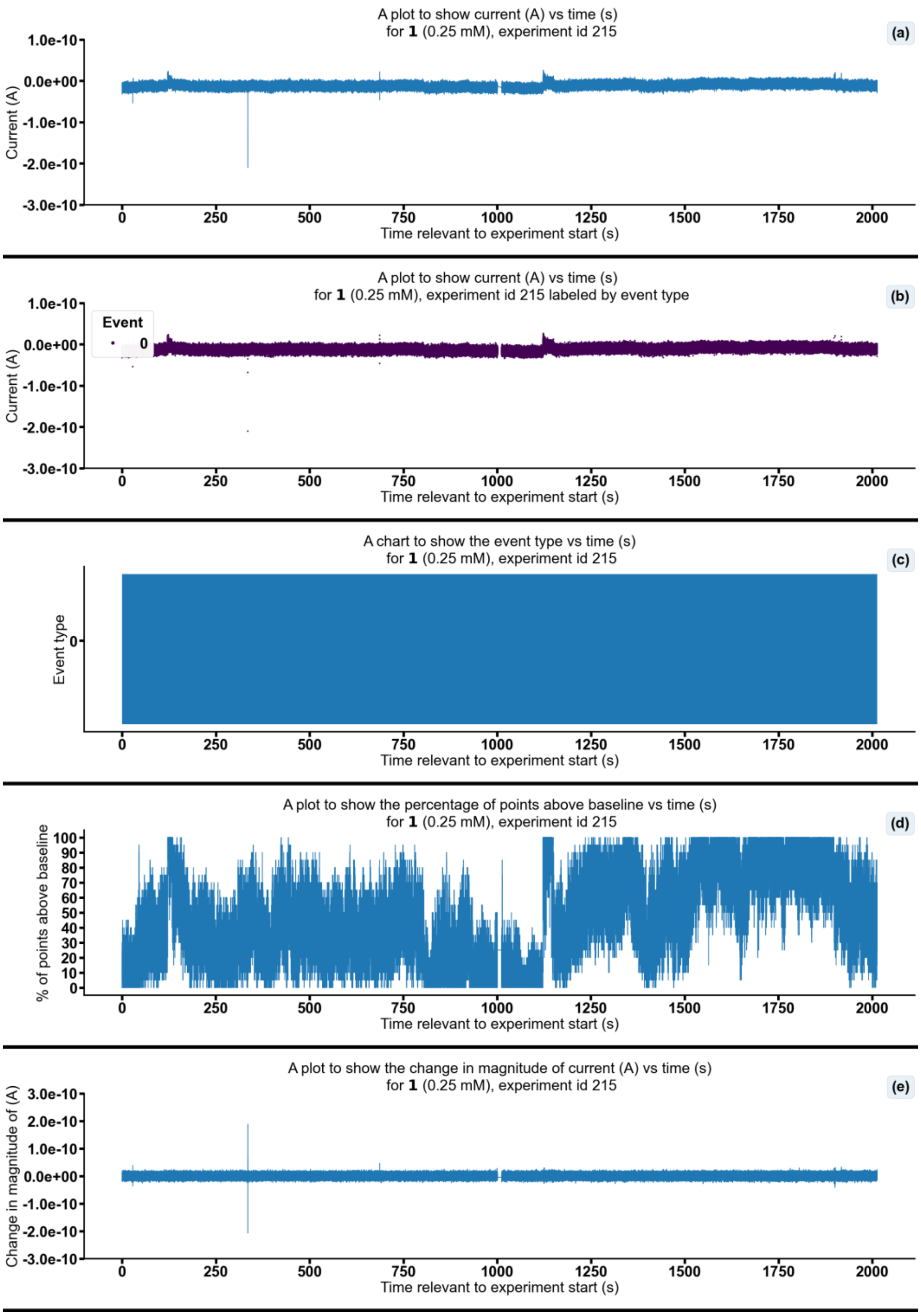

Figure S171 - Summarising the data from the patch clamp experiment for **1** (0.25 mM), experiment id 215 at +100 mV. Events were categorised into the following types: 0: -∞ A ≤ Event 0 < 5.00e-11 A, 1: 5.00e-11 A ≤ Event 1 < 5.00e-10 A, 2: 5.00e-10 A ≤ Event 2 < 4.90e-08 A, 3: 4.90e-08 A ≤ Event 3 < ∞ A. Subfigure (d) shows the percentage of points found above the threshold -1.2e-11 (A) in a rolling look ahead window of 20 points. The threshold is the mean + standard deviation of the noise of the first 200 datapoints.

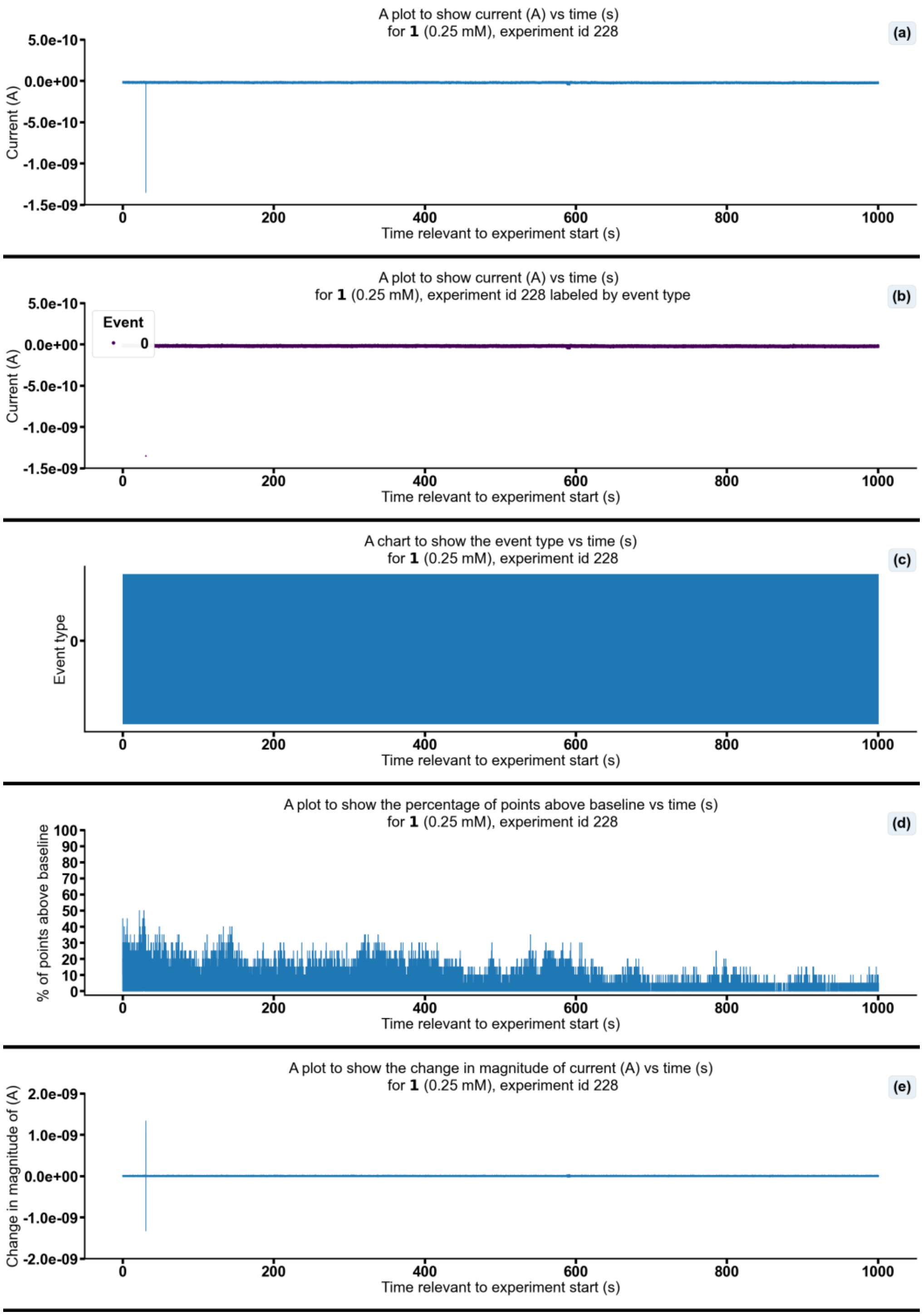

Figure S172 - Summarising the data from the patch clamp experiment for **1** (0.25 mM), experiment id 228 at +100 mV. Events were categorised into the following types: 0: -∞ A ≤ Event 0 < 5.00e-11 A, 1: 5.00e-11 A ≤ Event 1 < 5.00e-10 A, 2: 5.00e-10 A ≤ Event 2 < 4.90e-08 A, 3: 4.90e-08 A ≤ Event 3 < ∞ A. For (d), the percentage of points found above -1.5e-11 (A) in a rolling look ahead window of 20 points. The threshold is the mean + standard deviation of the noise of the first 200 datapoints.

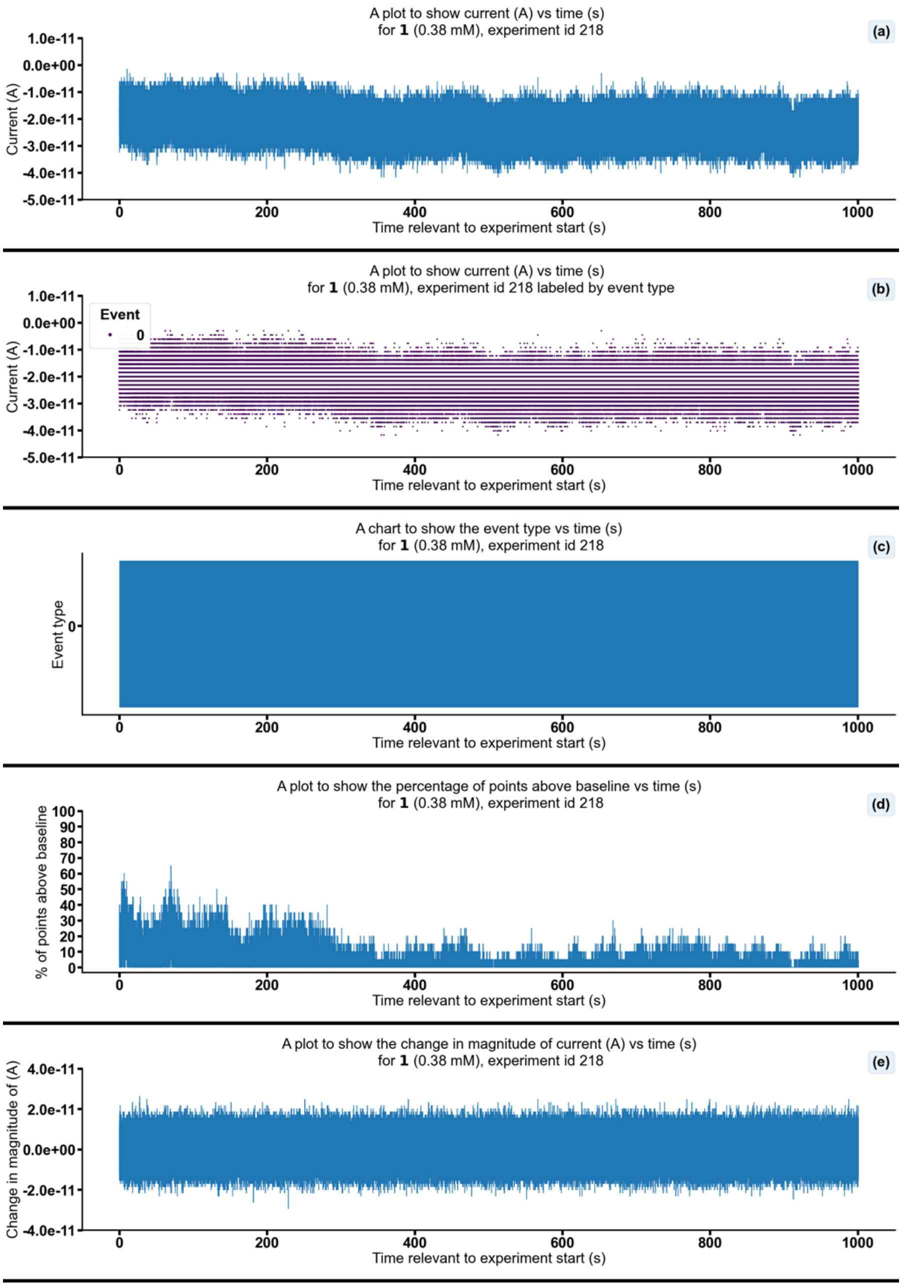

Figure S173 - Summarising the data from the patch clamp experiment for **1** (0.38 mM), experiment id 218 at +100 mV. Events were categorised into the following types: 0: -∞ A ≤ Event 0 < 5.00e-11 A, 1: 5.00e-11 A ≤ Event 1 < 5.00e-10 A, 2: 5.00e-10 A ≤ Event 2 < 4.90e-08 A, 3: 4.90e-08 A ≤ Event 3 < ∞ A. Subfigure (d) shows the percentage of points found above the threshold -1.4e-11 (A) in a rolling look ahead window of 20 points. The threshold is the mean + standard deviation of the noise of the first 200 datapoints.

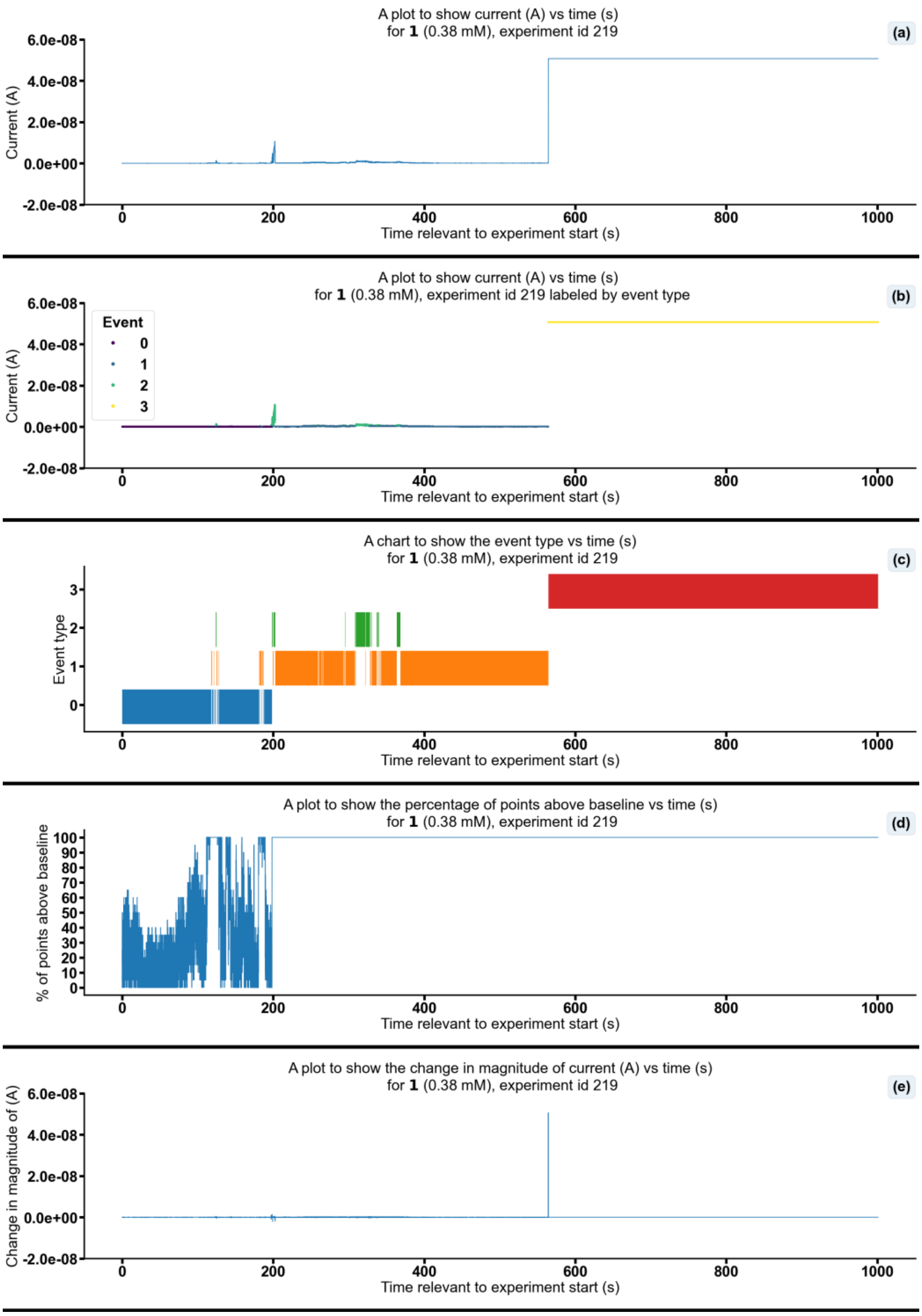

Figure S174 - Summarising the data from the patch clamp experiment for **1** (0.38 mM), experiment id 219 at +100 mV. Events were categorised into the following types: 0: -∞ A ≤ Event 0 < 5.00e-11 A, 1: 5.00e-11 A ≤ Event 1 < 5.00e-10 A, 2: 5.00e-10 A ≤ Event 2 < 4.90e-08 A, 3: 4.90e-08 A ≤ Event 3 < ∞ A. Subfigure (d) shows the percentage of points found above the threshold 1.1e-11 (A) in a rolling look ahead window of 20 points. The threshold is the mean + standard deviation of the noise of the first 200 datapoints.

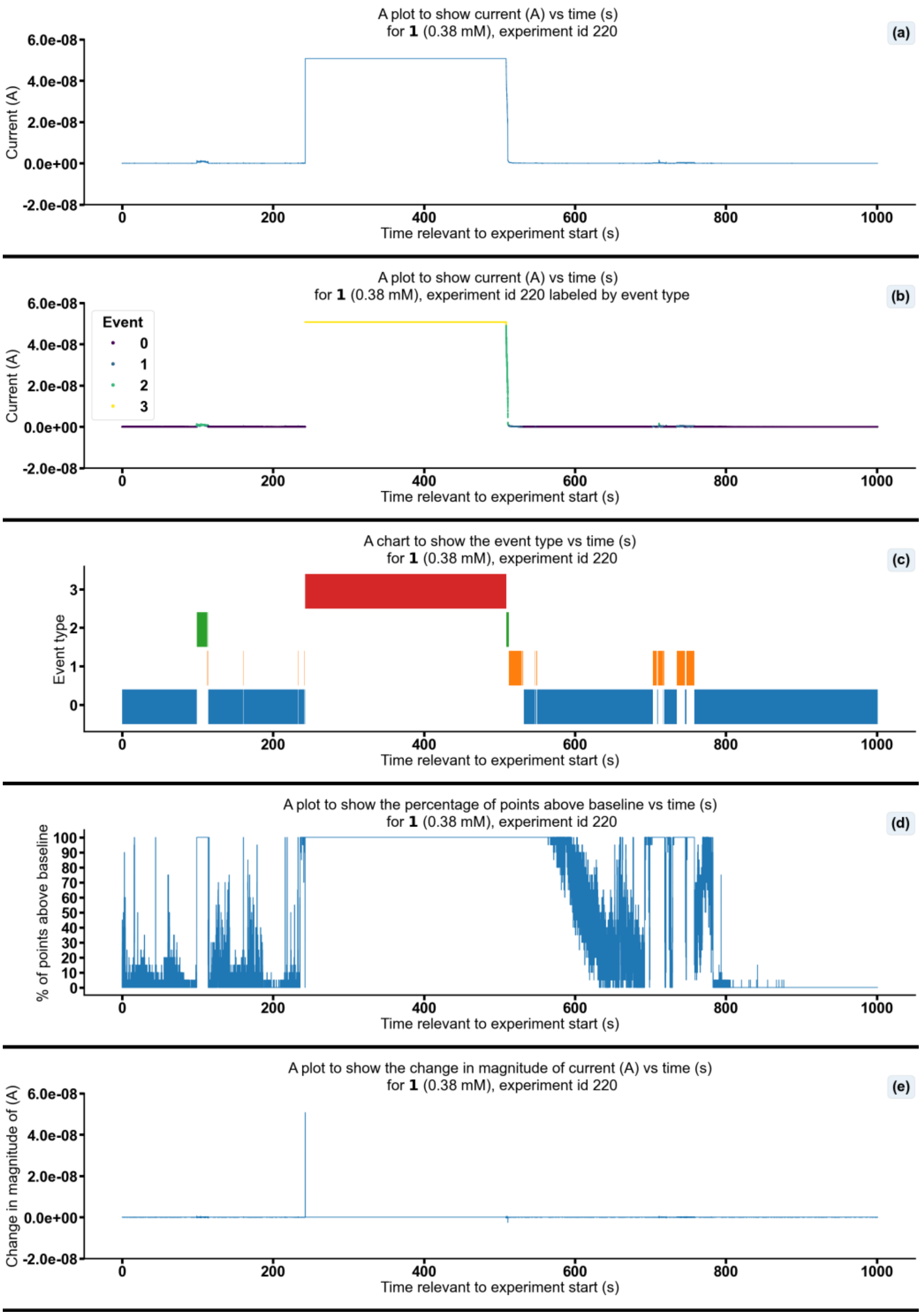

Figure S175 - Summarising the data from the patch clamp experiment for **1** (0.38 mM), experiment id 220 at +100 mV. Events were categorised into the following types: 0: -∞ A ≤ Event 0 < 5.00e-11 A, 1: 5.00e-11 A ≤ Event 1 < 5.00e-10 A, 2: 5.00e-10 A ≤ Event 2 < 4.90e-08 A, 3: 4.90e-08 A ≤ Event 3 < ∞ A. Subfigure (d) shows the percentage of points found above the threshold -9.4e-13 (A) in a rolling look ahead window of 20 points. The threshold is the mean + standard deviation of the noise of the first 200 datapoints.

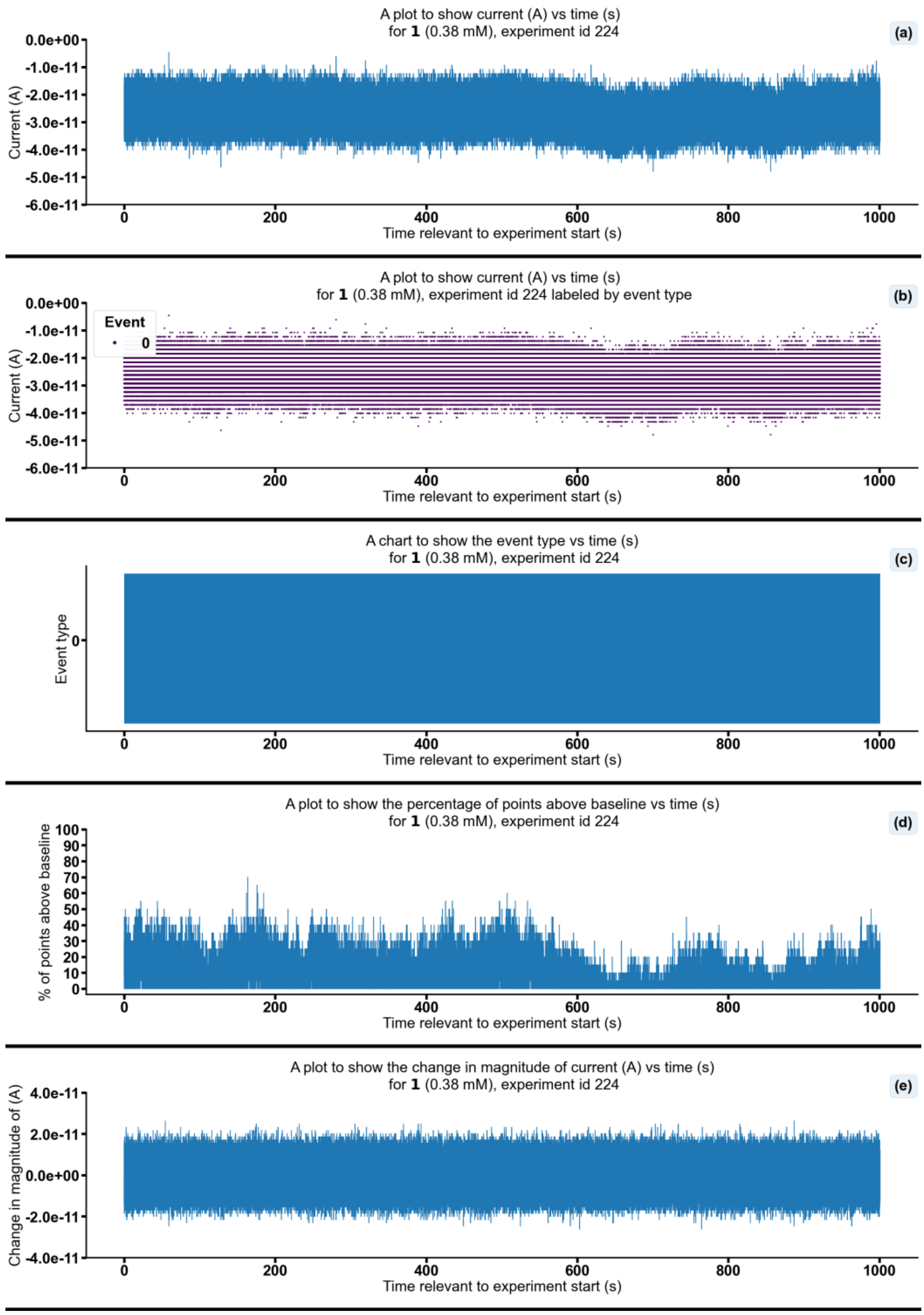

Figure S176 - Summarising the data from the patch clamp experiment for **1** (0.38 mM), experiment id 224 at +100 mV. Events were categorised into the following types: 0: -∞ A ≤ Event 0 < 5.00e-11 A, 1: 5.00e-11 A ≤ Event 1 < 5.00e-10 A, 2: 5.00e-10 A ≤ Event 2 < 4.90e-08 A, 3: 4.90e-08 A ≤ Event 3 < ∞ A. Subfigure (d) shows the percentage of points found above the threshold -2.1e-11 (A) in a rolling look ahead window of 20 points. The threshold is the mean + standard deviation of the noise of the first 200 datapoints.

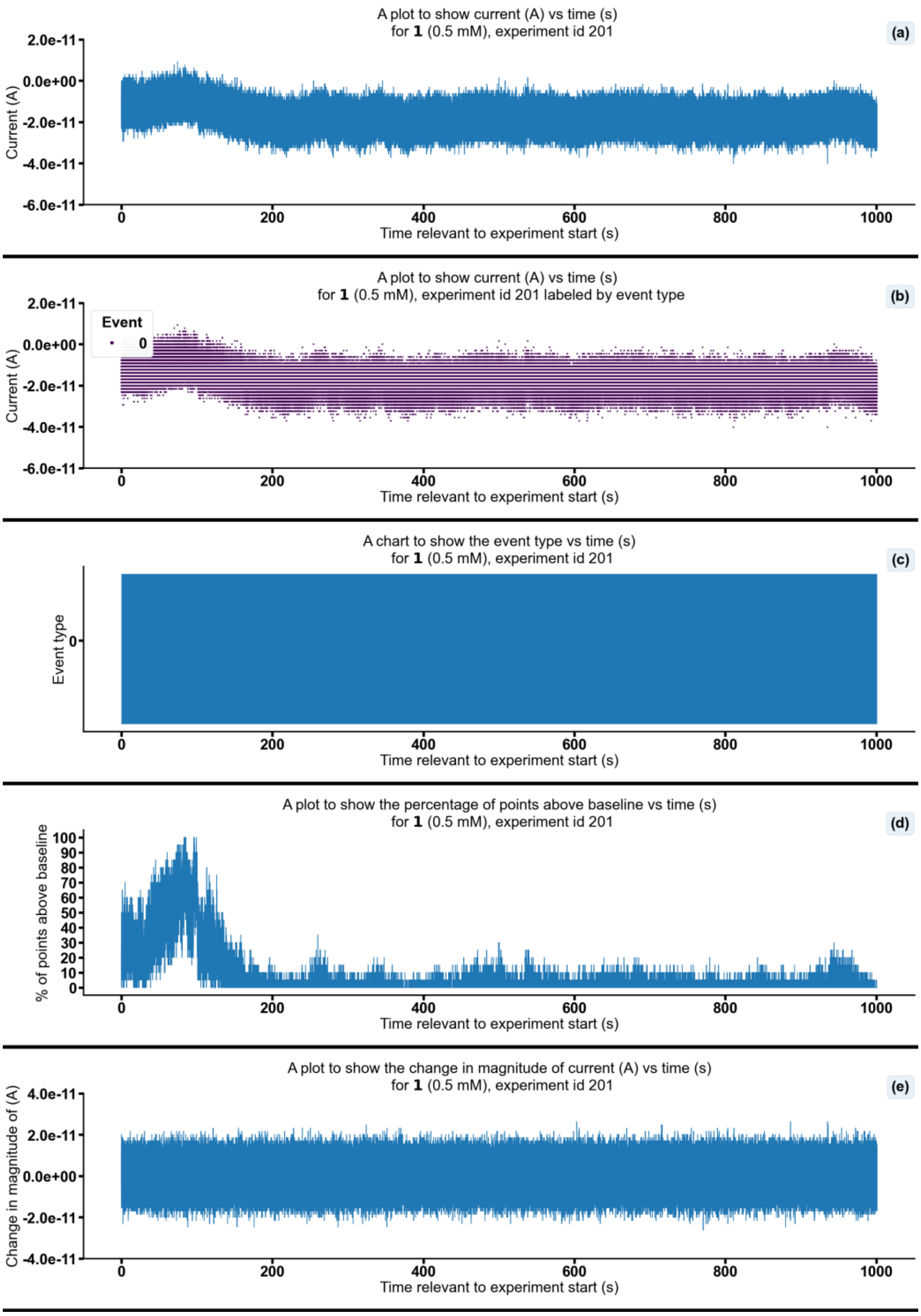

Figure S177 - Summarising the data from the patch clamp experiment for **1** (0.5 mM), experiment id 201 at +100 mV. Events were categorised into the following types: 0: -∞ A ≤ Event 0 < 5.00e-11 A, 1: 5.00e-11 A ≤ Event 1 < 5.00e-10 A, 2: 5.00e-10 A ≤ Event 2 < 4.90e-08 A, 3: 4.90e-08 A ≤ Event 3 < ∞ A. Subfigure (d) shows the percentage of points found above the threshold -9.3e-12 (A) in a rolling look ahead window of 20 points. The threshold is the mean + standard deviation of the noise of the first 200 datapoints.

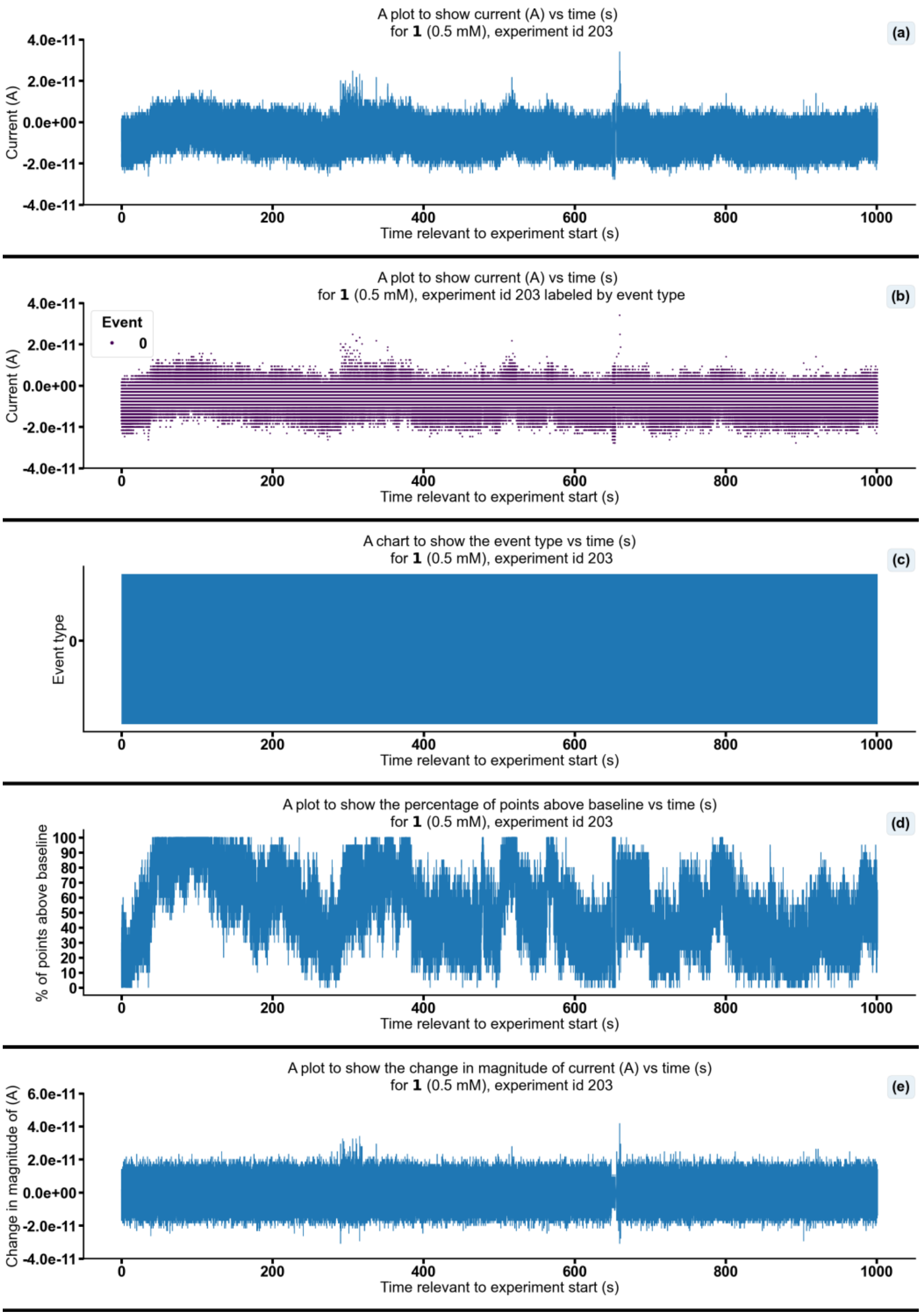

Figure S178 - Summarising the data from the patch clamp experiment for **1** (0.5 mM), experiment id 203 at +100 mV. Events were categorised into the following types: 0: -∞ A ≤ Event 0 < 5.00e-11 A, 1: 5.00e-11 A ≤ Event 1 < 5.00e-10 A, 2: 5.00e-10 A ≤ Event 2 < 4.90e-08 A, 3: 4.90e-08 A ≤ Event 3 < ∞ A. For (d), the percentage of points found above -6.6e-12 (A) in a rolling look ahead window of 20 points. The threshold is the mean + standard deviation of the noise of the first 200 datapoints.

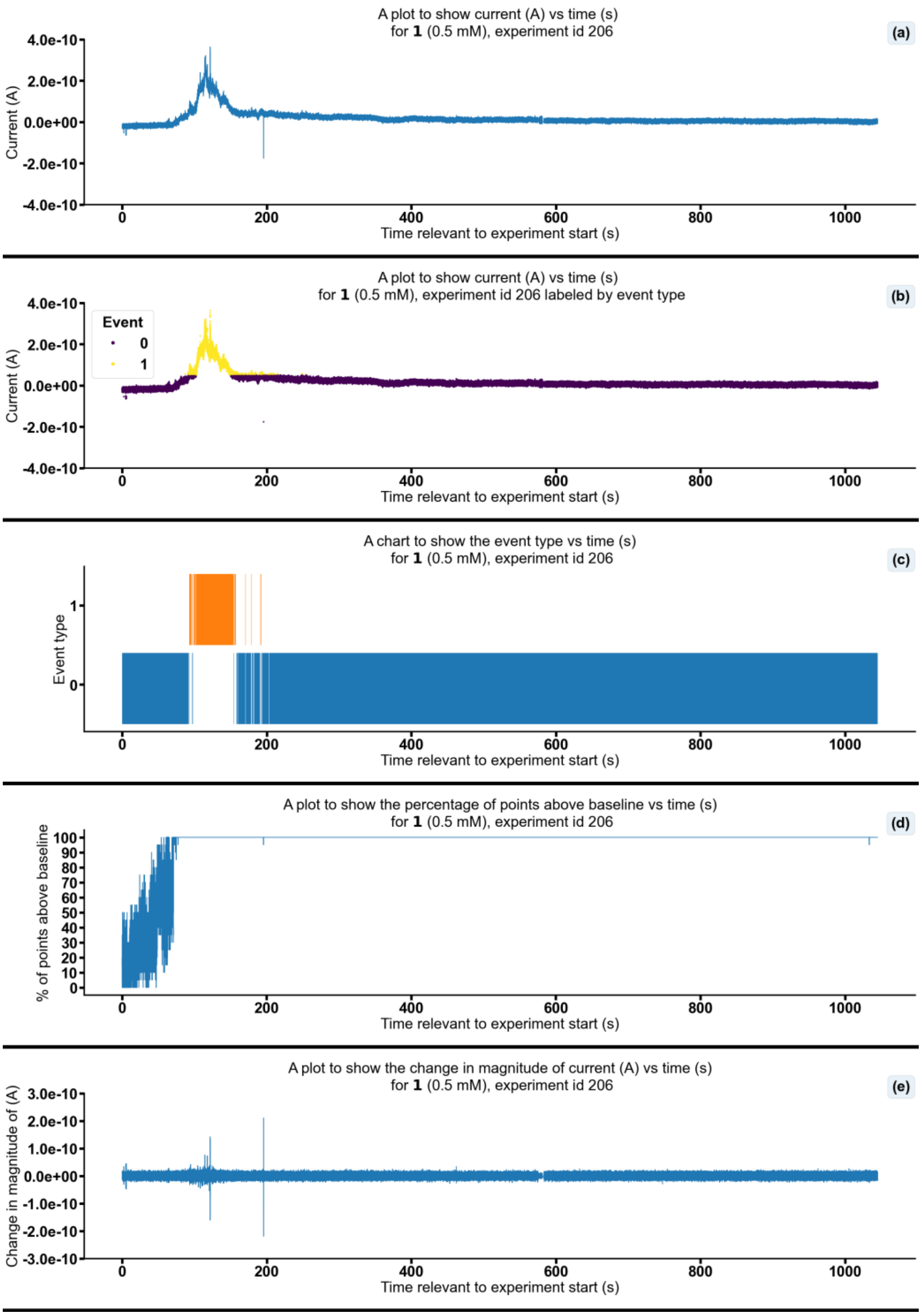

Figure S179 - Summarising the data from the patch clamp experiment for **1** (0.5 mM), experiment id 206 at +100 mV. Events were categorised into the following types: 0: -∞ A ≤ Event 0 < 5.00e-11 A, 1: 5.00e-11 A ≤ Event 1 < 5.00e-10 A, 2: 5.00e-10 A ≤ Event 2 < 4.90e-08 A, 3: 4.90e-08 A ≤ Event 3 < ∞ A. Subfigure (d) shows the percentage of points found above the threshold -1.7e-11 (A) in a rolling look ahead window of 20 points. The threshold is the mean + standard deviation of the noise of the first 200 datapoints.

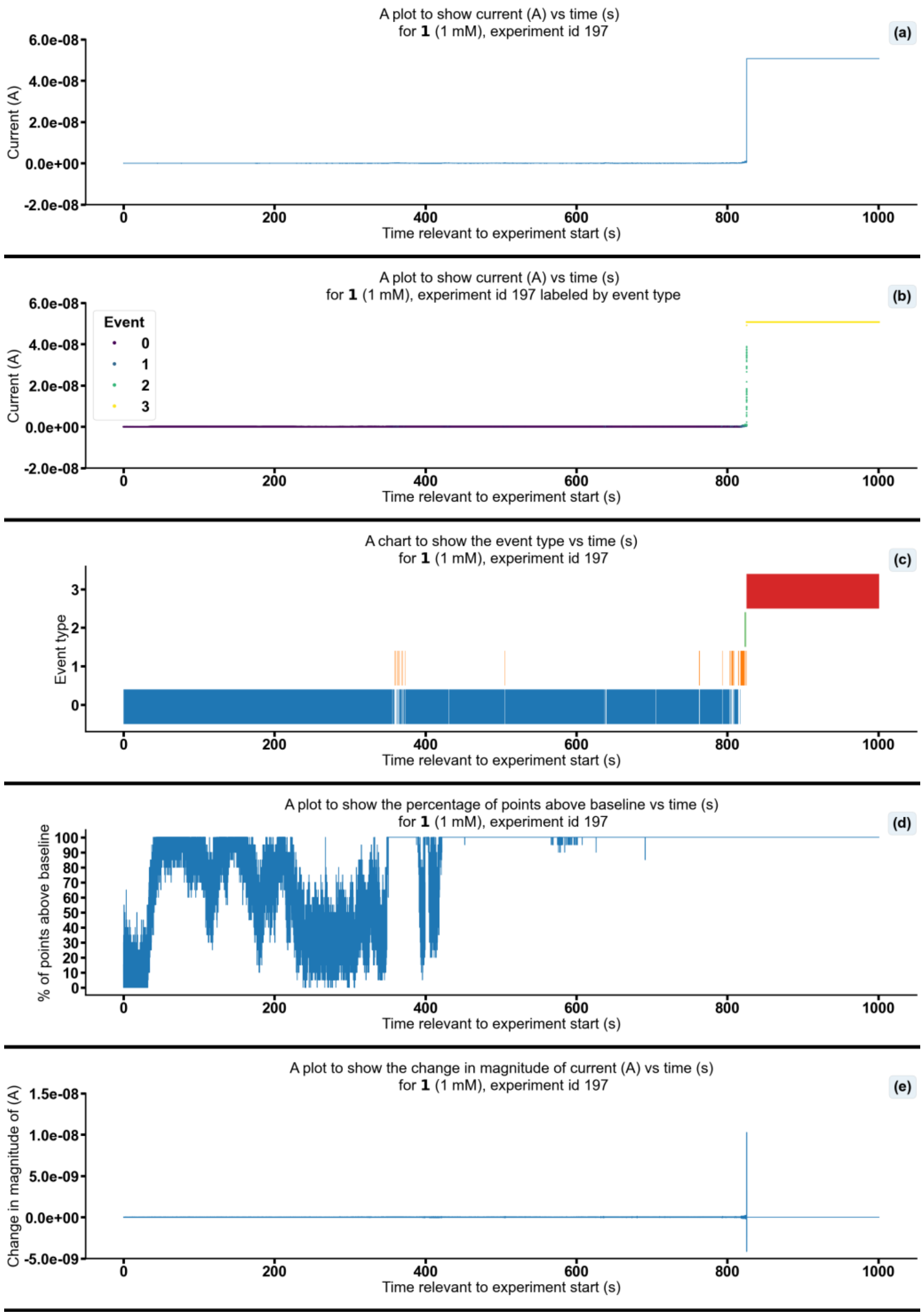

Figure S180 - Summarising the data from the patch clamp experiment for **1** (1 mM), experiment id 197 at +100 mV. Events were categorised into the following types: 0: -∞ A ≤ Event 0 < 5.00e-11 A, 1: 5.00e-11 A ≤ Event 1 < 5.00e-10 A, 2: 5.00e-10 A ≤ Event 2 < 4.90e-08 A, 3: 4.90e-08 A ≤ Event 3 < ∞ A. Subfigure (d) shows the percentage of points found above the threshold -1.1e-11 (A) in a rolling look ahead window of 20 points. The threshold is the mean + standard deviation of the noise of the first 200 datapoints.

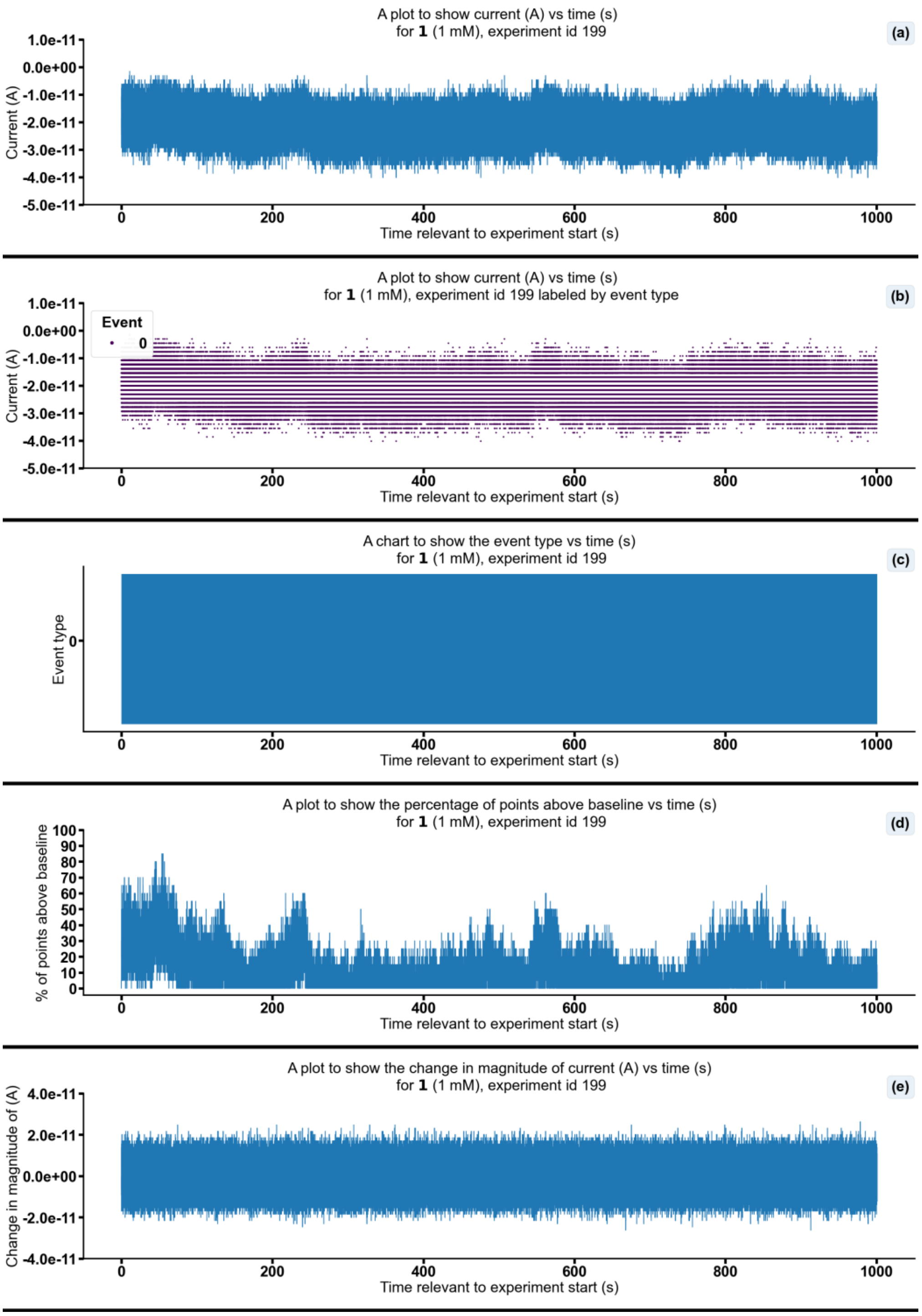

Figure S181 - Summarising the data from the patch clamp experiment for **1** (1 mM), experiment id 199 at +100 mV. Events were categorised into the following types: 0: -∞ A ≤ Event 0 < 5.00e-11 A, 1: 5.00e-11 A ≤ Event 1 < 5.00e-10 A, 2: 5.00e-10 A ≤ Event 2 < 4.90e-08 A, 3: 4.90e-08 A ≤ Event 3 < ∞ A. Subfigure (d) shows the percentage of points found above the threshold -1.6e-11 (A) in a rolling look ahead window of 20 points. The threshold is the mean + standard deviation of the noise of the first 200 datapoints.

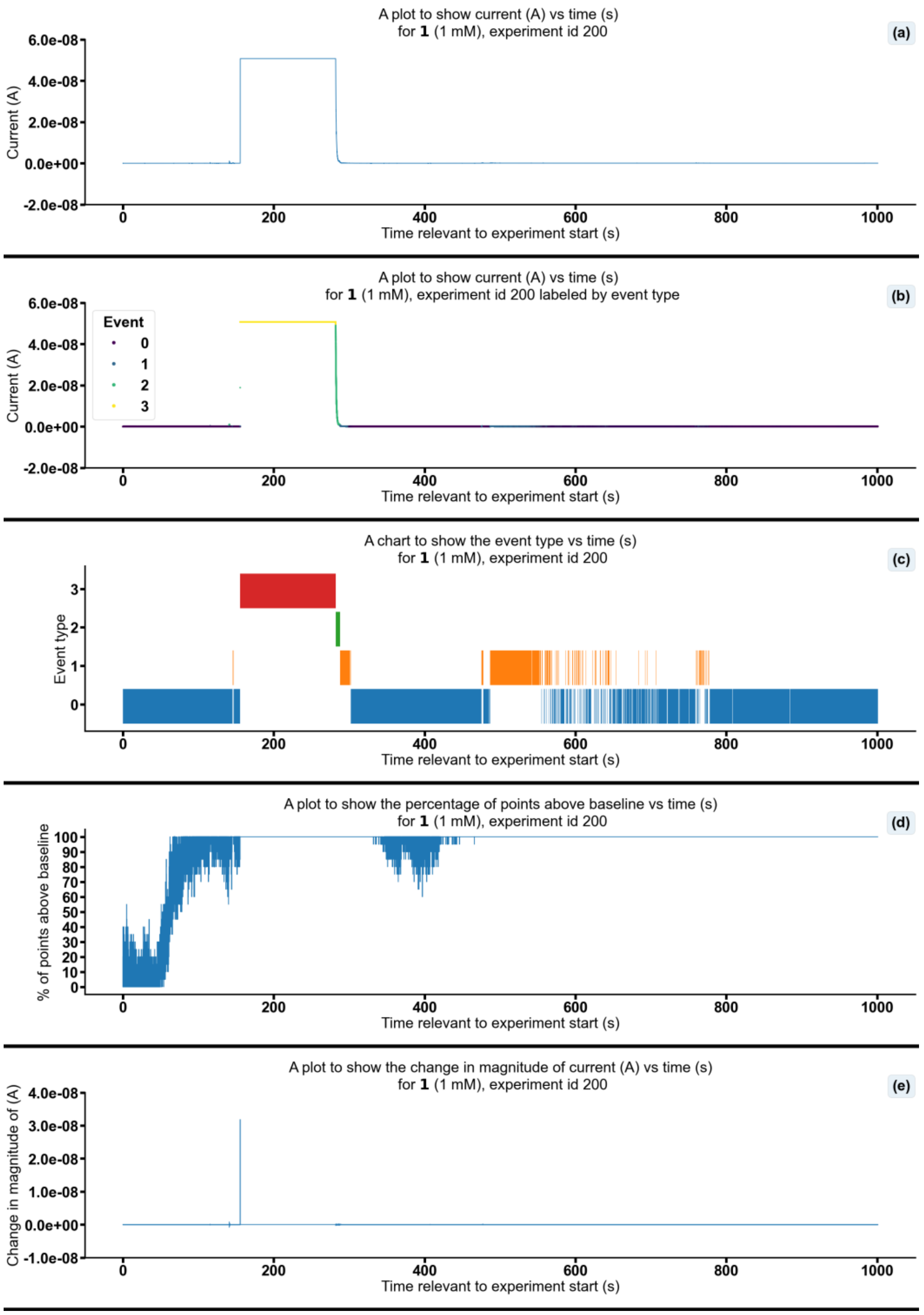

Figure S182 - Summarising the data from the patch clamp experiment for **1** (1 mM), experiment id 200 at +100 mV. Events were categorised into the following types: 0: -∞ A ≤ Event 0 < 5.00e-11 A, 1: 5.00e-11 A ≤ Event 1 < 5.00e-10 A, 2: 5.00e-10 A ≤ Event 2 < 4.90e-08 A, 3: 4.90e-08 A ≤ Event 3 < ∞ A Subfigure (d) shows the percentage of points found above the threshold -4.4e-12 (A) in a rolling look ahead window of 20 points. The threshold is the mean + standard deviation of the noise of the first 200 datapoints.

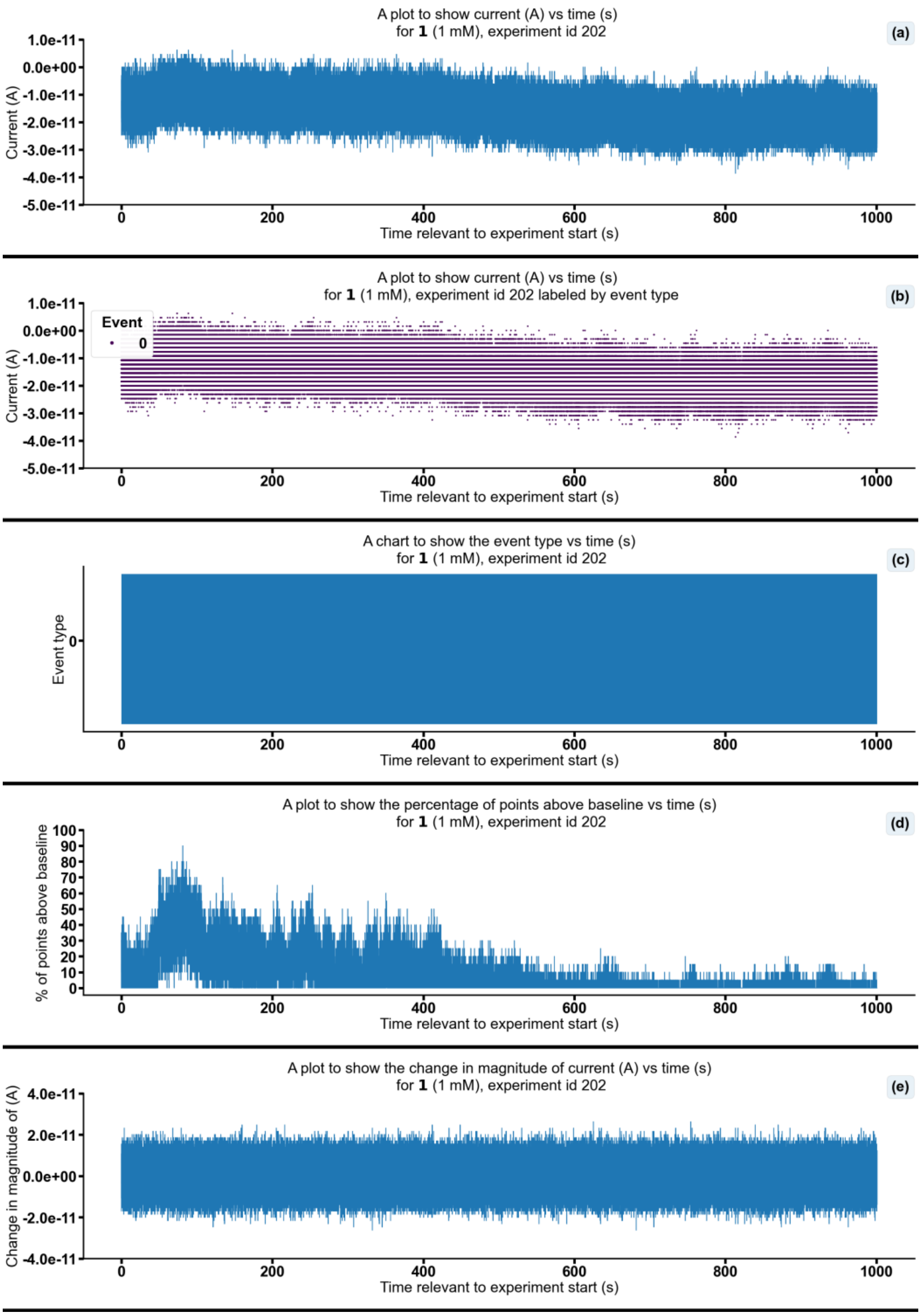

Figure S183 - Summarising the data from the patch clamp experiment for **1** (1 mM), experiment id 202 at +100 mV. Events were categorised into the following types: 0: -∞ A ≤ Event 0 < 5.00e-11 A, 1: 5.00e-11 A ≤ Event 1 < 5.00e-10 A, 2: 5.00e-10 A ≤ Event 2 < 4.90e-08 A, 3: 4.90e-08 A ≤ Event 3 < ∞ A. Subfigure (d) shows the percentage of points found above the threshold -9.1e-12 (A) in a rolling look ahead window of 20 points. The threshold is the mean + standard deviation of the noise of the first 200 datapoints.

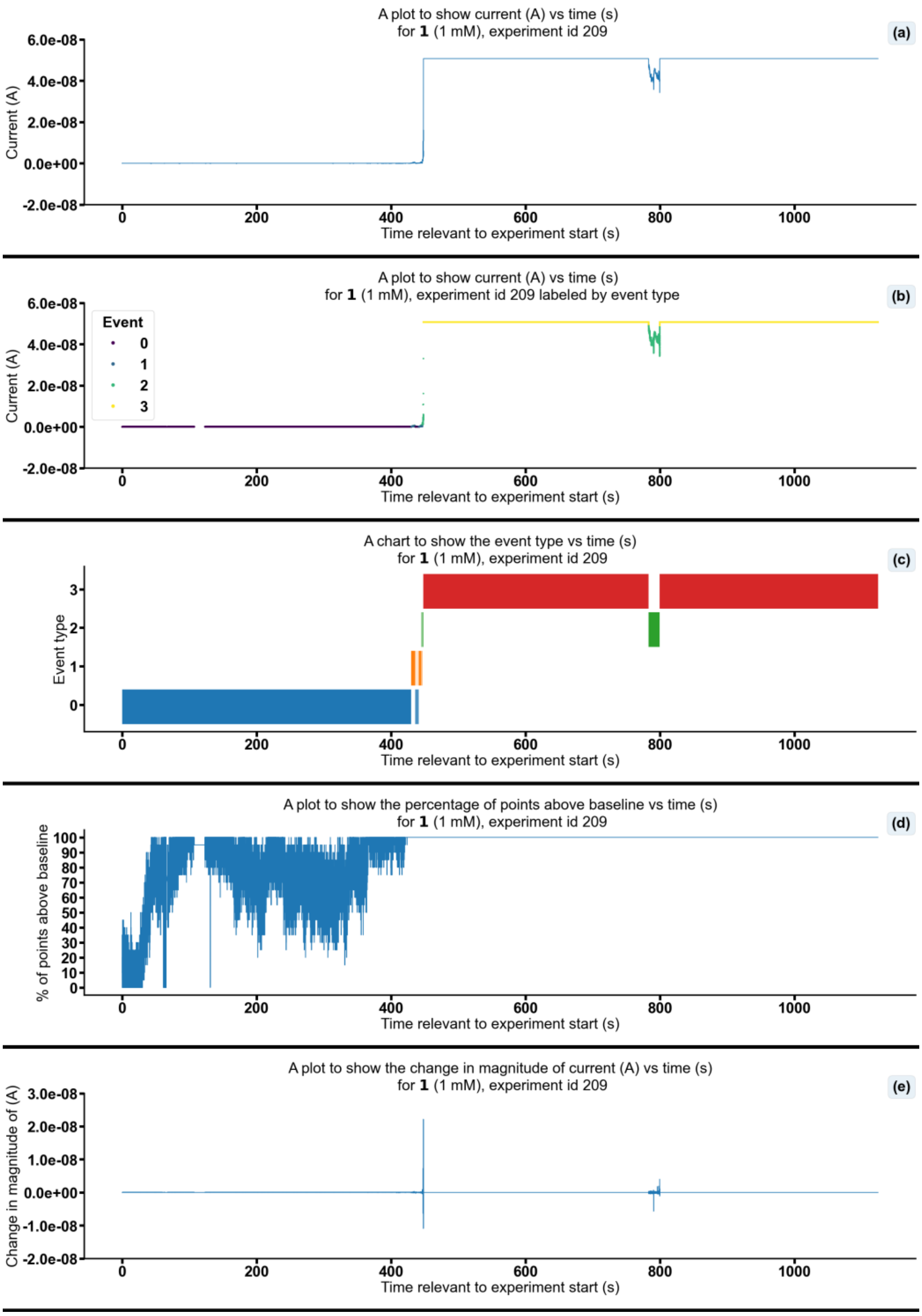

Figure S184 - Summarising the data from the patch clamp experiment for **1** (1 mM), experiment id 209 at +100 mV. Events were categorised into the following types: 0: -∞ A ≤ Event 0 < 5.00e-11 A, 1: 5.00e-11 A ≤ Event 1 < 5.00e-10 A, 2: 5.00e-10 A ≤ Event 2 < 4.90e-08 A, 3: 4.90e-08 A ≤ Event 3 < ∞ A. Subfigure (d) shows the percentage of points found above the threshold -4.2e-12 (A) in a rolling look ahead window of 20 points. The threshold is the mean + standard deviation of the noise of the first 200 datapoints.

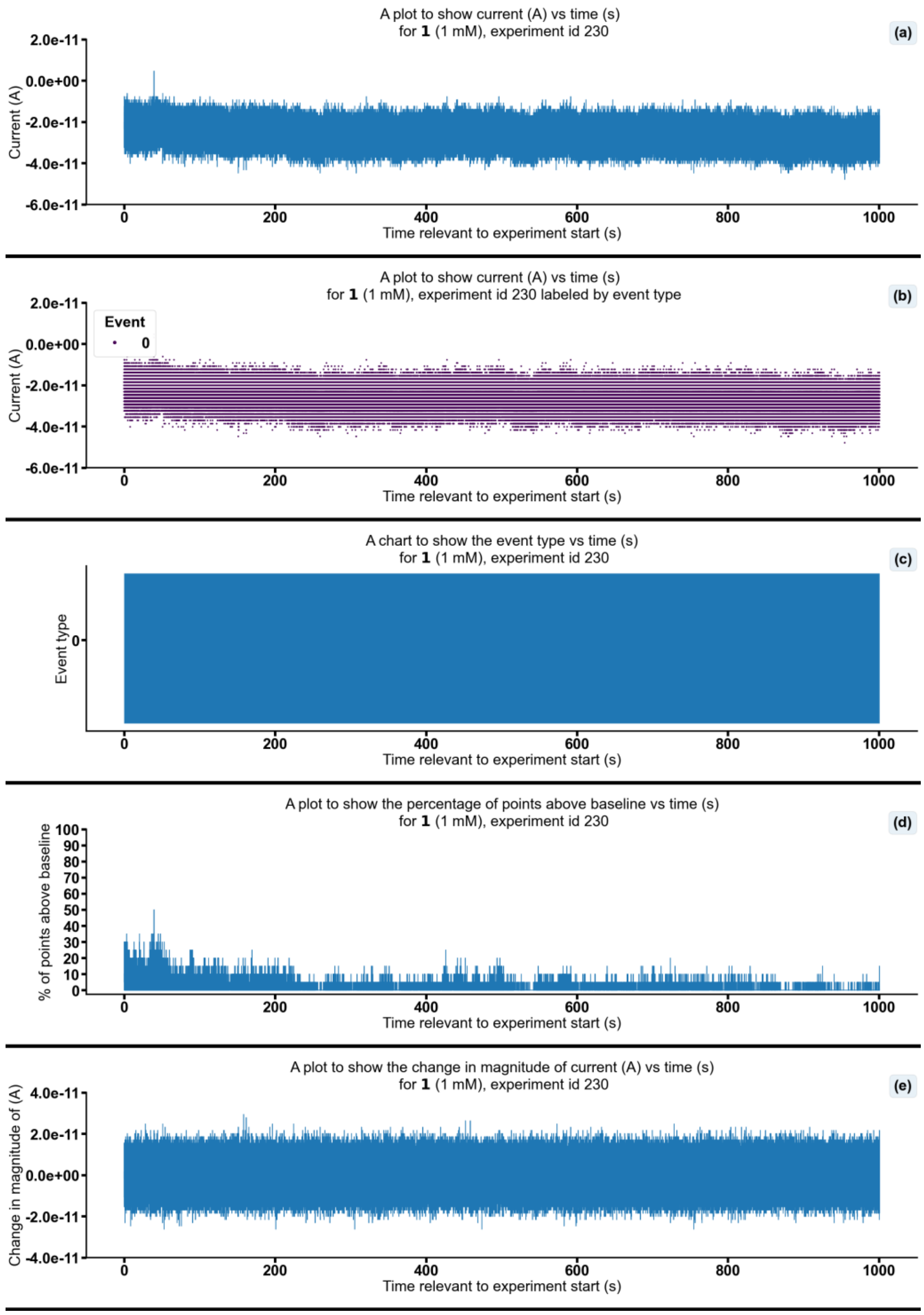

Figure S185 - Summarising the data from the patch clamp experiment for **1** (1 mM), experiment id 230 at +100 mV. Events were categorised into the following types: 0: -∞ A ≤ Event 0 < 5.00e-11 A, 1: 5.00e-11 A ≤ Event 1 < 5.00e-10 A, 2: 5.00e-10 A ≤ Event 2 < 4.90e-08 A, 3: 4.90e-08 A ≤ Event 3 < ∞ A. Subfigure (d) shows the percentage of points found above the threshold -1.7e-11 (A) in a rolling look ahead window of 20 points. The threshold is the mean + standard deviation of the noise of the first 200 datapoints.

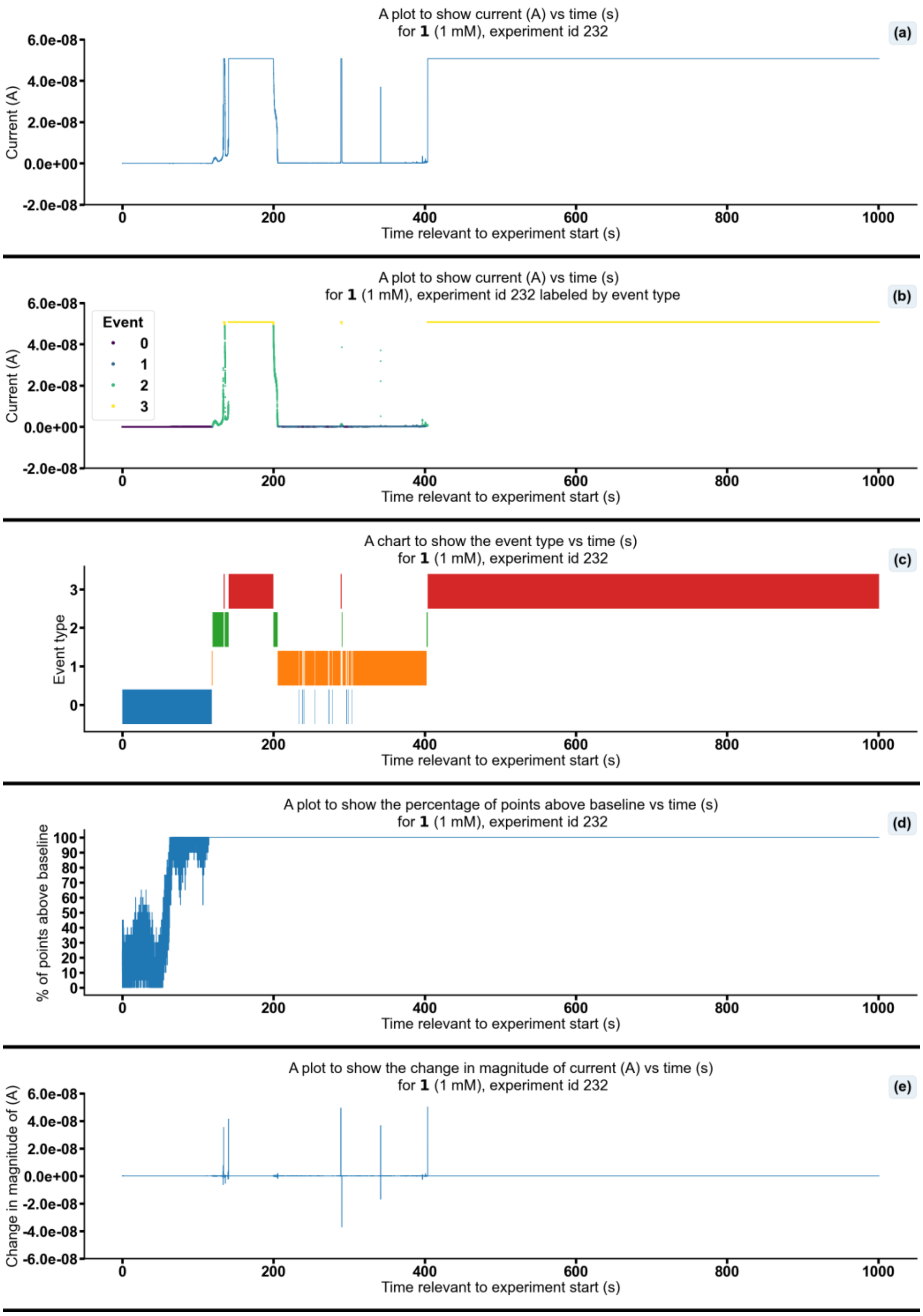

Figure S186 - Summarising the data from the patch clamp experiment for **1** (1 mM), experiment id 232 at +100 mV. Events were categorised into the following types: 0: -∞ A ≤ Event 0 < 5.00e-11 A, 1: 5.00e-11 A ≤ Event 1 < 5.00e-10 A, 2: 5.00e-10 A ≤ Event 2 < 4.90e-08 A, 3: 4.90e-08 A ≤ Event 3 < ∞ A. Subfigure (d) shows the percentage of points found above the threshold -1.6e-11 (A) in a rolling look ahead window of 20 points. The threshold is the mean + standard deviation of the noise of the first 200 datapoints.

Changing concentration of **3**

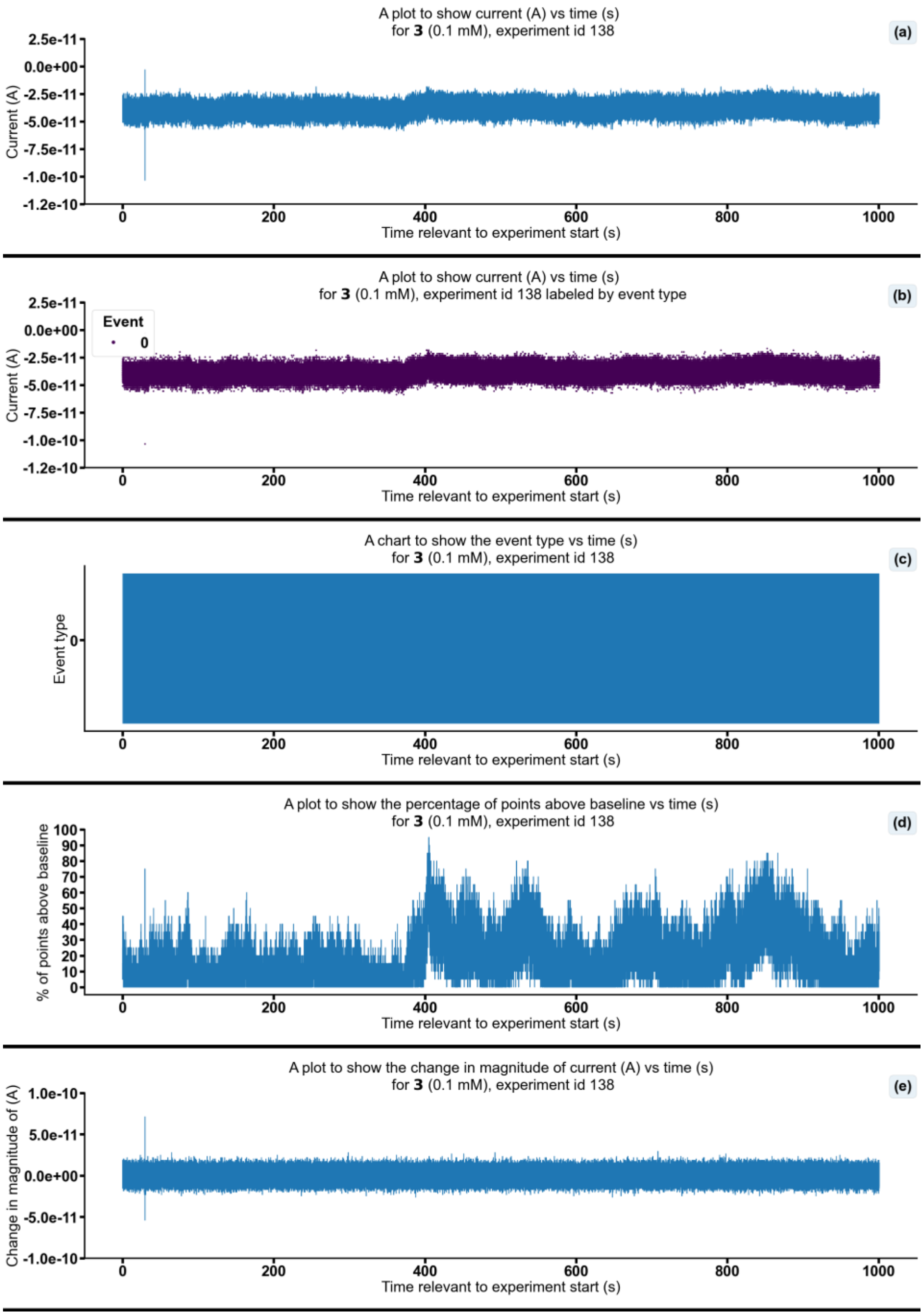

Figure S187 - Summarising the data from the patch clamp experiment for **3** (0.1 mM), experiment id 138 at +100 mV. Events were categorised into the following types: 0: -∞ A ≤ Event 0 < 5.00e-11 A, 1: 5.00e-11 A ≤ Event 1 < 5.00e-10 A, 2: 5.00e-10 A ≤ Event 2 < 4.90e-08 A, 3: 4.90e-08 A ≤ Event 3 < ∞ A. Subfigure (d) shows the percentage of points found above the threshold -3.5e-11 (A) in a rolling look ahead window of 20 points. The threshold is the mean + standard deviation of the noise of the first 200 datapoints.

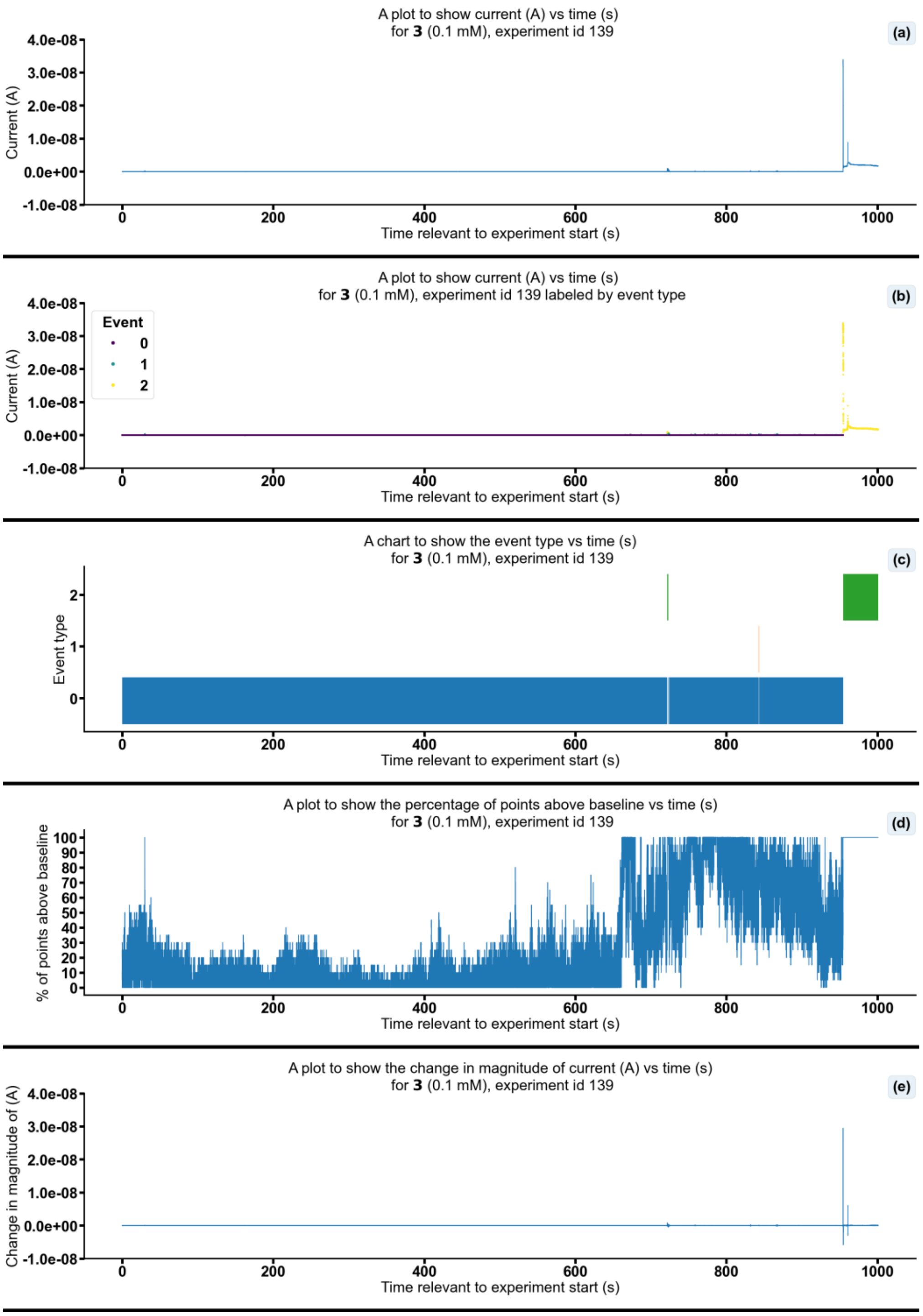

Figure S188 - Summarising the data from the patch clamp experiment for **3** (0.1 mM), experiment id 139 at +100 mV. Events were categorised into the following types: 0: -∞ A ≤ Event 0 < 5.00e-11 A, 1: 5.00e-11 A ≤ Event 1 < 5.00e-10 A, 2: 5.00e-10 A ≤ Event 2 < 4.90e-08 A, 3: 4.90e-08 A ≤ Event 3 < ∞ A. Subfigure (d) shows the percentage of points found above the threshold -1.2e-11 (A) in a rolling look ahead window of 20 points. The threshold is the mean + standard deviation of the noise of the first 200 datapoints.

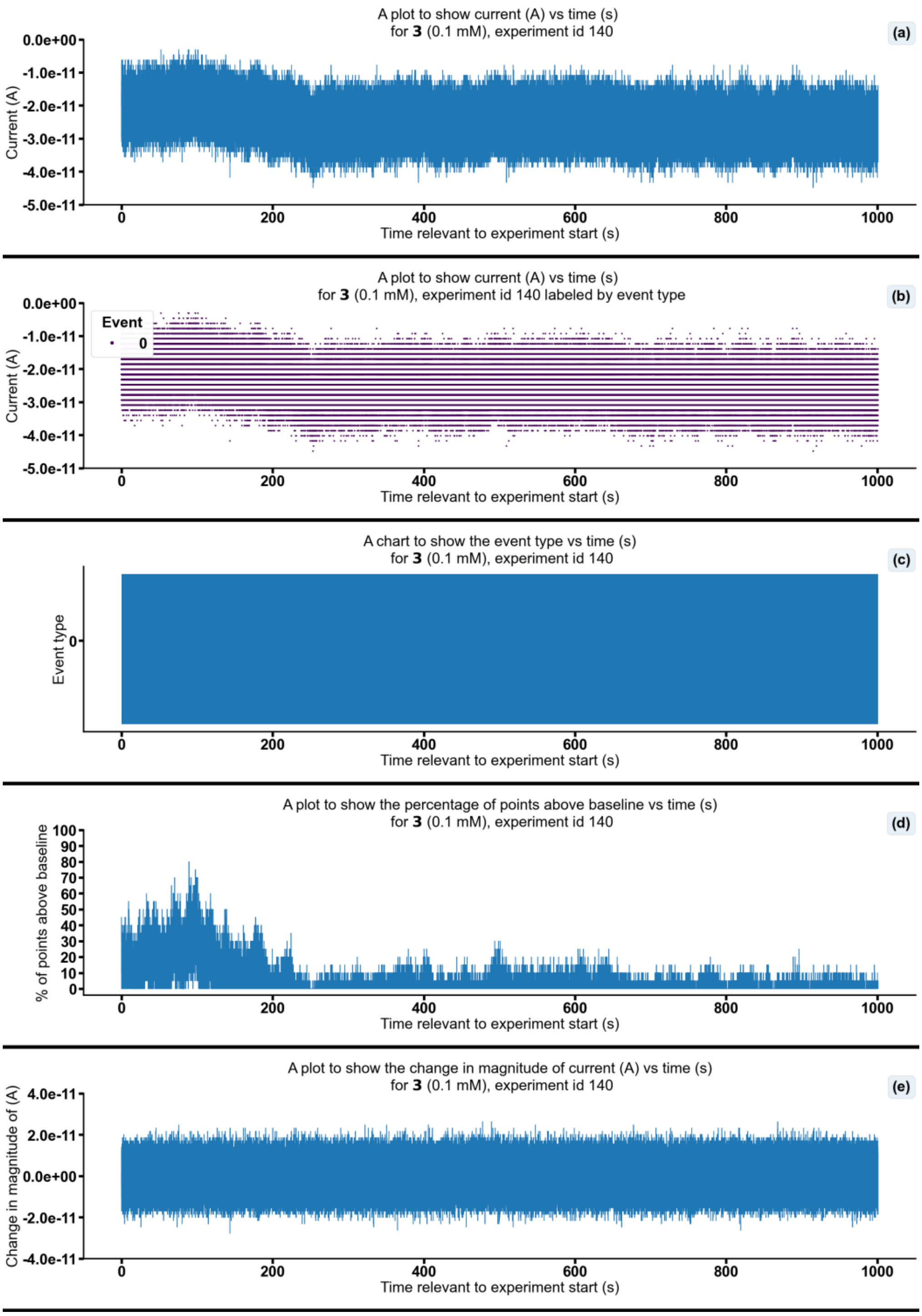

Figure S189 - Summarising the data from the patch clamp experiment for **3** (0.1 mM), experiment id 140 at +100 mV. Events were categorised into the following types: 0: -∞ A ≤ Event 0 < 5.00e-11 A, 1: 5.00e-11 A ≤ Event 1 < 5.00e-10 A, 2: 5.00e-10 A ≤ Event 2 < 4.90e-08 A, 3: 4.90e-08 A ≤ Event 3 < ∞ A. Subfigure (d) shows the percentage of points found above the threshold -1.6e-11 (A) in a rolling look ahead window of 20 points. The threshold is the mean + standard deviation of the noise of the first 200 datapoints.

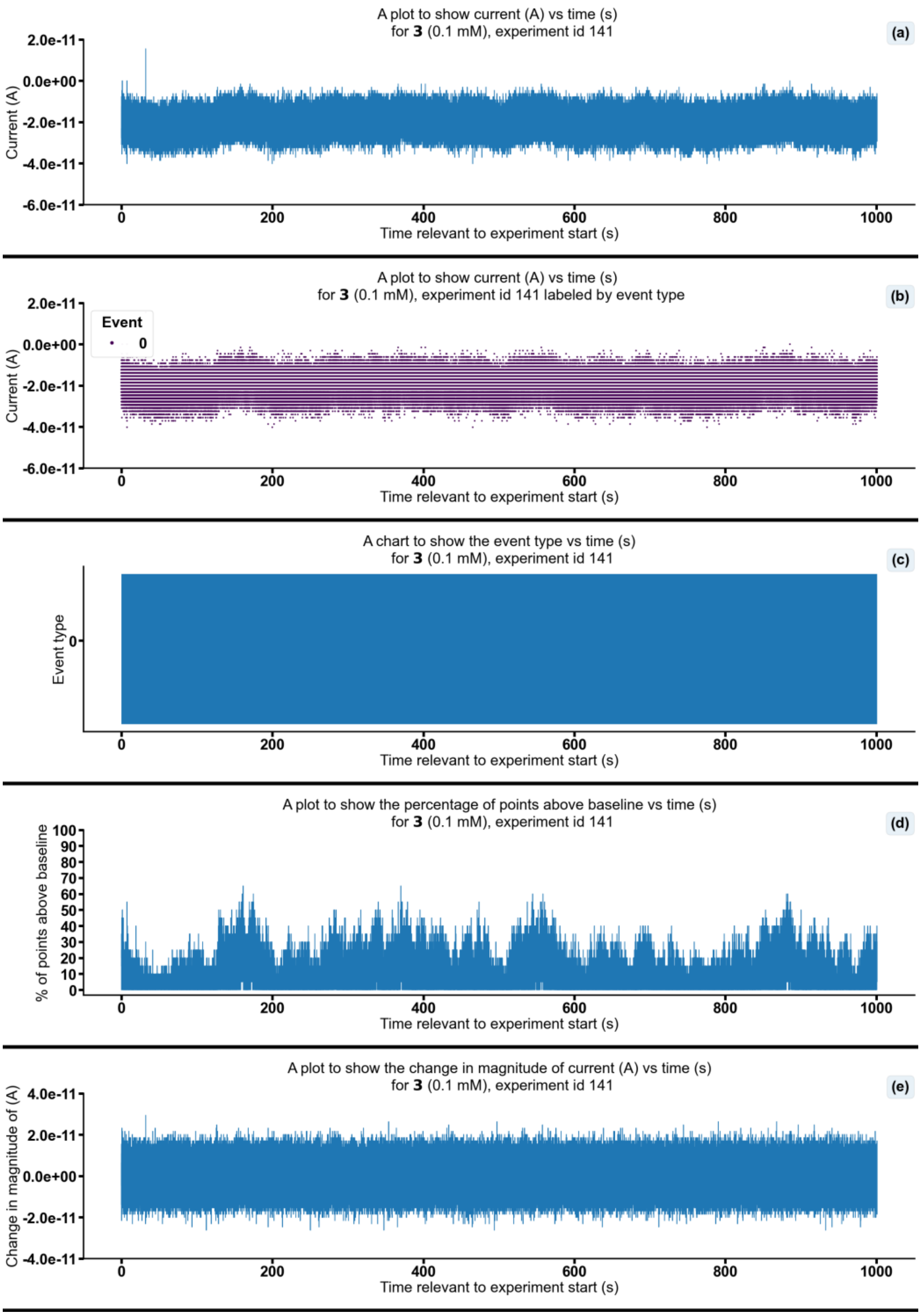

Figure S190 - Summarising the data from the patch clamp experiment for **3** (0.1 mM), experiment id 141 at +100 mV. Events were categorised into the following types: 0: -∞ A ≤ Event 0 < 5.00e-11 A, 1: 5.00e-11 A ≤ Event 1 < 5.00e-10 A, 2: 5.00e-10 A ≤ Event 2 < 4.90e-08 A, 3: 4.90e-08 A ≤ Event 3 < ∞ A. Subfigure (d) shows the percentage of points found above the threshold -1.5e-11 (A) in a rolling look ahead window of 20 points. The threshold is the mean + standard deviation of the noise of the first 200 datapoints.

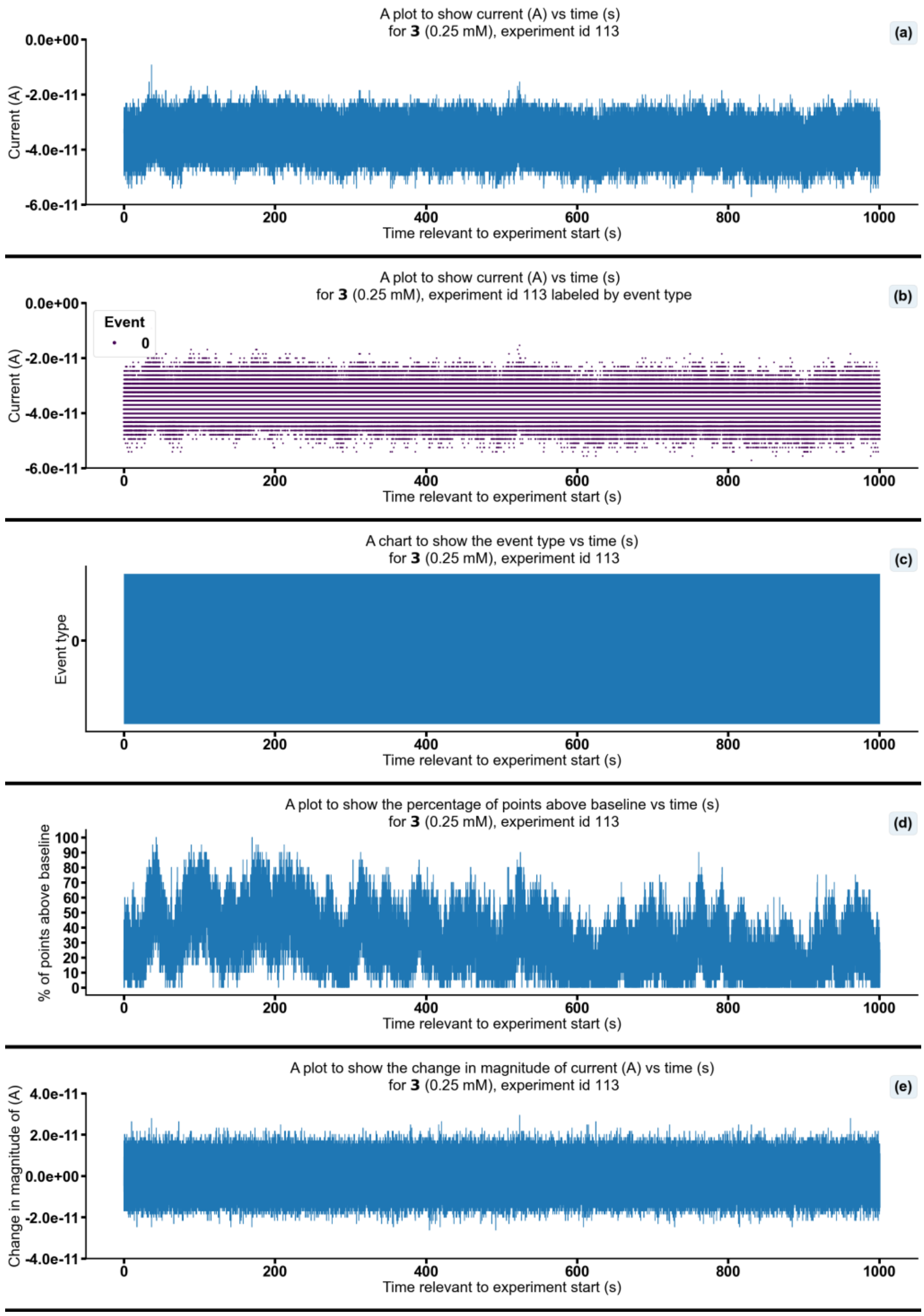

Figure S191 - Summarising the data from the patch clamp experiment for **3** (0.25 mM), experiment id 113 at +100 mV. Events were categorised into the following types: 0: -∞ A ≤ Event 0 < 5.00e-11 A, 1: 5.00e-11 A ≤ Event 1 < 5.00e-10 A, 2: 5.00e-10 A ≤ Event 2 < 4.90e-08 A, 3: 4.90e-08 A ≤ Event 3 < ∞ A. Subfigure (d) shows the percentage of points found above the threshold -3.5e-11 (A) in a rolling look ahead window of 20 points. The threshold is the mean + standard deviation of the noise of the first 200 datapoints.

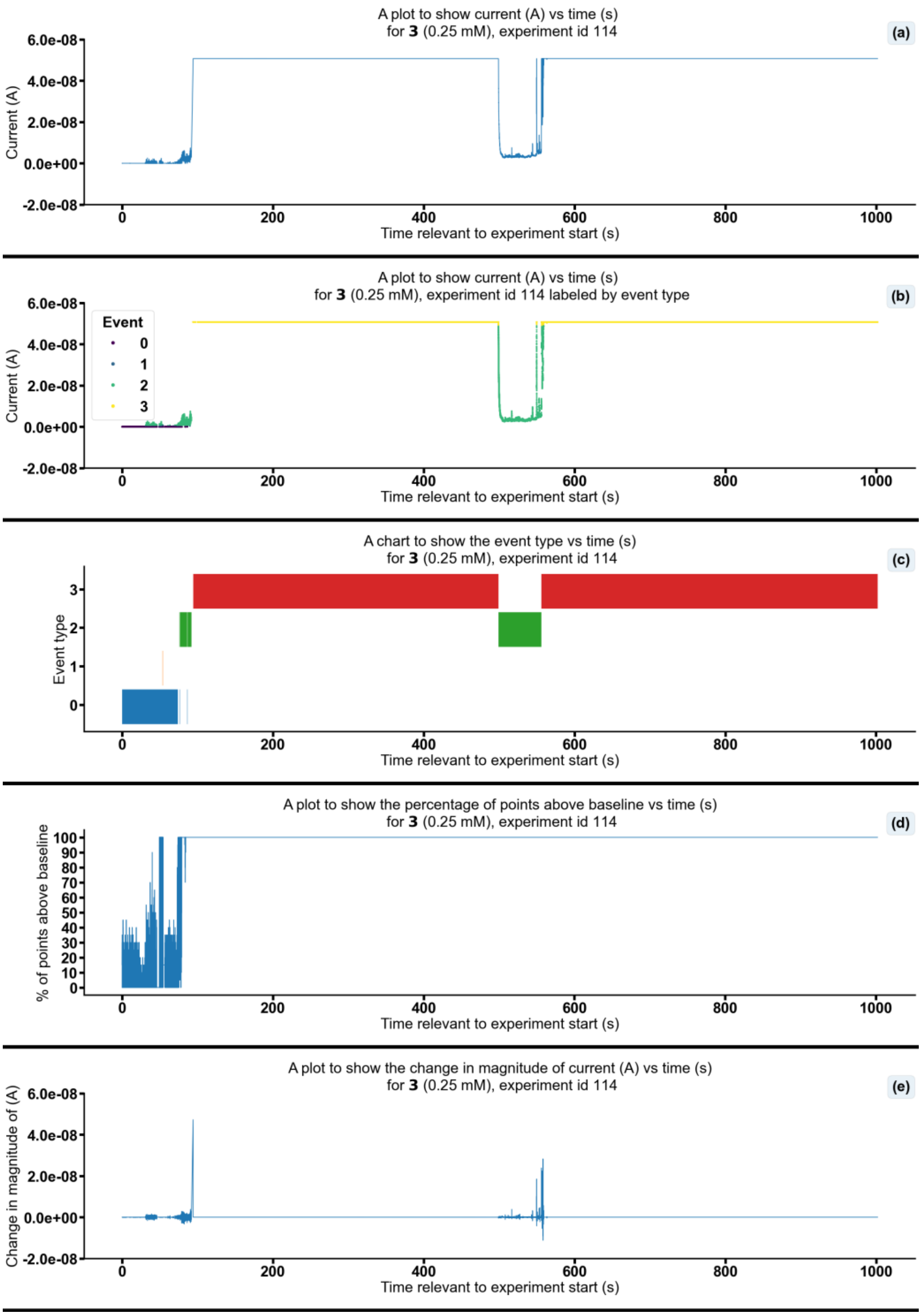

Figure S192 - Summarising the data from the patch clamp experiment for **3** (0.25 mM), experiment id 114 at +100 mV. Events were categorised into the following types: 0: -∞ A ≤ Event 0 < 5.00e-11 A, 1: 5.00e-11 A ≤ Event 1 < 5.00e-10 A, 2: 5.00e-10 A ≤ Event 2 < 4.90e-08 A, 3: 4.90e-08 A ≤ Event 3 < ∞ A. Subfigure (d) shows the percentage of points found above the threshold 5.6e-12 (A) in a rolling look ahead window of 20 points. The threshold is the mean + standard deviation of the noise of the first 200 datapoints.

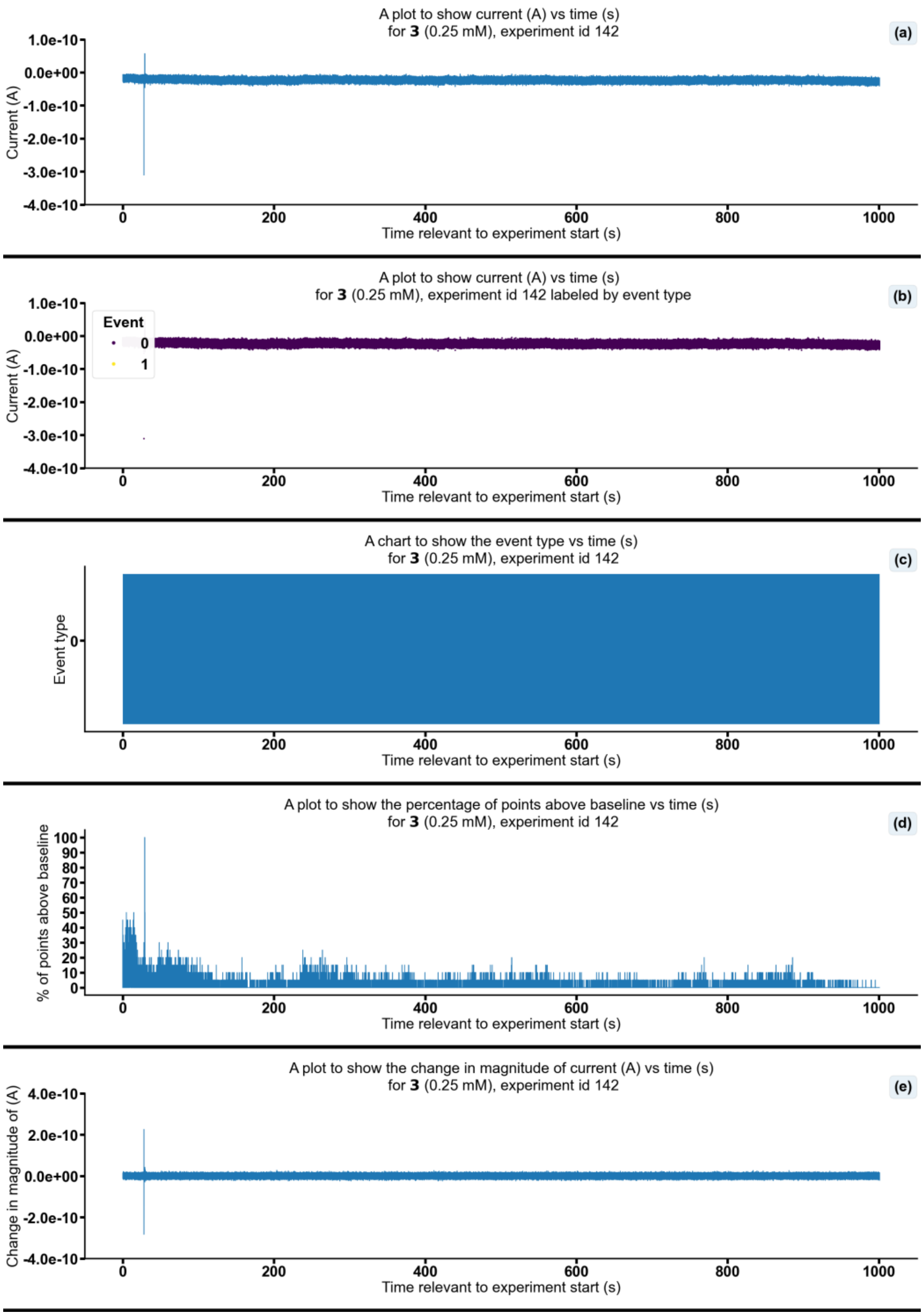

Figure S193 - Summarising the data from the patch clamp experiment for **3** (0.25 mM), experiment id 142 at +100 mV. Events were categorised into the following types: 0: -∞ A ≤ Event 0 < 5.00e-11 A, 1: 5.00e-11 A ≤ Event 1 < 5.00e-10 A, 2: 5.00e-10 A ≤ Event 2 < 4.90e-08 A, 3: 4.90e-08 A ≤ Event 3 < ∞ A. Subfigure (d) shows the percentage of points found above the threshold -1.4e-11 (A) in a rolling look ahead window of 20 points. The threshold is the mean + standard deviation of the noise of the first 200 datapoints.

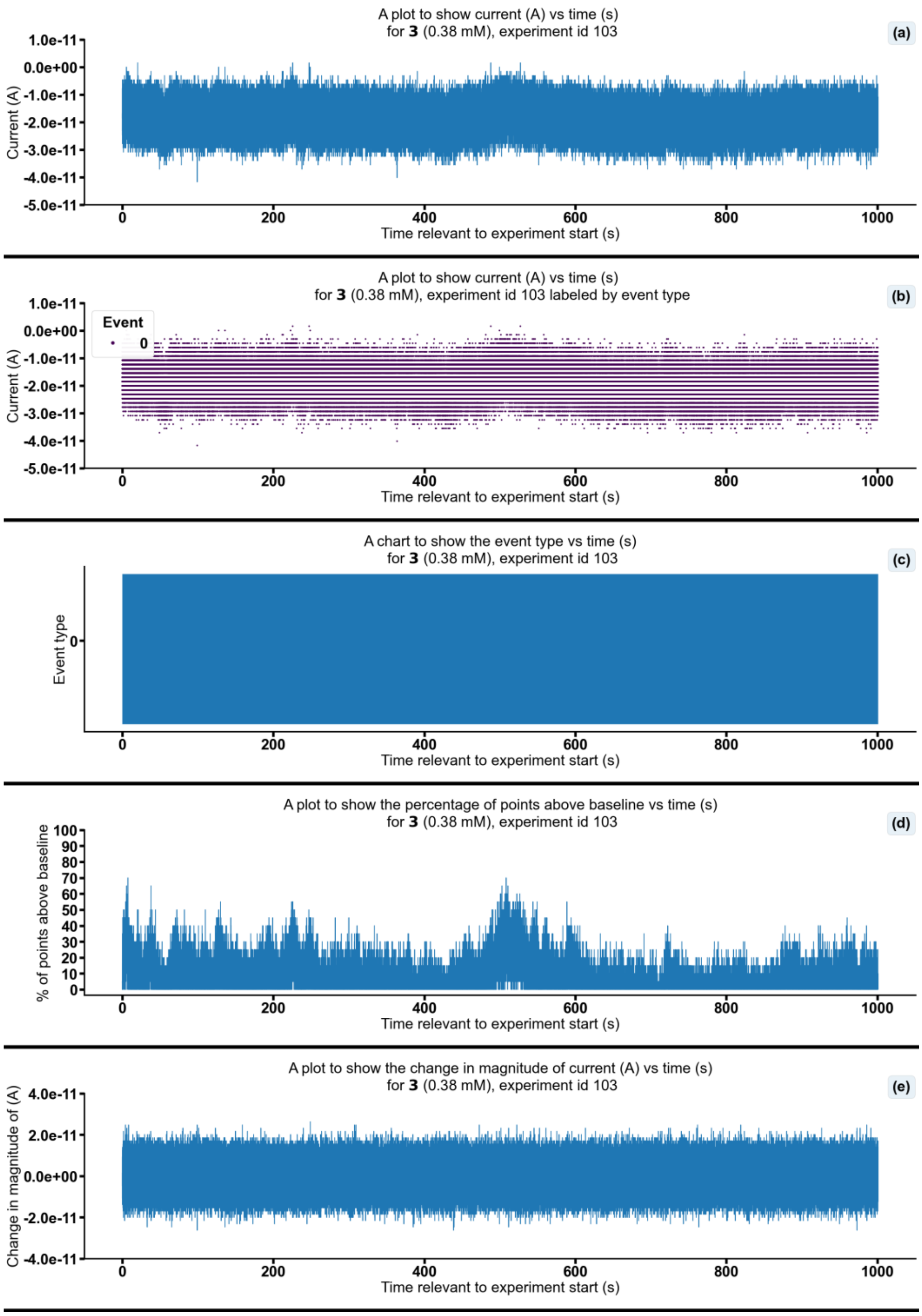

Figure S194 - Summarising the data from the patch clamp experiment for **3** (0.38 mM), experiment id 103 at +100 mV. Events were categorised into the following types: 0: -∞ A ≤ Event 0 < 5.00e-11 A, 1: 5.00e-11 A ≤ Event 1 < 5.00e-10 A, 2: 5.00e-10 A ≤ Event 2 < 4.90e-08 A, 3: 4.90e-08 A ≤ Event 3 < ∞ A. Subfigure (d) shows the percentage of points found above the threshold -1.3e-11 (A) in a rolling look ahead window of 20 points. The threshold is the mean + standard deviation of the noise of the first 200 datapoints.

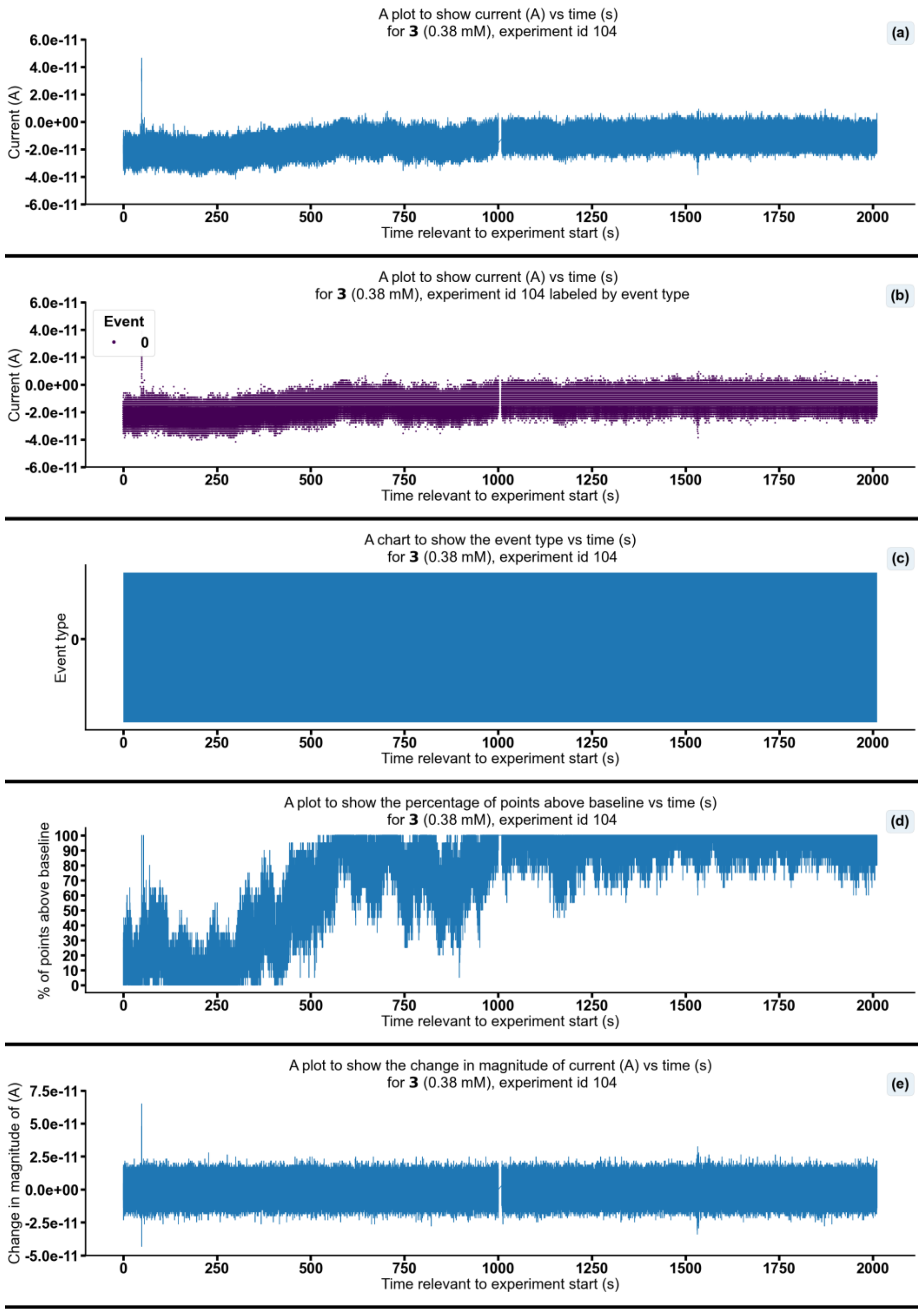

Figure S195 - Summarising the data from the patch clamp experiment for **3** (0.38 mM), experiment id 104 at +100 mV. Events were categorised into the following types: 0: -∞ A ≤ Event 0 < 5.00e-11 A, 1: 5.00e-11 A ≤ Event 1 < 5.00e-10 A, 2: 5.00e-10 A ≤ Event 2 < 4.90e-08 A, 3: 4.90e-08 A ≤ Event 3 < ∞ A. Subfigure (d) shows the percentage of points found above the threshold -1.8e-11 (A) in a rolling look ahead window of 20 points. The threshold is the mean + standard deviation of the noise of the first 200 datapoints.

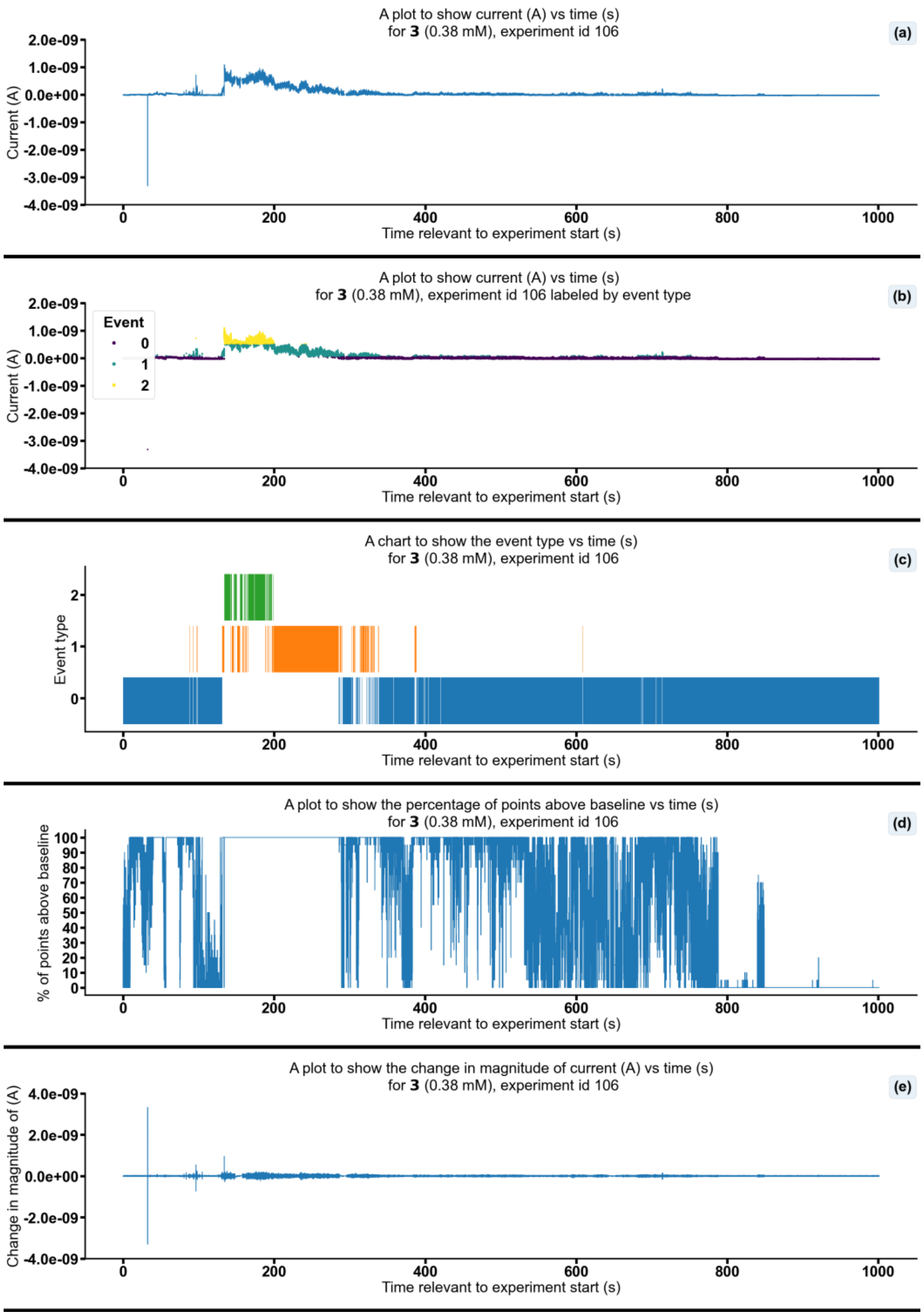

Figure S196 - Summarising the data from the patch clamp experiment for **3** (0.38 mM), experiment id 106 at +100 mV. Events were categorised into the following types: 0: -∞ A ≤ Event 0 < 5.00e-11 A, 1: 5.00e-11 A ≤ Event 1 < 5.00e-10 A, 2: 5.00e-10 A ≤ Event 2 < 4.90e-08 A, 3: 4.90e-08 A ≤ Event 3 < ∞ A. Subfigure (d) shows the percentage of points found above the threshold -1.4e-11 (A) in a rolling look ahead window of 20 points. The threshold is the mean + standard deviation of the noise of the first 200 datapoints.

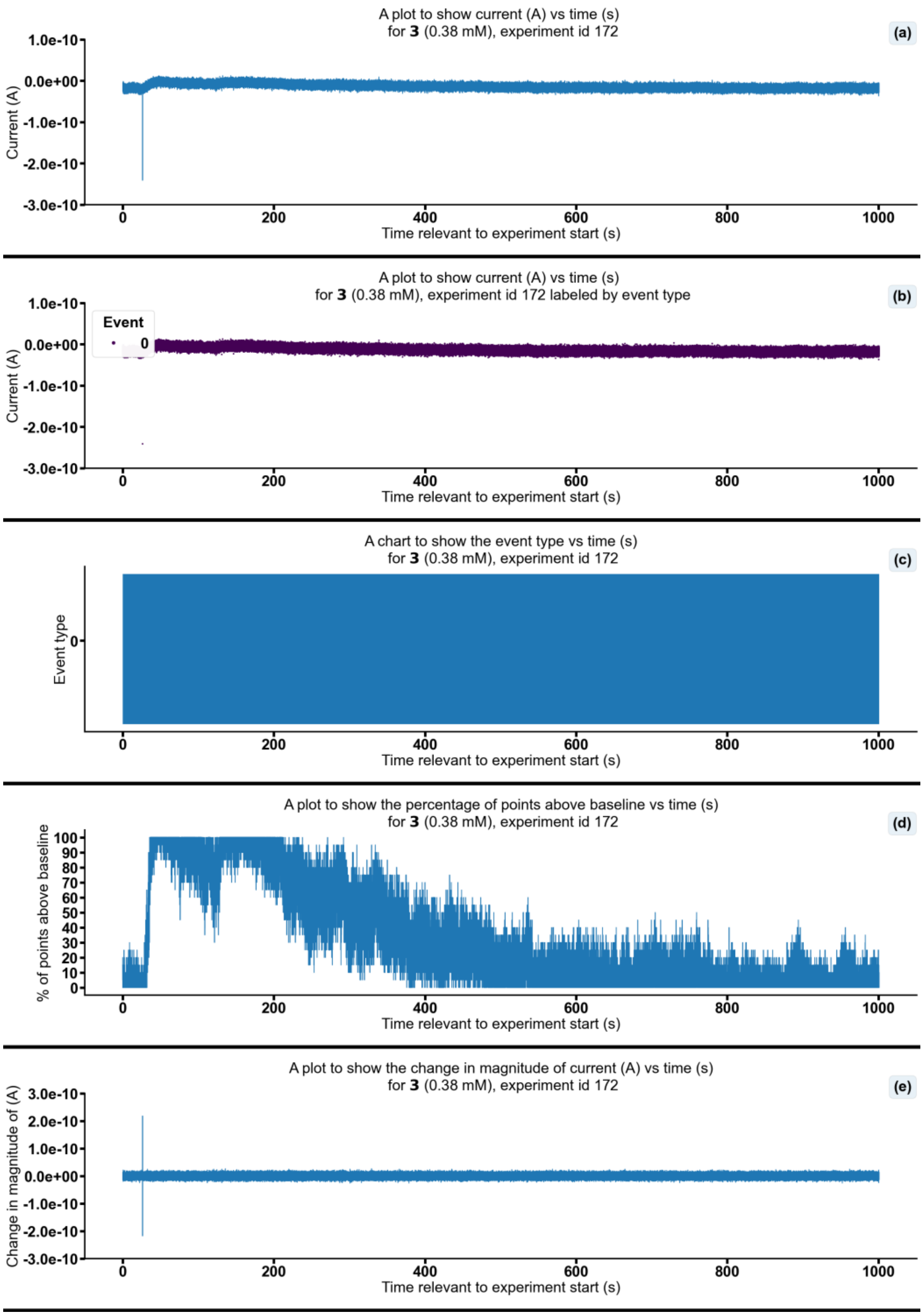

Figure S197 - Summarising the data from the patch clamp experiment for **3** (0.38 mM), experiment id 172 at +100 mV. Events were categorised into the following types: 0: -∞ A ≤ Event 0 < 5.00e-11 A, 1: 5.00e-11 A ≤ Event 1 < 5.00e-10 A, 2: 5.00e-10 A ≤ Event 2 < 4.90e-08 A, 3: 4.90e-08 A ≤ Event 3 < ∞ A. Subfigure (d) shows the percentage of points found above the threshold -1.2e-11 (A) in a rolling look ahead window of 20 points. The threshold is the mean + standard deviation of the noise of the first 200 datapoints.

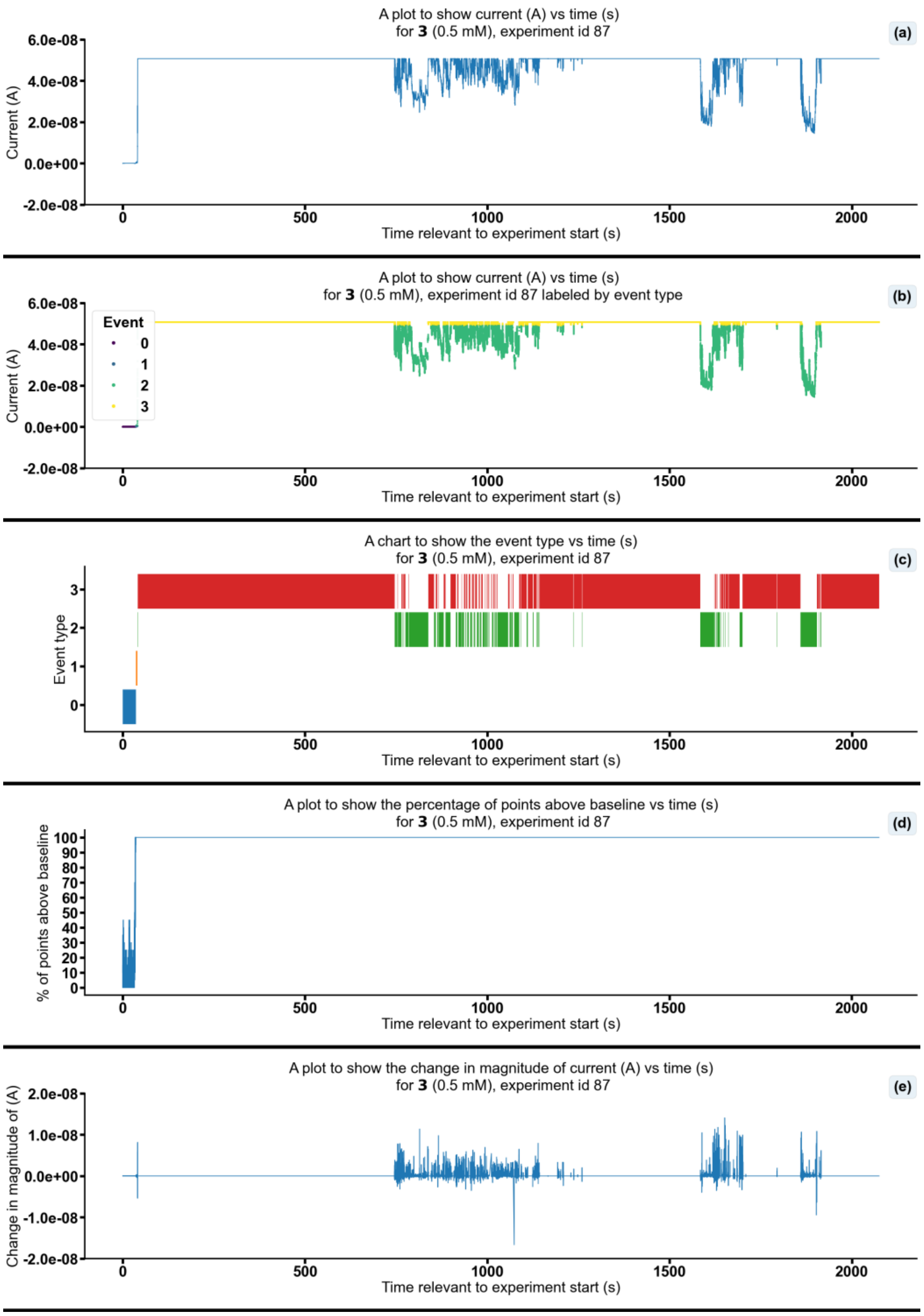

Figure S198 - Summarising the data from the patch clamp experiment for **3** (0.5 mM), experiment id 87 at +100 mV. Events were categorised into the following types: 0: -∞ A ≤ Event 0 < 5.00e-11 A, 1: 5.00e-11 A ≤ Event 1 < 5.00e-10 A, 2: 5.00e-10 A ≤ Event 2 < 4.90e-08 A, 3: 4.90e-08 A ≤ Event 3 < ∞ A. Subfigure (d) shows the percentage of points found above the threshold 1.2e-11 (A) in a rolling look ahead window of 20 points. The threshold is the mean + standard deviation of the noise of the first 200 datapoints.

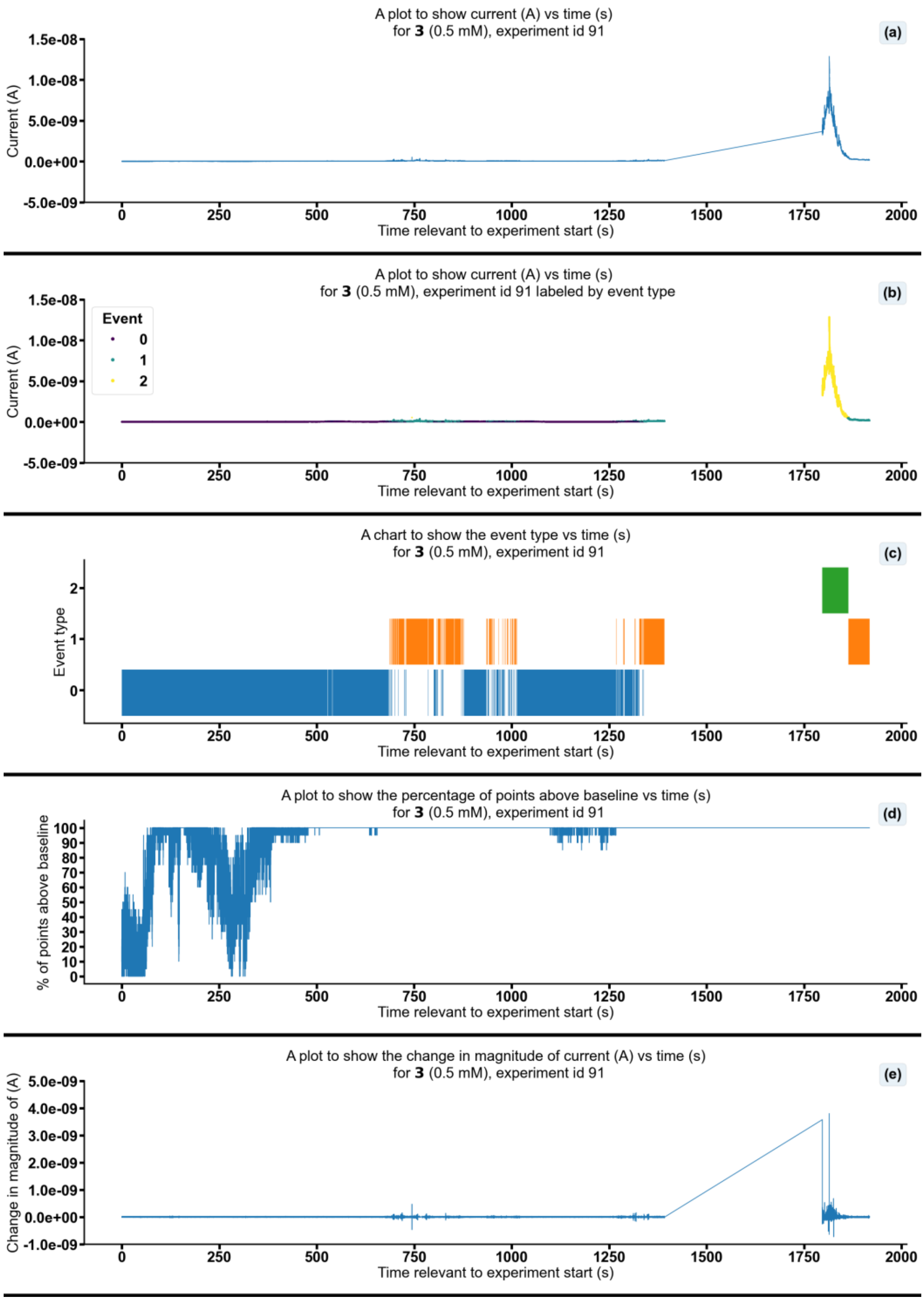

Figure S199 - Summarising the data from the patch clamp experiment for **3** (0.5 mM), experiment id 91 at +100 mV. Events were categorised into the following types: 0: -∞ A ≤ Event 0 < 5.00e-11 A, 1: 5.00e-11 A ≤ Event 1 < 5.00e-10 A, 2: 5.00e-10 A ≤ Event 2 < 4.90e-08 A, 3: 4.90e-08 A ≤ Event 3 < ∞ A. Subfigure (d) shows the percentage of points found above the threshold 1.0e-11 (A) in a rolling look ahead window of 20 points. The threshold is the mean + standard deviation of the noise of the first 200 datapoints.

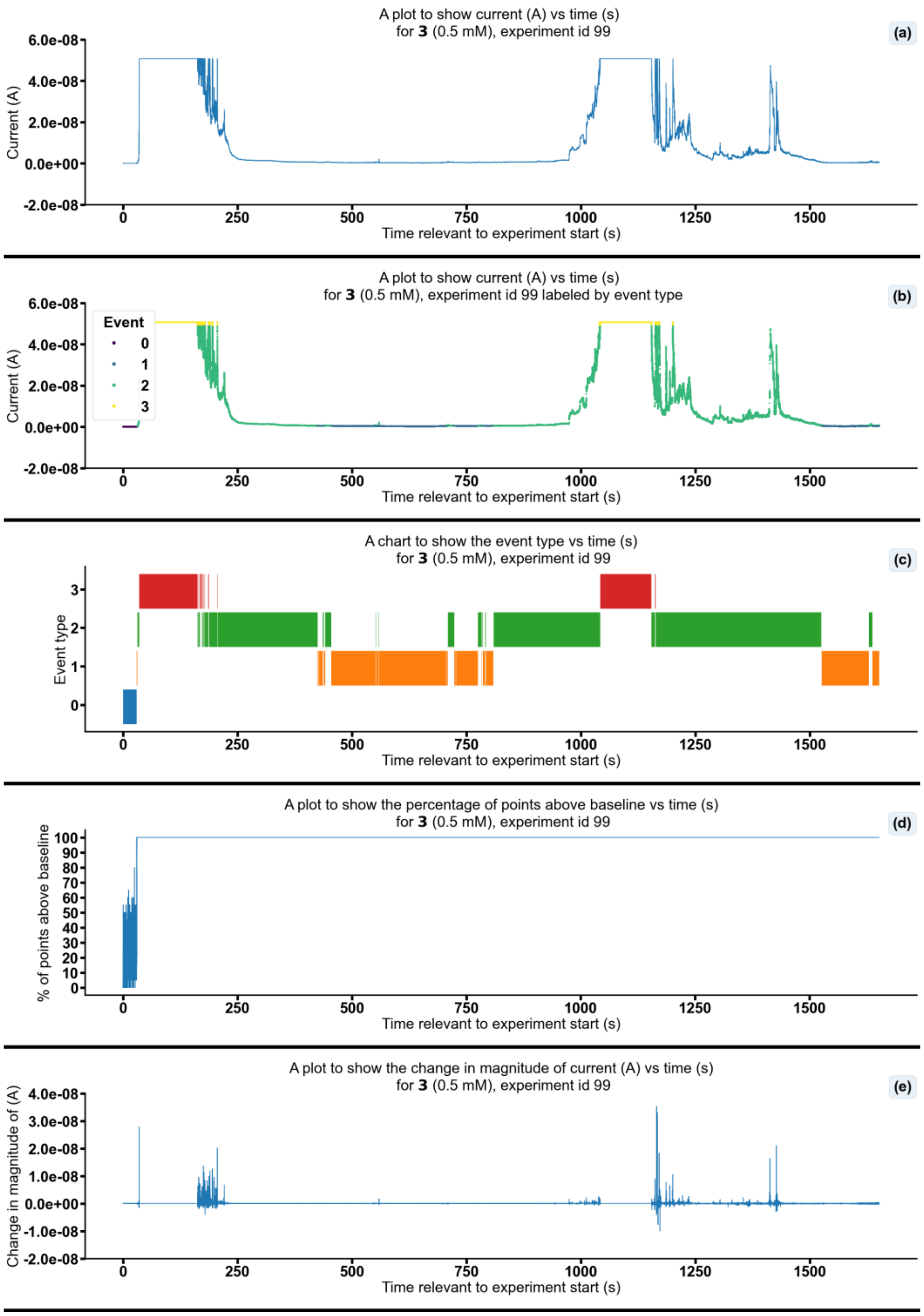

Figure S200 - Summarising the data from the patch clamp experiment for **3** (0.5 mM), experiment id 99 at +100 mV. Events were categorised into the following types: 0: -∞ A ≤ Event 0 < 5.00e-11 A, 1: 5.00e-11 A ≤ Event 1 < 5.00e-10 A, 2: 5.00e-10 A ≤ Event 2 < 4.90e-08 A, 3: 4.90e-08 A ≤ Event 3 < ∞ A. Subfigure (d) shows the percentage of points found above the threshold 1.7e-11 (A) in a rolling look ahead window of 20 points. The threshold is the mean + standard deviation of the noise of the first 200 datapoints.

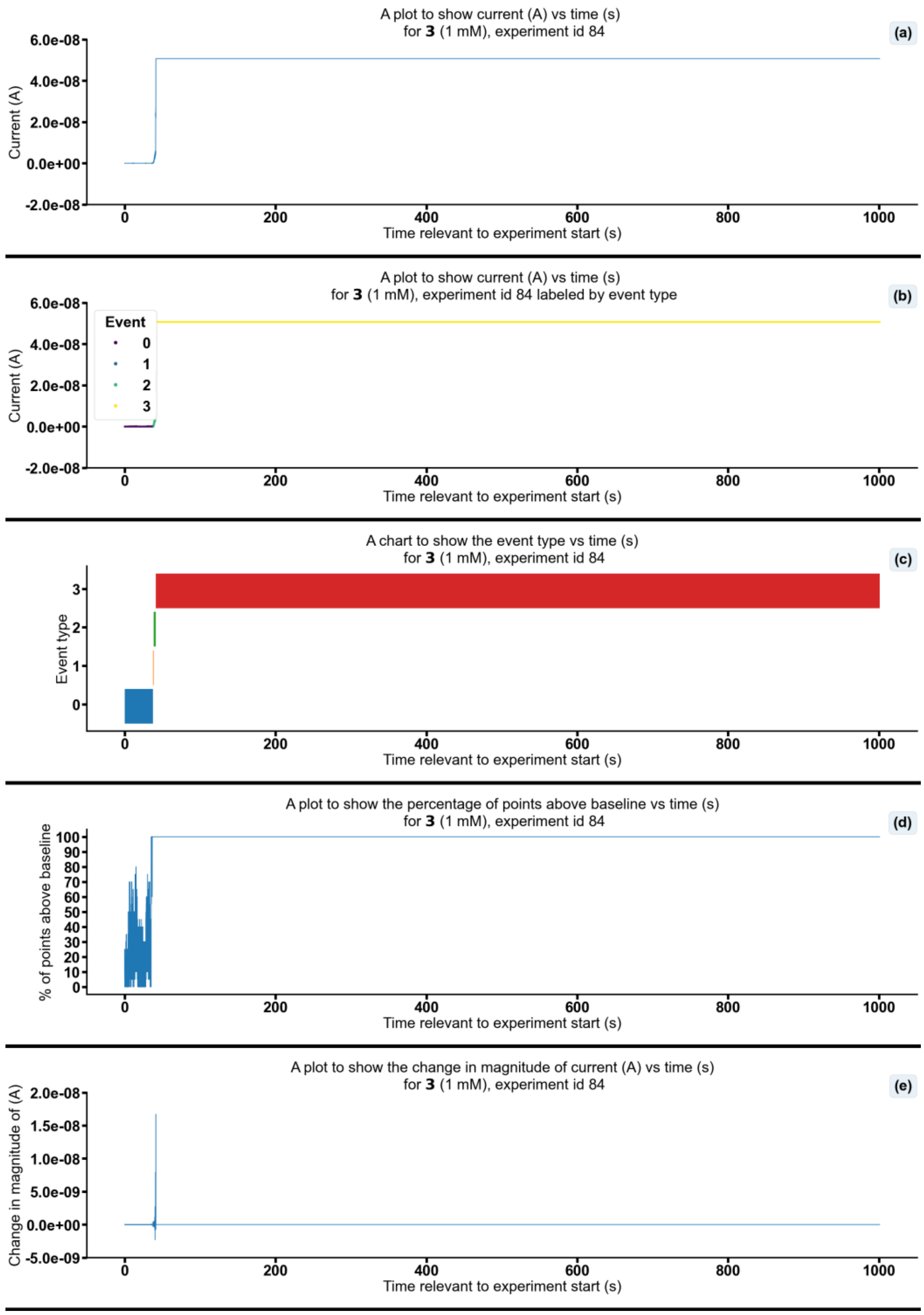

Figure S201 - Summarising the data from the patch clamp experiment for **3** (1 mM), experiment id 84 at +100 mV. Events were categorised into the following types: 0: -∞ A ≤ Event 0 < 5.00e-11 A, 1: 5.00e-11 A ≤ Event 1 < 5.00e-10 A, 2: 5.00e-10 A ≤ Event 2 < 4.90e-08 A, 3: 4.90e-08 A ≤ Event 3 < ∞ A. Subfigure (d) shows the percentage of points found above the threshold -1.1e-11 (A) in a rolling look ahead window of 20 points. The threshold is the mean + standard deviation of the noise of the first 200 datapoints.

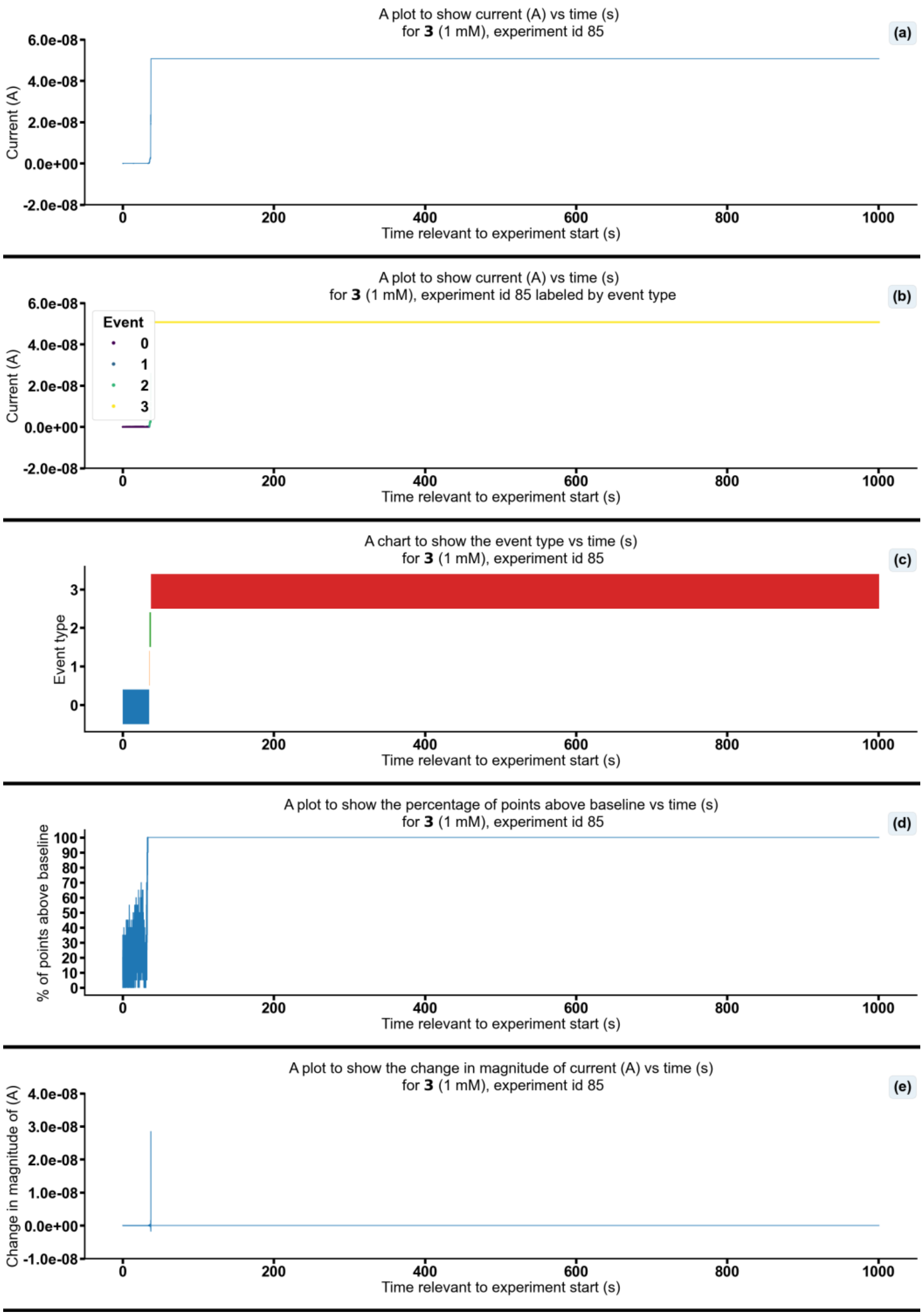

Figure S202 - Summarising the data from the patch clamp experiment for **3** (1 mM), experiment id 85 at +100 mV. Events were categorised into the following types: 0: -∞ A ≤ Event 0 < 5.00e-11 A, 1: 5.00e-11 A ≤ Event 1 < 5.00e-10 A, 2: 5.00e-10 A ≤ Event 2 < 4.90e-08 A, 3: 4.90e-08 A ≤ Event 3 < ∞ A. Subfigure (d) shows the percentage of points found above the threshold -9.1e-12 (A) in a rolling look ahead window of 20 points. The threshold is the mean + standard deviation of the noise of the first 200 datapoints.

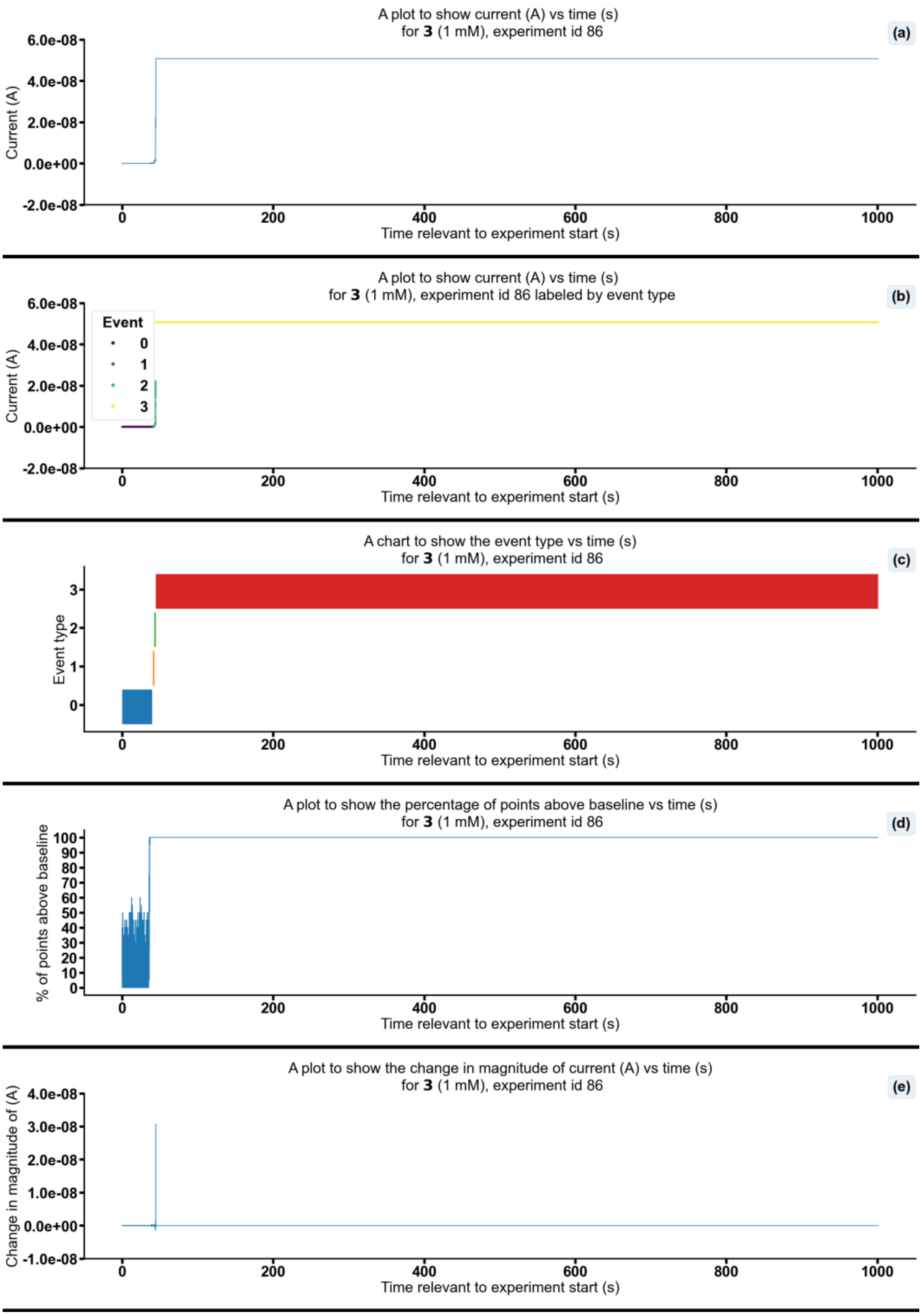

Figure S203 - Summarising the data from the patch clamp experiment for **3** (1 mM), experiment id 86 at +100 mV. Events were categorised into the following types: 0: -∞ A ≤ Event 0 < 5.00e-11 A, 1: 5.00e-11 A ≤ Event 1 < 5.00e-10 A, 2: 5.00e-10 A ≤ Event 2 < 4.90e-08 A, 3: 4.90e-08 A ≤ Event 3 < ∞ A. Subfigure (d) shows the percentage of points found above the threshold -5.4e-12 (A) in a rolling look ahead window of 20 points. The threshold is the mean + standard deviation of the noise of the first 200 datapoints.

Changing the concentration of **5**

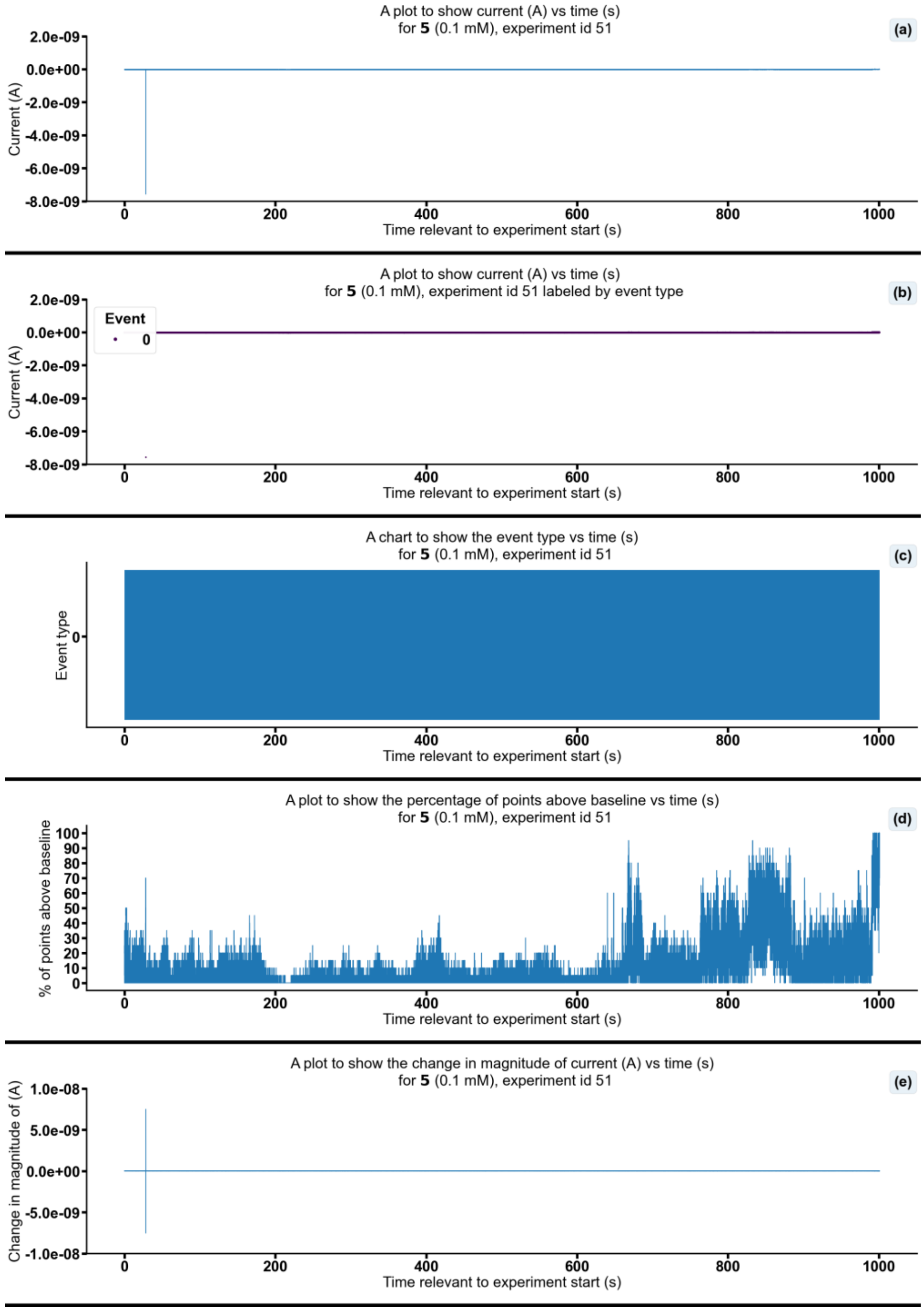

Figure S204 - Summarising the data from the patch clamp experiment for **5** (0.1 mM), experiment id 51 at +100 mV. Events were categorised into the following types: 0: -∞ A ≤ Event 0 < 5.00e-11 A, 1: 5.00e-11 A ≤ Event 1 < 5.00e-10 A, 2: 5.00e-10 A ≤ Event 2 < 4.90e-08 A, 3: 4.90e-08 A ≤ Event 3 < ∞ A. Subfigure (d) shows the percentage of points found above the threshold -1.4e-11 (A) in a rolling look ahead window of 20 points. The threshold is the mean + standard deviation of the noise of the first 200 datapoints.

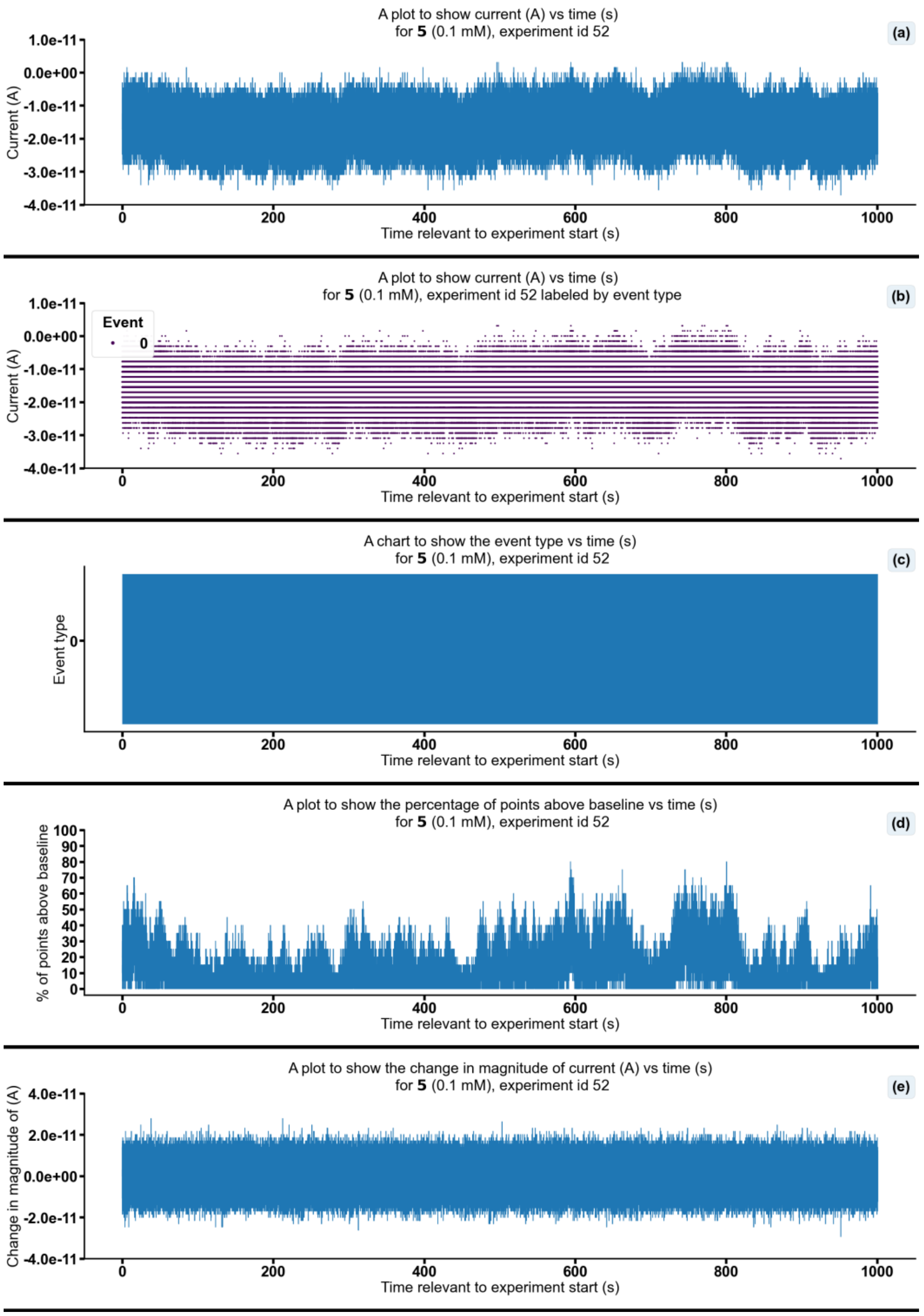

Figure S205 - Summarising the data from the patch clamp experiment for **5** (0.1 mM), experiment id 52 at +100 mV. Events were categorised into the following types: 0: -∞ A ≤ Event 0 < 5.00e-11 A, 1: 5.00e-11 A ≤ Event 1 < 5.00e-10 A, 2: 5.00e-10 A ≤ Event 2 < 4.90e-08 A, 3: 4.90e-08 A ≤ Event 3 < ∞ A. Subfigure (d) shows the percentage of points found above the threshold -1.1e-11 (A) in a rolling look ahead window of 20 points. The threshold is the mean + standard deviation of the noise of the first 200 datapoints.

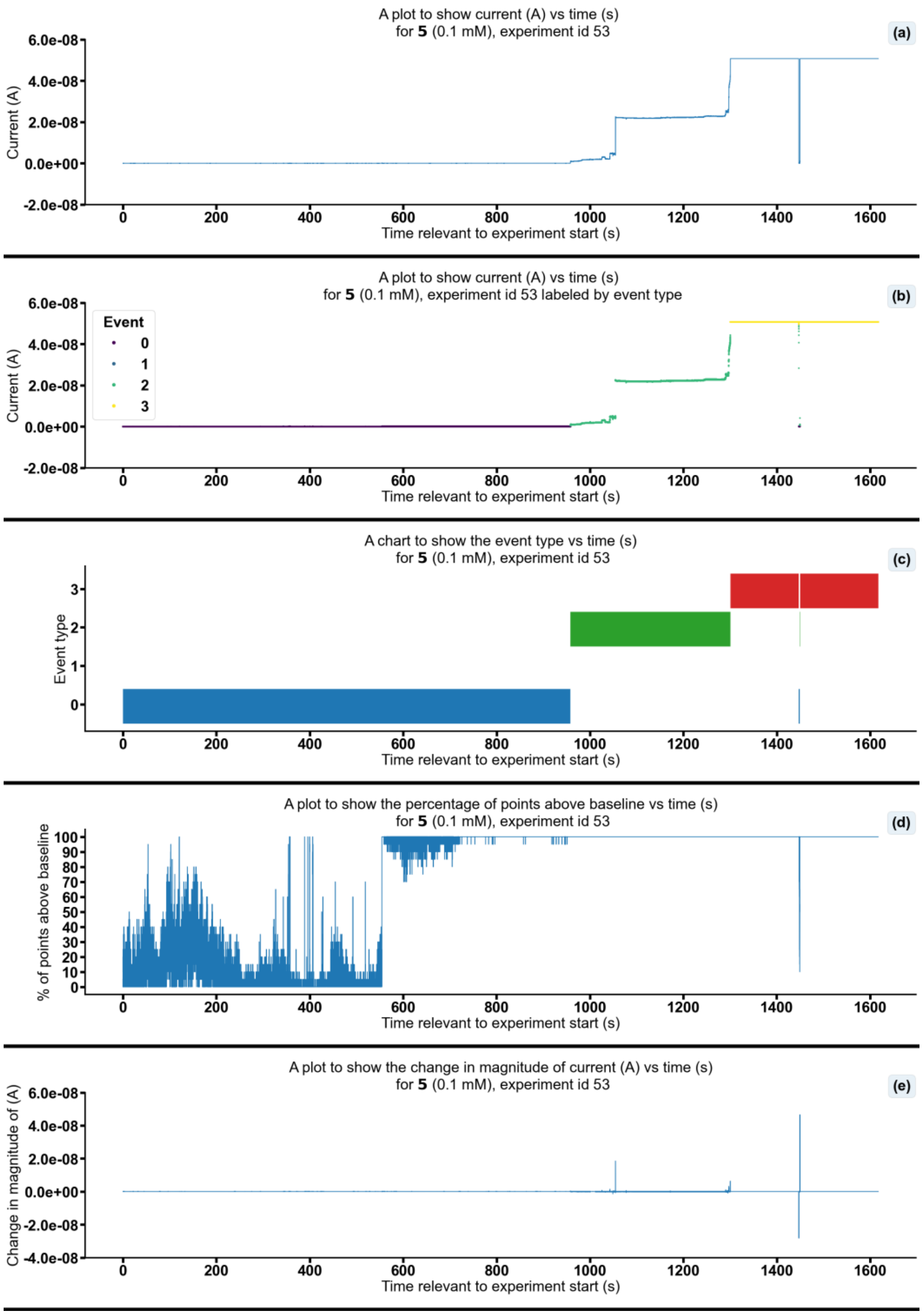

Figure S206 - Summarising the data from the patch clamp experiment for **5** (0.1 mM), experiment id 53 at +100 mV. Events were categorised into the following types: 0: -∞ A ≤ Event 0 < 5.00e-11 A, 1: 5.00e-11 A ≤ Event 1 < 5.00e-10 A, 2: 5.00e-10 A ≤ Event 2 < 4.90e-08 A, 3: 4.90e-08 A ≤ Event 3 < ∞ A. Subfigure (d) shows the percentage of points found above the threshold -1.8e-11 (A) in a rolling look ahead window of 20 points. The threshold is the mean + standard deviation of the noise of the first 200 datapoints.

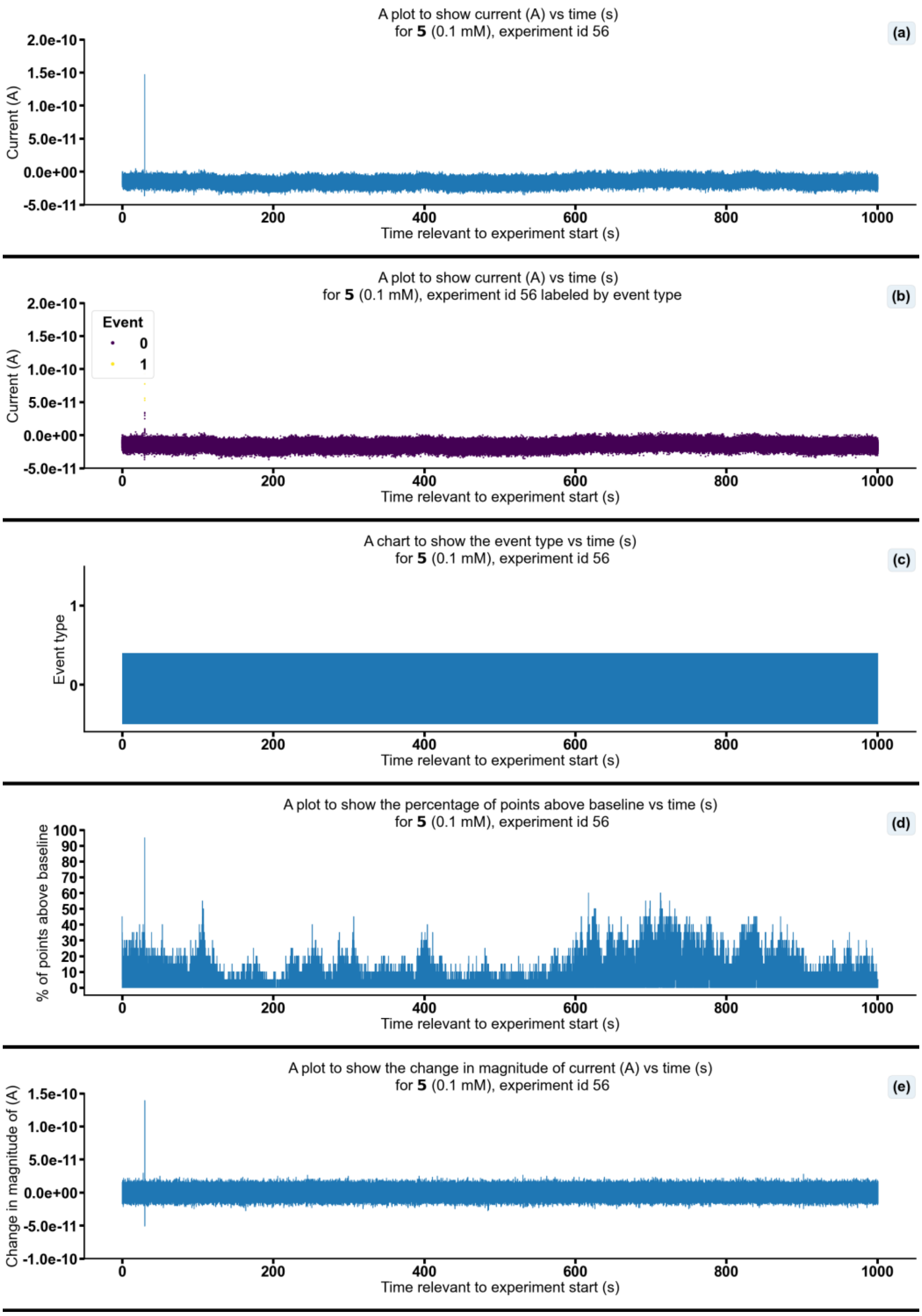

Figure S207 - Summarising the data from the patch clamp experiment for **5** (0.1 mM), experiment id 56 at +100 mV. Events were categorised into the following types: 0: -∞ A ≤ Event 0 < 5.00e-11 A, 1: 5.00e-11 A ≤ Event 1 < 5.00e-10 A, 2: 5.00e-10 A ≤ Event 2 < 4.90e-08 A, 3: 4.90e-08 A ≤ Event 3 < ∞ A. Subfigure (d) shows the percentage of points found above the threshold -8.2e-12 (A) in a rolling look ahead window of 20 points. The threshold is the mean + standard deviation of the noise of the first 200 datapoints.

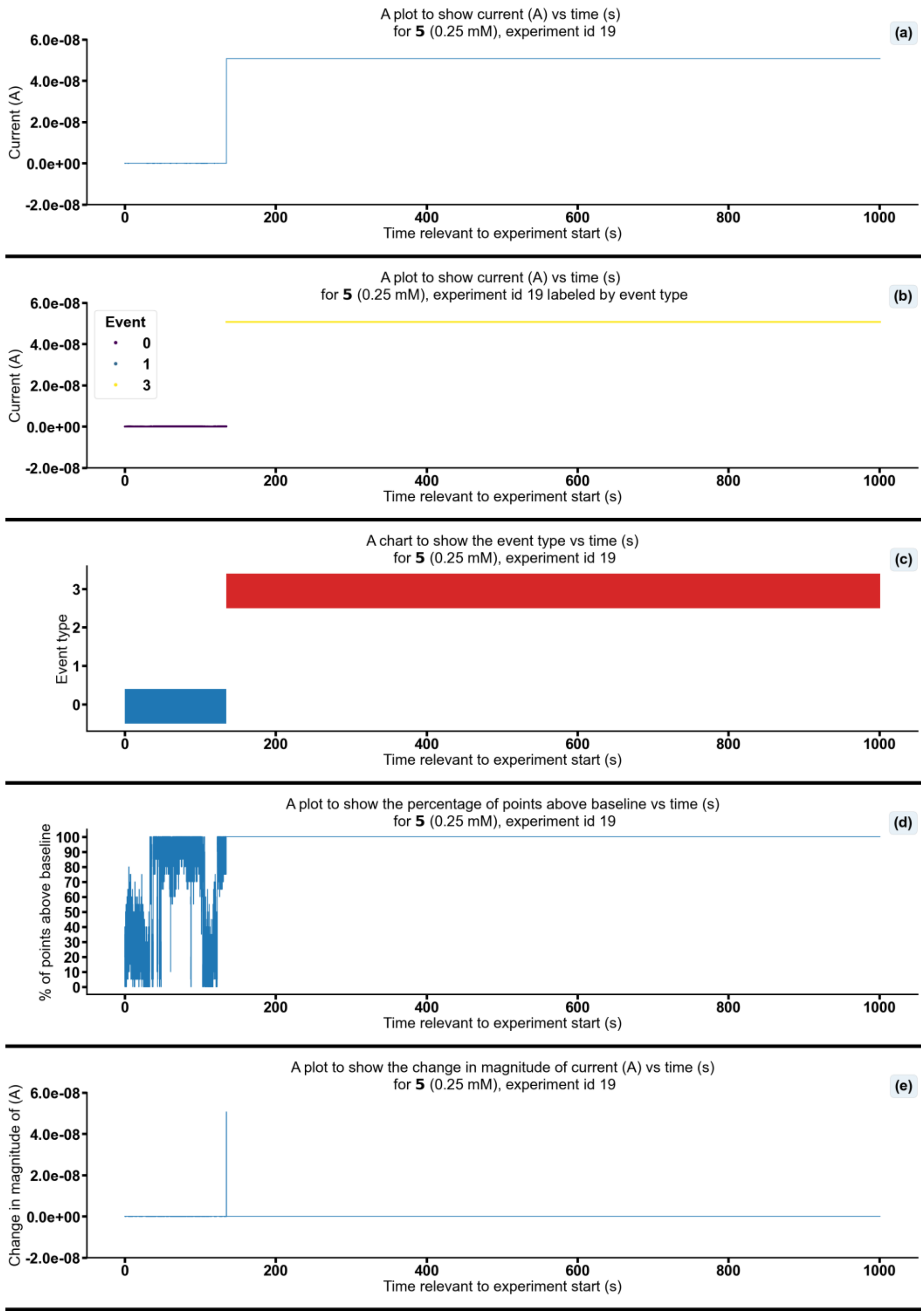

Figure S208 - Summarising the data from the patch clamp experiment for **5** (0.25 mM), experiment id 19 at +100 mV. Events were categorised into the following types: 0: -∞ A ≤ Event 0 < 5.00e-11 A, 1: 5.00e-11 A ≤ Event 1 < 5.00e-10 A, 2: 5.00e-10 A ≤ Event 2 < 4.90e-08 A, 3: 4.90e-08 A ≤ Event 3 < ∞ A. Subfigure (d) shows the percentage of points found above the threshold -9.4e-12 (A) in a rolling look ahead window of 20 points. The threshold is the mean + standard deviation of the noise of the first 200 datapoints.

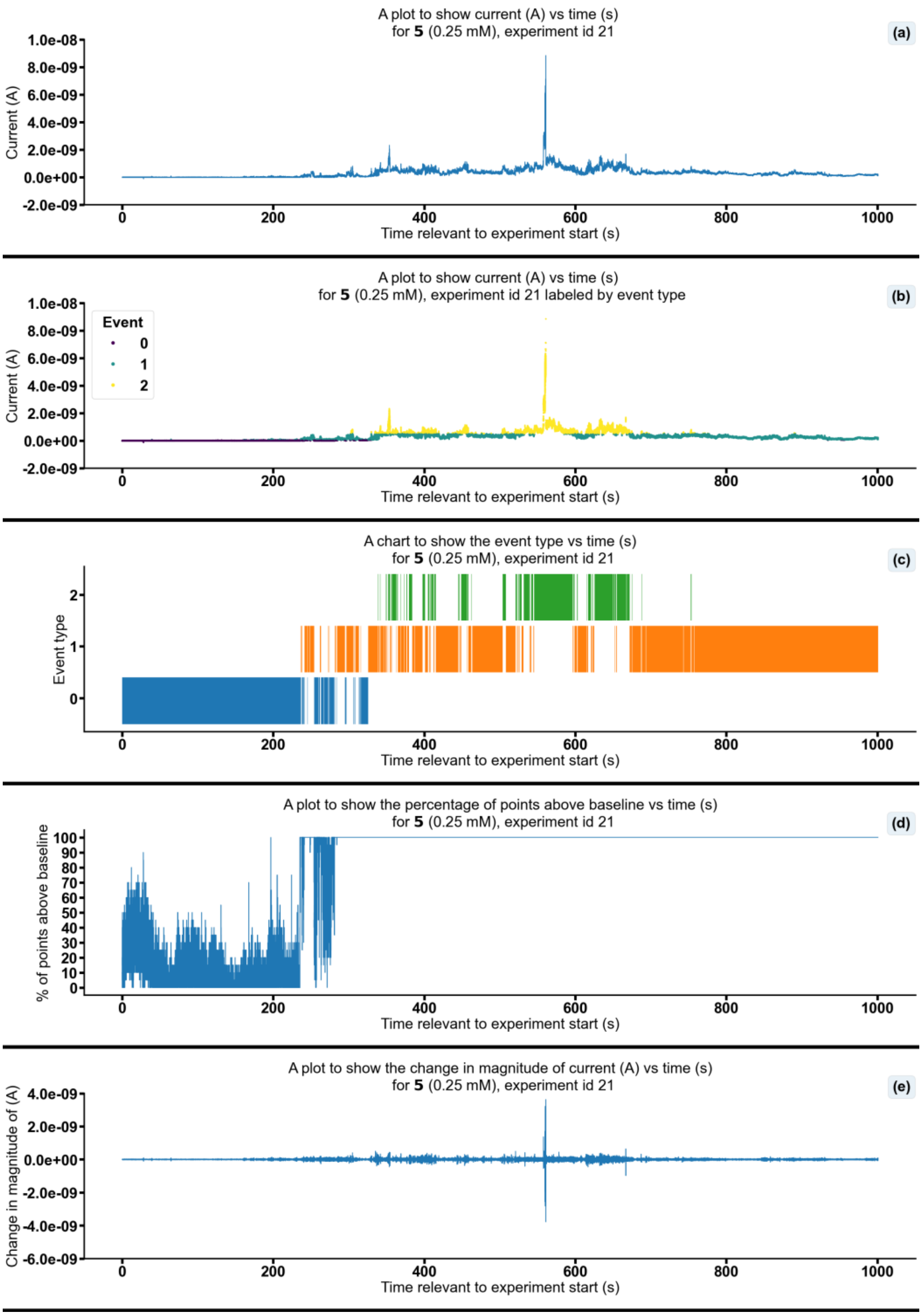

Figure S209- Summarising the data from the patch clamp experiment for **5** (0.25 mM), experiment id 21 at +100 mV. Events were categorised into the following types: 0: -∞ A ≤ Event 0 < 5.00e-11 A, 1: 5.00e-11 A ≤ Event 1 < 5.00e-10 A, 2: 5.00e-10 A ≤ Event 2 < 4.90e-08 A, 3: 4.90e-08 A ≤ Event 3 < ∞ A. Subfigure (d) shows the percentage of points found above the threshold -8.6e-12 (A) in a rolling look ahead window of 20 points. The threshold is the mean + standard deviation of the noise of the first 200 datapoints.

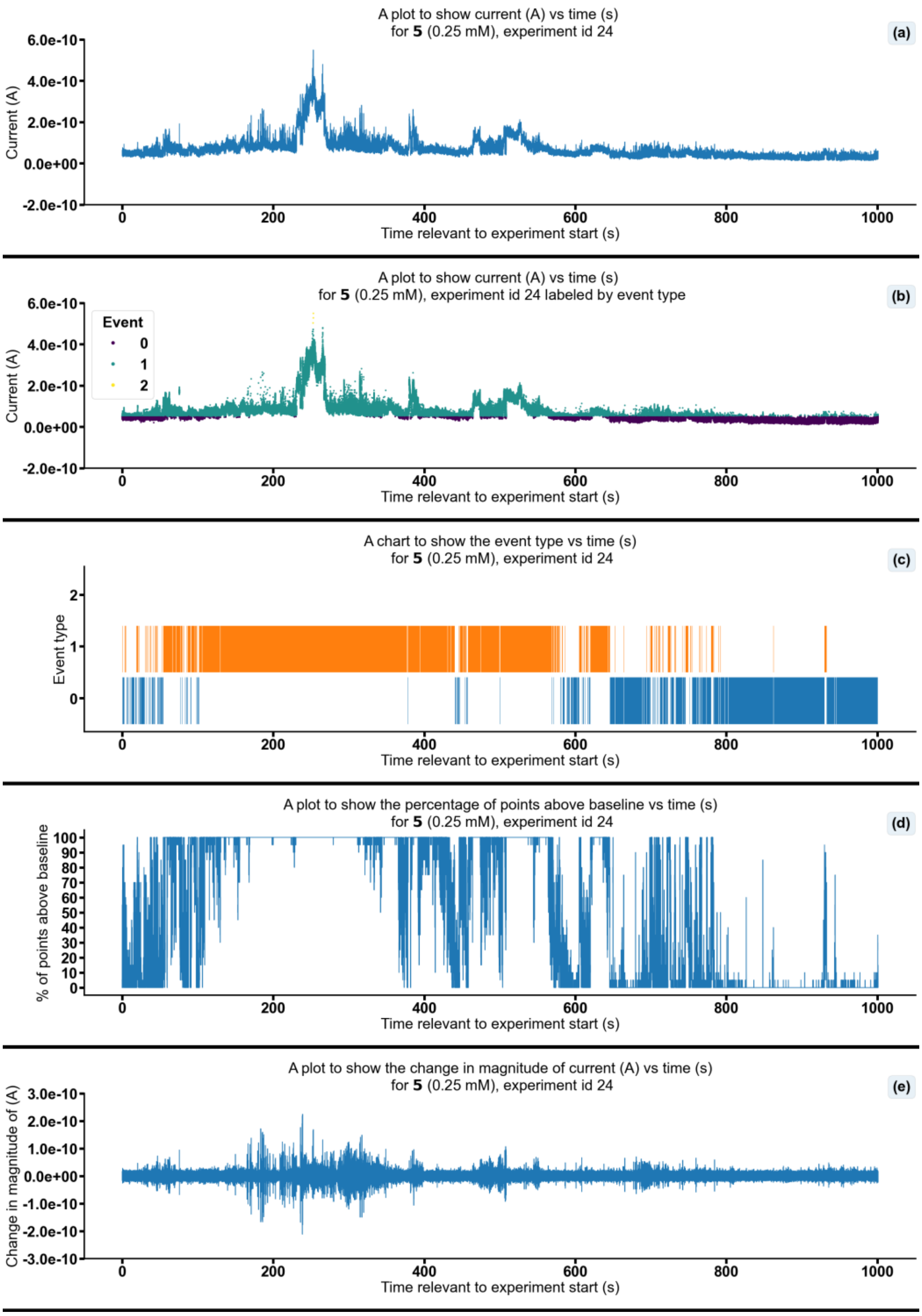

Figure S210 - Summarising the data from the patch clamp experiment for **5** (0.25 mM), experiment id 24 at +100 mV. Events were categorised into the following types: 0: -∞ A ≤ Event 0 < 5.00e-11 A, 1: 5.00e-11 A ≤ Event 1 < 5.00e-10 A, 2: 5.00e-10 A ≤ Event 2 < 4.90e-08 A, 3: 4.90e-08 A ≤ Event 3 < ∞ A. Subfigure (d) shows the percentage of points found above the threshold 5.5e-11 (A) in a rolling look ahead window of 20 points. The threshold is the mean + standard deviation of the noise of the first 200 datapoints.

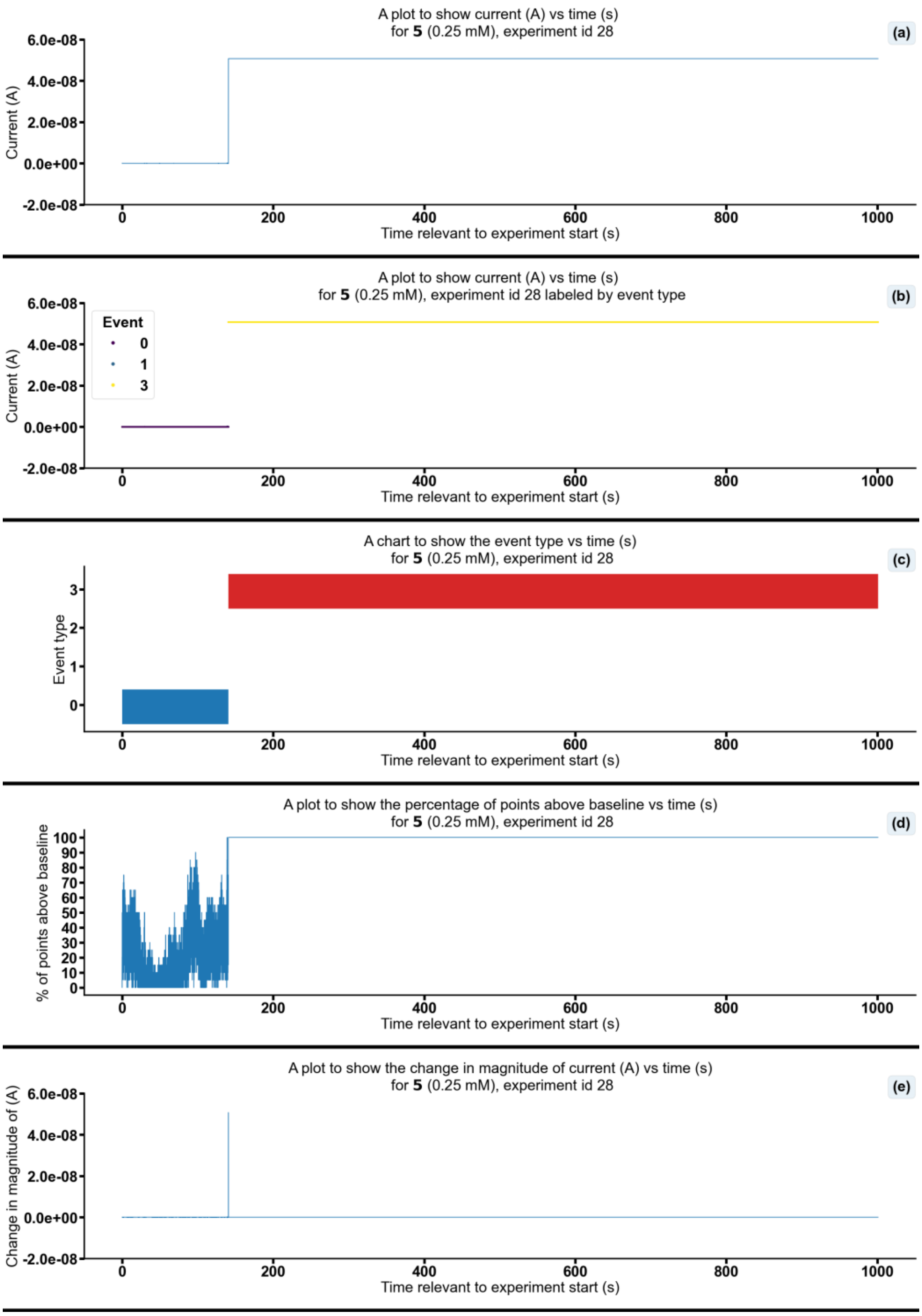

Figure S211 - Summarising the data from the patch clamp experiment for **5** (0.25 mM), experiment id 28 at +100 mV. Events were categorised into the following types: 0: -∞ A ≤ Event 0 < 5.00e-11 A, 1: 5.00e-11 A ≤ Event 1 < 5.00e-10 A, 2: 5.00e-10 A ≤ Event 2 < 4.90e-08 A, 3: 4.90e-08 A ≤ Event 3 < ∞ A. Subfigure (d) shows the percentage of points found above the threshold -1.9e-11 (A) in a rolling look ahead window of 20 points. The threshold is the mean + standard deviation of the noise of the first 200 datapoints.

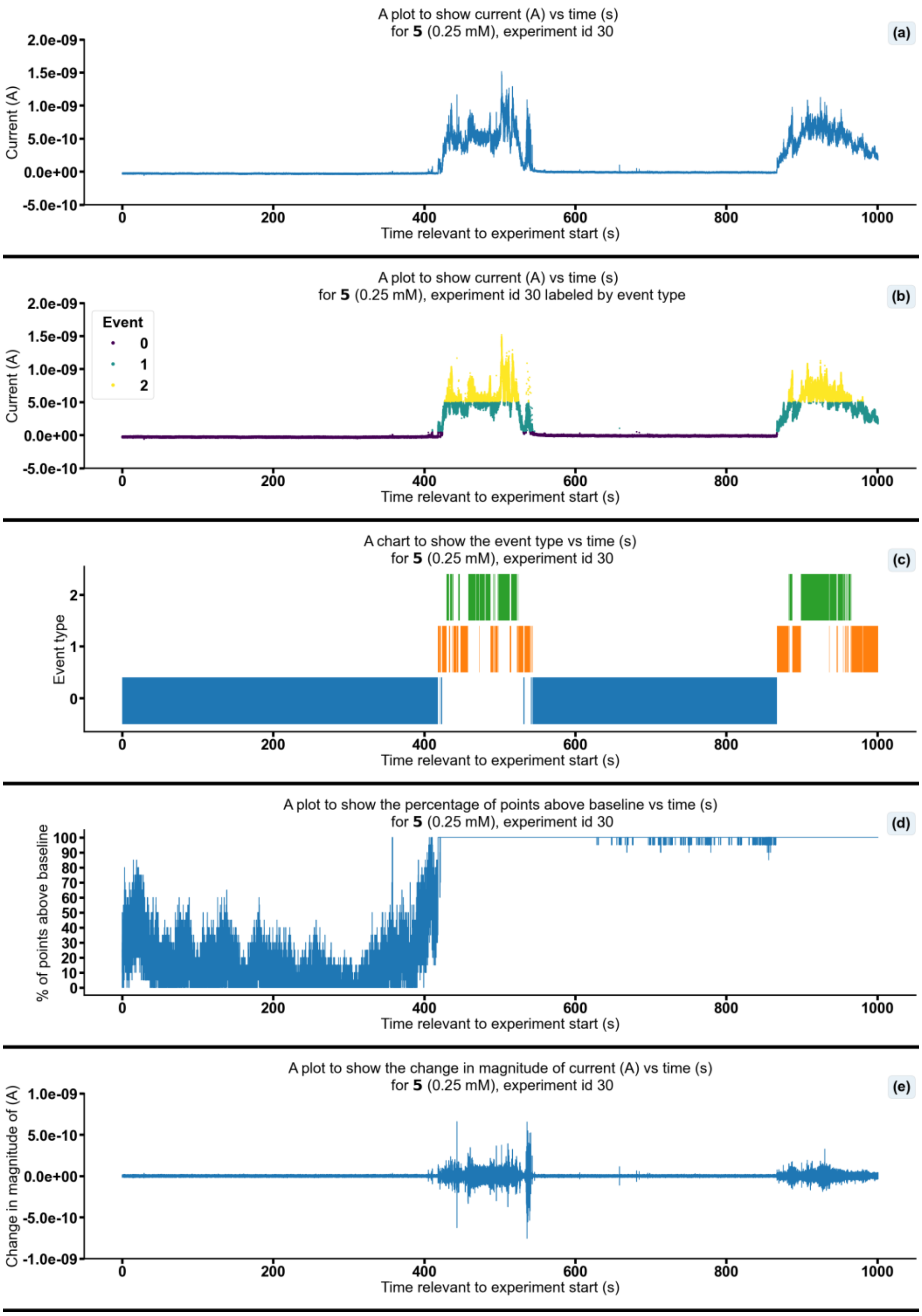

Figure S212 - Summarising the data from the patch clamp experiment for **5** (0.25 mM), experiment id 30 at +100 mV. Events were categorised into the following types: 0: -∞ A ≤ Event 0 < 5.00e-11 A, 1: 5.00e-11 A ≤ Event 1 < 5.00e-10 A, 2: 5.00e-10 A ≤ Event 2 < 4.90e-08 A, 3: 4.90e-08 A ≤ Event 3 < ∞ A. Subfigure (d) shows the percentage of points found above the threshold -2.5e-11 (A) in a rolling look ahead window of 20 points. The threshold is the mean + standard deviation of the noise of the first 200 datapoints.

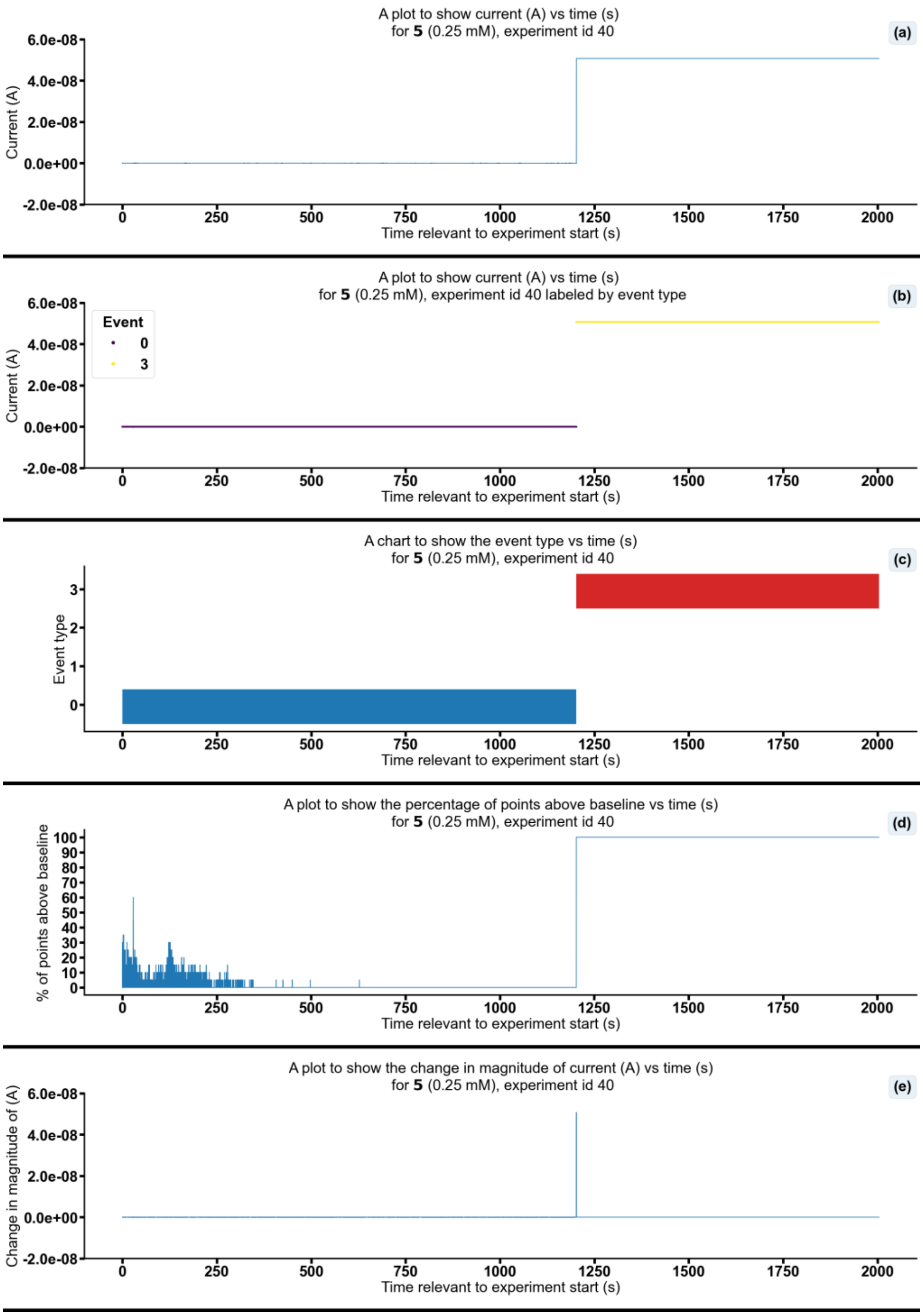

Figure S213 - Summarising the data from the patch clamp experiment for **5** (0.25 mM), experiment id 40 at +100 mV. Events were categorised into the following types: 0: -∞ A ≤ Event 0 < 5.00e-11 A, 1: 5.00e-11 A ≤ Event 1 < 5.00e-10 A, 2: 5.00e-10 A ≤ Event 2 < 4.90e-08 A, 3: 4.90e-08 A ≤ Event 3 < ∞ A. Subfigure (d) shows the percentage of points found above the threshold -3.8e-11 (A) in a rolling look ahead window of 20 points. The threshold is the mean + standard deviation of the noise of the first 200 datapoints.

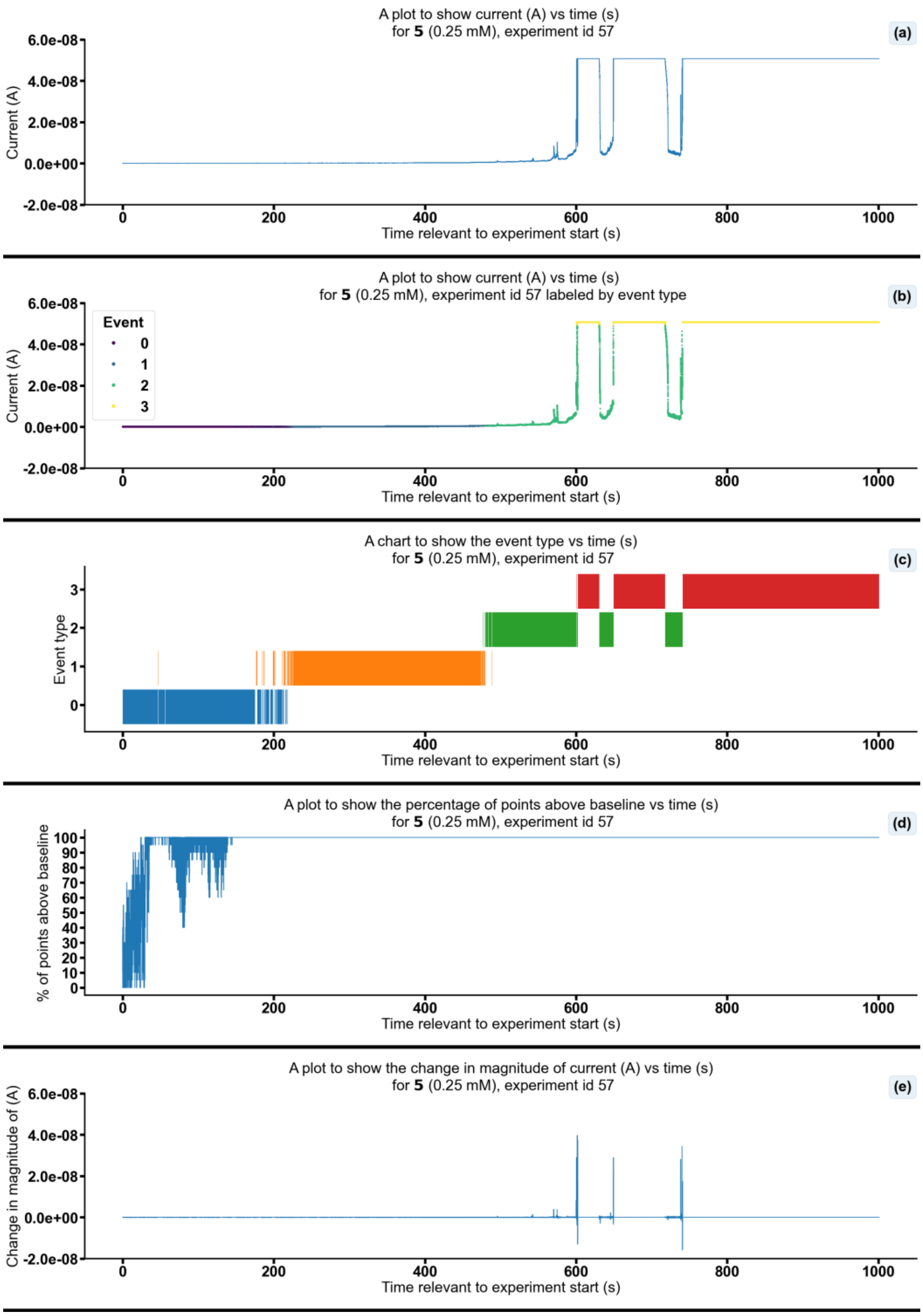

Figure S214 - Summarising the data from the patch clamp experiment for **5** (0.25 mM), experiment id 57 at +100 mV. Events were categorised into the following types: 0: -∞ A ≤ Event 0 < 5.00e-11 A, 1: 5.00e-11 A ≤ Event 1 < 5.00e-10 A, 2: 5.00e-10 A ≤ Event 2 < 4.90e-08 A, 3: 4.90e-08 A ≤ Event 3 < ∞ A. Subfigure (d) shows the percentage of points found above the threshold 2.3e-11 (A) in a rolling look ahead window of 20 points. The threshold is the mean + standard deviation of the noise of the first 200 datapoints.

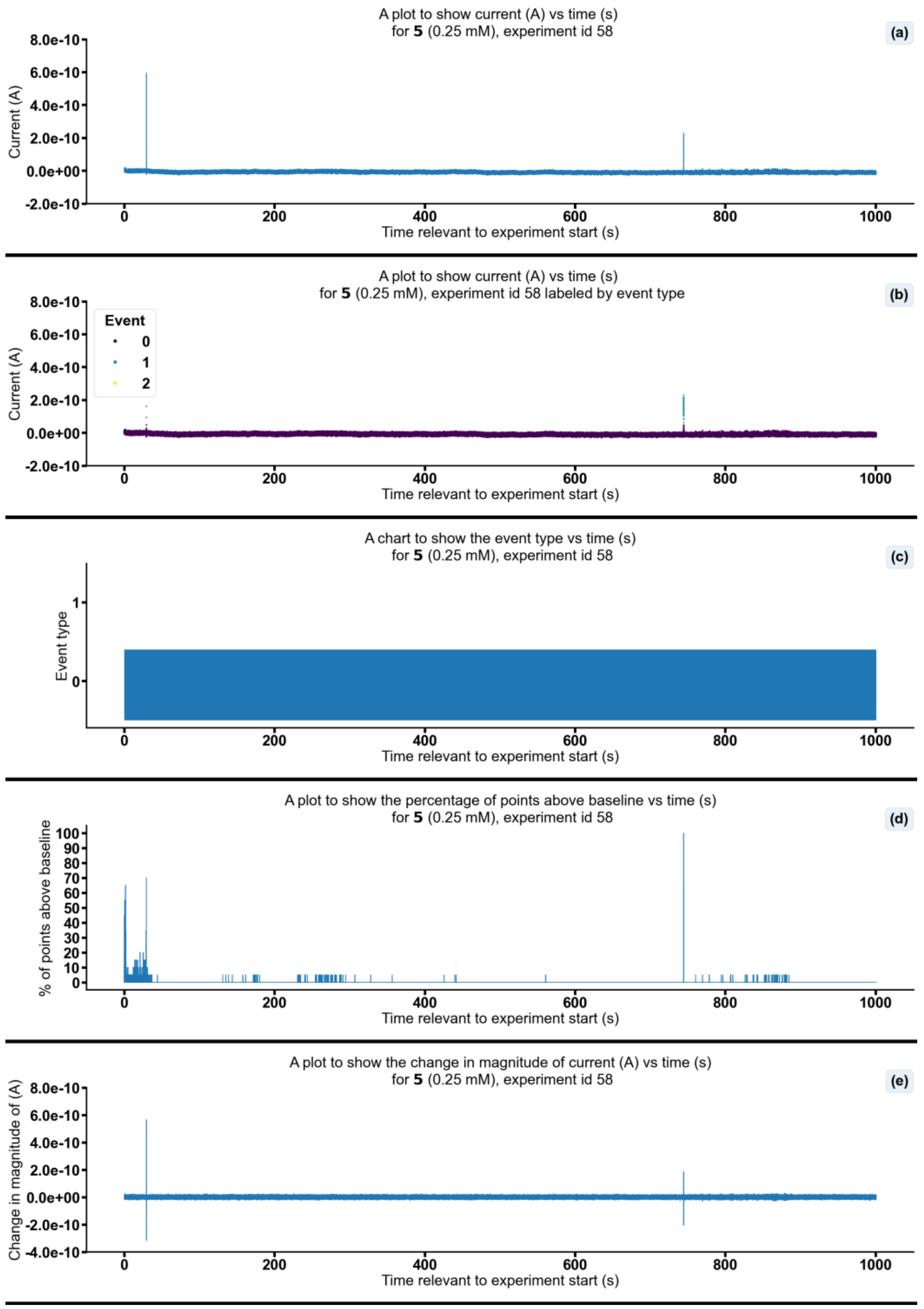

Figure S215 - Summarising the data from the patch clamp experiment for **5** (0.25 mM), experiment id 58 at +100 mV. Events were categorised into the following types: 0: -∞ A ≤ Event 0 < 5.00e-11 A, 1: 5.00e-11 A ≤ Event 1 < 5.00e-10 A, 2: 5.00e-10 A ≤ Event 2 < 4.90e-08 A, 3: 4.90e-08 A ≤ Event 3 < ∞ A. Subfigure (d) shows the percentage of points found above the threshold 8.3e-12 (A) in a rolling look ahead window of 20 points. The threshold is the mean + standard deviation of the noise of the first 200 datapoints.

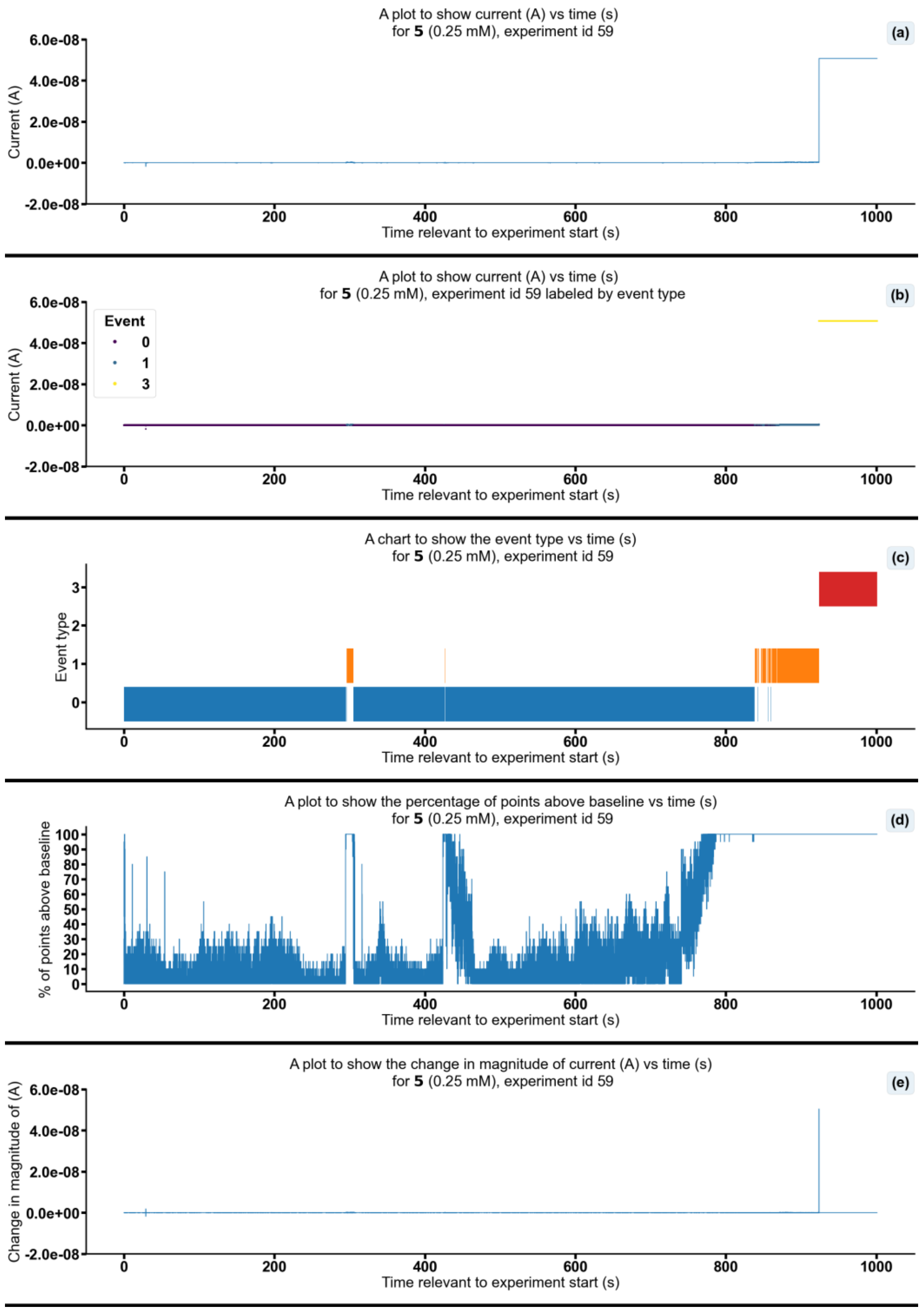

Figure S216 - Summarising the data from the patch clamp experiment for **5** (0.25 mM), experiment id 59 at +100 mV. Events were categorised into the following types: 0: -∞ A ≤ Event 0 < 5.00e-11 A, 1: 5.00e-11 A ≤ Event 1 < 5.00e-10 A, 2: 5.00e-10 A ≤ Event 2 < 4.90e-08 A, 3: 4.90e-08 A ≤ Event 3 < ∞ A. Subfigure (d) shows the percentage of points found above the threshold 1.6e-12 (A) in a rolling look ahead window of 20 points. The threshold is the mean + standard deviation of the noise of the first 200 datapoints.

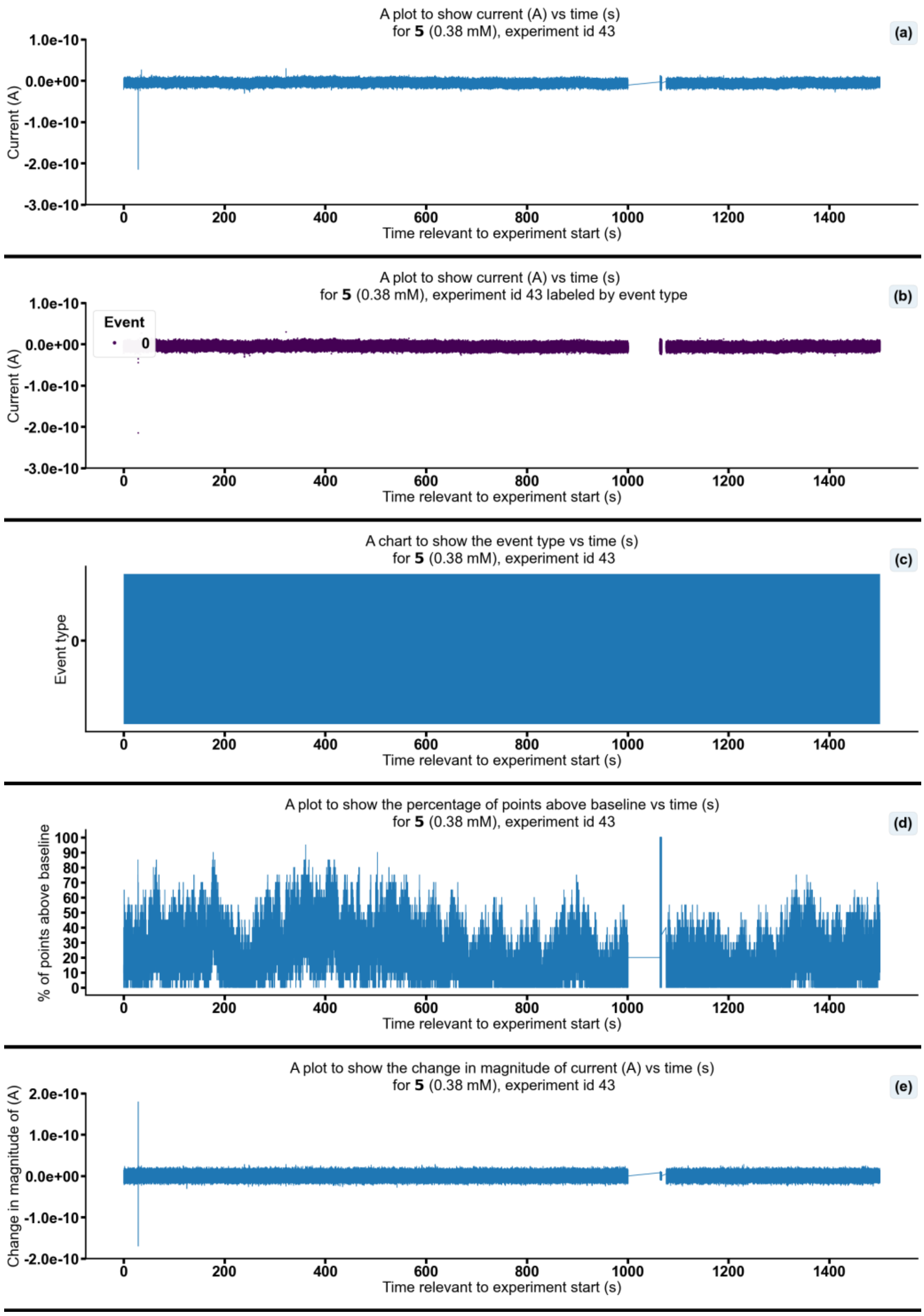

Figure S217 - Summarising the data from the patch clamp experiment for **5** (0.38 mM), experiment id 43 at +100 mV. Events were categorised into the following types: 0: -∞ A ≤ Event 0 < 5.00e-11 A, 1: 5.00e-11 A ≤ Event 1 < 5.00e-10 A, 2: 5.00e-10 A ≤ Event 2 < 4.90e-08 A, 3: 4.90e-08 A ≤ Event 3 < ∞ A. Subfigure (d) shows the percentage of points found above the threshold -2.6e-12 (A) in a rolling look ahead window of 20 points. The threshold is the mean + standard deviation of the noise of the first 200 datapoints.

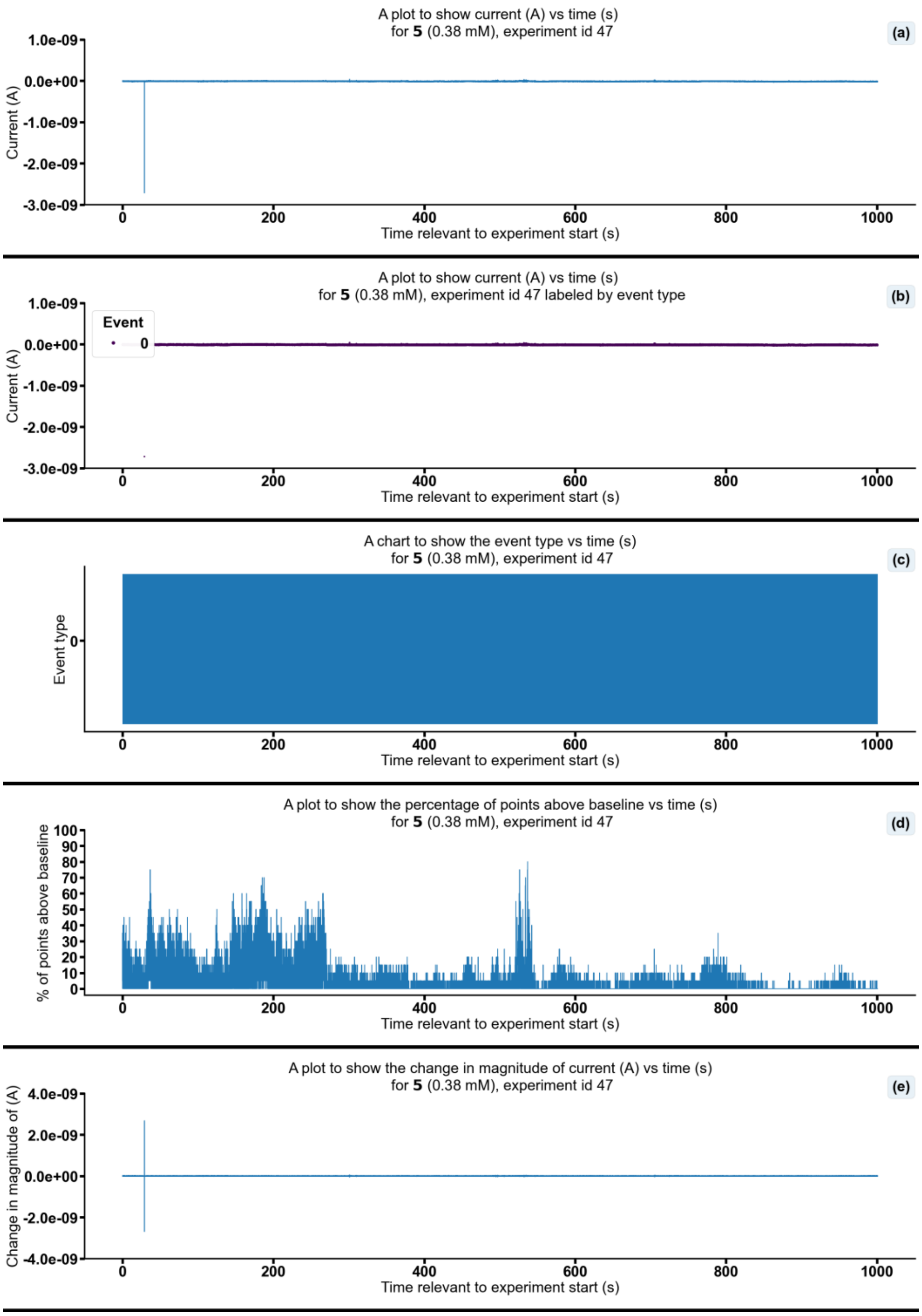

Figure S218 - Summarising the data from the patch clamp experiment for **5** (0.38 mM), experiment id 47 at +100 mV. Events were categorised into the following types: 0: -∞ A ≤ Event 0 < 5.00e-11 A, 1: 5.00e-11 A ≤ Event 1 < 5.00e-10 A, 2: 5.00e-10 A ≤ Event 2 < 4.90e-08 A, 3: 4.90e-08 A ≤ Event 3 < ∞ A. Subfigure (d) shows the percentage of points found above the threshold -5.0e-12 (A) in a rolling look ahead window of 20 points. The threshold is the mean + standard deviation of the noise of the first 200 datapoints.

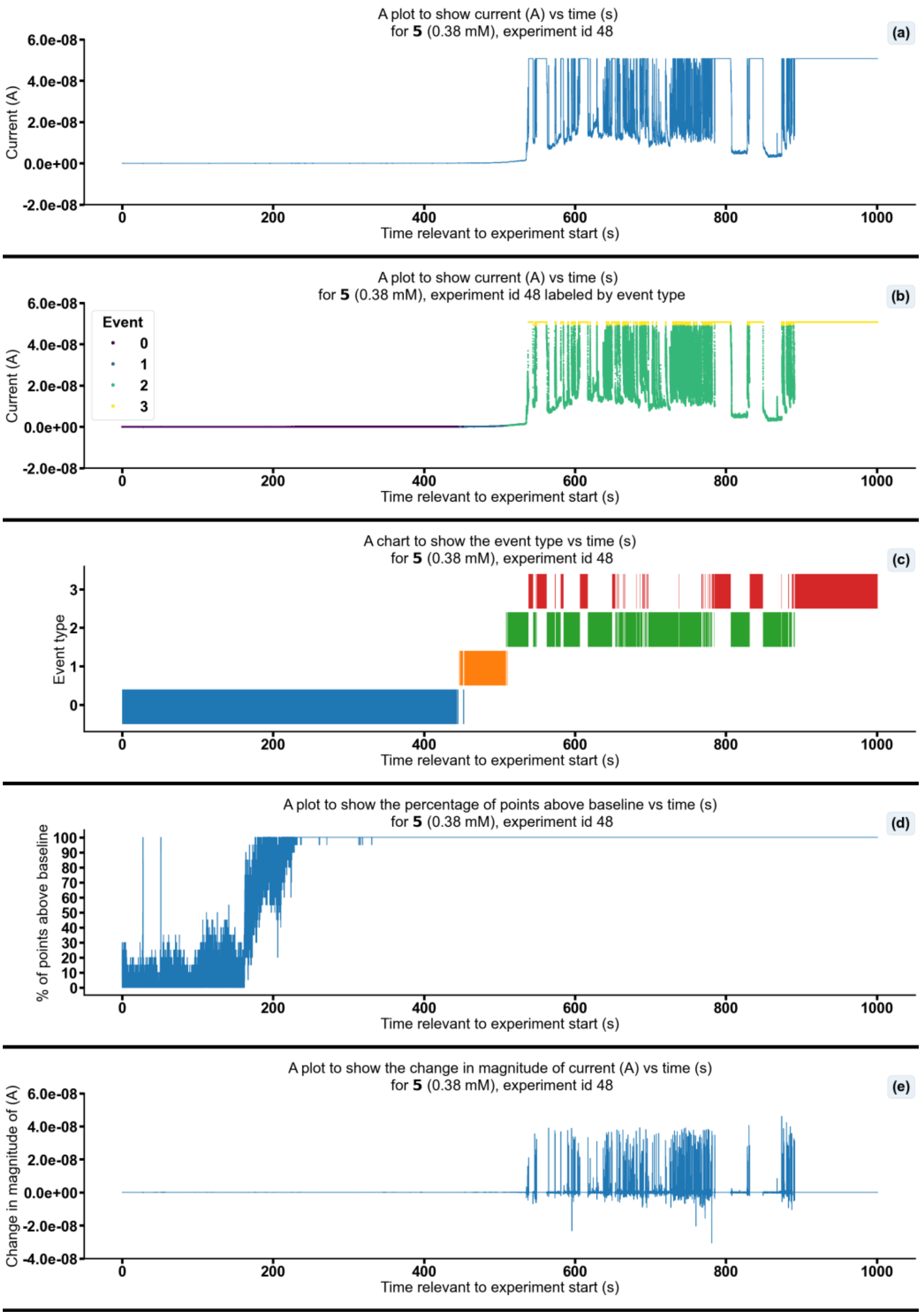

Figure S219 - Summarising the data from the patch clamp experiment for **5** (0.38 mM), experiment id 48 at +100 mV. Events were categorised into the following types: 0: -∞ A ≤ Event 0 < 5.00e-11 A, 1: 5.00e-11 A ≤ Event 1 < 5.00e-10 A, 2: 5.00e-10 A ≤ Event 2 < 4.90e-08 A, 3: 4.90e-08 A ≤ Event 3 < ∞ A. Subfigure (d) shows the percentage of points found above the threshold -2.3e-11 (A) in a rolling look ahead window of 20 points. The threshold is the mean + standard deviation of the noise of the first 200 datapoints.

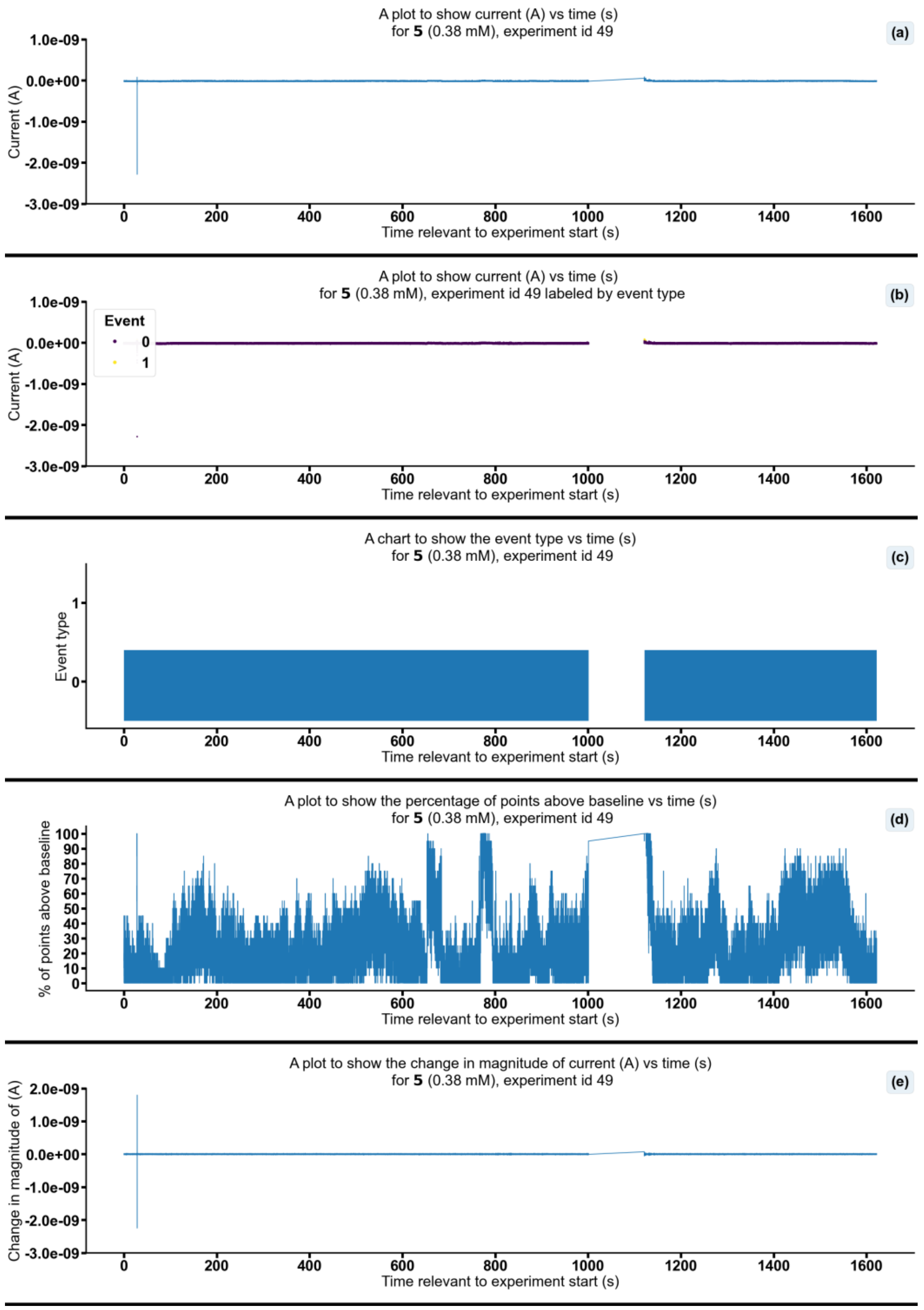

Figure S220 - Summarising the data from the patch clamp experiment for **5** (0.38 mM), experiment id 49 at +100 mV. Events were categorised into the following types: 0: -∞ A ≤ Event 0 < 5.00e-11 A, 1: 5.00e-11 A ≤ Event 1 < 5.00e-10 A, 2: 5.00e-10 A ≤ Event 2 < 4.90e-08 A, 3: 4.90e-08 A ≤ Event 3 < ∞ A. Subfigure (d) shows the percentage of points found above the threshold -1.2e-11 (A) in a rolling look ahead window of 20 points. The threshold is the mean + standard deviation of the noise of the first 200 datapoints.

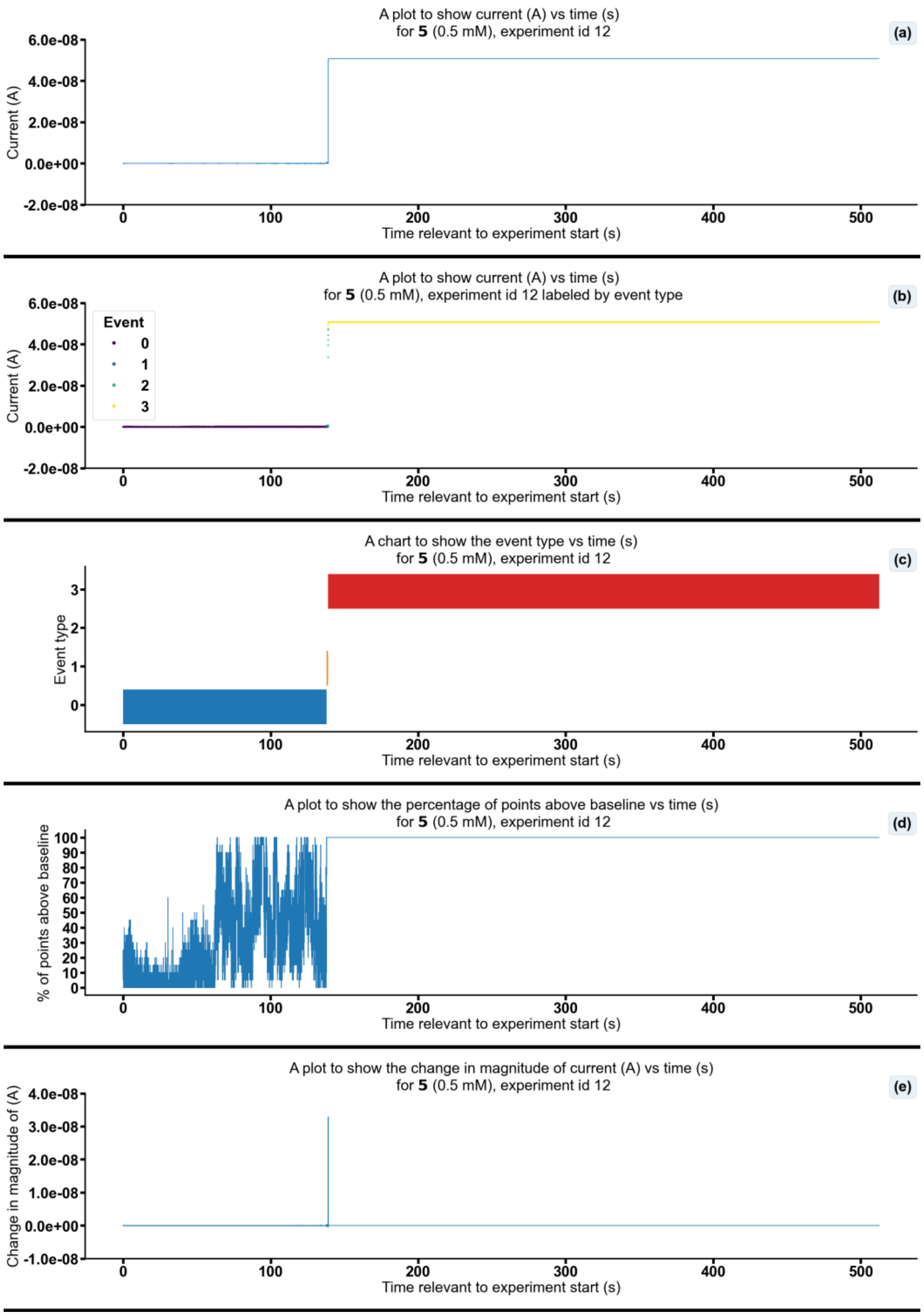

Figure S221 - Summarising the data from the patch clamp experiment for **5** (0.5 mM), experiment id 12 at +100 mV. Events were categorised into the following types: 0: -∞ A ≤ Event 0 < 5.00e-11 A, 1: 5.00e-11 A ≤ Event 1 < 5.00e-10 A, 2: 5.00e-10 A ≤ Event 2 < 4.90e-08 A, 3: 4.90e-08 A ≤ Event 3 < ∞ A. Subfigure (d) shows the percentage of points found above the threshold -5.9e-12 (A) in a rolling look ahead window of 20 points. The threshold is the mean + standard deviation of the noise of the first 200 datapoints.

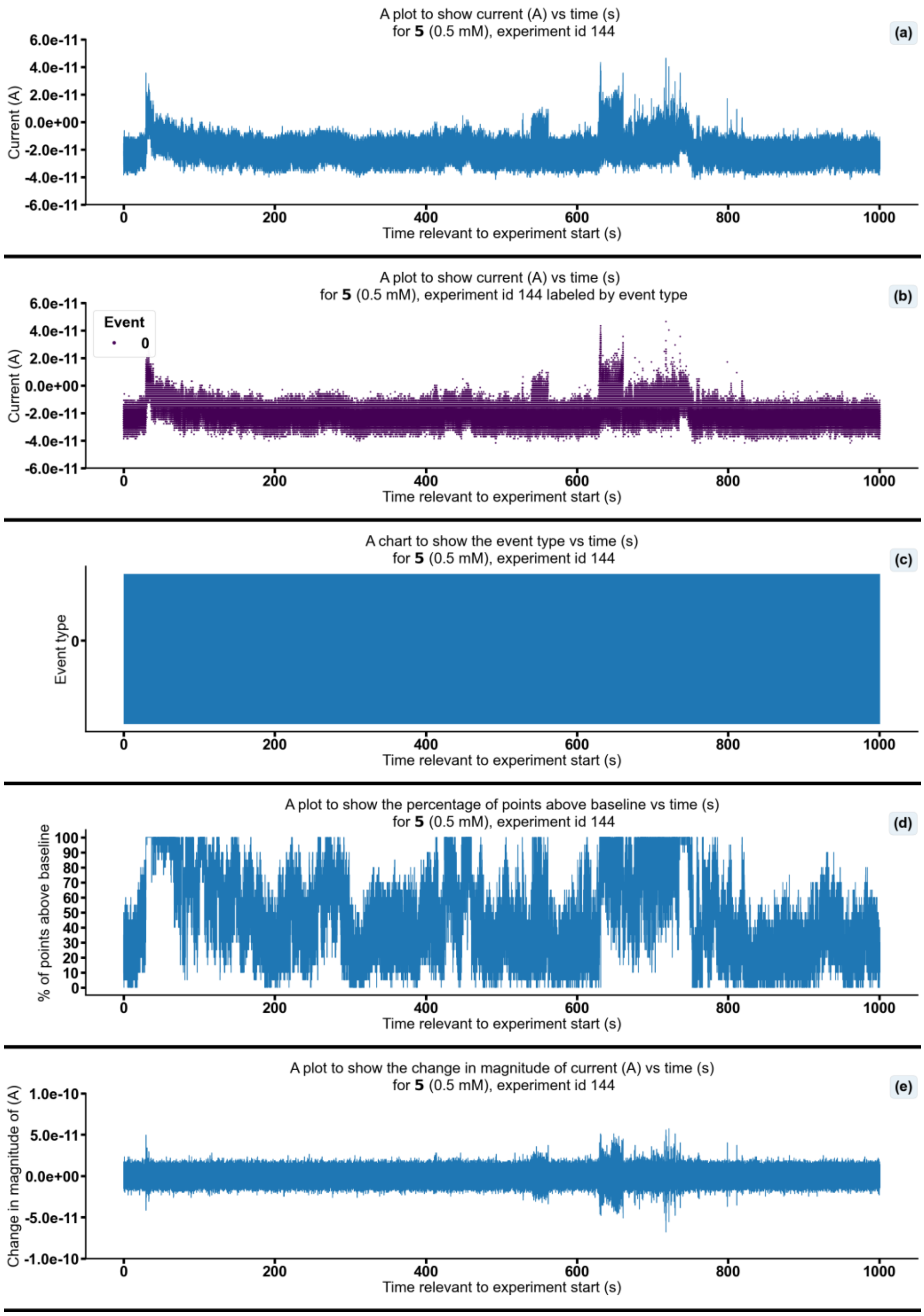

Figure S222 - Summarising the data from the patch clamp experiment for **5** (0.5 mM), experiment id 144 at +100 mV. Events were categorised into the following types: 0: -∞ A ≤ Event 0 < 5.00e-11 A, 1: 5.00e-11 A ≤ Event 1 < 5.00e-10 A, 2: 5.00e-10 A ≤ Event 2 < 4.90e-08 A, 3: 4.90e-08 A ≤ Event 3 < ∞ A. Subfigure (d) shows the percentage of points found above the threshold -2.0e-11 (A) in a rolling look ahead window of 20 points. The threshold is the mean + standard deviation of the noise of the first 200 datapoints.

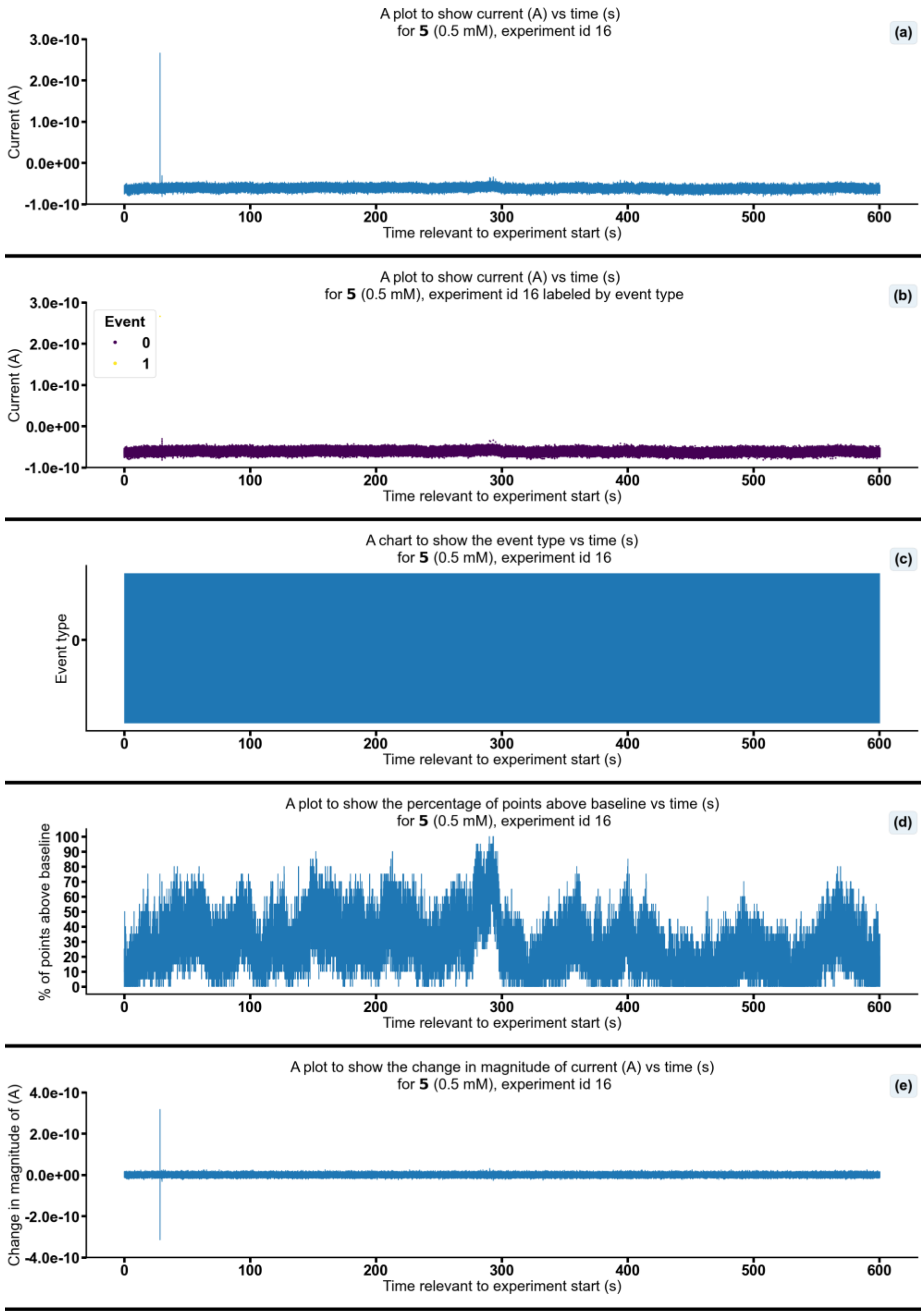

Figure S223 - Summarising the data from the patch clamp experiment for **5** (0.5 mM), experiment id 16 at +100 mV. Events were categorised into the following types: 0: -∞ A ≤ Event 0 < 5.00e-11 A, 1: 5.00e-11 A ≤ Event 1 < 5.00e-10 A, 2: 5.00e-10 A ≤ Event 2 < 4.90e-08 A, 3: 4.90e-08 A ≤ Event 3 < ∞ A. Subfigure (d) shows the percentage of points found above the threshold -6.0e-11 (A) in a rolling look ahead window of 20 points. The threshold is the mean + standard deviation of the noise of the first 200 datapoints.

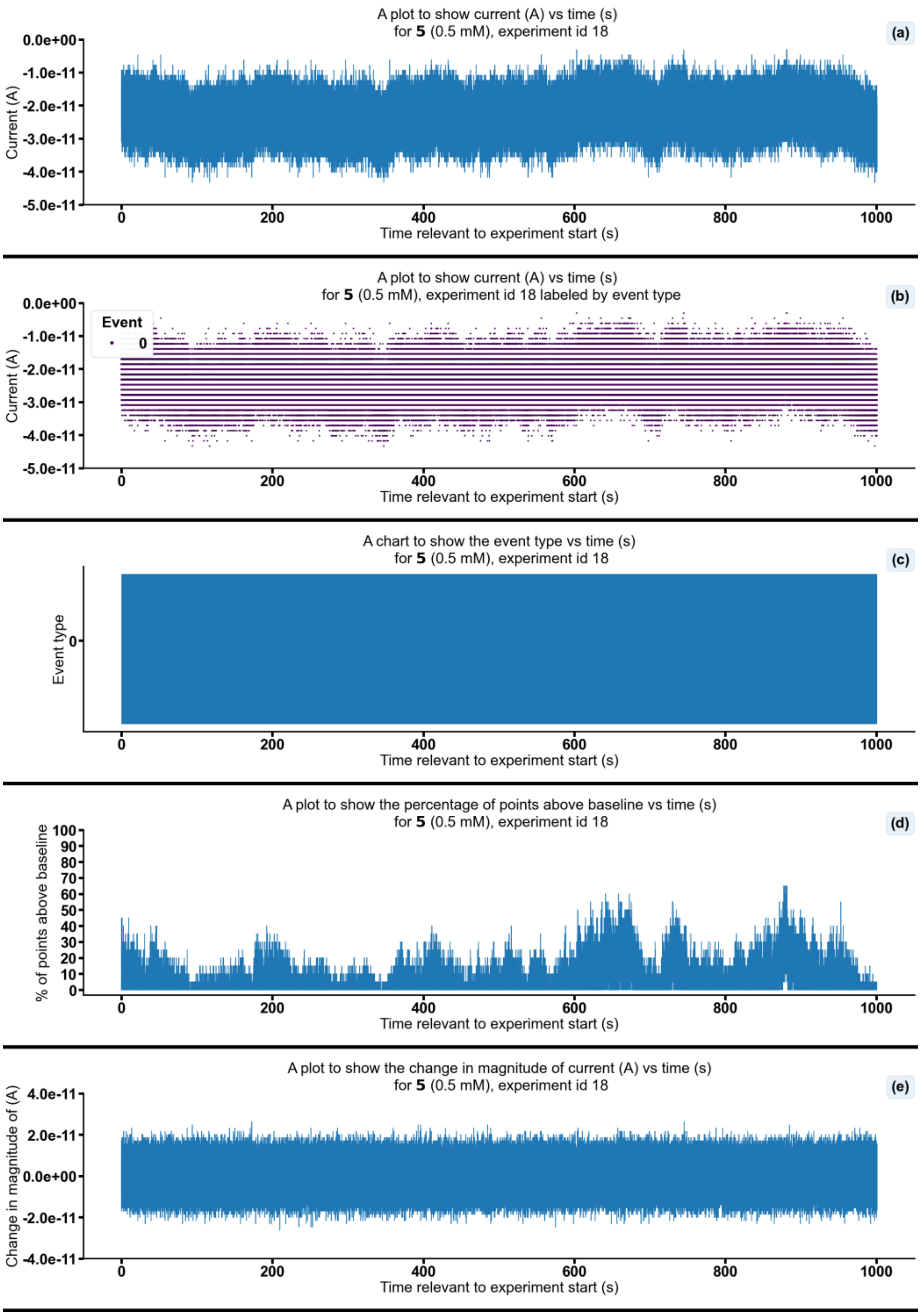

Figure S224 - Summarising the data from the patch clamp experiment for **5** (0.5 mM), experiment id 18 at +100 mV. Events were categorised into the following types: 0: -∞ A ≤ Event 0 < 5.00e-11 A, 1: 5.00e-11 A ≤ Event 1 < 5.00e-10 A, 2: 5.00e-10 A ≤ Event 2 < 4.90e-08 A, 3: 4.90e-08 A ≤ Event 3 < ∞ A. Subfigure (d) shows the percentage of points found above the threshold -1.6e-11 (A) in a rolling look ahead window of 20 points. The threshold is the mean + standard deviation of the noise of the first 200 datapoints.

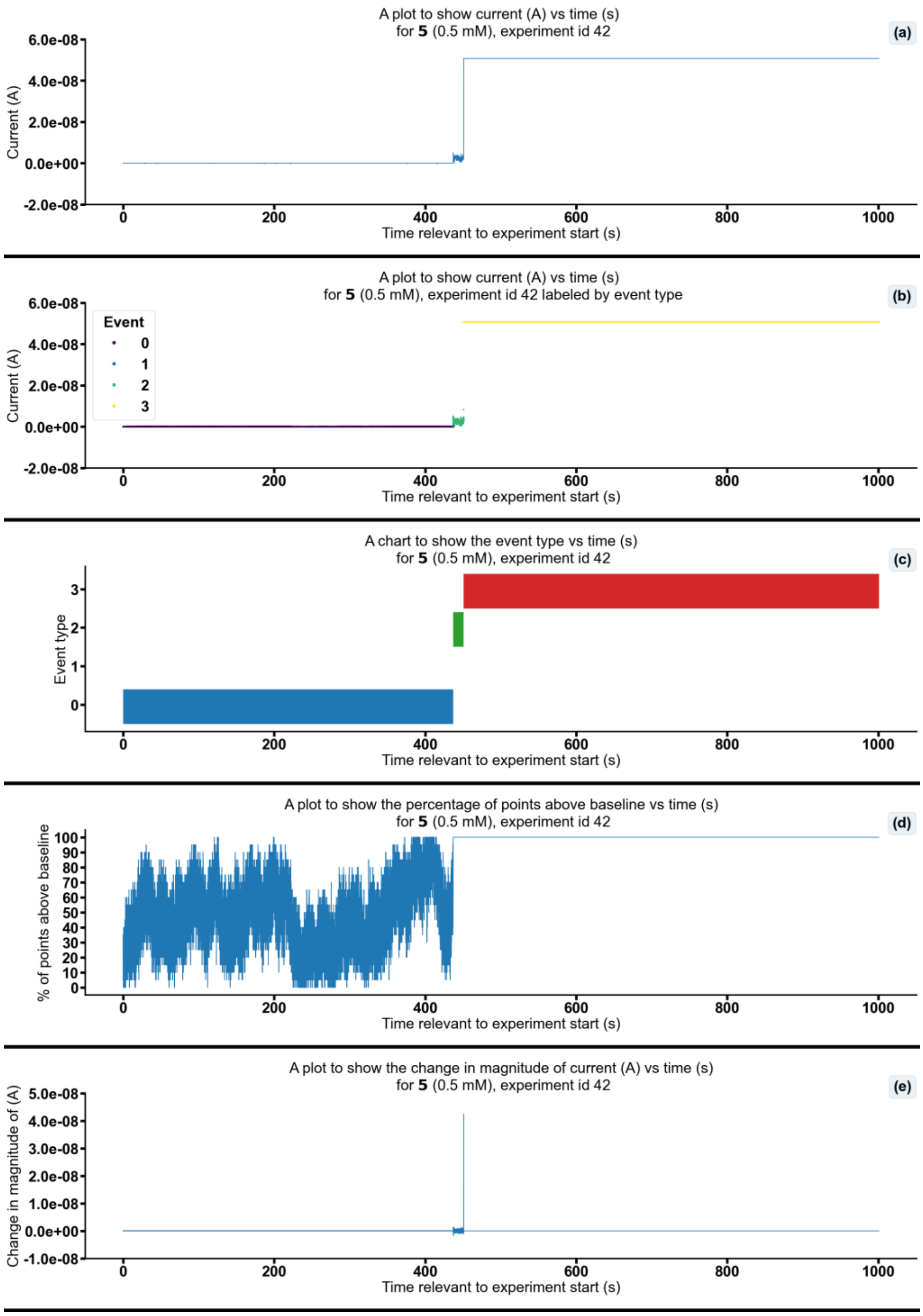

Figure S225 - Summarising the data from the patch clamp experiment for **5** (0.5 mM), experiment id 42 at +100 mV. Events were categorised into the following types: 0: -∞ A ≤ Event 0 < 5.00e-11 A, 1: 5.00e-11 A ≤ Event 1 < 5.00e-10 A, 2: 5.00e-10 A ≤ Event 2 < 4.90e-08 A, 3: 4.90e-08 A ≤ Event 3 < ∞ A. Subfigure (d) shows the percentage of points found above the threshold -1.0e-11 (A) in a rolling look ahead window of 20 points. The threshold is the mean + standard deviation of the noise of the first 200 datapoints.

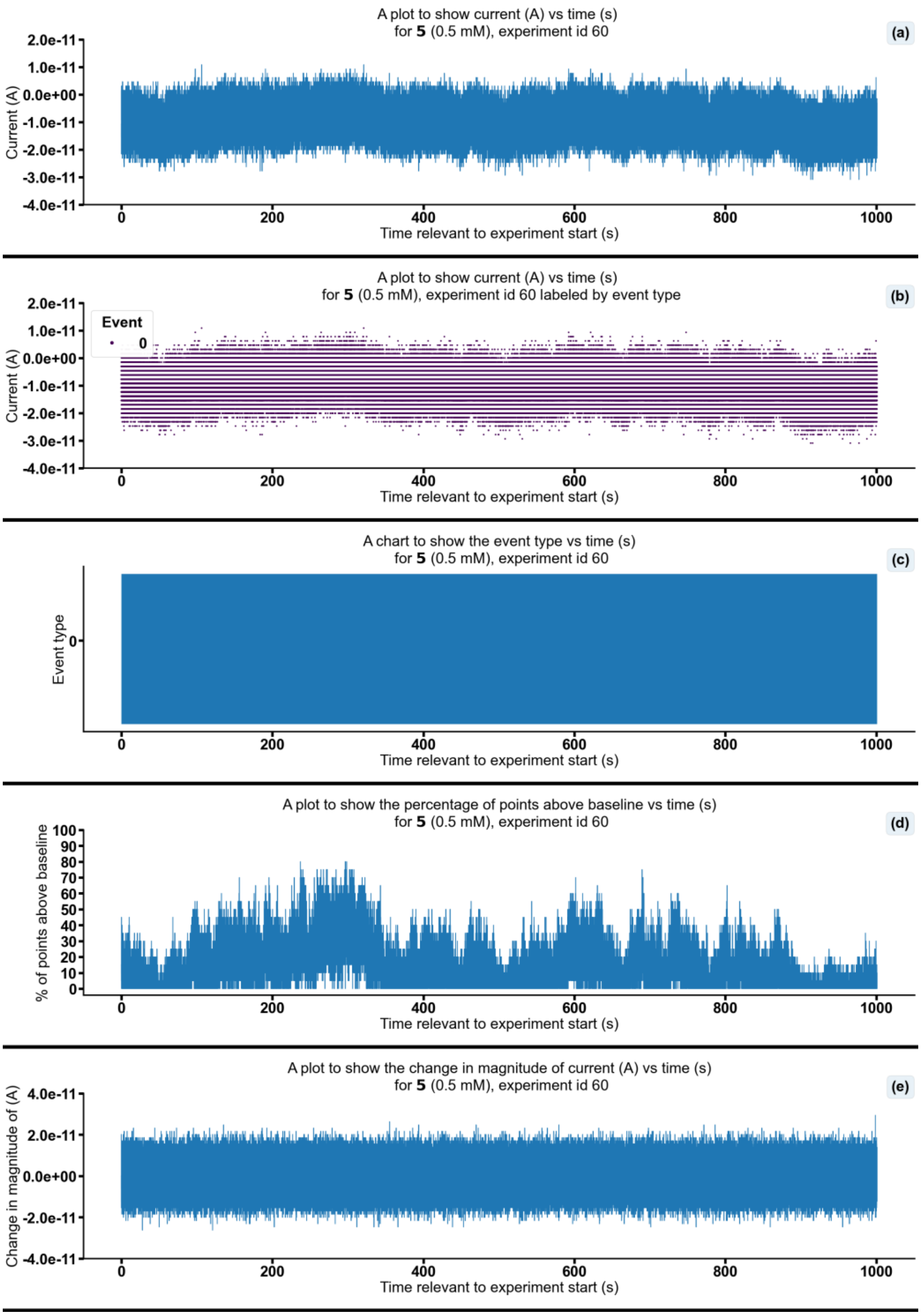

Figure S226 - Summarising the data from the patch clamp experiment for **5** (0.5 mM), experiment id 60 at +100 mV. Events were categorised into the following types: 0: -∞ A ≤ Event 0 < 5.00e-11 A, 1: 5.00e-11 A ≤ Event 1 < 5.00e-10 A, 2: 5.00e-10 A ≤ Event 2 < 4.90e-08 A, 3: 4.90e-08 A ≤ Event 3 < ∞ A. Subfigure (d) shows the percentage of points found above the threshold -4.7e-12 (A) in a rolling look ahead window of 20 points. The threshold is the mean + standard deviation of the noise of the first 200 datapoints.

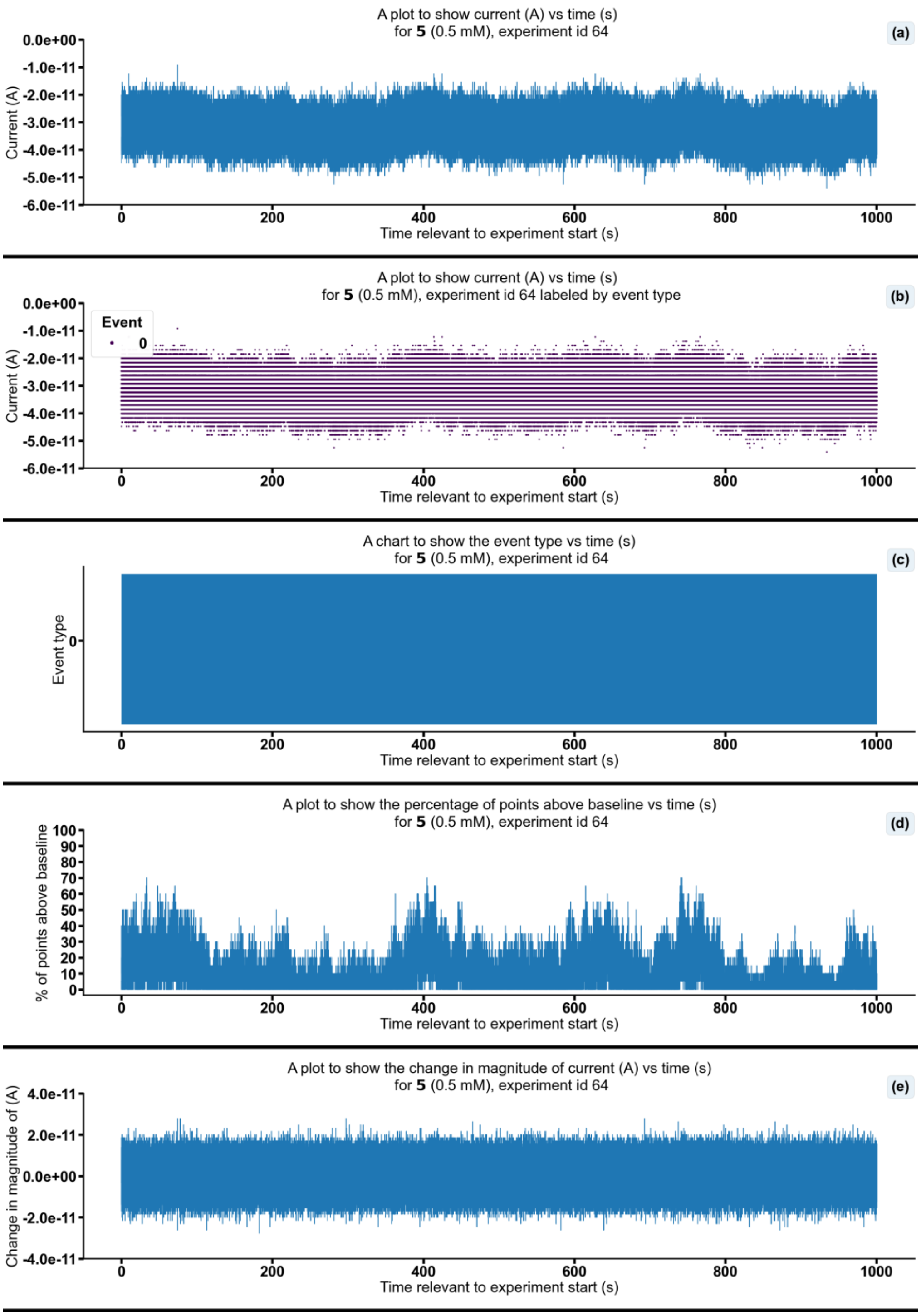

Figure S227 - Summarising the data from the patch clamp experiment for **5** (0.5 mM), experiment id 64 at +100 mV. Events were categorised into the following types: 0: -∞ A ≤ Event 0 < 5.00e-11 A, 1: 5.00e-11 A ≤ Event 1 < 5.00e-10 A, 2: 5.00e-10 A ≤ Event 2 < 4.90e-08 A, 3: 4.90e-08 A ≤ Event 3 < ∞ A. Subfigure (d) shows the percentage of points found above the threshold -2.7e-11 (A) in a rolling look ahead window of 20 points. The threshold is the mean + standard deviation of the noise of the first 200 datapoints.

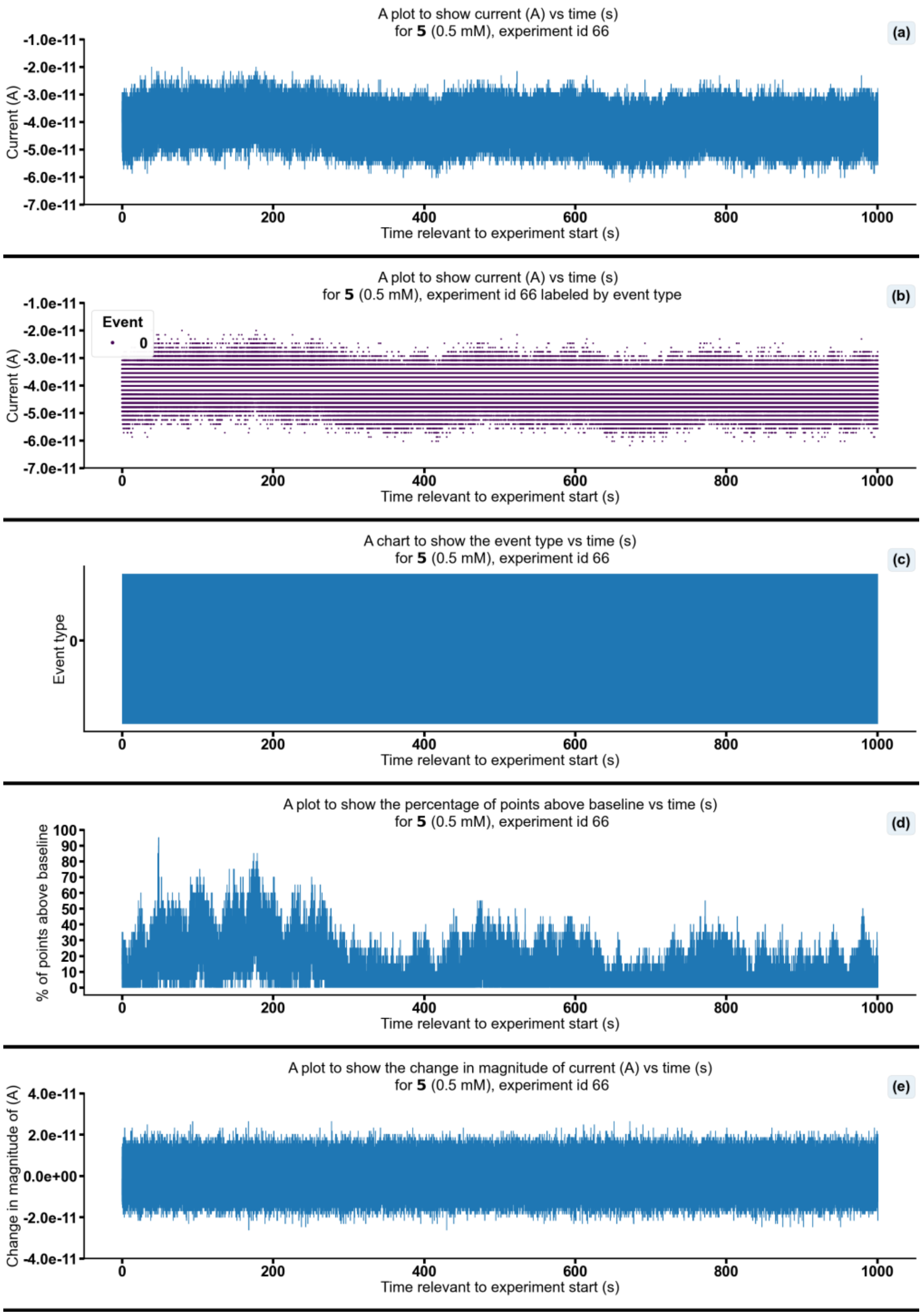

Figure S228 - Summarising the data from the patch clamp experiment for **5** (0.5 mM), experiment id 66 at +100 mV. Events were categorised into the following types: 0: -∞ A ≤ Event 0 < 5.00e-11 A, 1: 5.00e-11 A ≤ Event 1 < 5.00e-10 A, 2: 5.00e-10 A ≤ Event 2 < 4.90e-08 A, 3: 4.90e-08 A ≤ Event 3 < ∞ A. Subfigure (d) shows the percentage of points found above the threshold -3.6e-11 (A) in a rolling look ahead window of 20 points. The threshold is the mean + standard deviation of the noise of the first 200 datapoints.

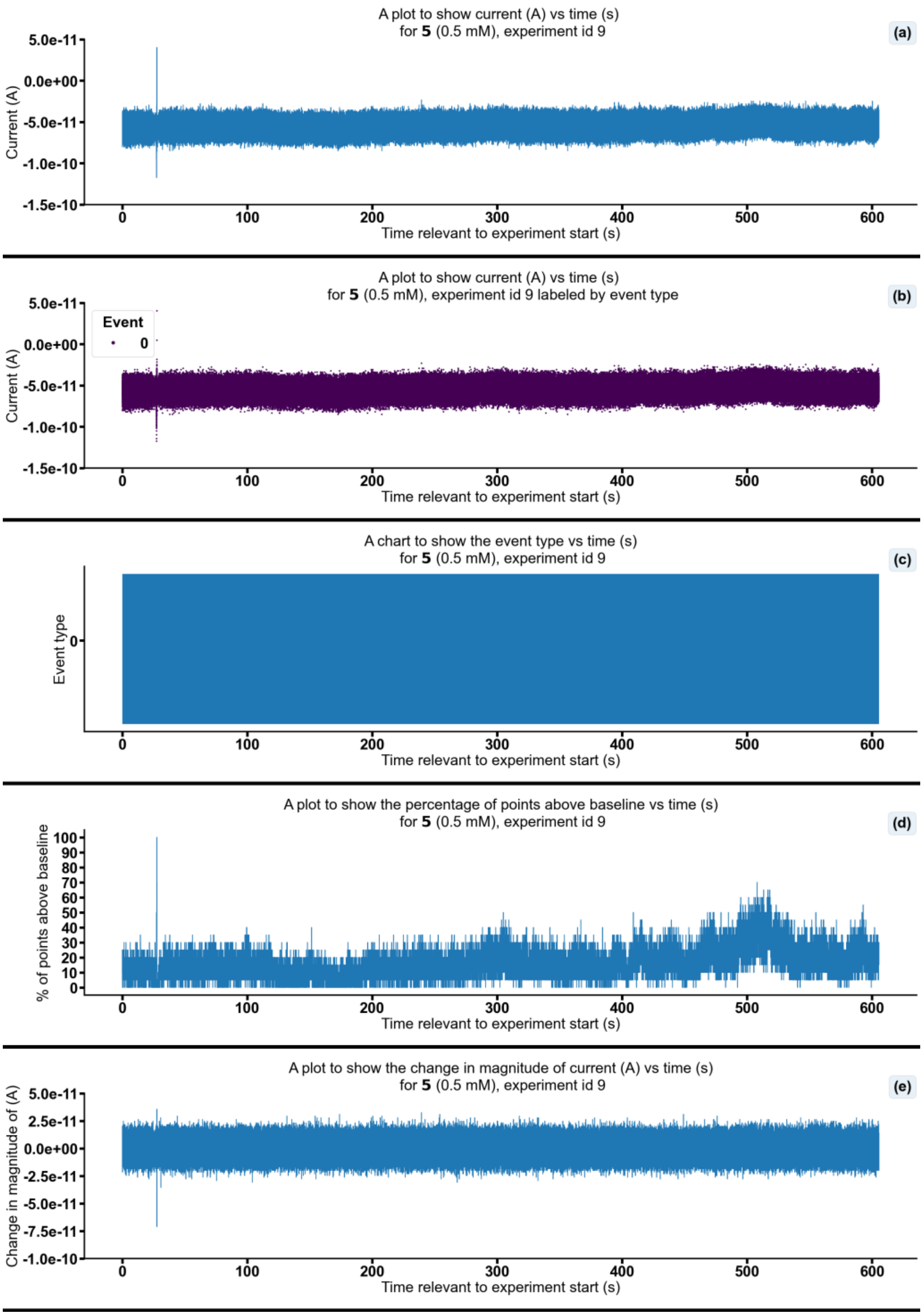

Figure S229 - Summarising the data from the patch clamp experiment for **5** (0.5 mM), experiment id 9 at +100 mV. Events were categorised into the following types: 0: -∞ A ≤ Event 0 < 5.00e-11 A, 1: 5.00e-11 A ≤ Event 1 < 5.00e-10 A, 2: 5.00e-10 A ≤ Event 2 < 4.90e-08 A, 3: 4.90e-08 A ≤ Event 3 < ∞ A. Subfigure (d) shows the percentage of points found above the threshold -4.8e-11 (A) in a rolling look ahead window of 20 points. The threshold is the mean + standard deviation of the noise of the first 200 datapoints.

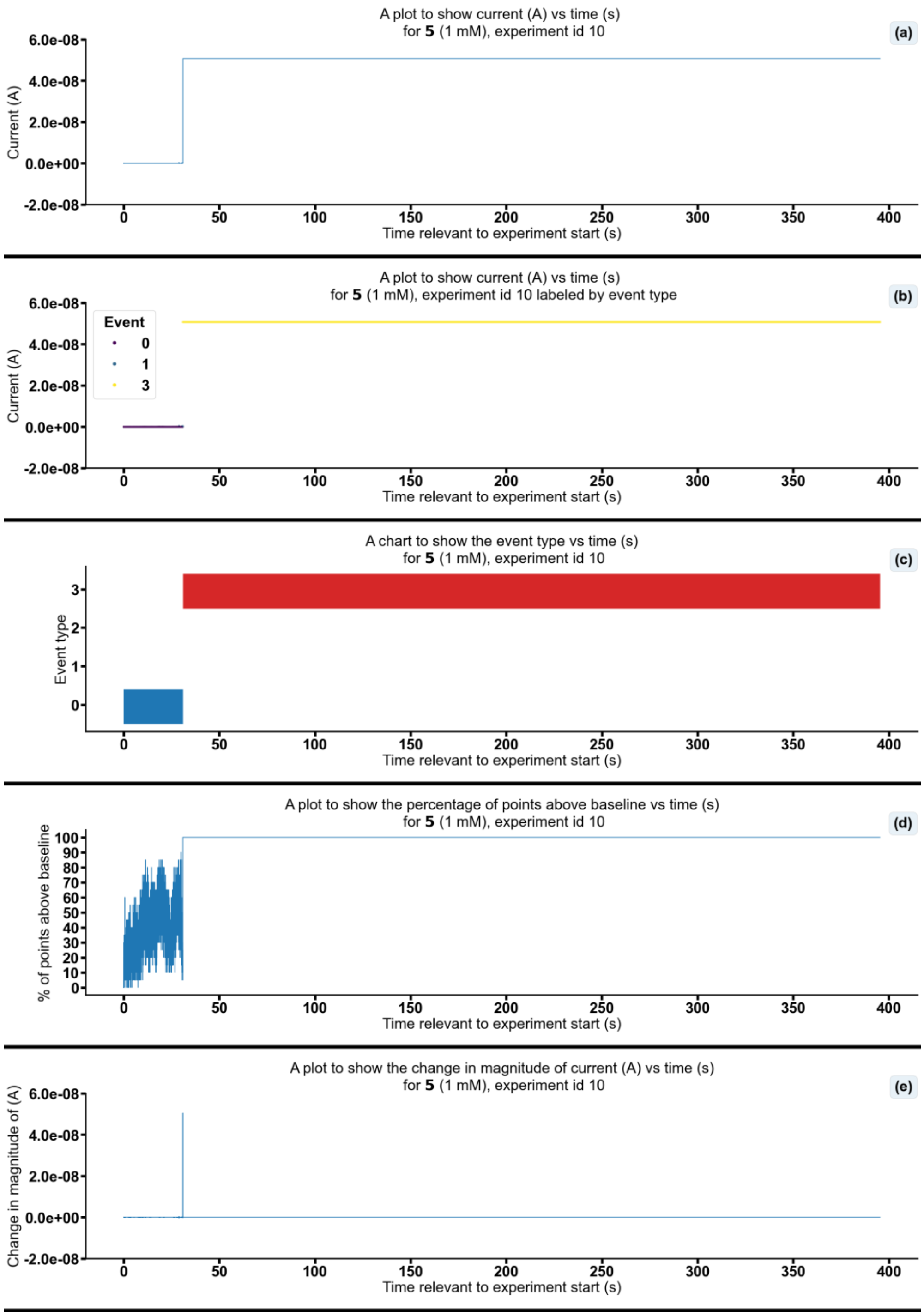

Figure S230 - Summarising the data from the patch clamp experiment for **5** (1 mM), experiment id 10 at +100 mV. Events were categorised into the following types: 0: -∞ A ≤ Event 0 < 5.00e-11 A, 1: 5.00e-11 A ≤ Event 1 < 5.00e-10 A, 2: 5.00e-10 A ≤ Event 2 < 4.90e-08 A, 3: 4.90e-08 A ≤ Event 3 < ∞ A. Subfigure (d) shows the percentage of points found above the threshold -1.3e-11 (A) in a rolling look ahead window of 20 points. The threshold is the mean + standard deviation of the noise of the first 200 datapoints.

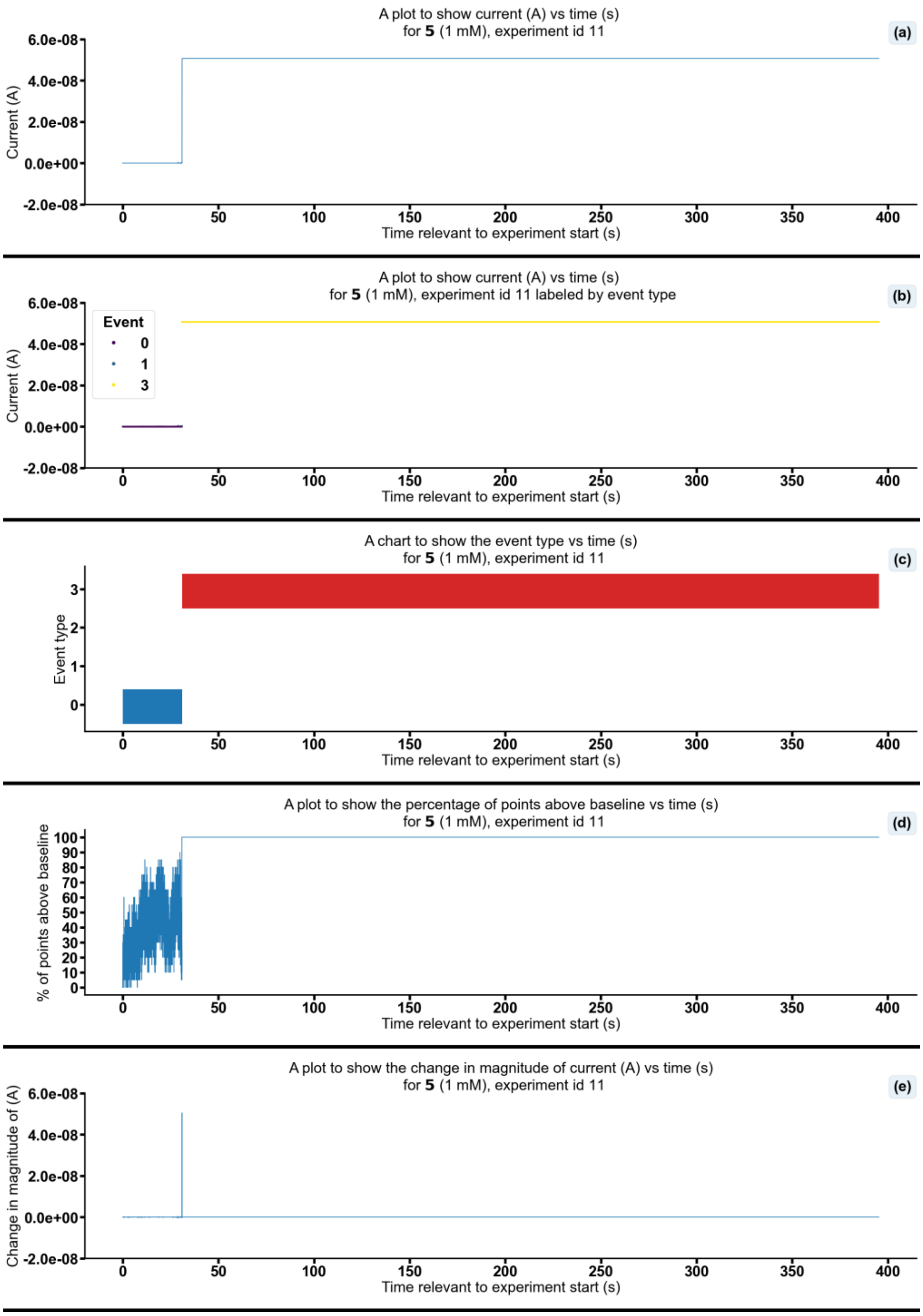

Figure S231 - Summarising the data from the patch clamp experiment for **5** (1 mM), experiment id 11 at +100 mV. Events were categorised into the following types: 0: -∞ A ≤ Event 0 < 5.00e-11 A, 1: 5.00e-11 A ≤ Event 1 < 5.00e-10 A, 2: 5.00e-10 A ≤ Event 2 < 4.90e-08 A, 3: 4.90e-08 A ≤ Event 3 < ∞ A. Subfigure (d) shows the percentage of points found above the threshold -1.3e-11 (A) in a rolling look ahead window of 20 points. The threshold is the mean + standard deviation of the noise of the first 200 datapoints.

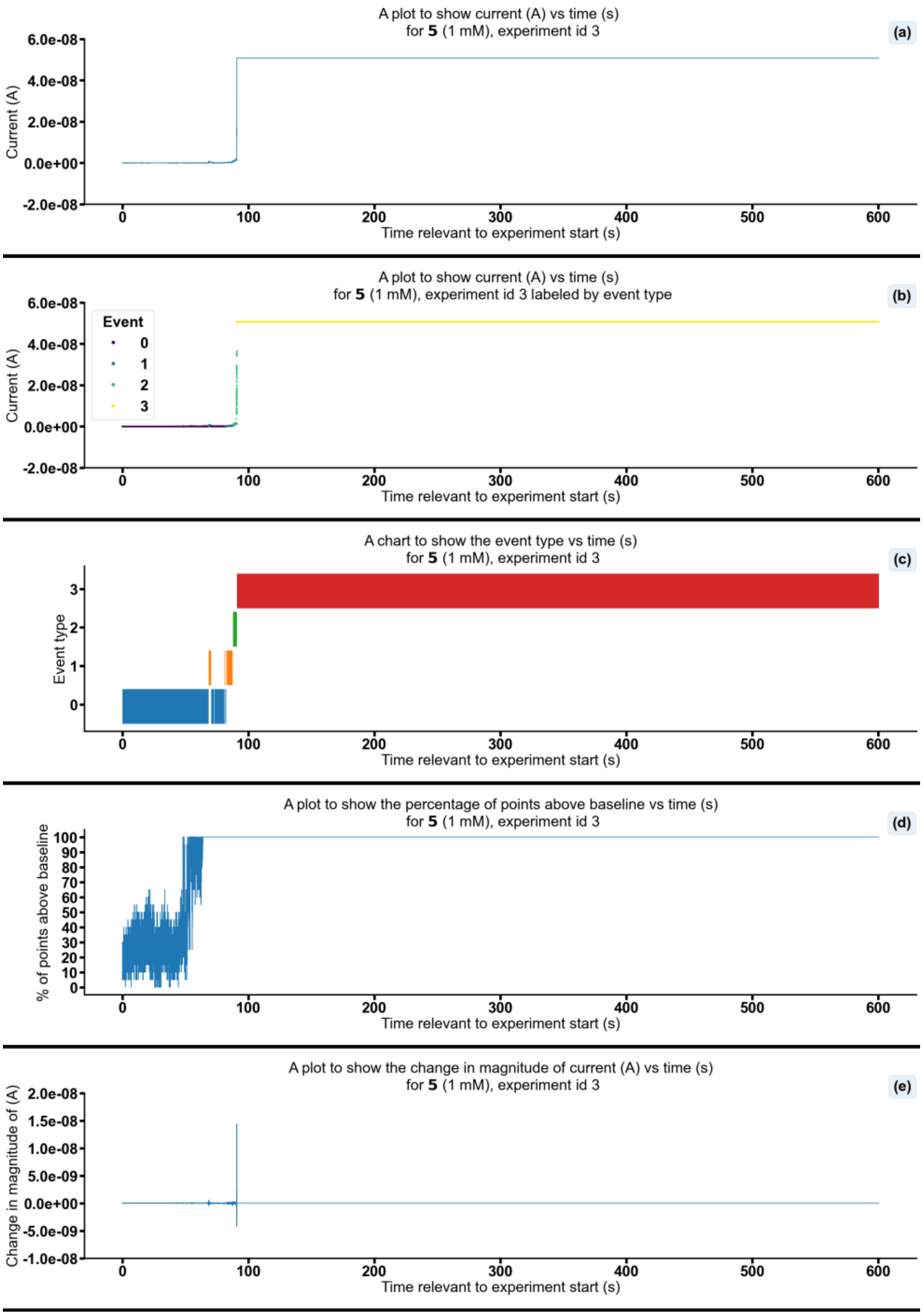

Figure S232 - Summarising the data from the patch clamp experiment for **5** (1 mM), experiment id 3 at +100 mV. Events were categorised into the following types: 0: -∞ A ≤ Event 0 < 5.00e-11 A, 1: 5.00e-11 A ≤ Event 1 < 5.00e-10 A, 2: 5.00e-10 A ≤ Event 2 < 4.90e-08 A, 3: 4.90e-08 A ≤ Event 3 < ∞ A. Subfigure (d) shows the percentage of points found above the threshold -2.7e-11 (A) in a rolling look ahead window of 20 points. The threshold is the mean + standard deviation of the noise of the first 200 datapoints.

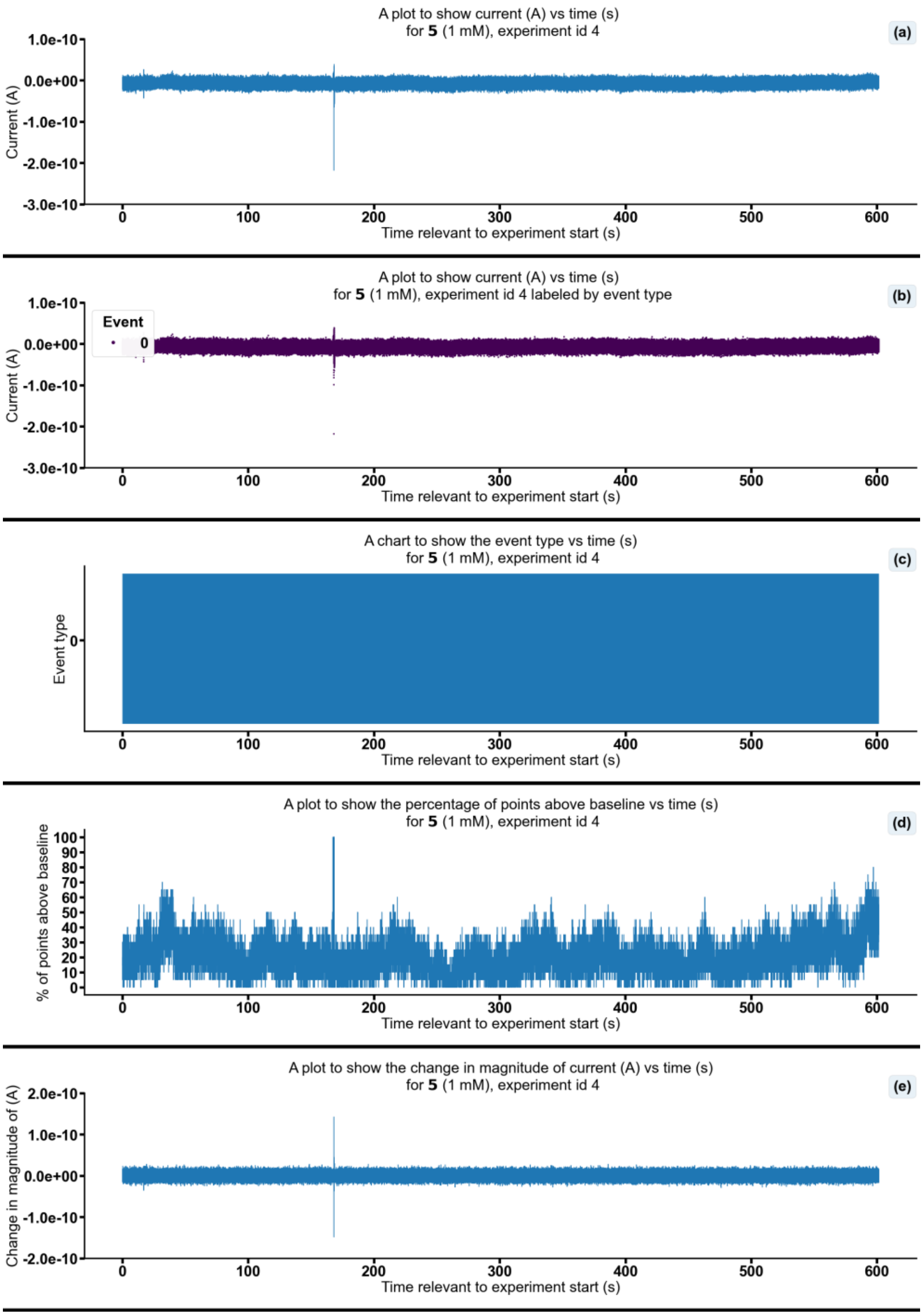

Figure S233 - Summarising the data from the patch clamp experiment for **5** (1 mM), experiment id 4 at +100 mV. Events were categorised into the following types: 0: -∞ A ≤ Event 0 < 5.00e-11 A, 1: 5.00e-11 A ≤ Event 1 < 5.00e-10 A, 2: 5.00e-10 A ≤ Event 2 < 4.90e-08 A, 3: 4.90e-08 A ≤ Event 3 < ∞ A. Subfigure (d) shows the percentage of points found above the threshold -2.1e-12 (A) in a rolling look ahead window of 20 points. The threshold is the mean + standard deviation of the noise of the first 200 datapoints.

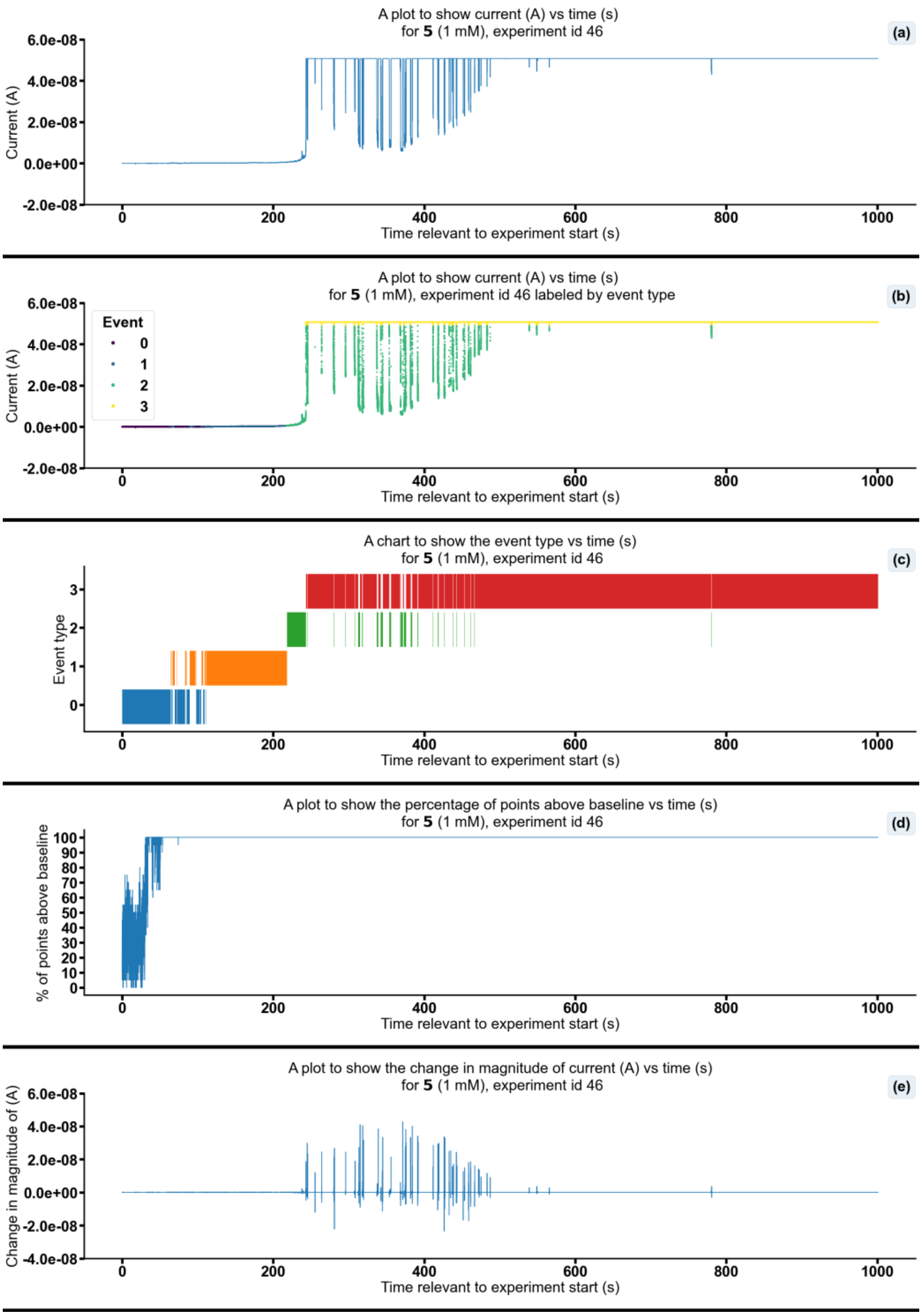

Figure S234 - Summarising the data from the patch clamp experiment for **5** (1 mM), experiment id 46 at +100 mV. Events were categorised into the following types: 0: -∞ A ≤ Event 0 < 5.00e-11 A, 1: 5.00e-11 A ≤ Event 1 < 5.00e-10 A, 2: 5.00e-10 A ≤ Event 2 < 4.90e-08 A, 3: 4.90e-08 A ≤ Event 3 < ∞ A. Subfigure (d) shows the percentage of points found above the threshold -6.3e-12 (A) in a rolling look ahead window of 20 points. The threshold is the mean + standard deviation of the noise of the first 200 datapoints.

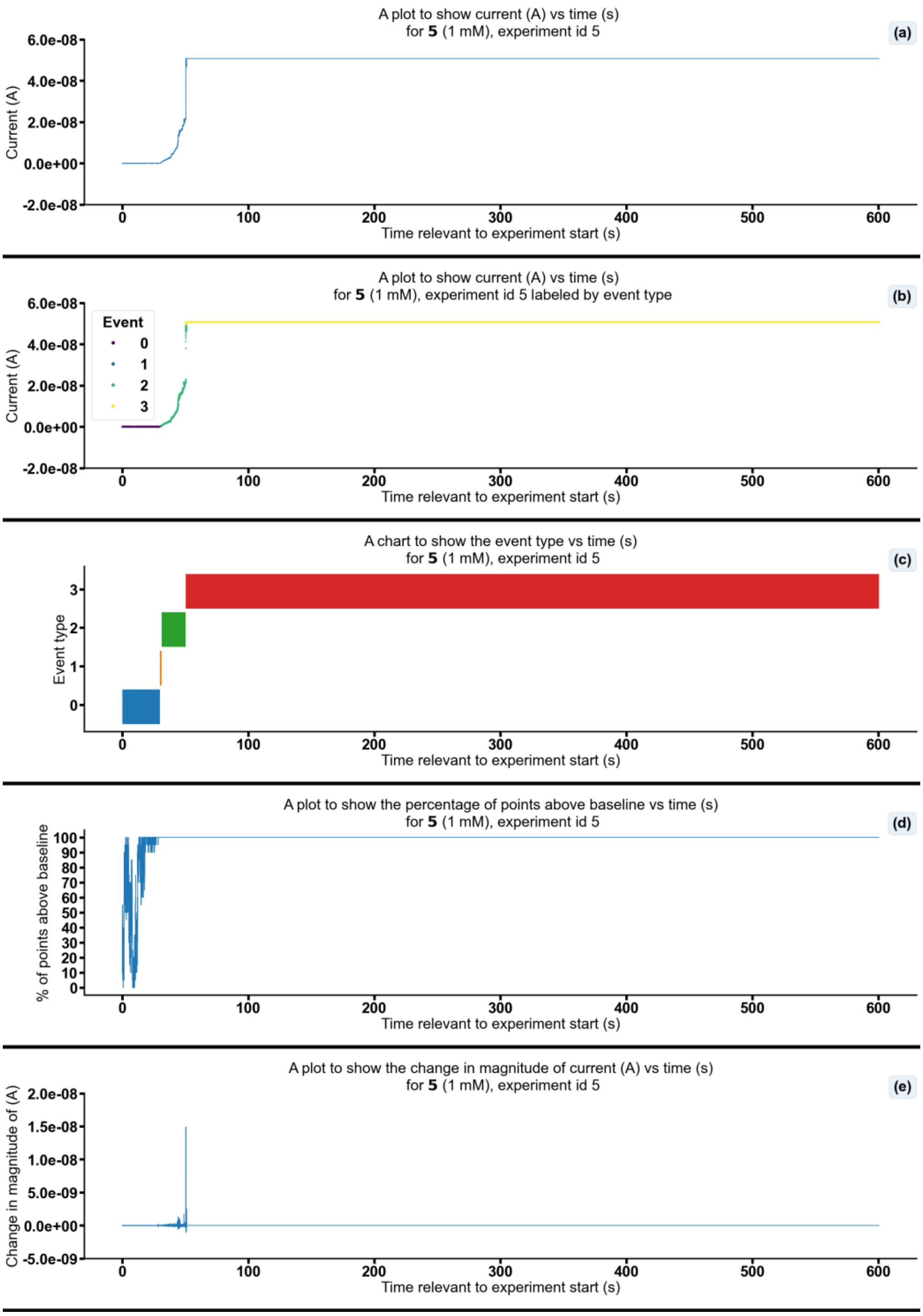

Figure S235 - Summarising the data from the patch clamp experiment for **5** (1 mM), experiment id 5 at +100 mV. Events were categorised into the following types: 0: -∞ A ≤ Event 0 < 5.00e-11 A, 1: 5.00e-11 A ≤ Event 1 < 5.00e-10 A, 2: 5.00e-10 A ≤ Event 2 < 4.90e-08 A, 3: 4.90e-08 A ≤ Event 3 < ∞ A. Subfigure (d) shows the percentage of points found above the threshold -4.7e-12 (A) in a rolling look ahead window of 20 points. The threshold is the mean + standard deviation of the noise of the first 200 datapoints.

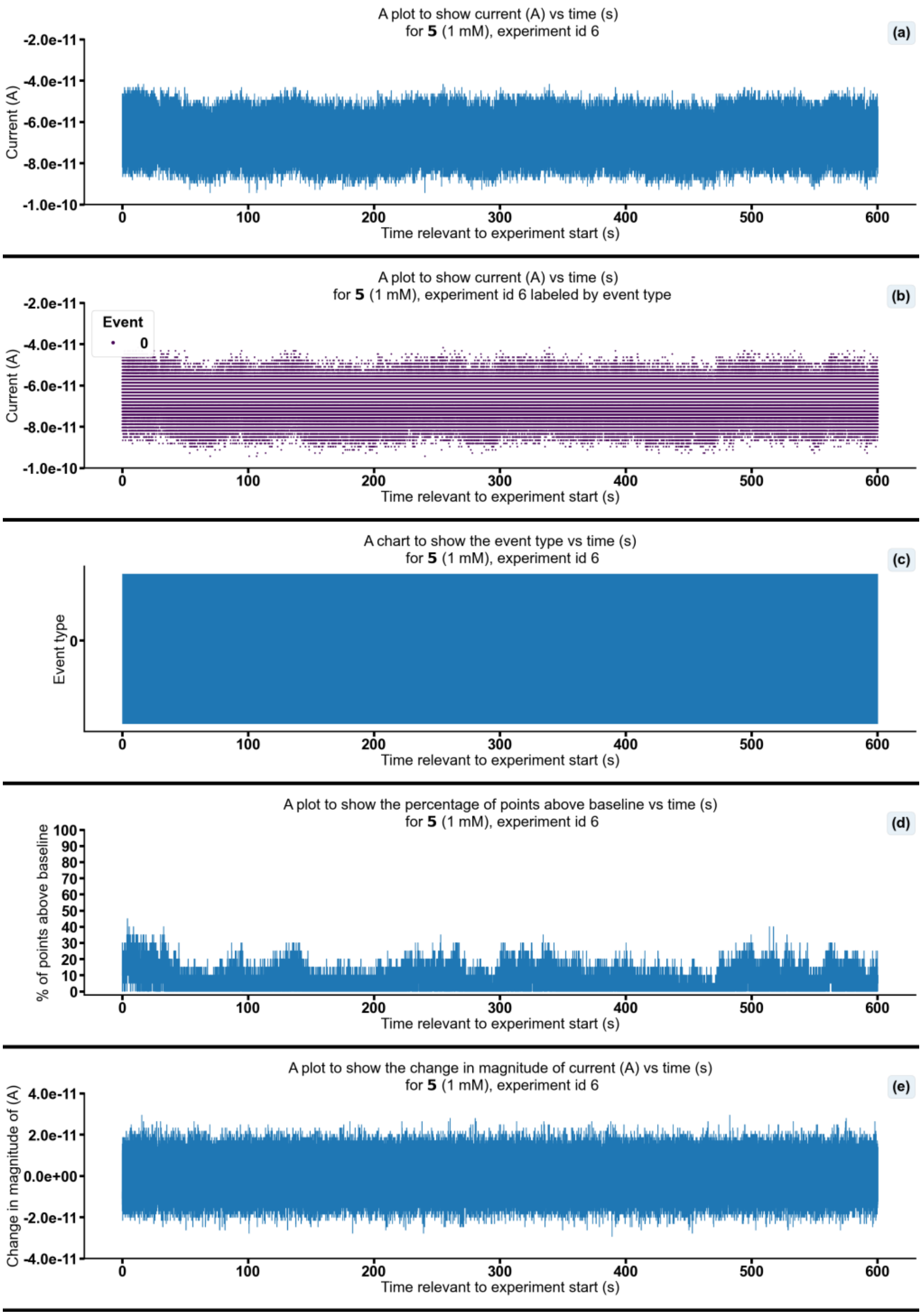

Figure S236 - Summarising the data from the patch clamp experiment for **5** (1 mM), experiment id 6 at +100 mV. Events were categorised into the following types: 0: -∞ A ≤ Event 0 < 5.00e-11 A, 1: 5.00e-11 A ≤ Event 1 < 5.00e-10 A, 2: 5.00e-10 A ≤ Event 2 < 4.90e-08 A, 3: 4.90e-08 A ≤ Event 3 < ∞ A. Subfigure (d) shows the percentage of points found above the threshold -5.8e-11 (A) in a rolling look ahead window of 20 points. The threshold is the mean + standard deviation of the noise of the first 200 datapoints.

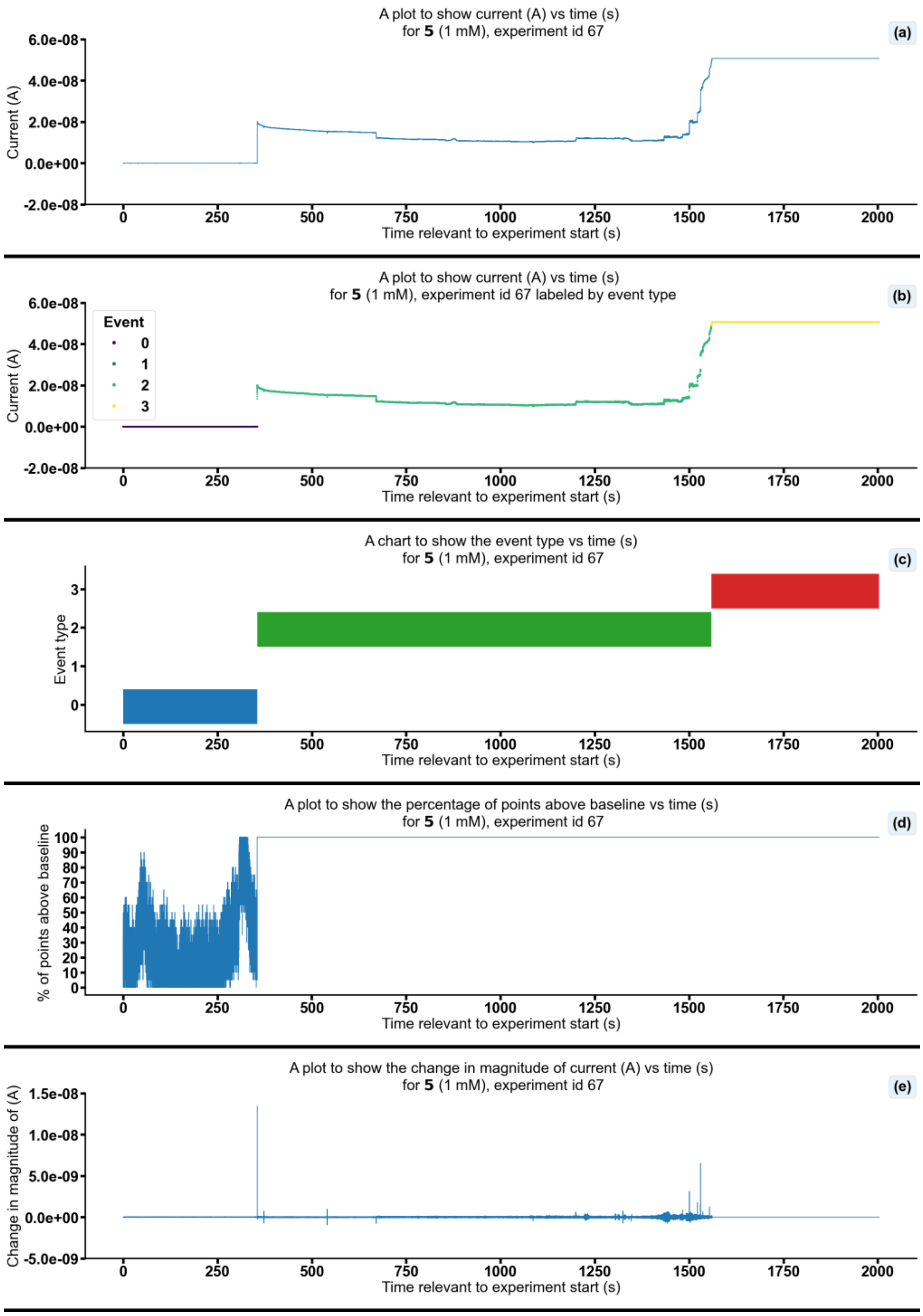

Figure S237 - Summarising the data from the patch clamp experiment for **5** (1 mM), experiment id 67 at +100 mV. Events were categorised into the following types: 0: -∞ A ≤ Event 0 < 5.00e-11 A, 1: 5.00e-11 A ≤ Event 1 < 5.00e-10 A, 2: 5.00e-10 A ≤ Event 2 < 4.90e-08 A, 3: 4.90e-08 A ≤ Event 3 < ∞ A. Subfigure (d) shows the percentage of points found above the threshold -2.0e-11 (A) in a rolling look ahead window of 20 points. The threshold is the mean + standard deviation of the noise of the first 200 datapoints.

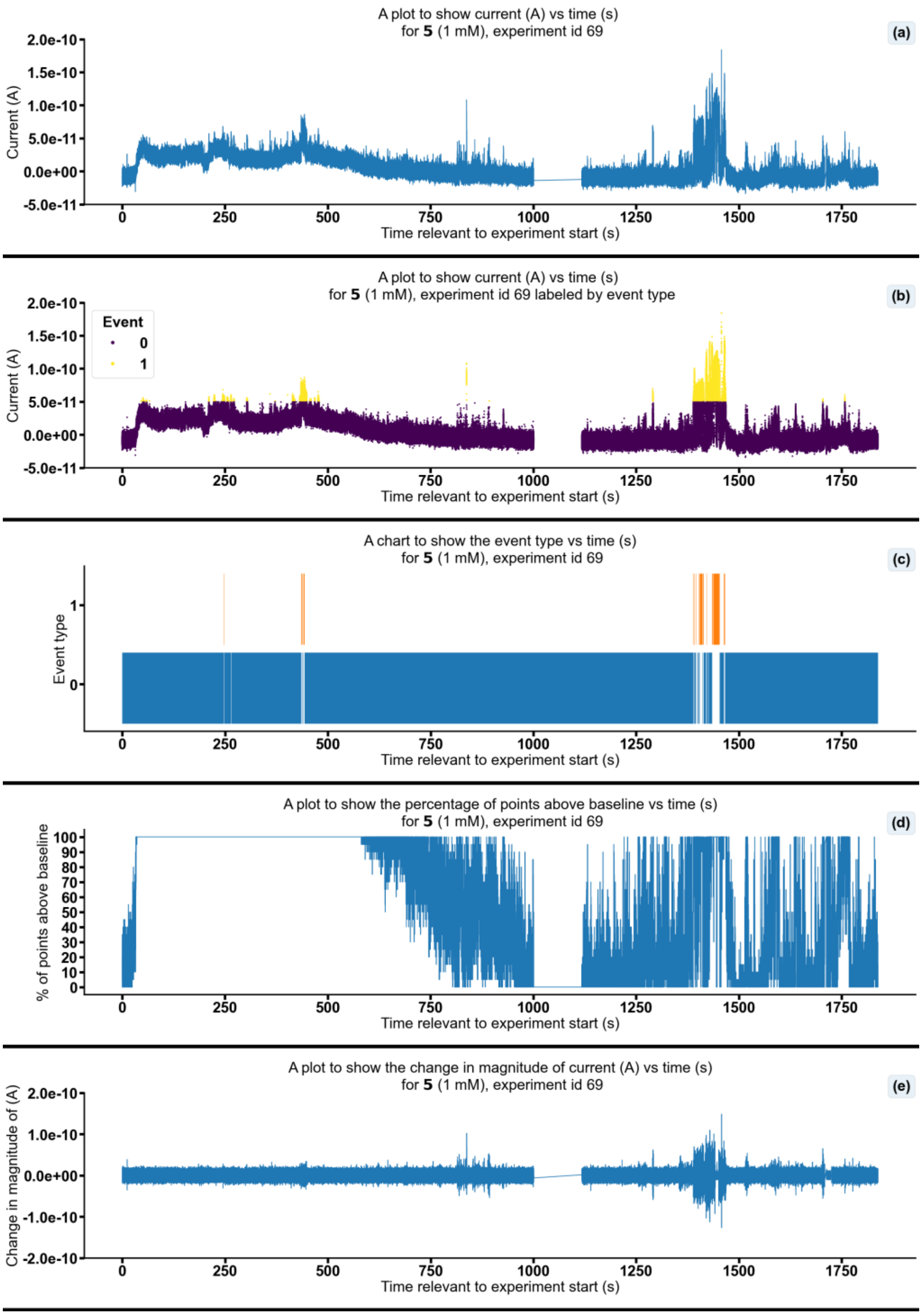

Figure S238 - Summarising the data from the patch clamp experiment for **5** (1 mM), experiment id 69 at +100 mV. Events were categorised into the following types: 0: -∞ A ≤ Event 0 < 5.00e-11 A, 1: 5.00e-11 A ≤ Event 1 < 5.00e-10 A, 2: 5.00e-10 A ≤ Event 2 < 4.90e-08 A, 3: 4.90e-08 A ≤ Event 3 < ∞ A. Subfigure (d) shows the percentage of points found above the threshold -4.2e-12 (A) in a rolling look ahead window of 20 points. The threshold is the mean + standard deviation of the noise of the first 200 datapoints.

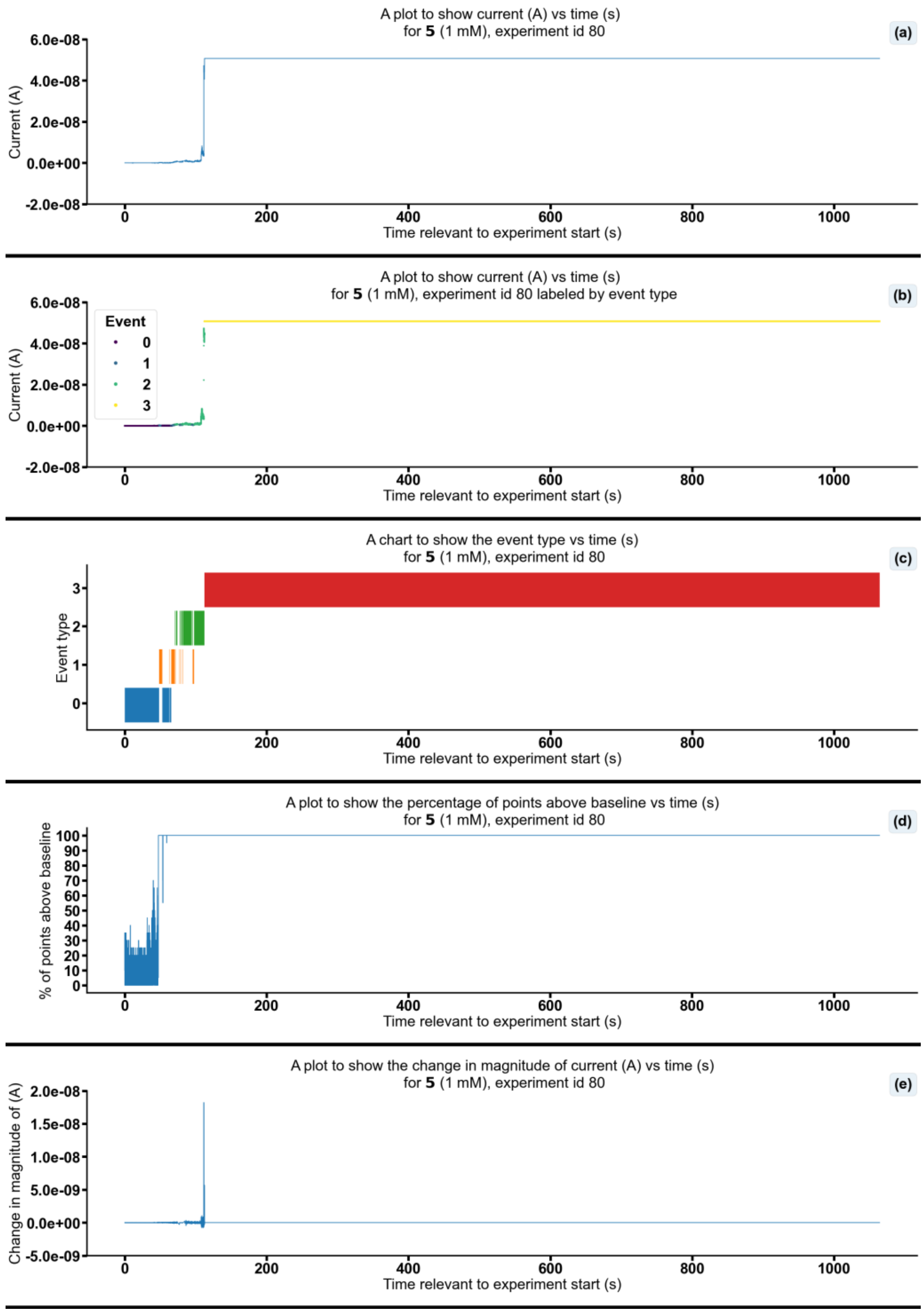

Figure S239 - Summarising the data from the patch clamp experiment for **5** (1 mM), experiment id 80 at +100 mV. Events were categorised into the following types: 0: -∞ A ≤ Event 0 < 5.00e-11 A, 1: 5.00e-11 A ≤ Event 1 < 5.00e-10 A, 2: 5.00e-10 A ≤ Event 2 < 4.90e-08 A, 3: 4.90e-08 A ≤ Event 3 < ∞ A. Subfigure (d) shows the percentage of points found above the threshold -1.3e-11 (A) in a rolling look ahead window of 20 points. The threshold is the mean + standard deviation of the noise of the first 200 datapoints.

Changing holding voltage with **5**

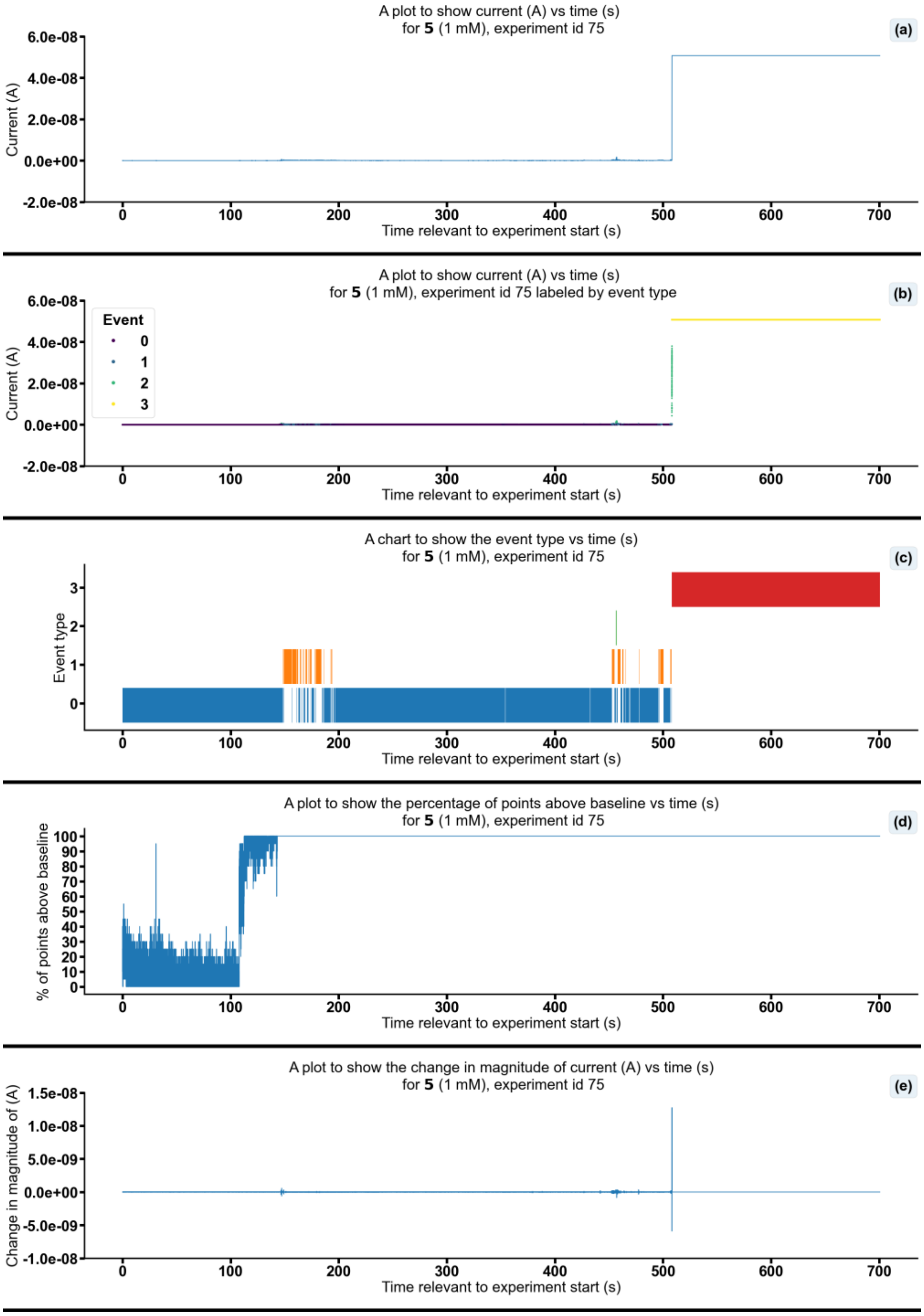

Figure S240 - Summarising the data from the patch clamp experiment for **5** (1 mM), experiment id 75 at +100 mV. Events were categorised into the following types: 0: -∞ A ≤ Event 0 < 5.00e-11 A, 1: 5.00e-11 A ≤ Event 1 < 5.00e-10 A, 2: 5.00e-10 A ≤ Event 2 < 4.90e-08 A, 3: 4.90e-08 A ≤ Event 3 < ∞ A. Subfigure (d) shows the percentage of points found above the threshold -3.6e-11 (A) in a rolling look ahead window of 20 points. The threshold is the mean + standard deviation of the noise of the first 200 datapoints.

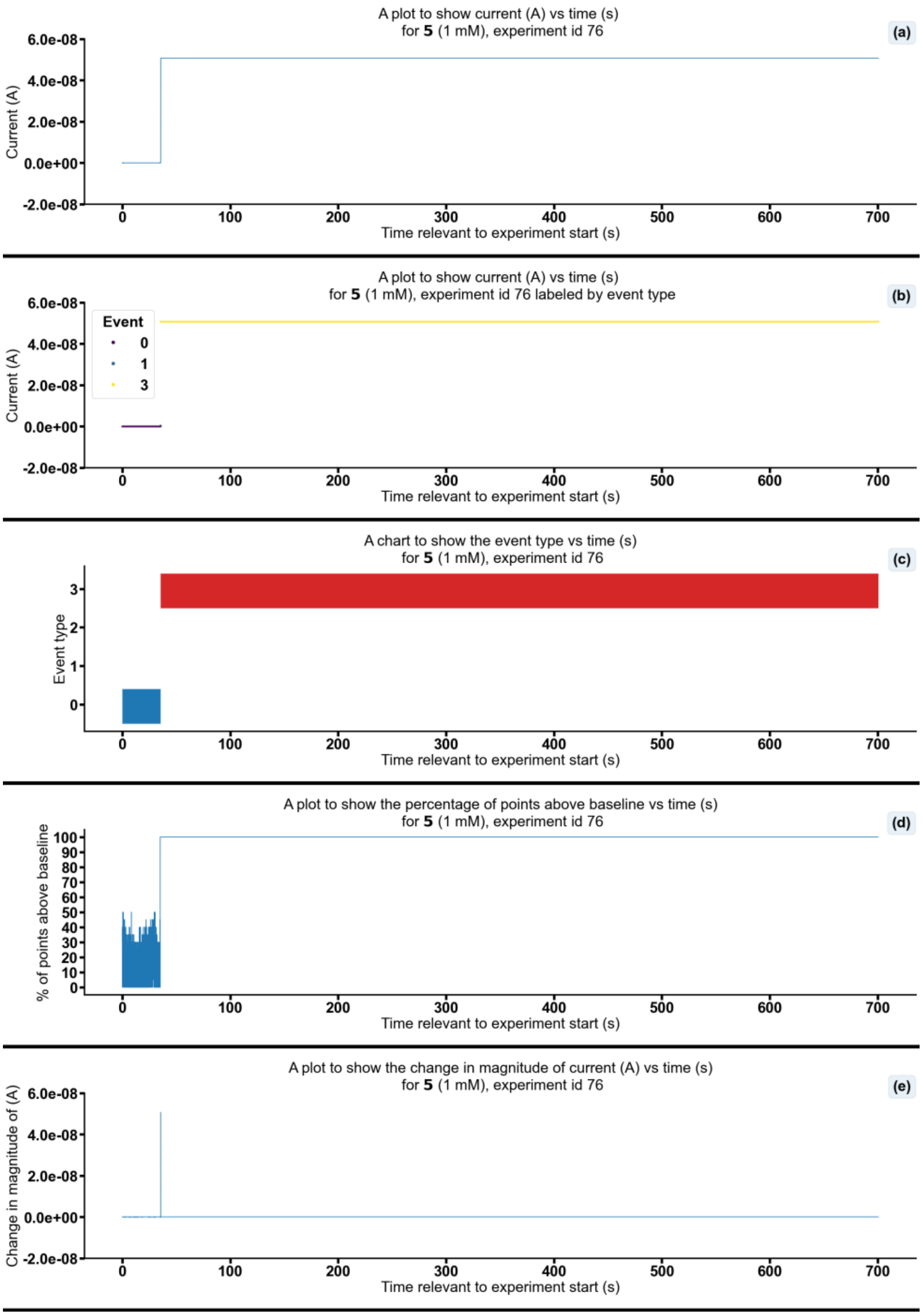

Figure S241 - Summarising the data from the patch clamp experiment for **5** (1 mM), experiment id 76 at +100 mV. Events were categorised into the following types: 0: -∞ A ≤ Event 0 < 5.00e-11 A, 1: 5.00e-11 A ≤ Event 1 < 5.00e-10 A, 2: 5.00e-10 A ≤ Event 2 < 4.90e-08 A, 3: 4.90e-08 A ≤ Event 3 < ∞ A. Subfigure (d) shows the percentage of points found above the threshold -3.4e-11 (A) in a rolling look ahead window of 20 points. The threshold is the mean + standard deviation of the noise of the first 200 datapoints.

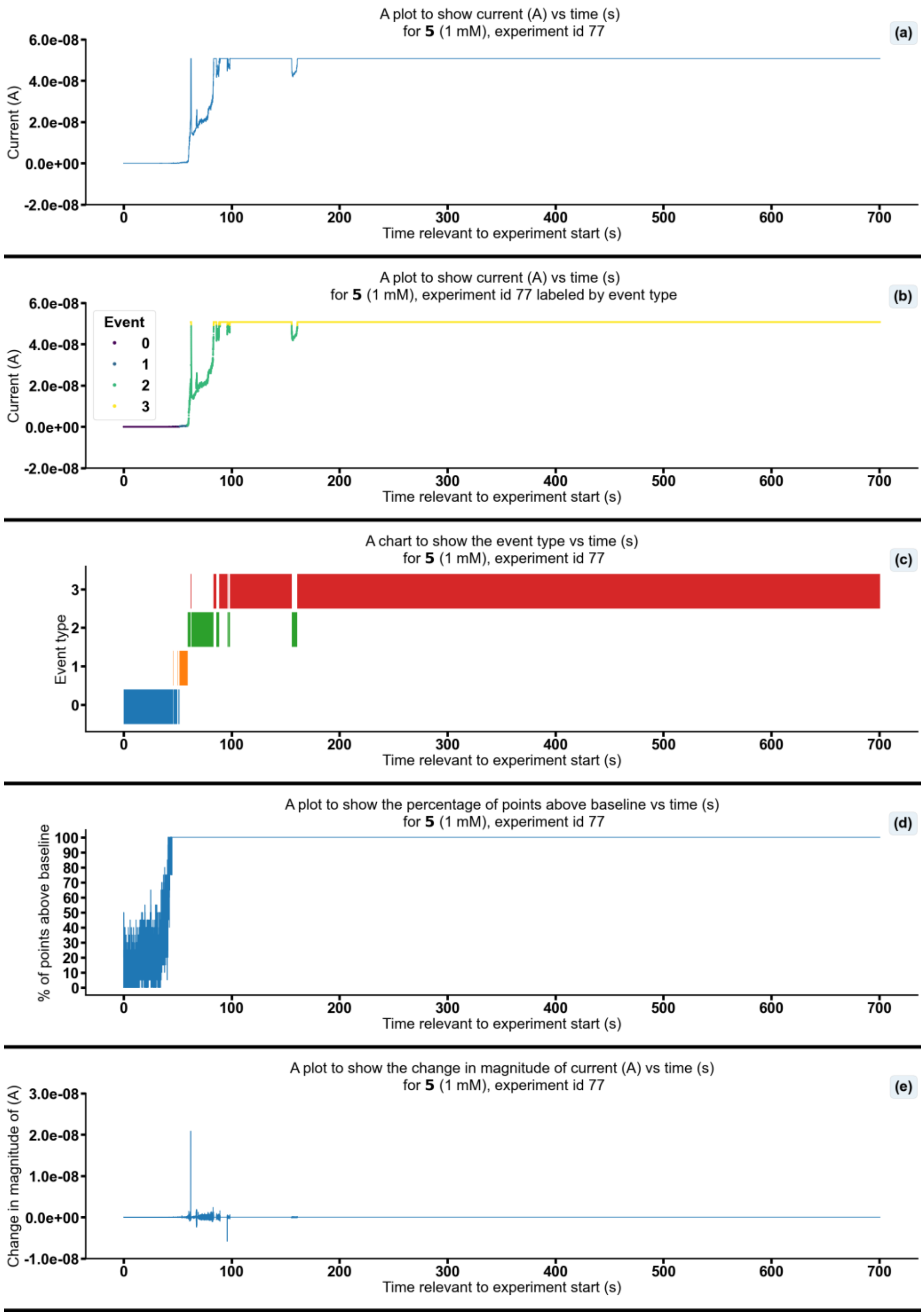

Figure S242 - Summarising the data from the patch clamp experiment for **5** (1 mM), experiment id 77 at +100 mV. Events were categorised into the following types: 0: -∞ A ≤ Event 0 < 5.00e-11 A, 1: 5.00e-11 A ≤ Event 1 < 5.00e-10 A, 2: 5.00e-10 A ≤ Event 2 < 4.90e-08 A, 3: 4.90e-08 A ≤ Event 3 < ∞ A. Subfigure (d) shows the percentage of points found above the threshold -3.2e-11 (A) in a rolling look ahead window of 20 points. The threshold is the mean + standard deviation of the noise of the first 200 datapoints.

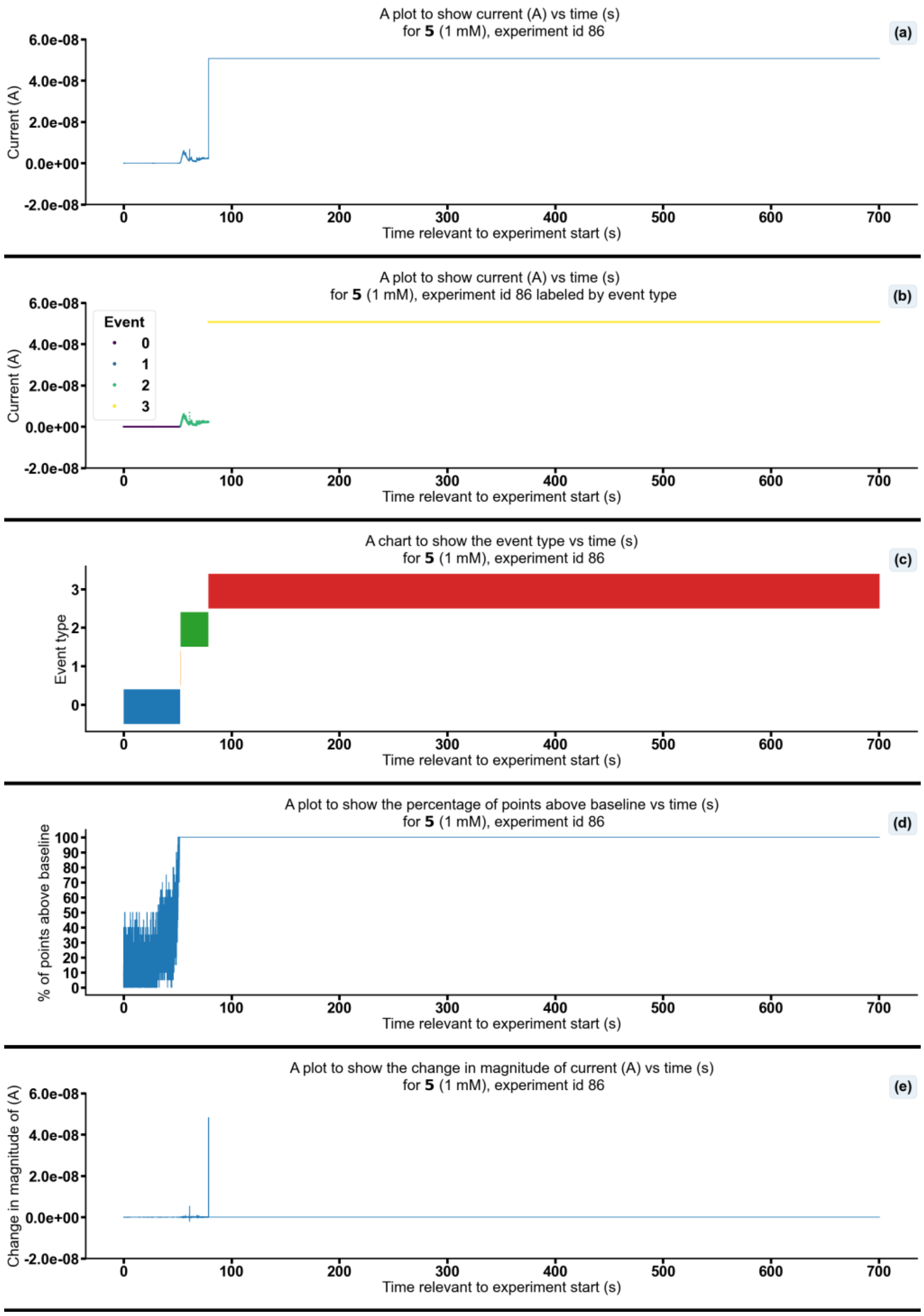

Figure S243 - Summarising the data from the patch clamp experiment for **5** (1 mM), experiment id 86 at +100 mV. Events were categorised into the following types: 0: -∞ A ≤ Event 0 < 5.00e-11 A, 1: 5.00e-11 A ≤ Event 1 < 5.00e-10 A, 2: 5.00e-10 A ≤ Event 2 < 4.90e-08 A, 3: 4.90e-08 A ≤ Event 3 < ∞ A. Subfigure (d) shows the percentage of points found above the threshold -3.1e-11 (A) in a rolling look ahead window of 20 points. The threshold is the mean + standard deviation of the noise of the first 200 datapoints.

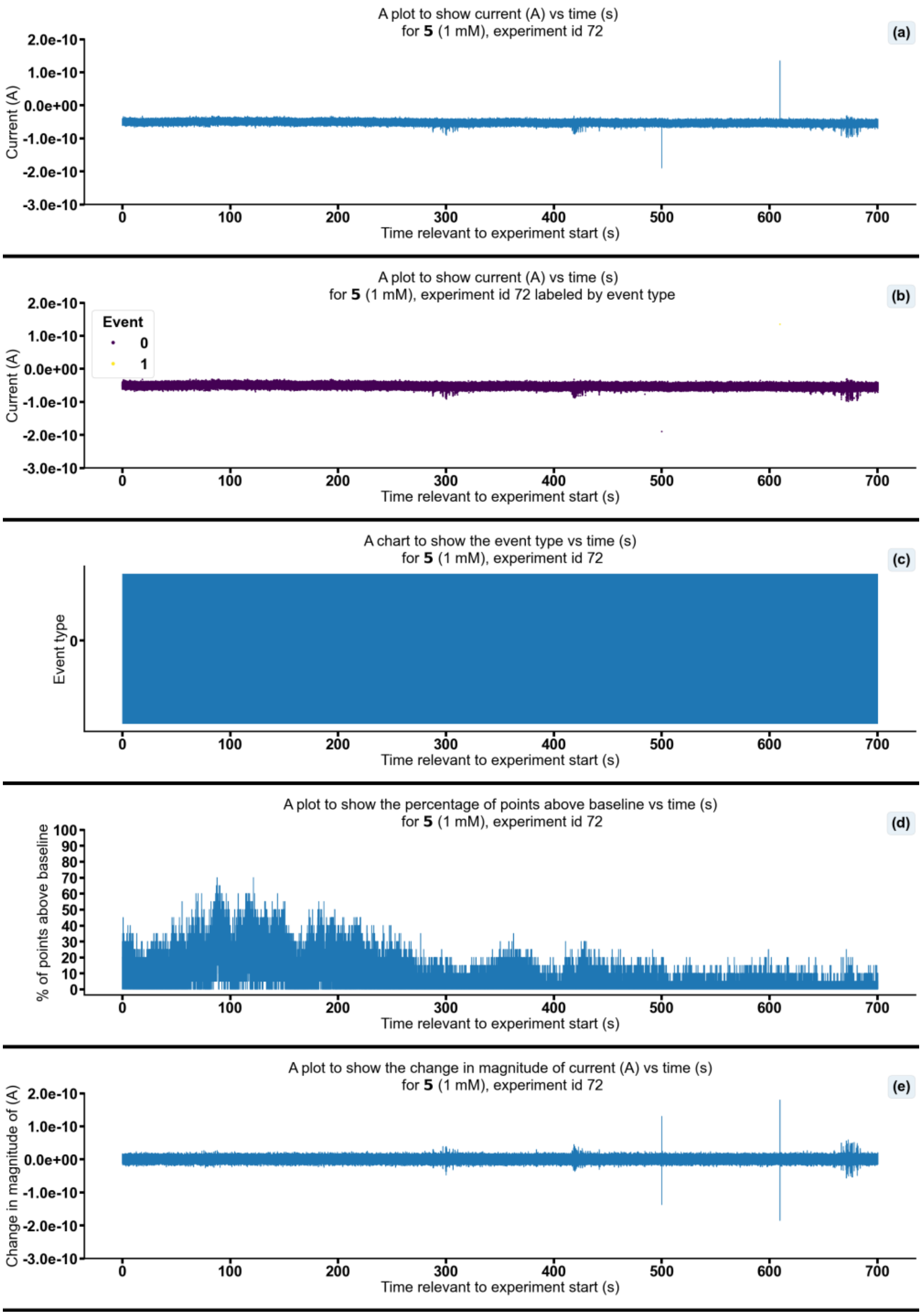

Figure S244 - Summarising the data from the patch clamp experiment for **5** (1 mM), experiment id 72 at +50 mV. Events were categorised into the following types: 0: -∞ A ≤ Event 0 < 2.50e-11 A, 1: 2.50e-11 A ≤ Event 1 < 2.50e-10 A, 2: 2.50e-10 A ≤ Event 2 < 4.90e-08 A, 3: 4.90e-08 A ≤ Event 3 < ∞ A. Subfigure (d) shows the percentage of points found above the threshold -4.6e-11 (A) in a rolling look ahead window of 20 points. The threshold is the mean + standard deviation of the noise of the first 200 datapoints.

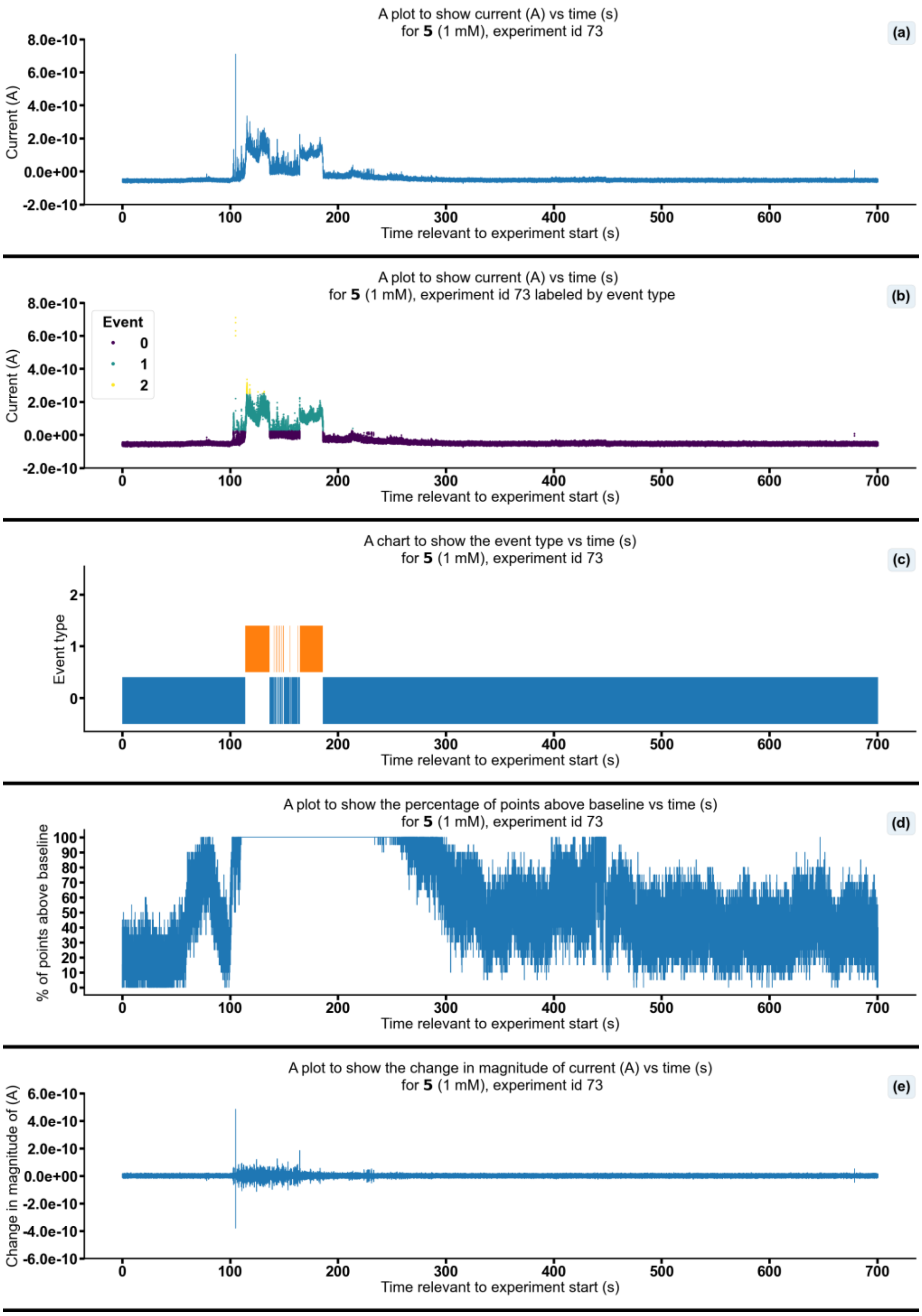

Figure S245 - Summarising the data from the patch clamp experiment for **5** (1 mM), experiment id 73 at +50 mV. Events were categorised into the following types: 0: -∞ A ≤ Event 0 < 2.50e-11 A, 1: 2.50e-11 A ≤ Event 1 < 2.50e-10 A, 2: 2.50e-10 A ≤ Event 2 < 4.90e-08 A, 3: 4.90e-08 A ≤ Event 3 < ∞ A. Subfigure (d) shows the percentage of points found above the threshold -5.3e-11 (A) in a rolling look ahead window of 20 points. The threshold is the mean + standard deviation of the noise of the first 200 datapoints.

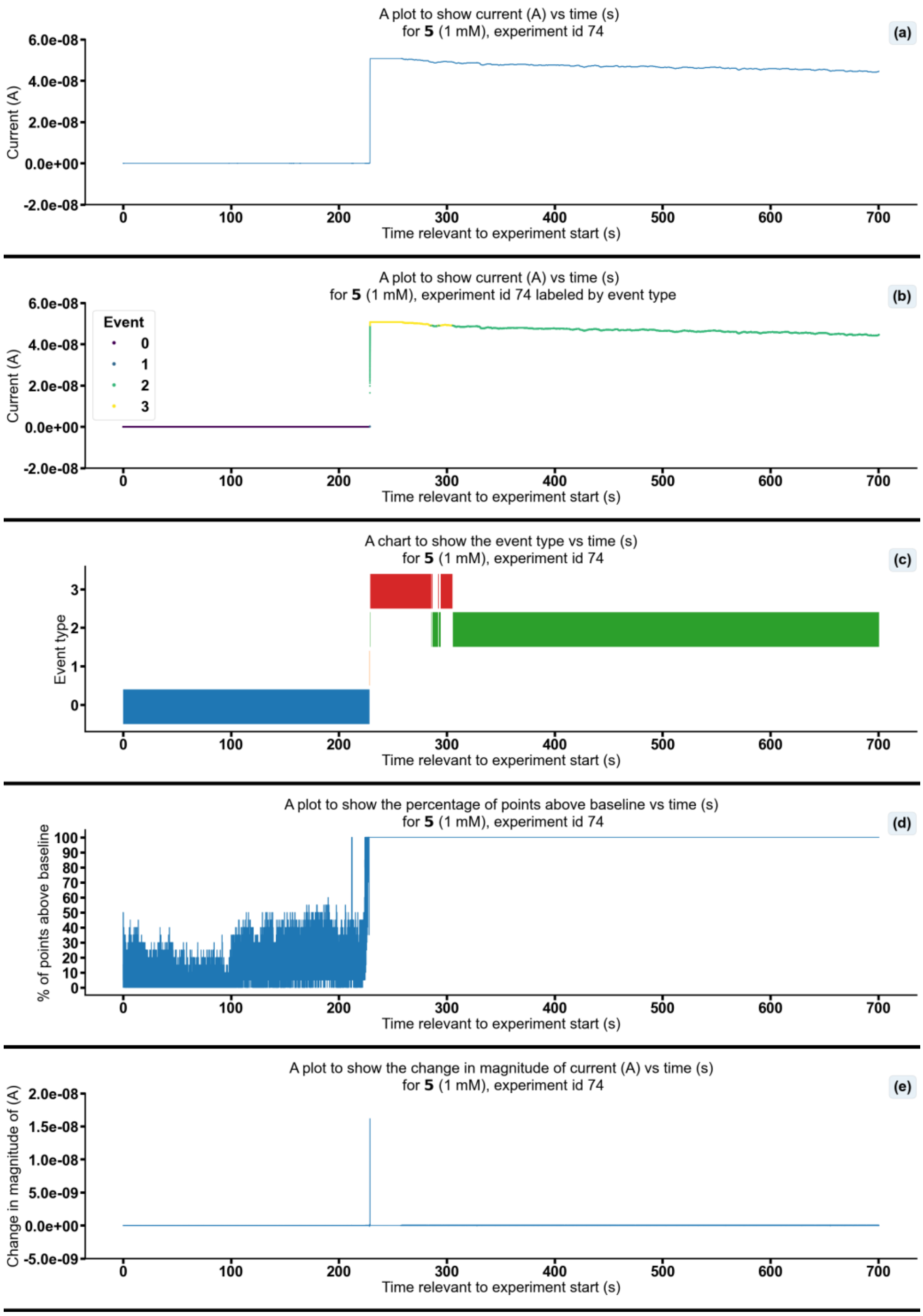

Figure S246 - Summarising the data from the patch clamp experiment for **5** (1 mM), experiment id 74 at +50 mV. Events were categorised into the following types: 0: -∞ A ≤ Event 0 < 2.50e-11 A, 1: 2.50e-11 A ≤ Event 1 < 2.50e-10 A, 2: 2.50e-10 A ≤ Event 2 < 4.90e-08 A, 3: 4.90e-08 A ≤ Event 3 < ∞ A. Subfigure (d) shows the percentage of points found above the threshold -5.0e-11 (A) in a rolling look ahead window of 20 points. The threshold is the mean + standard deviation of the noise of the first 200 datapoints.

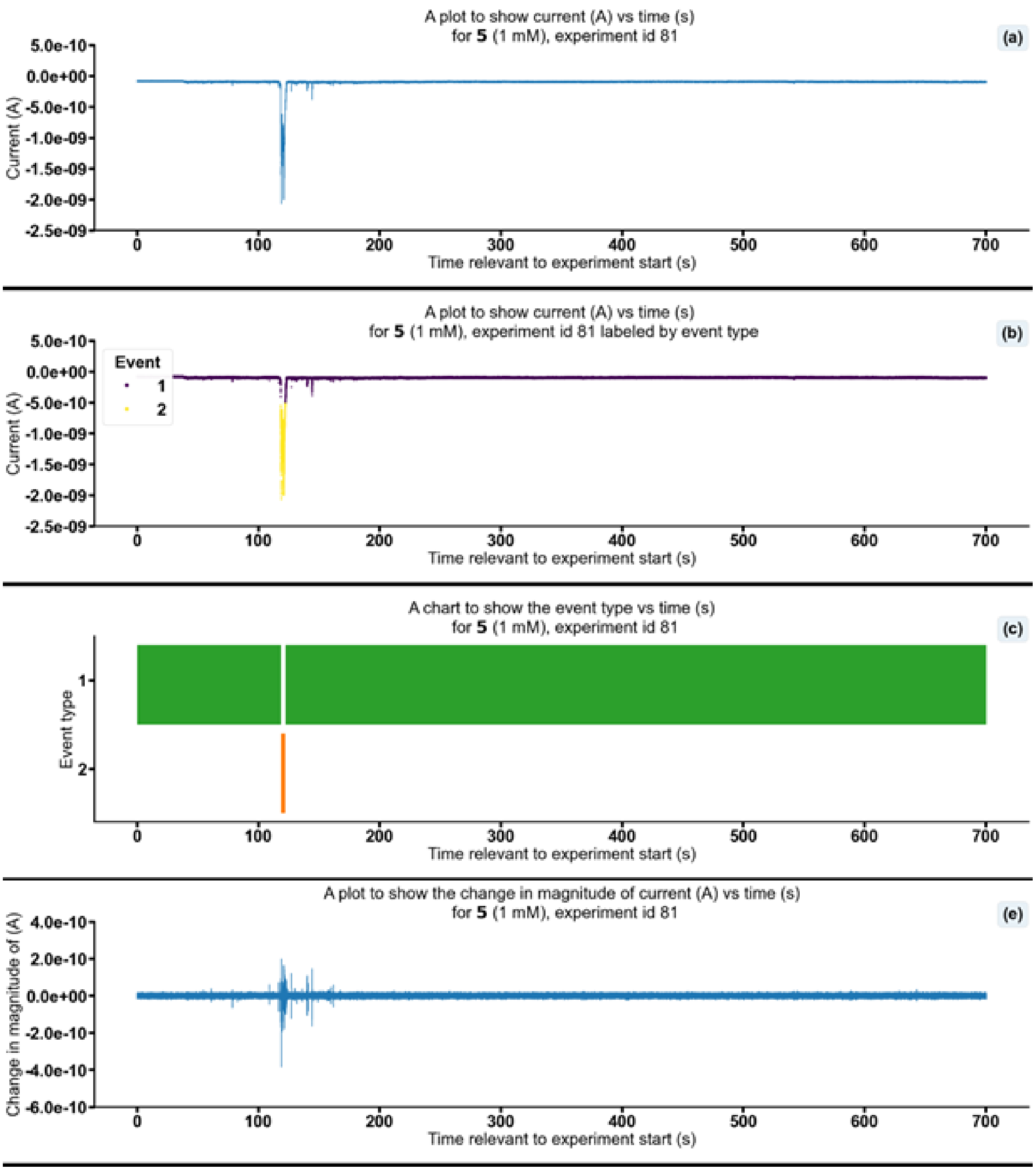

Figure S247 - Summarising the data from the patch clamp experiment for **5** (1 mM), experiment id 81 at -100 mV. Events were categorised into the following types: 0: -5.00e-11 A ≤ Event 0 < ∞ A, 1: -5.00e-10 A ≤ Event 1 < -5.00e-11 A, 2: -4.90e-08 A ≤ Event 2 < -5.00e-10 A, 3: -∞ A ≤ Event 3 < -4.90e-08 A. There is no Event 0 as the current of the baseline noise is less than the lower bound of Event 0.

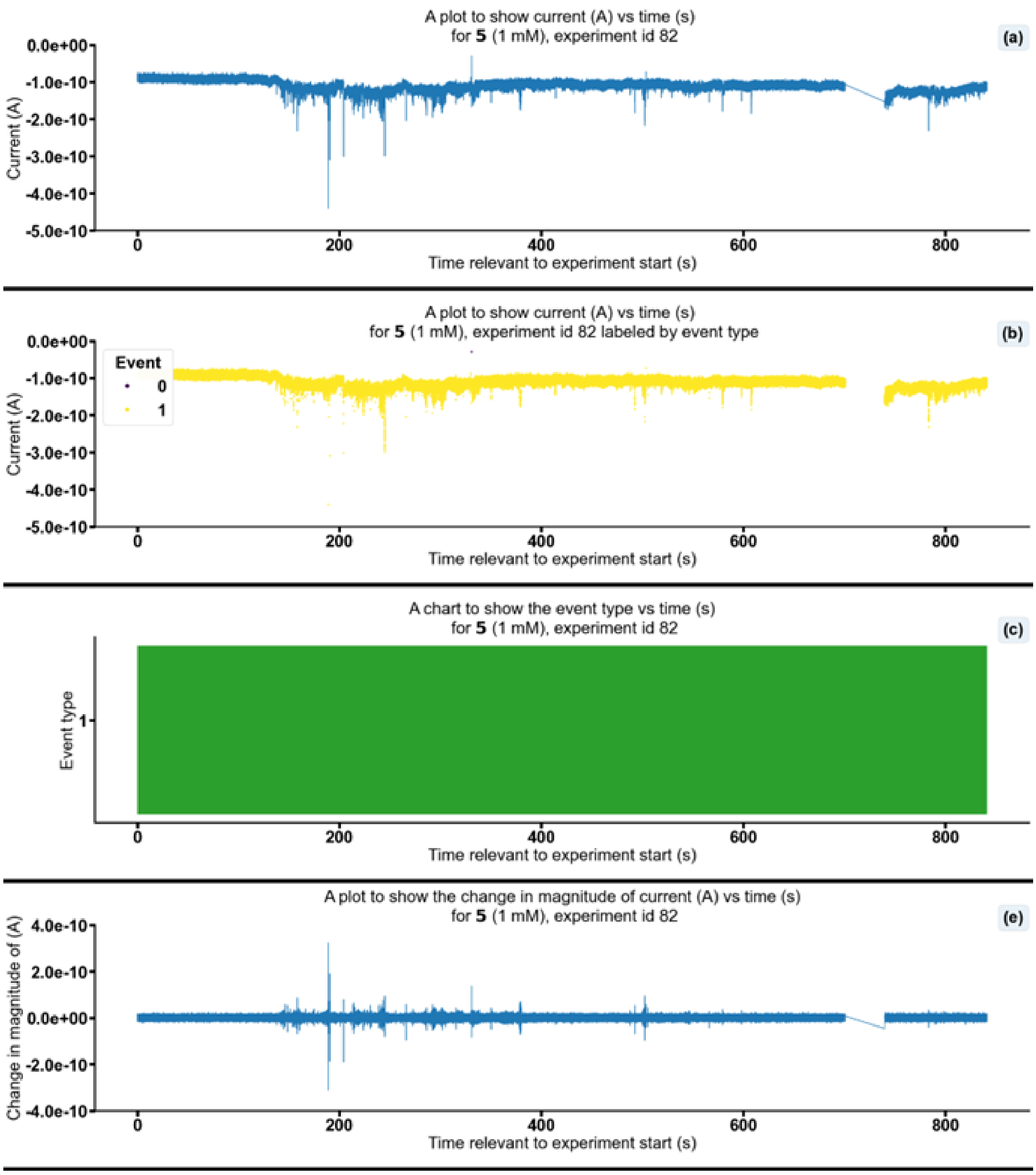

Figure S248 - Summarising the data from the patch clamp experiment for **5** (1 mM), experiment id 82 at -100 mV. Events were categorised into the following types: 0: -5.00e-11 A ≤ Event 0 < ∞ A, 1: -5.00e-10 A ≤ Event 1 < -5.00e-11 A, 2: -4.90e-08 A ≤ Event 2 < -5.00e-10 A, 3: -∞ A ≤ Event 3 < -4.90e-08 A.

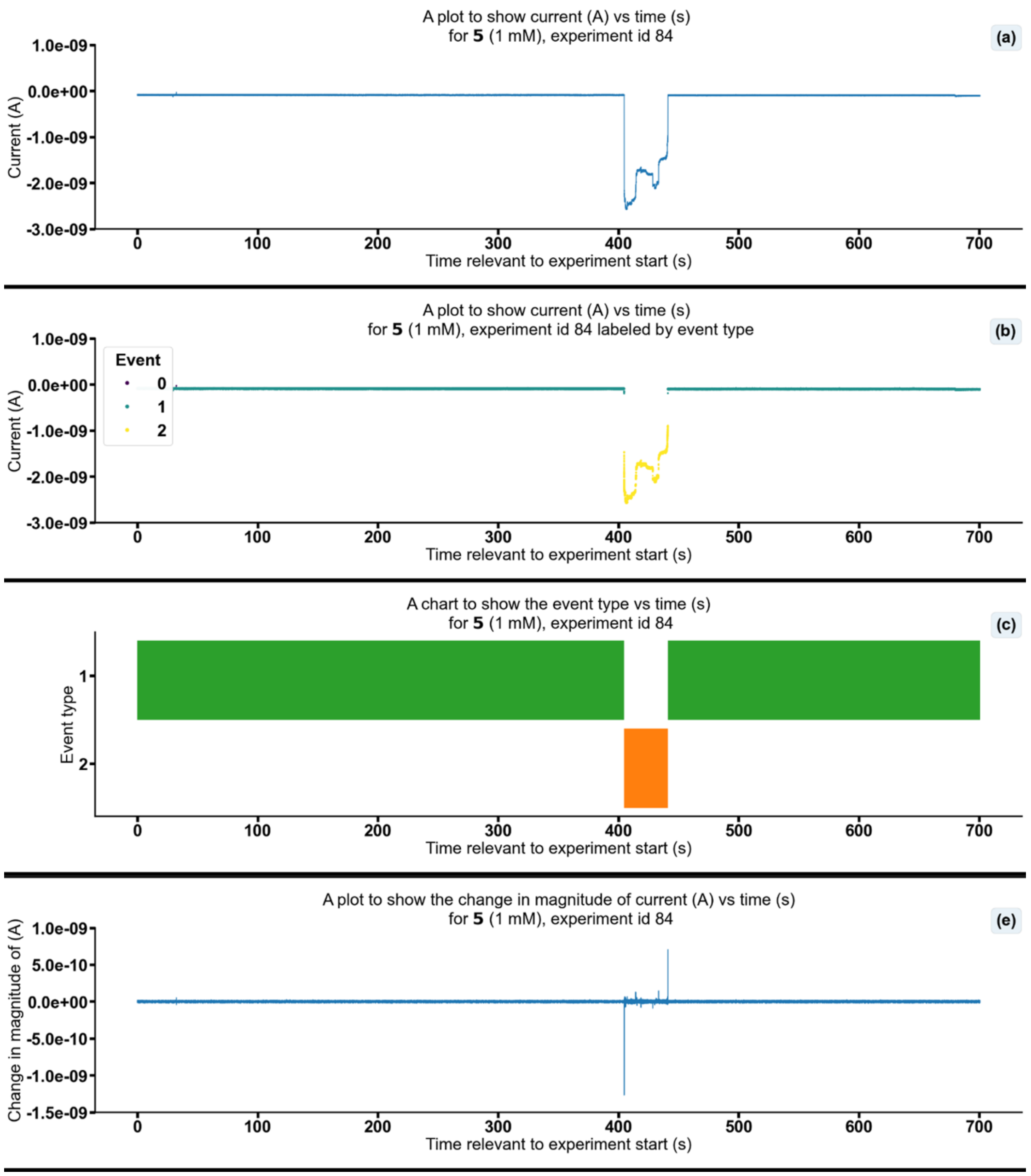

Figure S249 - Summarising the data from the patch clamp experiment for **5** (1 mM), experiment id 84 at -100 mV. Events were categorised into the following types: 0: -5.00e-11 A ≤ Event 0 < ∞ A, 1: -5.00e-10 A ≤ Event 1 < -5.00e-11 A, 2: -4.90e-08 A ≤ Event 2 < -5.00e-10 A, 3: -∞ A ≤ Event 3 < -4.90e-08 A.

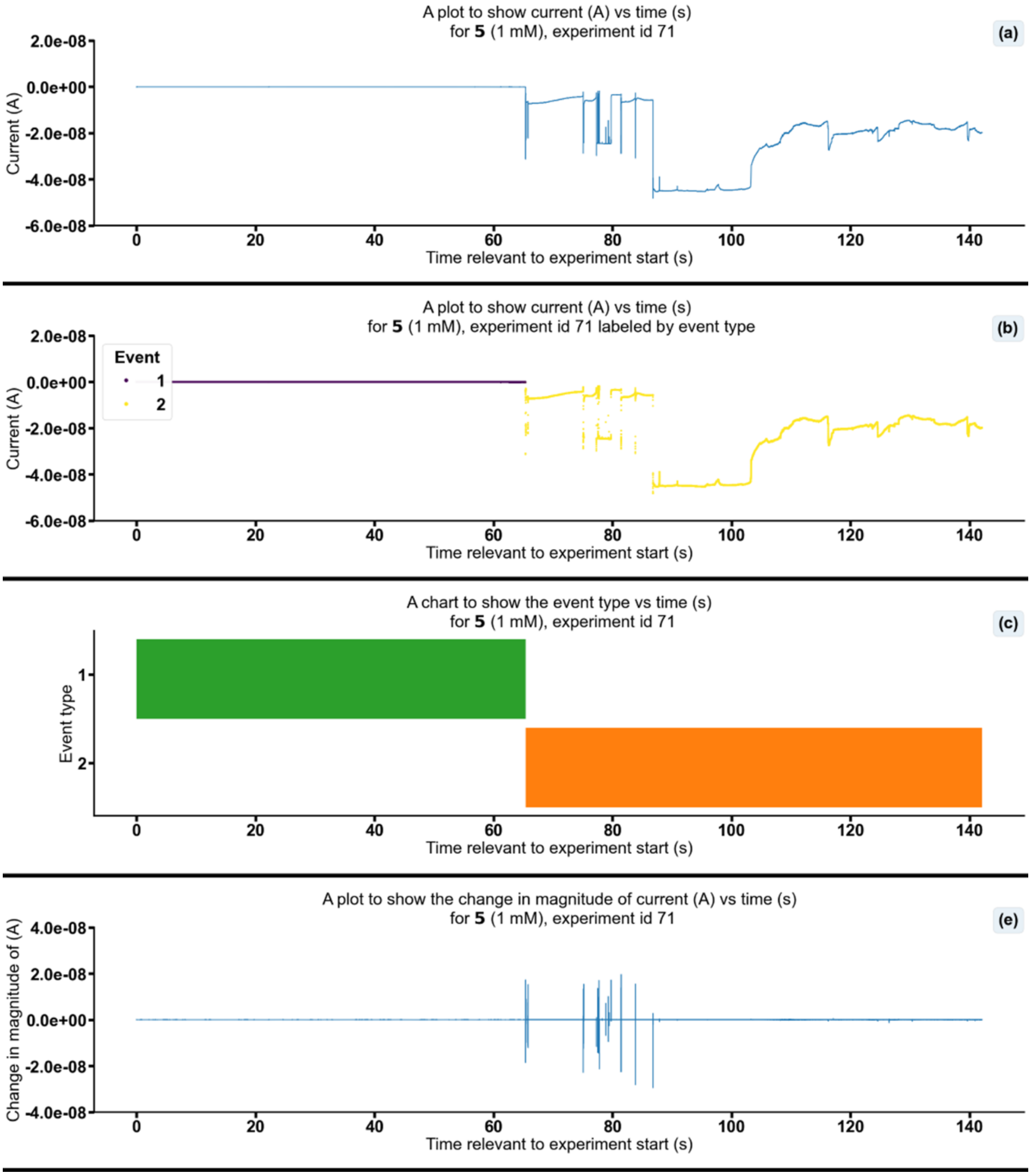

Figure S250 - Summarising the data from the patch clamp experiment for **5** (1 mM), experiment id 71 at -50 mV. Events were categorised into the following types: 0: -2.50e-11 A ≤ Event 0 < ∞ A, 1: -2.50e-10 A ≤ Event 1 < -2.50e-11 A, 2: -4.90e-08 A ≤ Event 2 < -2.50e-10 A, 3: -∞ A ≤ Event 3 < -4.90e-08 A. There is no Event 0 as the current of the baseline noise is less than the lower bound of Event 0.

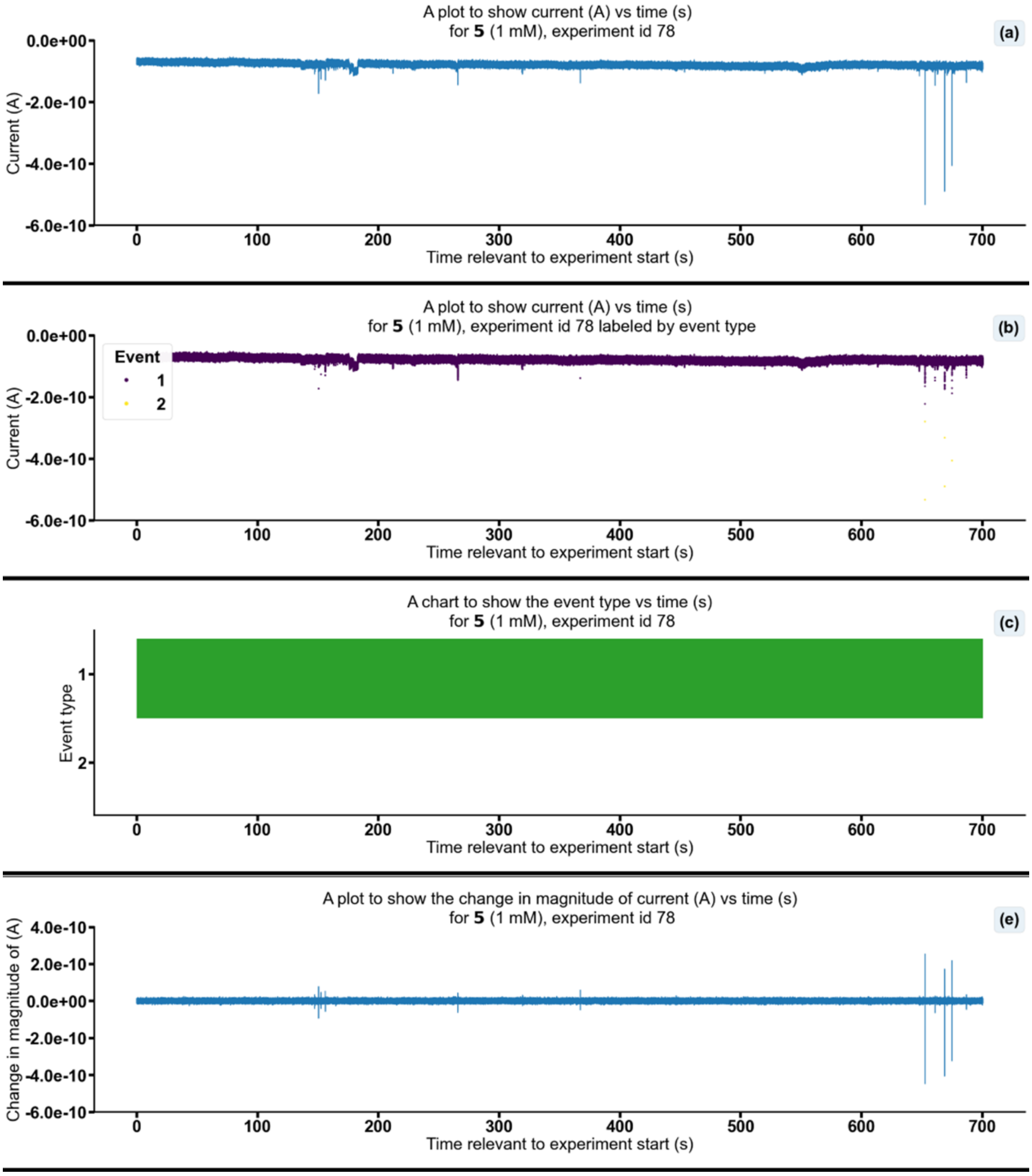

Figure S251 - Summarising the data from the patch clamp experiment for **5** (1 mM), experiment id 78 at -50 mV. Events were categorised into the following types: 0: -2.50e-11 A ≤ Event 0 < ∞ A, 1: -2.50e-10 A ≤ Event 1 < -2.50e-11 A, 2: -4.90e-08 A ≤ Event 2 < -2.50e-10 A, 3: -∞ A ≤ Event 3 < -4.90e-08 A. There is no Event 0 as the current of the baseline noise is less than the lower bound of Event 0.

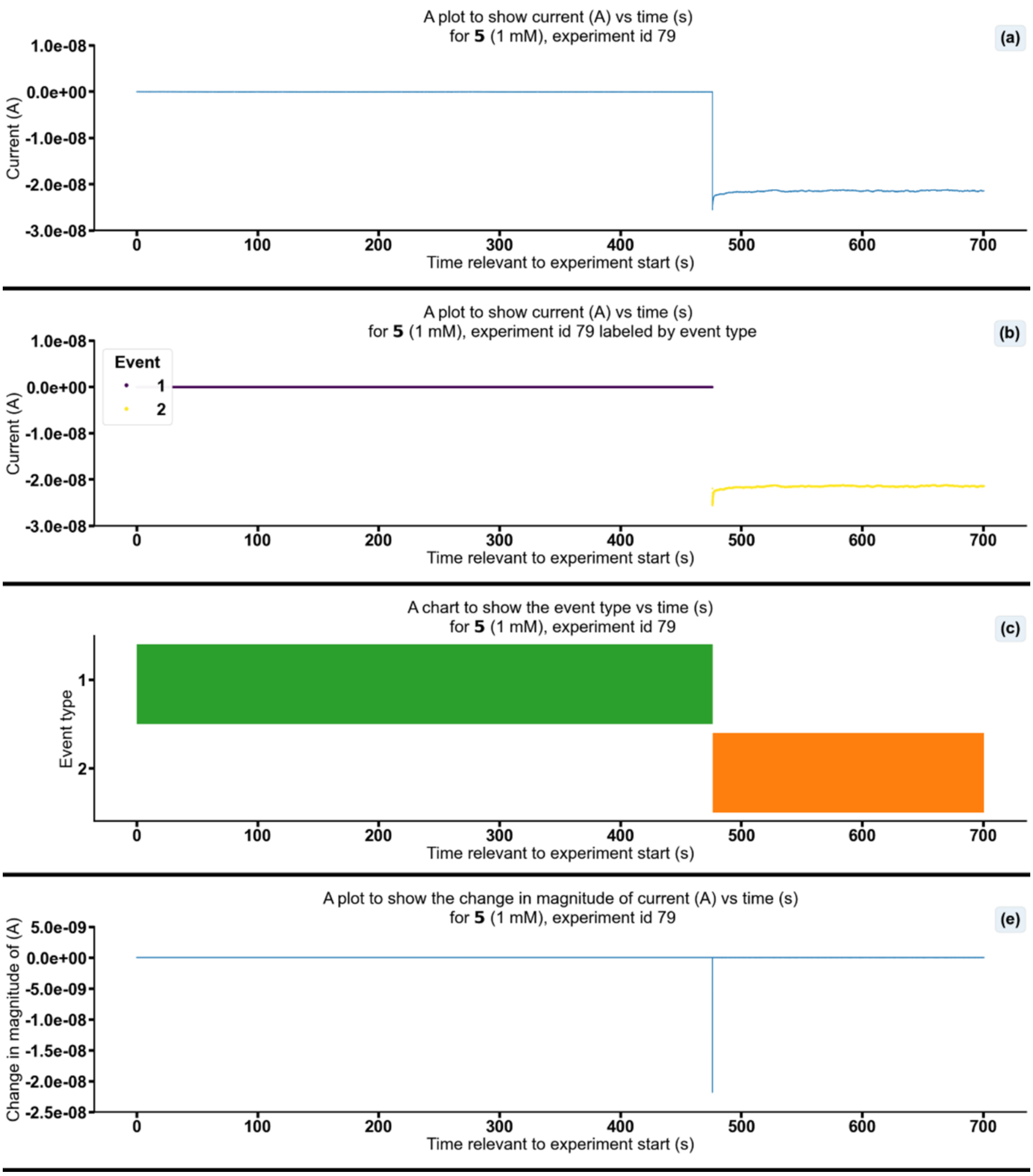

Figure S252 - Summarising the data from the patch clamp experiment for **5** (1 mM), experiment id 79 at -50 mV. Events were categorised into the following types: 0: -2.50e-11 A ≤ Event 0 < ∞ A, 1: -2.50e-10 A ≤ Event 1 < -2.50e-11 A, 2: -4.90e-08 A ≤ Event 2 < -2.50e-10 A, 3: -∞ A ≤ Event 3 < -4.90e-08 A. There is no Event 0 as the current of the baseline noise is less than the lower bound of Event 0.

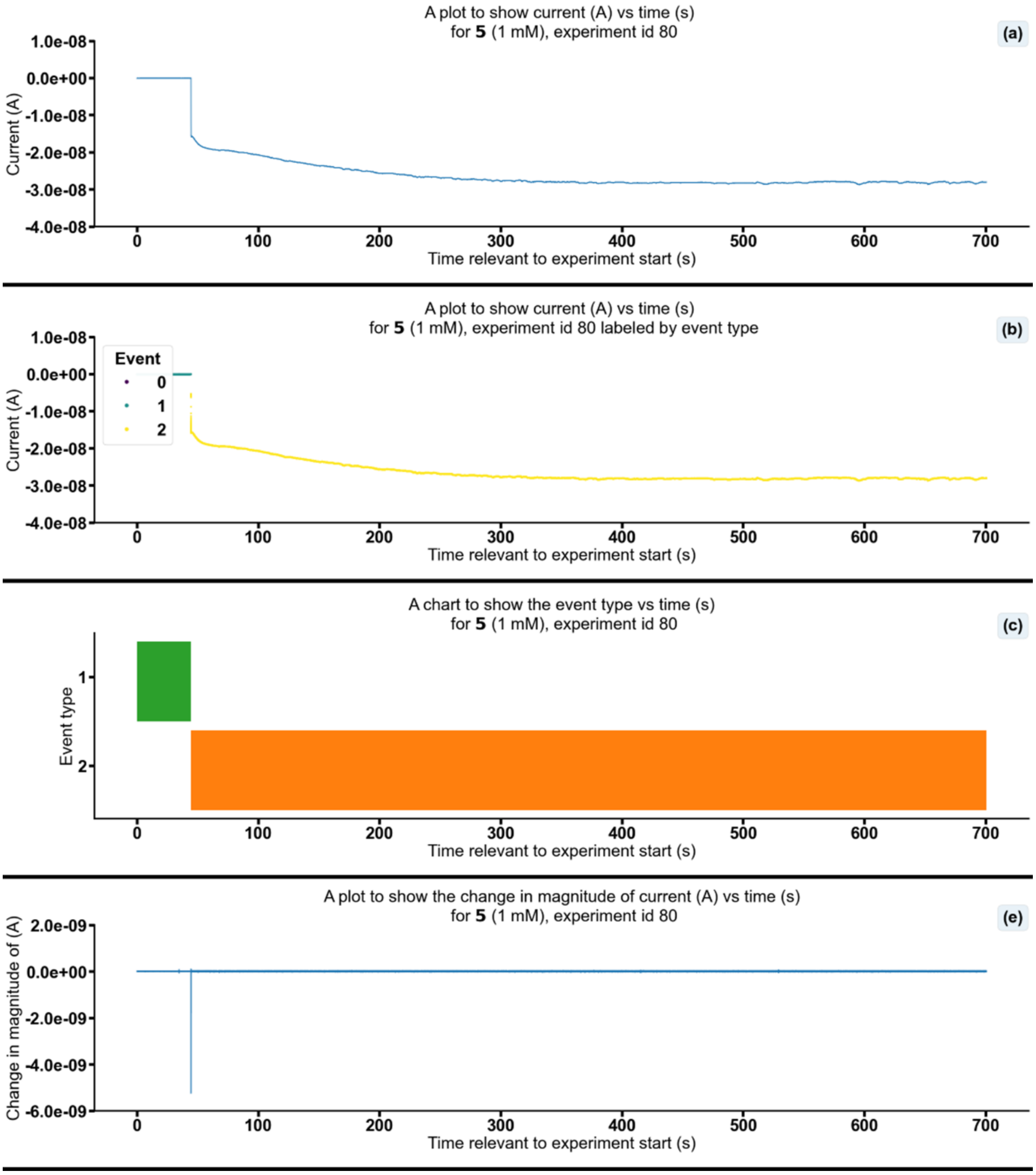

Figure S253 - Summarising the data from the patch clamp experiment for **5** (1 mM), experiment id 80 at -50 mV. Events were categorised into the following types: 0: -2.50e-11 A ≤ Event 0 < ∞ A, 1: -2.50e-10 A ≤ Event 1 < -2.50e-11 A, 2: -4.90e-08 A ≤ Event 2 < -2.50e-10 A, 3: -∞ A ≤ Event 3 < -4.90e-08 A.

Changing the concentration of **3** and **5** (1:1)

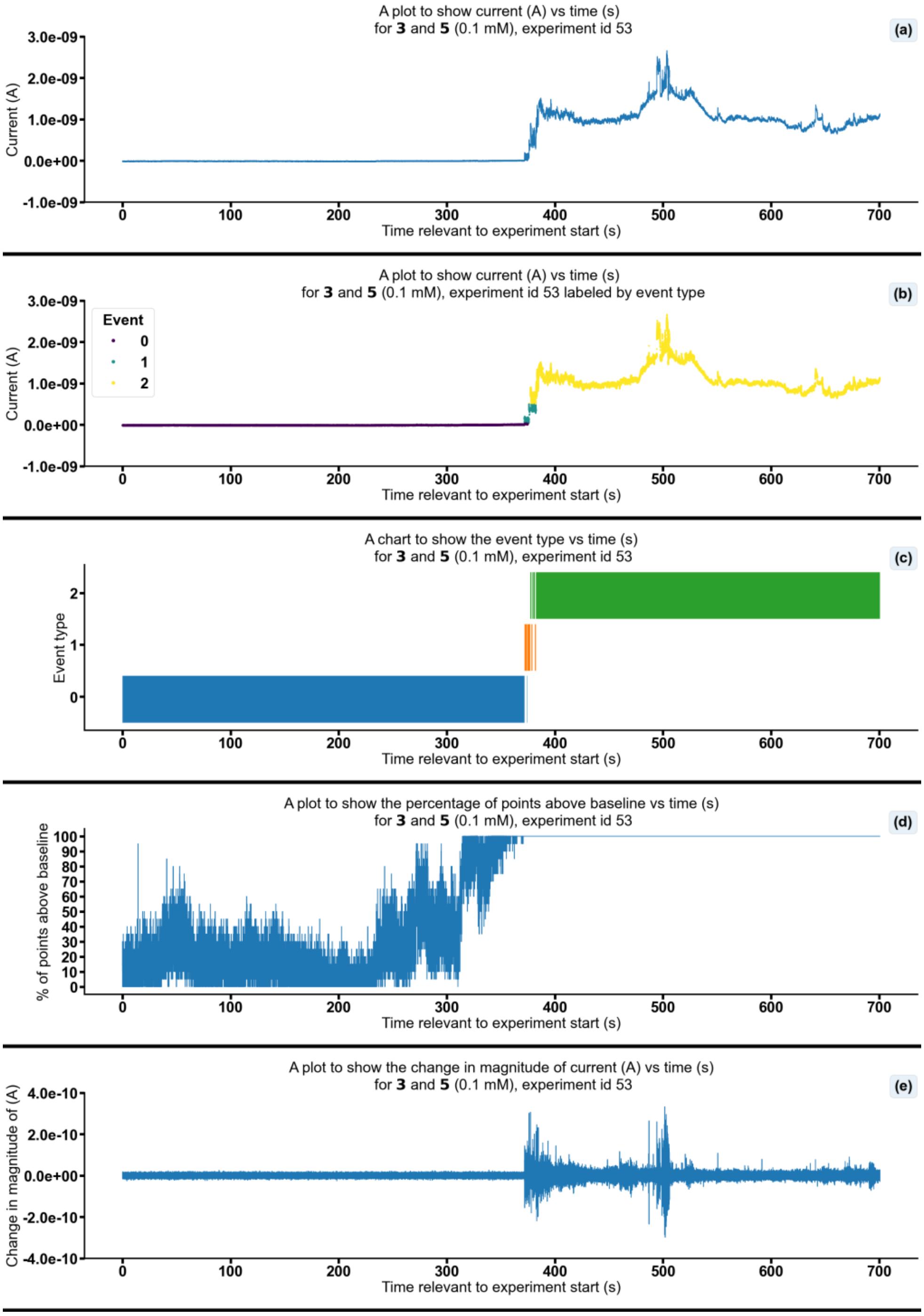

Figure S254 - Summarising the data from the patch clamp experiment for **3** and **5** (0.1 mM), experiment id 53 at +100 mV. Events were categorised into the following types: 0: -∞ A ≤ Event 0 < 5.00e-11 A, 1: 5.00e-11 A ≤ Event 1 < 5.00e-10 A, 2: 5.00e-10 A ≤ Event 2 < 4.90e-08 A, 3: 4.90e-08 A ≤ Event 3 < ∞ A. Subfigure (d) shows the percentage of points found above the threshold -1.1e-11 (A) in a rolling look ahead window of 20 points. The threshold is the mean + standard deviation of the noise of the first 200 datapoints.

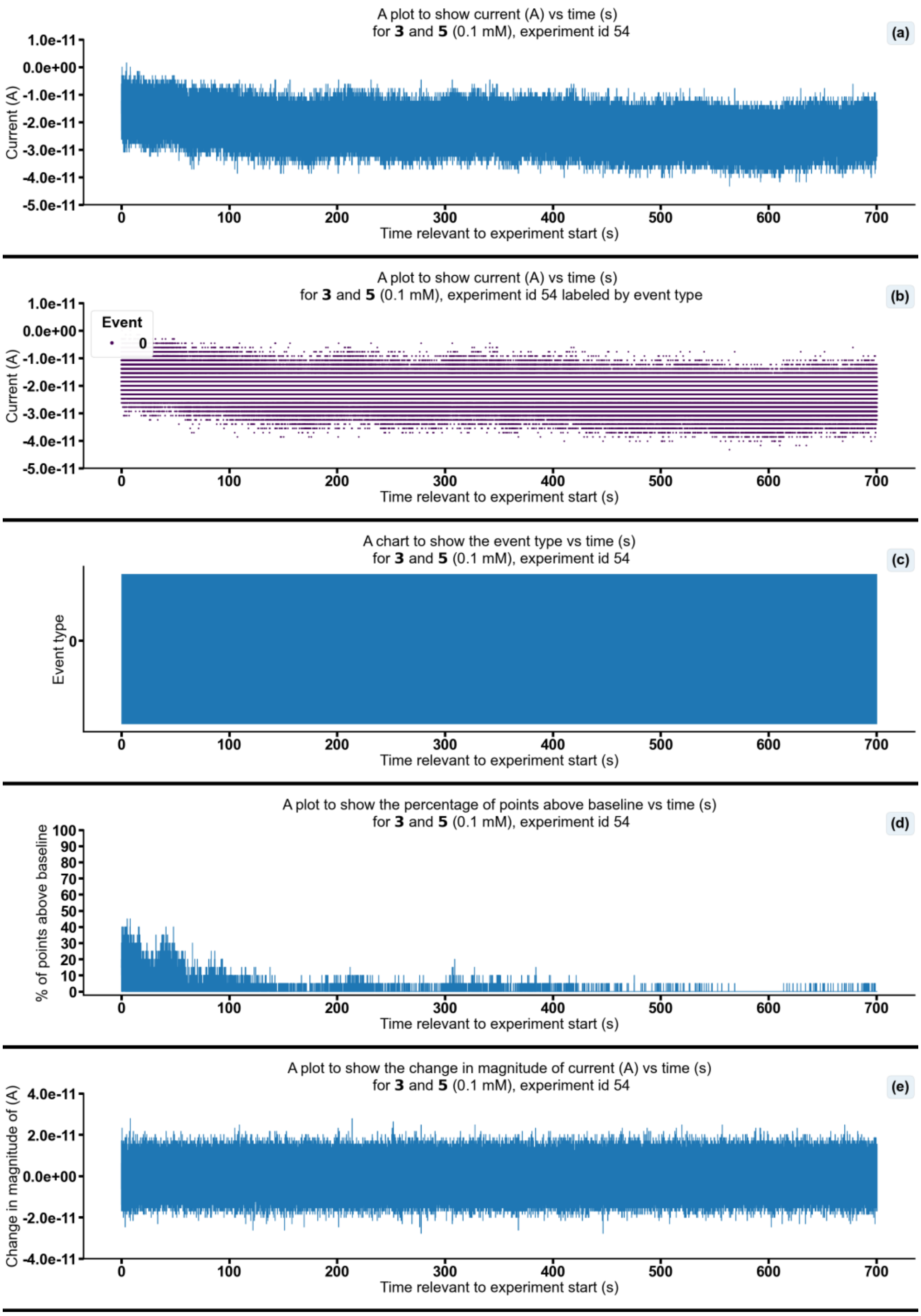

Figure S255 - Summarising the data from the patch clamp experiment for **3** and **5** (0.1 mM), experiment id 54 at +100 mV. Events were categorised into the following types: 0: -∞ A ≤ Event 0 < 5.00e-11 A, 1: 5.00e-11 A ≤ Event 1 < 5.00e-10 A, 2: 5.00e-10 A ≤ Event 2 < 4.90e-08 A, 3: 4.90e-08 A ≤ Event 3 < ∞ A. Subfigure (d) shows the percentage of points found above the threshold -1.2e-11 (A) in a rolling look ahead window of 20 points. The threshold is the mean + standard deviation of the noise of the first 200 datapoints.

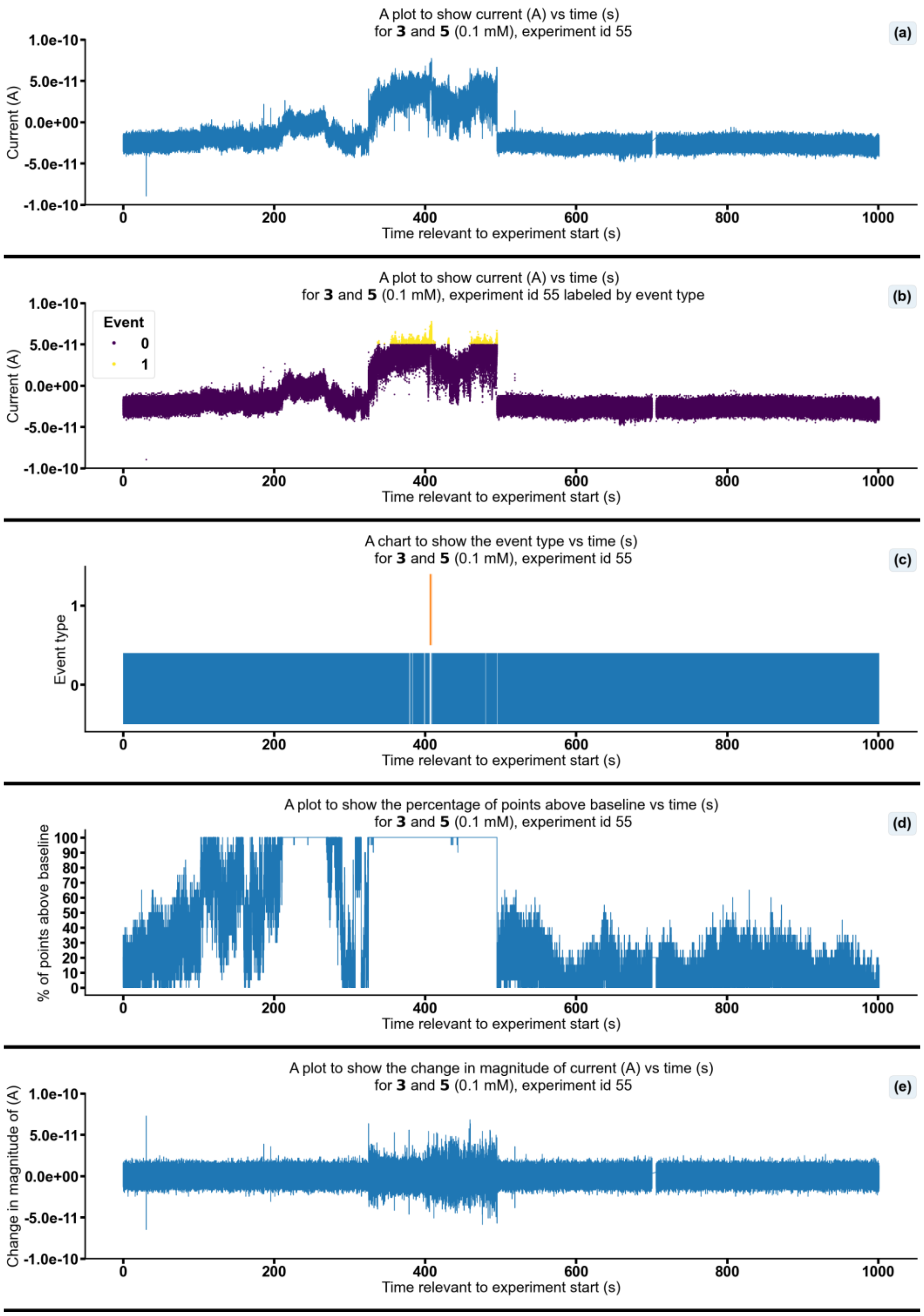

Figure S256 - Summarising the data from the patch clamp experiment for **3** and **5** (0.1 mM), experiment id 55 at +100 mV. Events were categorised into the following types: 0: -∞ A ≤ Event 0 < 5.00e-11 A, 1: 5.00e-11 A ≤ Event 1 < 5.00e-10 A, 2: 5.00e-10 A ≤ Event 2 < 4.90e-08 A, 3: 4.90e-08 A ≤ Event 3 < ∞ A. Subfigure (d) shows the percentage of points found above the threshold -2.1e-11 (A) in a rolling look ahead window of 20 points. The threshold is the mean + standard deviation of the noise of the first 200 datapoints.

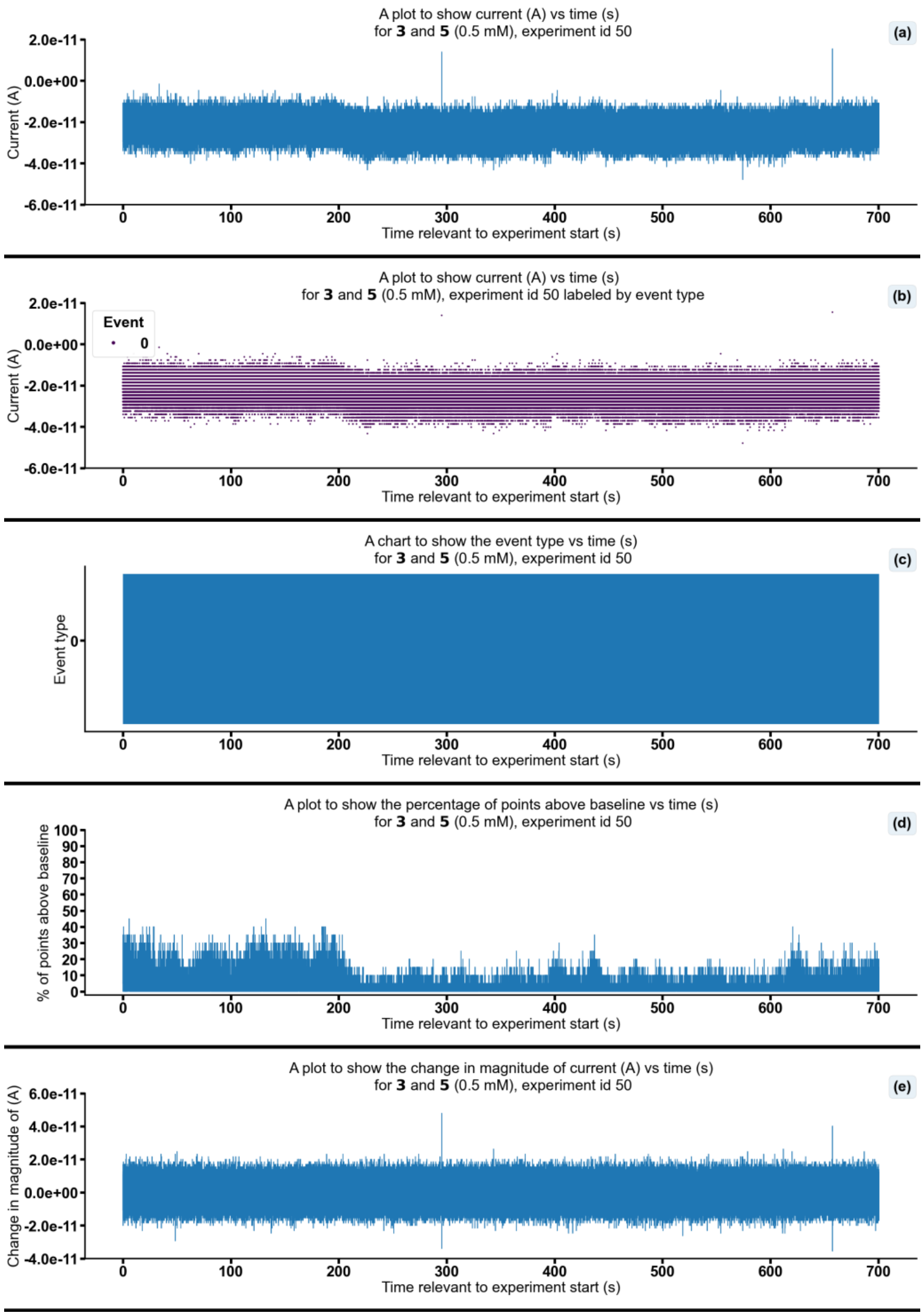

Figure S257 - Summarising the data from the patch clamp experiment for **3** and **5** (0.5 mM), experiment id 50 at +100 mV. Events were categorised into the following types: 0: -∞ A ≤ Event 0 < 5.00e-11 A, 1: 5.00e-11 A ≤ Event 1 < 5.00e-10 A, 2: 5.00e-10 A ≤ Event 2 < 4.90e-08 A, 3: 4.90e-08 A ≤ Event 3 < ∞ A. Subfigure (d) shows the percentage of points found above the threshold -1.6e-11 (A) in a rolling look ahead window of 20 points. The threshold is the mean + standard deviation of the noise of the first 200 datapoints.

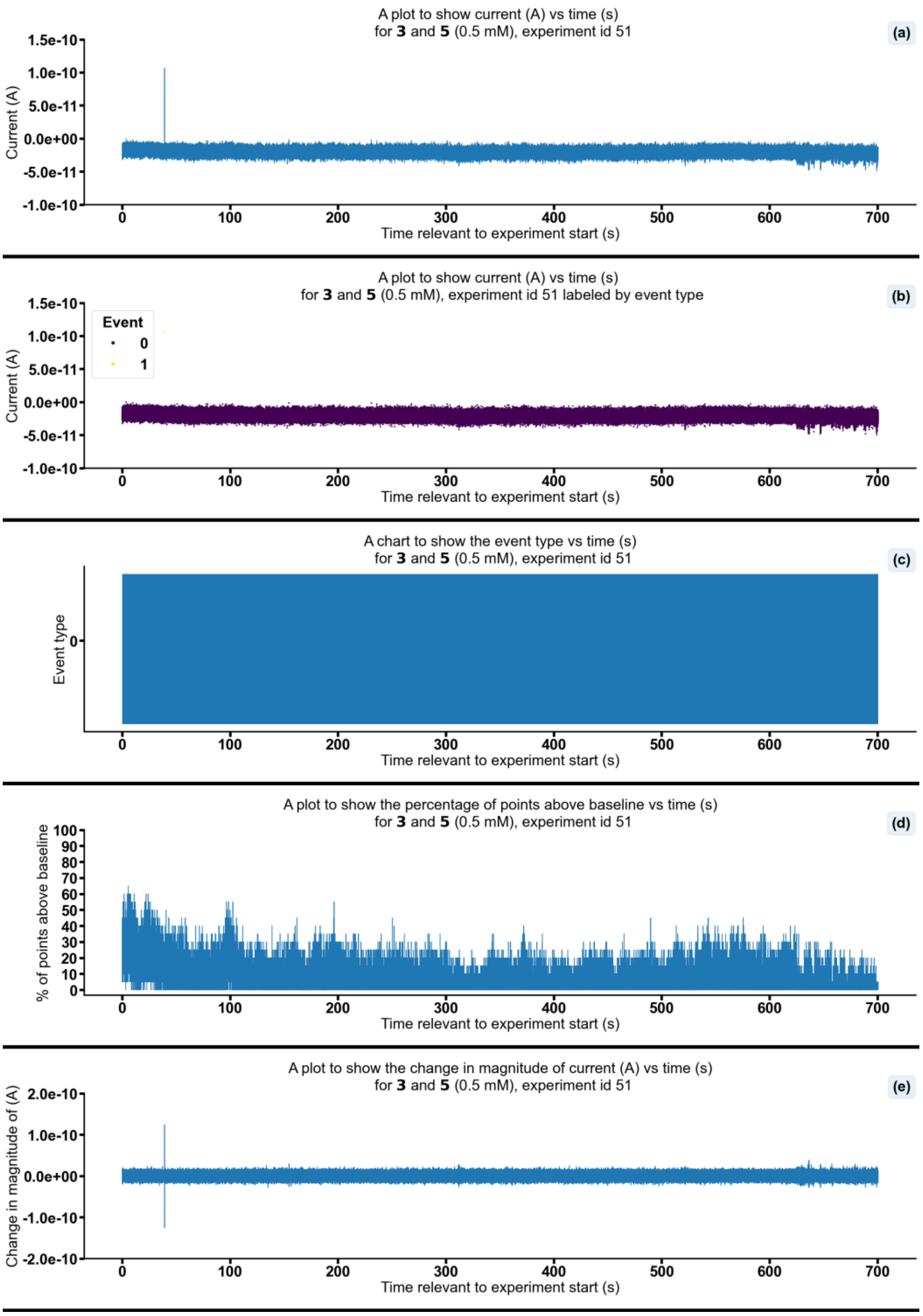

Figure S258 - Summarising the data from the patch clamp experiment for **3** and **5** (0.5 mM), experiment id 51 at +100 mV. Events were categorised into the following types: 0: -∞ A ≤ Event 0 < 5.00e-11 A, 1: 5.00e-11 A ≤ Event 1 < 5.00e-10 A, 2: 5.00e-10 A ≤ Event 2 < 4.90e-08 A, 3: 4.90e-08 A ≤ Event 3 < ∞ A. Subfigure (d) shows the percentage of points found above the threshold -1.4e-11 (A) in a rolling look ahead window of 20 points. The threshold is the mean + standard deviation of the noise of the first 200 datapoints.

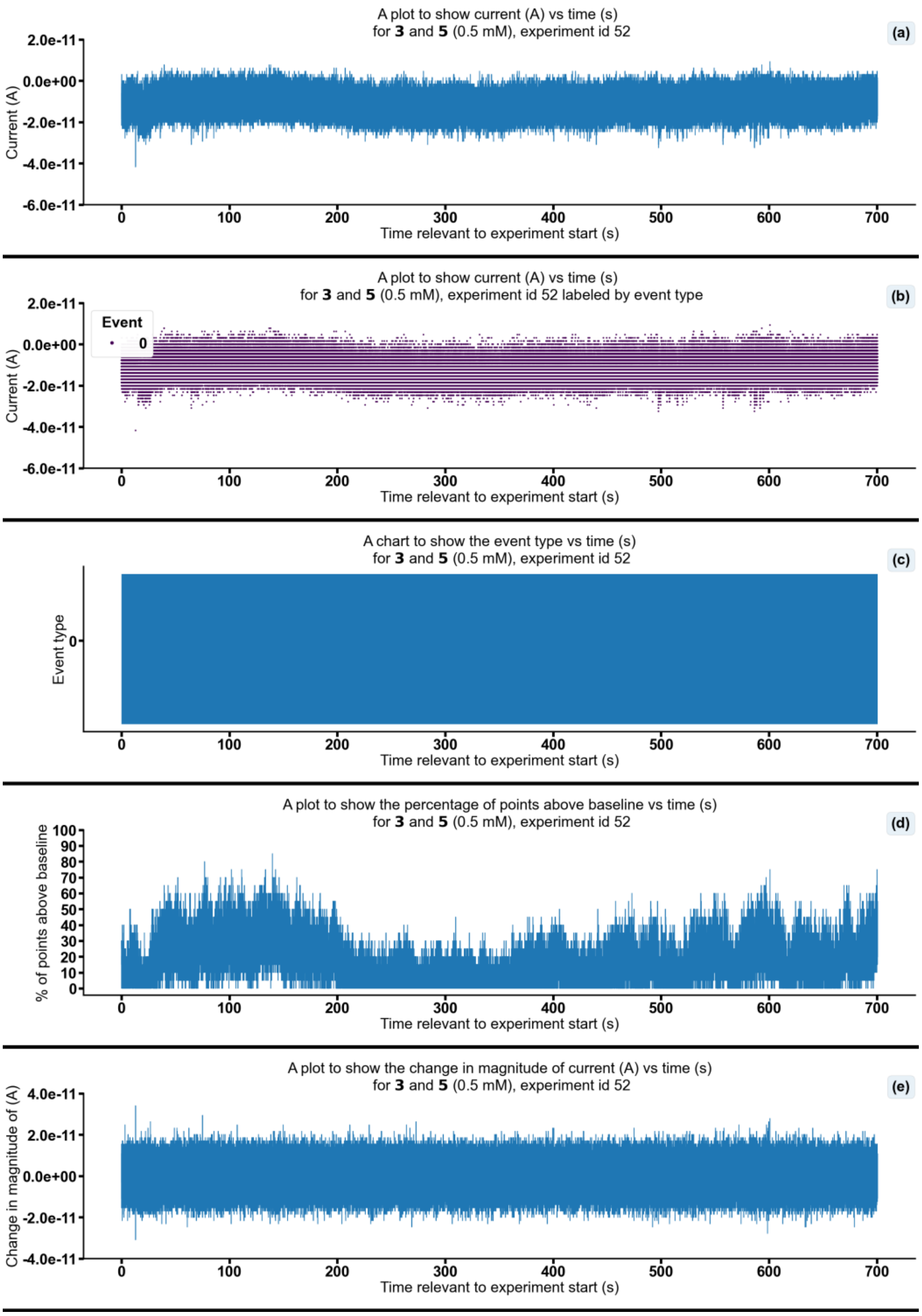

Figure S259 - Summarising the data from the patch clamp experiment for **3** and **5** (0.5 mM), experiment id 52 at +100 mV. Events were categorised into the following types: 0: -∞ A ≤ Event 0 < 5.00e-11 A, 1: 5.00e-11 A ≤ Event 1 < 5.00e-10 A, 2: 5.00e-10 A ≤ Event 2 < 4.90e-08 A, 3: 4.90e-08 A ≤ Event 3 < ∞ A. Subfigure (d) shows the percentage of points found above the threshold -7.6e-12 (A) in a rolling look ahead window of 20 points. The threshold is the mean + standard deviation of the noise of the first 200 datapoints.

Changing the concentration of **7**

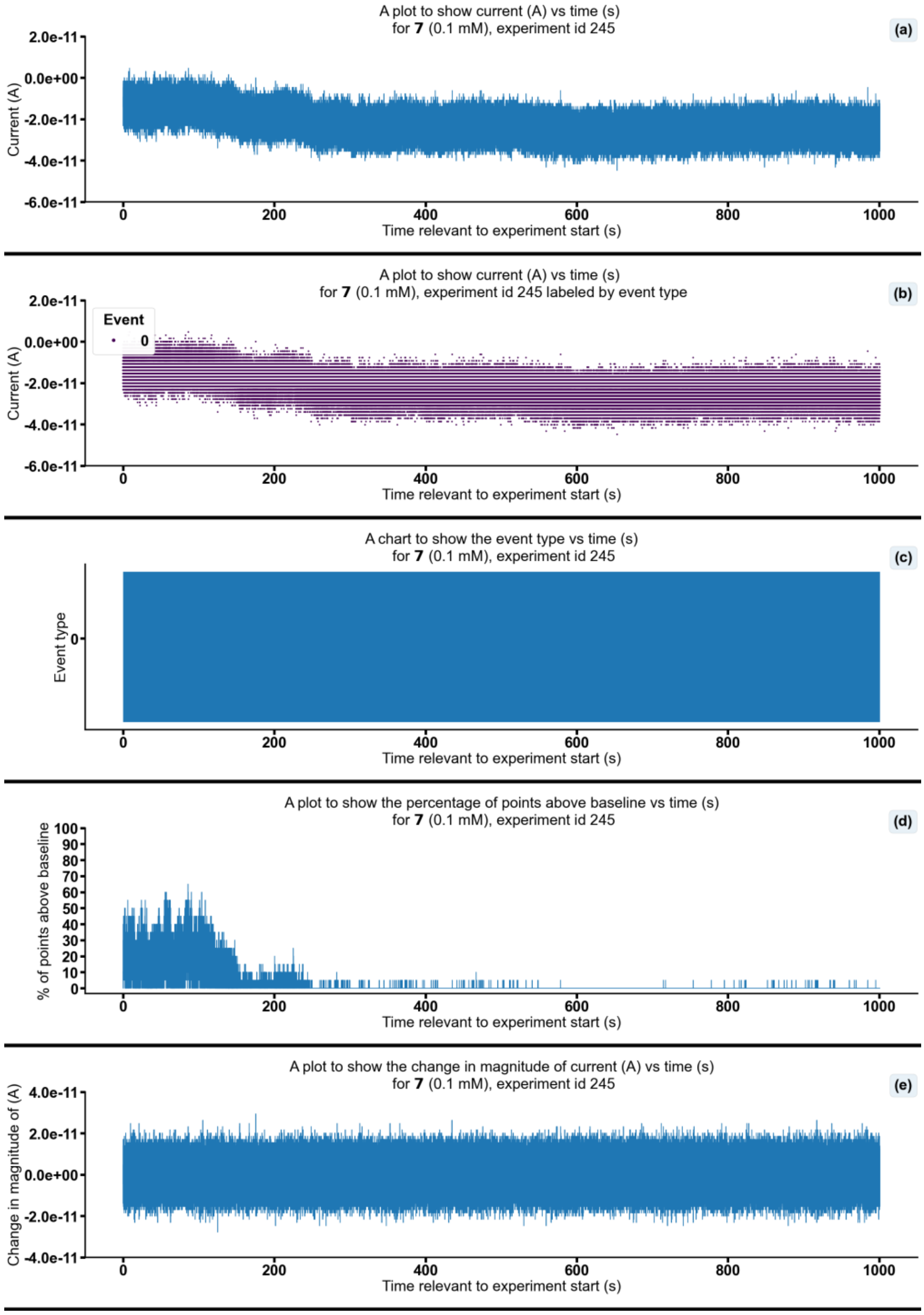

Figure S260 - Summarising the data from the patch clamp experiment for **7** (0.1 mM), experiment id 245 at +100 mV. Events were categorised into the following types: 0: -∞ A ≤ Event 0 < 5.00e-11 A, 1: 5.00e-11 A ≤ Event 1 < 5.00e-10 A, 2: 5.00e-10 A ≤ Event 2 < 4.90e-08 A, 3: 4.90e-08 A ≤ Event 3 < ∞ A. Subfigure (d) shows the percentage of points found above the threshold -9.6e-12 (A) in a rolling look ahead window of 20 points. The threshold is the mean + standard deviation of the noise of the first 200 datapoints.

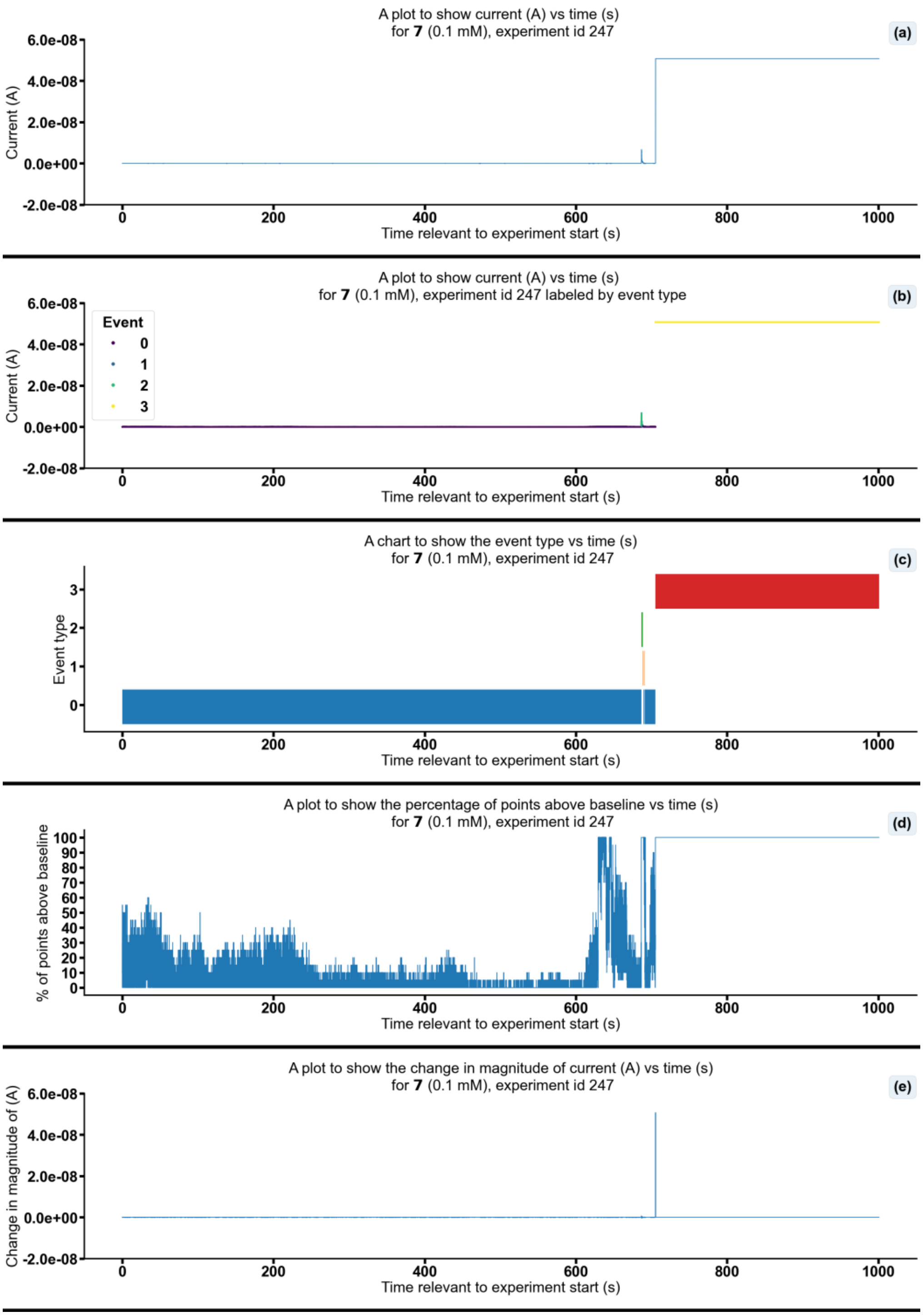

Figure S261 - Summarising the data from the patch clamp experiment for **7** (0.1 mM), experiment id 247 at +100 mV. Events were categorised into the following types: 0: -∞ A ≤ Event 0 < 5.00e-11 A, 1: 5.00e-11 A ≤ Event 1 < 5.00e-10 A, 2: 5.00e-10 A ≤ Event 2 < 4.90e-08 A, 3: 4.90e-08 A ≤ Event 3 < ∞ A. Subfigure (d) shows the percentage of points found above the threshold -7.0e-12 (A) in a rolling look ahead window of 20 points. The threshold is the mean + standard deviation of the noise of the first 200 datapoints.

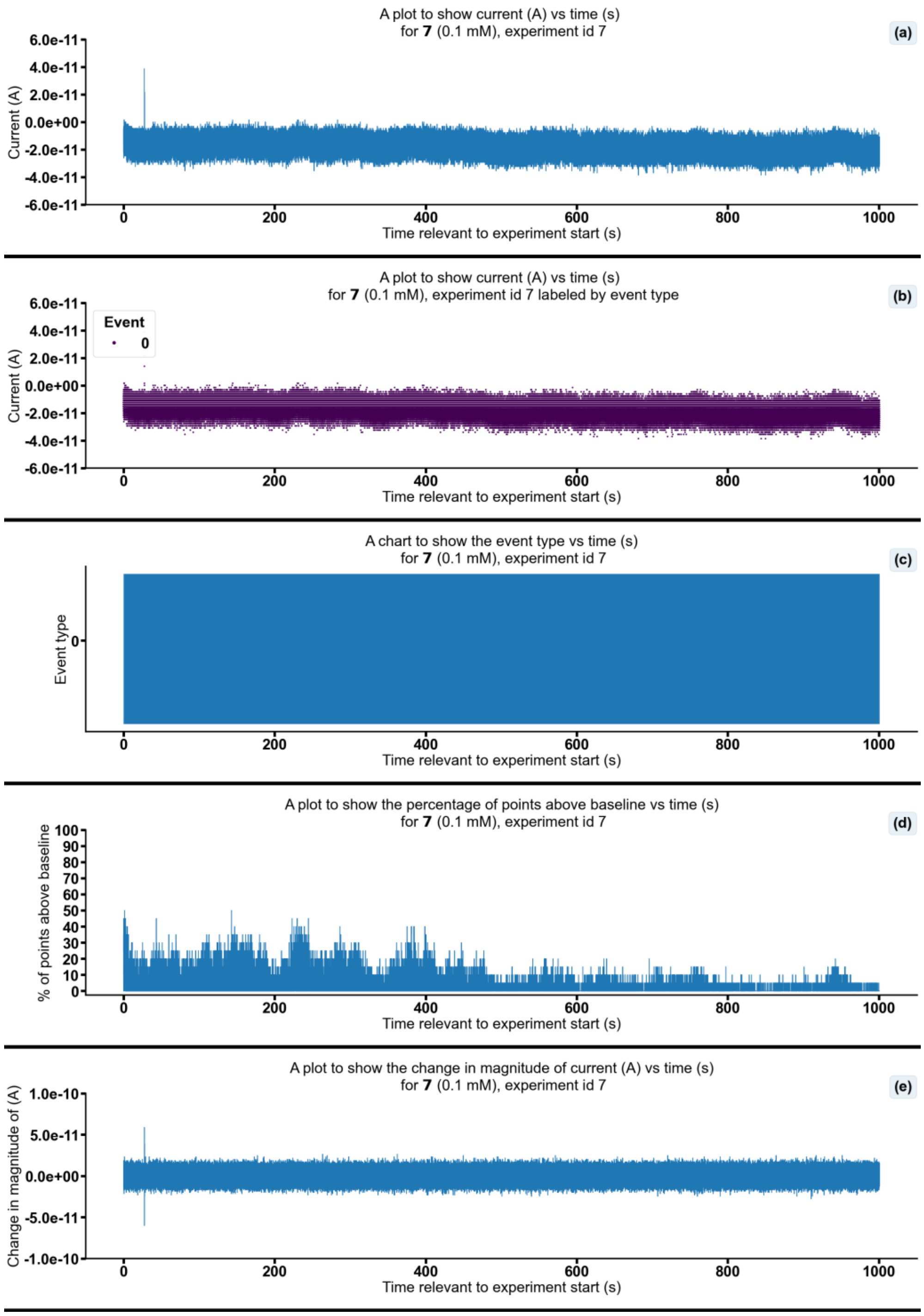

Figure S262 - Summarising the data from the patch clamp experiment for **7** (0.1 mM), experiment id 7 at +100 mV. Events were categorised into the following types: 0: -∞ A ≤ Event 0 < 5.00e-11 A, 1: 5.00e-11 A ≤ Event 1 < 5.00e-10 A, 2: 5.00e-10 A ≤ Event 2 < 4.90e-08 A, 3: 4.90e-08 A ≤ Event 3 < ∞ A. Subfigure (d) shows the percentage of points found above the threshold -9.9e-12 (A) in a rolling look ahead window of 20 points. The threshold is the mean + standard deviation of the noise of the first 200 datapoints.

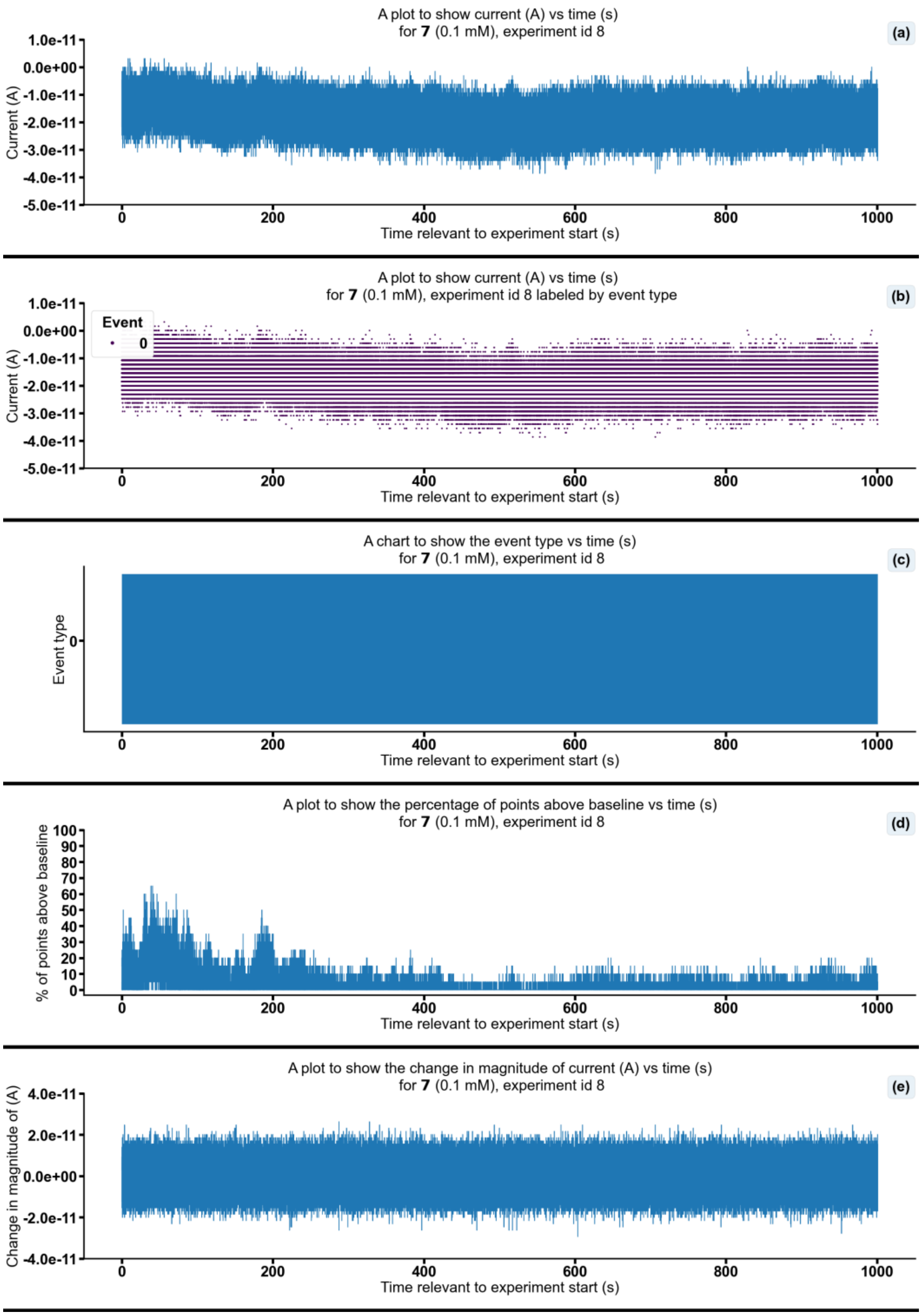

Figure S263 - Summarising the data from the patch clamp experiment for **7** (0.1 mM), experiment id 8 at +100 mV. Events were categorised into the following types: 0: -∞ A ≤ Event 0 < 5.00e-11 A, 1: 5.00e-11 A ≤ Event 1 < 5.00e-10 A, 2: 5.00e-10 A ≤ Event 2 < 4.90e-08 A, 3: 4.90e-08 A ≤ Event 3 < ∞ A. Subfigure (d) shows the percentage of points found above the threshold -1.1e-11 (A) in a rolling look ahead window of 20 points. The threshold is the mean + standard deviation of the noise of the first 200 datapoints.

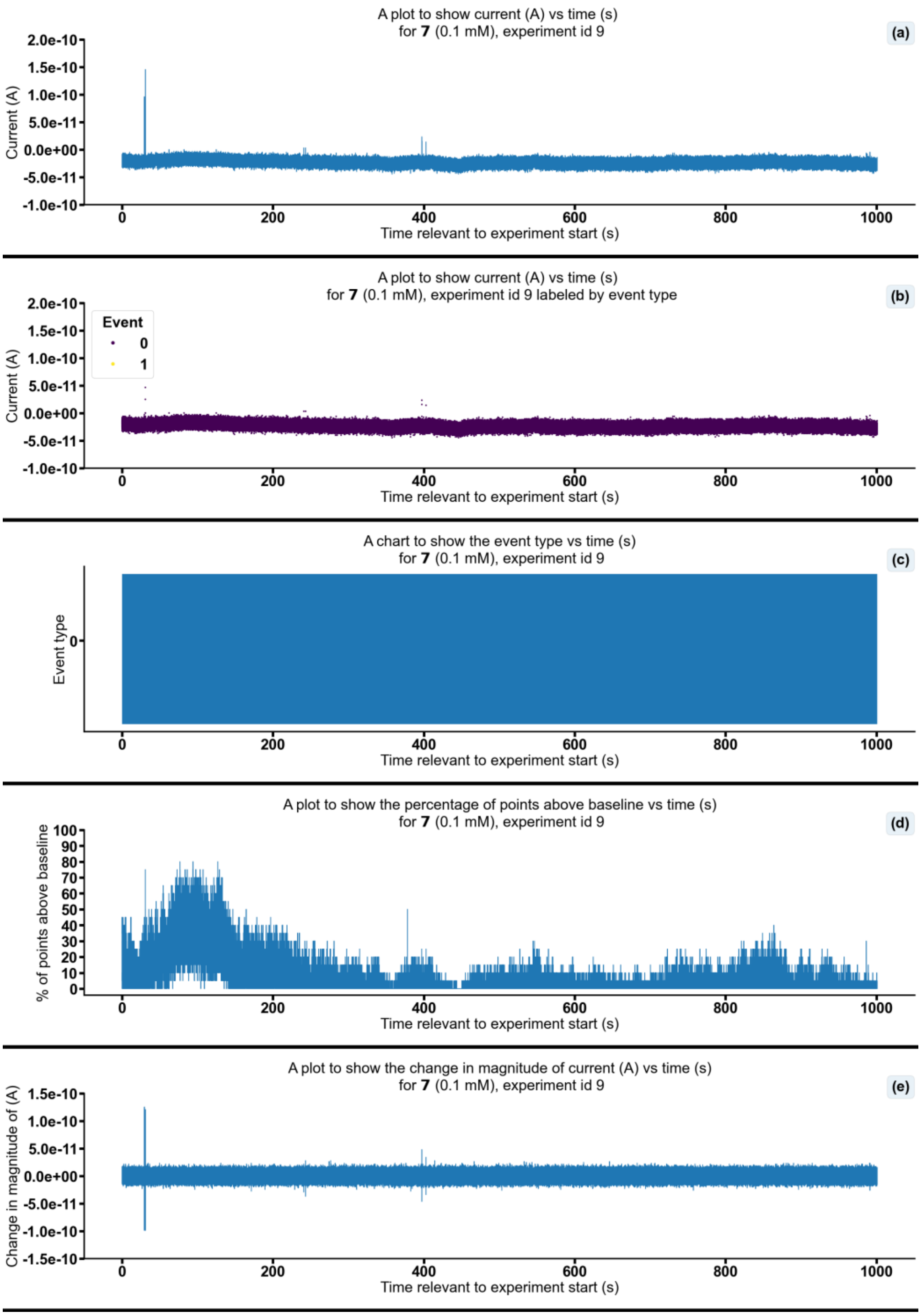

Figure S264- Summarising the data from the patch clamp experiment for **7** (0.1 mM), experiment id 9 at +100 mV. Events were categorised into the following types: 0: -∞ A ≤ Event 0 < 5.00e-11 A, 1: 5.00e-11 A ≤ Event 1 < 5.00e-10 A, 2: 5.00e-10 A ≤ Event 2 < 4.90e-08 A, 3: 4.90e-08 A ≤ Event 3 < ∞ A. Subfigure (d) shows the percentage of points found above the threshold -1.6e-11 (A) in a rolling look ahead window of 20 points. The threshold is the mean + standard deviation of the noise of the first 200 datapoints.

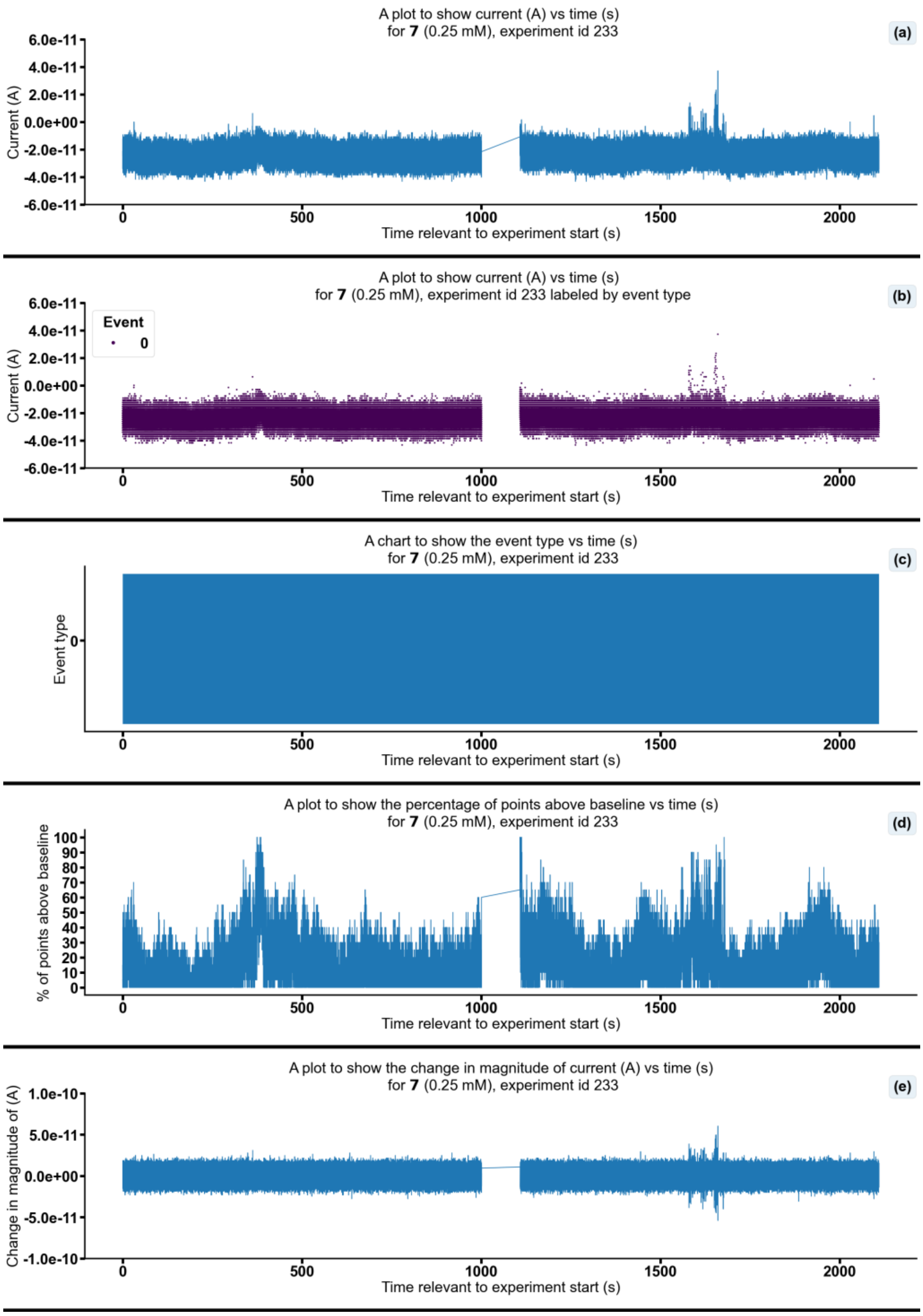

Figure S265 - Summarising the data from the patch clamp experiment for **7** (0.25 mM), experiment id 233 at +100 mV. Events were categorised into the following types: 0: -∞ A ≤ Event 0 < 5.00e-11 A, 1: 5.00e-11 A ≤ Event 1 < 5.00e-10 A, 2: 5.00e-10 A ≤ Event 2 < 4.90e-08 A, 3: 4.90e-08 A ≤ Event 3 < ∞ A. Subfigure (d) shows the percentage of points found above the threshold -2.0e-11 (A) in a rolling look ahead window of 20 points. The threshold is the mean + standard deviation of the noise of the first 200 datapoints.

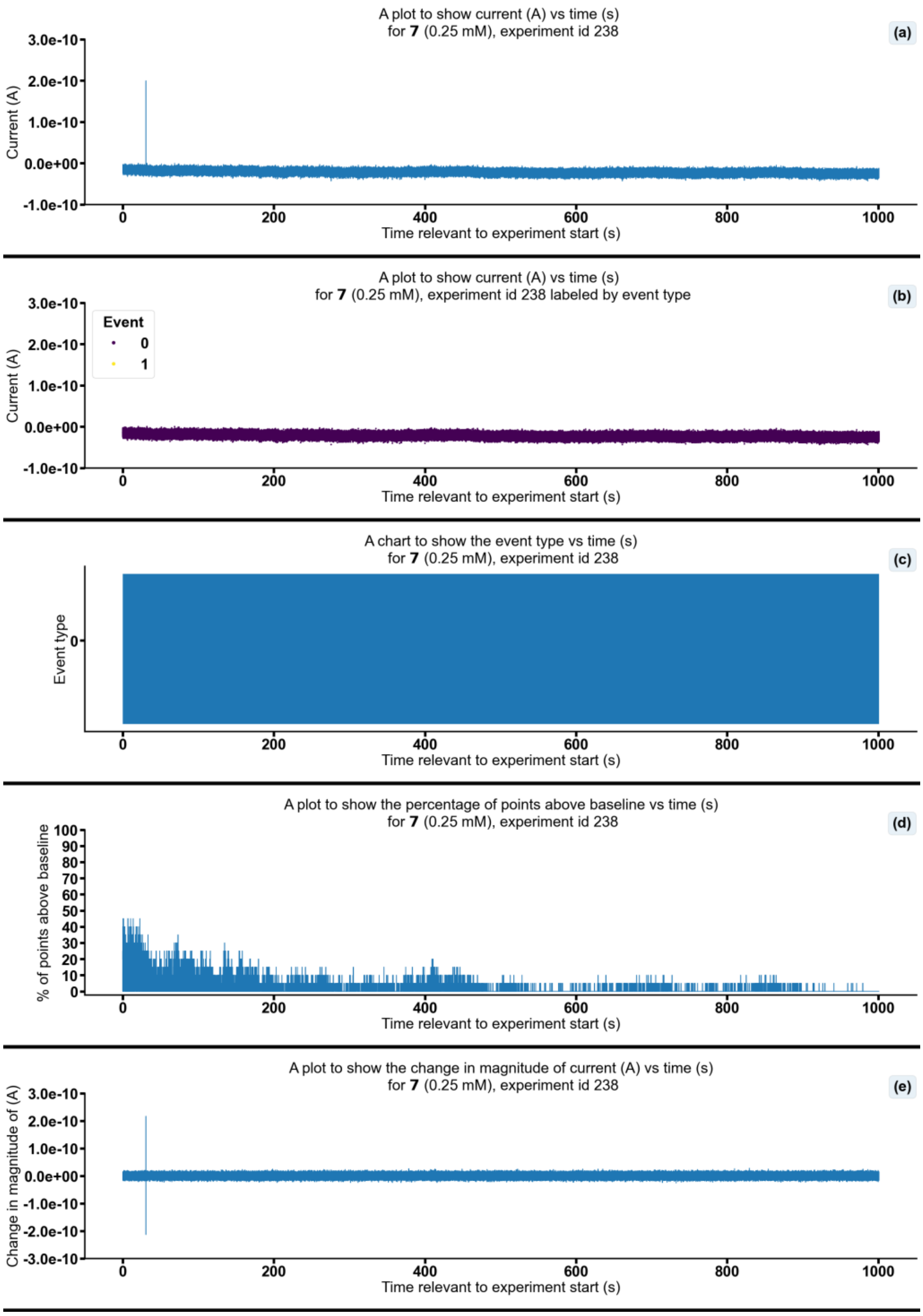

Figure S266 - Summarising the data from the patch clamp experiment for **7** (0.25 mM), experiment id 238 at +100 mV. Events were categorised into the following types: 0: -∞ A ≤ Event 0 < 5.00e-11 A, 1: 5.00e-11 A ≤ Event 1 < 5.00e-10 A, 2: 5.00e-10 A ≤ Event 2 < 4.90e-08 A, 3: 4.90e-08 A ≤ Event 3 < ∞ A. Subfigure (d) shows the percentage of points found above the threshold -1.1e-11 (A) in a rolling look ahead window of 20 points. The threshold is the mean + standard deviation of the noise of the first 200 datapoints.

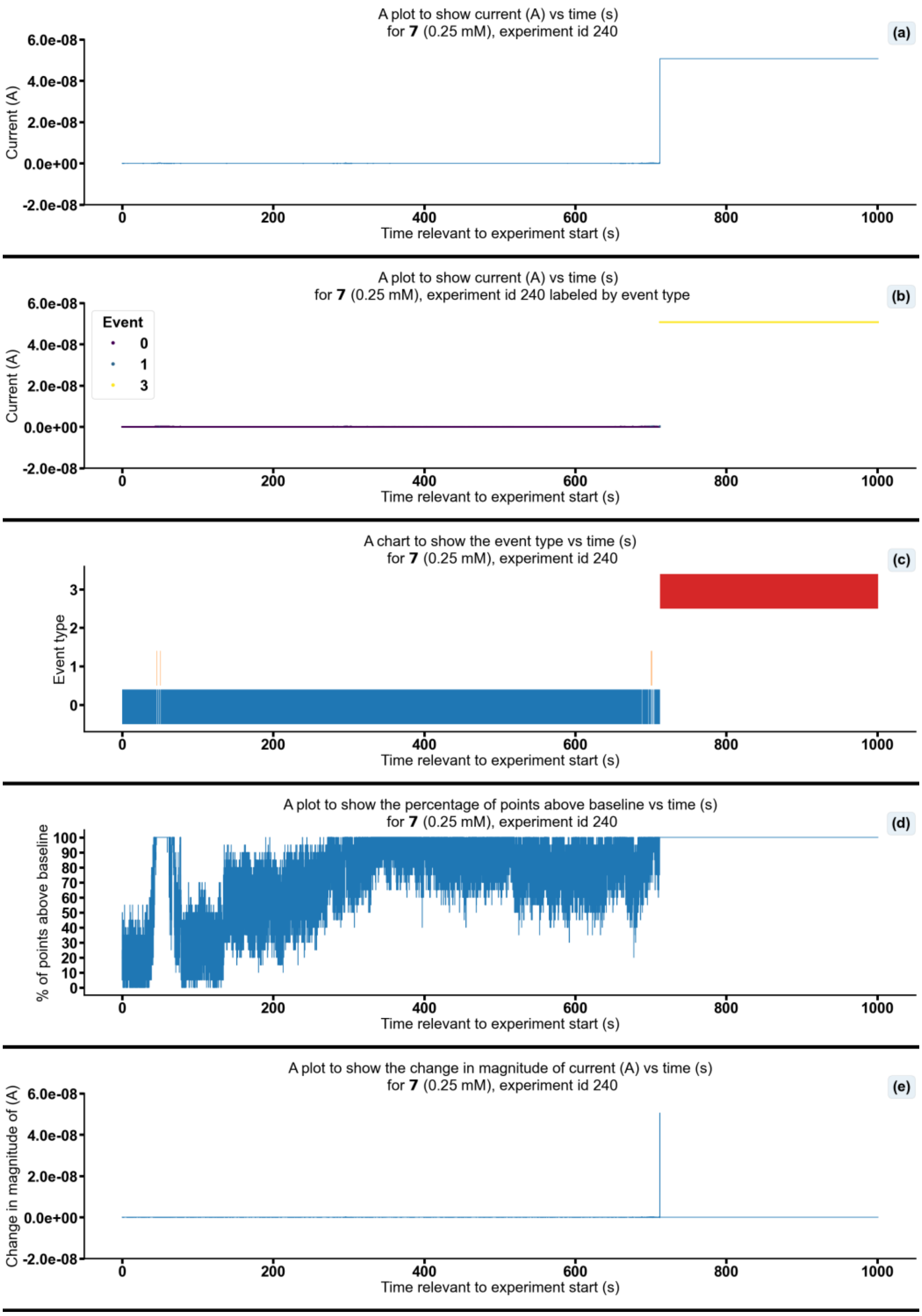

Figure S267 - Summarising the data from the patch clamp experiment for **7** (0.25 mM), experiment id 240 at +100 mV. Events were categorised into the following types: 0: -∞ A ≤ Event 0 < 5.00e-11 A, 1: 5.00e-11 A ≤ Event 1 < 5.00e-10 A, 2: 5.00e-10 A ≤ Event 2 < 4.90e-08 A, 3: 4.90e-08 A ≤ Event 3 < ∞ A. Subfigure (d) shows the percentage of points found above the threshold -2.3e-11 (A) in a rolling look ahead window of 20 points. The threshold is the mean + standard deviation of the noise of the first 200 datapoints.

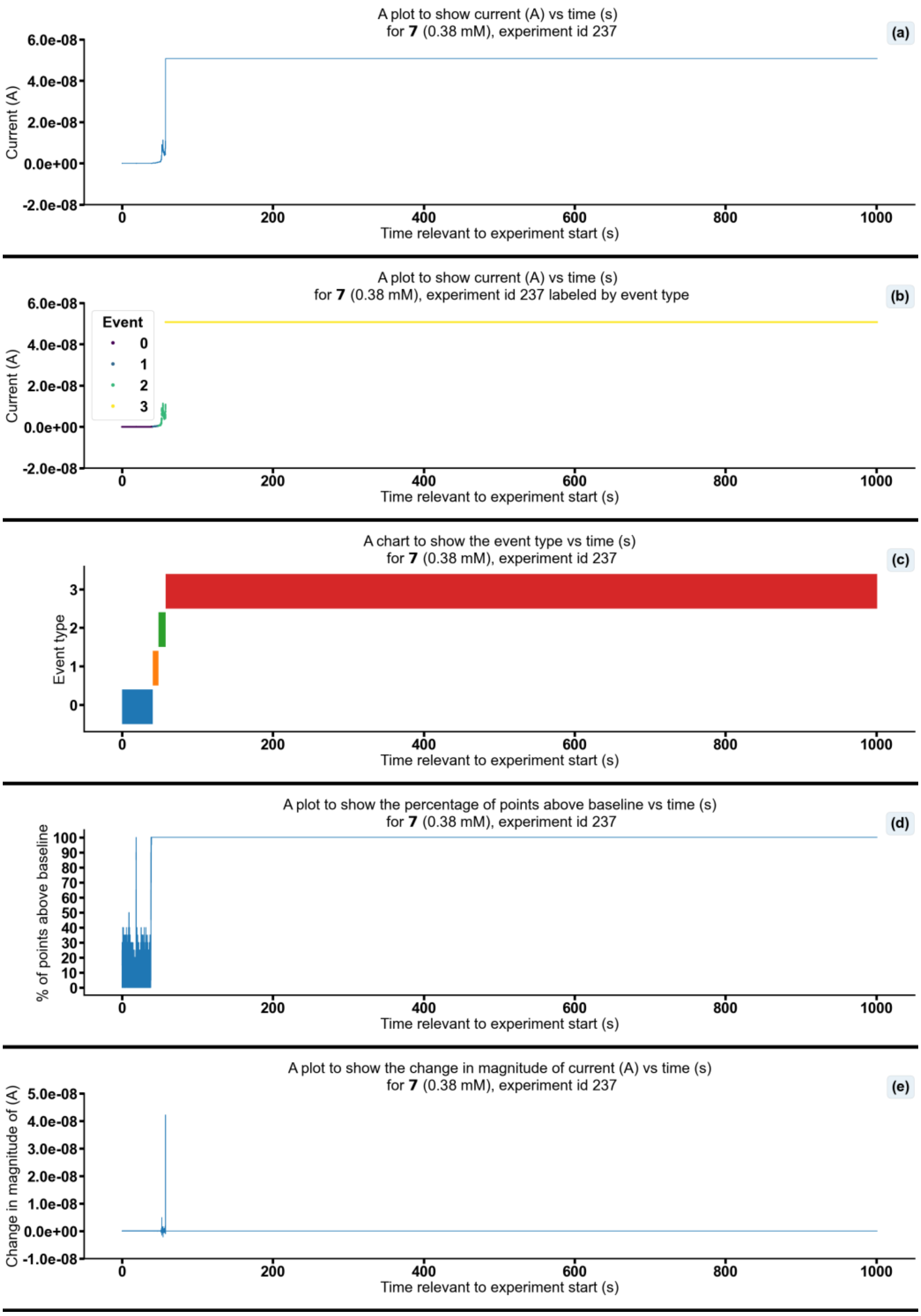

Figure S268 - Summarising the data from the patch clamp experiment for **7** (0.38 mM), experiment id 237 at +100 mV. Events were categorised into the following types: 0: -∞ A ≤ Event 0 < 5.00e-11 A, 1: 5.00e-11 A ≤ Event 1 < 5.00e-10 A, 2: 5.00e-10 A ≤ Event 2 < 4.90e-08 A, 3: 4.90e-08 A ≤ Event 3 < ∞ A. Subfigure (d) shows the percentage of points found above the threshold -1.5e-11 (A) in a rolling look ahead window of 20 points. The threshold is the mean + standard deviation of the noise of the first 200 datapoints.

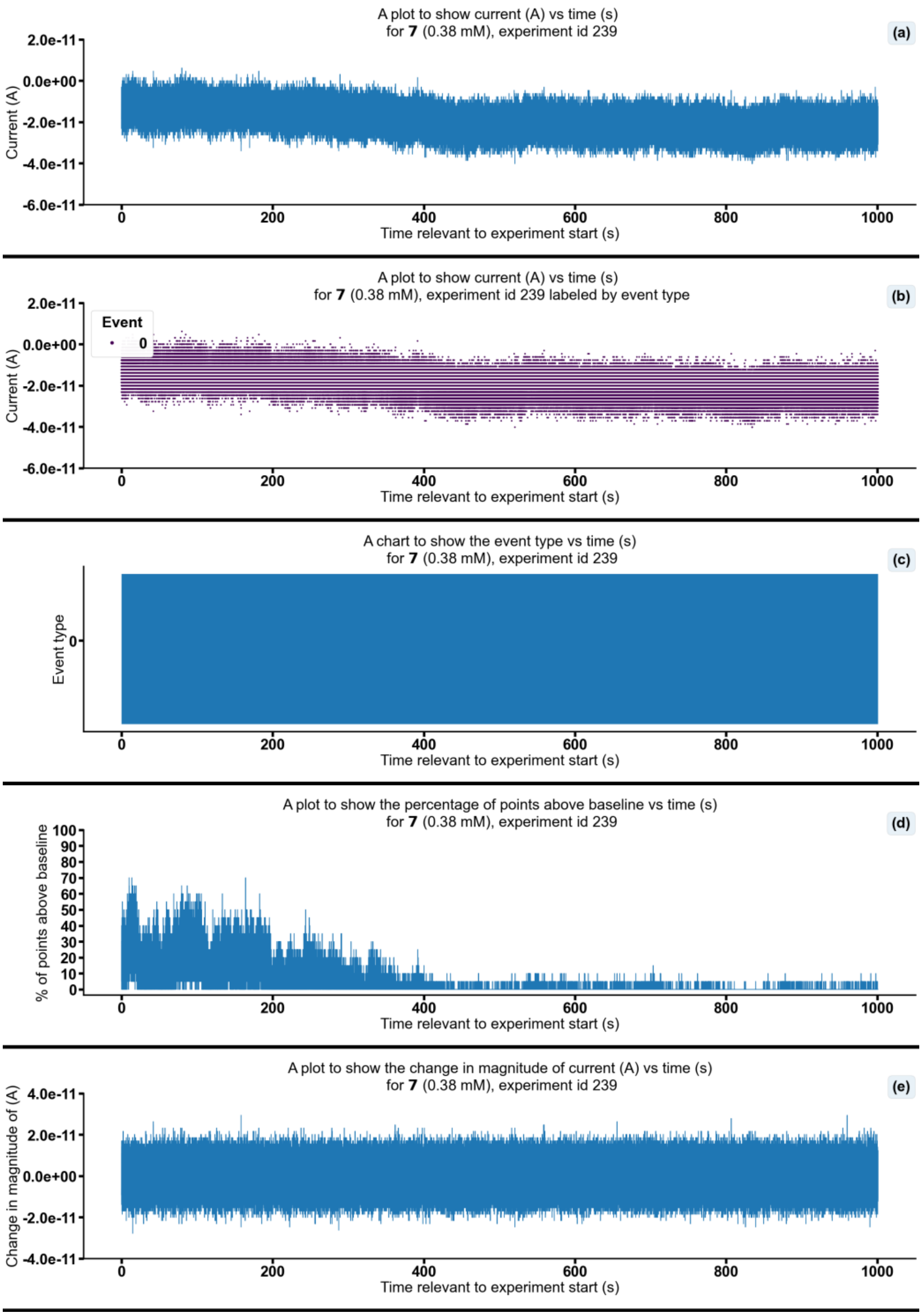

Figure S269 - Summarising the data from the patch clamp experiment for **7** (0.38 mM), experiment id 239 at +100 mV. Events were categorised into the following types: 0: -∞ A ≤ Event 0 < 5.00e-11 A, 1: 5.00e-11 A ≤ Event 1 < 5.00e-10 A, 2: 5.00e-10 A ≤ Event 2 < 4.90e-08 A, 3: 4.90e-08 A ≤ Event 3 < ∞ A. Subfigure (d) shows the percentage of points found above the threshold -9.5e-12 (A) in a rolling look ahead window of 20 points. The threshold is the mean + standard deviation of the noise of the first 200 datapoints.

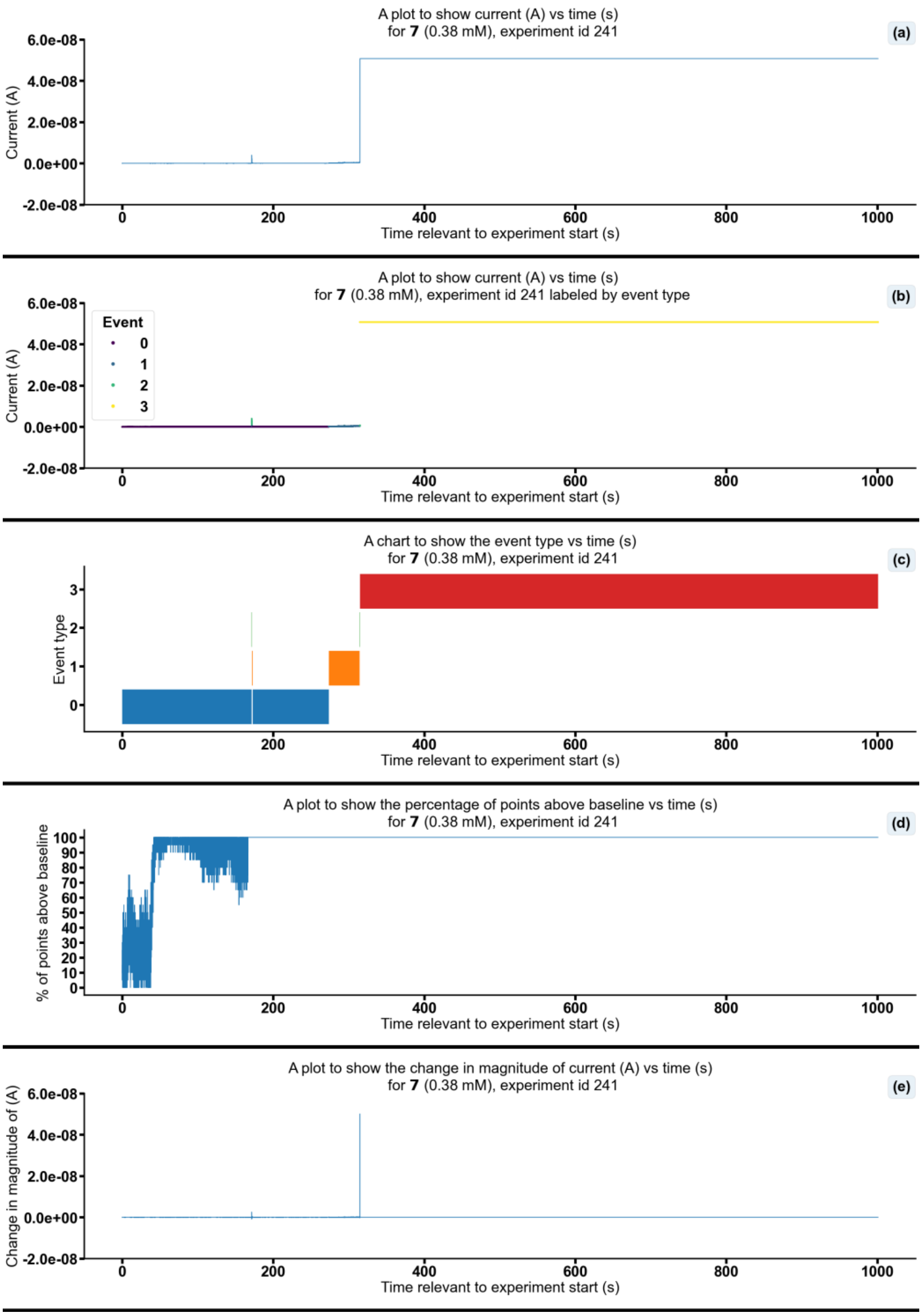

Figure S270 - Summarising the data from the patch clamp experiment for **7** (0.38 mM), experiment id 241 at +100 mV. Events were categorised into the following types: 0: -∞ A ≤ Event 0 < 5.00e-11 A, 1: 5.00e-11 A ≤ Event 1 < 5.00e-10 A, 2: 5.00e-10 A ≤ Event 2 < 4.90e-08 A, 3: 4.90e-08 A ≤ Event 3 < ∞ A. Subfigure (d) shows the percentage of points found above the threshold -9.1e-12 (A) in a rolling look ahead window of 20 points. The threshold is the mean + standard deviation of the noise of the first 200 datapoints.

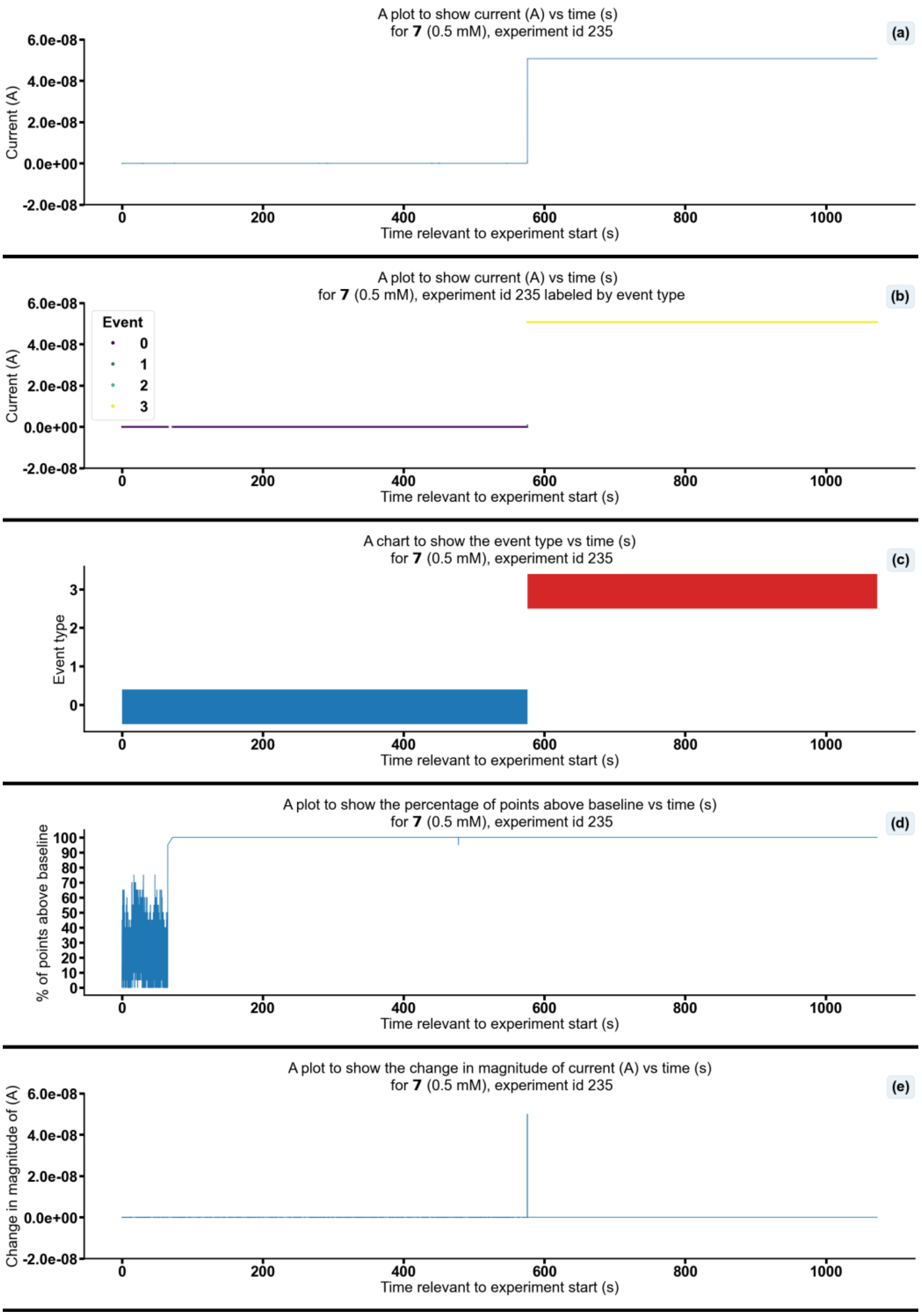

Figure S271 - Summarising the data from the patch clamp experiment for **7** (0.5 mM), experiment id 235 at +100 mV. Events were categorised into the following types: 0: -∞ A ≤ Event 0 < 5.00e-11 A, 1: 5.00e-11 A ≤ Event 1 < 5.00e-10 A, 2: 5.00e-10 A ≤ Event 2 < 4.90e-08 A, 3: 4.90e-08 A ≤ Event 3 < ∞ A. Subfigure (d) shows the percentage of points found above the threshold -4.5e-11 (A) in a rolling look ahead window of 20 points. The threshold is the mean + standard deviation of the noise of the first 200 datapoints.

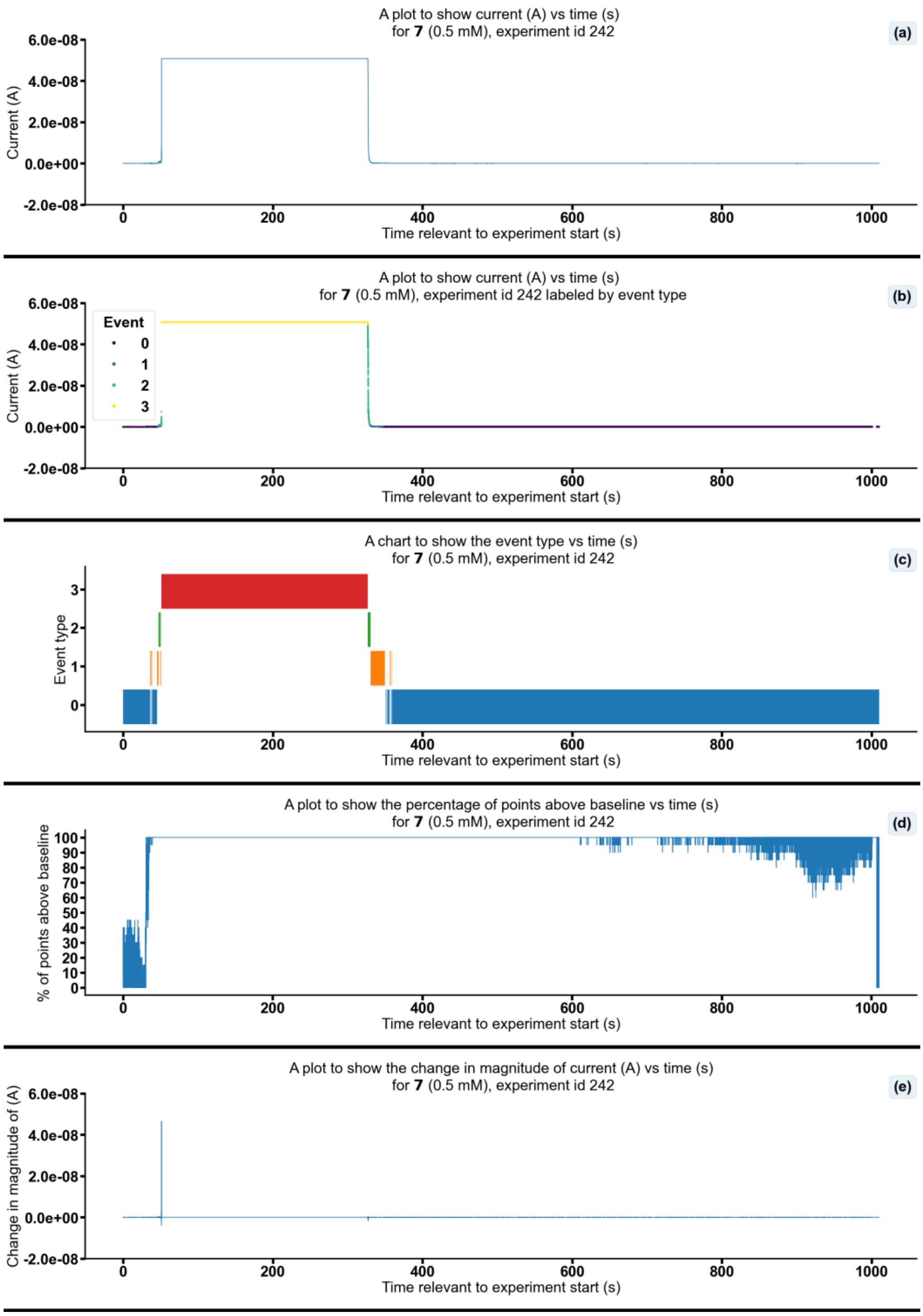

Figure S272 - Summarising the data from the patch clamp experiment for **7** (0.5 mM), experiment id 242 at +100 mV. Events were categorised into the following types: 0: -∞ A ≤ Event 0 < 5.00e-11 A, 1: 5.00e-11 A ≤ Event 1 < 5.00e-10 A, 2: 5.00e-10 A ≤ Event 2 < 4.90e-08 A, 3: 4.90e-08 A ≤ Event 3 < ∞ A. Subfigure (d) shows the percentage of points found above the threshold -9.4e-12 (A) in a rolling look ahead window of 20 points. The threshold is the mean + standard deviation of the noise of the first 200 datapoints.

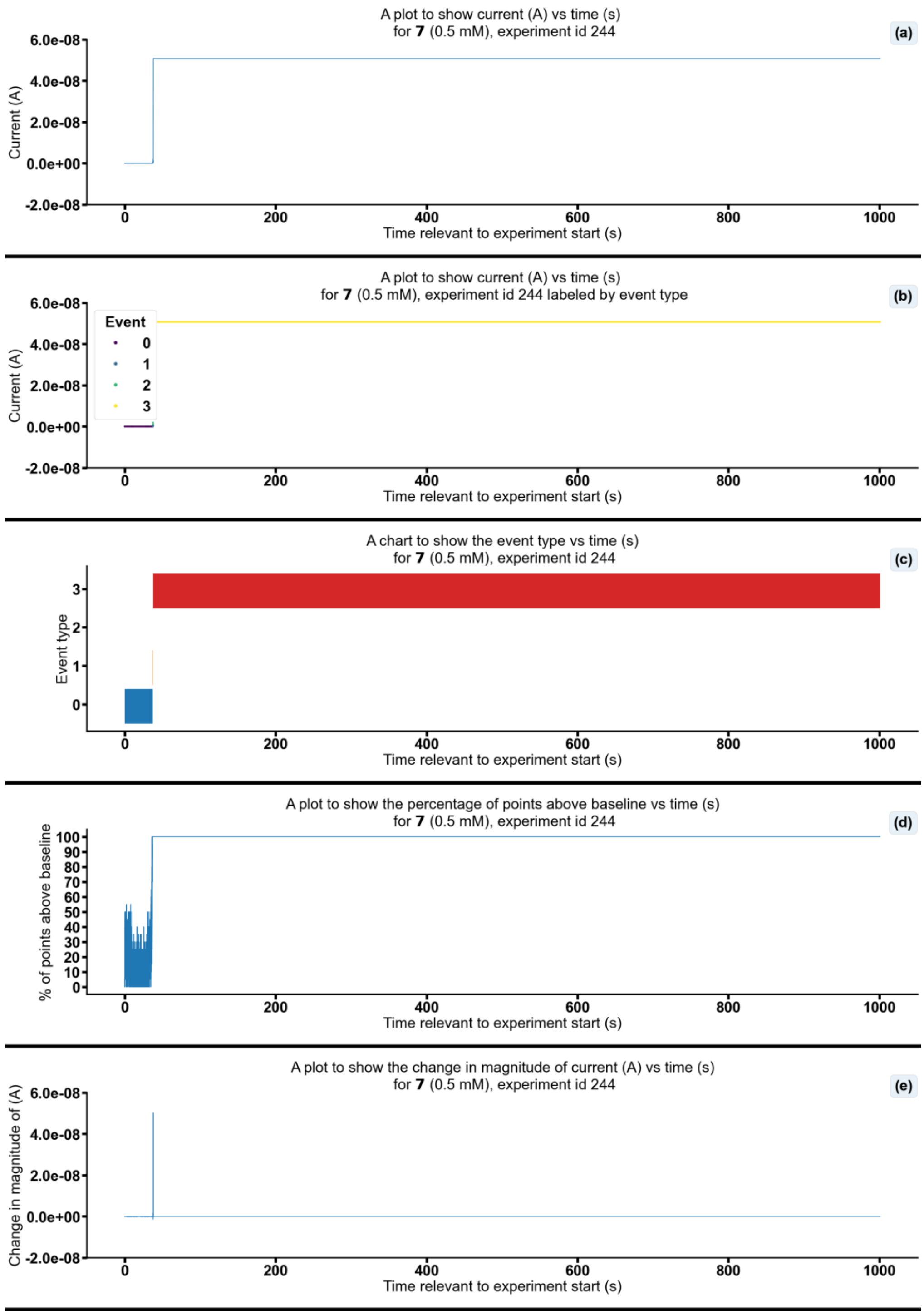

Figure S273 - Summarising the data from the patch clamp experiment for **7** (0.5 mM), experiment id 244 at +100 mV. Events were categorised into the following types: 0: -∞ A ≤ Event 0 < 5.00e-11 A, 1: 5.00e-11 A ≤ Event 1 < 5.00e-10 A, 2: 5.00e-10 A ≤ Event 2 < 4.90e-08 A, 3: 4.90e-08 A ≤ Event 3 < ∞ A. Subfigure (d) shows the percentage of points found above the threshold -1.5e-11 (A) in a rolling look ahead window of 20 points. The threshold is the mean + standard deviation of the noise of the first 200 datapoints.

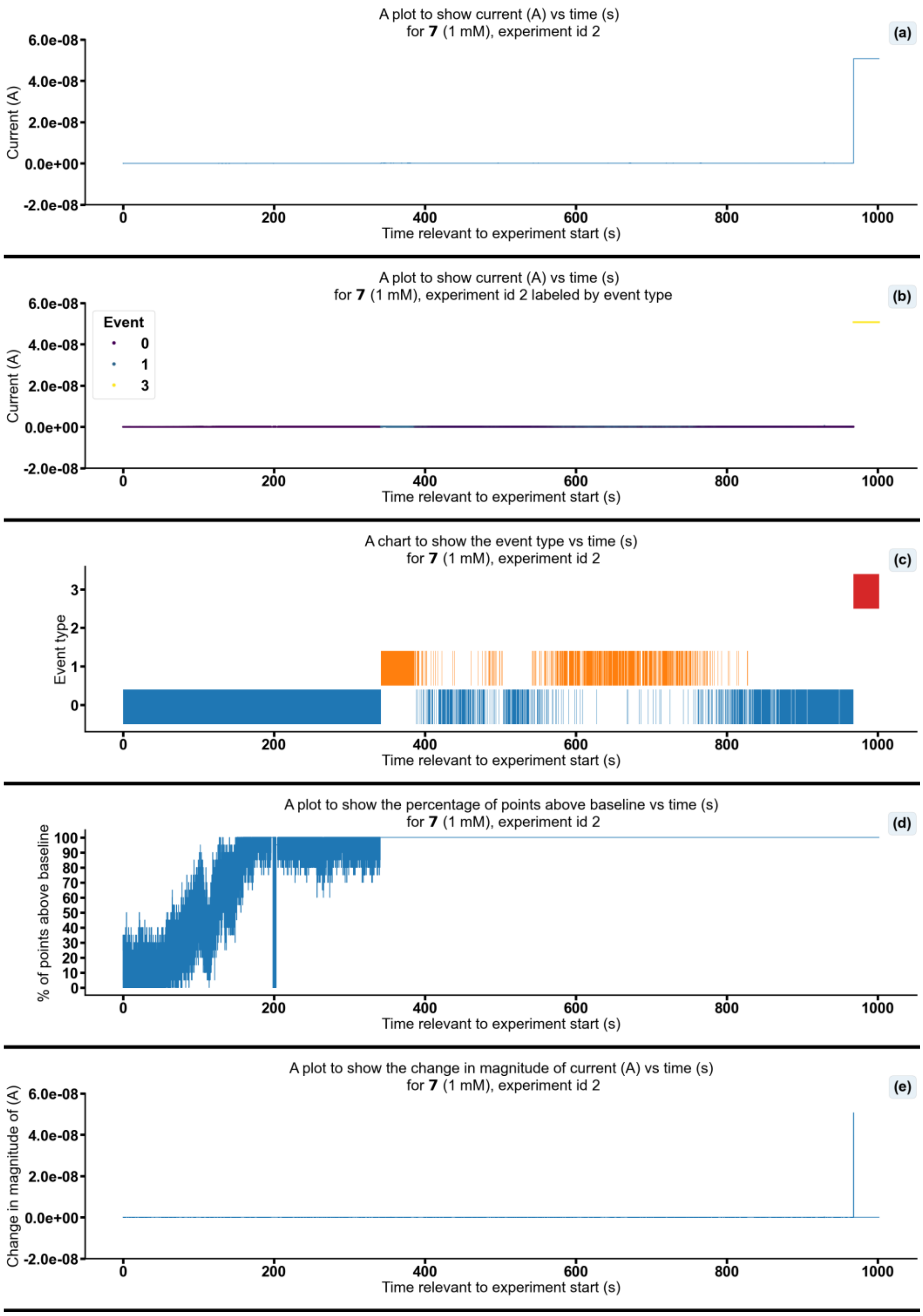

Figure S274 - Summarising the data from the patch clamp experiment for **7** (1 mM), experiment id 2 at +100 mV. Events were categorised into the following types: 0: -∞ A ≤ Event 0 < 5.00e-11 A, 1: 5.00e-11 A ≤ Event 1 < 5.00e-10 A, 2: 5.00e-10 A ≤ Event 2 < 4.90e-08 A, 3: 4.90e-08 A ≤ Event 3 < ∞ A. Subfigure (d) shows the percentage of points found above the threshold -1.2e-11 (A) in a rolling look ahead window of 20 points. The threshold is the mean + standard deviation of the noise of the first 200 datapoints.

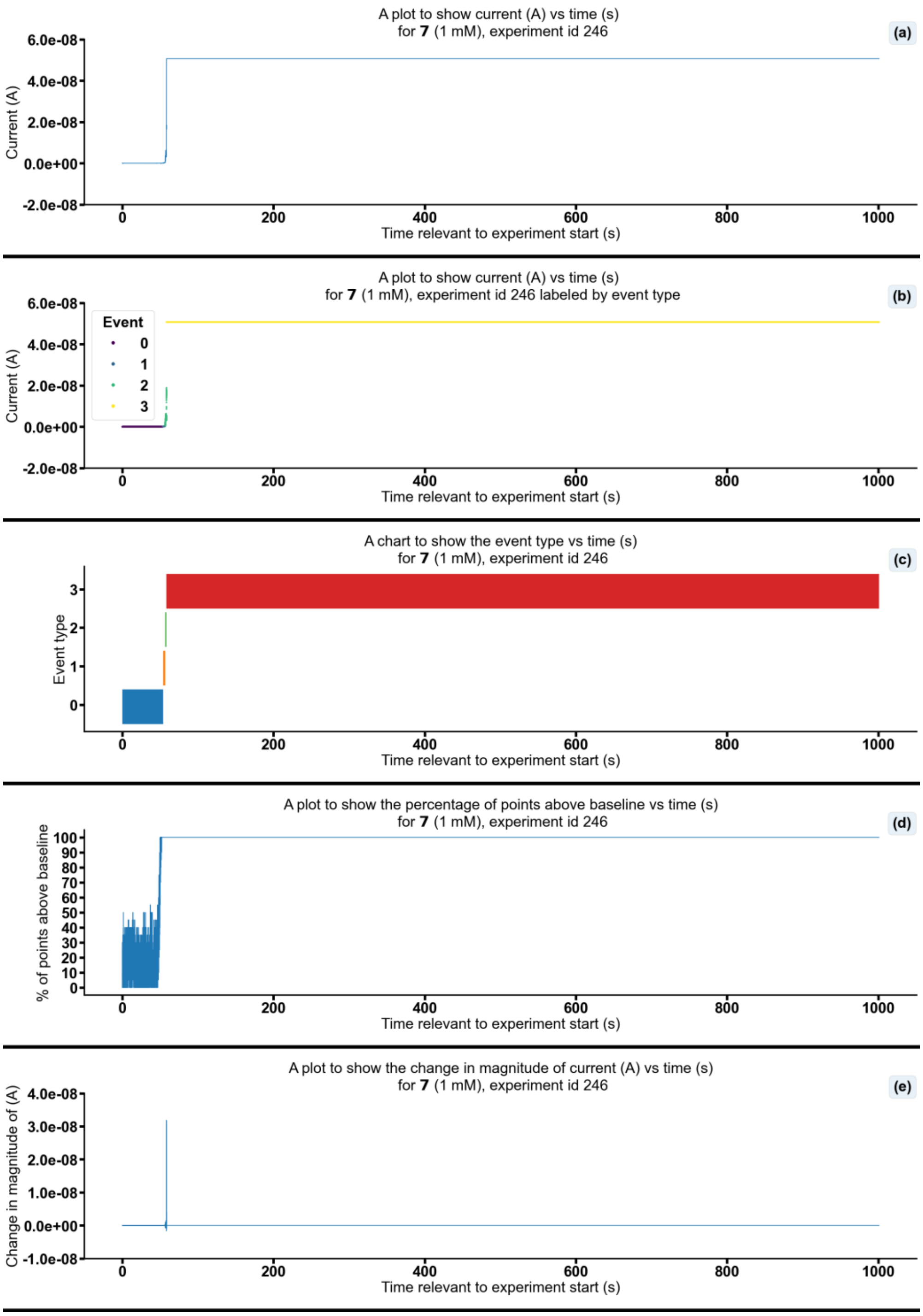

Figure S275 - Summarising the data from the patch clamp experiment for **7** (1 mM), experiment id 246 at +100 mV. Events were categorised into the following types: 0: -∞ A ≤ Event 0 < 5.00e-11 A, 1: 5.00e-11 A ≤ Event 1 < 5.00e-10 A, 2: 5.00e-10 A ≤ Event 2 < 4.90e-08 A, 3: 4.90e-08 A ≤ Event 3 < ∞ A. Subfigure (d) shows the percentage of points found above the threshold -3.1e-12 (A) in a rolling look ahead window of 20 points. The threshold is the mean + standard deviation of the noise of the first 200 datapoints.

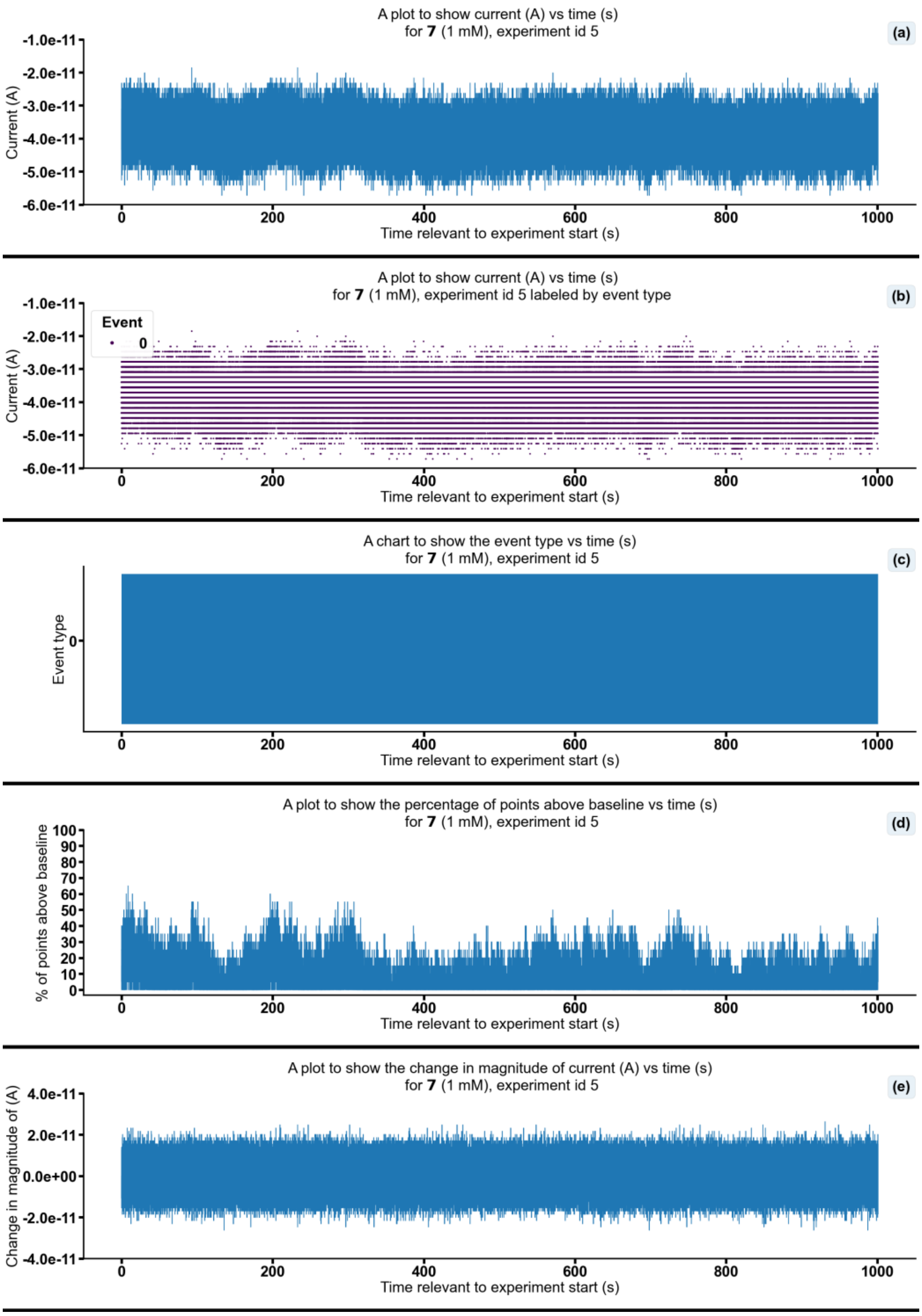

Figure S276 - Summarising the data from the patch clamp experiment for **7** (1 mM), experiment id 5 at +100 mV. Events were categorised into the following types: 0: -∞ A ≤ Event 0 < 5.00e-11 A, 1: 5.00e-11 A ≤ Event 1 < 5.00e-10 A, 2: 5.00e-10 A ≤ Event 2 < 4.90e-08 A, 3: 4.90e-08 A ≤ Event 3 < ∞ A. Subfigure (d) shows the percentage of points found above the threshold -3.3e-11 (A) in a rolling look ahead window of 20 points. The threshold is the mean + standard deviation of the noise of the first 200 datapoints.

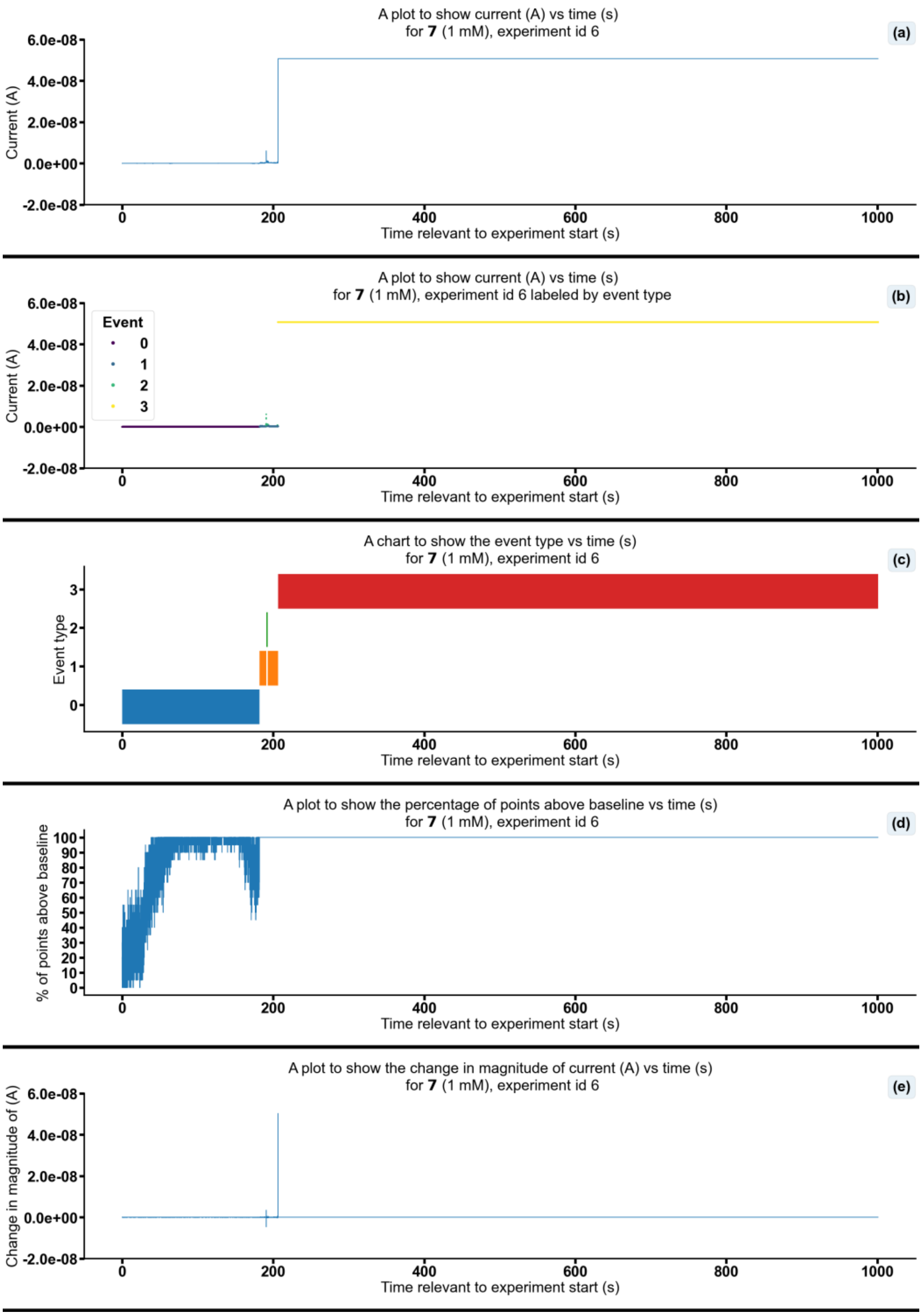

Figure S277 - Summarising the data from the patch clamp experiment for **7** (1 mM), experiment id 6 at +100 mV. Events were categorised into the following types: 0: -∞ A ≤ Event 0 < 5.00e-11 A, 1: 5.00e-11 A ≤ Event 1 < 5.00e-10 A, 2: 5.00e-10 A ≤ Event 2 < 4.90e-08 A, 3: 4.90e-08 A ≤ Event 3 < ∞ A. Subfigure (d) shows the percentage of points found above the threshold -4.7e-12 (A) in a rolling look ahead window of 20 points. The threshold is the mean + standard deviation of the noise of the first 200 datapoints.

Changing the concentration of **9**

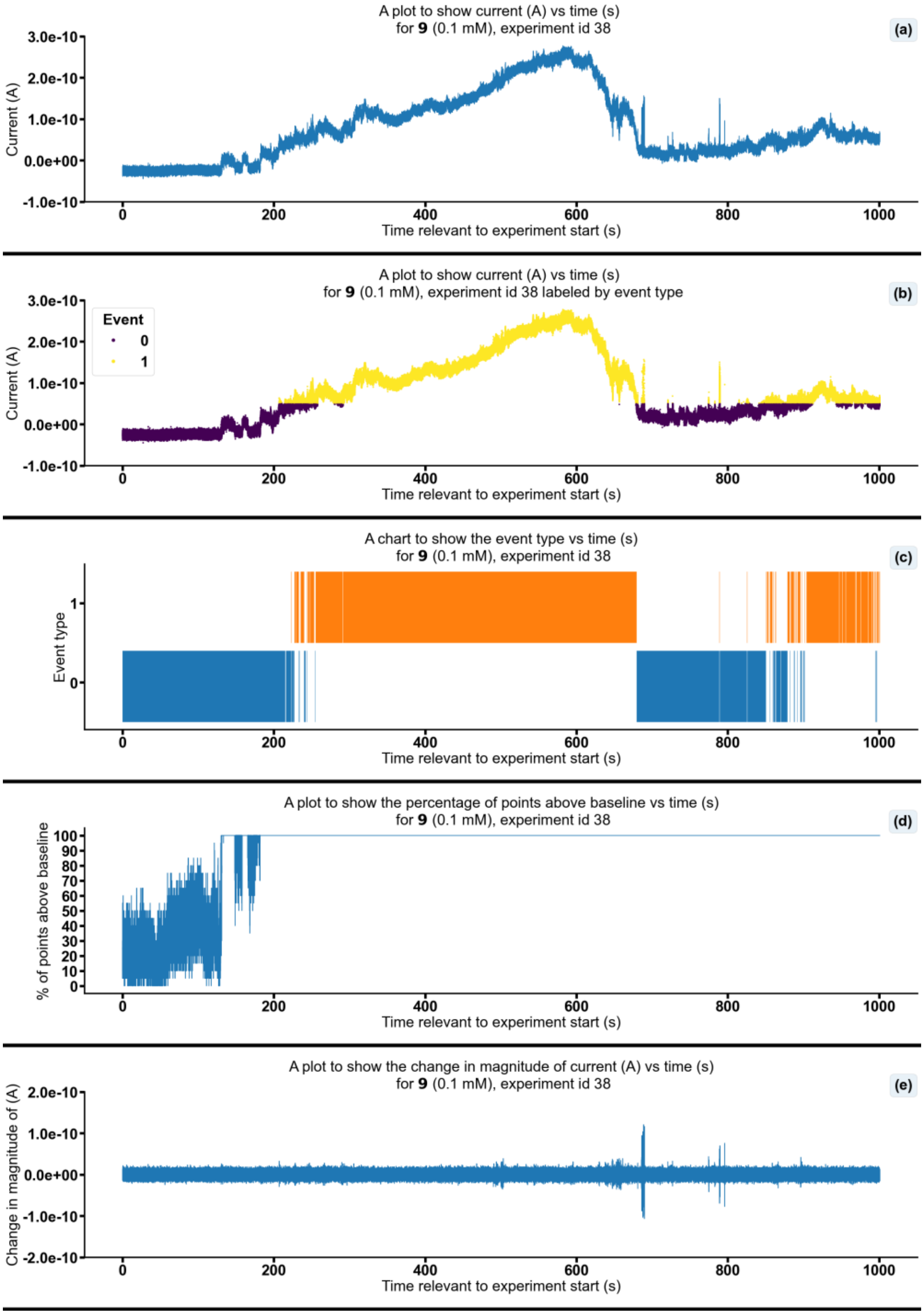

Figure S278 - Summarising the data from the patch clamp experiment for **9** (0.1 mM), experiment id 38 at +100 mV. Events were categorised into the following types: 0: -∞ A ≤ Event 0 < 5.00e-11 A, 1: 5.00e-11 A ≤ Event 1 < 5.00e-10 A, 2: 5.00e-10 A ≤ Event 2 < 4.90e-08 A, 3: 4.90e-08 A ≤ Event 3 < ∞ A. Subfigure (d) shows the percentage of points found above the threshold -2.2e-11 (A) in a rolling look ahead window of 20 points. The threshold is the mean + standard deviation of the noise of the first 200 datapoints.

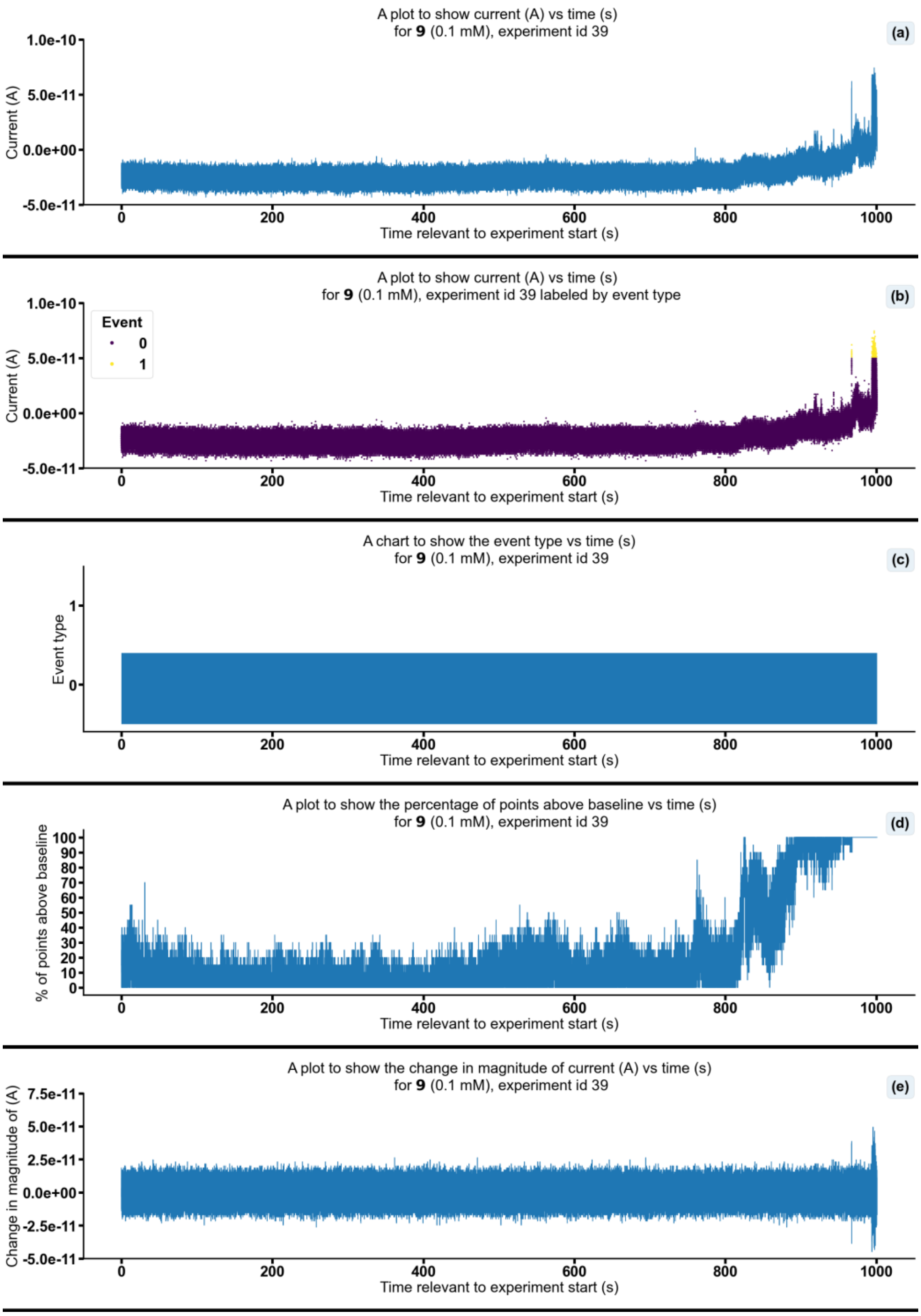

Figure S279 - Summarising the data from the patch clamp experiment for **9** (0.1 mM), experiment id 39 at +100 mV. Events were categorised into the following types: 0: -∞ A ≤ Event 0 < 5.00e-11 A, 1: 5.00e-11 A ≤ Event 1 < 5.00e-10 A, 2: 5.00e-10 A ≤ Event 2 < 4.90e-08 A, 3: 4.90e-08 A ≤ Event 3 < ∞ A. Subfigure (d) shows the percentage of points found above the threshold -1.9e-11 (A) in a rolling look ahead window of 20 points. The threshold is the mean + standard deviation of the noise of the first 200 datapoints.

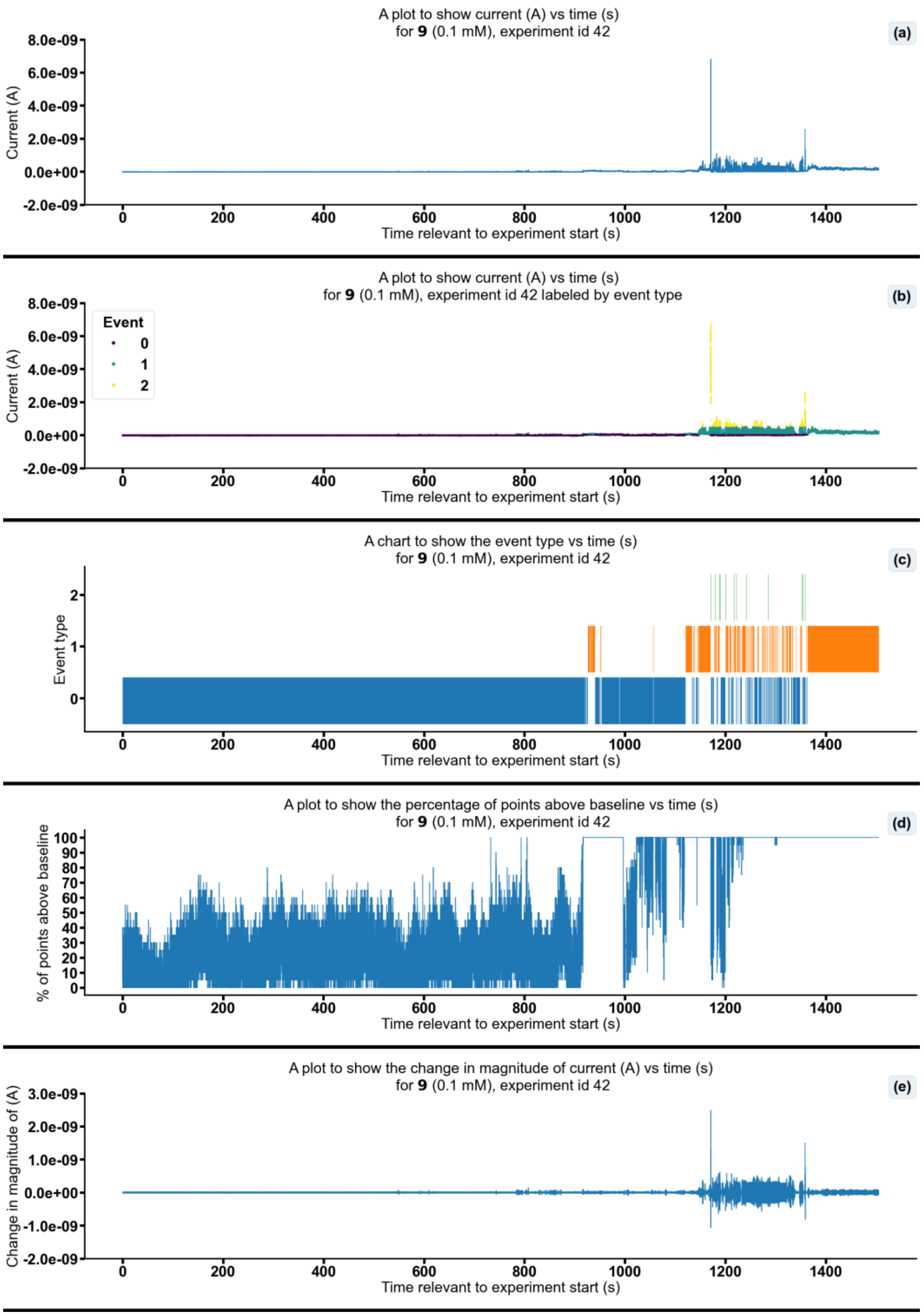

Figure S280 - Summarising the data from the patch clamp experiment for **9** (0.1 mM), experiment id 42 at +100 mV. Events were categorised into the following types: 0: -∞ A ≤ Event 0 < 5.00e-11 A, 1: 5.00e-11 A ≤ Event 1 < 5.00e-10 A, 2: 5.00e-10 A ≤ Event 2 < 4.90e-08 A, 3: 4.90e-08 A ≤ Event 3 < ∞ A. Subfigure (d) shows the percentage of points found above the threshold -1.7e-11 (A) in a rolling look ahead window of 20 points. The threshold is the mean + standard deviation of the noise of the first 200 datapoints.

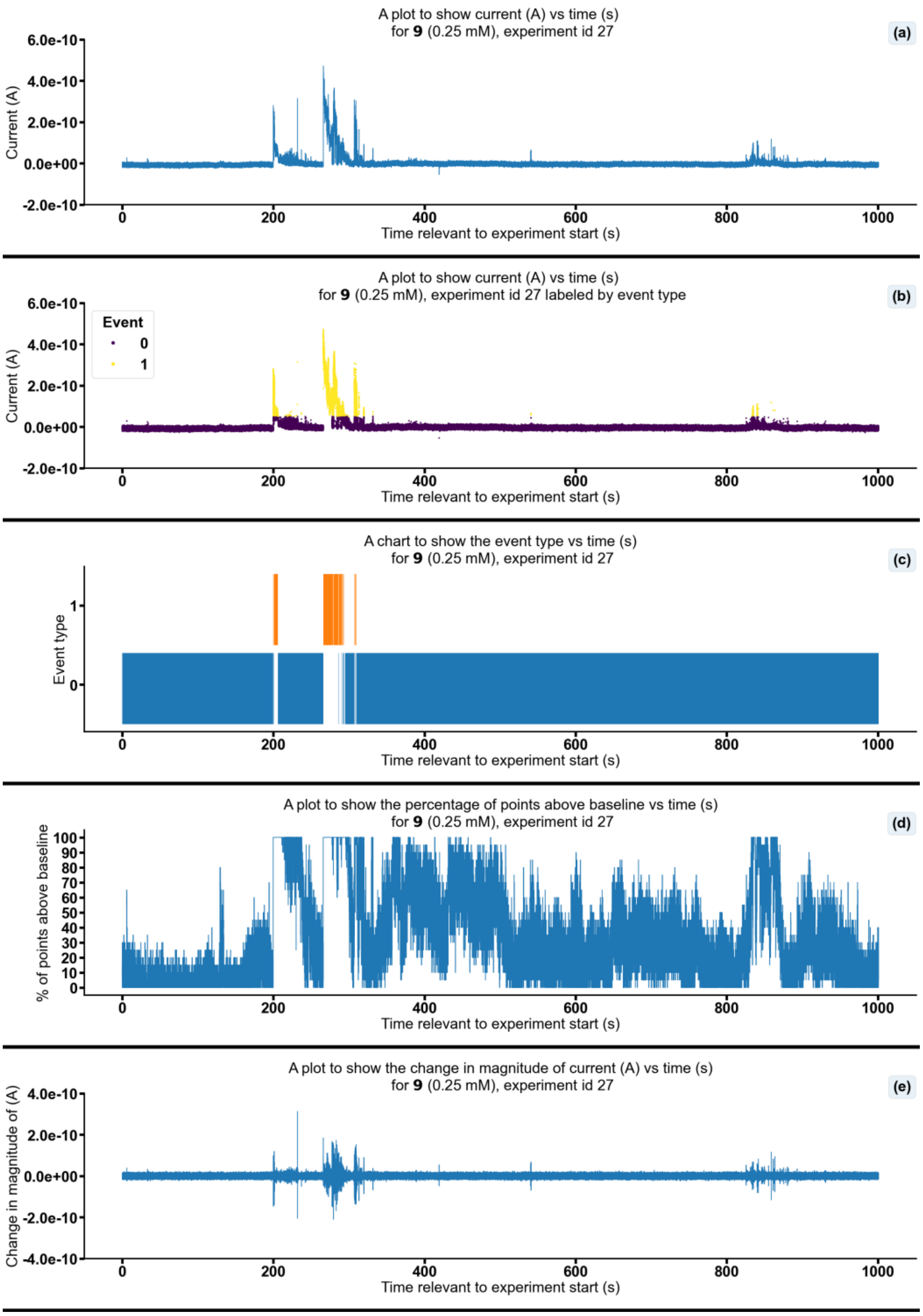

Figure S281 - Summarising the data from the patch clamp experiment for **9** (0.25 mM), experiment id 27 at +100 mV. Events were categorised into the following types: 0: -∞ A ≤ Event 0 < 5.00e-11 A, 1: 5.00e-11 A ≤ Event 1 < 5.00e-10 A, 2: 5.00e-10 A ≤ Event 2 < 4.90e-08 A, 3: 4.90e-08 A ≤ Event 3 < ∞ A. Subfigure (d) shows the percentage of points found above the threshold -3.0e-12 (A) in a rolling look ahead window of 20 points. The threshold is the mean + standard deviation of the noise of the first 200 datapoints.

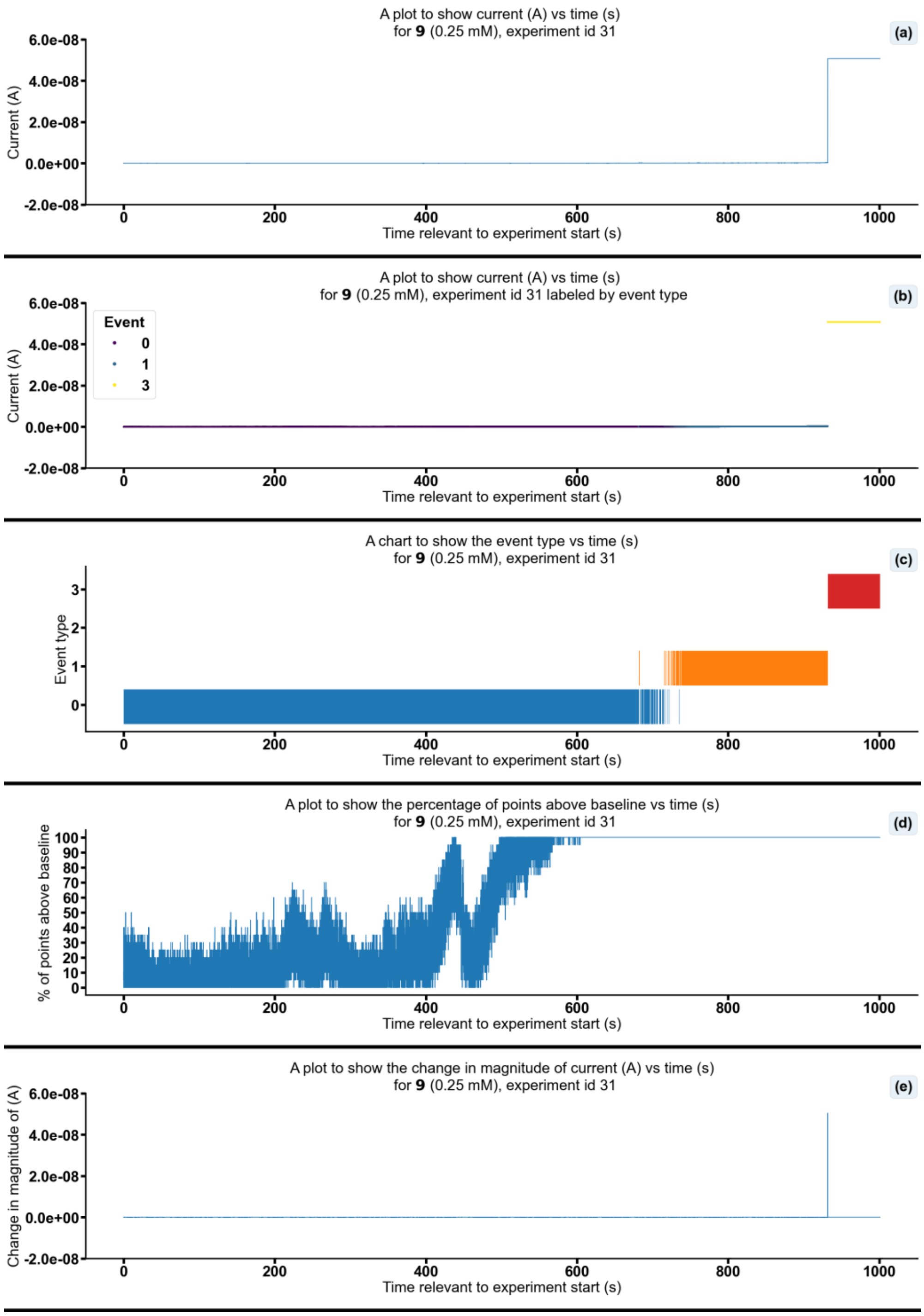

Figure S282 - Summarising the data from the patch clamp experiment for **9** (0.25 mM), experiment id 31 at +100 mV. Events were categorised into the following types: 0: -∞ A ≤ Event 0 < 5.00e-11 A, 1: 5.00e-11 A ≤ Event 1 < 5.00e-10 A, 2: 5.00e-10 A ≤ Event 2 < 4.90e-08 A, 3: 4.90e-08 A ≤ Event 3 < ∞ A. Subfigure (d) shows the percentage of points found above the threshold -7.3e-12 (A) in a rolling look ahead window of 20 points. The threshold is the mean + standard deviation of the noise of the first 200 datapoints.

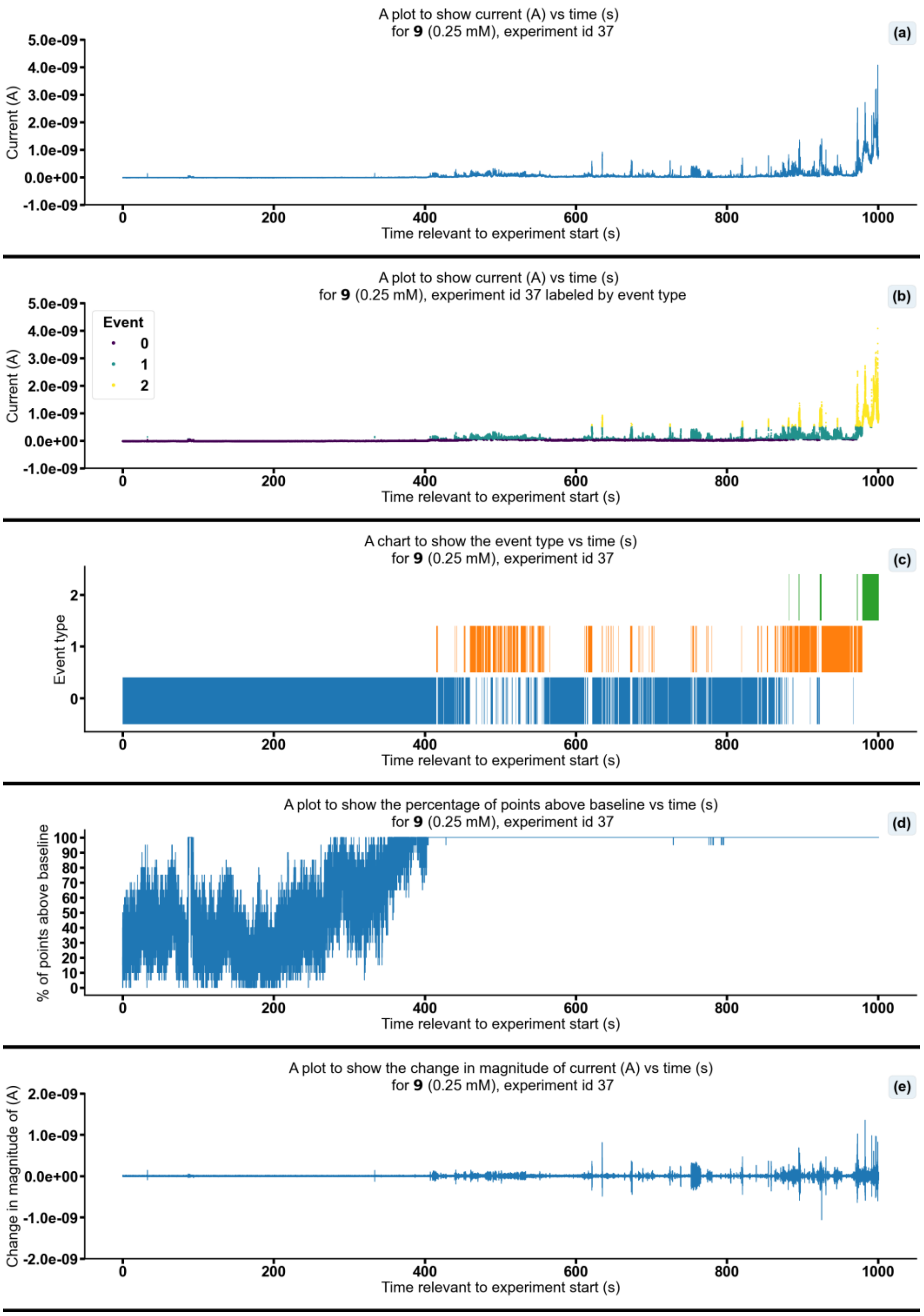

Figure S283 - Summarising the data from the patch clamp experiment for **9** (0.25 mM), experiment id 37 at +100 mV. Events were categorised into the following types: 0: -∞ A ≤ Event 0 < 5.00e-11 A, 1: 5.00e-11 A ≤ Event 1 < 5.00e-10 A, 2: 5.00e-10 A ≤ Event 2 < 4.90e-08 A, 3: 4.90e-08 A ≤ Event 3 < ∞ A. Subfigure (d) shows the percentage of points found above the threshold -1.7e-11 (A) in a rolling look ahead window of 20 points. The threshold is the mean + standard deviation of the noise of the first 200 datapoints.

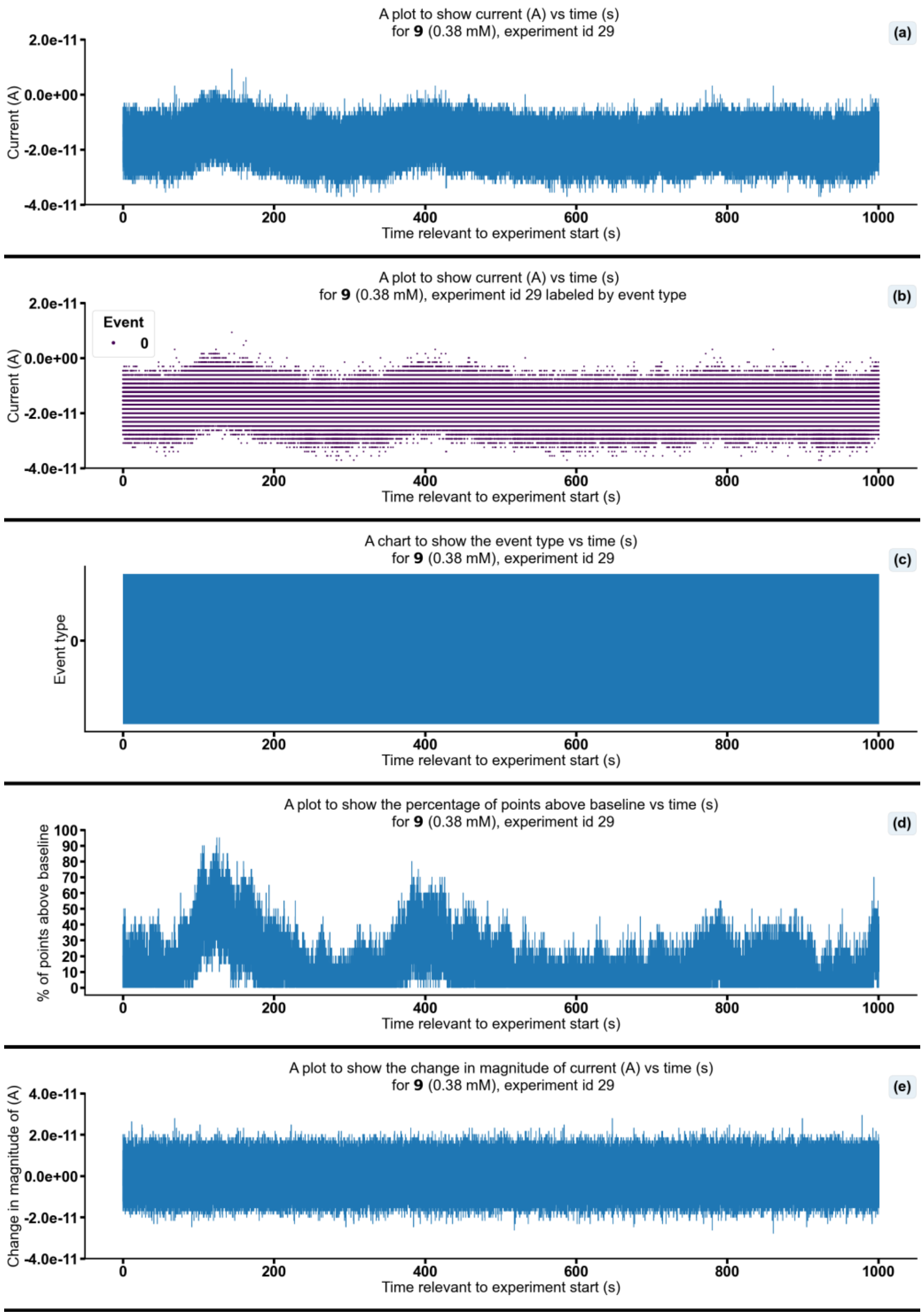

Figure S284 - Summarising the data from the patch clamp experiment for **9** (0.38 mM), experiment id 29 at +100 mV. Events were categorised into the following types: 0: -∞ A ≤ Event 0 < 5.00e-11 A, 1: 5.00e-11 A ≤ Event 1 < 5.00e-10 A, 2: 5.00e-10 A ≤ Event 2 < 4.90e-08 A, 3: 4.90e-08 A ≤ Event 3 < ∞ A. Subfigure (d) shows the percentage of points found above the threshold -1.3e-11 (A) in a rolling look ahead window of 20 points. The threshold is the mean + standard deviation of the noise of the first 200 datapoints.

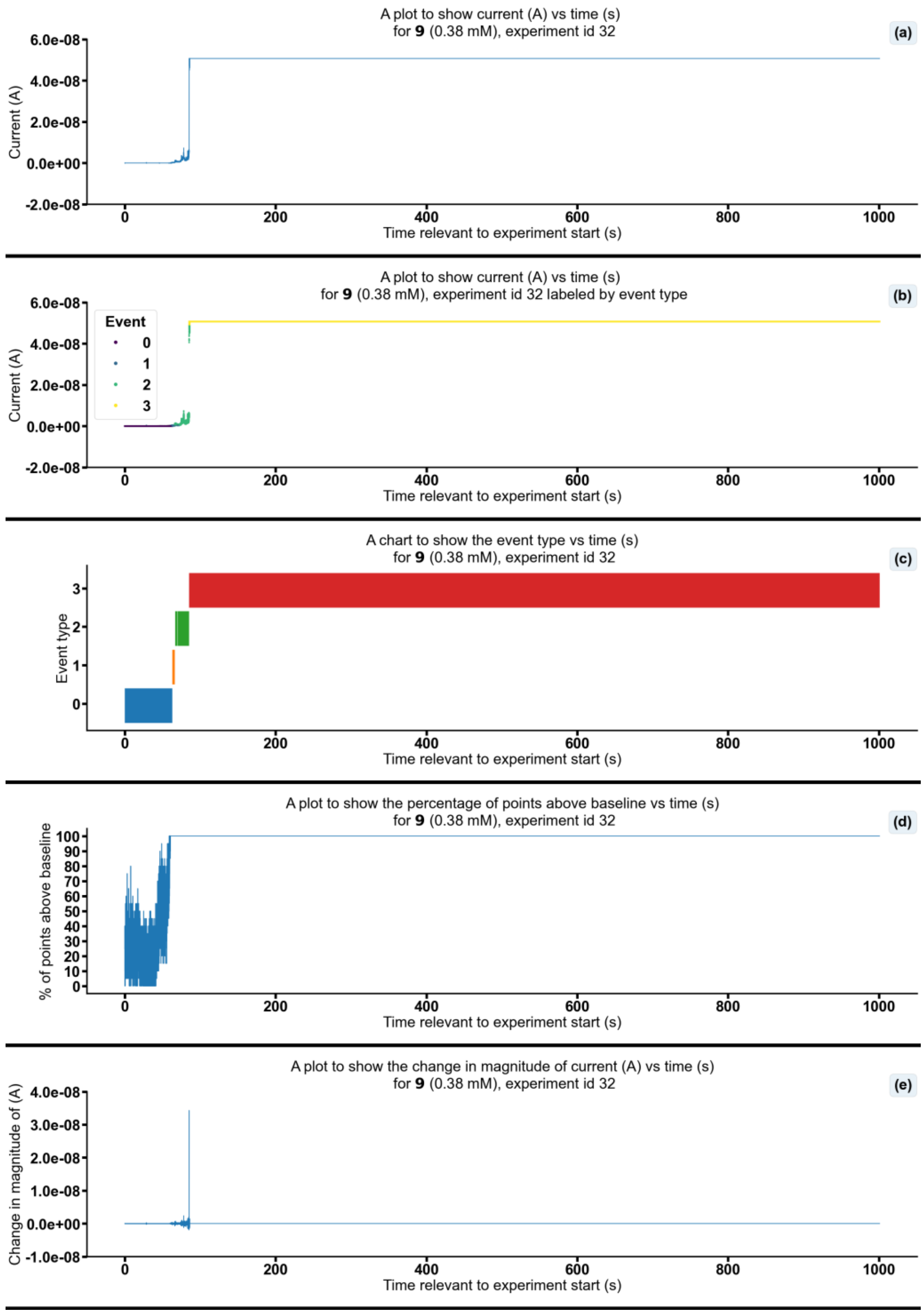

Figure S285 - Summarising the data from the patch clamp experiment for **9** (0.38 mM), experiment id 32 at +100 mV. Events were categorised into the following types: 0: -∞ A ≤ Event 0 < 5.00e-11 A, 1: 5.00e-11 A ≤ Event 1 < 5.00e-10 A, 2: 5.00e-10 A ≤ Event 2 < 4.90e-08 A, 3: 4.90e-08 A ≤ Event 3 < ∞ A. Subfigure (d) shows the percentage of points found above the threshold -1.4e-11 (A) in a rolling look ahead window of 20 points. The threshold is the mean + standard deviation of the noise of the first 200 datapoints.

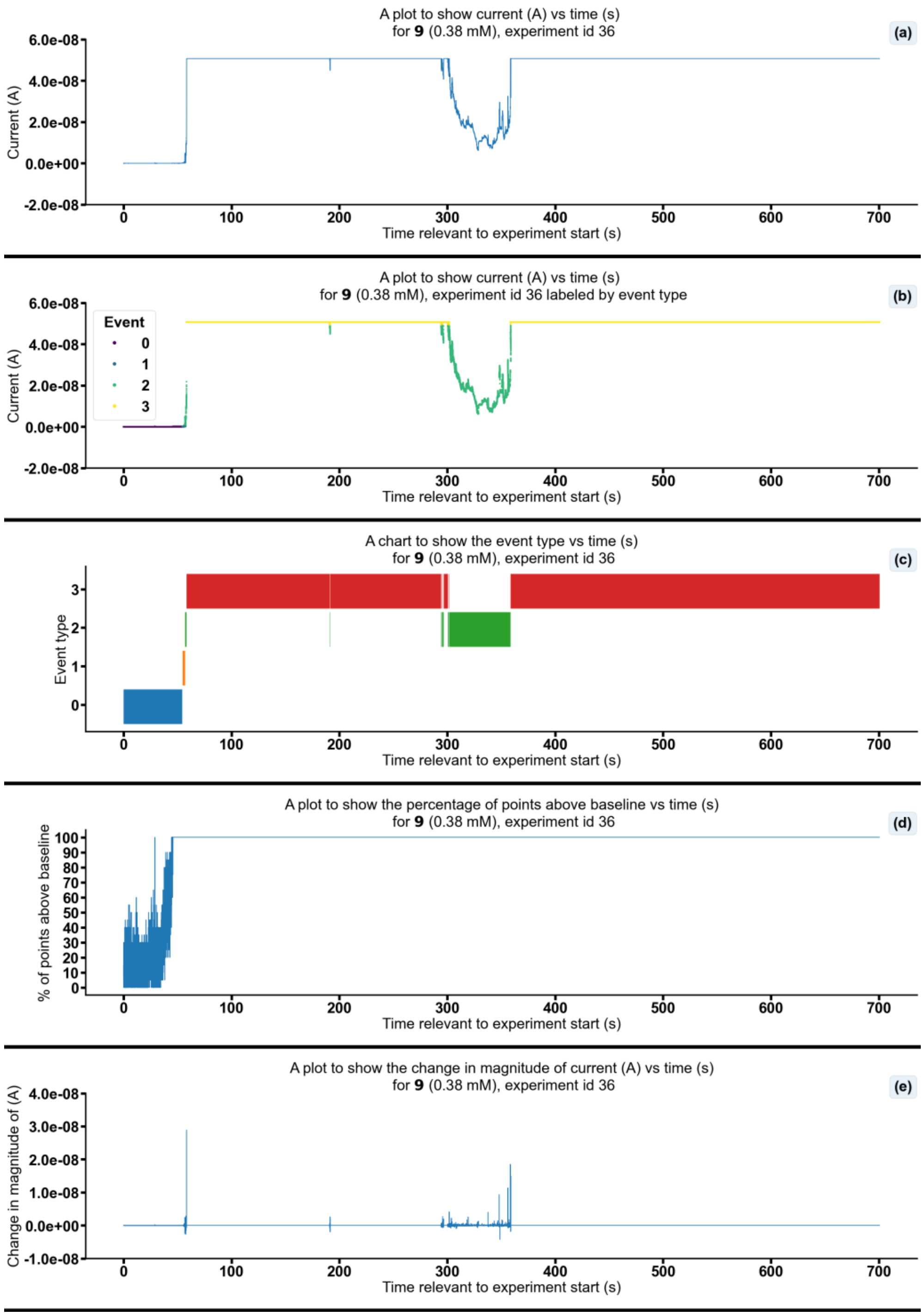

Figure S286 - Summarising the data from the patch clamp experiment for **9** (0.38 mM), experiment id 36 at +100 mV. Events were categorised into the following types: 0: -∞ A ≤ Event 0 < 5.00e-11 A, 1: 5.00e-11 A ≤ Event 1 < 5.00e-10 A, 2: 5.00e-10 A ≤ Event 2 < 4.90e-08 A, 3: 4.90e-08 A ≤ Event 3 < ∞ A. Subfigure (d) shows the percentage of points found above the threshold -1.8e-11 (A) in a rolling look ahead window of 20 points. The threshold is the mean + standard deviation of the noise of the first 200 datapoints.

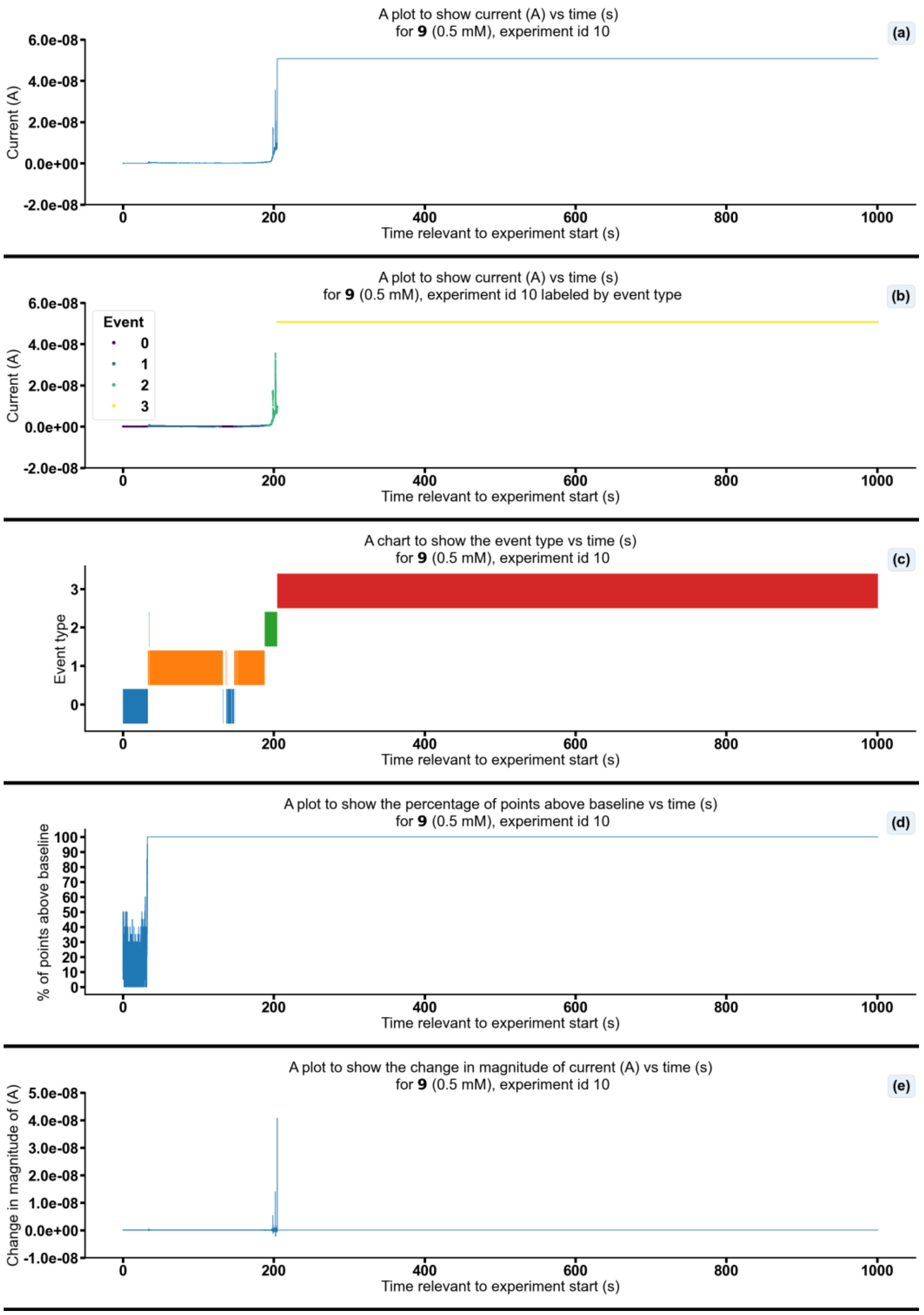

Figure S287 - Summarising the data from the patch clamp experiment for **9** (0.5 mM), experiment id 10 at +100 mV. Events were categorised into the following types: 0: -∞ A ≤ Event 0 < 5.00e-11 A, 1: 5.00e-11 A ≤ Event 1 < 5.00e-10 A, 2: 5.00e-10 A ≤ Event 2 < 4.90e-08 A, 3: 4.90e-08 A ≤ Event 3 < ∞ A. Subfigure (d) shows the percentage of points found above the threshold -8.0e-12 (A) in a rolling look ahead window of 20 points. The threshold is the mean + standard deviation of the noise of the first 200 datapoints.

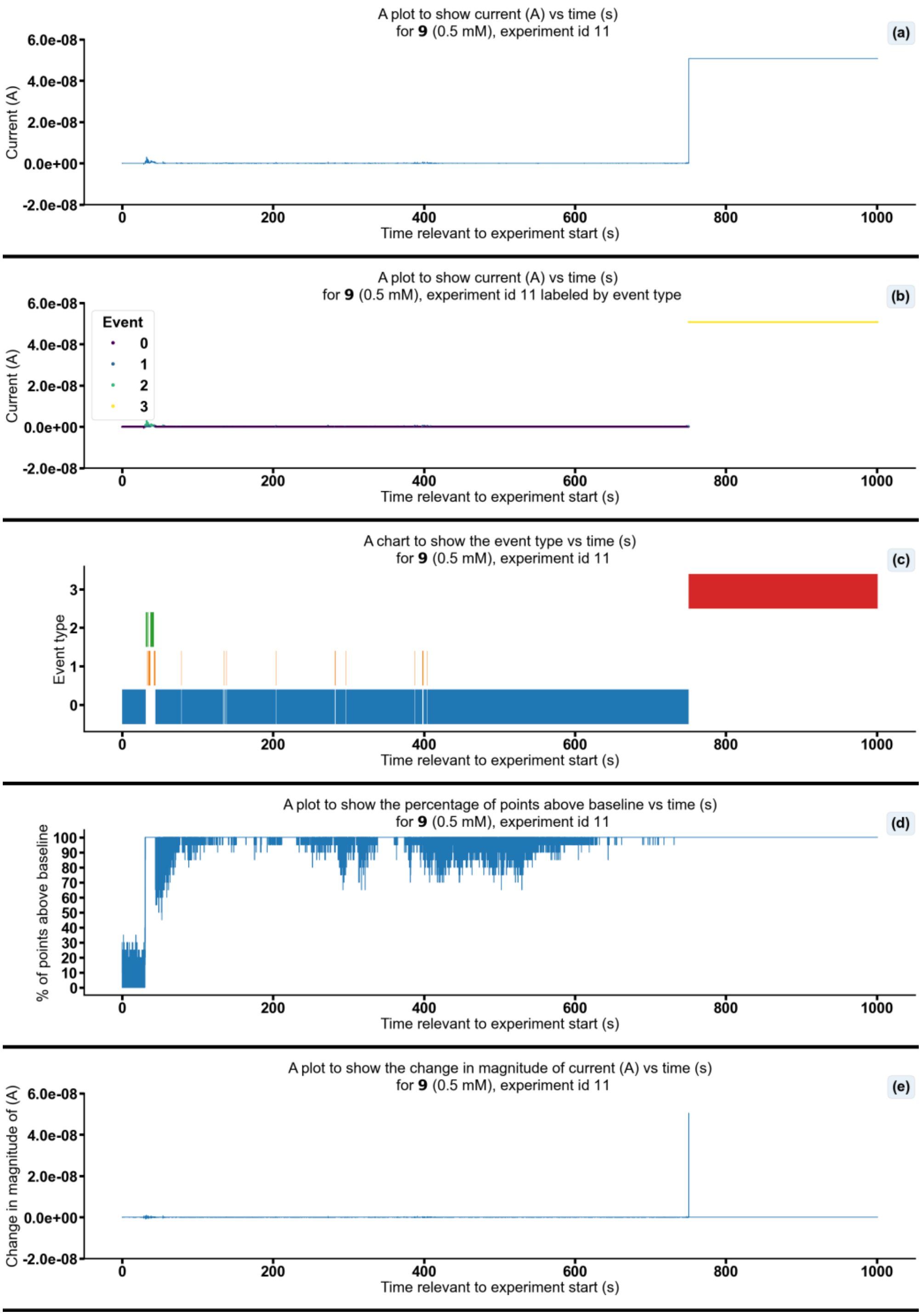

Figure S288 - Summarising the data from the patch clamp experiment for **9** (0.5 mM), experiment id 11 at +100 mV. Events were categorised into the following types: 0: -∞ A ≤ Event 0 < 5.00e-11 A, 1: 5.00e-11 A ≤ Event 1 < 5.00e-10 A, 2: 5.00e-10 A ≤ Event 2 < 4.90e-08 A, 3: 4.90e-08 A ≤ Event 3 < ∞ A. Subfigure (d) shows the percentage of points found above the threshold -4.5e-12 (A) in a rolling look ahead window of 20 points. The threshold is the mean + standard deviation of the noise of the first 200 datapoints.

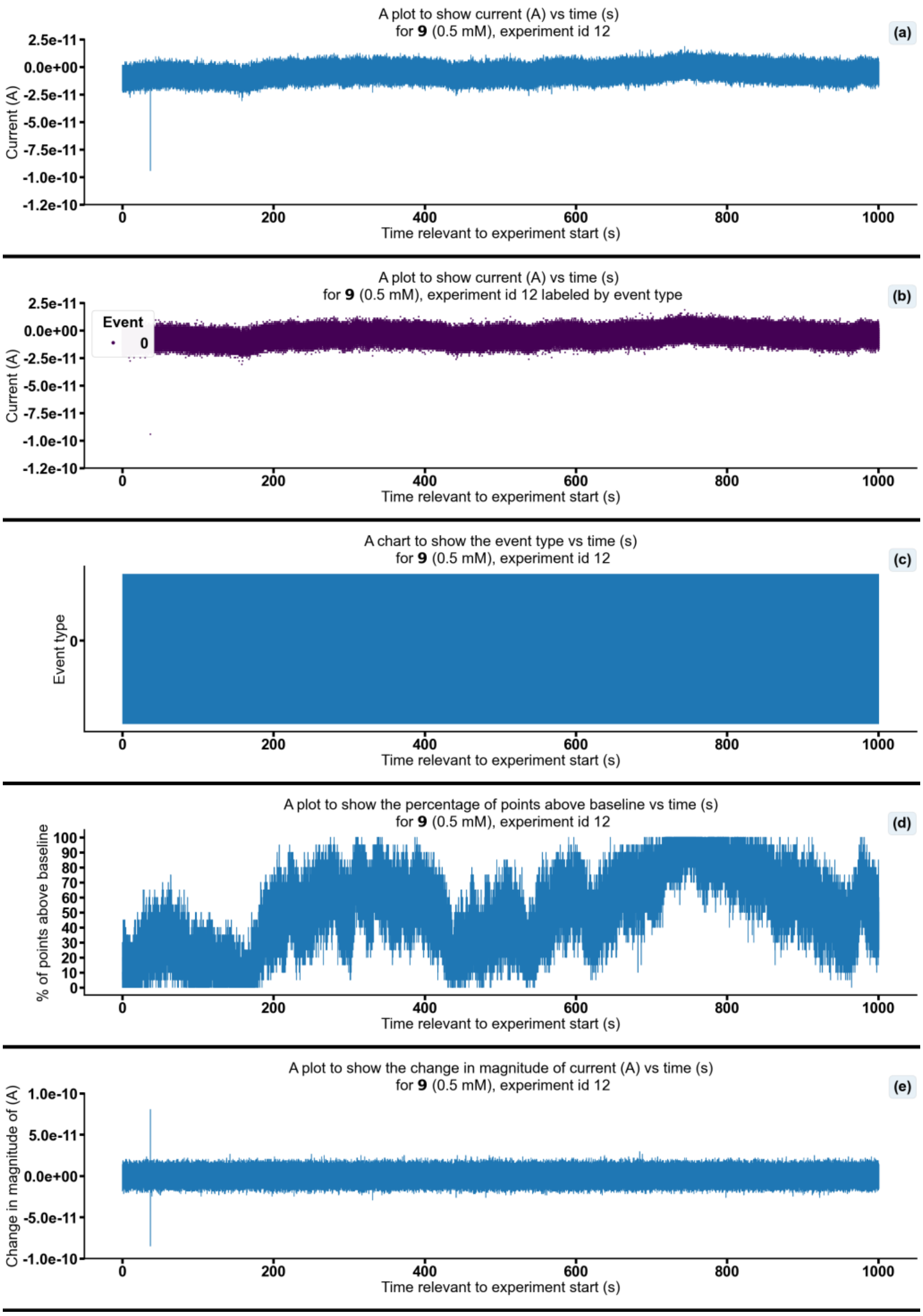

Figure S289 - Summarising the data from the patch clamp experiment for **9** (0.5 mM), experiment id 12 at +100 mV. Events were categorised into the following types: 0: -∞ A ≤ Event 0 < 5.00e-11 A, 1: 5.00e-11 A ≤ Event 1 < 5.00e-10 A, 2: 5.00e-10 A ≤ Event 2 < 4.90e-08 A, 3: 4.90e-08 A ≤ Event 3 < ∞ A. Subfigure (d) shows the percentage of points found above the threshold -5.2e-12 (A) in a rolling look ahead window of 20 points. The threshold is the mean + standard deviation of the noise of the first 200 datapoints.

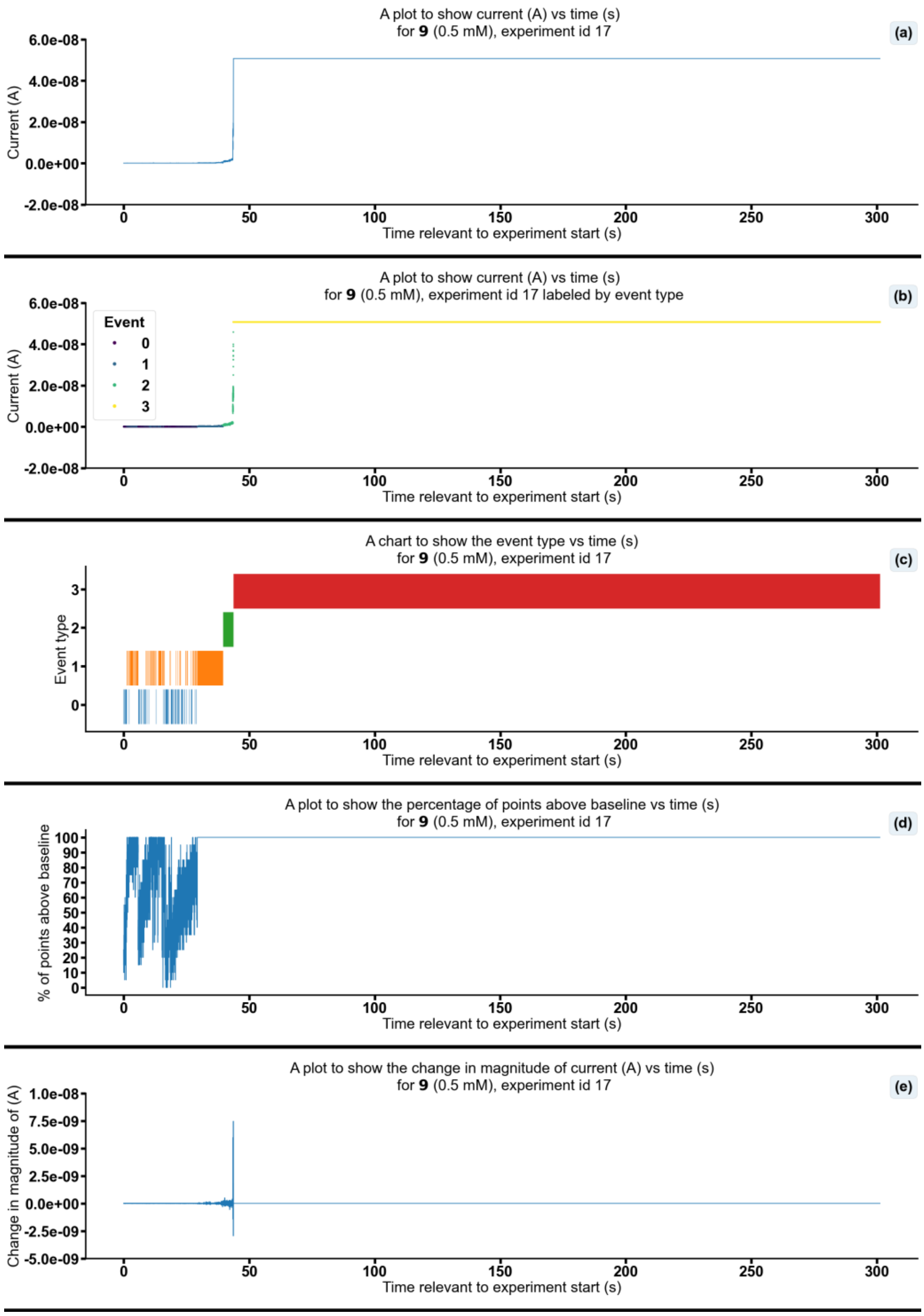

Figure S290- Summarising the data from the patch clamp experiment for **9** (0.5 mM), experiment id 17 at +100 mV. Events were categorised into the following types: 0: -∞ A ≤ Event 0 < 5.00e-11 A, 1: 5.00e-11 A ≤ Event 1 < 5.00e-10 A, 2: 5.00e-10 A ≤ Event 2 < 4.90e-08 A, 3: 4.90e-08 A ≤ Event 3 < ∞ A. Subfigure (d) shows the percentage of points found above the threshold 4.9e-11 (A) in a rolling look ahead window of 20 points. The threshold is the mean + standard deviation of the noise of the first 200 datapoints.

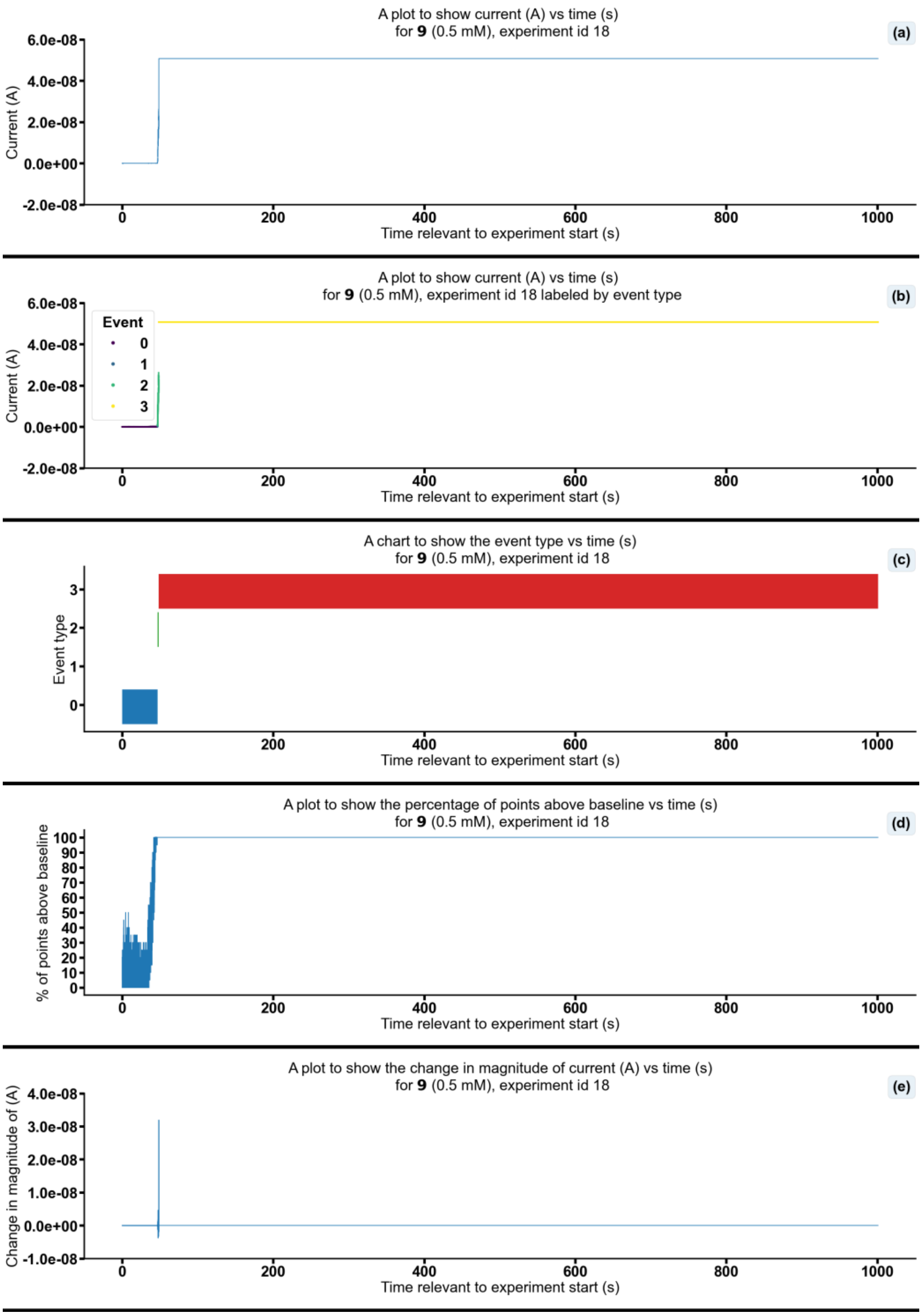

Figure S291 - Summarising the data from the patch clamp experiment for **9** (0.5 mM), experiment id 18 at +100 mV. Events were categorised into the following types: 0: -∞ A ≤ Event 0 < 5.00e-11 A, 1: 5.00e-11 A ≤ Event 1 < 5.00e-10 A, 2: 5.00e-10 A ≤ Event 2 < 4.90e-08 A, 3: 4.90e-08 A ≤ Event 3 < ∞ A. Subfigure (d) shows the percentage of points found above the threshold -1.0e-11 (A) in a rolling look ahead window of 20 points. The threshold is the mean + standard deviation of the noise of the first 200 datapoints.

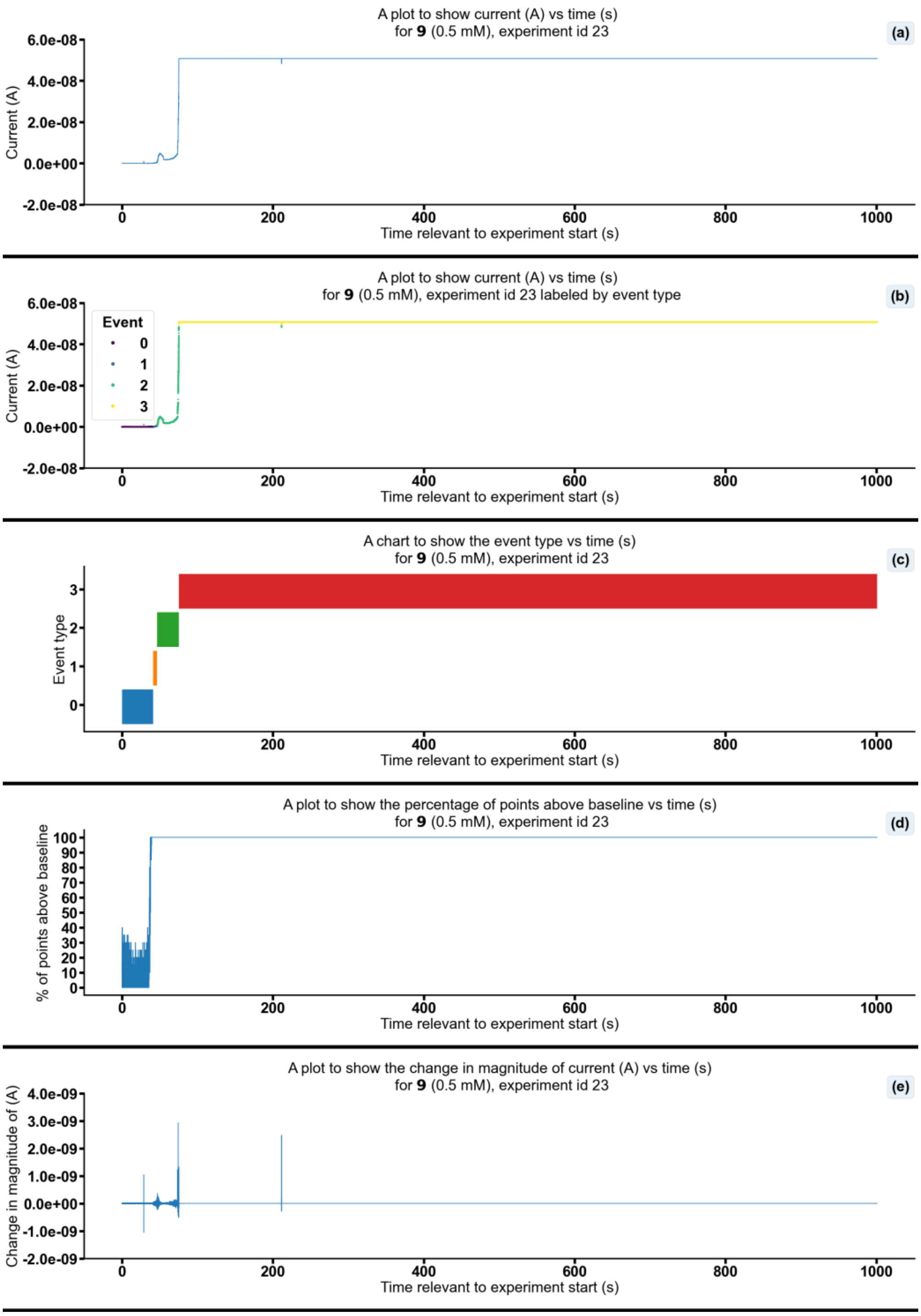

Figure S292 - Summarising the data from the patch clamp experiment for **9** (0.5 mM), experiment id 23 at +100 mV. Events were categorised into the following types: 0: -∞ A ≤ Event 0 < 5.00e-11 A, 1: 5.00e-11 A ≤ Event 1 < 5.00e-10 A, 2: 5.00e-10 A ≤ Event 2 < 4.90e-08 A, 3: 4.90e-08 A ≤ Event 3 < ∞ A. Subfigure (d) shows the percentage of points found above the threshold -8.6e-12 (A) in a rolling look ahead window of 20 points. The threshold is the mean + standard deviation of the noise of the first 200 datapoints.

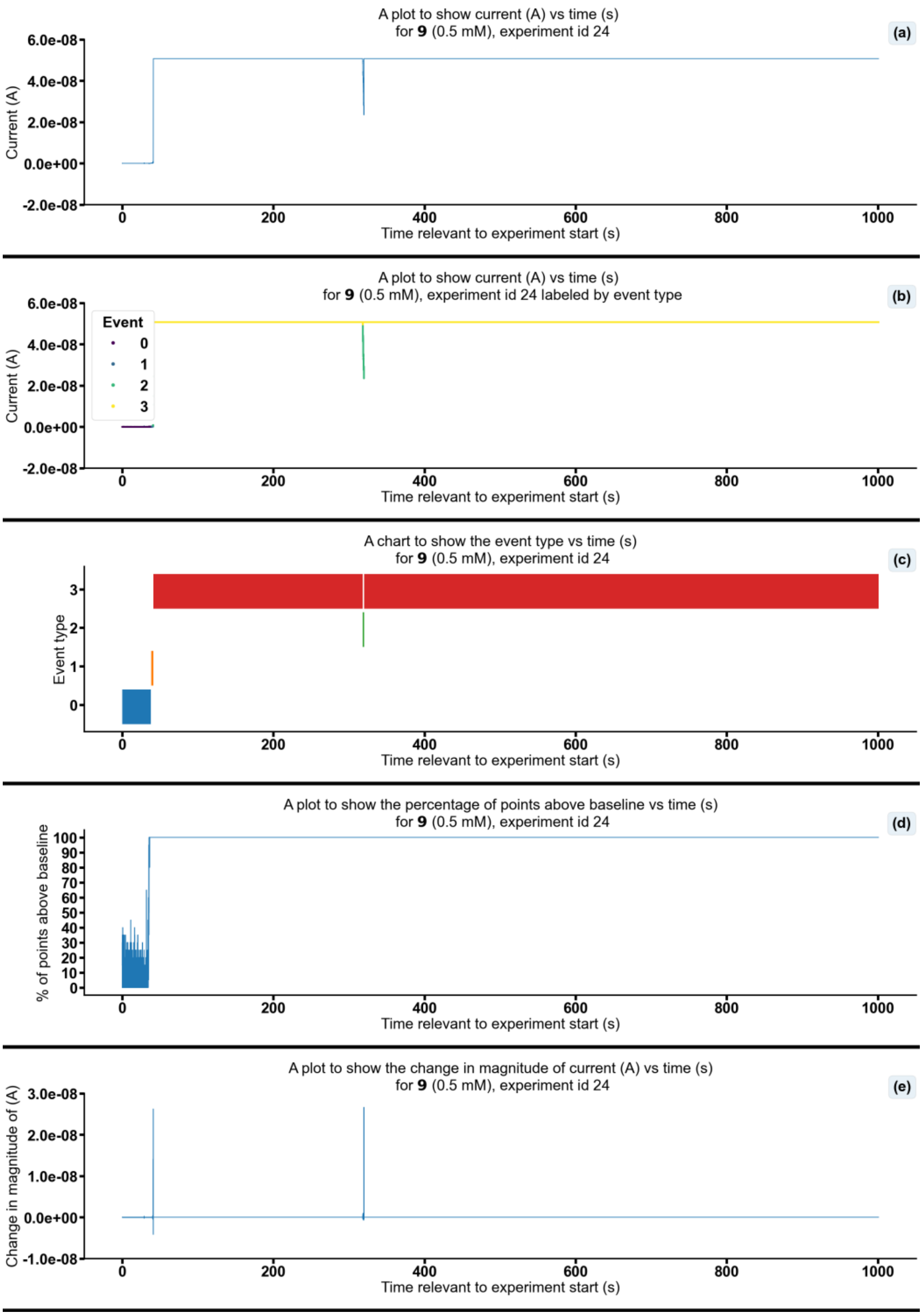

Figure S293 - Summarising the data from the patch clamp experiment for **9** (0.5 mM), experiment id 24 at +100 mV. Events were categorised into the following types: 0: -∞ A ≤ Event 0 < 5.00e-11 A, 1: 5.00e-11 A ≤ Event 1 < 5.00e-10 A, 2: 5.00e-10 A ≤ Event 2 < 4.90e-08 A, 3: 4.90e-08 A ≤ Event 3 < ∞ A. Subfigure (d) shows the percentage of points found above the threshold -9.0e-12 (A) in a rolling look ahead window of 20 points. The threshold is the mean + standard deviation of the noise of the first 200 datapoints.

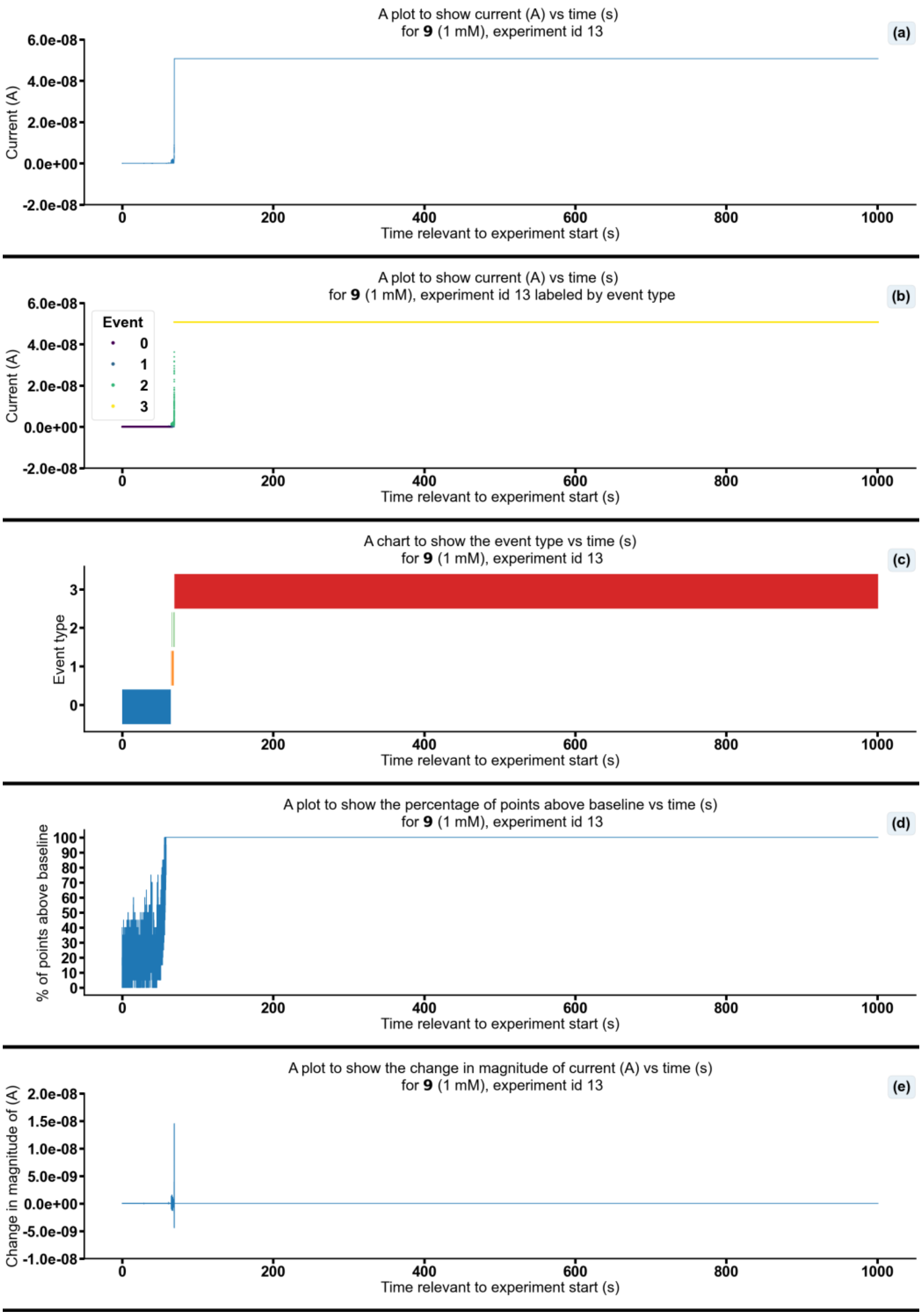

Figure S294 - Summarising the data from the patch clamp experiment for **9** (1 mM), experiment id 13 at +100 mV. Events were categorised into the following types: 0: -∞ A ≤ Event 0 < 5.00e-11 A, 1: 5.00e-11 A ≤ Event 1 < 5.00e-10 A, 2: 5.00e-10 A ≤ Event 2 < 4.90e-08 A, 3: 4.90e-08 A ≤ Event 3 < ∞ A. Subfigure (d) shows the percentage of points found above the threshold 6.9e-12 (A) in a rolling look ahead window of 20 points. The threshold is the mean + standard deviation of the noise of the first 200 datapoints.

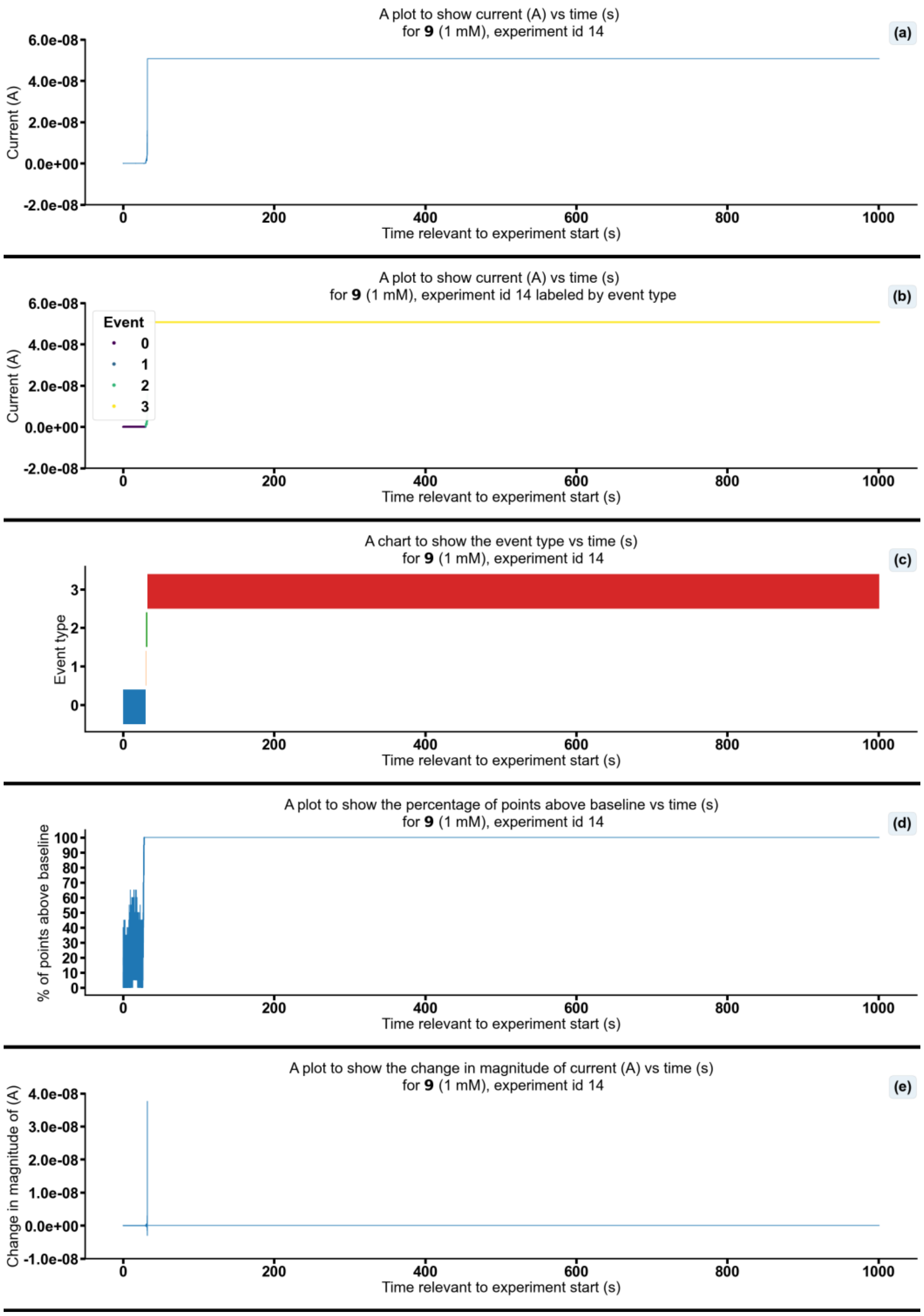

Figure S295 - Summarising the data from the patch clamp experiment for **9** (1 mM), experiment id 14 at +100 mV. Events were categorised into the following types: 0: -∞ A ≤ Event 0 < 5.00e-11 A, 1: 5.00e-11 A ≤ Event 1 < 5.00e-10 A, 2: 5.00e-10 A ≤ Event 2 < 4.90e-08 A, 3: 4.90e-08 A ≤ Event 3 < ∞ A. Subfigure (d) shows the percentage of points found above the threshold -6.6e-13 (A) in a rolling look ahead window of 20 points. The threshold is the mean + standard deviation of the noise of the first 200 datapoints.

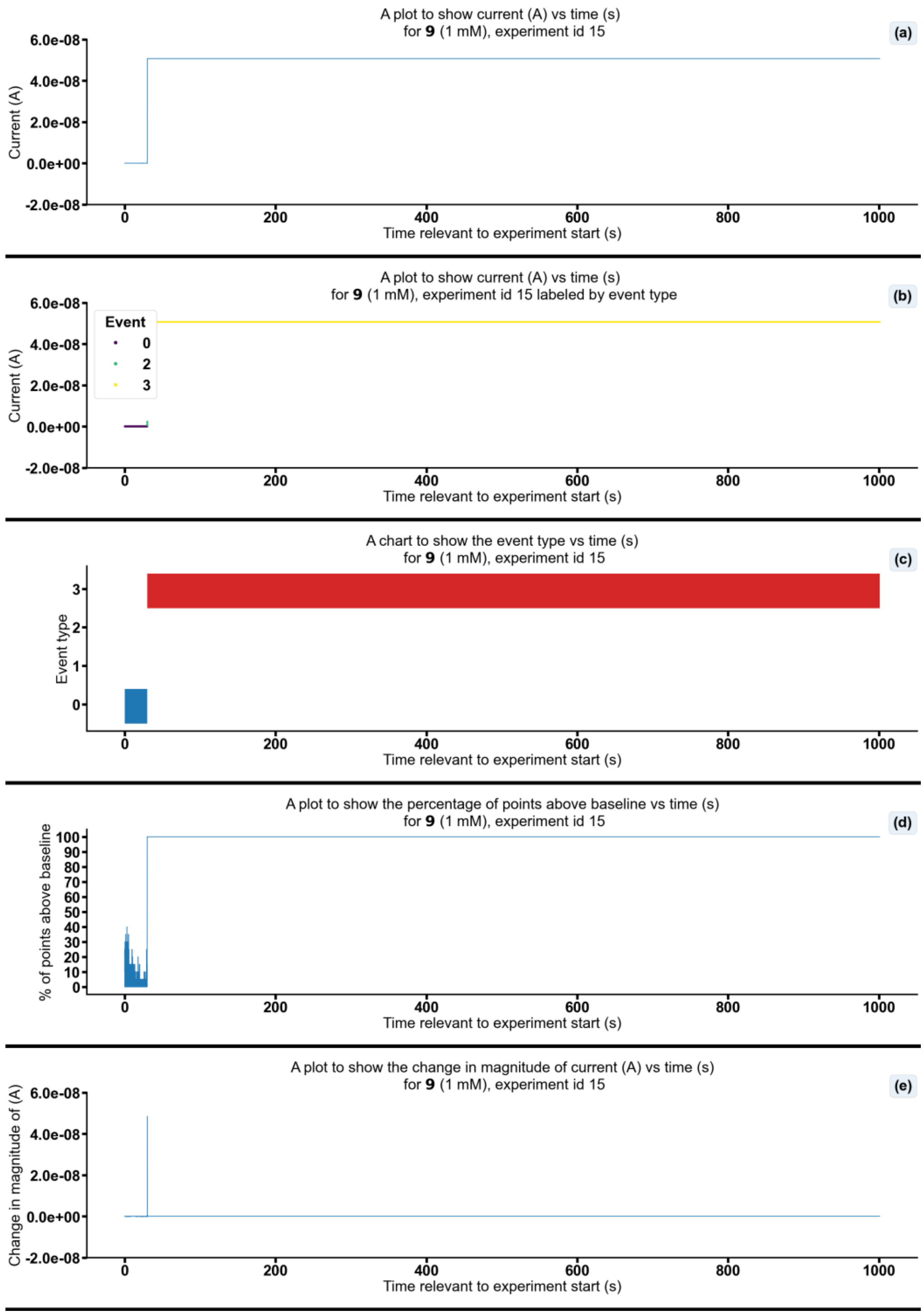

Figure S296 - Summarising the data from the patch clamp experiment for **9** (1 mM), experiment id 15 at +100 mV. Events were categorised into the following types: 0: -∞ A ≤ Event 0 < 5.00e-11 A, 1: 5.00e-11 A ≤ Event 1 < 5.00e-10 A, 2: 5.00e-10 A ≤ Event 2 < 4.90e-08 A, 3: 4.90e-08 A ≤ Event 3 < ∞ A. Subfigure (d) shows the percentage of points found above the threshold 2.8e-11 (A) in a rolling look ahead window of 20 points. The threshold is the mean + standard deviation of the noise of the first 200 datapoints.

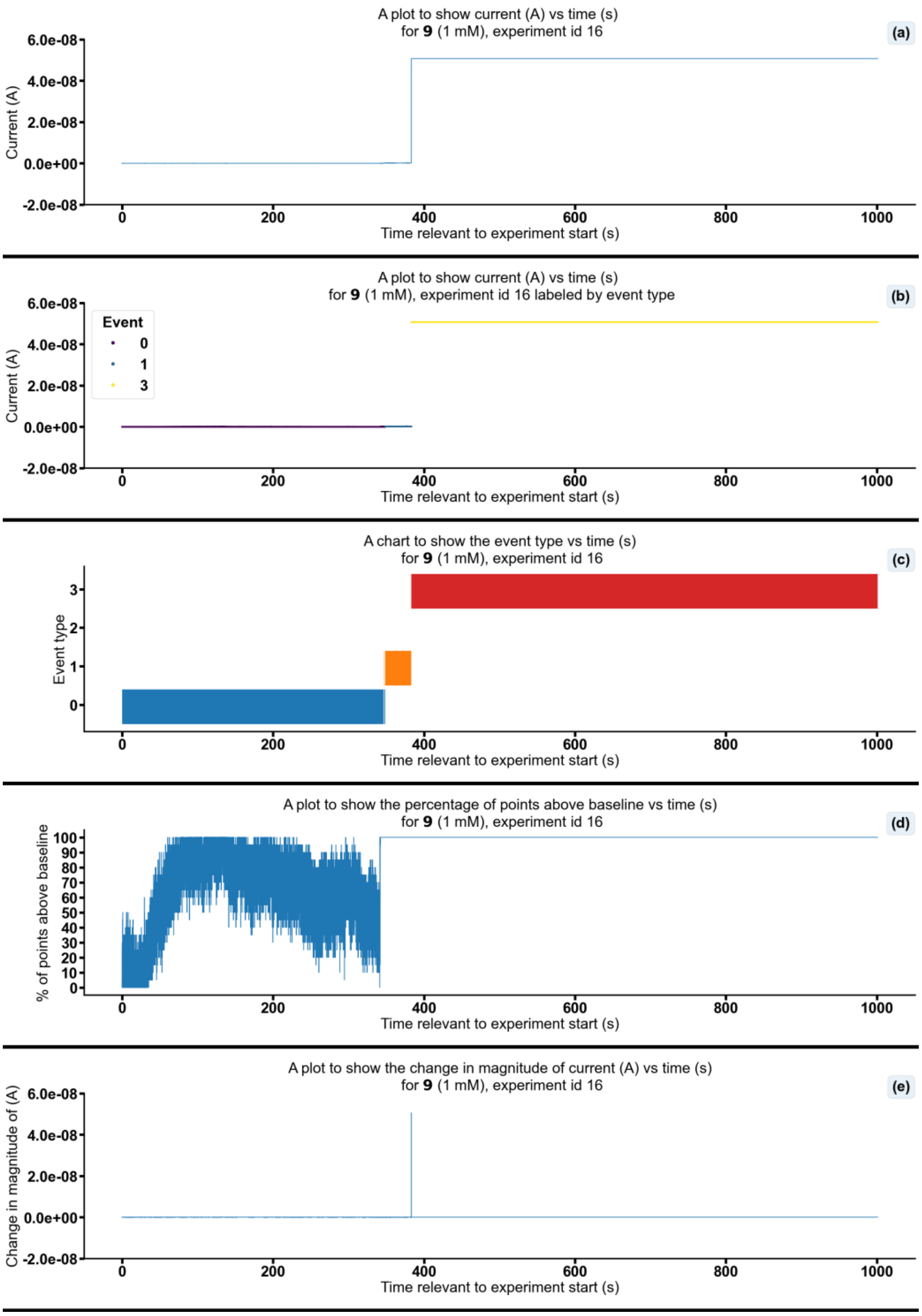

Figure S297 - Summarising the data from the patch clamp experiment for **9** (1 mM), experiment id 16 at +100 mV. Events were categorised into the following types: 0: -∞ A ≤ Event 0 < 5.00e-11 A, 1: 5.00e-11 A ≤ Event 1 < 5.00e-10 A, 2: 5.00e-10 A ≤ Event 2 < 4.90e-08 A, 3: 4.90e-08 A ≤ Event 3 < ∞ A. Subfigure (d) shows the percentage of points found above the threshold -1.7e-11 (A) in a rolling look ahead window of 20 points. The threshold is the mean + standard deviation of the noise of the first 200 datapoints.

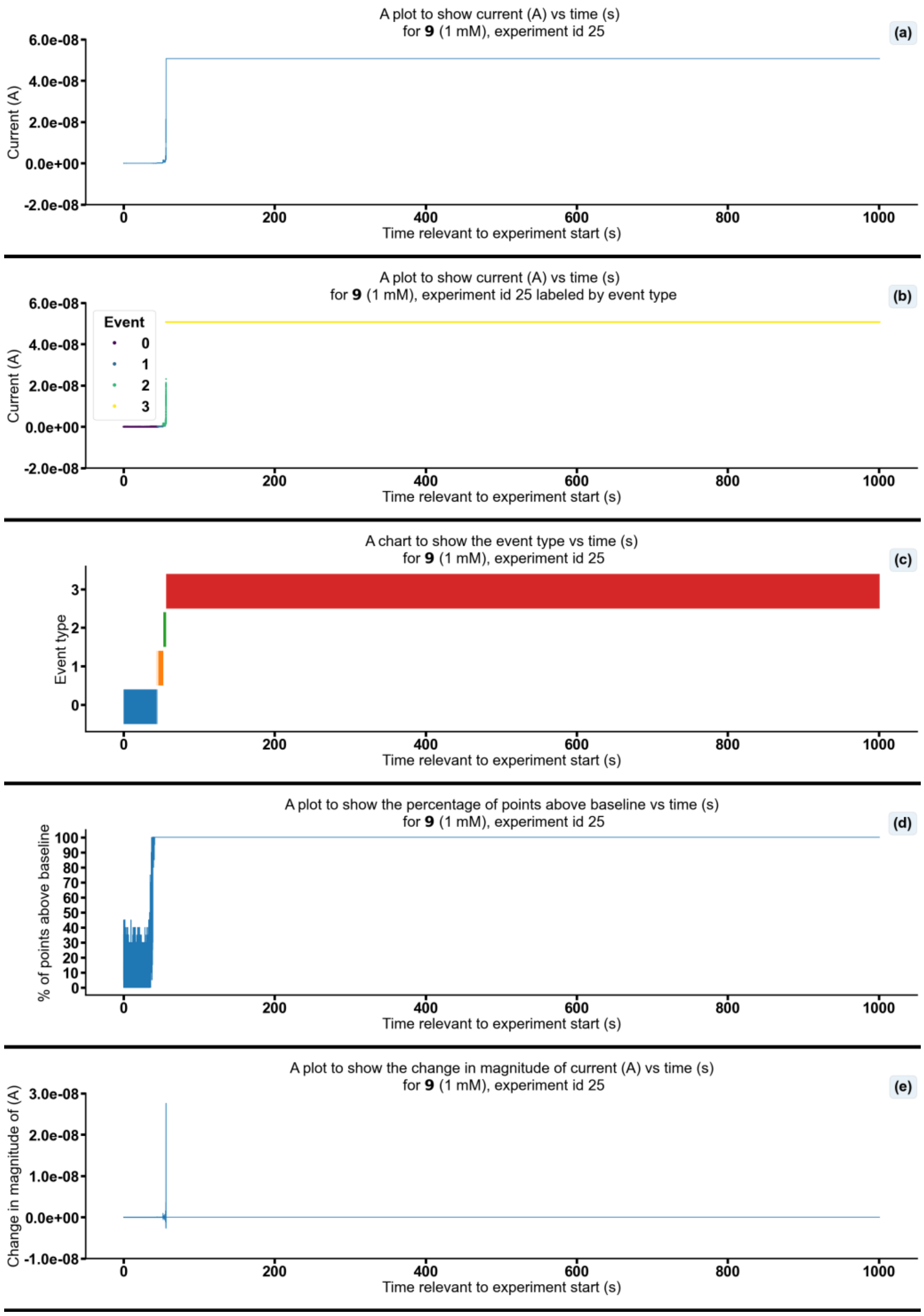

Figure S298 - Summarising the data from the patch clamp experiment for **9** (1 mM), experiment id 25 at +100 mV. Events were categorised into the following types: 0: -∞ A ≤ Event 0 < 5.00e-11 A, 1: 5.00e-11 A ≤ Event 1 < 5.00e-10 A, 2: 5.00e-10 A ≤ Event 2 < 4.90e-08 A, 3: 4.90e-08 A ≤ Event 3 < ∞ A. Subfigure (d) shows the percentage of points found above the threshold -7.9e-12 (A) in a rolling look ahead window of 20 points. The threshold is the mean + standard deviation of the noise of the first 200 datapoints.

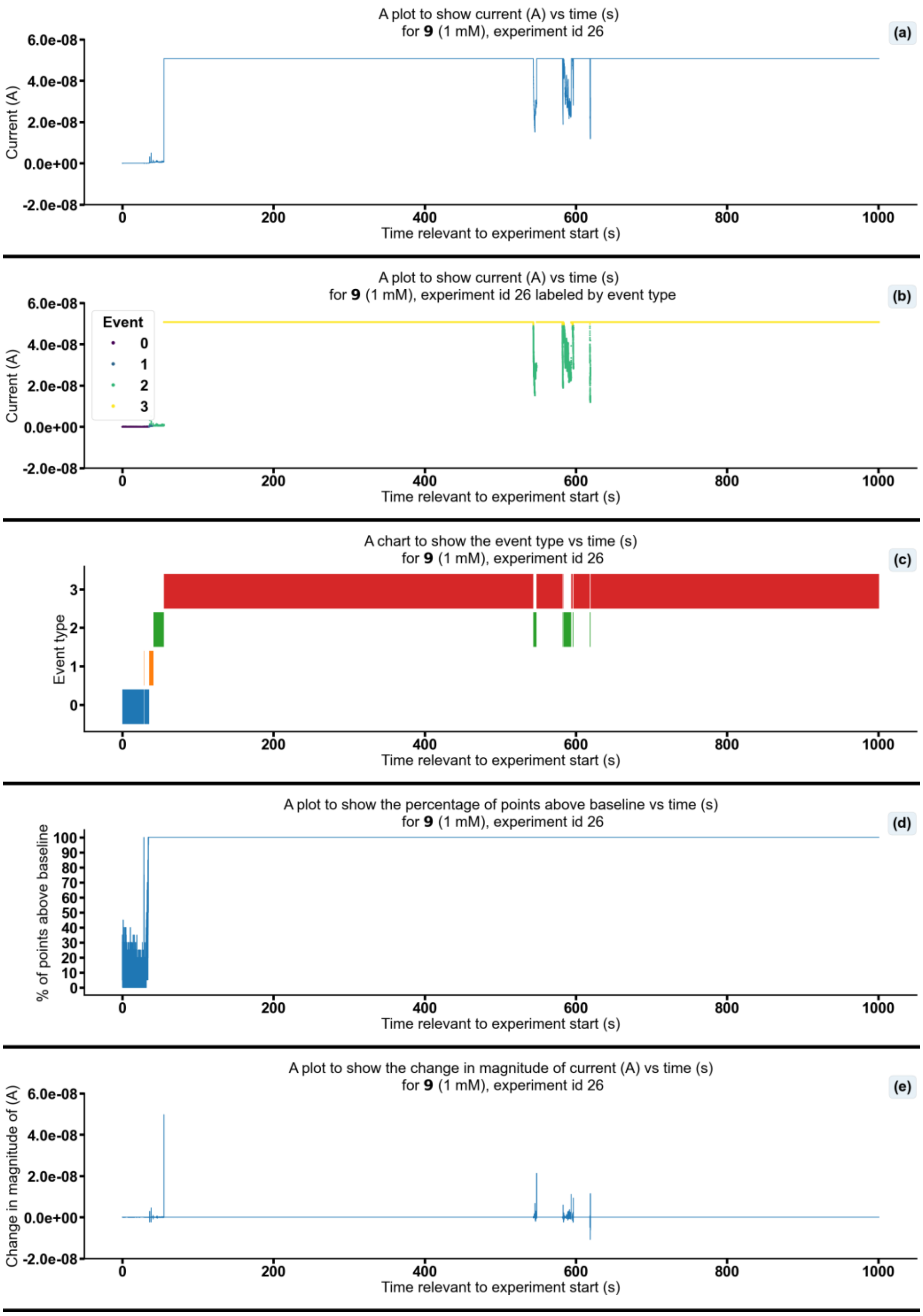

Figure S299 - Summarising the data from the patch clamp experiment for **9** (1 mM), experiment id 26 at +100 mV. Events were categorised into the following types: 0: -∞ A ≤ Event 0 < 5.00e-11 A, 1: 5.00e-11 A ≤ Event 1 < 5.00e-10 A, 2: 5.00e-10 A ≤ Event 2 < 4.90e-08 A, 3: 4.90e-08 A ≤ Event 3 < ∞ A. Subfigure (d) shows the percentage of points found above the threshold -7.1e-12 (A) in a rolling look ahead window of 20 points. The threshold is the mean + standard deviation of the noise of the first 200 datapoints.

Changing the concentration of **7** and **9** (1:1)

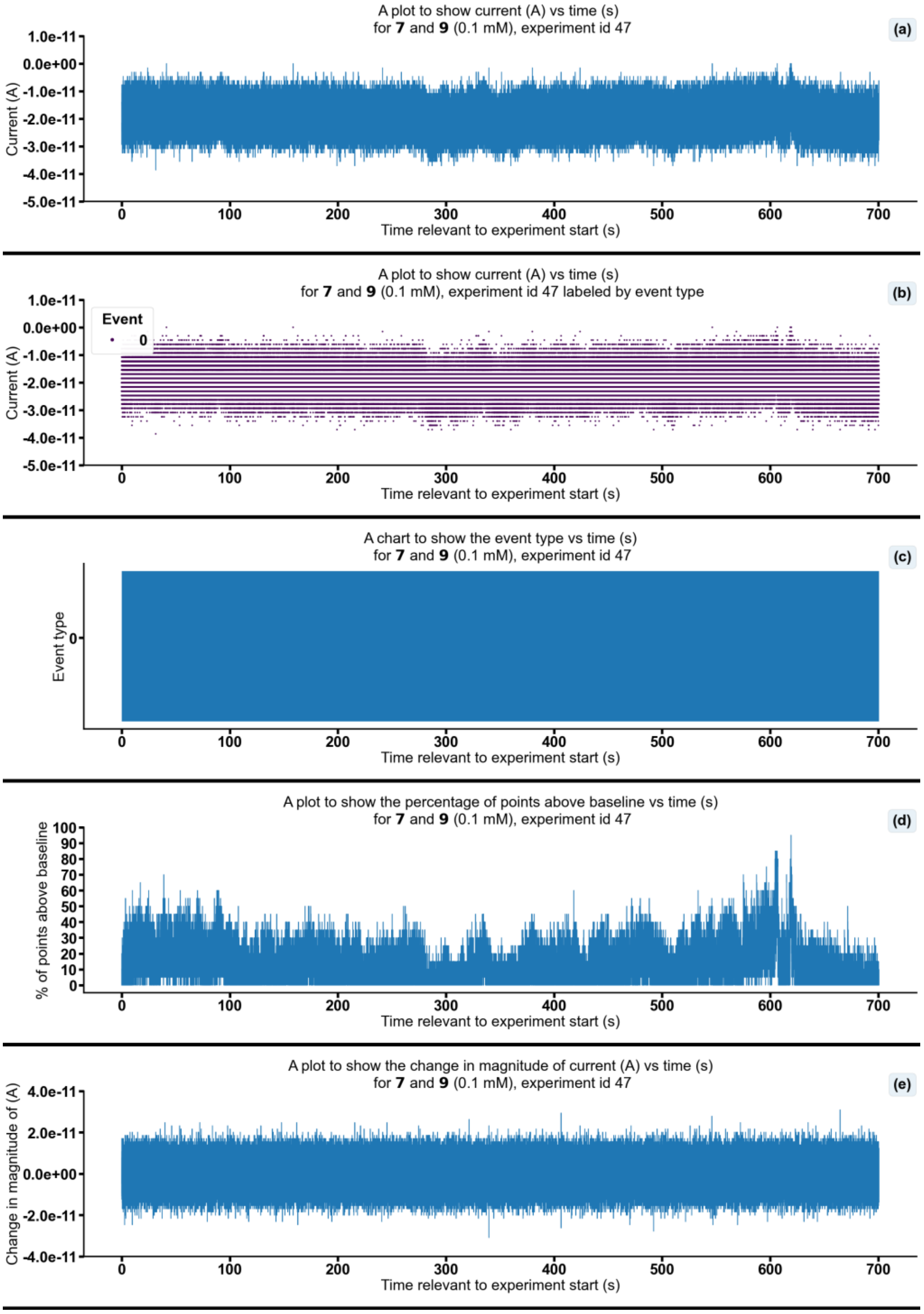

Figure S300 - Summarising the data from the patch clamp experiment for **7** and **9** (0.1 mM), experiment id 47 at +100 mV. Events were categorised into the following types: 0: -∞ A ≤ Event 0 < 5.00e-11 A, 1: 5.00e-11 A ≤ Event 1 < 5.00e-10 A, 2: 5.00e-10 A ≤ Event 2 < 4.90e-08 A, 3: 4.90e-08 A ≤ Event 3 < ∞ A. Subfigure (d) shows the percentage of points found above the threshold -1.5e-11 (A) in a rolling look ahead window of 20 points. The threshold is the mean + standard deviation of the noise of the first 200 datapoints.

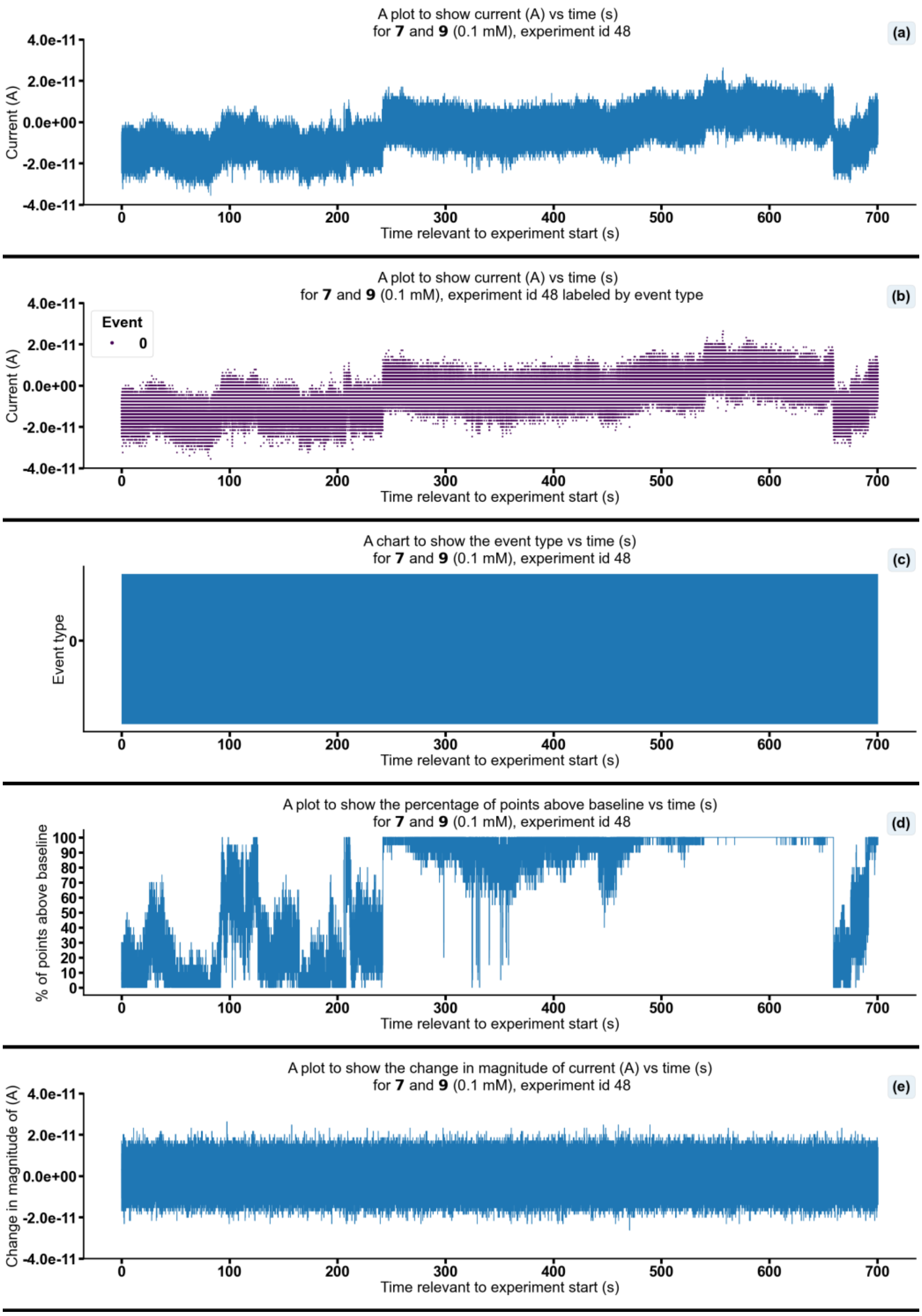

Figure S301 - Summarising the data from the patch clamp experiment for **7** and **9** (0.1 mM), experiment id 48 at +100 mV. Events were categorised into the following types: 0: -∞ A ≤ Event 0 < 5.00e-11 A, 1: 5.00e-11 A ≤ Event 1 < 5.00e-10 A, 2: 5.00e-10 A ≤ Event 2 < 4.90e-08 A, 3: 4.90e-08 A ≤ Event 3 < ∞ A. Subfigure (d) shows the percentage of points found above the threshold -1.1e-11 (A) in a rolling look ahead window of 20 points. The threshold is the mean + standard deviation of the noise of the first 200 datapoints.

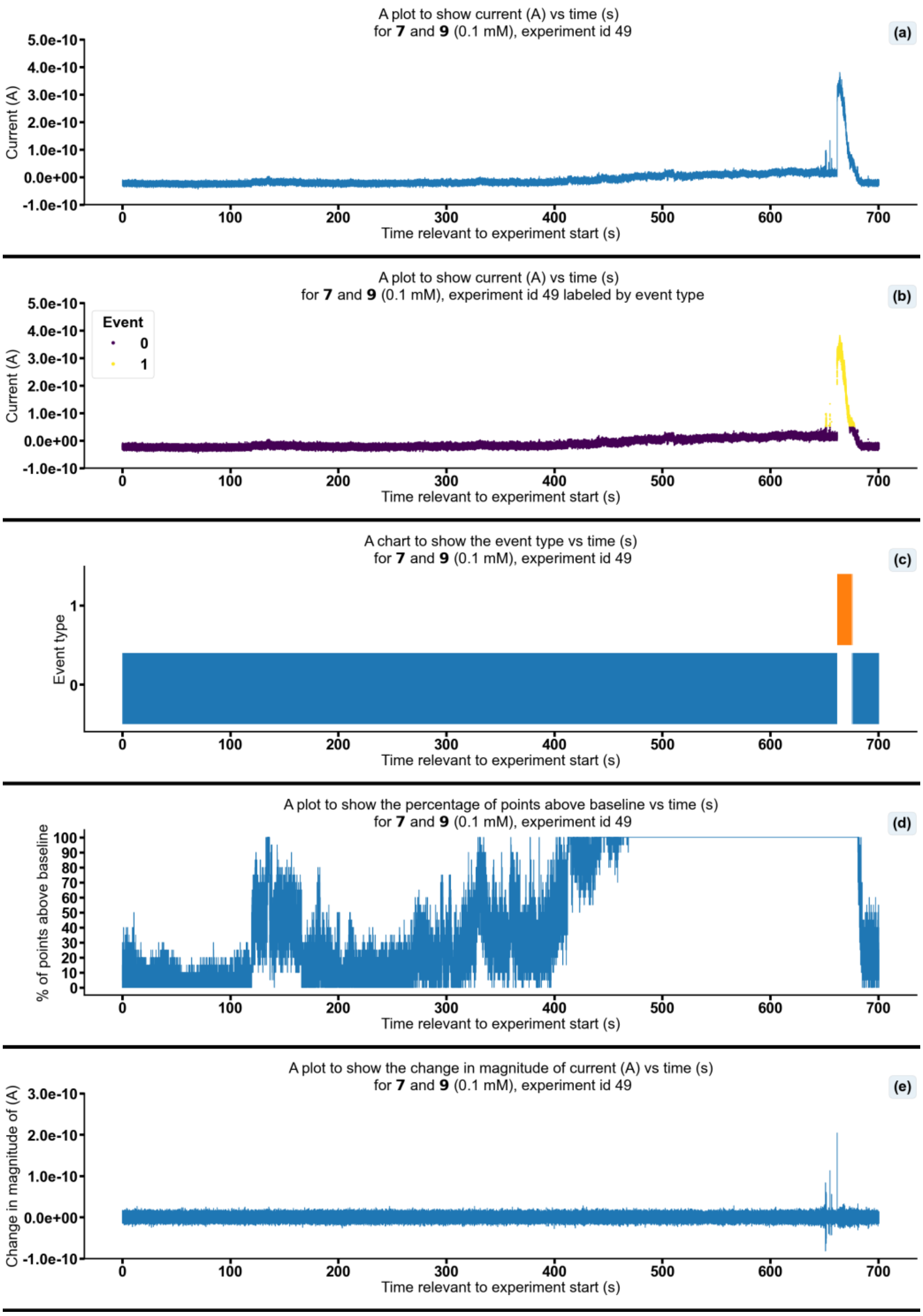

Figure S302 - Summarising the data from the patch clamp experiment for **7** and **9** (0.1 mM), experiment id 49 at +100 mV. Events were categorised into the following types: 0: -∞ A ≤ Event 0 < 5.00e-11 A, 1: 5.00e-11 A ≤ Event 1 < 5.00e-10 A, 2: 5.00e-10 A ≤ Event 2 < 4.90e-08 A, 3: 4.90e-08 A ≤ Event 3 < ∞ A. Subfigure (d) shows the percentage of points found above the threshold -1.9e-11 (A) in a rolling look ahead window of 20 points. The threshold is the mean + standard deviation of the noise of the first 200 datapoints.

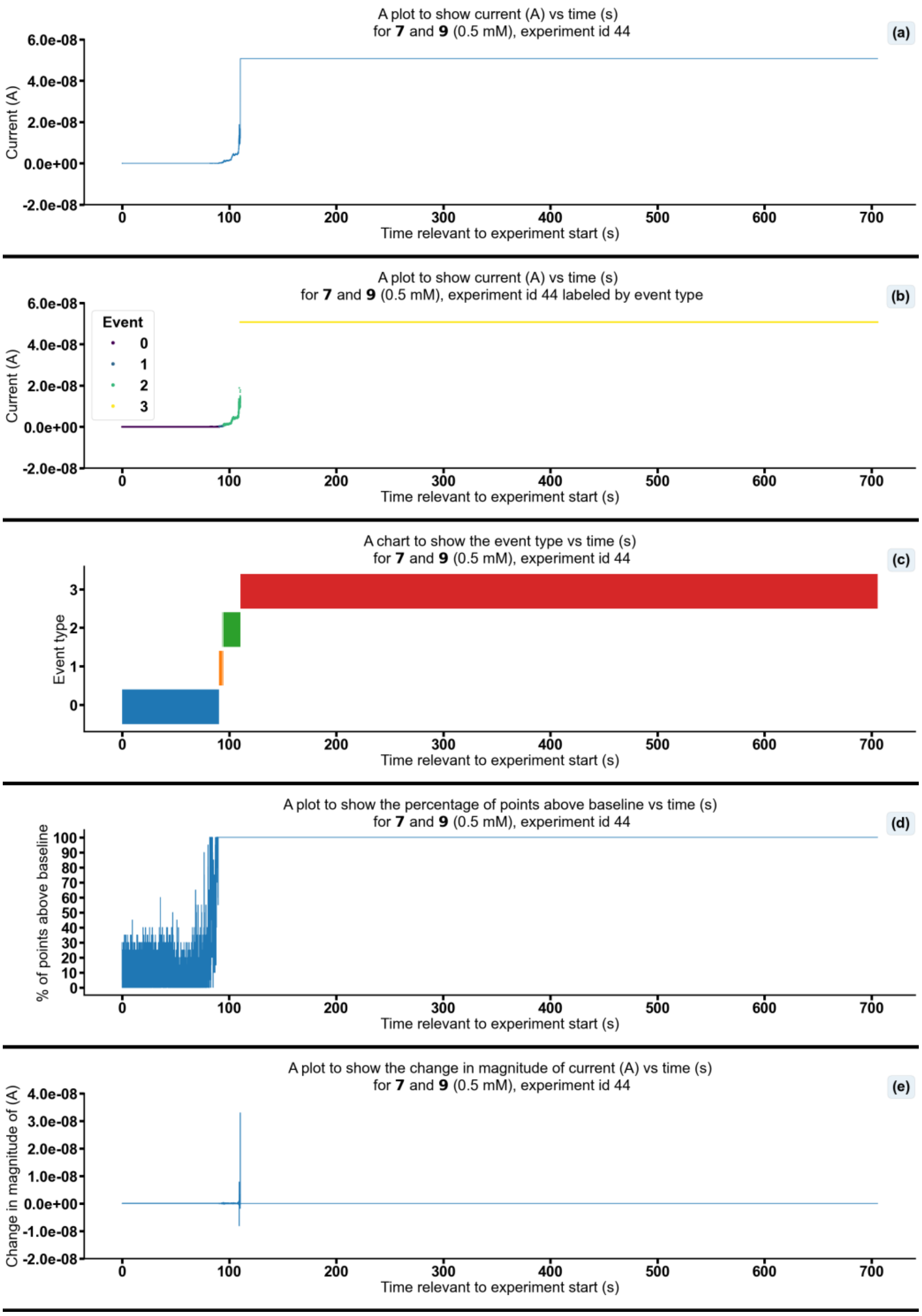

Figure S303 - Summarising the data from the patch clamp experiment for **7** and **9** (0.5 mM), experiment id 44 at +100 mV. Events were categorised into the following types: 0: -∞ A ≤ Event 0 < 5.00e-11 A, 1: 5.00e-11 A ≤ Event 1 < 5.00e-10 A, 2: 5.00e-10 A ≤ Event 2 < 4.90e-08 A, 3: 4.90e-08 A ≤ Event 3 < ∞ A. Subfigure (d) shows the percentage of points found above the threshold -1.8e-11 (A) in a rolling look ahead window of 20 points. The threshold is the mean + standard deviation of the noise of the first 200 datapoints.

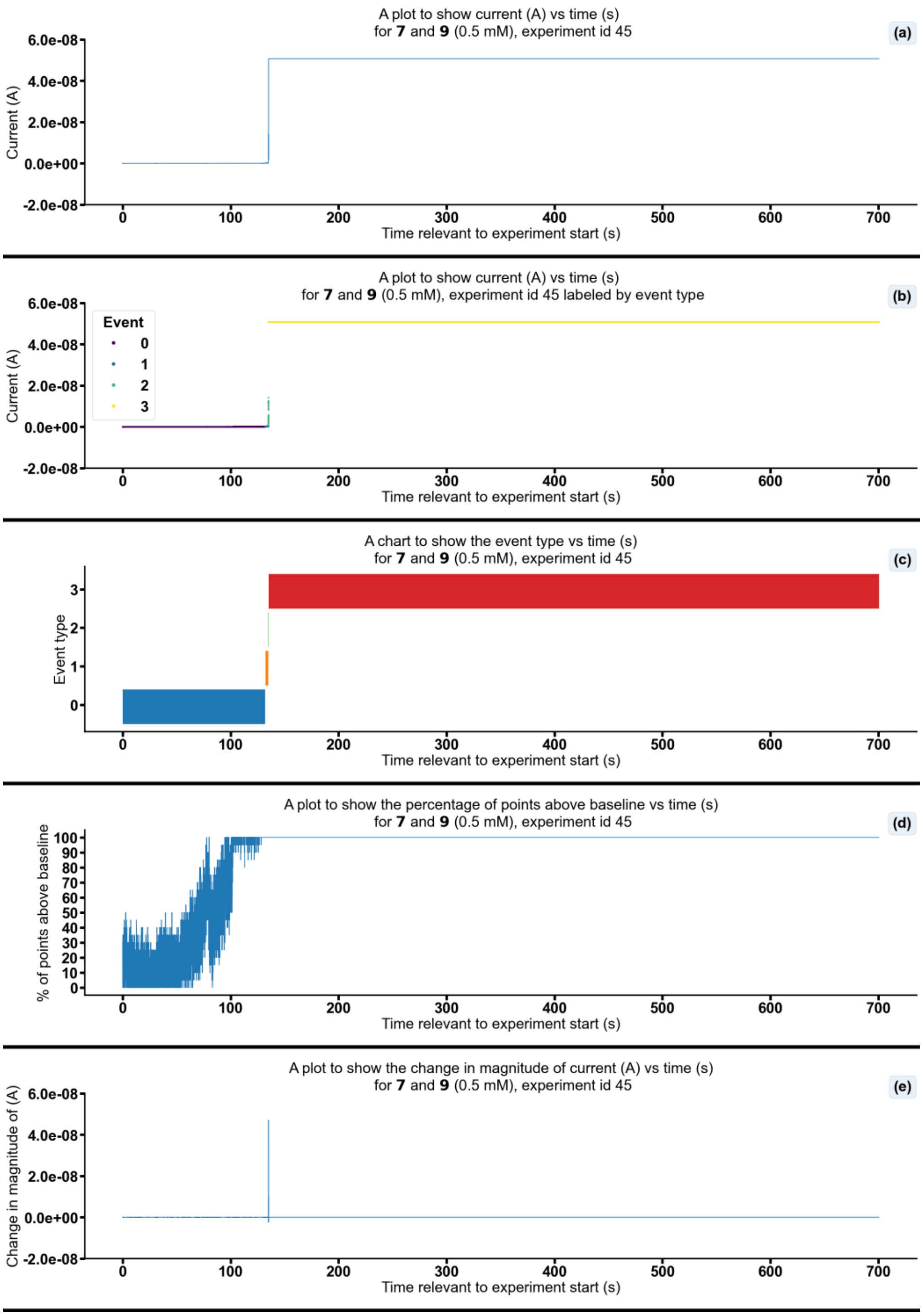

Figure S304 - Summarising the data from the patch clamp experiment for **7** and **9** (0.5 mM), experiment id 45 at +100 mV. Events were categorised into the following types: 0: -∞ A ≤ Event 0 < 5.00e-11 A, 1: 5.00e-11 A ≤ Event 1 < 5.00e-10 A, 2: 5.00e-10 A ≤ Event 2 < 4.90e-08 A, 3: 4.90e-08 A ≤ Event 3 < ∞ A. Subfigure (d) shows the percentage of points found above the threshold -1.8e-11 (A) in a rolling look ahead window of 20 points. The threshold is the mean + standard deviation of the noise of the first 200 datapoints.

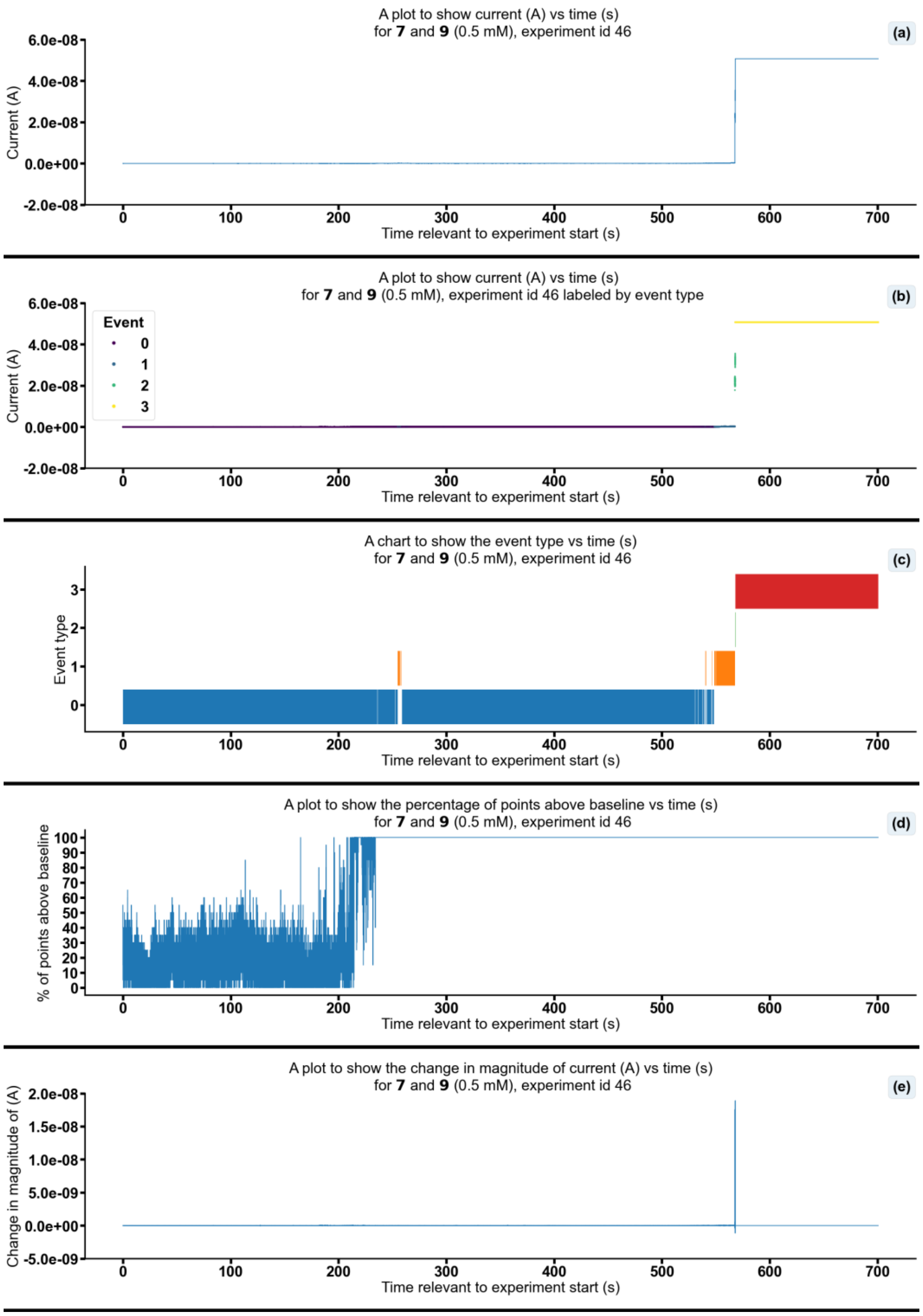

Figure S305 - Summarising the data from the patch clamp experiment for **7** and **9** (0.5 mM), experiment id 46 at +100 mV. Events were categorised into the following types: 0: -∞ A ≤ Event 0 < 5.00e-11 A, 1: 5.00e-11 A ≤ Event 1 < 5.00e-10 A, 2: 5.00e-10 A ≤ Event 2 < 4.90e-08 A, 3: 4.90e-08 A ≤ Event 3 < ∞ A. Subfigure (d) shows the percentage of points found above the threshold -1.4e-11 (A) in a rolling look ahead window of 20 points. The threshold is the mean + standard deviation of the noise of the first 200 datapoints.

Control experiments

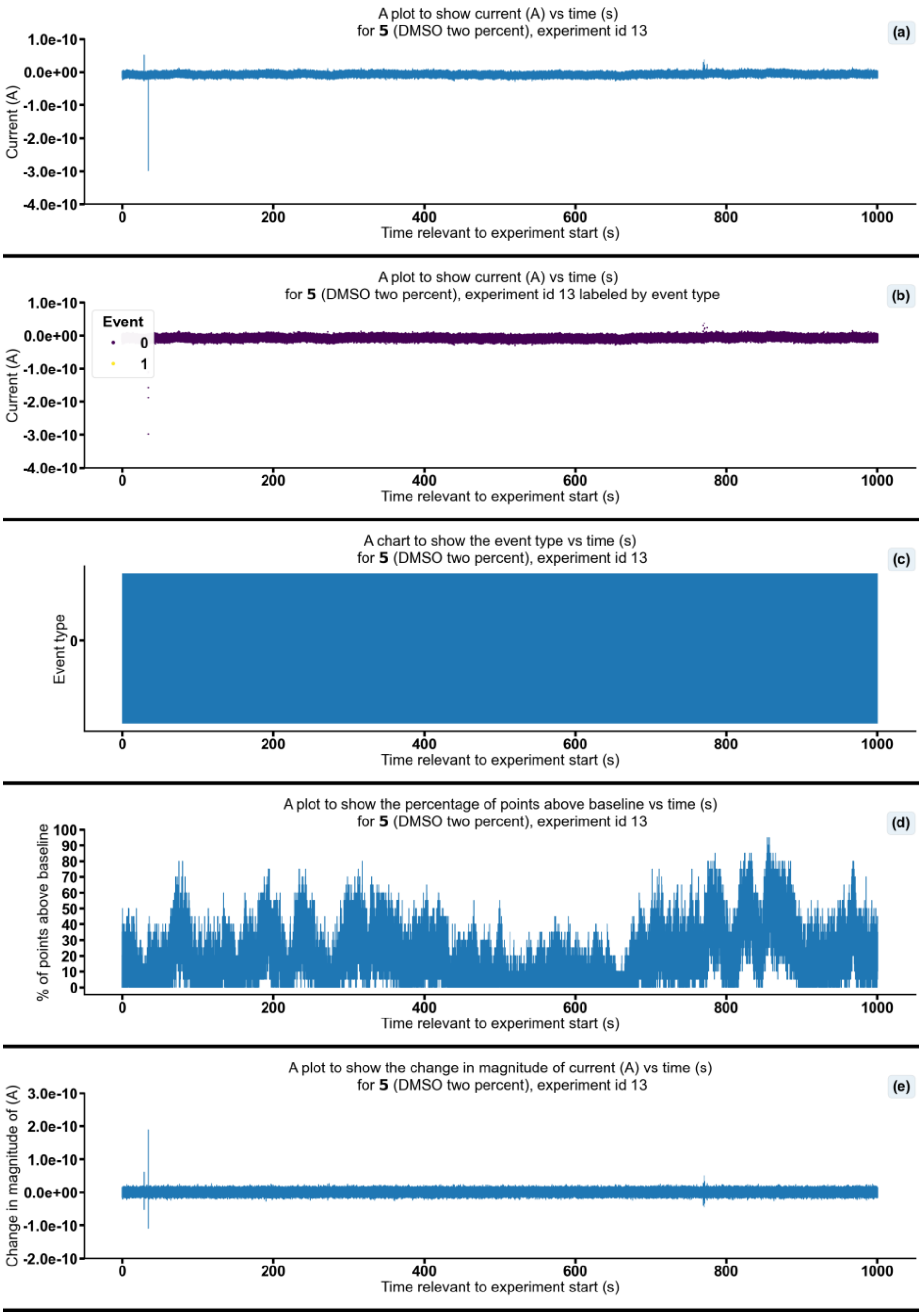

Figure S306 - Summarising the data from the patch clamp experiment for the DMSO two percent control (no SSA was present during the running of this experiment), experiment id 13 at +100 mV. Events were categorised into the following types: 0: -∞ A ≤ Event 0 < 5.00e-11 A, 1: 5.00e-11 A ≤ Event 1 < 5.00e-10 A, 2: 5.00e-10 A ≤ Event 2 < 4.90e-08 A, 3: 4.90e-08 A ≤ Event 3 < ∞ A. Subfigure (d) shows the percentage of points found above the threshold -4.4e-12 (A) in a rolling look ahead window of 20 points. The threshold is the mean + standard deviation of the noise of the first 200 datapoints.

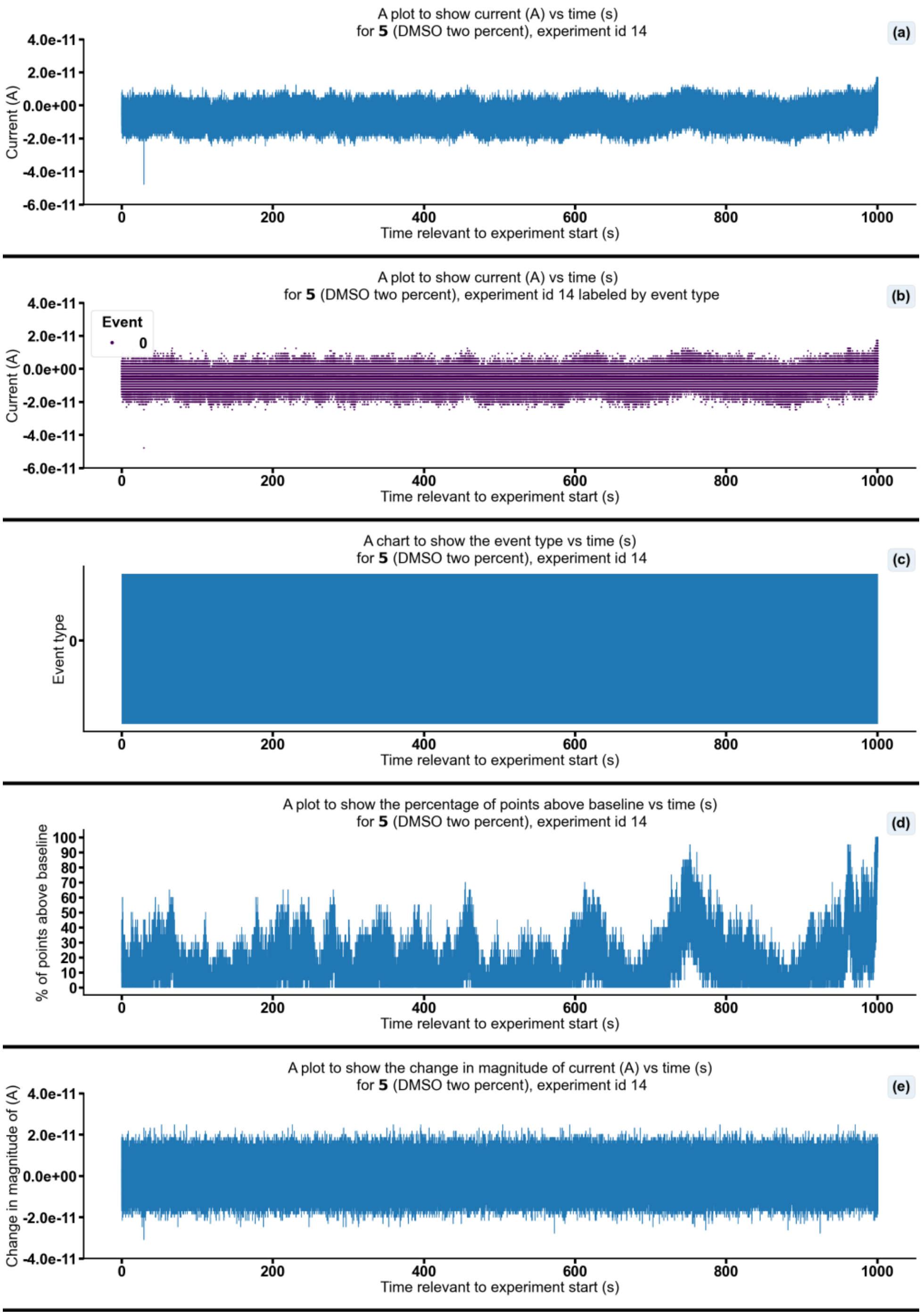

Figure S307 - Summarising the data from the patch clamp experiment for the DMSO two percent control (no SSA was present during the running of this experiment), experiment id 14 at +100 mV. Events were categorised into the following types: 0: -∞ A ≤ Event 0 < 5.00e-11 A, 1: 5.00e-11 A ≤ Event 1 < 5.00e-10 A, 2: 5.00e-10 A ≤ Event 2 < 4.90e-08 A, 3: 4.90e-08 A ≤ Event 3 < ∞ A. Subfigure (d) shows the percentage of points found above the threshold -1.9e-12 (A) in a rolling look ahead window of 20 points. The threshold is the mean + standard deviation of the noise of the first 200 datapoints.

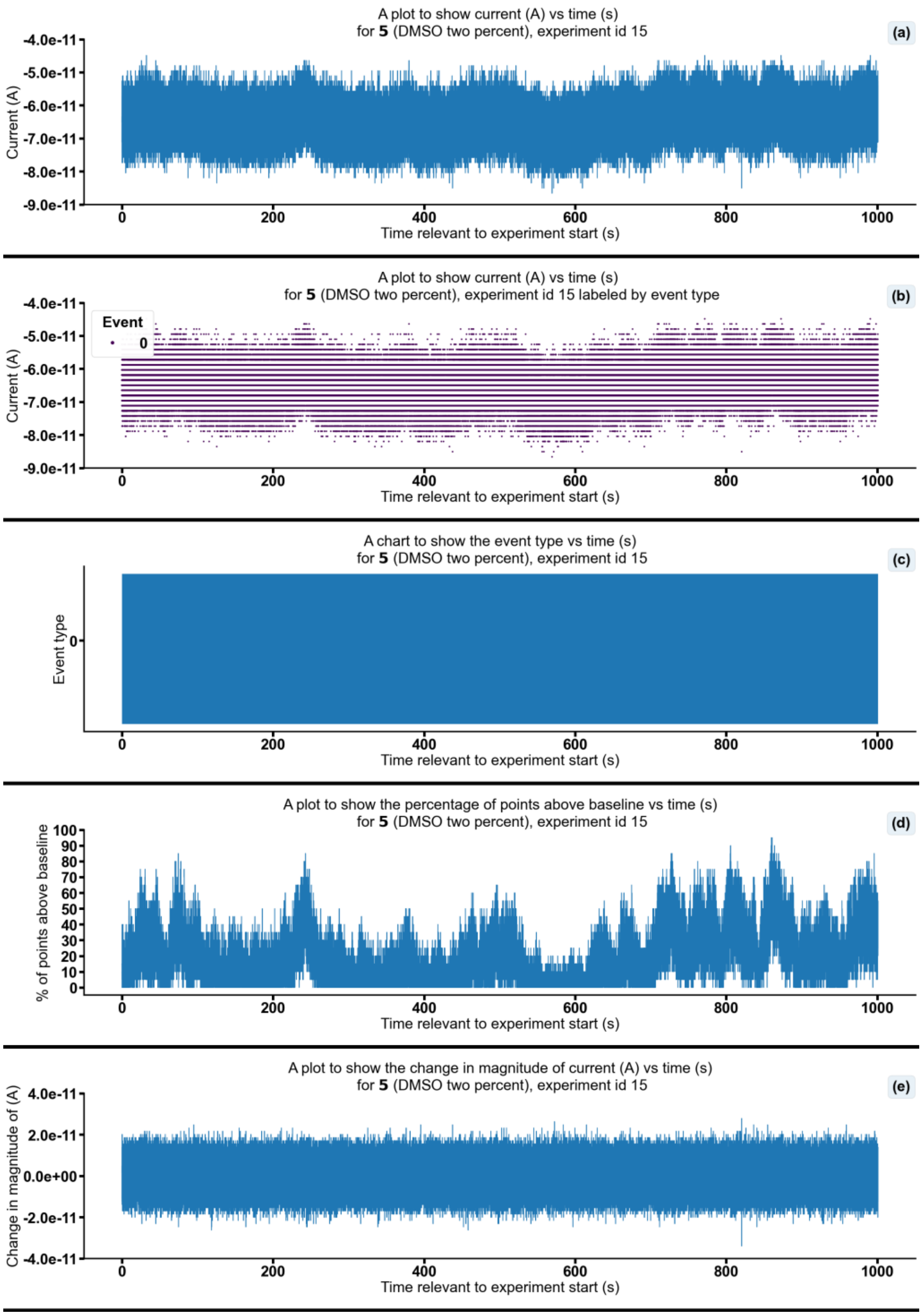

Figure S308 - Summarising the data from the patch clamp experiment for the DMSO two percent control (no SSA was present during the running of this experiment), experiment id 15 at +100 mV. Events were categorised into the following types: 0: -∞ A ≤ Event 0 < 5.00e-11 A, 1: 5.00e-11 A ≤ Event 1 < 5.00e-10 A, 2: 5.00e-10 A ≤ Event 2 < 4.90e-08 A, 3: 4.90e-08 A ≤ Event 3 < ∞ A. Subfigure (d) shows the percentage of points found above the threshold -6.1e-11 (A) in a rolling look ahead window of 20 points. The threshold is the mean + standard deviation of the noise of the first 200 datapoints.

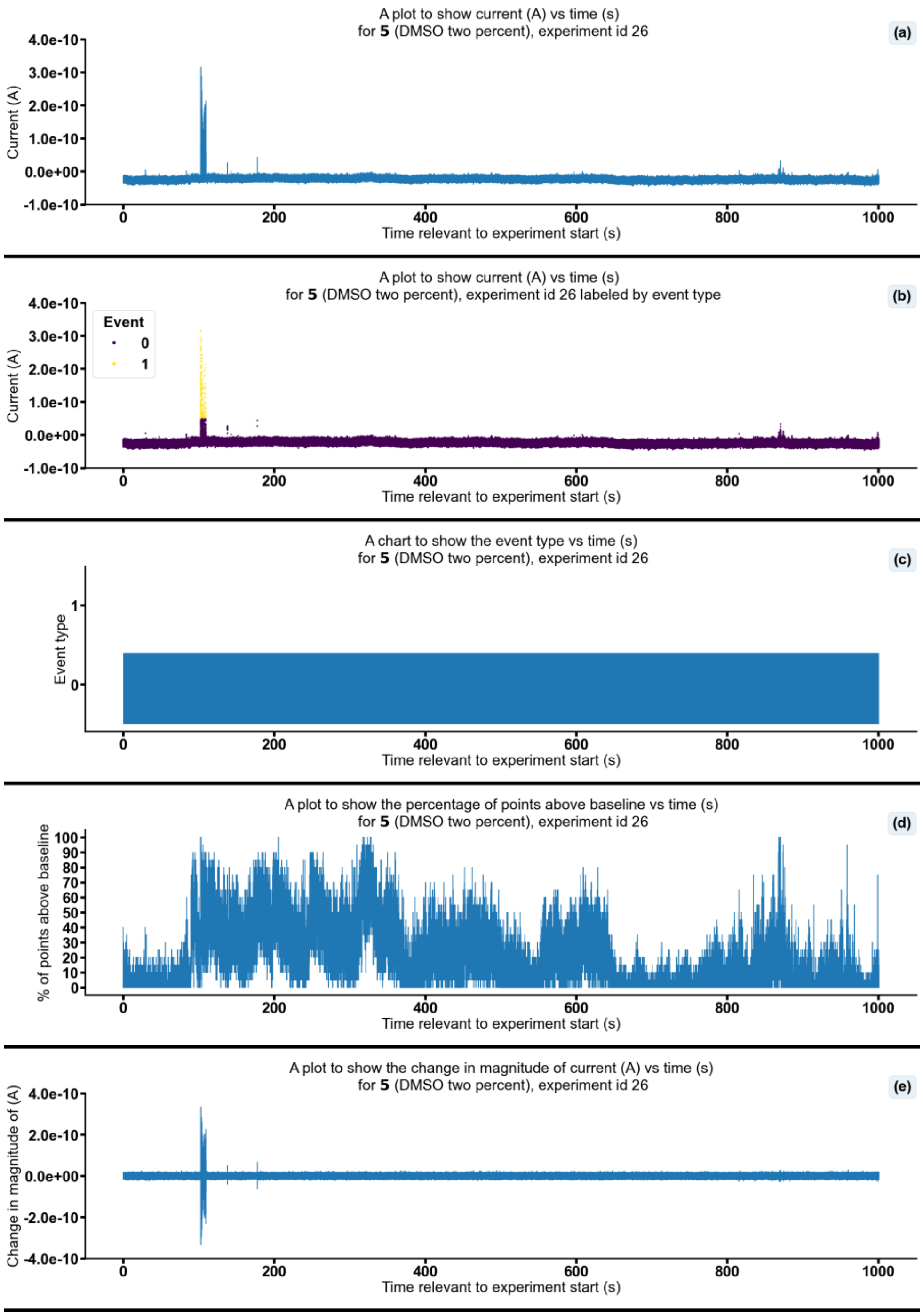

Figure S309 - Summarising the data from the patch clamp experiment for the DMSO two percent control (no SSA was present during the running of this experiment), experiment id 26 at +100 mV. Events were categorised into the following types: 0: -∞ A ≤ Event 0 < 5.00e-11 A, 1: 5.00e-11 A ≤ Event 1 < 5.00e-10 A, 2: 5.00e-10 A ≤ Event 2 < 4.90e-08 A, 3: 4.90e-08 A ≤ Event 3 < ∞ A. Subfigure (d) shows the percentage of points found above the threshold -2.0e-11 (A) in a rolling look ahead window of 20 points. The threshold is the mean + standard deviation of the noise of the first 200 datapoints.

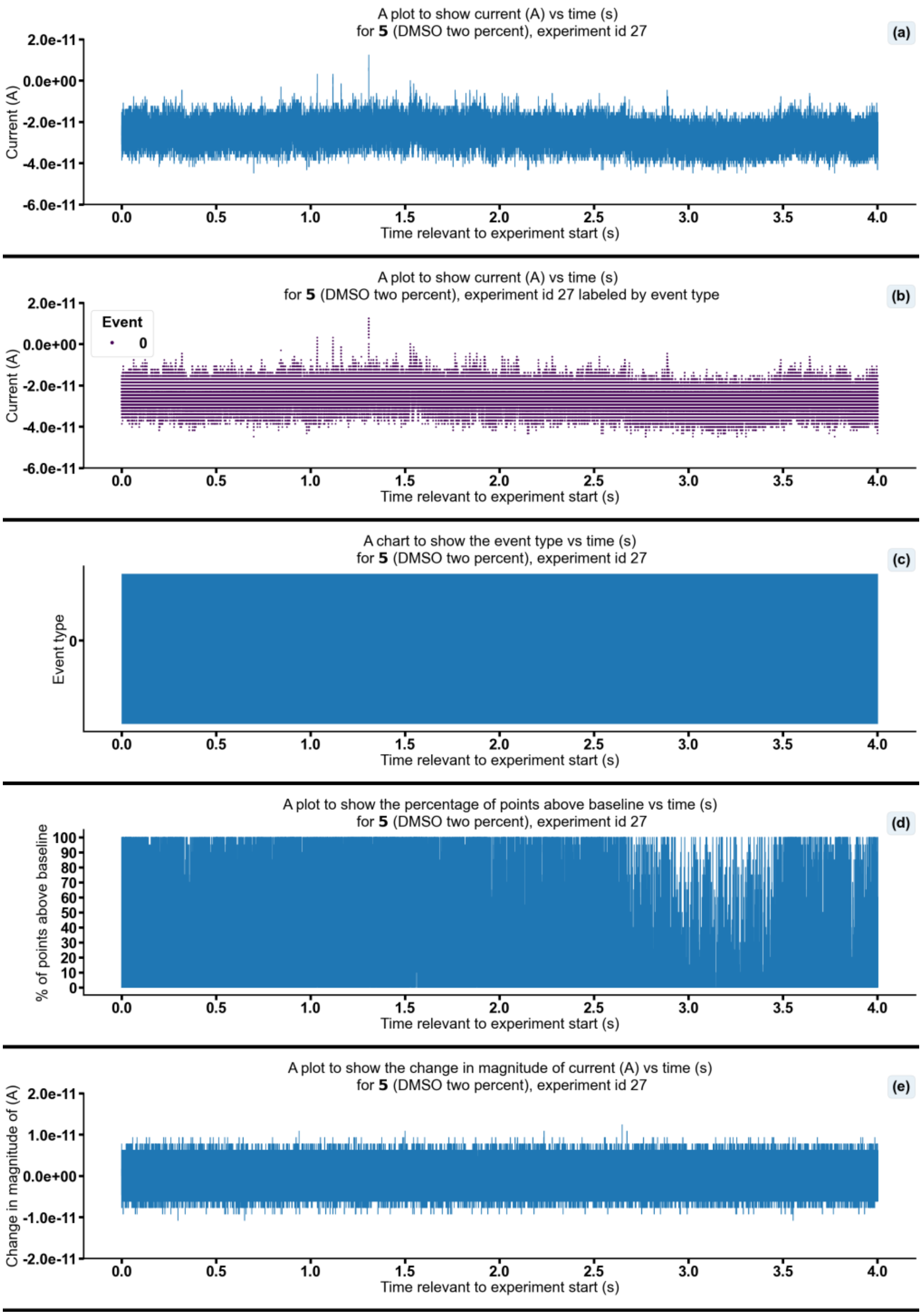

Figure S310 - Summarising the data from the patch clamp experiment for the DMSO two percent control (no SSA was present during the running of this experiment), experiment id 27 at +100 mV. Events were categorised into the following types: 0: -∞ A ≤ Event 0 < 5.00e-11 A, 1: 5.00e-11 A ≤ Event 1 < 5.00e-10 A, 2: 5.00e-10 A ≤ Event 2 < 4.90e-08 A, 3: 4.90e-08 A ≤ Event 3 < ∞ A. Subfigure (d) shows the percentage of points found above the threshold -2.2e-11 (A) in a rolling look ahead window of 20 points. The threshold is the mean + standard deviation of the noise of the first 200 datapoints.

Magnified portions of patch clamp data sets

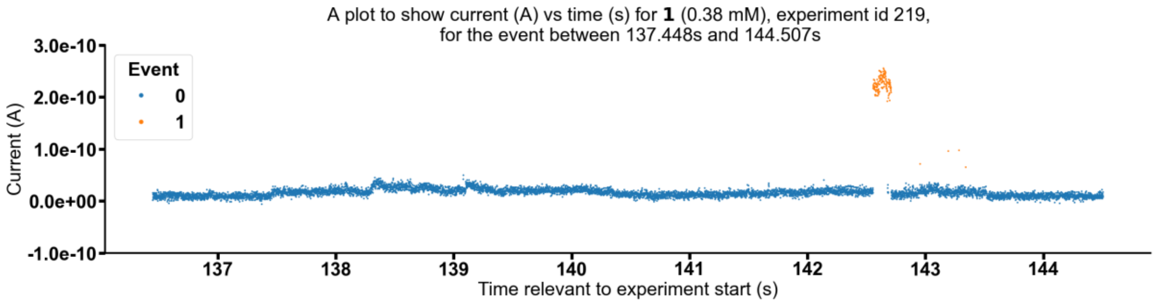

Figure S311 - Graph showing current (A) vs time (s) for **1** (0.38 mM), experiment id 219 at +100 mV, for the event between 137.448s and 144.507s.

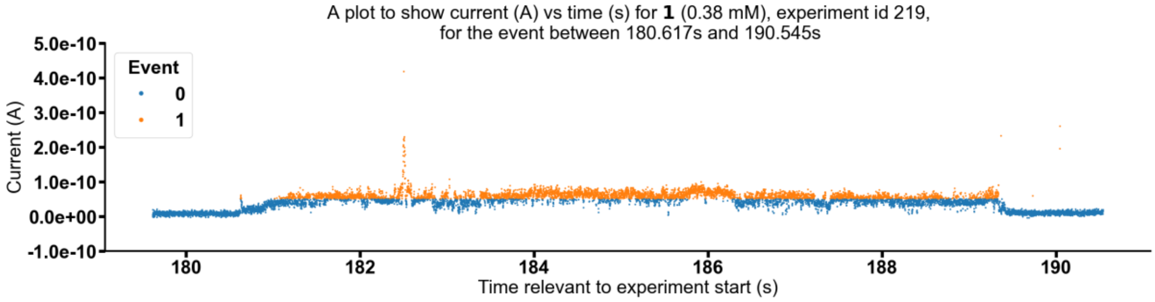

Figure S312 - Graph showing current (A) vs time (s) for **1** (0.38 mM), experiment id 219 at +100 mV, for the event between 180.617s and 190.545s.

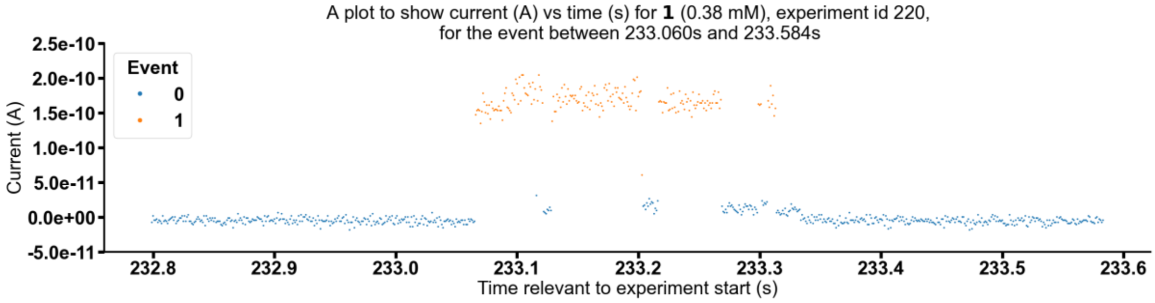

Figure S313 - Graph showing current (A) vs time (s) for **1** (0.38 mM), experiment id 220 at +100 mV, for the event between 233.060s and 233.584s.

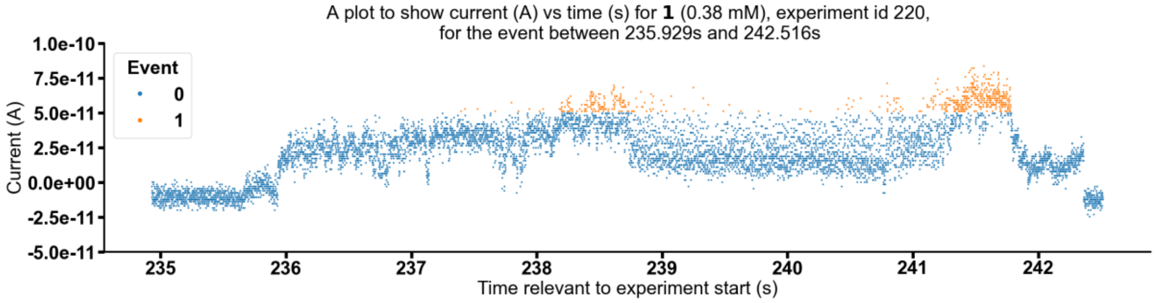

Figure S314 - Graph showing current (A) vs time (s) for **1** (0.38 mM), experiment id 220 at +100 mV, for the event between 235.929s and 242.516s.

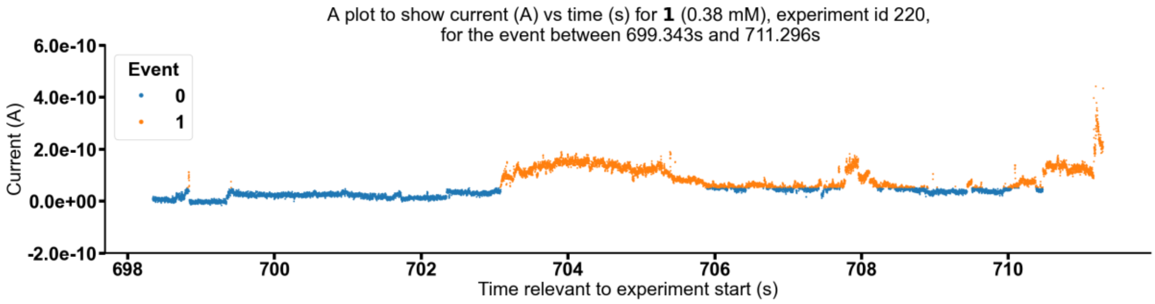

Figure S315 - Graph showing current (A) vs time (s) for **1** (0.38 mM), experiment id 220 at +100 mV, for the event between 699.343s and 711.296s.

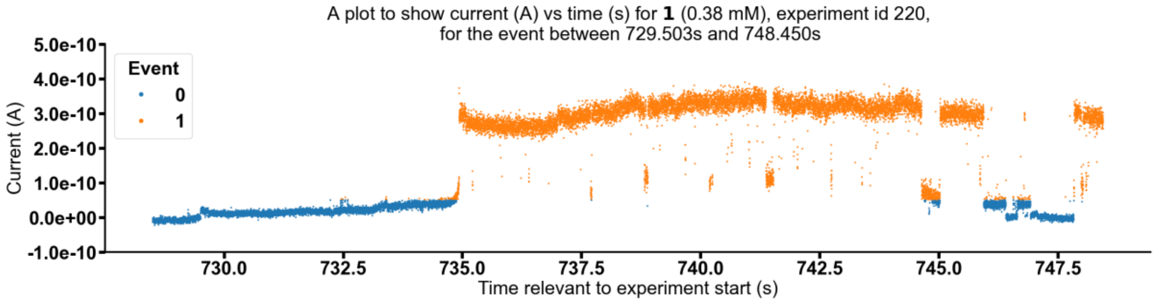

Figure S316 - Graph showing current (A) vs time (s) for **1** (0.38 mM), experiment id 220 at +100 mV, for the event between 729.503s and 748.450s.

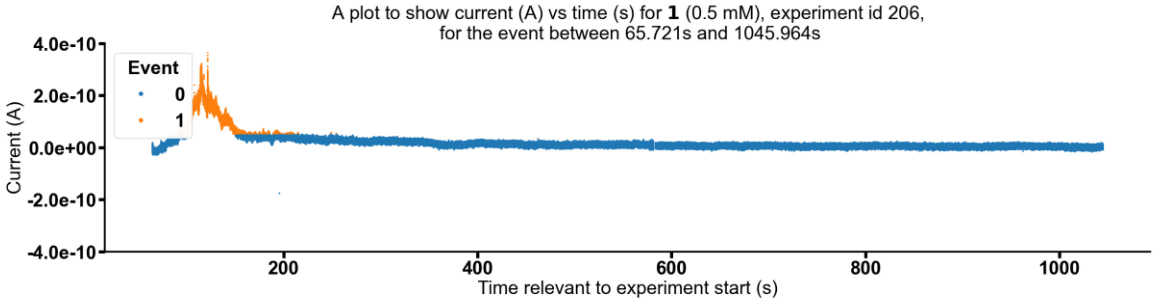

Figure S317 - Graph showing current (A) vs time (s) for **1** (0.5 mM), experiment id 206 at +100 mV, for the event between 65.721s and 1045.964s.

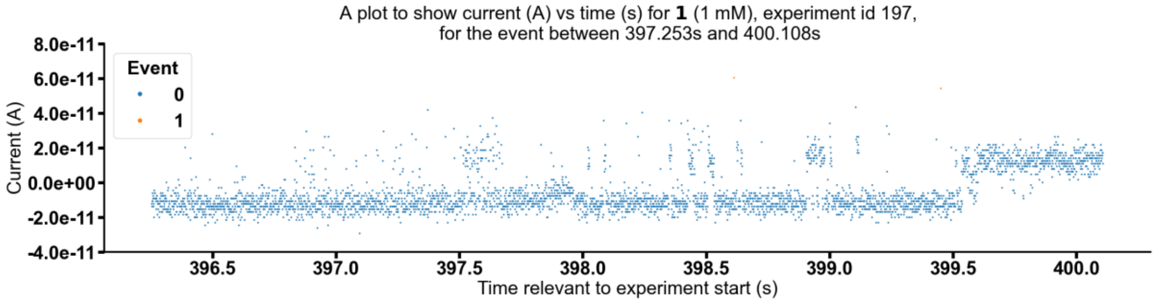

Figure S318 - Graph showing current (A) vs time (s) for **1** (1 mM), experiment id 197 at +100 mV, for the event between 397.253s and 400.108s.

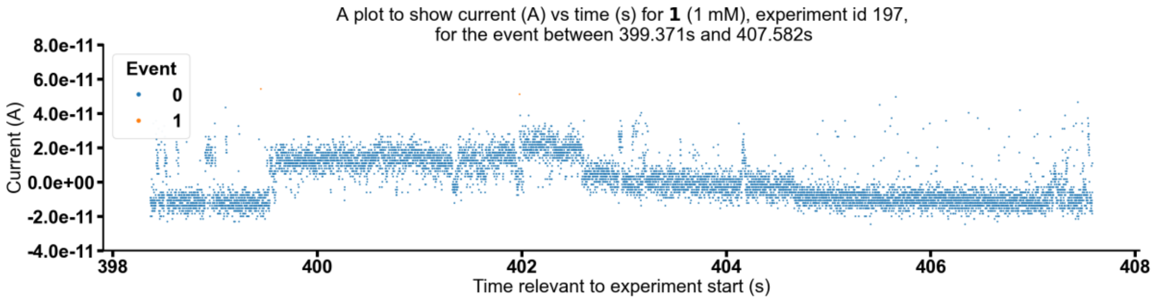

Figure S319 - Graph showing current (A) vs time (s) for **1** (1 mM), experiment id 197 at +100 mV, for the event between 399.371s and 407.582s.

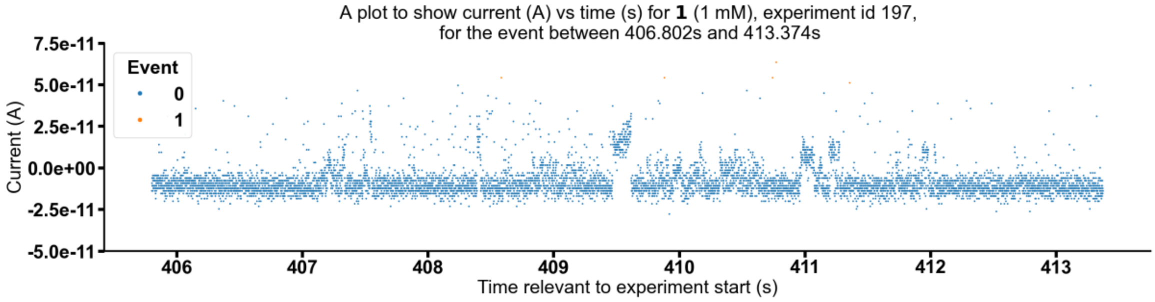

Figure S320 - Graph showing current (A) vs time (s) for **1** (1 mM), experiment id 197 at +100 mV, for the event between 406.802s and 413.374s.

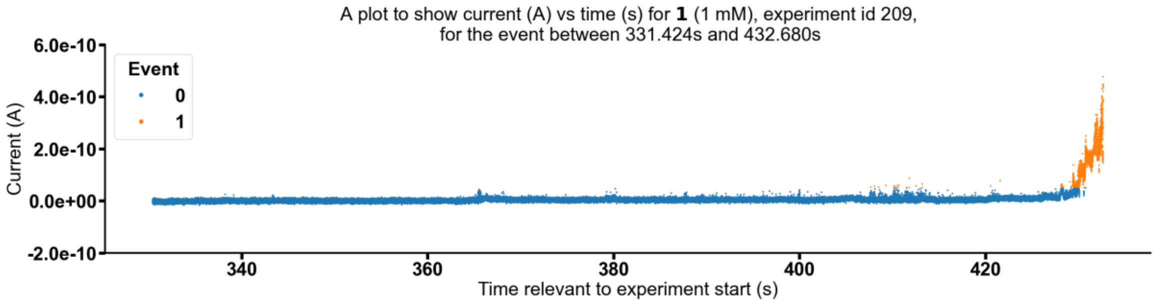

Figure S321 - Graph showing current (A) vs time (s) for **1** (1 mM), experiment id 209 at +100 mV, for the event between 331.424s and 432.680s.

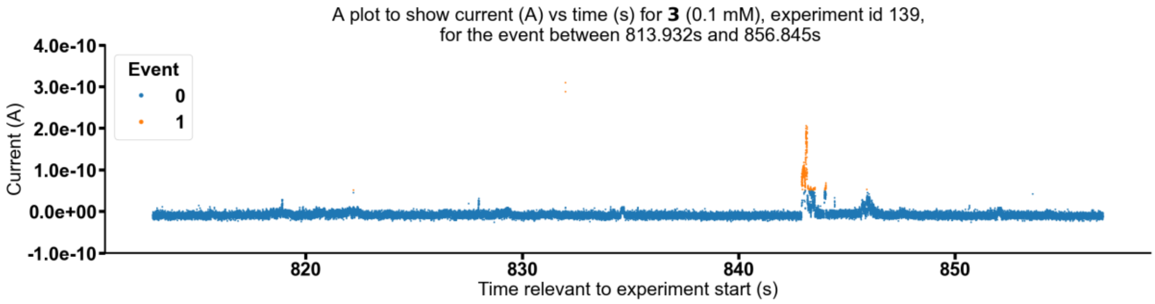

Figure S322 - Graph showing current (A) vs time (s) for **3** (0.1 mM), experiment id 139 at +100 mV, for the event between 813.932s and 856.845s.

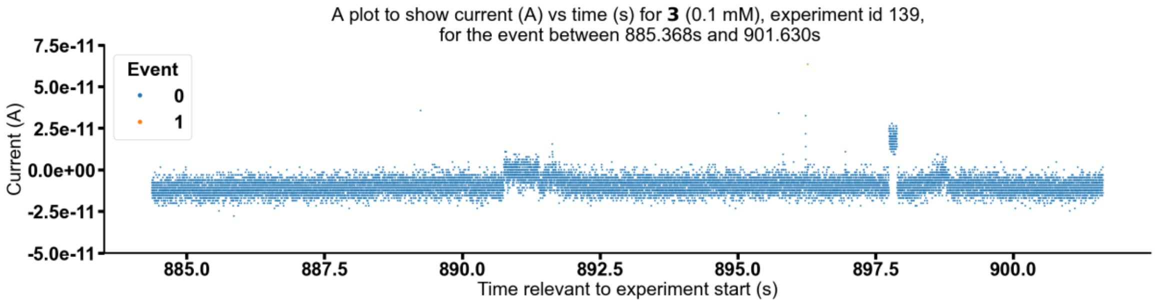

Figure S323 - Graph showing current (A) vs time (s) for **3** (0.1 mM), experiment id 139 at +100 mV, for the event between 885.368s and 901.630s.

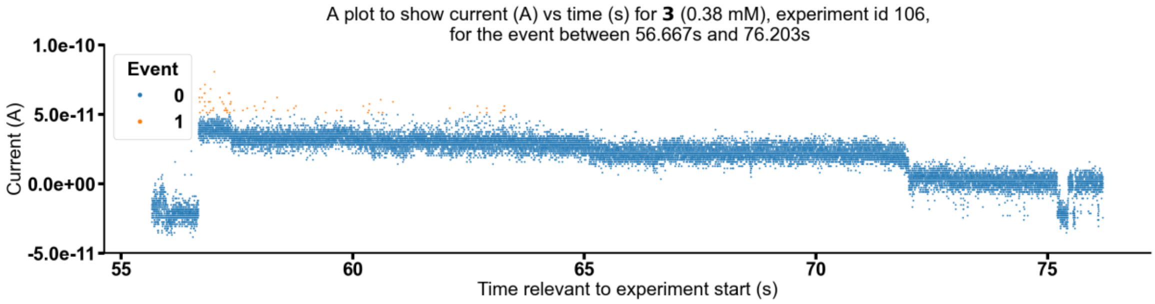

Figure S324 - Graph showing current (A) vs time (s) for **3** (0.38 mM), experiment id 106 at +100 mV, for the event between 56.667s and 76.203s.

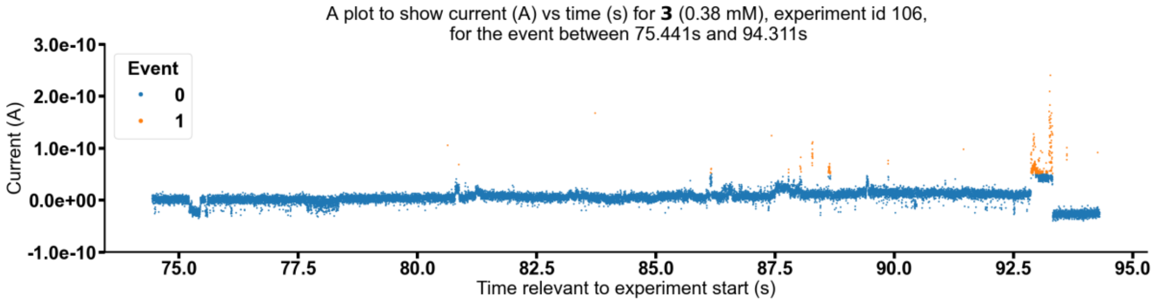

Figure S325 - Graph showing current (A) vs time (s) for **3** (0.38 mM), experiment id 106 at +100 mV, for the event between 75.441s and 94.311s.

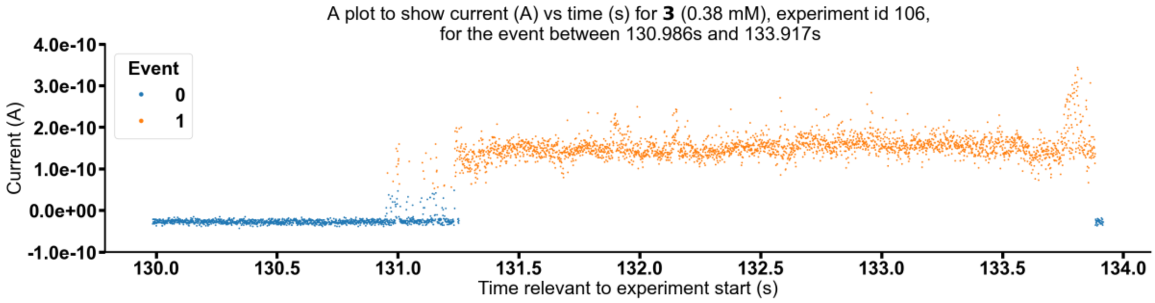

Figure S326 - Graph showing current (A) vs time (s) for **3** (0.38 mM), experiment id 106 at +100 mV, for the event between 130.986s and 133.917s.

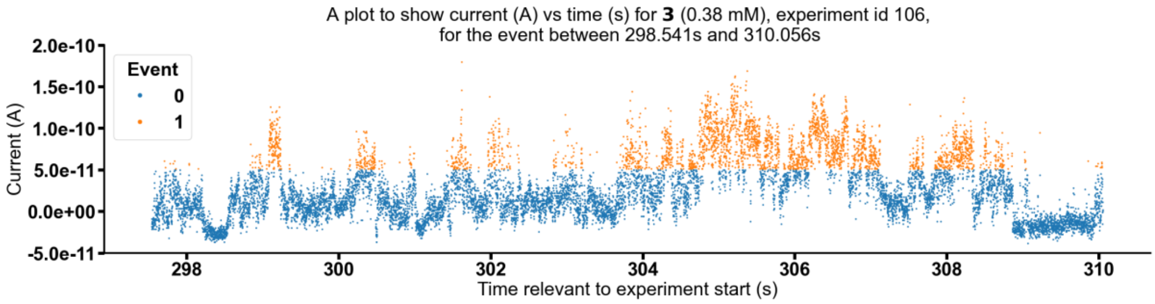

Figure S327 - Graph showing current (A) vs time (s) for **3** (0.38 mM), experiment id 106 at +100 mV, for the event between 298.541s and 310.056s.

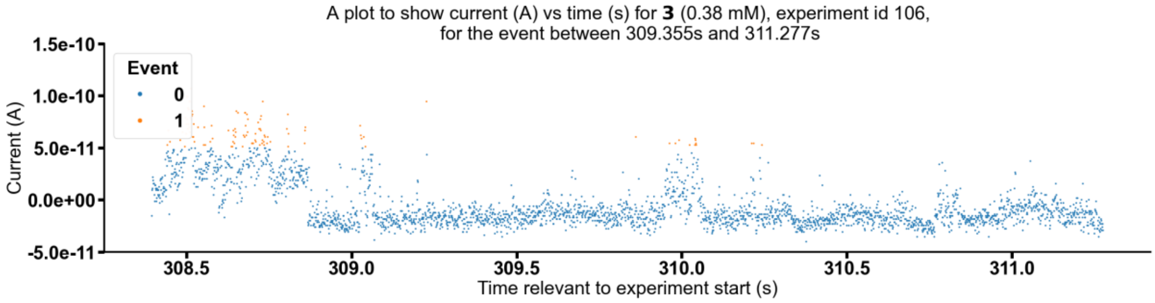

Figure S328 - Graph showing current (A) vs time (s) for **3** (0.38 mM), experiment id 106 at +100 mV, for the event between 309.355s and 311.277s.

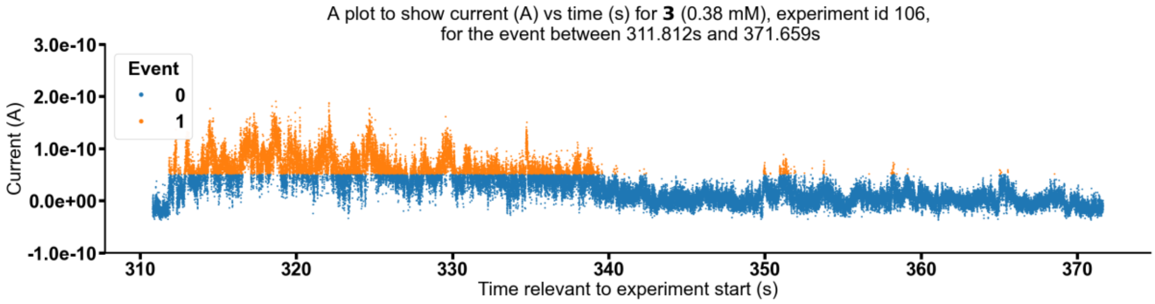

Figure S329 - Graph showing current (A) vs time (s) for **3** (0.38 mM), experiment id 106 at +100 mV, for the event between 311.812s and 371.659s.

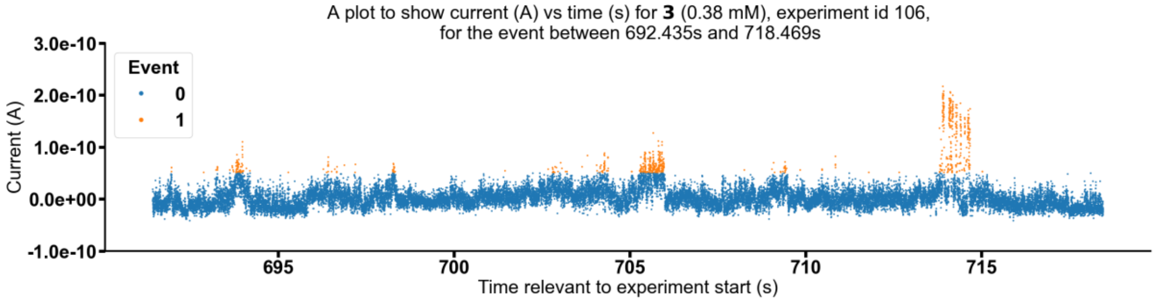

Figure S330 - Graph showing current (A) vs time (s) for **3** (0.38 mM), experiment id 106 at +100 mV, for the event between 692.435s and 718.469s.

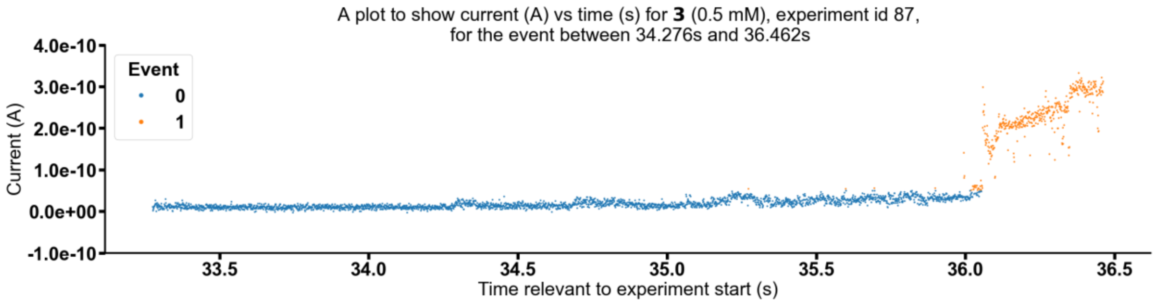

Figure S331 - Graph showing current (A) vs time (s) for **3** (0.5 mM), experiment id 87 at +100 mV, for the event between 34.276s and 36.462s.

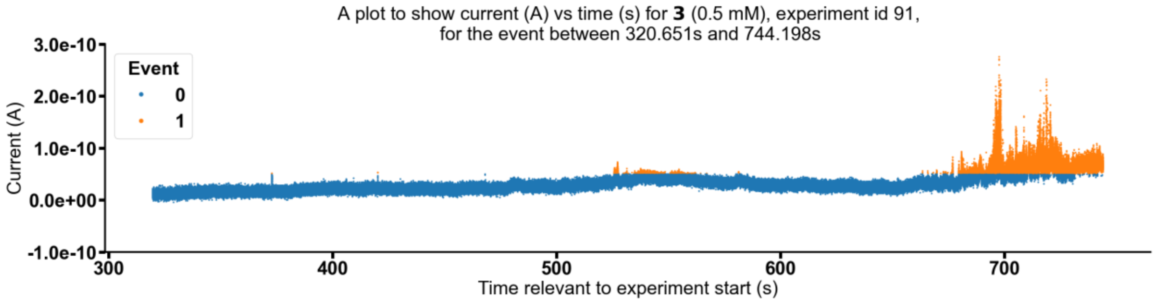

Figure S332 - Graph showing current (A) vs time (s) for **3** (0.5 mM), experiment id 91 at +100 mV, for the event between 320.651s and 744.198s.

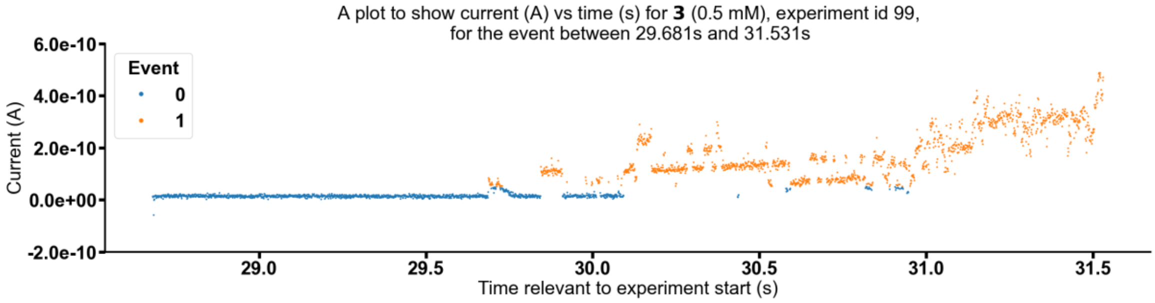

Figure S333 - Graph showing current (A) vs time (s) for **3** (0.5 mM), experiment id 99 at +100 mV, for the event between 29.681s and 31.531s.

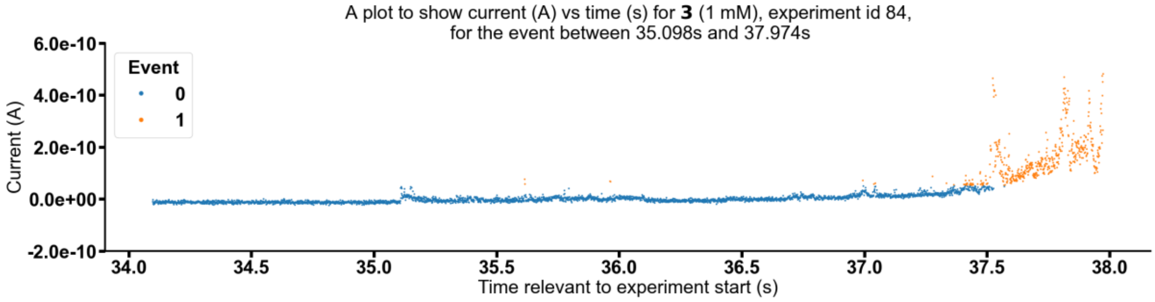

Figure S334 - Graph showing current (A) vs time (s) for **3** (1 mM), experiment id 84 at +100 mV, for the event between 35.098s and 37.974s.

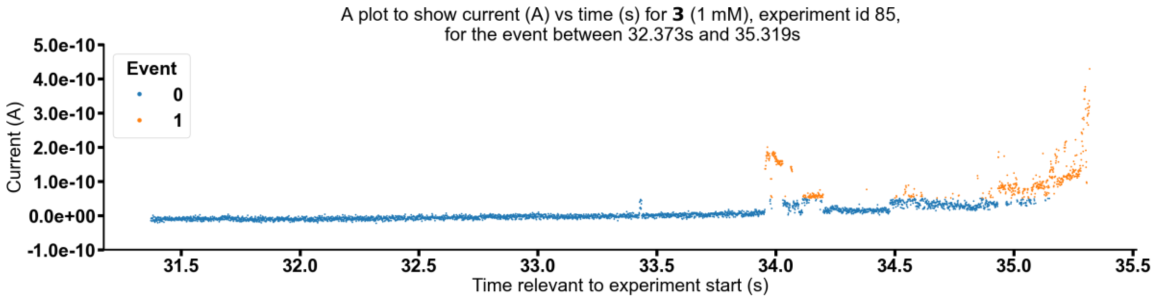

Figure S335 - Graph showing current (A) vs time (s) for **3** (1 mM), experiment id 85 at +100 mV, for the event between 32.373s and 35.319s.

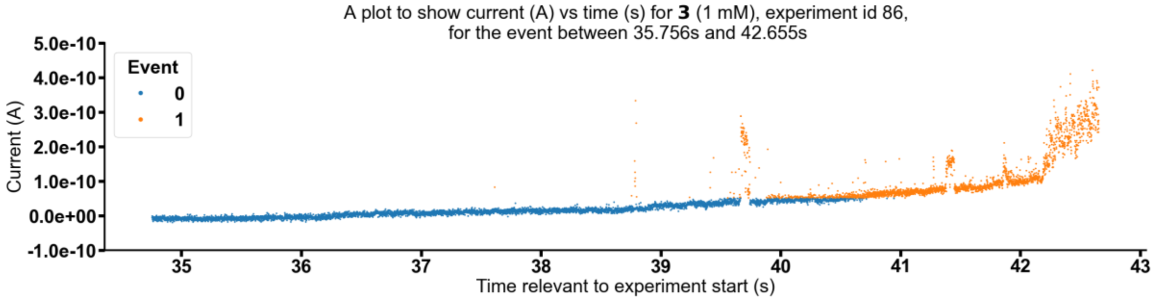

Figure S336 - Graph showing current (A) vs time (s) for **3** (1 mM), experiment id 86 at +100 mV, for the event between 35.756s and 42.655s.

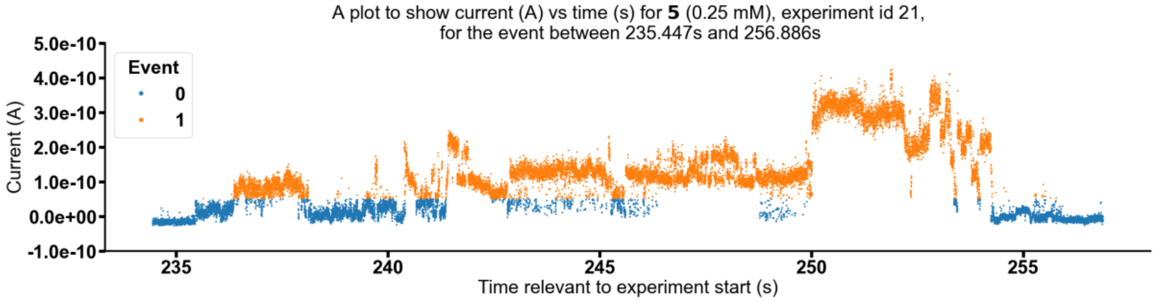

Figure S337 - Graph showing current (A) vs time (s) for **5** (0.25 mM), experiment id 21 at +100 mV, for the event between 235.447s and 256.886s.

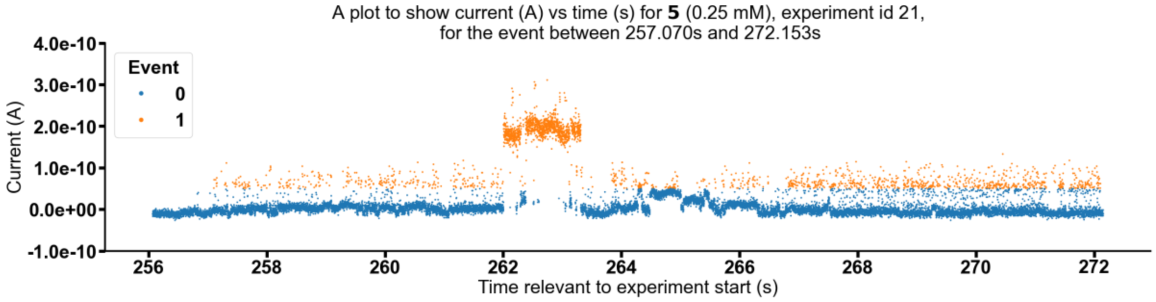

Figure S338 - Graph showing current (A) vs time (s) for **5** (0.25 mM), experiment id 21 at +100 mV, for the event between 257.070s and 272.153s.

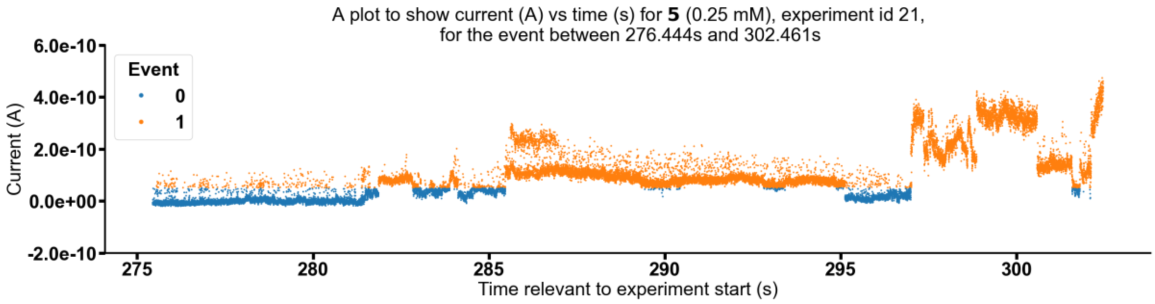

Figure S339 - Graph showing current (A) vs time (s) for **5** (0.25 mM), experiment id 21 at +100 mV, for the event between 276.444s and 302.461s.

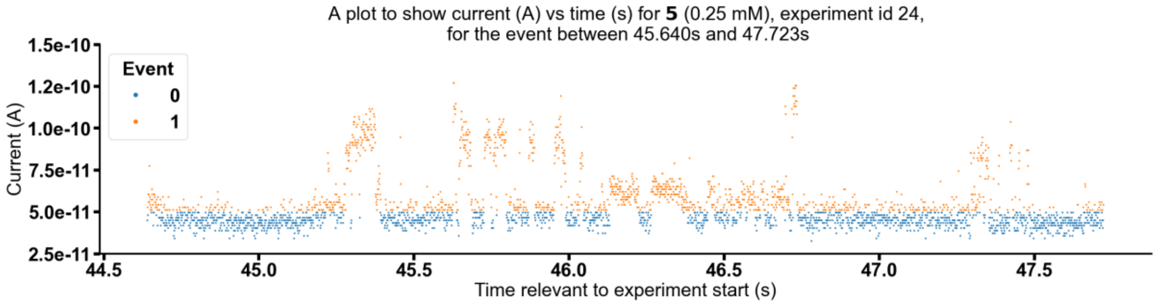

Figure S340 - Graph showing current (A) vs time (s) for **5** (0.25 mM), experiment id 24 at +100 mV, for the event between 45.640s and 47.723s.

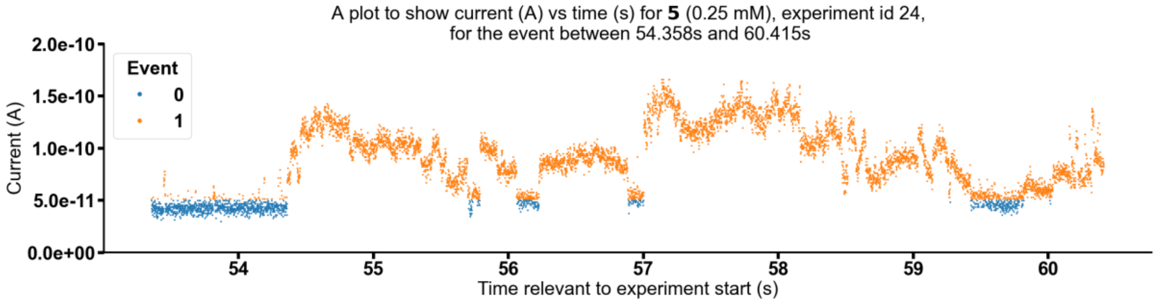

Figure S341 - Graph showing current (A) vs time (s) for **5** (0.25 mM), experiment id 24 at +100 mV, for the event between 54.358s and 60.415s.

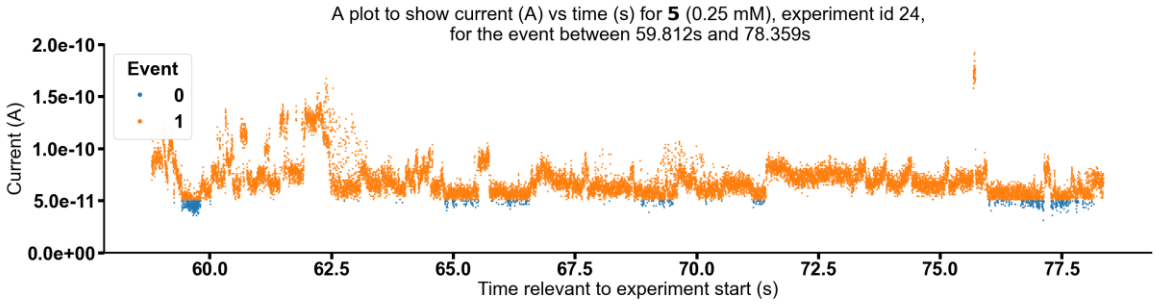

Figure S342 - Graph showing current (A) vs time (s) for **5** (0.25 mM), experiment id 24 at +100 mV, for the event between 59.812s and 78.359s.

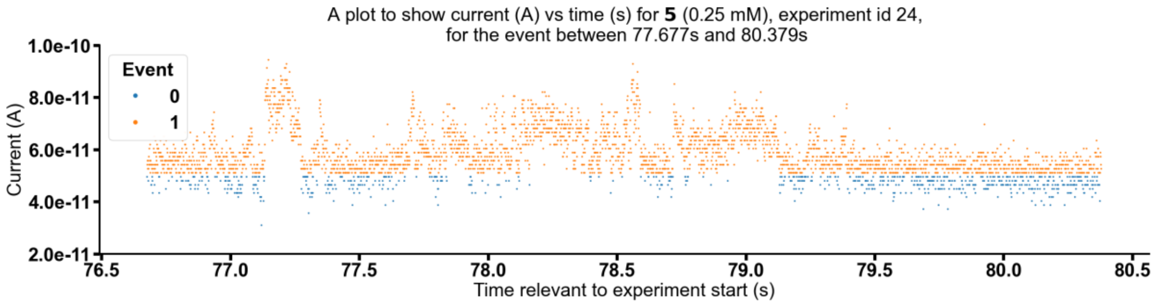

Figure S343 - Graph showing current (A) vs time (s) for **5** (0.25 mM), experiment id 24 at +100 mV, for the event between 77.677s and 80.379s.

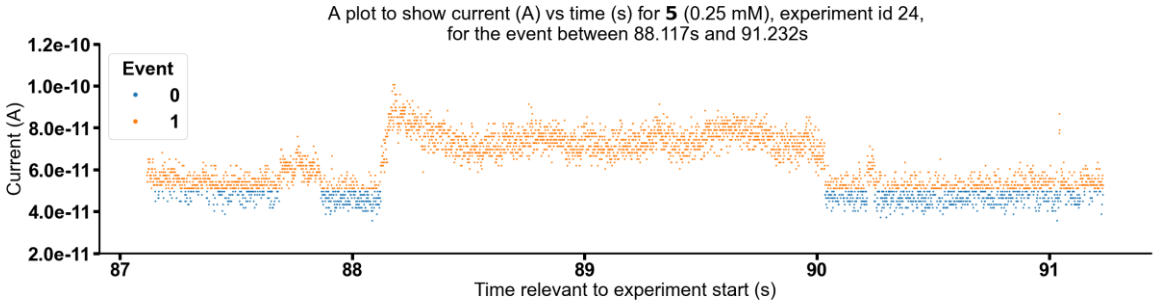

Figure S344 - Graph showing current (A) vs time (s) for **5** (0.25 mM), experiment id 24 at +100 mV, for the event between 88.117s and 91.232s.

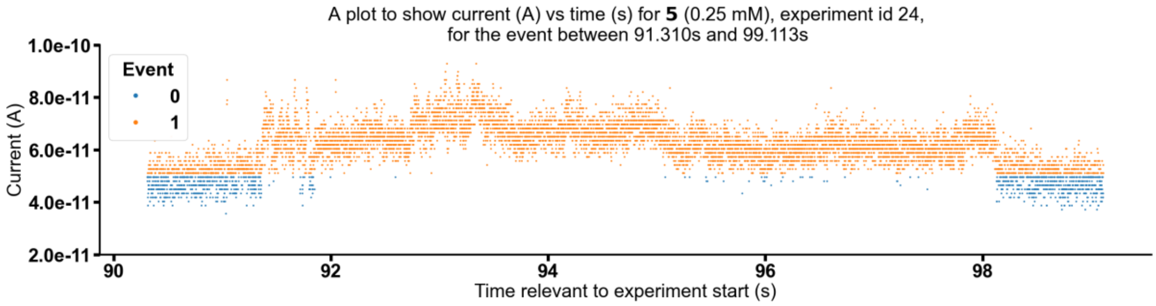

Figure S345 - Graph showing current (A) vs time (s) for **5** (0.25 mM), experiment id 24 at +100 mV, for the event between 91.310s and 99.113s.

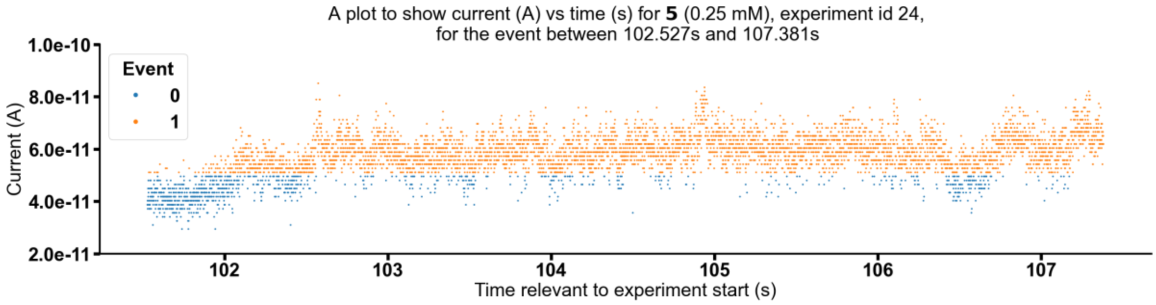

Figure S346 - Graph showing current (A) vs time (s) for **5** (0.25 mM), experiment id 24 at +100 mV, for the event between 102.527s and 107.381s.

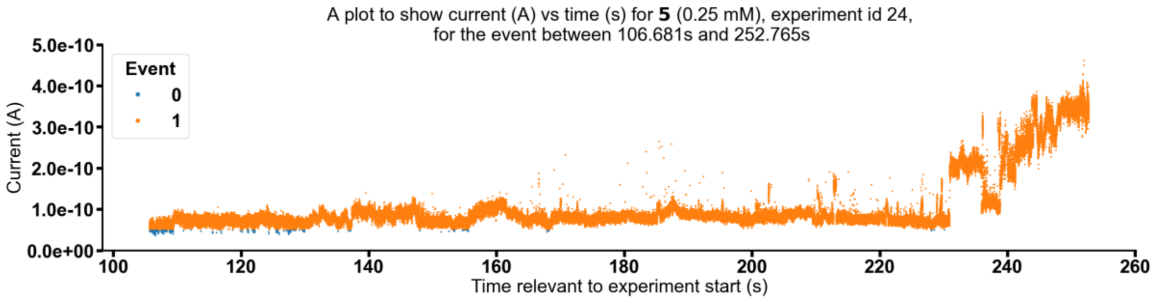

Figure S347 - Graph showing current (A) vs time (s) for **5** (0.25 mM), experiment id 24 at +100 mV, for the event between 106.681s and 252.765s.

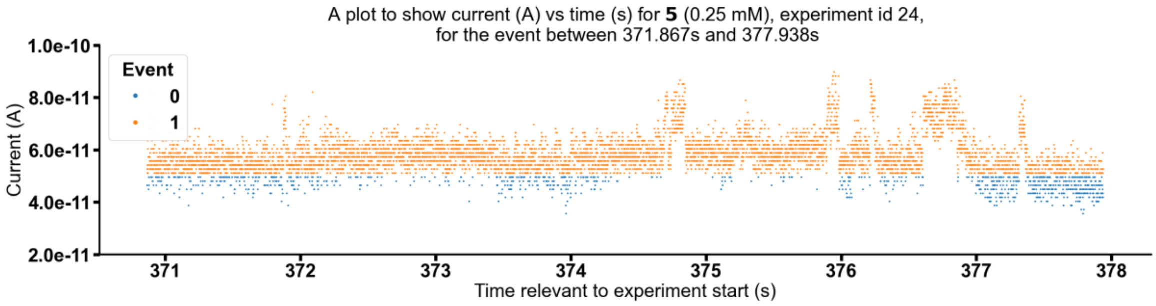

Figure S348 - Graph showing current (A) vs time (s) for **5** (0.25 mM), experiment id 24 at +100 mV, for the event between 371.867s and 377.938s.

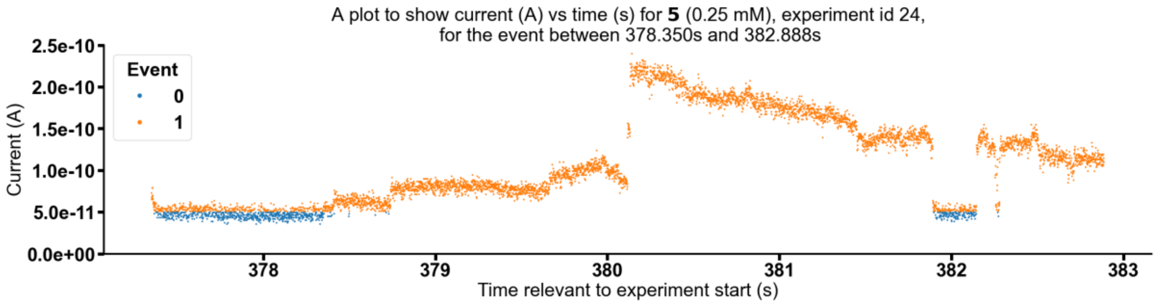

Figure S349 - Graph showing current (A) vs time (s) for **5** (0.25 mM), experiment id 24 at +100 mV, for the event between 378.350s and 382.888s.

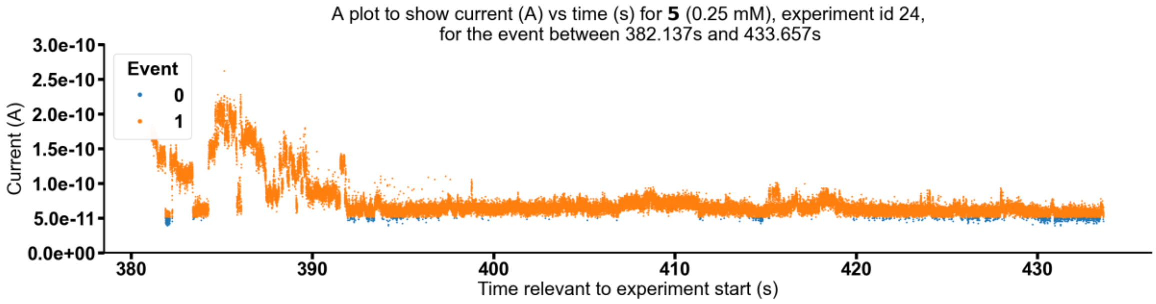

Figure S350 - Graph showing current (A) vs time (s) for **5** (0.25 mM), experiment id 24 at +100 mV, for the event between 382.137s and 433.657s.

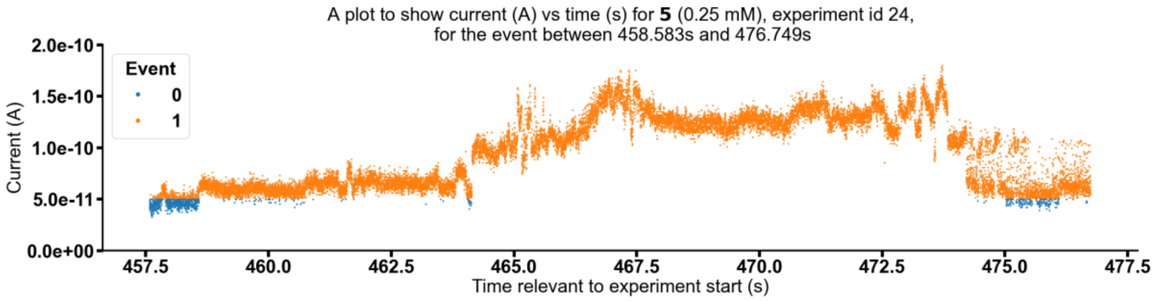

Figure S351 - Graph showing current (A) vs time (s) for **5** (0.25 mM), experiment id 24 at +100 mV, for the event between 458.583s and 476.749s.

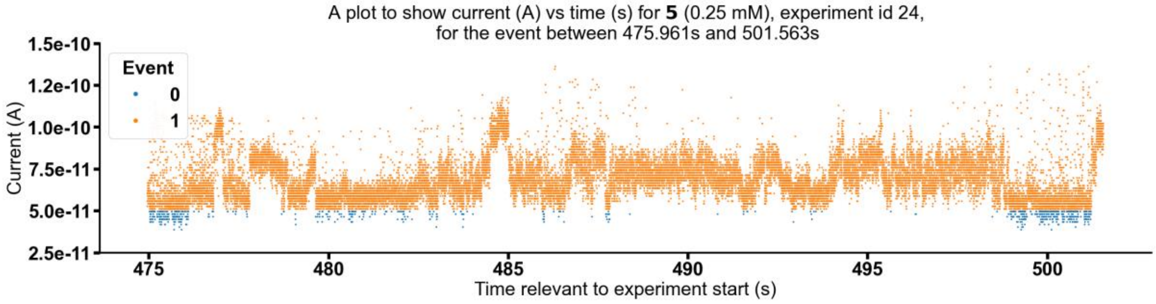

Figure S352 - Graph showing current (A) vs time (s) for **5** (0.25 mM), experiment id 24 at +100 mV, for the event between 475.961s and 501.563s.

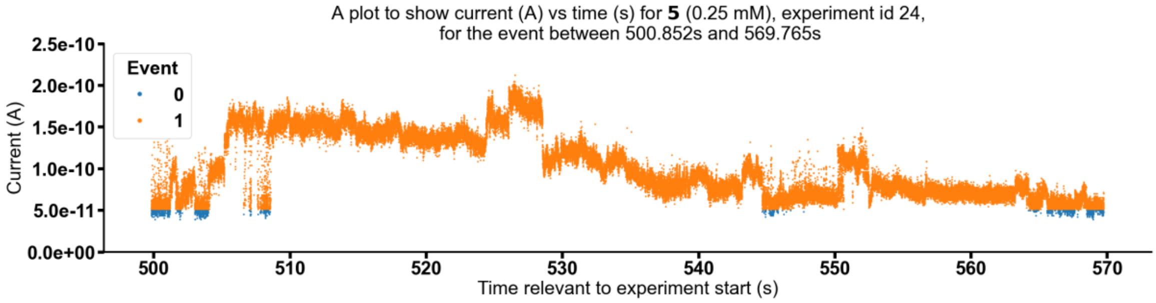

Figure S353 - Graph showing current (A) vs time (s) for **5** (0.25 mM), experiment id 24 at +100 mV, for the event between 500.852s and 569.765s.

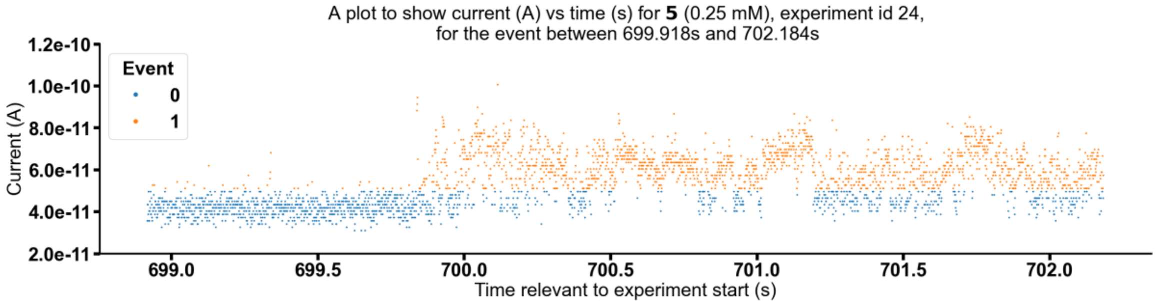

Figure S354 - Graph showing current (A) vs time (s) for **5** (0.25 mM), experiment id 24 at +100 mV, for the event between 699.918s and 702.184s.

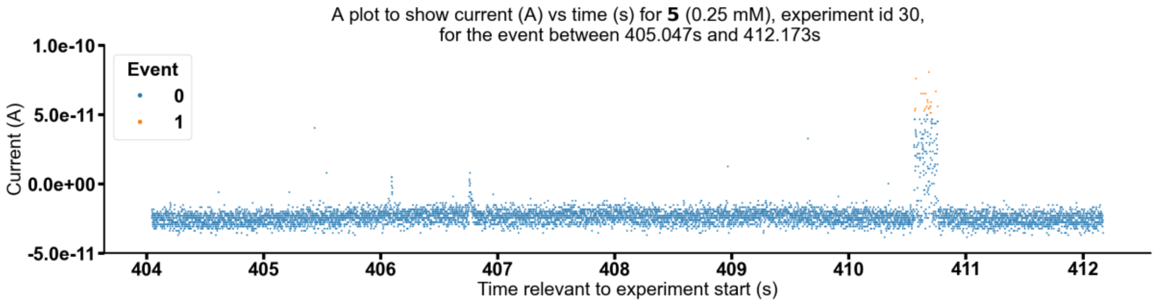

Figure S355 - Graph showing current (A) vs time (s) for **5** (0.25 mM), experiment id 30 at +100 mV, for the event between 405.047s and 412.173s.

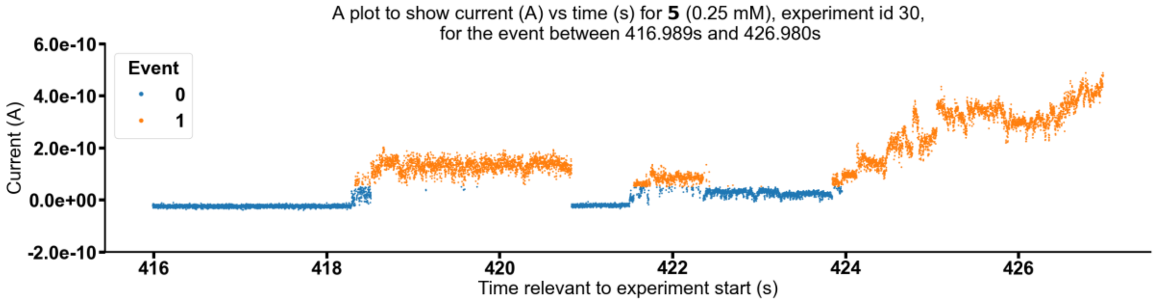

Figure S356 - Graph showing current (A) vs time (s) for **5** (0.25 mM), experiment id 30 at +100 mV, for the event between 416.989s and 426.980s.

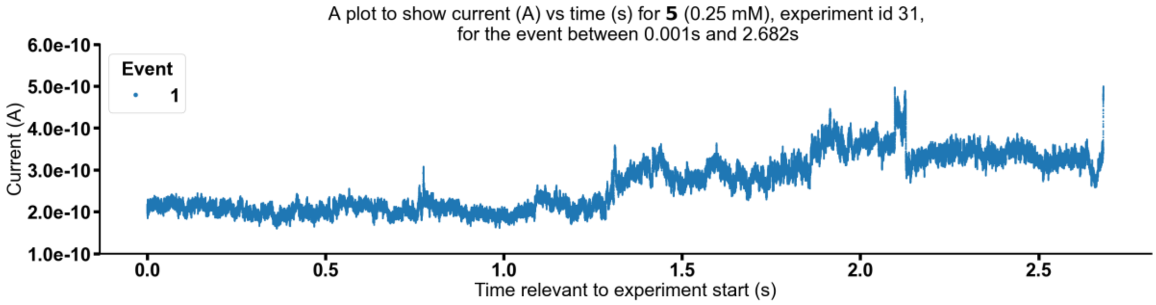

Figure S357 - Graph showing current (A) vs time (s) for **5** (0.25 mM), experiment id 31 at +100 mV, for the event between 0.001s and 2.682s.

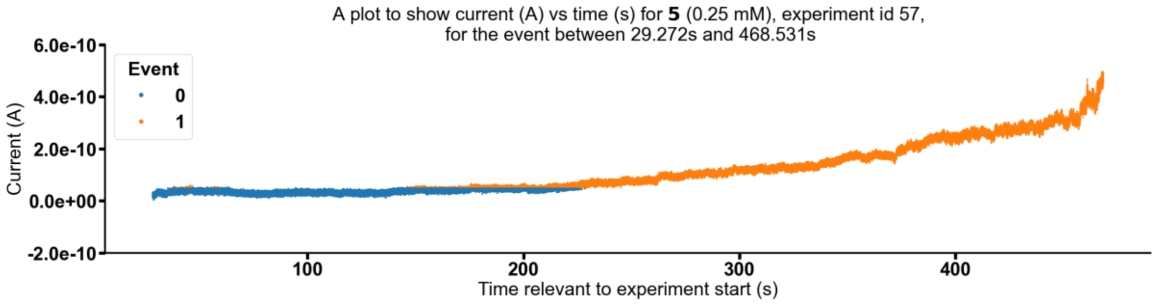

Figure S358 - Graph showing current (A) vs time (s) for **5** (0.25 mM), experiment id 57 at +100 mV, for the event between 29.272s and 468.531s.

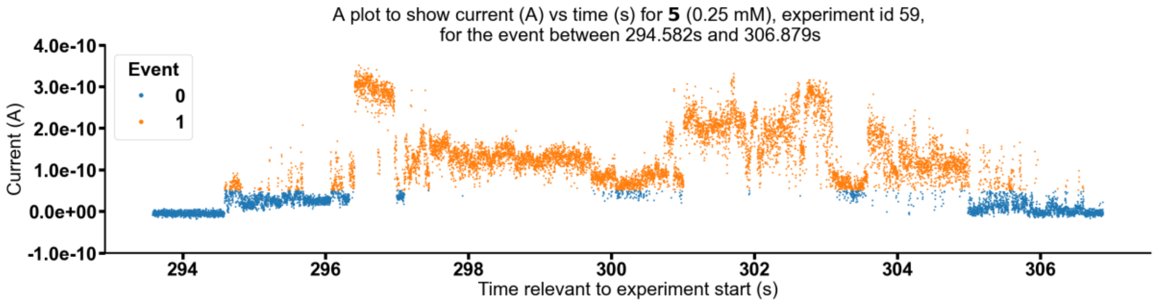

Figure S359 - Graph showing current (A) vs time (s) for **5** (0.25 mM), experiment id 59 at +100 mV, for the event between 294.582s and 306.879s.

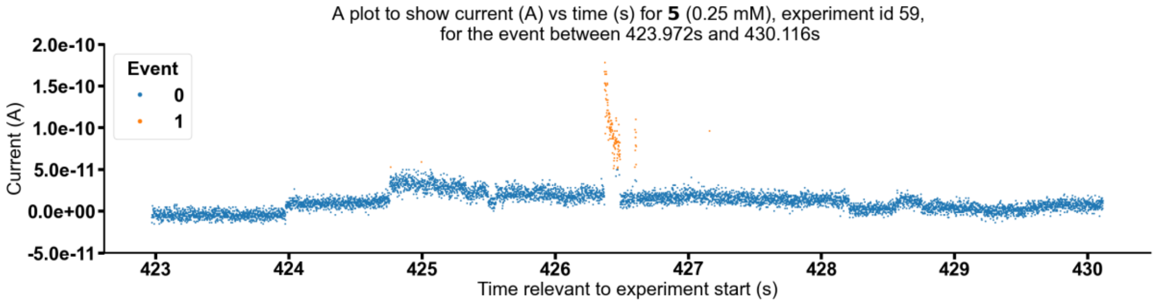

Figure S360 - Graph showing current (A) vs time (s) for **5** (0.25 mM), experiment id 59 at +100 mV, for the event between 423.972s and 430.116s.

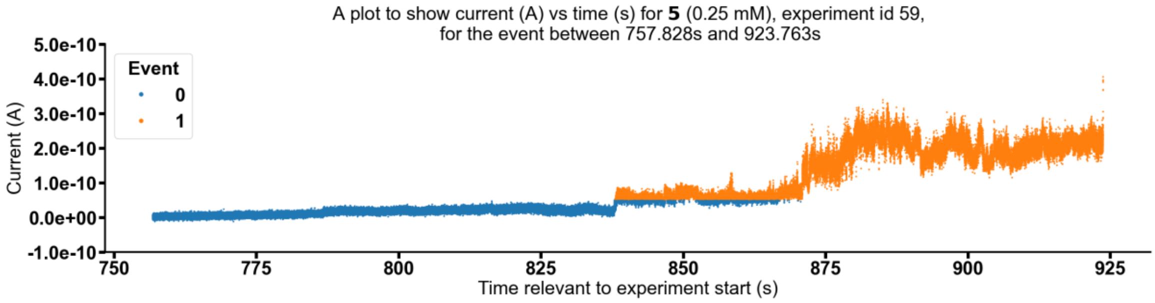

Figure S361 - Graph showing current (A) vs time (s) for **5** (0.25 mM), experiment id 59 at +100 mV, for the event between 757.828s and 923.763s.

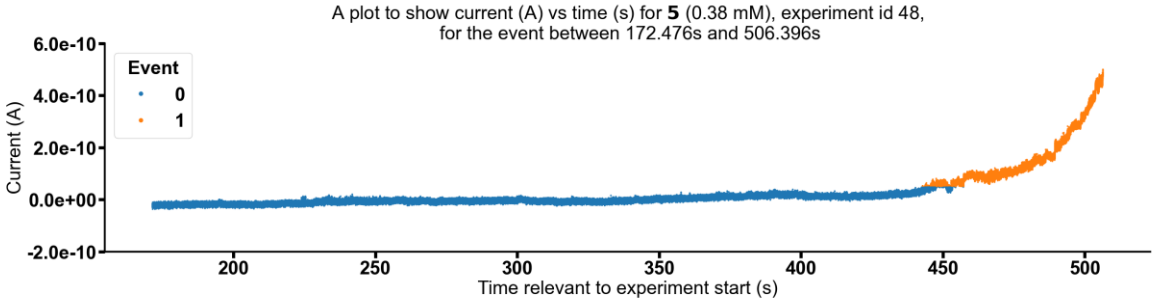

Figure S362 - Graph showing current (A) vs time (s) for **5** (0.38 mM), experiment id 48 at +100 mV, for the event between 172.476s and 506.396s.

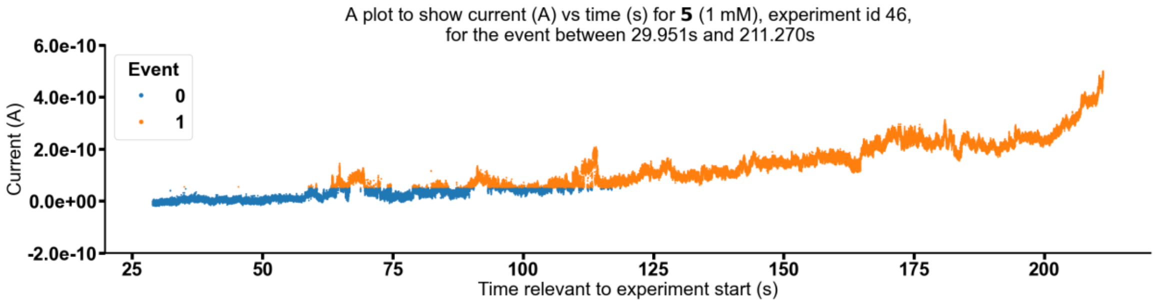

Figure S363 - Graph showing current (A) vs time (s) for **5** (1 mM), experiment id 46 at +100 mV, for the event between 29.951s and 211.270s.

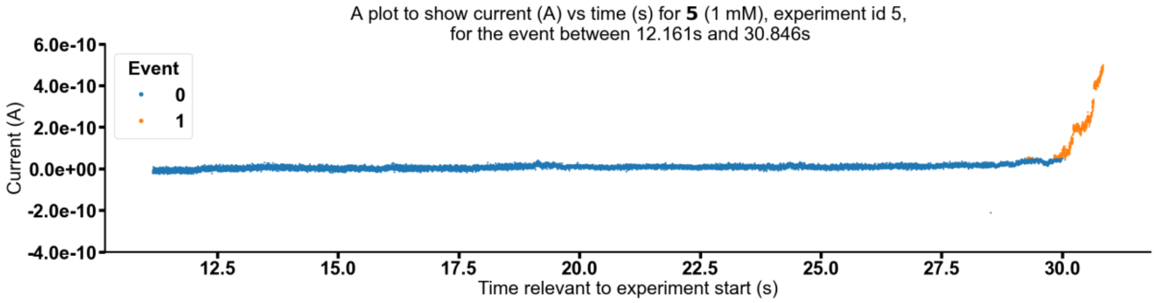

Figure S364 - Graph showing current (A) vs time (s) for **5** (1 mM), experiment id 5 at +100 mV, for the event between 12.161s and 30.846s.

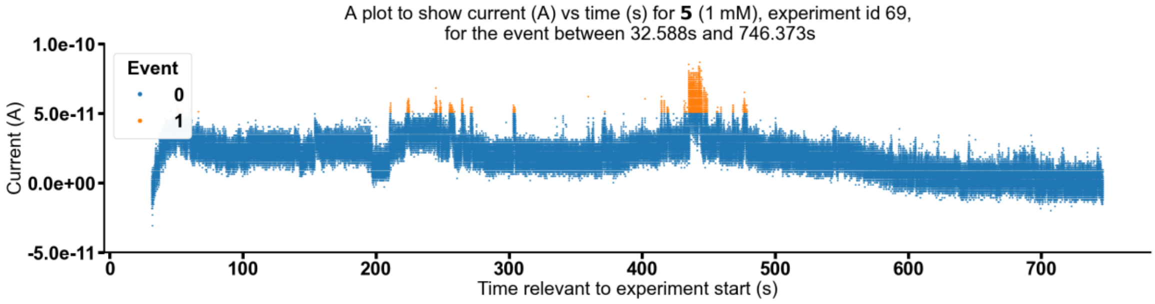

Figure S365 - Graph showing current (A) vs time (s) for **5** (1 mM), experiment id 69 at +100 mV, for the event between 32.588s and 746.373s.

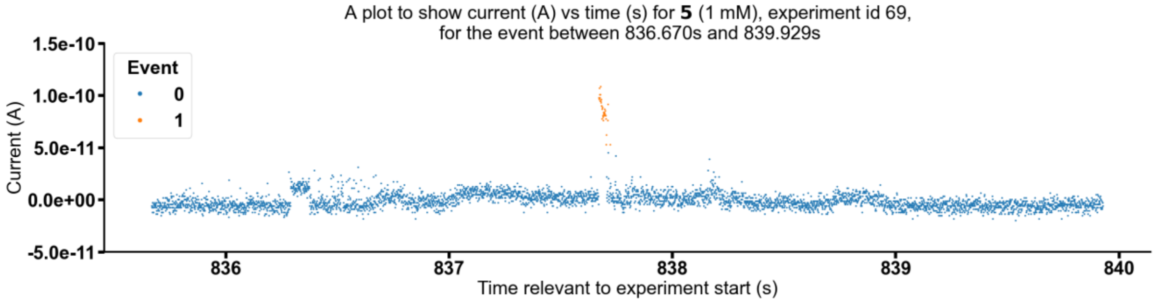

Figure S366 - Graph showing current (A) vs time (s) for **5** (1 mM), experiment id 69 at +100 mV, for the event between 836.670s and 839.929s.

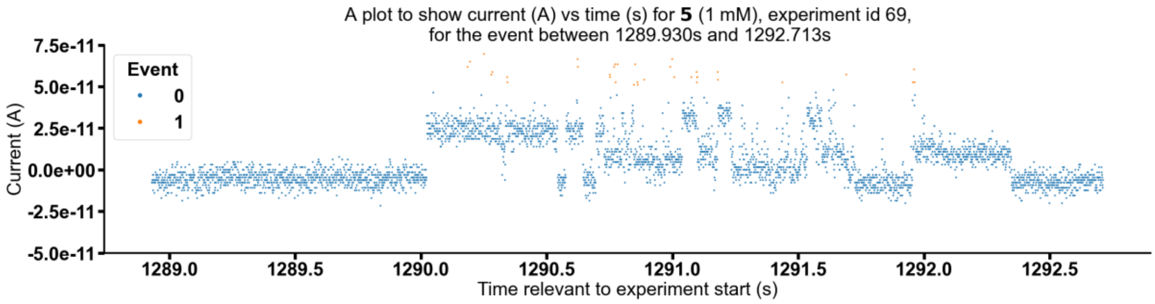

Figure S367 - Graph showing current (A) vs time (s) for **5** (1 mM), experiment id 69 at +100 mV, for the event between 1289.930s and 1292.713s.

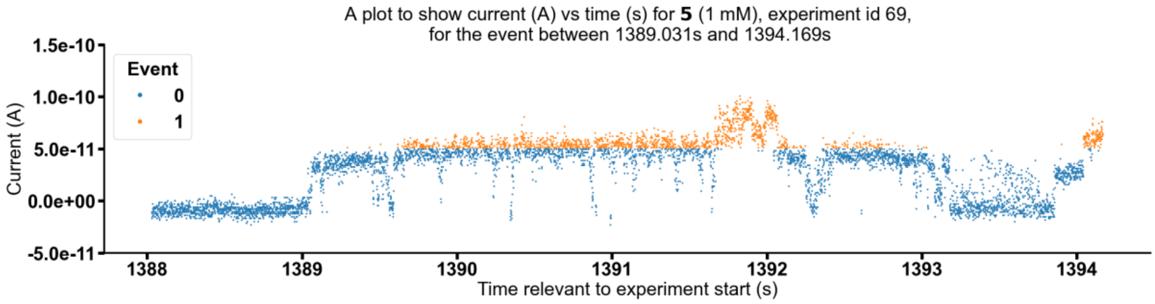

Figure S368 - Graph showing current (A) vs time (s) for **5** (1 mM), experiment id 69 at +100 mV, for the event between 1389.031s and 1394.169s.

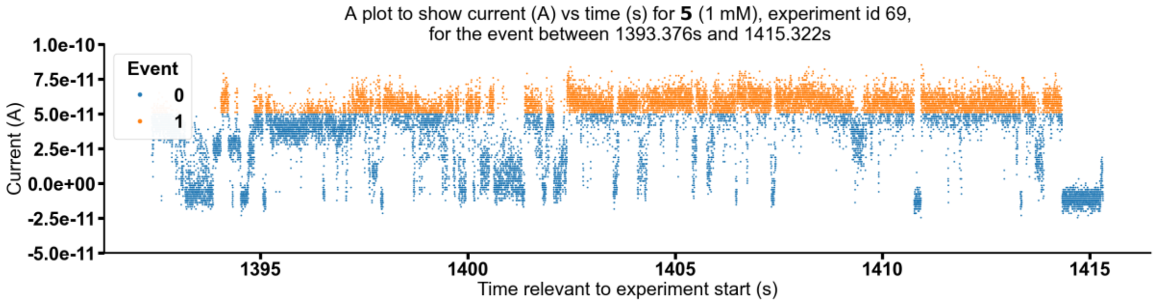

Figure S369 - Graph showing current (A) vs time (s) for **5** (1 mM), experiment id 69 at +100 mV, for the event between 1393.376s and 1415.322s.

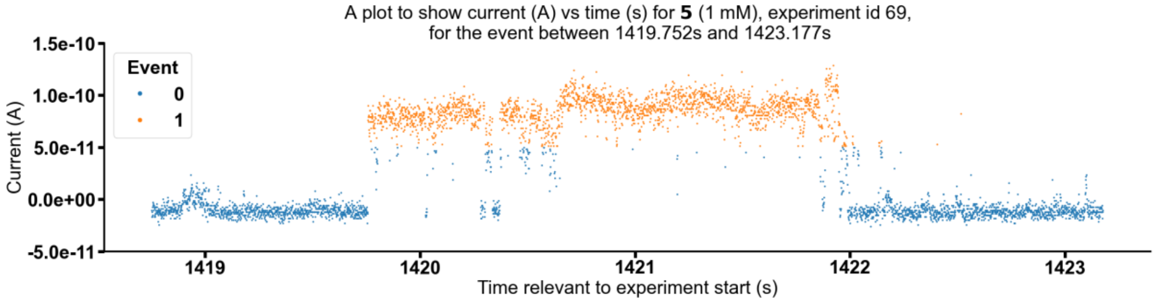

Figure S370 - Graph showing current (A) vs time (s) for **5** (1 mM), experiment id 69 at +100 mV, for the event between 1419.752s and 1423.177s.

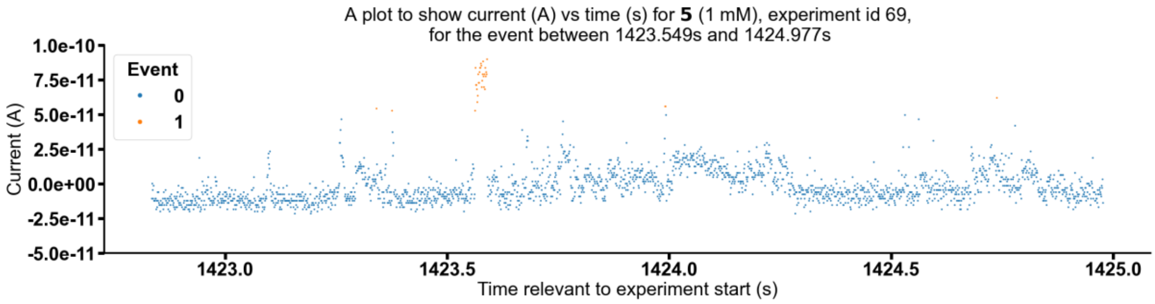

Figure S371 - Graph showing current (A) vs time (s) for **5** (1 mM), experiment id 69 at +100 mV, for the event between 1423.549s and 1424.977s.

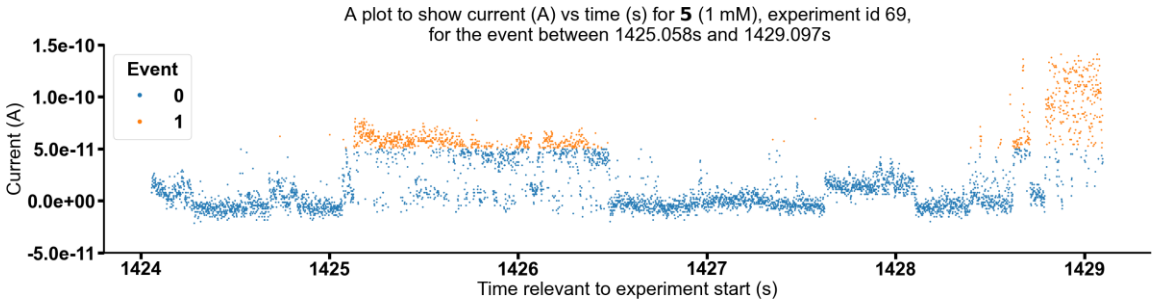

Figure S372 - Graph showing current (A) vs time (s) for **5** (1 mM), experiment id 69 at +100 mV, for the event between 1425.058s and 1429.097s.

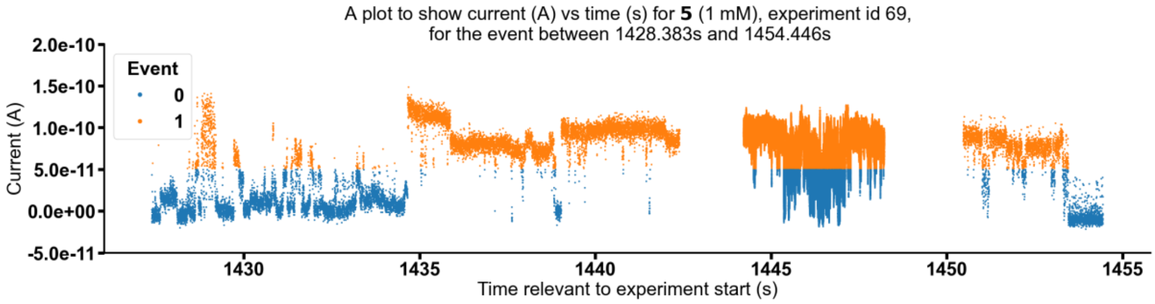

Figure S373 - Graph showing current (A) vs time (s) for **5** (1 mM), experiment id 69 at +100 mV, for the event between 1428.383s and 1454.446s.

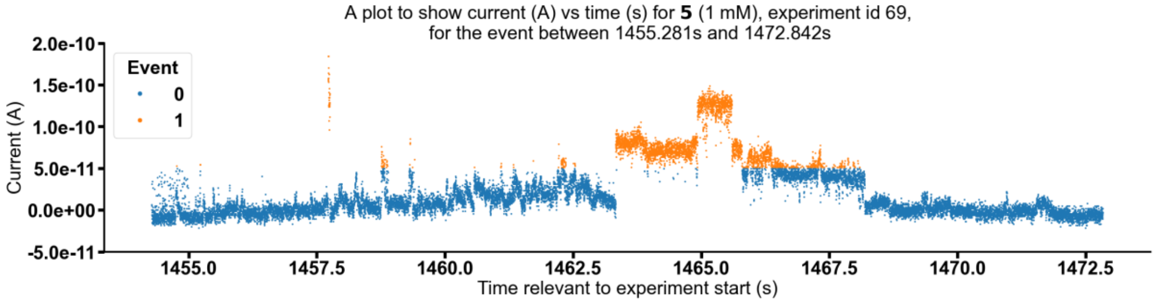

Figure S374 - Graph showing current (A) vs time (s) for **5** (1 mM), experiment id 69 at +100 mV, for the event between 1455.281s and 1472.842s.

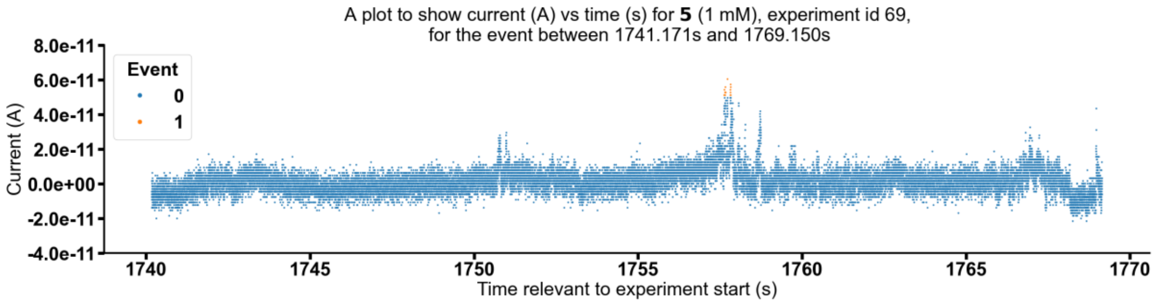

Figure S375 - Graph showing current (A) vs time (s) for **5** (1 mM), experiment id 69 at +100 mV, for the event between 1741.171s and 1769.150s.

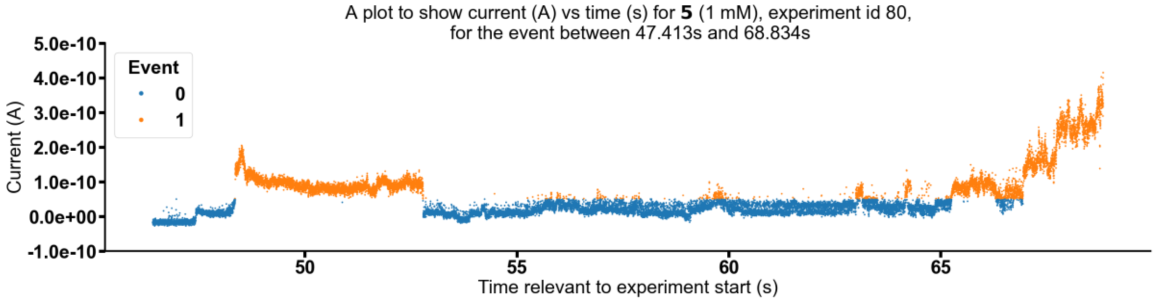

Figure S376 - Graph showing current (A) vs time (s) for **5** (1 mM), experiment id 80 at +100 mV, for the event between 47.413s and 68.834s.

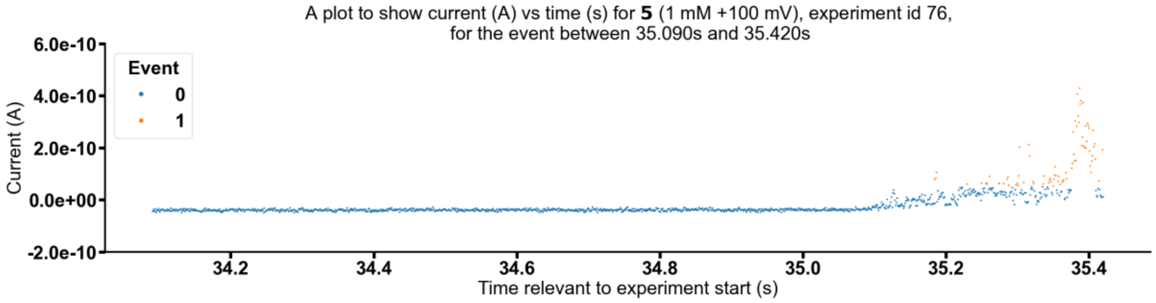

Figure S377 - Graph showing current (A) vs time (s) for **5** (1 mM), experiment id 76 at +100 mV, for the event between 35.090s and 35.420s.

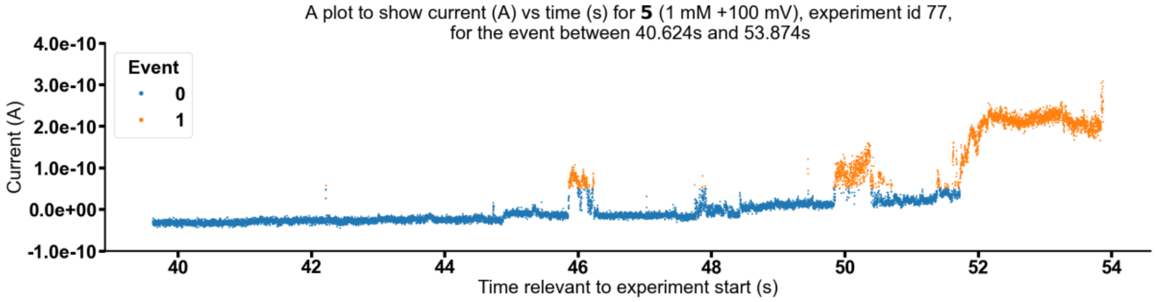

Figure S378 - Graph showing current (A) vs time (s) for **5** (1 mM), experiment id 77 at +100 mV, for the event between 40.624s and 53.874s.

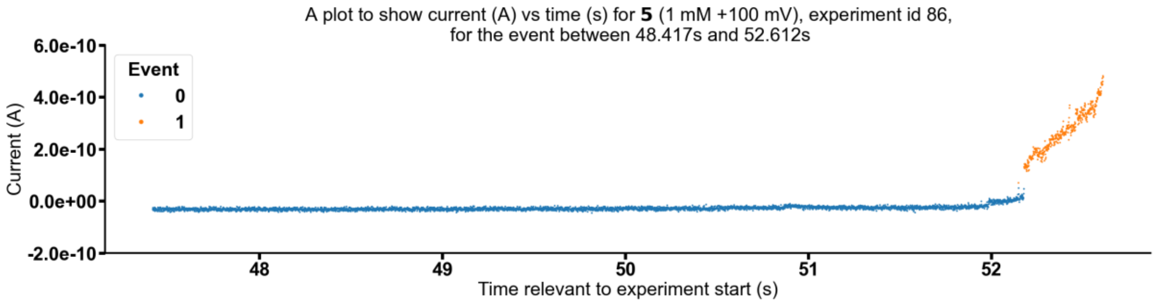

Figure S379 - Graph showing current (A) vs time (s) for **5** (1 mM), experiment id 86 at +100 mV, for the event between 48.417s and 52.612s.

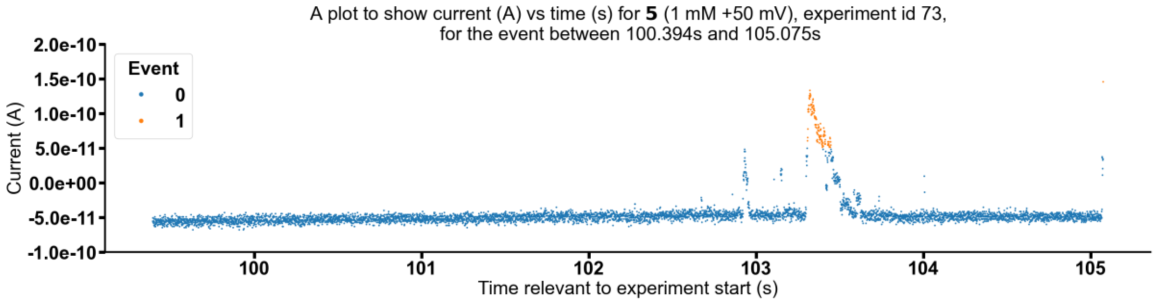

Figure S380 - Graph showing current (A) vs time (s) for **5** (1 mM), experiment id 73 at +50 mV, for the event between 100.394s and 105.075s.

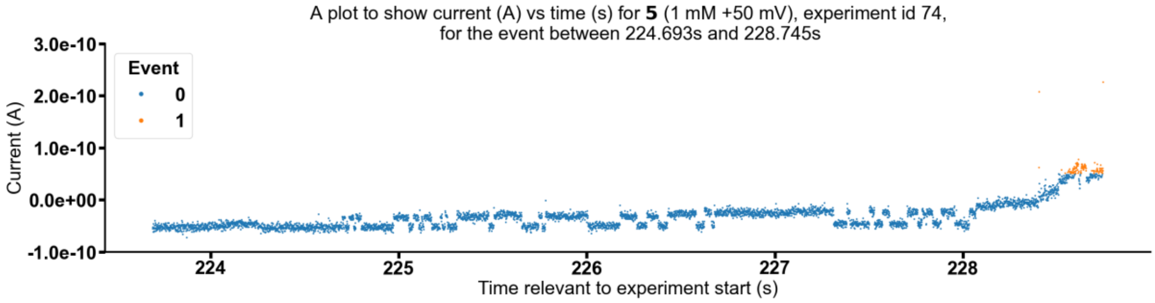

Figure S381 - Graph showing current (A) vs time (s) for **5** (1 mM), experiment id 74 at +50 mV, for the event between 224.693s and 228.745s.

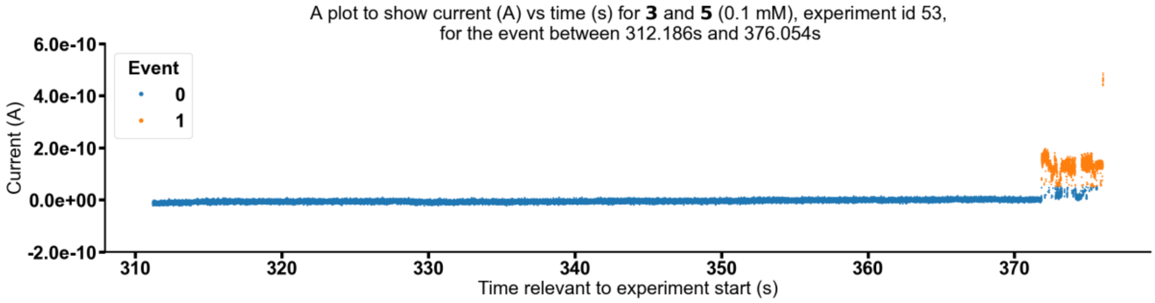

Figure S382 - Graph showing current (A) vs time (s) for **3** and **5** (0.1 mM), experiment id 53 at +100 mV, for the event between 312.186s and 376.054s.

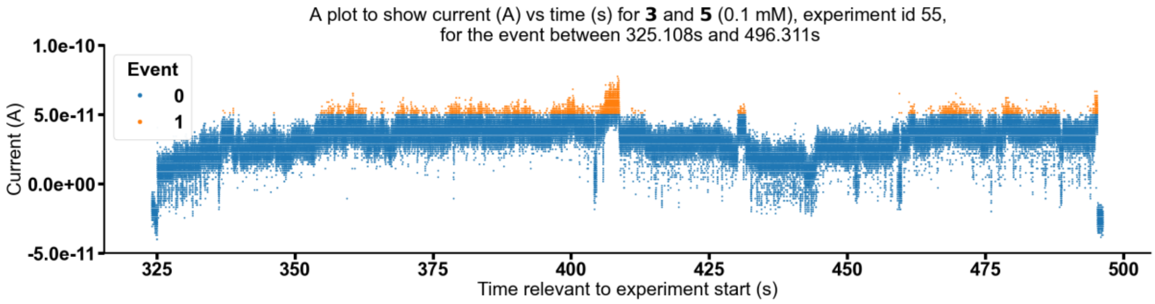

Figure S383 - Graph showing current (A) vs time (s) for **3** and **5** (0.1 mM), experiment id 55 at +100 mV, for the event between 325.108s and 496.311s.

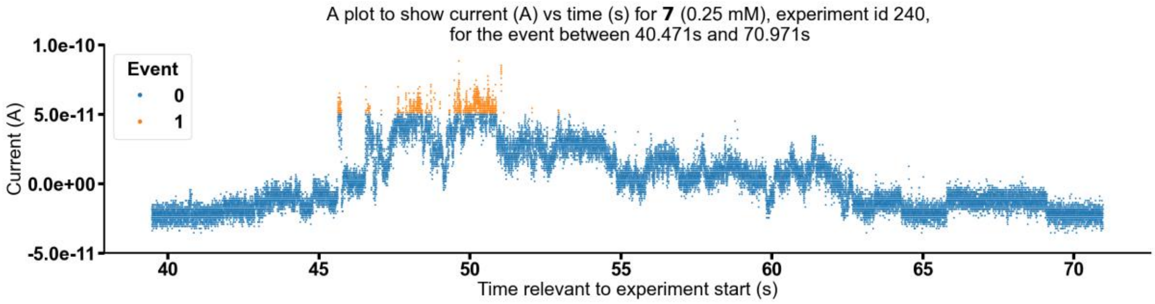

Figure S384 - Graph showing current (A) vs time (s) for **7** (0.25 mM), experiment id 240 at +100 mV, for the event between 40.471s and 70.971s.

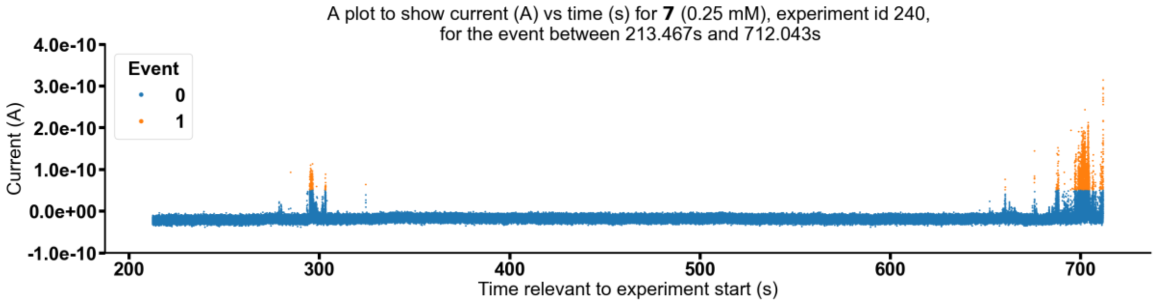

Figure S385 - Graph showing current (A) vs time (s) for **7** (0.25 mM), experiment id 240 at +100 mV, for the event between 213.467s and 712.043s.

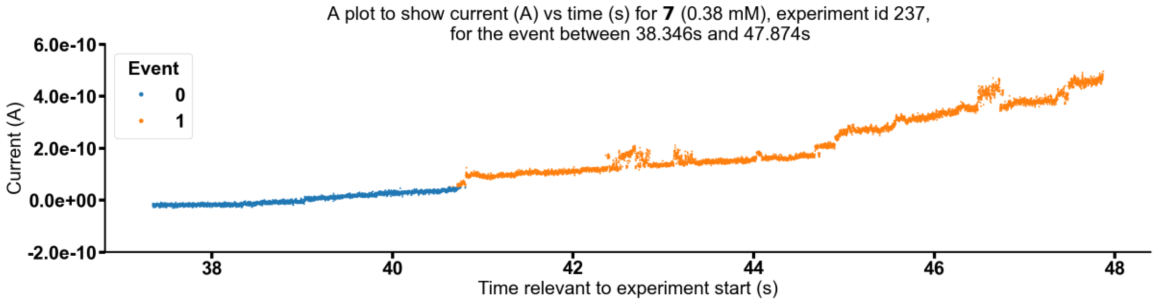

Figure S386 - Graph showing current (A) vs time (s) for **7** (0.38 mM), experiment id 237 at +100 mV, for the event between 38.346s and 47.874s.

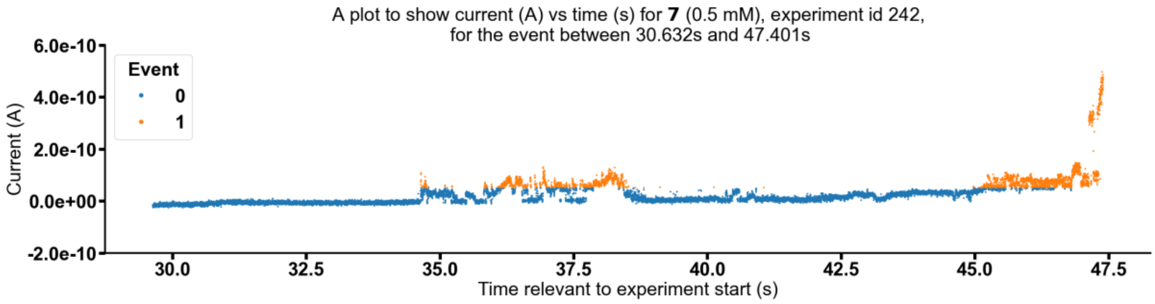

Figure S387 - Graph showing current (A) vs time (s) for **7** (0.5 mM), experiment id 242 at +100 mV, for the event between 30.632s and 47.401s.

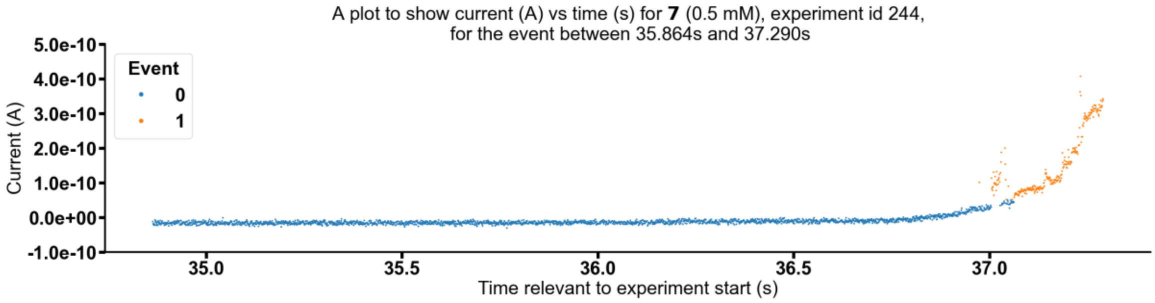

Figure S388 - Graph showing current (A) vs time (s) for **7** (0.5 mM), experiment id 244 at +100 mV, for the event between 35.864s and 37.290s.

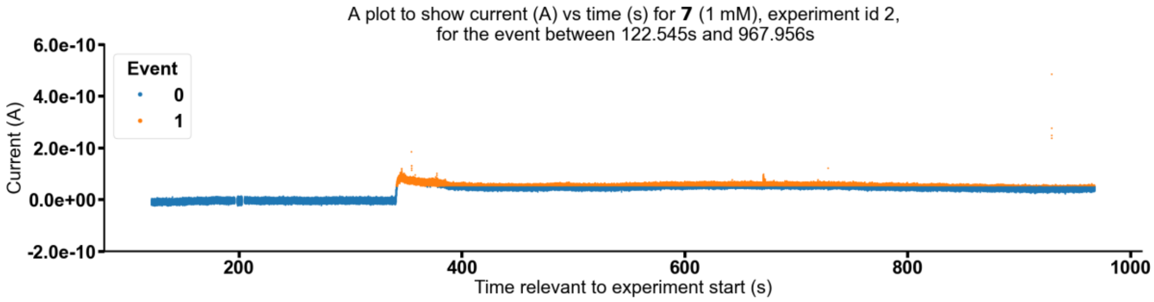

Figure S389 - Graph showing current (A) vs time (s) for **7** (1 mM), experiment id 2 at +100 mV, for the event between 122.545s and 967.956s.

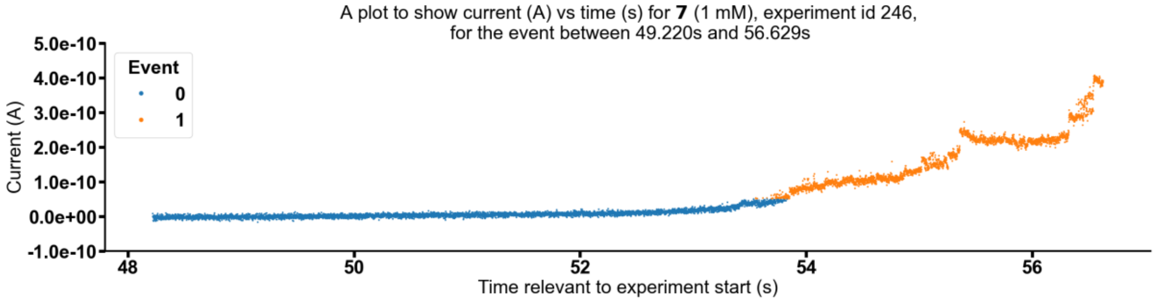

Figure S390 - Graph showing current (A) vs time (s) for **7** (1 mM), experiment id 246 at +100 mV, for the event between 49.220s and 56.629s.

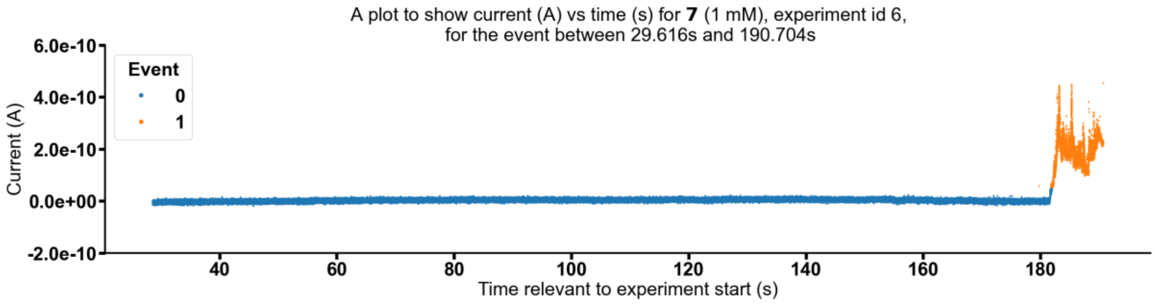

Figure S391 - Graph showing current (A) vs time (s) for **7** (1 mM), experiment id 6 at +100 mV, for the event between 29.616s and 190.704s.

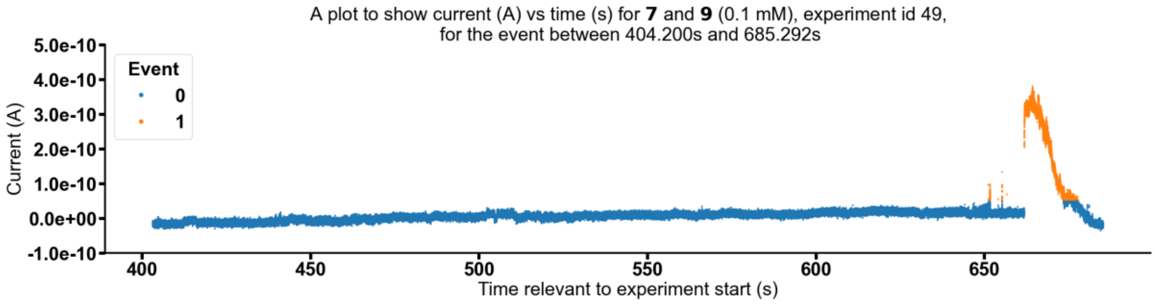

Figure S392 - Graph showing current (A) vs time (s) for **7** and **9** (0.1 mM), experiment id 49 at +100 mV, for the event between 404.200s and 685.292s.

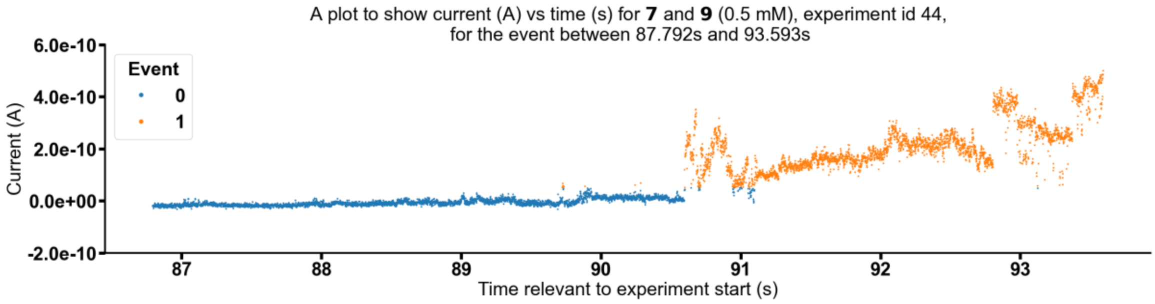

Figure S393 - Graph showing current (A) vs time (s) for **7** and **9** (0.5 mM), experiment id 44 at +100 mV, for the event between 87.792s and 93.593s.

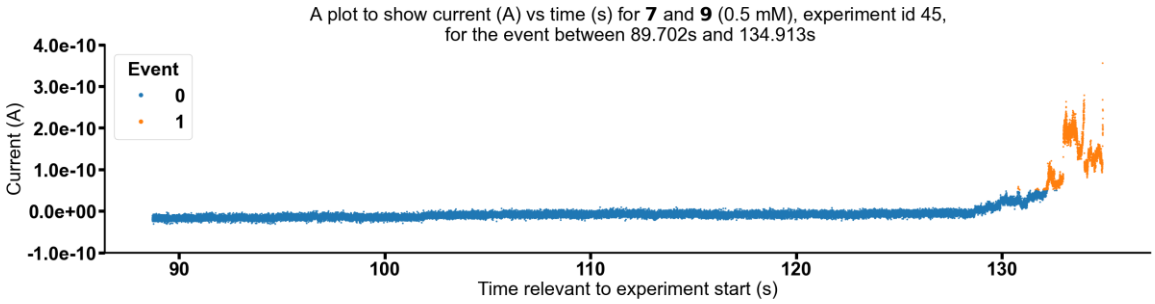

Figure S394 - Graph showing current (A) vs time (s) for **7** and **9** (0.5 mM), experiment id 45 at +100 mV, for the event between 89.702s and 134.913s.

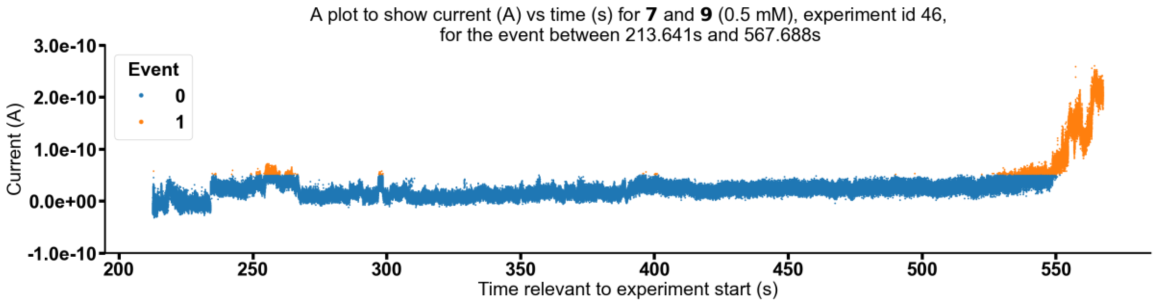

Figure S395 - Graph showing current (A) vs time (s) for **7** and **9** (0.5 mM), experiment id 46 at +100 mV, for the event between 213.641s and 567.688s.

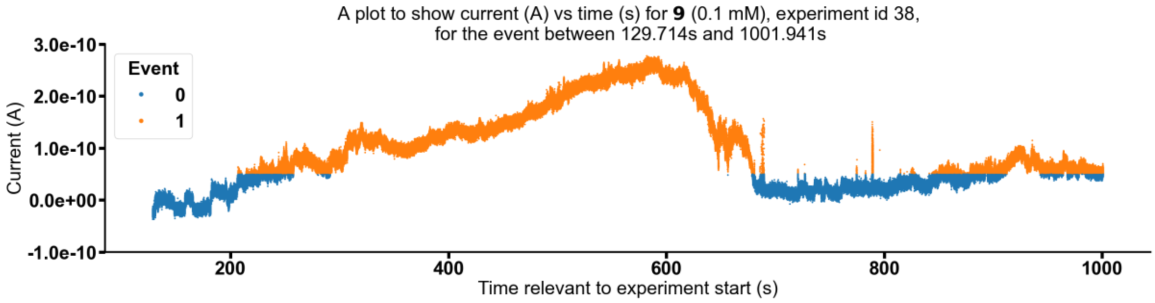

Figure S396 - Graph showing current (A) vs time (s) for **9** (0.1 mM), experiment id 38 at +100 mV, for the event between 129.714s and 1001.941s.

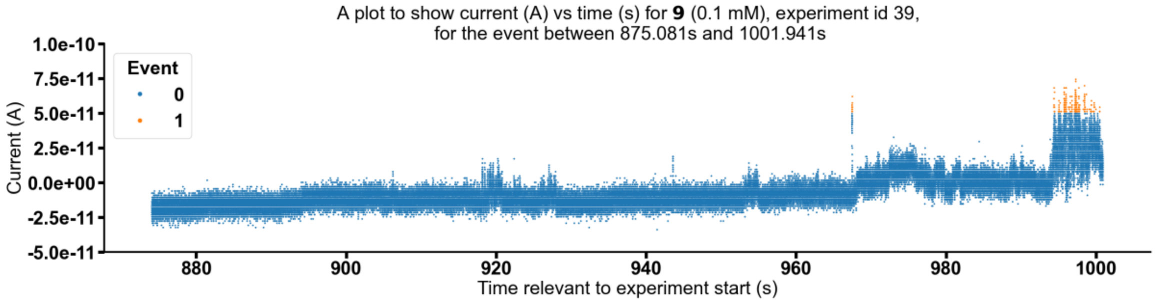

Figure S397 - Graph showing current (A) vs time (s) for **9** (0.1 mM), experiment id 39 at +100 mV, for the event between 875.081s and 1001.941s.

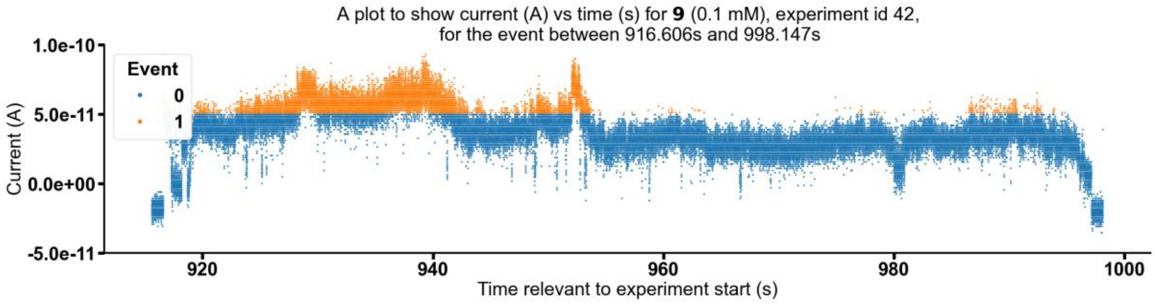

Figure S398 - Graph showing current (A) vs time (s) for **9** (0.1 mM), experiment id 42 at +100 mV, for the event between 916.606s and 998.147s.

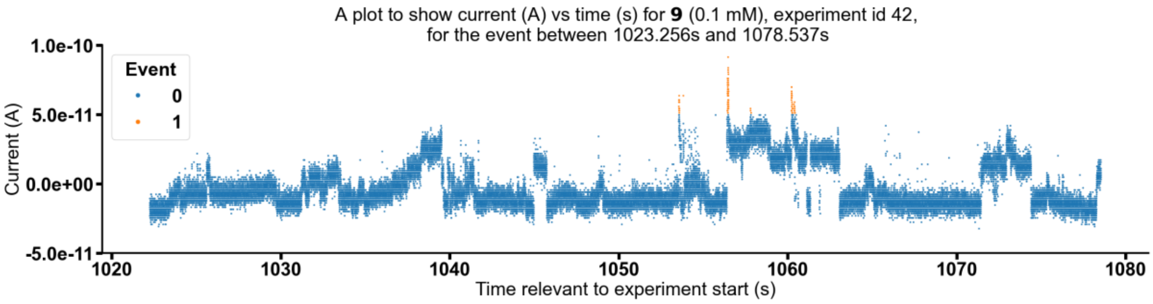

Figure S399 - Graph showing current (A) vs time (s) for **9** (0.1 mM), experiment id 42 at +100 mV, for the event between 1023.256s and 1078.537s.

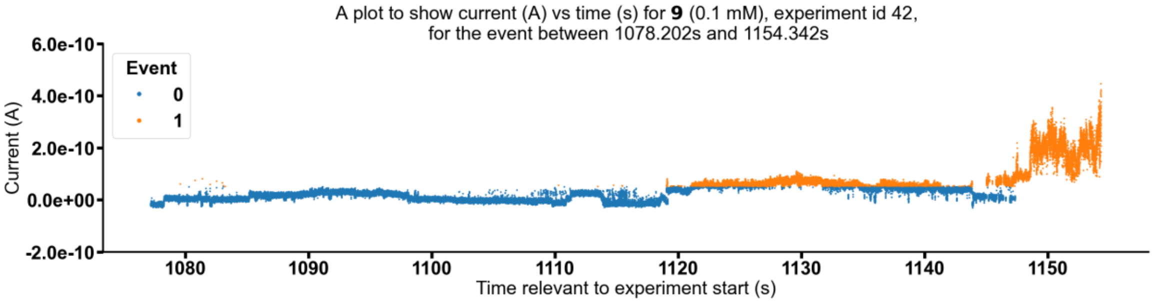

Figure S400 - Graph showing current (A) vs time (s) for **9** (0.1 mM), experiment id 42 at +100 mV, for the event between 1078.202s and 1154.342s.

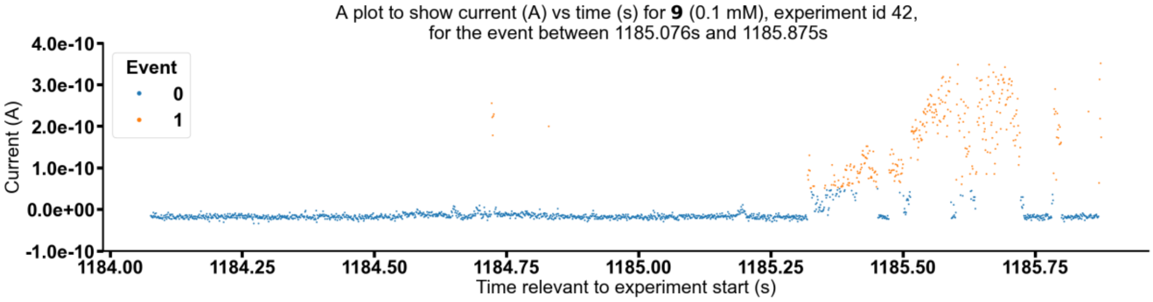

Figure S401 - Graph showing current (A) vs time (s) for **9** (0.1 mM), experiment id 42 at +100 mV, for the event between 1185.076s and 1185.875s.

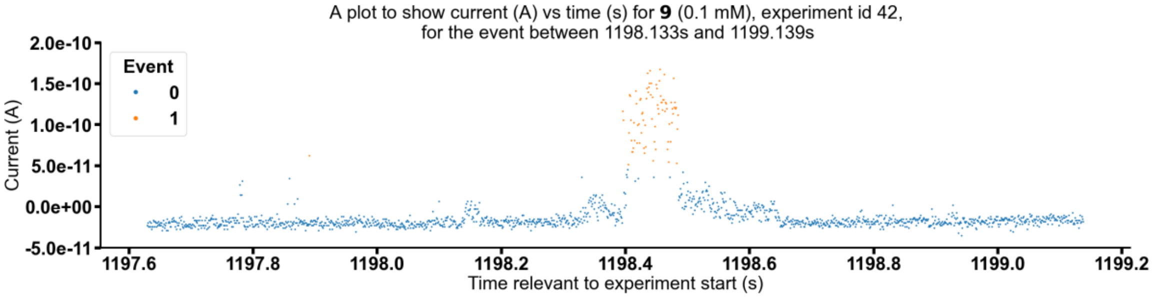

Figure S402 - Graph showing current (A) vs time (s) for **9** (0.1 mM), experiment id 42 at +100 mV, for the event between 1198.133s and 1199.139s.

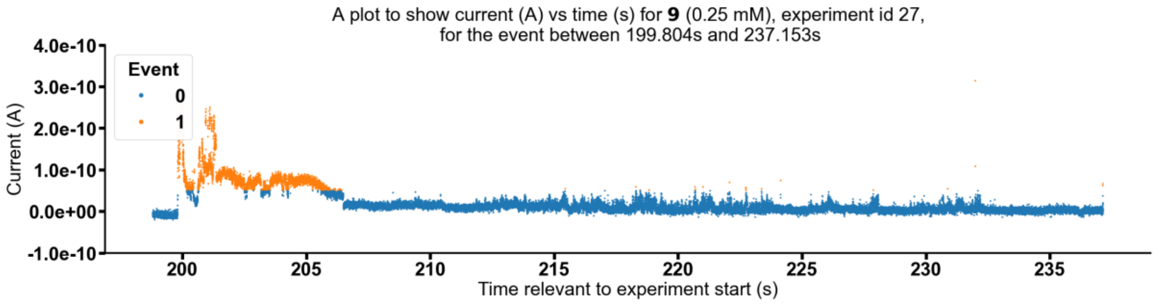

Figure S403 - Graph showing current (A) vs time (s) for **9** (0.25 mM), experiment id 27 at +100 mV, for the event between 199.804s and 237.153s.

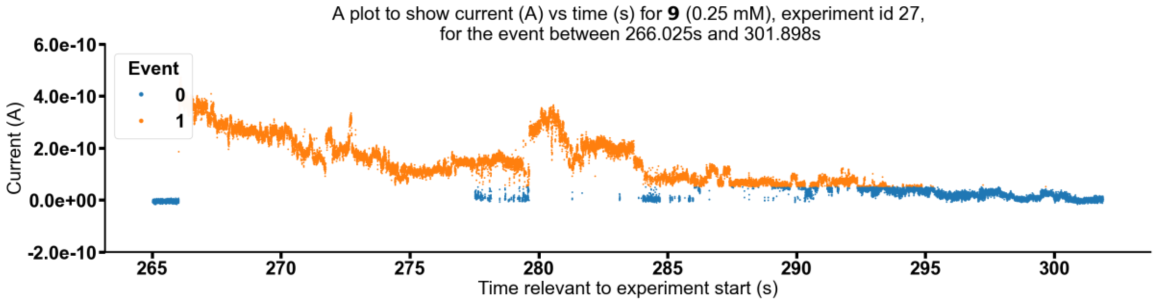

Figure S404 - Graph showing current (A) vs time (s) for **9** (0.25 mM), experiment id 27 at +100 mV, for the event between 266.025s and 301.898s.

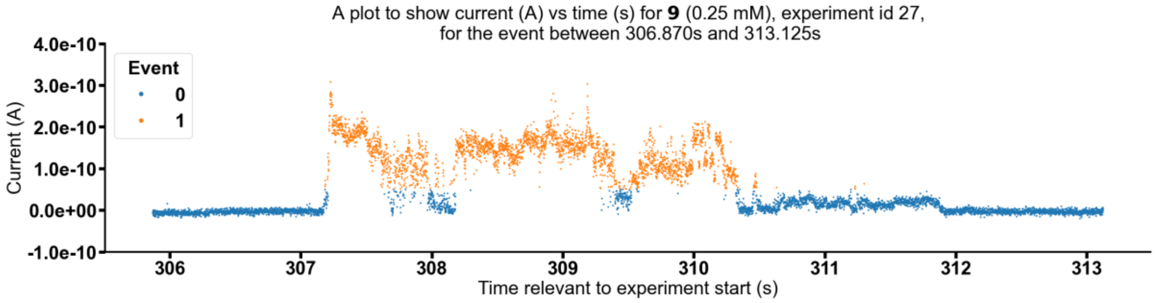

Figure S405 - Graph showing current (A) vs time (s) for **9** (0.25 mM), experiment id 27 at +100 mV, for the event between 306.870s and 313.125s.

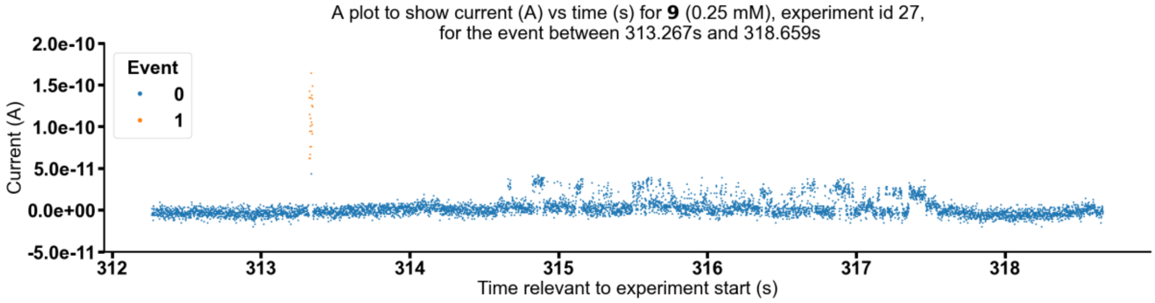

Figure S406 - Graph showing current (A) vs time (s) for **9** (0.25 mM), experiment id 27 at +100 mV, for the event between 313.267s and 318.659s.

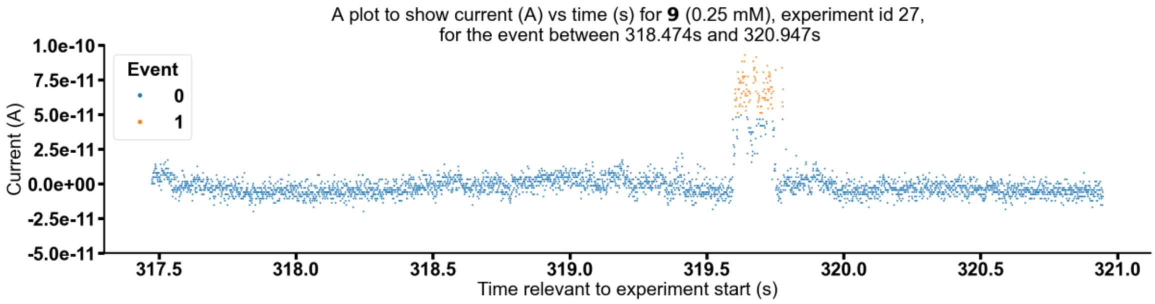

Figure S407 - Graph showing current (A) vs time (s) for **9** (0.25 mM), experiment id 27 at +100 mV, for the event between 318.474s and 320.947s.

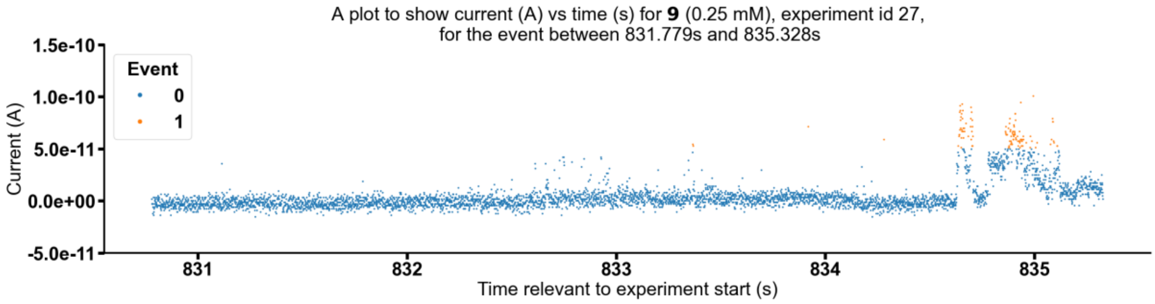

Figure S408 - Graph showing current (A) vs time (s) for **9** (0.25 mM), experiment id 27 at +100 mV, for the event between 831.779s and 835.328s.

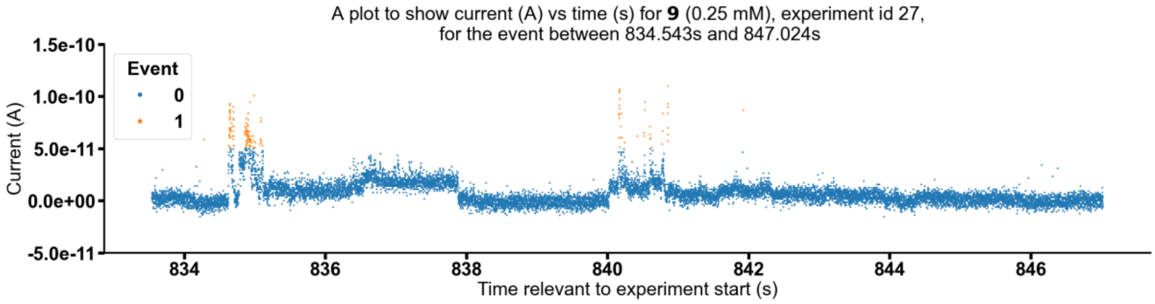

Figure S409 - Graph showing current (A) vs time (s) for **9** (0.25 mM), experiment id 27 at +100 mV, for the event between 834.543s and 847.024s.

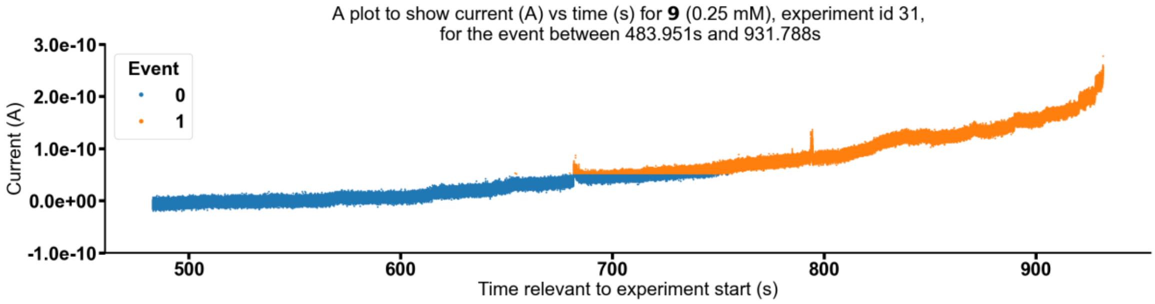

Figure S410 - Graph showing current (A) vs time (s) for **9** (0.25 mM), experiment id 31 at +100 mV, for the event between 483.951s and 931.788s.

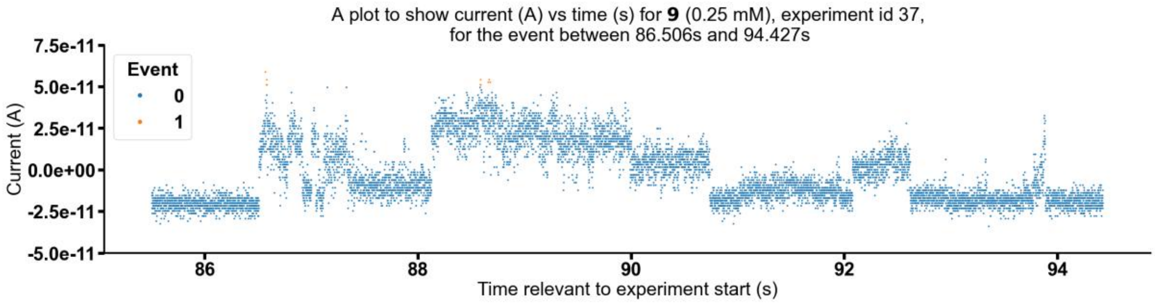

Figure S411 - Graph showing current (A) vs time (s) for **9** (0.25 mM), experiment id 37 at +100 mV, for the event between 86.506s and 94.427s.

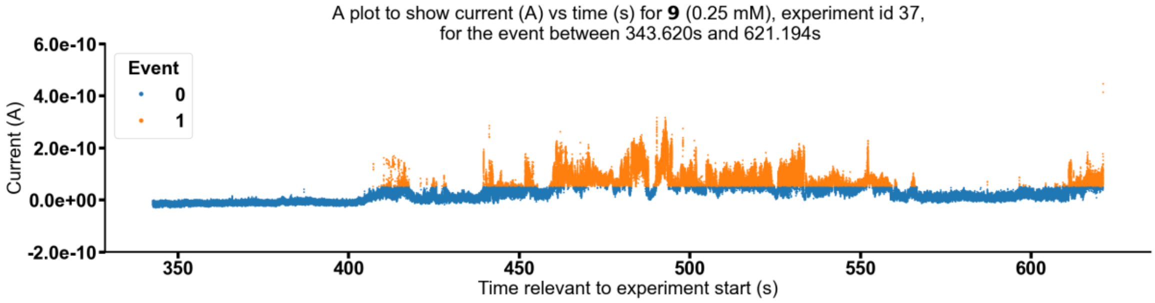

Figure S412 - Graph showing current (A) vs time (s) for **9** (0.25 mM), experiment id 37 at +100 mV, for the event between 343.620s and 621.194s.

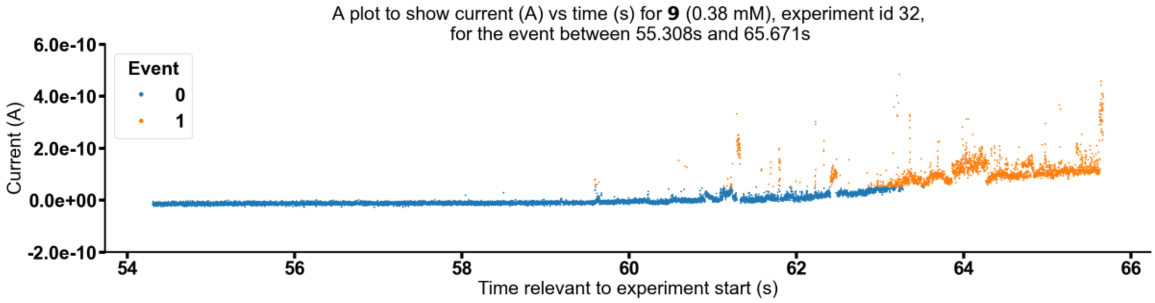

Figure S413 - Graph showing current (A) vs time (s) for **9** (0.38 mM), experiment id 32 at +100 mV, for the event between 55.308s and 65.671s.

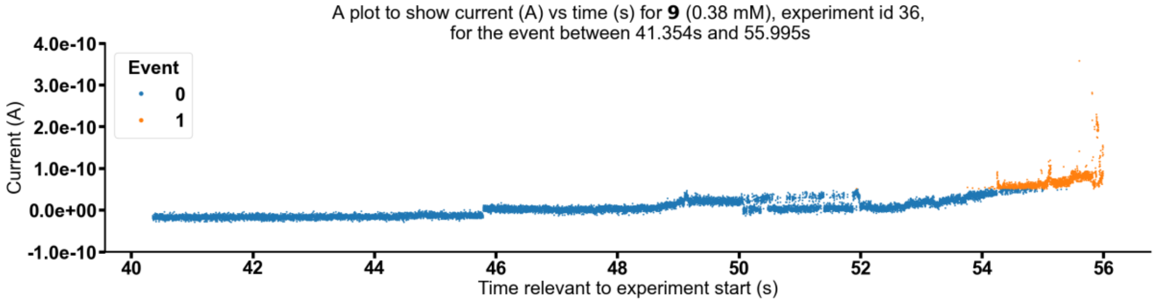

Figure S414 - Graph showing current (A) vs time (s) for **9** (0.38 mM), experiment id 36 at +100 mV, for the event between 41.354s and 55.995s.

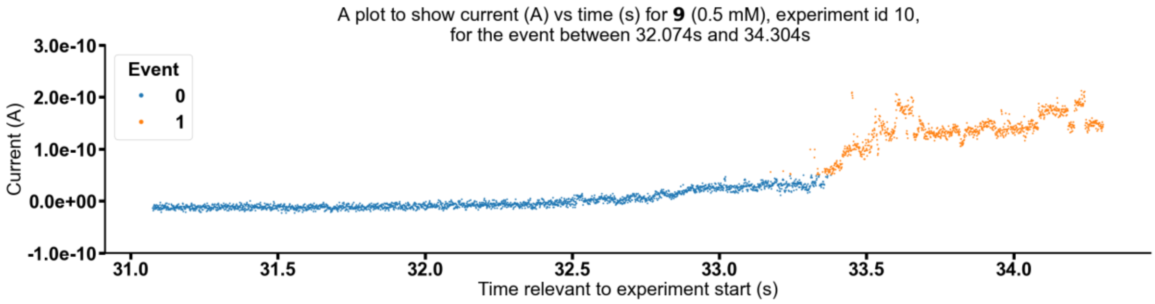

Figure S415 - Graph showing current (A) vs time (s) for **9** (0.5 mM), experiment id 10 at +100 mV, for the event between 32.074s and 34.304s.

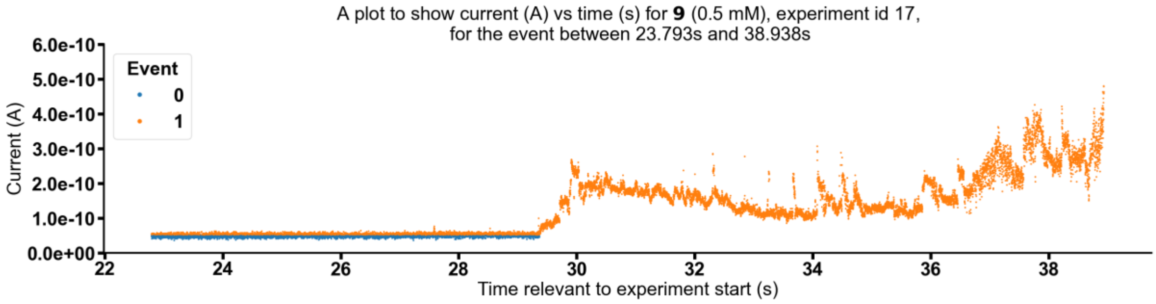

Figure S416 - Graph showing current (A) vs time (s) for **9** (0.5 mM), experiment id 17 at +100 mV, for the event between 23.793s and 38.938s.

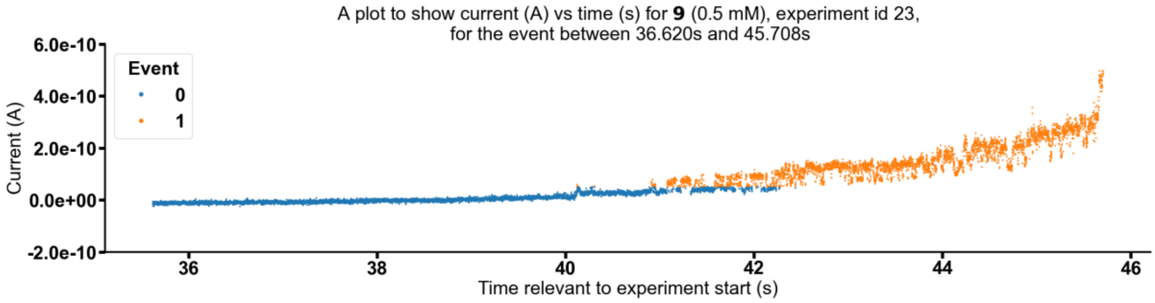

Figure S417 - Graph showing current (A) vs time (s) for **9** (0.5 mM), experiment id 23 at +100 mV, for the event between 36.620s and 45.708s.

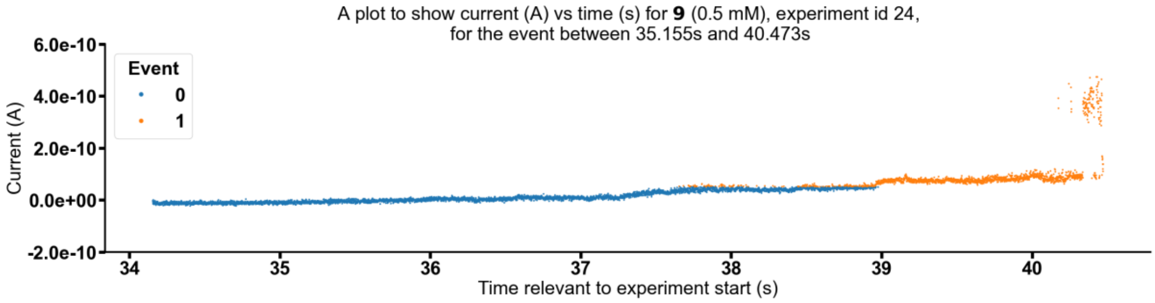

Figure S418 - Graph showing current (A) vs time (s) for **9** (0.5 mM), experiment id 24 at +100 mV, for the event between 35.155s and 40.473s.

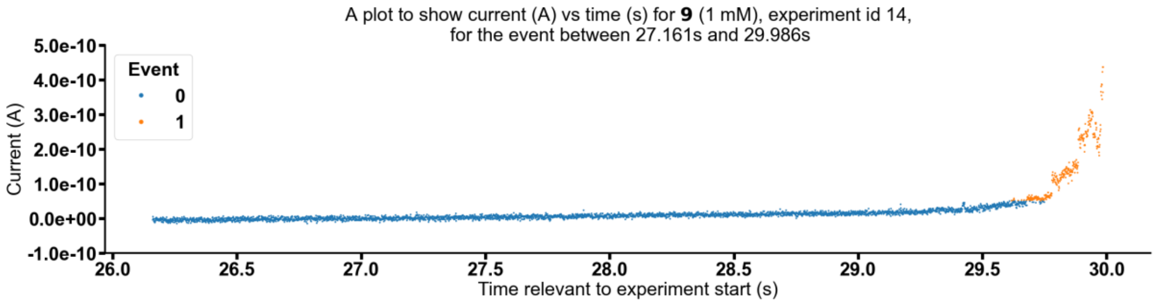

Figure S419 - Graph showing current (A) vs time (s) for **9** (1 mM), experiment id 14 at +100 mV, for the event between 27.161s and 29.986s.

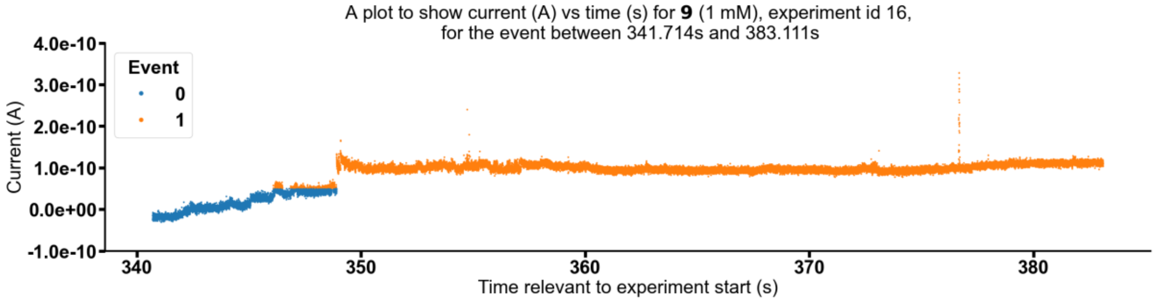

Figure S420 - Graph showing current (A) vs time (s) for **9** (1 mM), experiment id 16 at +100 mV, for the event between 341.714s and 383.111s.

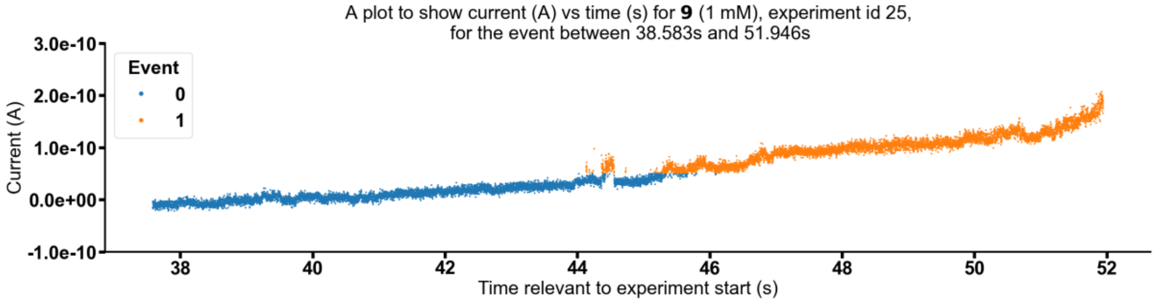

Figure S421 - Graph showing current (A) vs time (s) for **9** (1 mM), experiment id 25 at +100 mV, for the event between 38.583s and 51.946s.

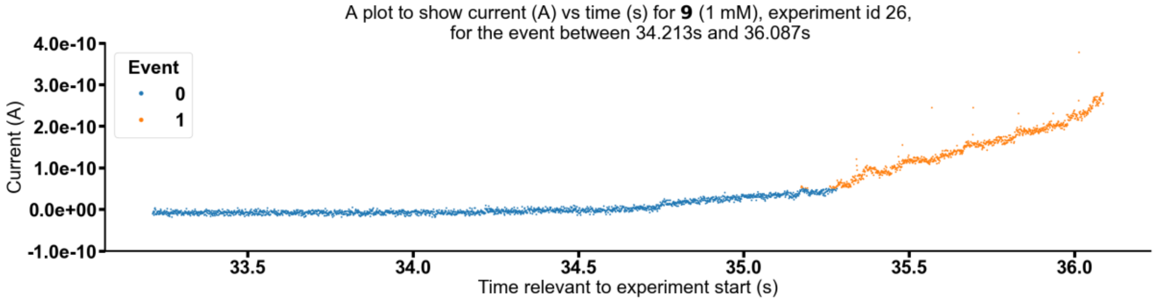

Figure S422 - Graph showing current (A) vs time (s) for **9** (1 mM), experiment id 26 at +100 mV, for the event between 34.213s and 36.087s.

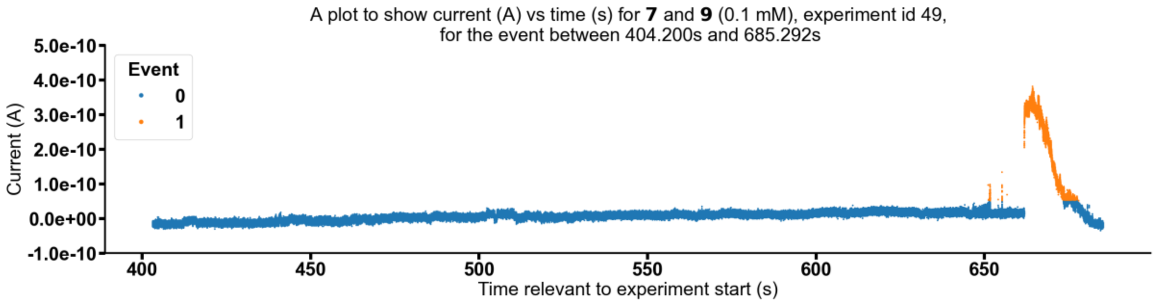

Figure S423 - Graph showing current (A) vs time (s) for **7** and **9** (0.1 mM), experiment id 49 at +100 mV, for the event between 404.200s and 685.292s.

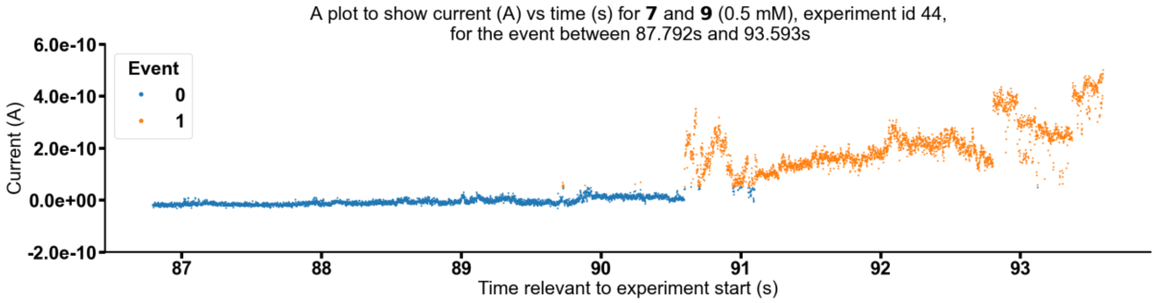

Figure S424 - Graph showing current (A) vs time (s) for **7** and **9** (0.5 mM), experiment id 44 at +100 mV, for the event between 87.792s and 93.593s.

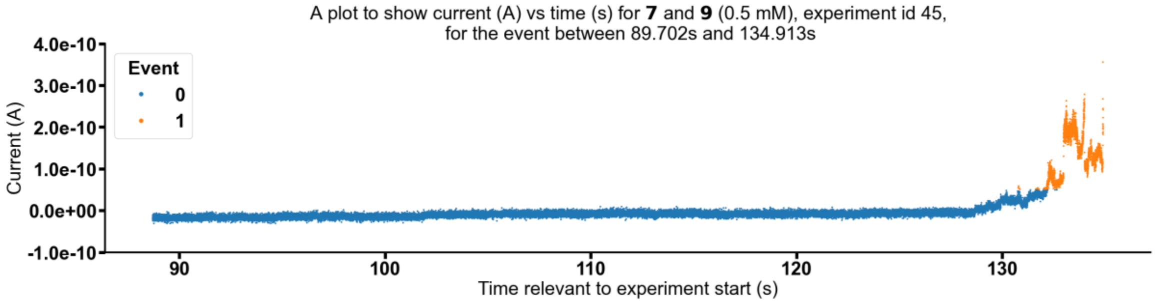

Figure S425 - Graph showing current (A) vs time (s) for **7** and **9** (0.5 mM), experiment id 45 at +100 mV, for the event between 89.702s and 134.913s.

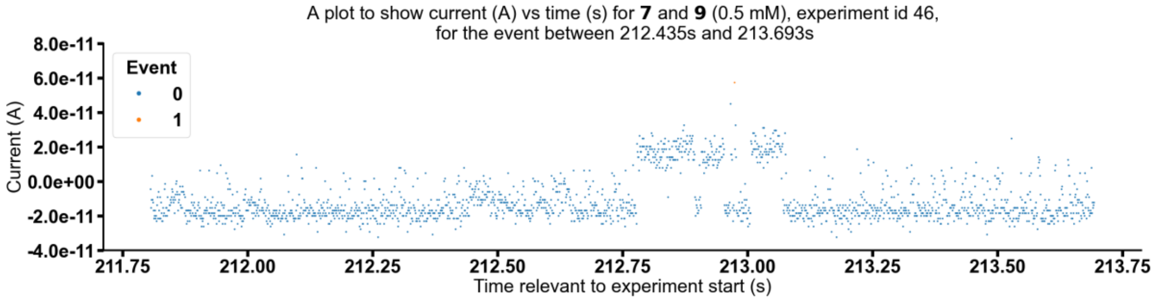

Figure S426 - Graph showing current (A) vs time (s) for **7** and **9** (0.5 mM), experiment id 46 at +100 mV, for the event between 212.435s and 213.693s.

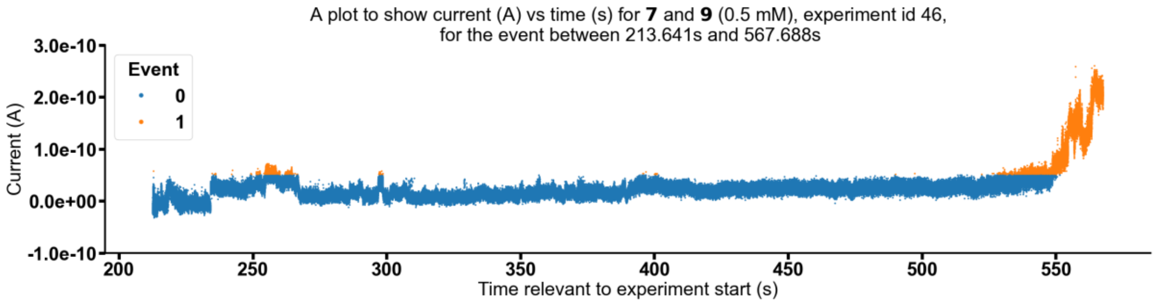

Figure S427 - Graph showing current (A) vs time (s) for **7** and **9** (0.5 mM), experiment id 46 at +100 mV, for the event between 213.641s and 567.688s.

Data analysed magnified portions of patch clamp data sets

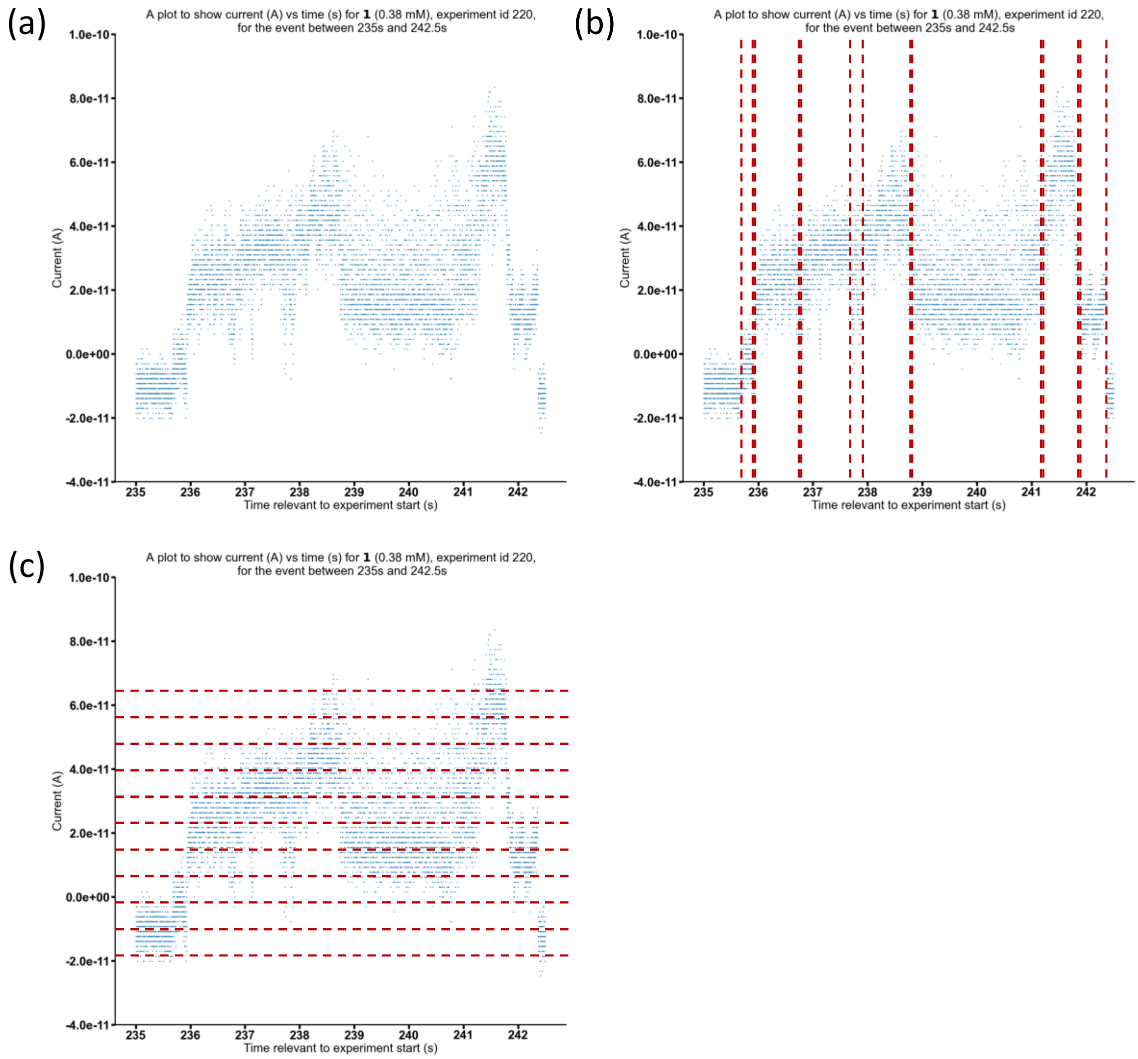

Figure S428– Graph plots of current (A) vs time (s) for **1** (0.38 mM): (a) see graph title; (b) same as (a) but illustrates event time; (c) same as (a) but illustrates event magnitude.

(a)

(b)

(c)

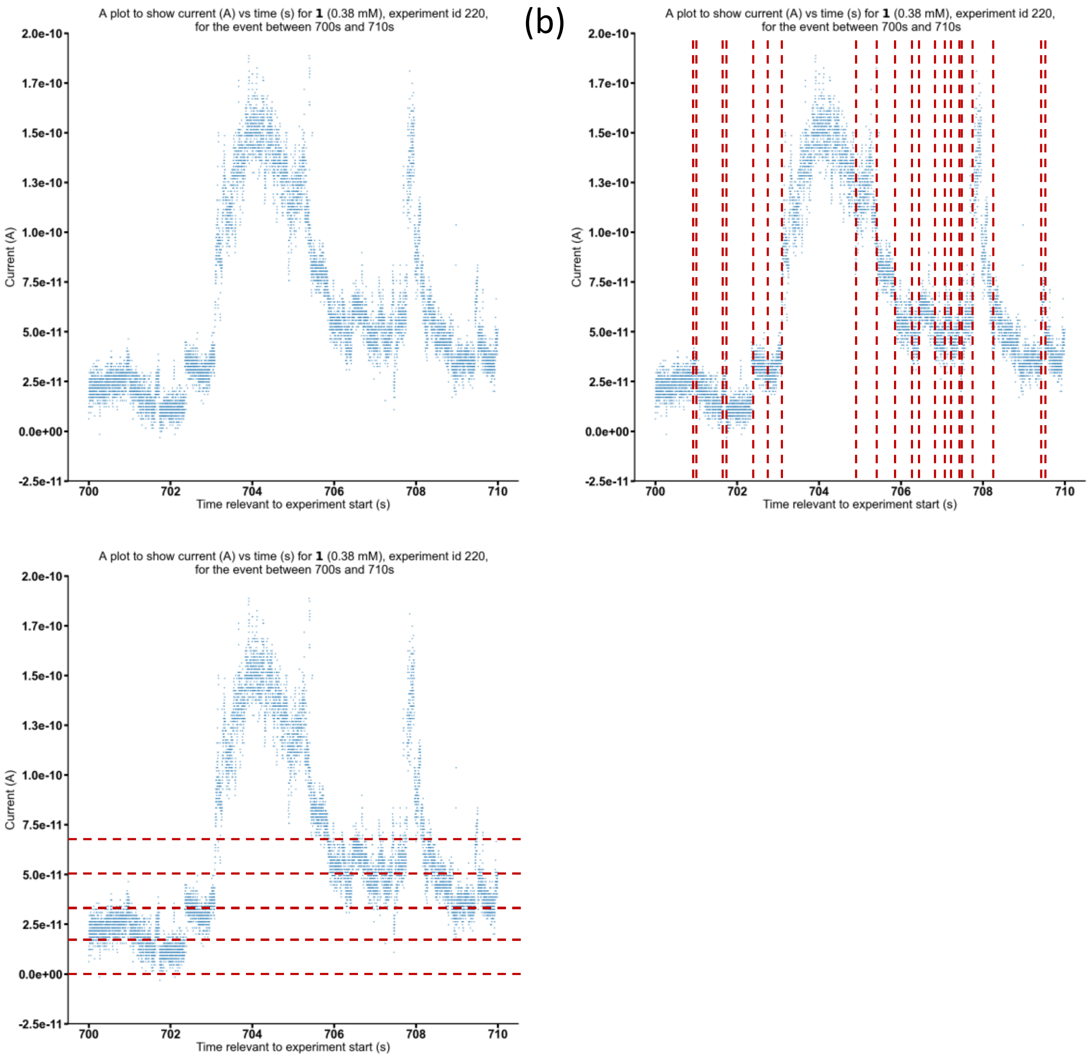

Figure S429 – Graph plots of current (A) vs time (s) for **1** (0.38 mM): (a) see graph title; (b) same as (a) but illustrates event time; (c) same as (a) but illustrates event magnitude.

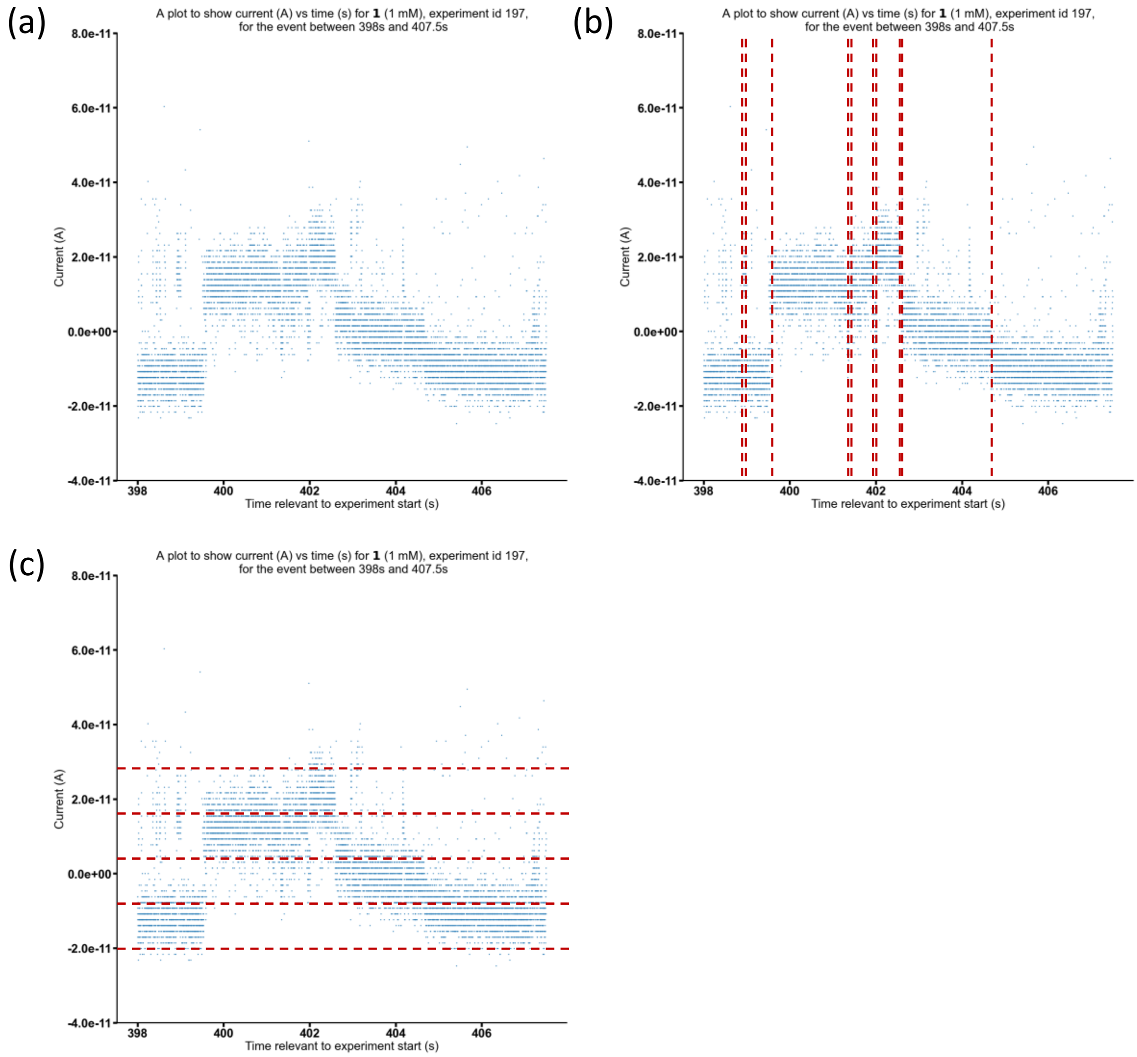

Figure S430 – Graph plots of current (A) vs time (s) for **1** (1 mM): (a) see graph title; (b) same as (a) but illustrates event time; (c) same as (a) but illustrates event magnitude.

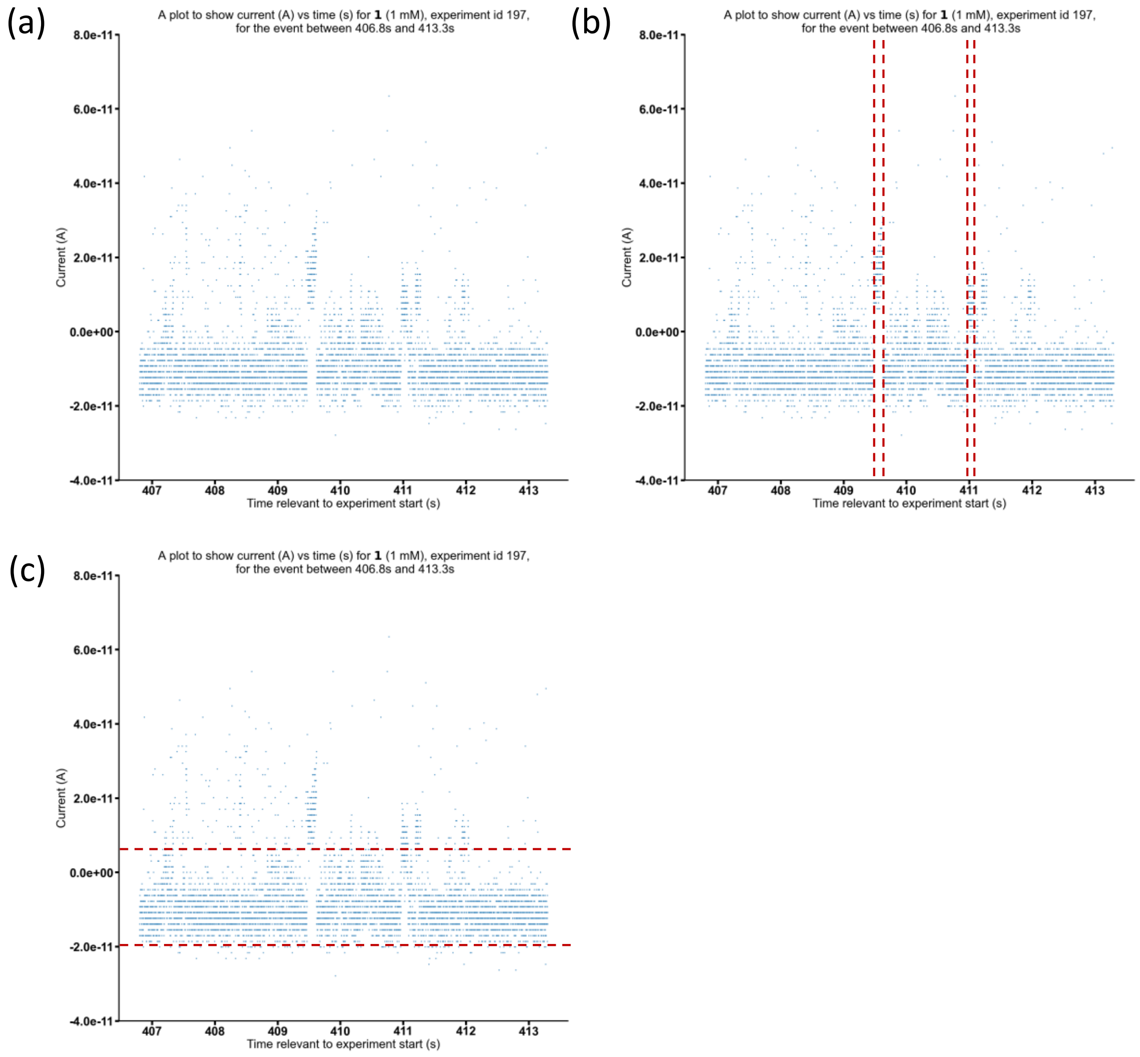

Figure S431 – Graph plots of current (A) vs time (s) for **1** (1 mM): (a) see graph title; (b) same as (a) but illustrates event time; (c) same as (a) but illustrates event magnitude.

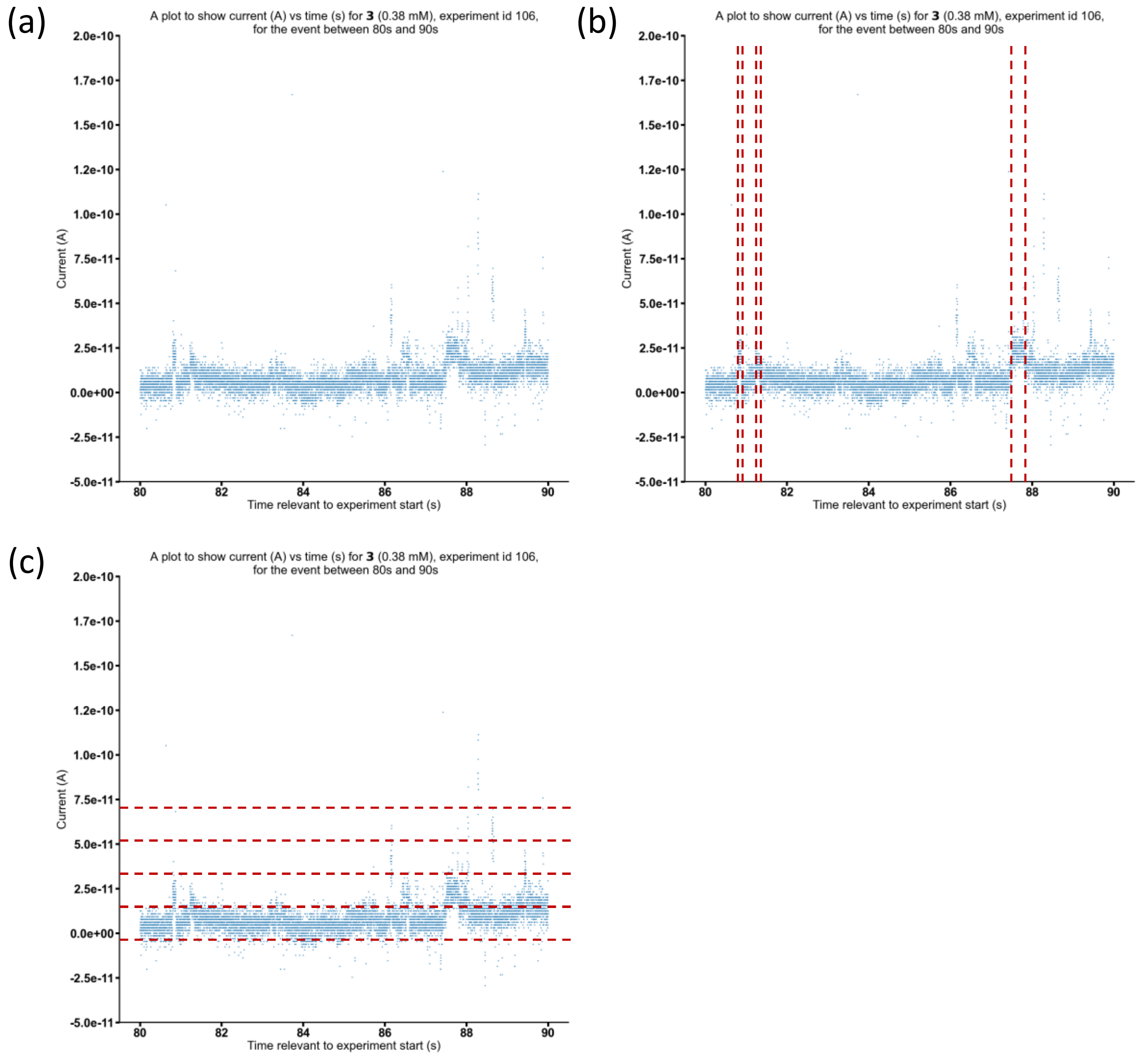

Figure S432 – Graph plots of current (A) vs time (s) for **3** (0.38 mM): (a) see graph title; (b) same as (a) but illustrates event time; (c) same as (a) but illustrates event magnitude.

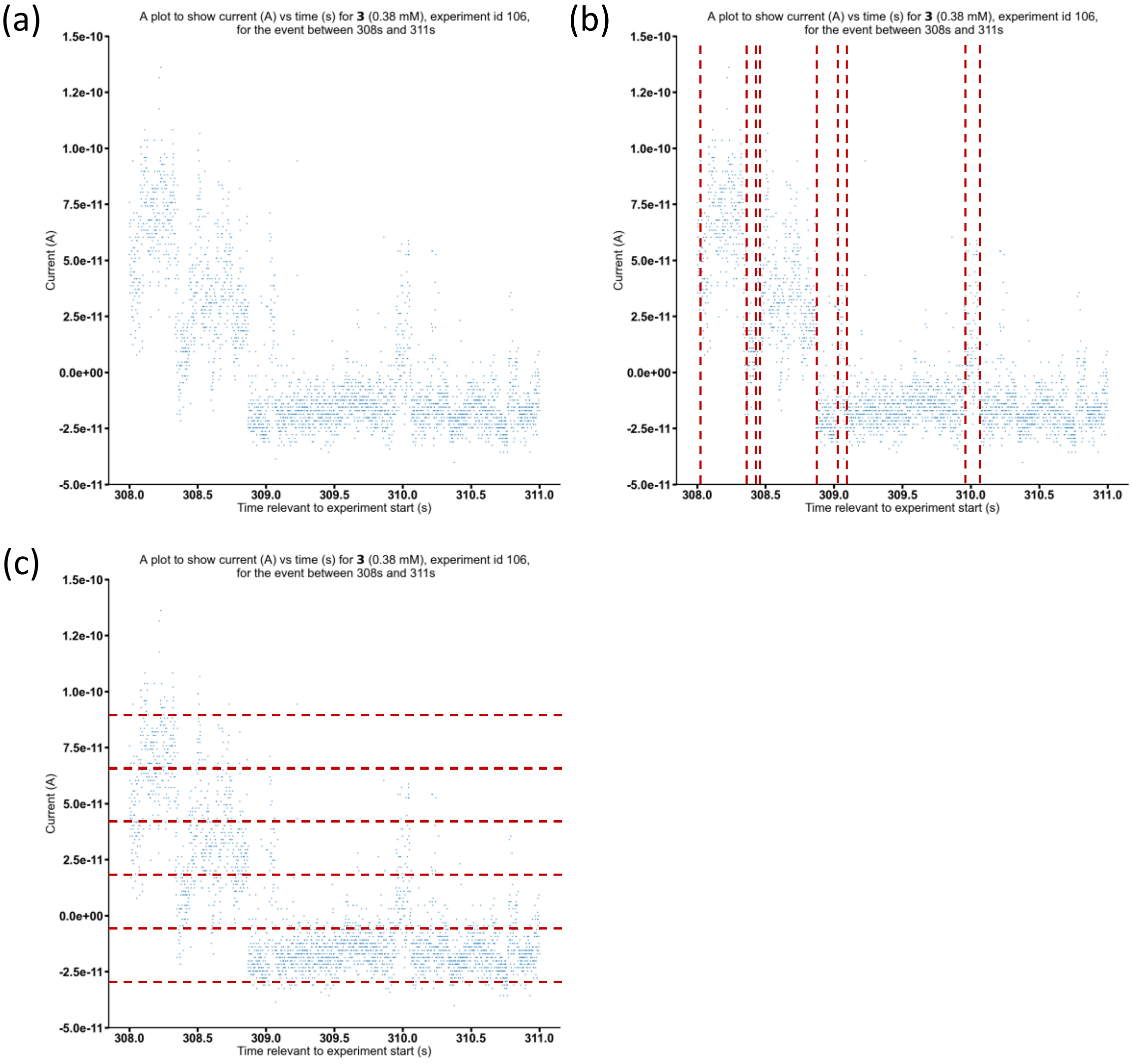

Figure S433 – Graph plots of current (A) vs time (s) for **3** (0.38 mM): (a) see graph title; (b) same as (a) but illustrates event time; (c) same as (a) but illustrates event magnitude.

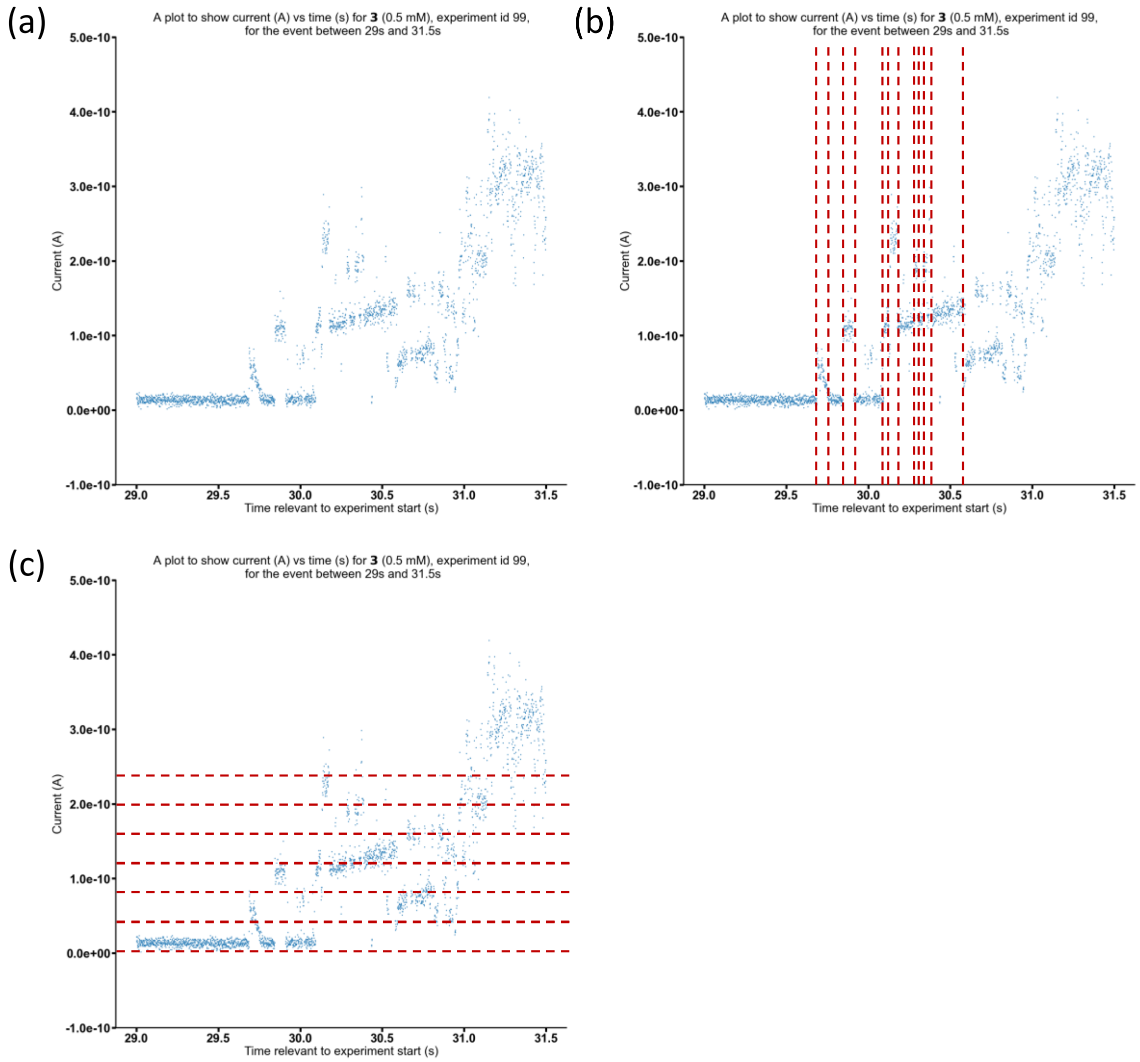

Figure S434 – Graph plots of current (A) vs time (s) for **3** (0.5 mM): (a) see graph title; (b) same as (a) but illustrates event time; (c) same as (a) but illustrates event magnitude.

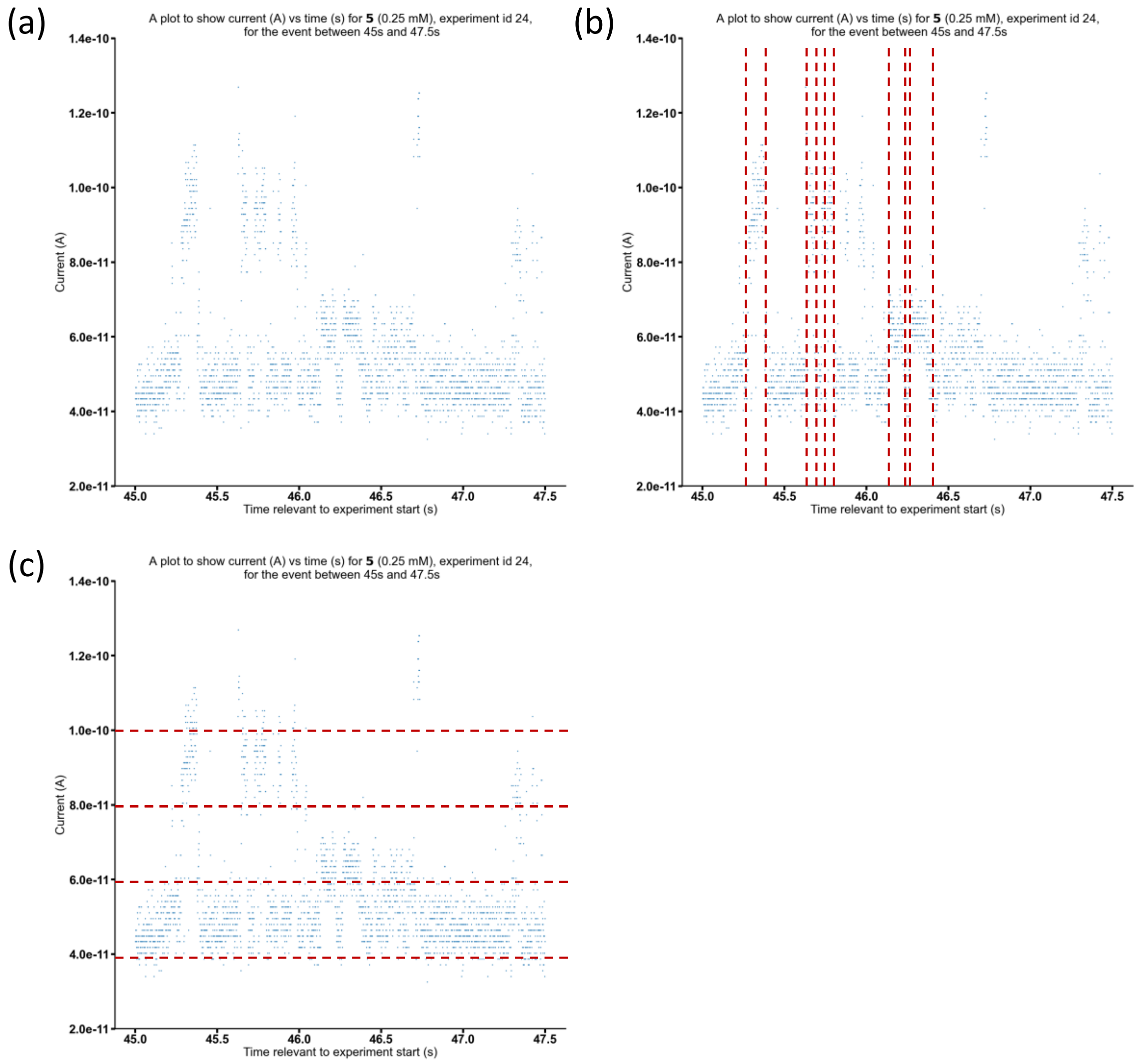

Figure S435 – Graph plots of current (A) vs time (s) for **5** (0.25 mM): (a) see graph title; (b) same as (a) but illustrates event time; (c) same as (a) but illustrates event magnitude.

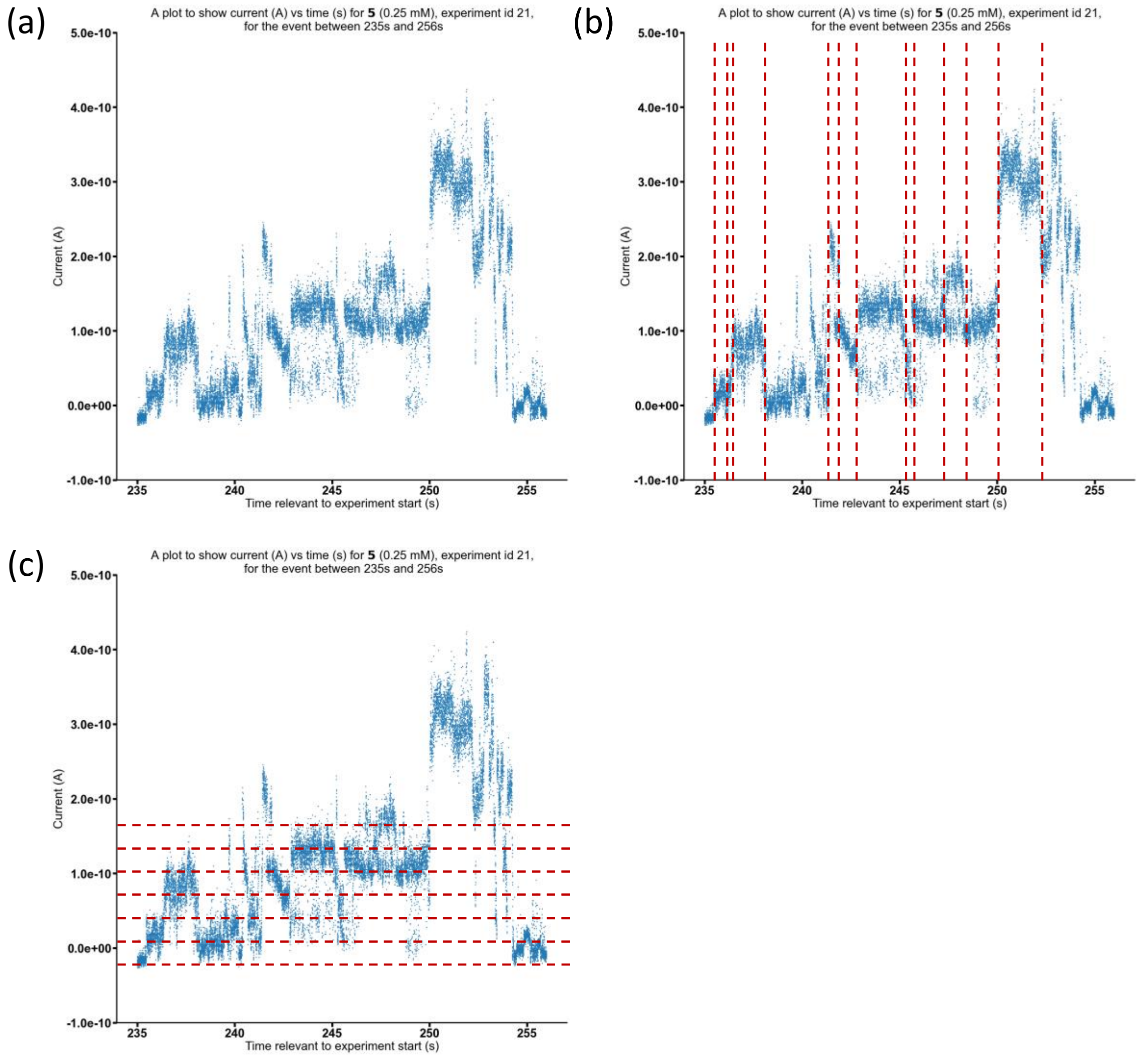

Figure S436 – Graph plots of current (A) vs time (s) for **5** (0.25 mM): (a) see graph title; (b) same as (a) but illustrates event time; (c) same as (a) but illustrates event magnitude.

(a)

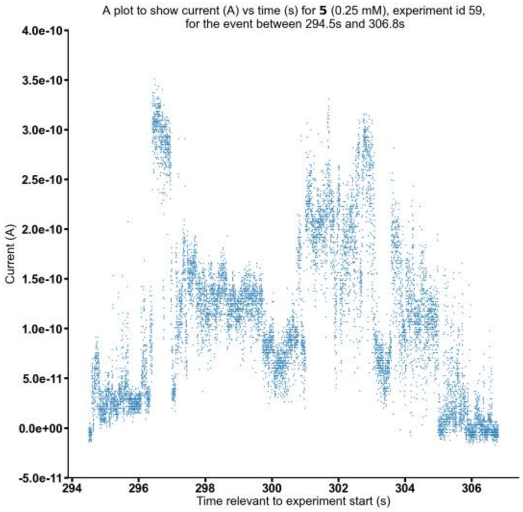

(b)

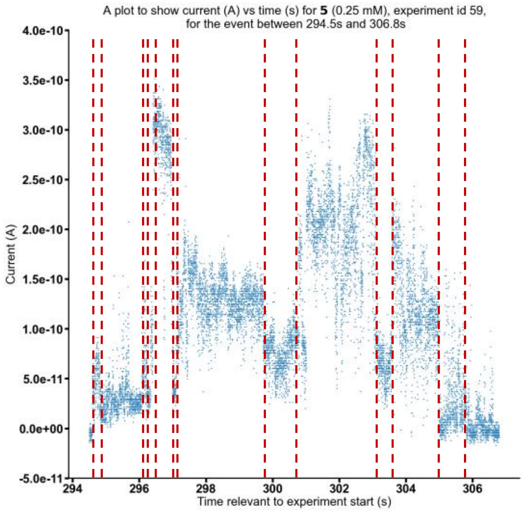

(c)

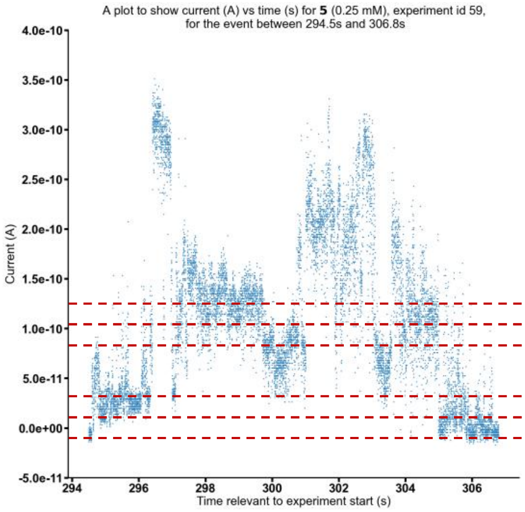

Figure S437 – Graph plots of current (A) vs time (s) for **5** (0.25 mM): (a) see graph title; (b) same as (a) but illustrates event time; (c) same as (a) but illustrates event magnitude.

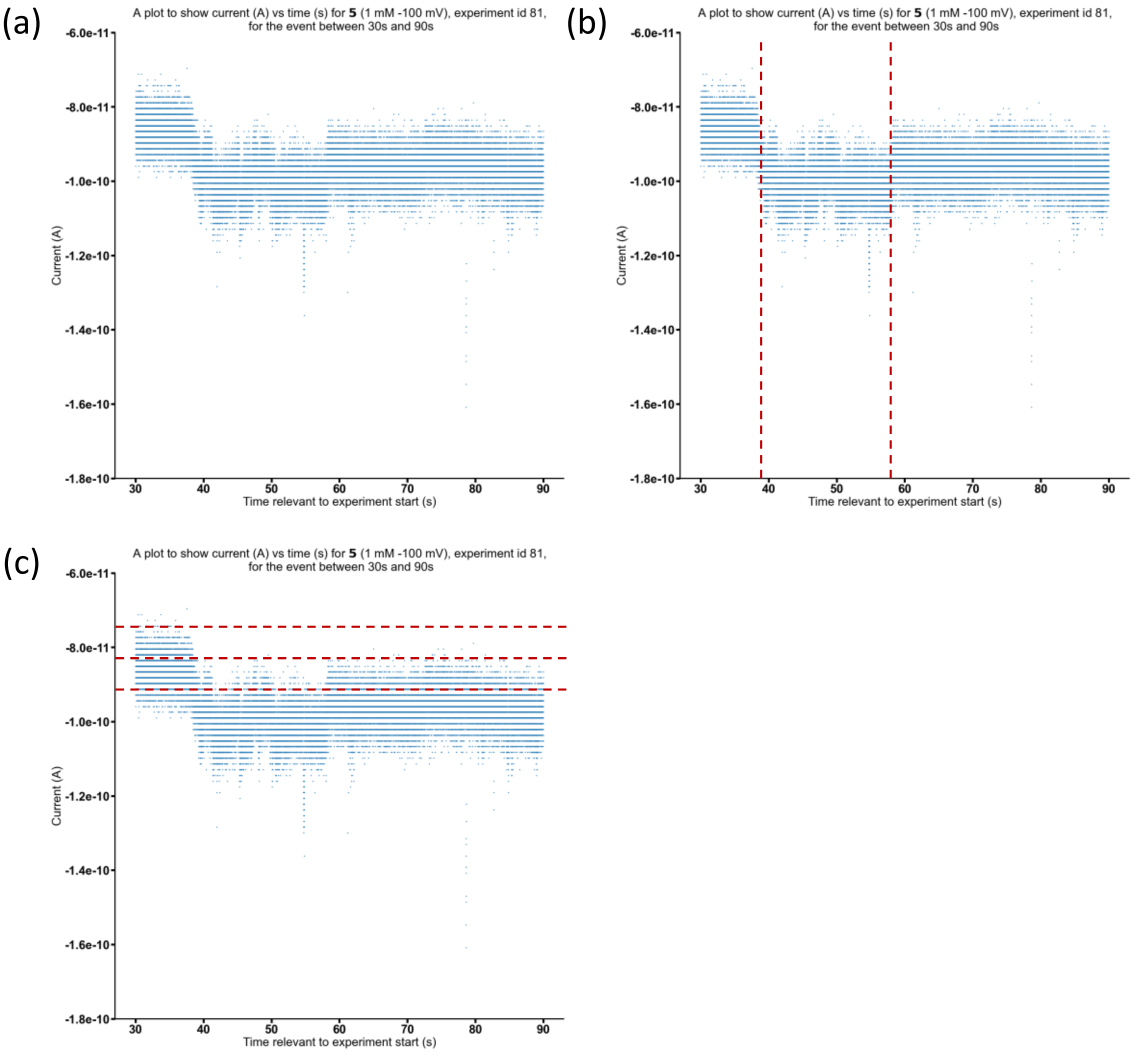

Figure S438 – Graph plots of current (A) vs time (s) for **5** (1 mM): (a) see graph title; (b) same as (a) but illustrates event time; (c) same as (a) but illustrates event magnitude.

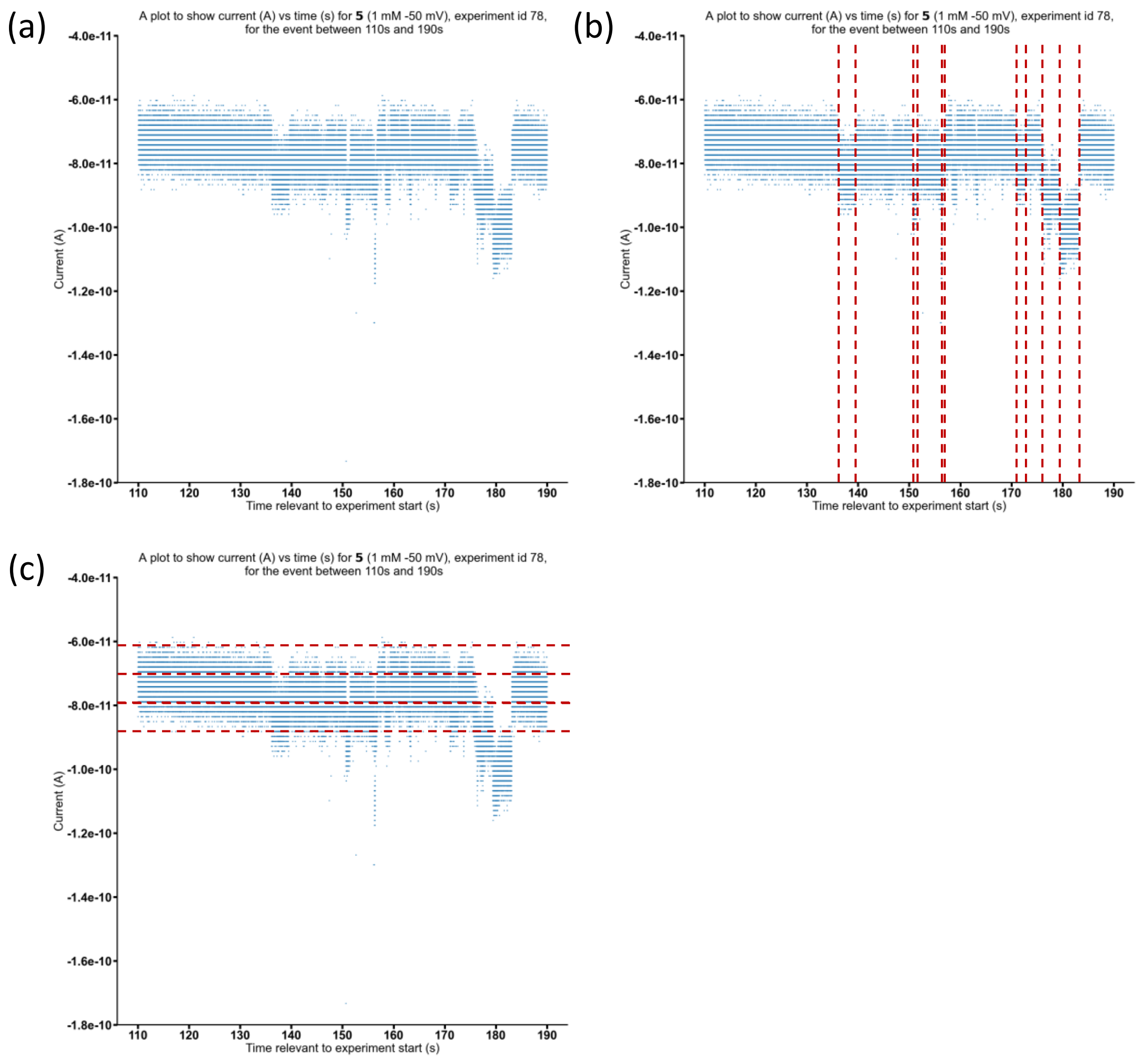

Figure S439 – Graph plots of current (A) vs time (s) for **5** (1 mM): (a) see graph title; (b) same as (a) but illustrates event time; (c) same as (a) but illustrates event magnitude.

(a) (b) (c)

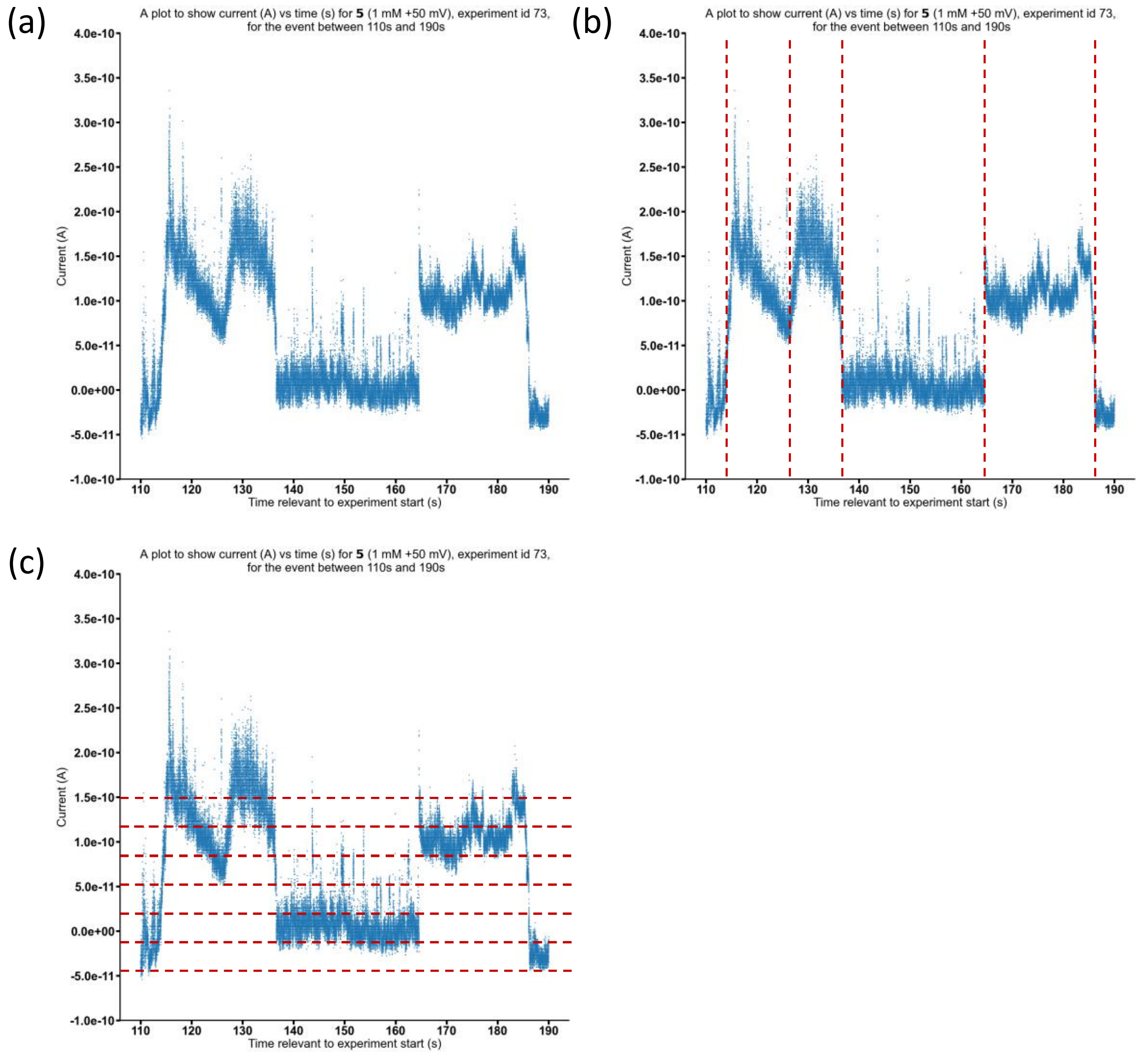

Figure S440 – Graph plots of current (A) vs time (s) for **5** (1 mM): (a) see graph title; (b) same as (a) but illustrates event time; (c) same as (a) but illustrates event magnitude.

(a) (b) (c)

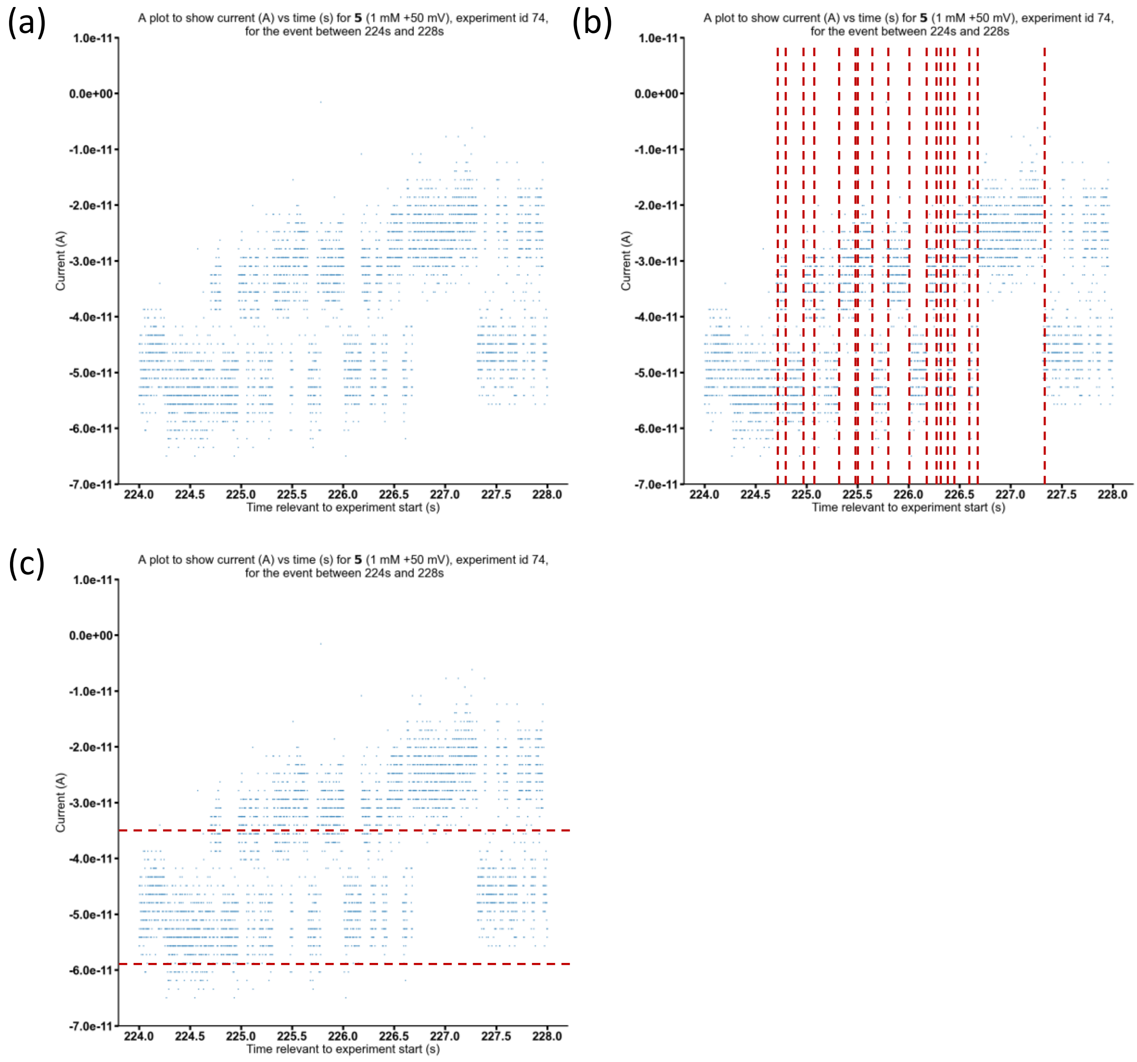

Figure S441 – Graph plots of current (A) vs time (s) for **5** (1 mM): (a) see graph title; (b) same as (a) but illustrates event time; (c) same as (a) but illustrates event magnitude.

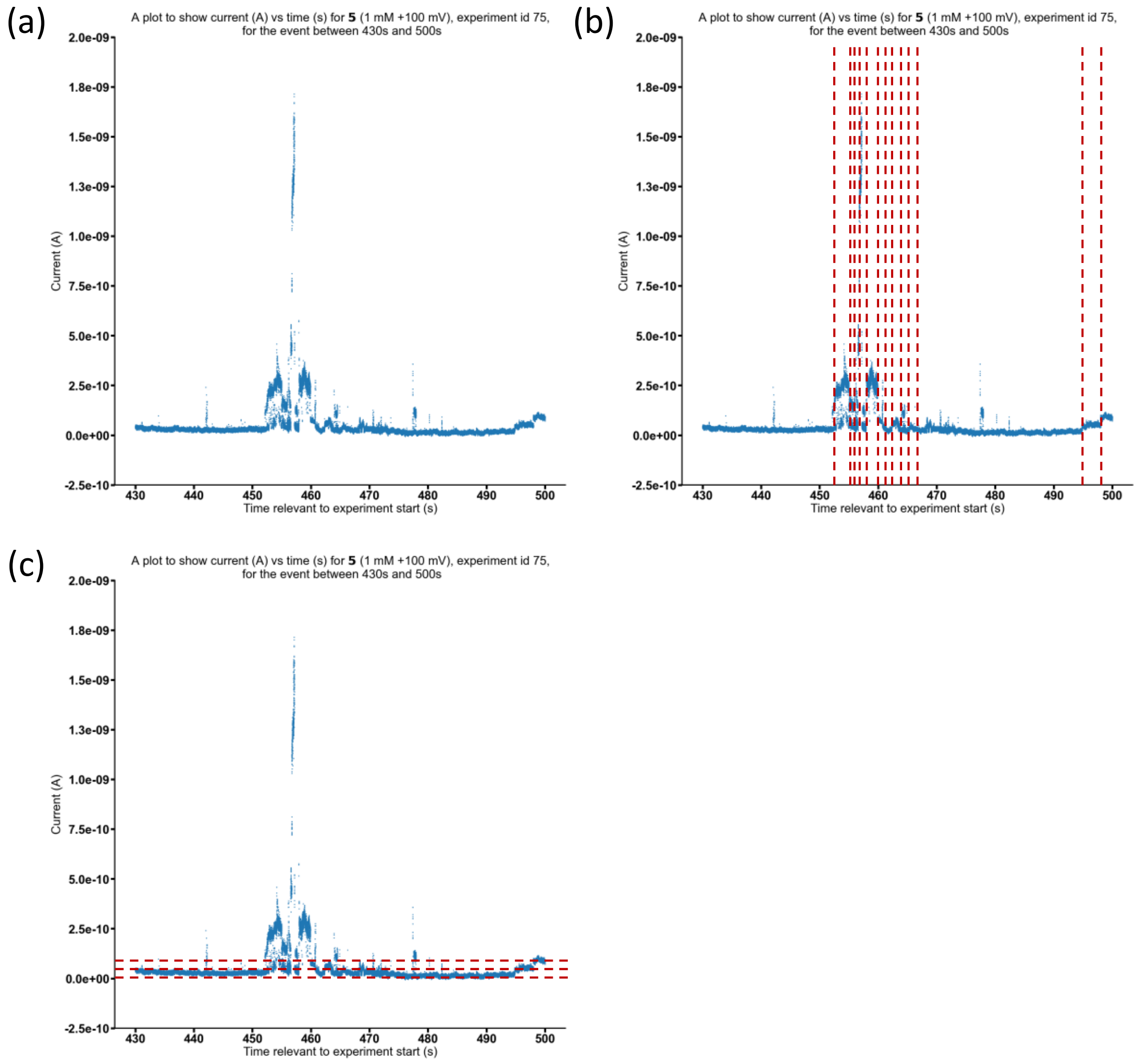

Figure S442 – Graph plots of current (A) vs time (s) for **5** (1 mM): (a) see graph title; (b) same as (a) but illustrates event time; (c) same as (a) but illustrates event magnitude.

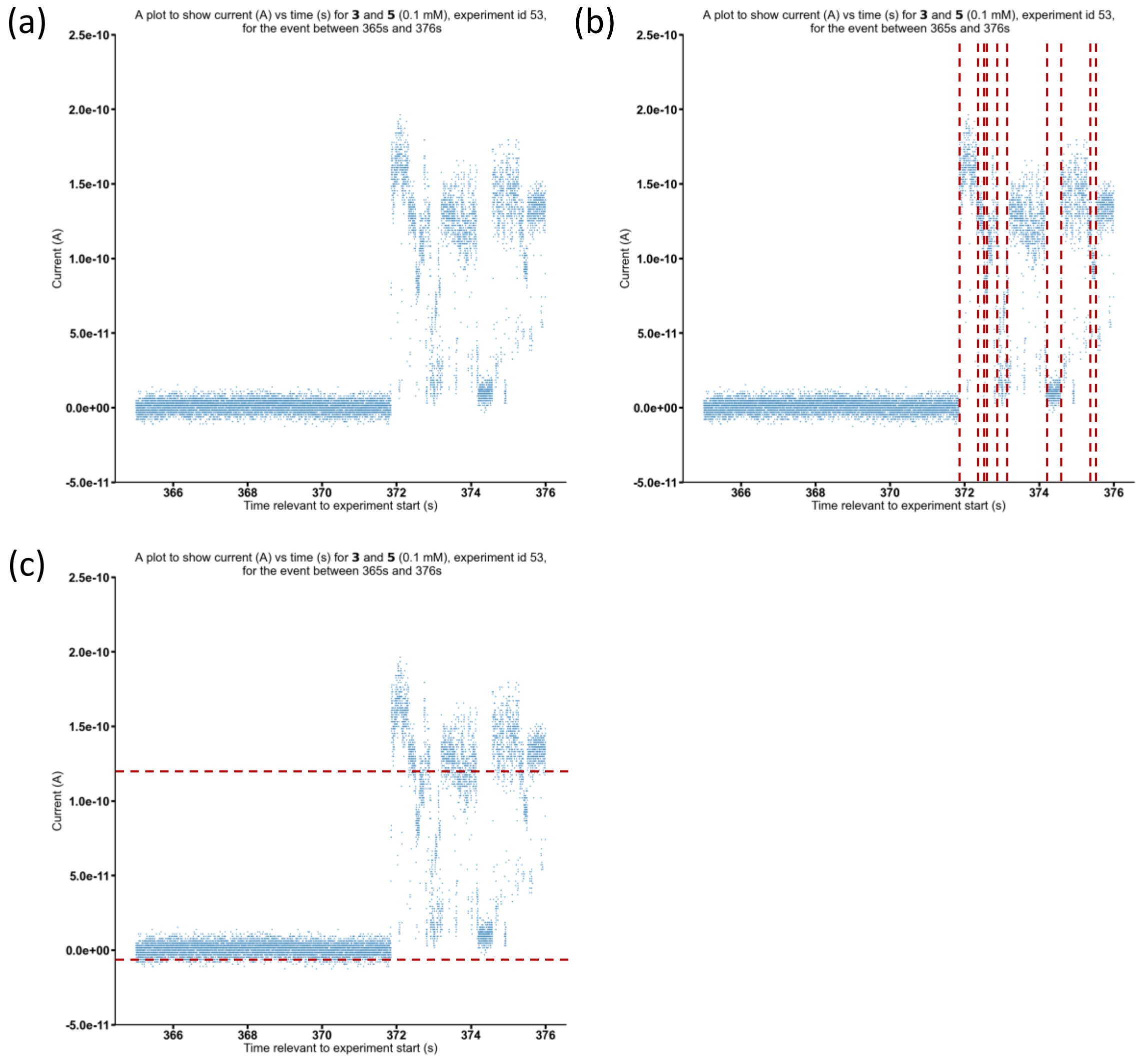

Figure S443 – Graph plots of current (A) vs time (s) for **3** and **5** (0.1 mM): (a) see graph title; (b) same as (a) but illustrates event time; (c) same as (a) but illustrates event magnitude.

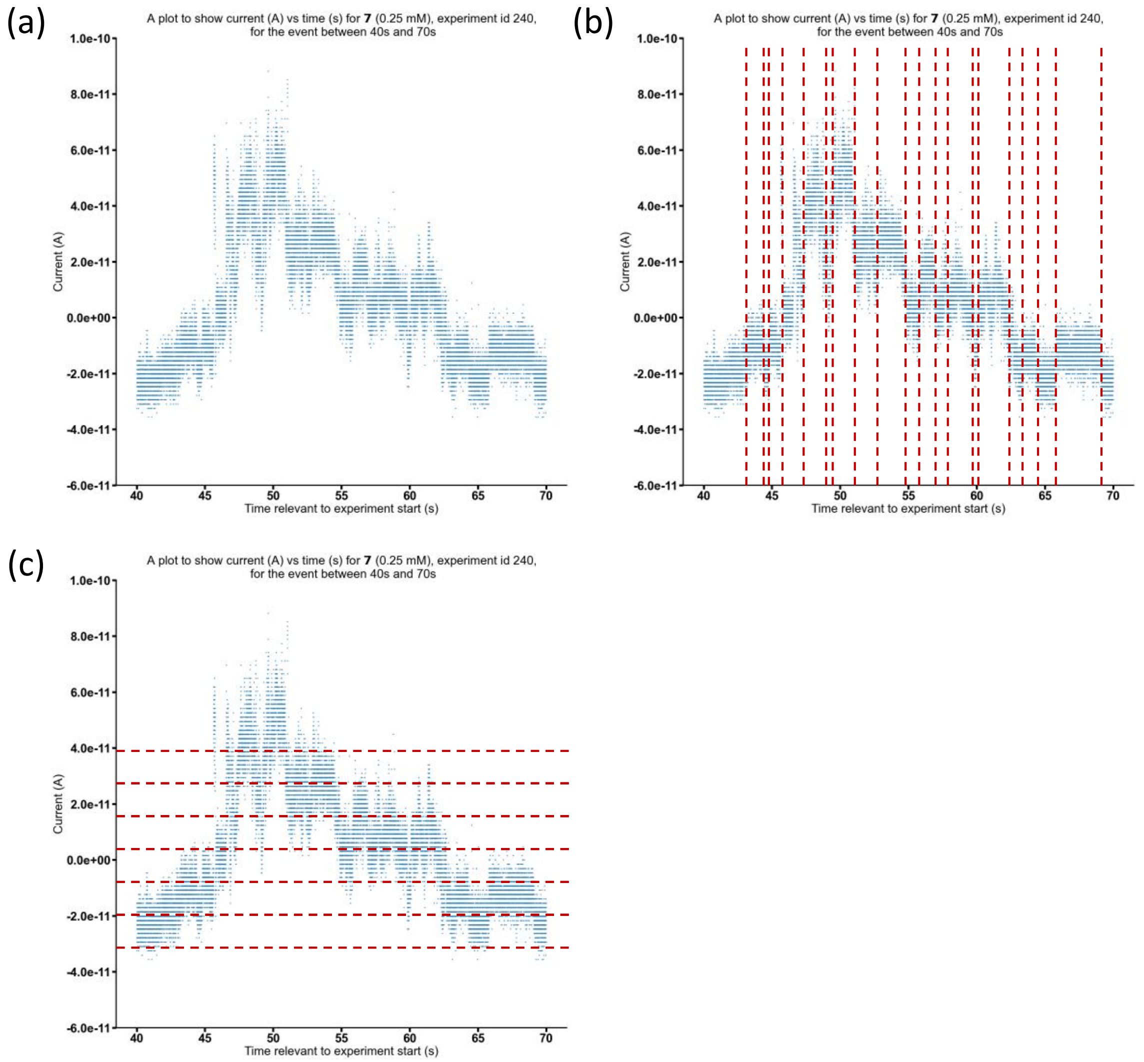

Figure S444 – Graph plots of current (A) vs time (s) for **7** (0.25 mM): (a) see graph title; (b) same as (a) but illustrates event time; (c) same as (a) but illustrates event magnitude.

(a)

(b)

(c)

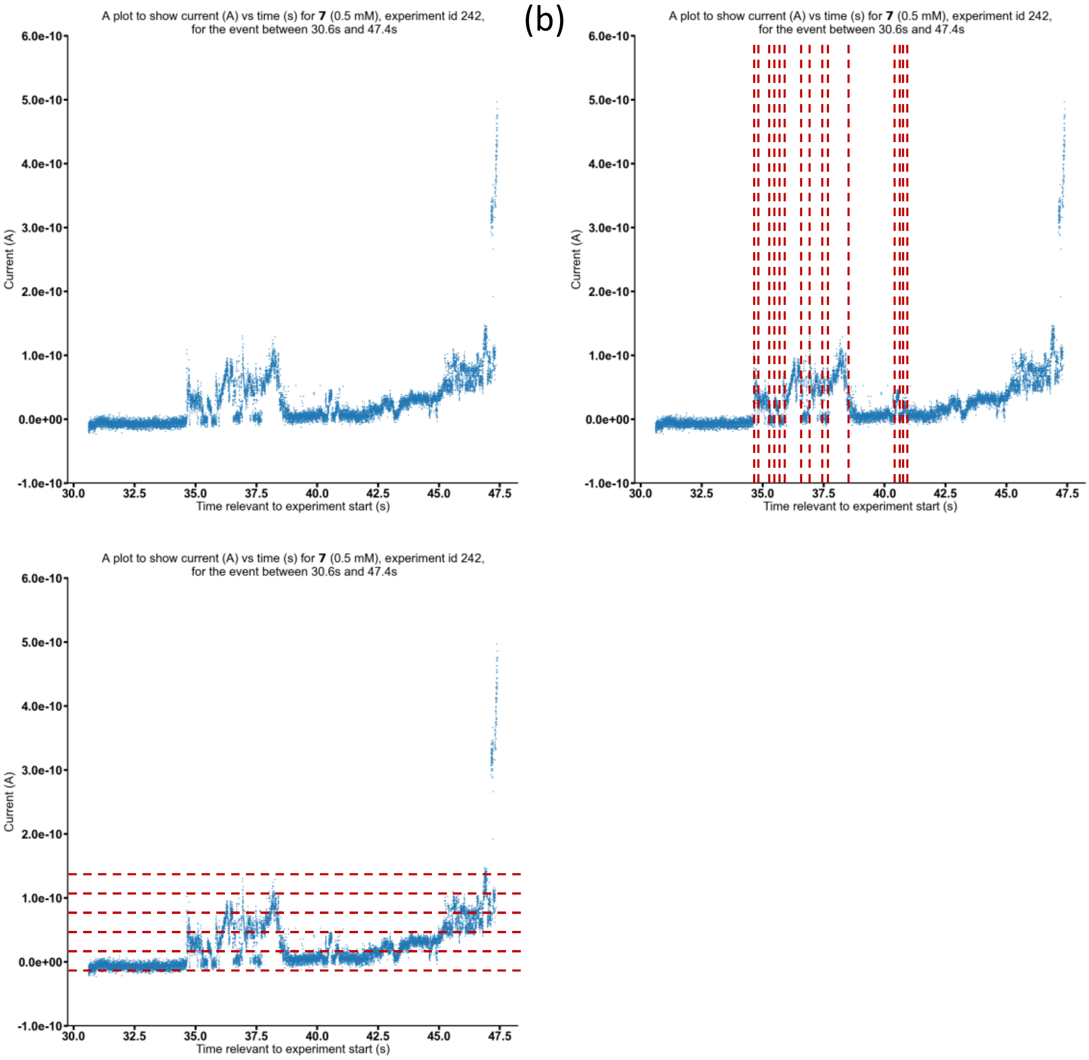

Figure S445 – Graph plots of current (A) vs time (s) for **7** (0.5 mM): (a) see graph title; (b) same as (a) but illustrates event time; (c) same as (a) but illustrates event magnitude.

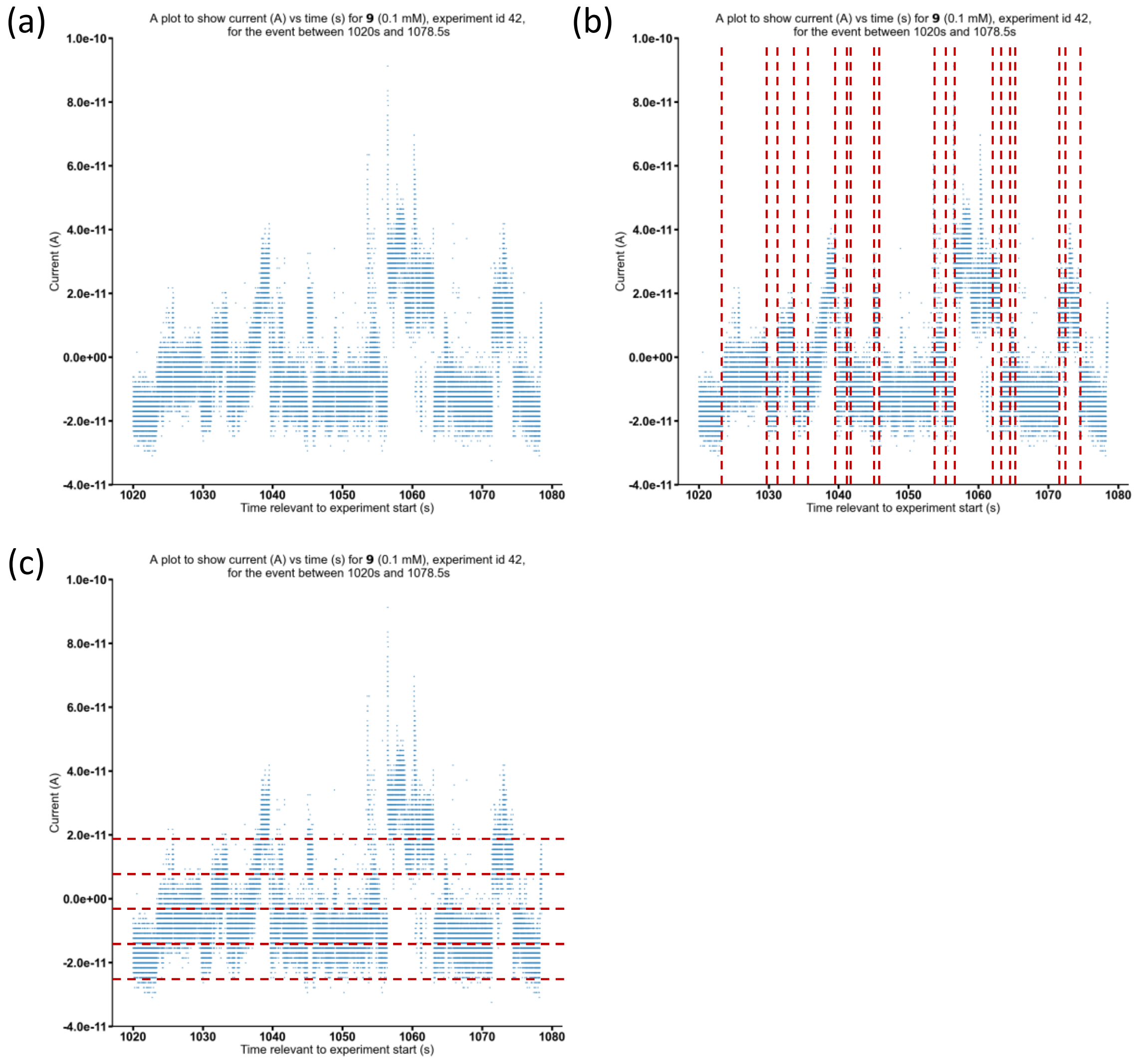

Figure S446 – Graph plots of current (A) vs time (s) for **9** (0.1 mM): (a) see graph title; (b) same as (a) but illustrates event time; (c) same as (a) but illustrates event magnitude.

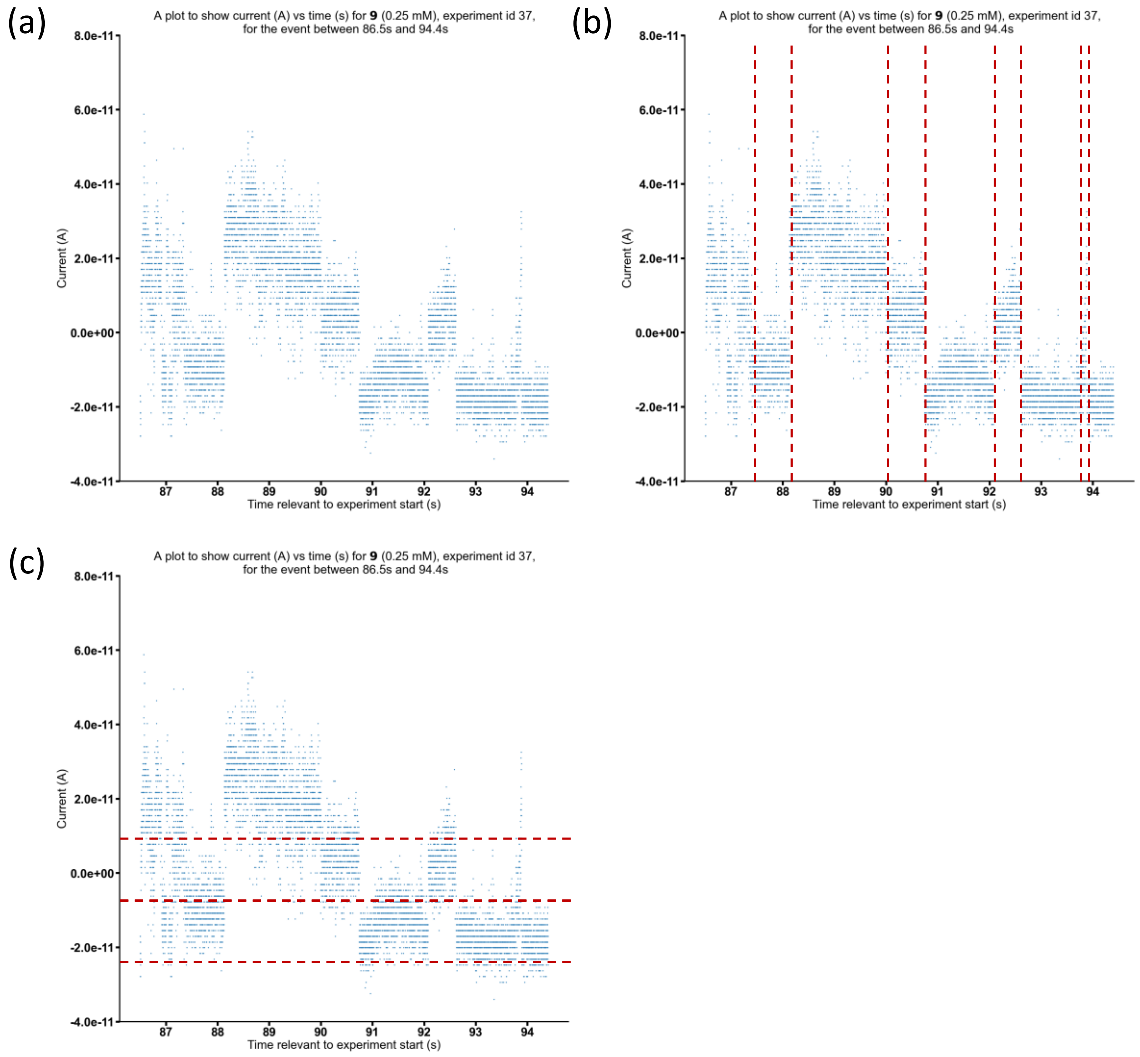

Figure S447 – Graph plots of current (A) vs time (s) for **9** (0.25 mM): (a) see graph title; (b) same as (a) but illustrates event time; (c) same as (a) but illustrates event magnitude.

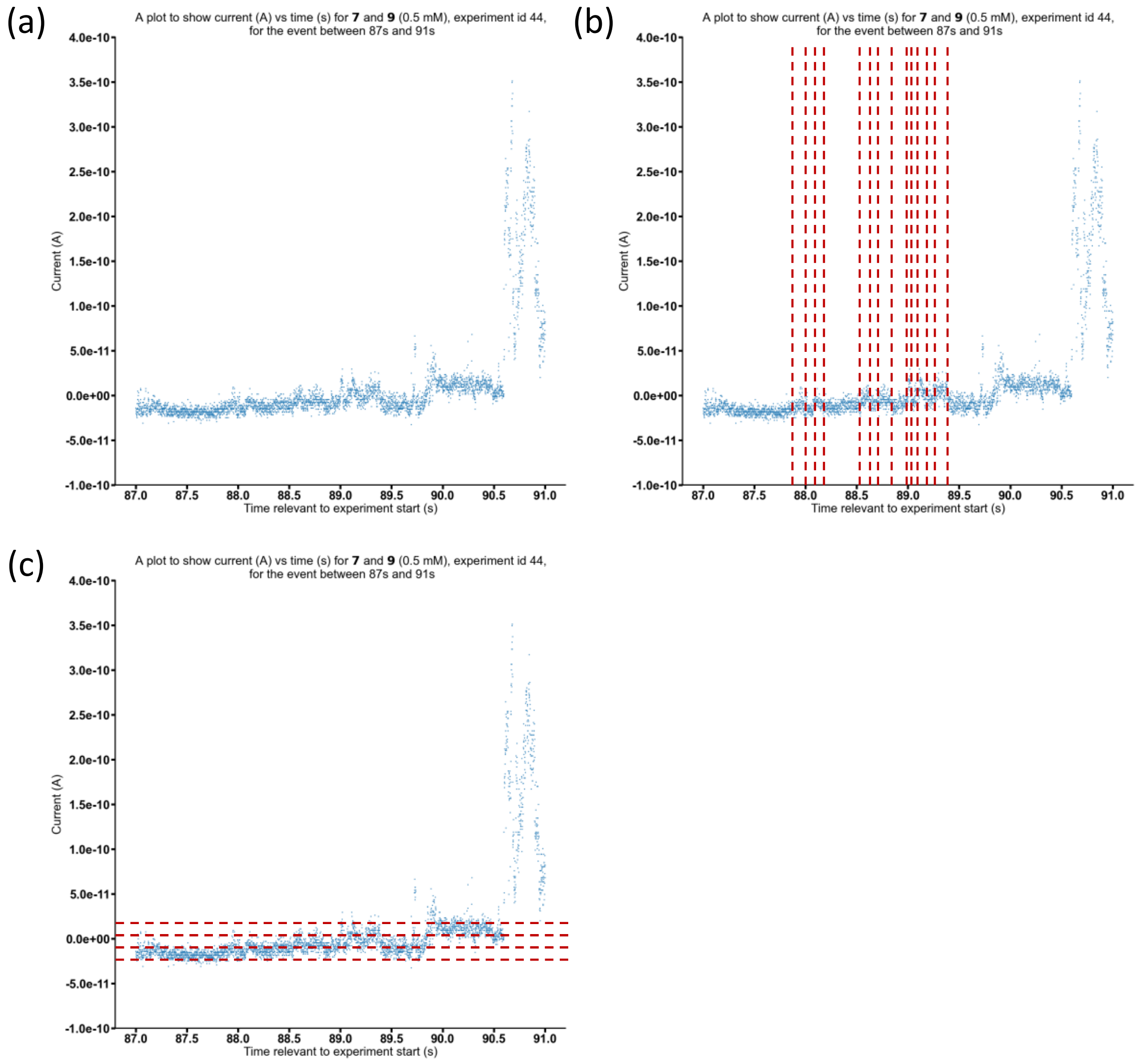

Figure S448 – Graph plots of current (A) vs time (s) for **7** and **9** (0.5 mM): (a) see graph title; (b) same as (a) but illustrates event time; (c) same as (a) but illustrates event magnitude.

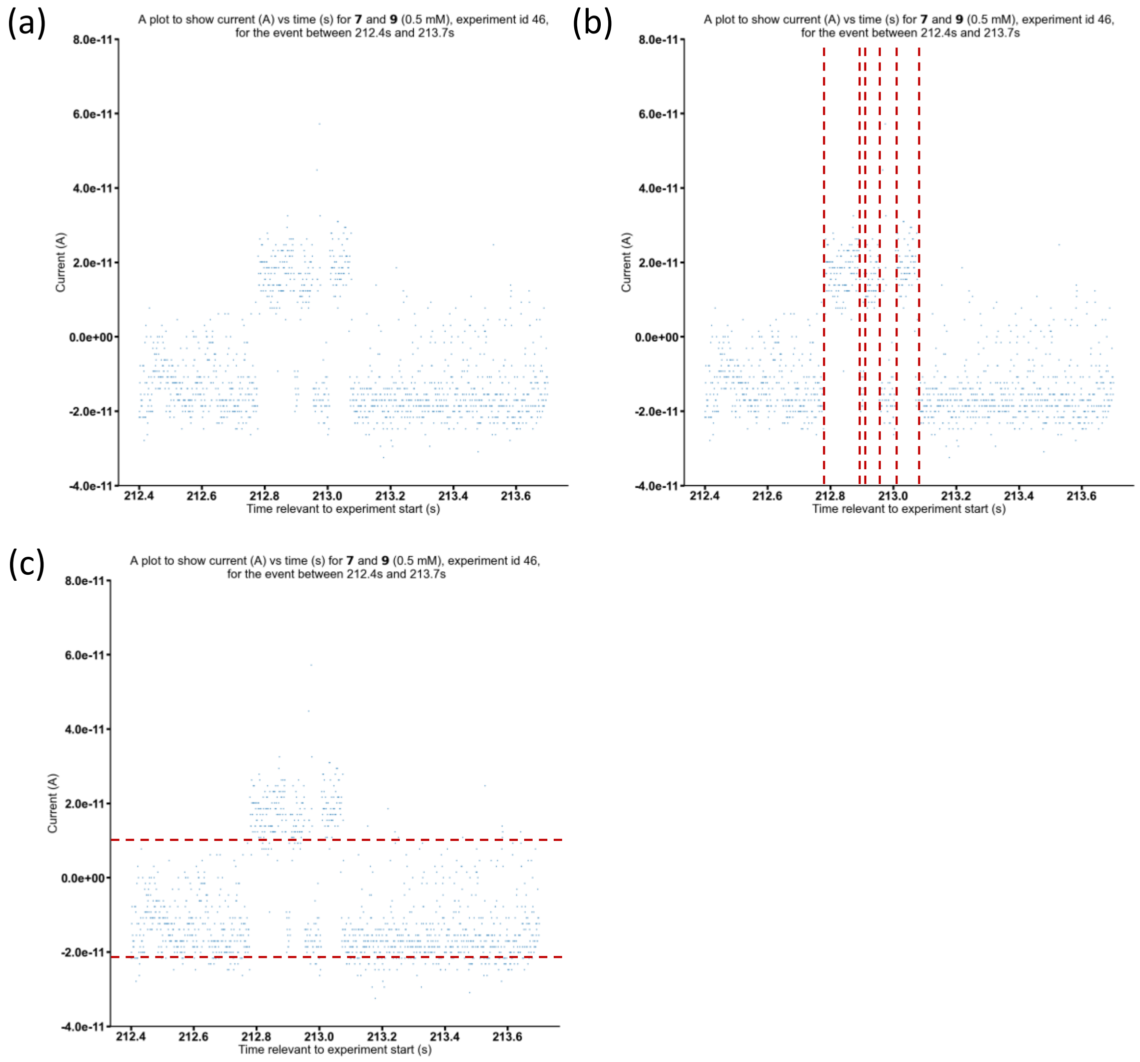

Figure S449 – Graph plots of current (A) vs time (s) for **7** and **9** (0.5 mM): (a) see graph title; (b) same as (a) but illustrates event time; (c) same as (a) but illustrates event magnitude.

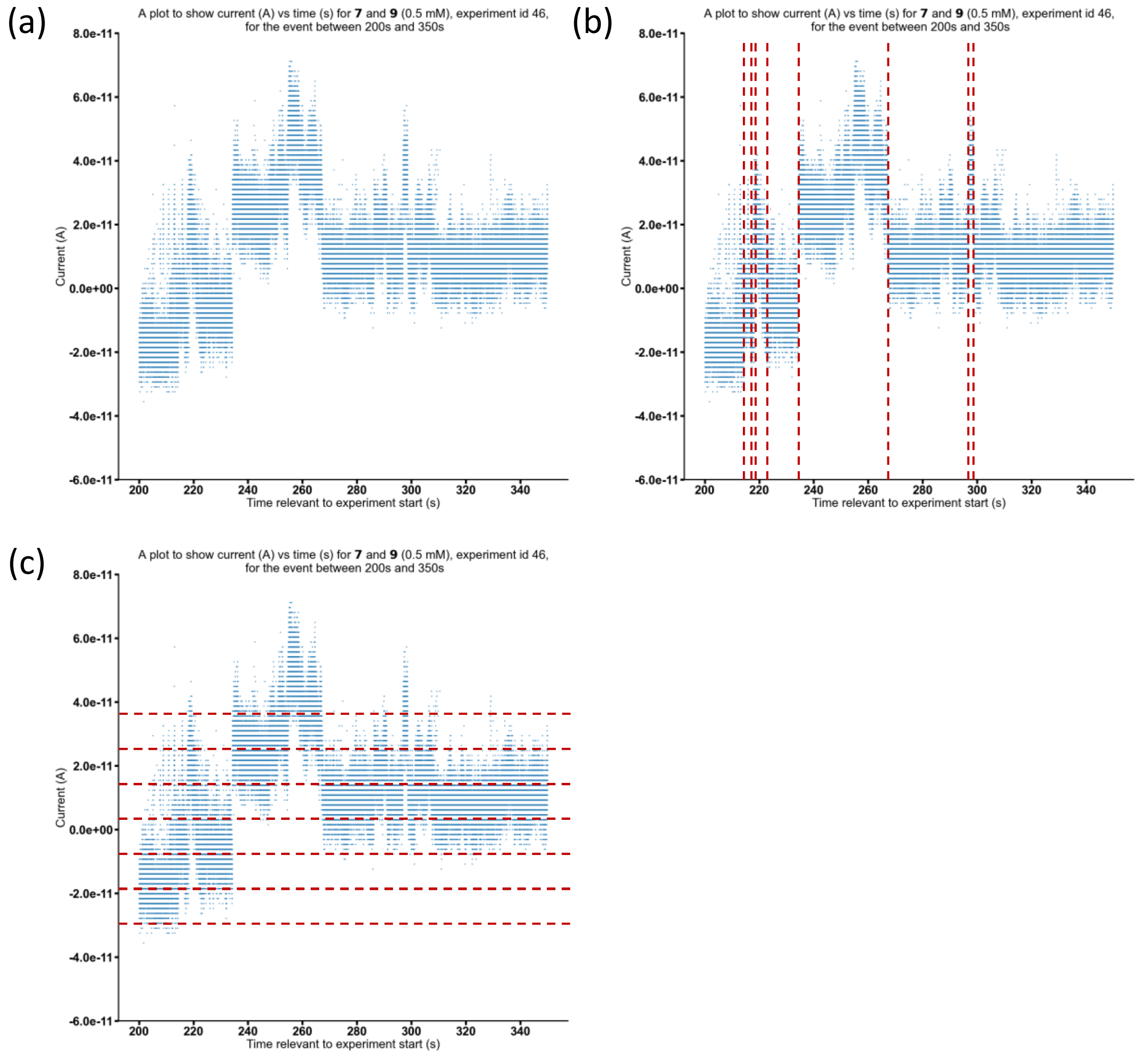

Figure S450 – Graph plots of current (A) vs time (s) for **7** and **9** (0.5 mM): (a) see graph title; (b) same as (a) but illustrates event time; (c) same as (a) but illustrates event magnitude.

Section S23: Molecular modelling and simulation

Table S20 - Summary of molecular properties generated using Chem3D (version 22.2.0) for **2**, **4**, **6**, **8** and the anionic component of **1**, **3**, **5**, **7** and **9**.

Compound	Length (Å)	Length (nm)	Dipole-dipole energy (kcal/mol)	Total energy (kcal/mol)
**1**	10.382	1.0382	-11.0797	-8.8246
**2** or **4**	13.868	1.3868	-11.3471	-8.6799
**3** or **5**	12.497	1.2497	-11.1608	-2.3045
**6** or **8**	14.067	1.4067	-10.7879	1.1185
**7** or **9**	15.434	1.5434	-10.8106	-5.6314

Section S24: Low level *in silico* modelling

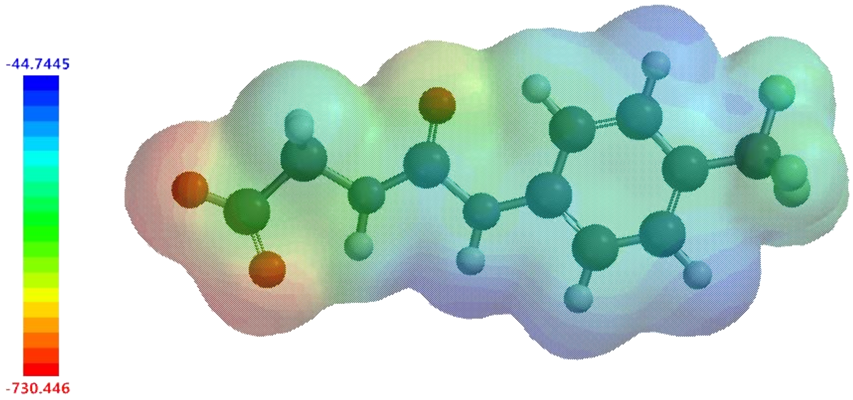

Figure S451 - Electrostatic potential map calculated for the anionic component of **1**. E_{max} and E_{min} values depicted in the figure legends are given in KJ mol^{-1}.

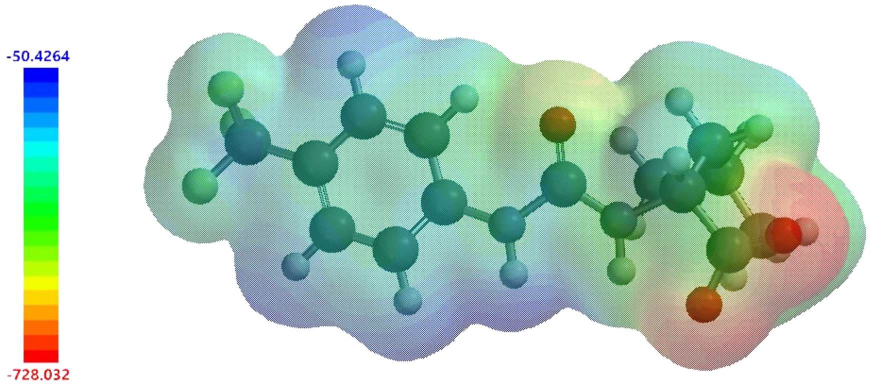

Figure S452 - Electrostatic potential map calculated for anionic component of **3**. E_{max} and E_{min} values depicted in the Figure legends are given in KJ mol^{-1}.

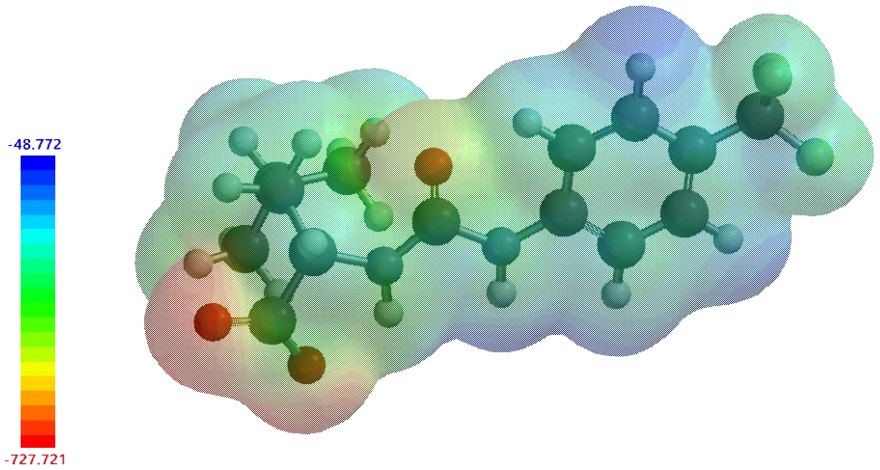

Figure S453 - Electrostatic potential map calculated for the anionic component of **5**. E_{max} and E_{min} values depicted in the Figure legends are given in KJ mol^{-1}.

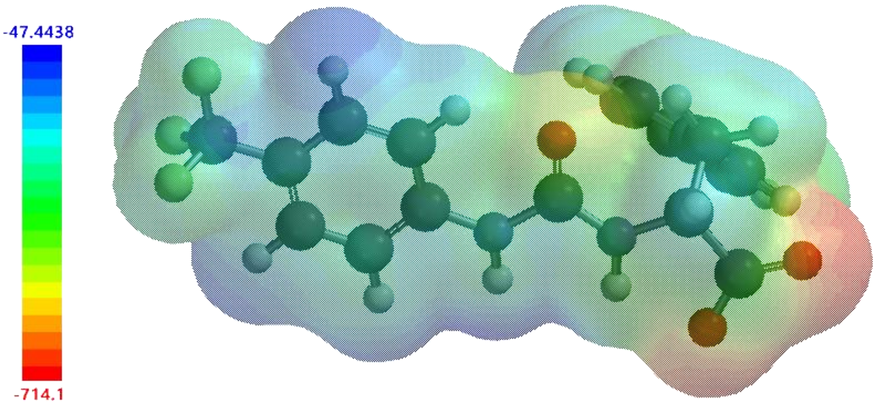

Figure S454 - Electrostatic potential map calculated for the anionic component of **7**. E_{max} and E_{min} values depicted in the figure legends are given in KJ mol^{-1}.

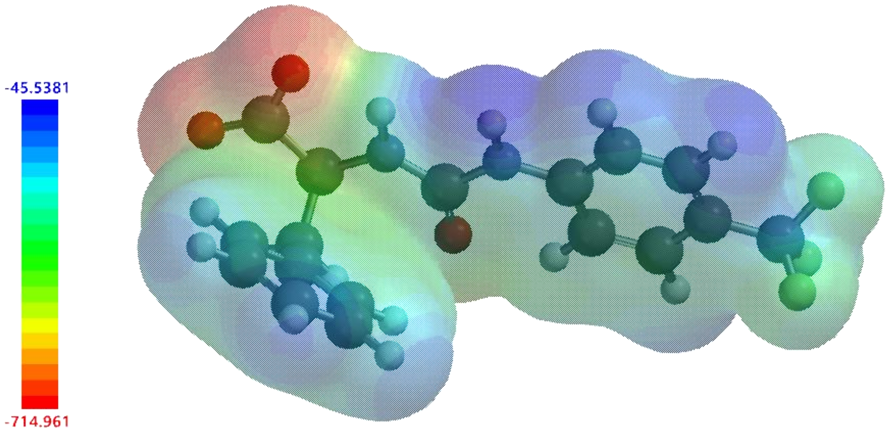

Figure S455 - Electrostatic potential map calculated for the anionic component of **9**. E_{max} and E_{min} values depicted in the figure legends are given in KJ mol^{-1}.

Summary

Table S21 – Summary of maximum E_{max} and minimum E_{min} values calculated for the anionic components of **1**, **3**, **5**, **7** and **9**.

Compound	E_{max} (KJ mol^{-1})	E_{min} (KJ mol^{-1})
**1**	-730.446	-44.744
**3** or **5**	-727.721	-48.772
**7** or **9**	-714.961	-45.538

Calculation of LogP values using swissADME

Table S22 – Summary of LogP values calculated for **1**, **3**, **5**, **7** and **9** using SwissADME.[6]

Compound	iLOGP	XLOGP3	WLOGP	MLOGP	Silicos-IT Log P	Consensus Log P
**1**	-2.07	6.69	6.54	1.03	2.29	2.9
**3**	-0.04	8.41	7.96	1.8	2.29	4.08
**5**	-0.09	8.41	7.96	1.8	2.29	4.07
**7**	-1.06	8.68	8.15	2.12	2.29	4.03
**9**	-0.13	8.68	8.15	2.12	2.29	4.22

Table S23 – Summary of LogP values calculated for the anionic component of **1**, **3**, **5**, **7** and **9** using SwissADME.[6]

Compound	iLOGP	XLOGP3	WLOGP	MLOGP	Silicos-IT Log P	Consensus Log P
**1**	1.27	1.41	1.54	1.83	1.03	1.42
**3**	2.63	3.13	2.95	2.88	2.24	2.77
**5**	2.24	3.13	2.95	2.88	2.24	2.69
**7**	2.16	3.2	3.15	3.36	2.78	2.93
**9**	2.26	3.2	3.15	3.36	2.78	2.95

Section S25: DFT SSA-Model Lipid Interaction Calculations

As the headgroup of a phospholipid is expected to be the main portion interacting with the SSAs, modelling of phospholipids was performed on truncated structures as used previously.[7] However, as steric interactions were expected to be more significant in influencing the discrepancies between entantiomers, larger model lipids were used in this study. The structures of the simplified model lipid headgroups is shown in Figure S456.

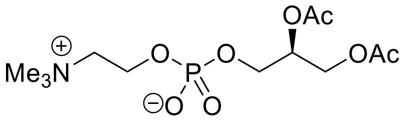

PC-Model

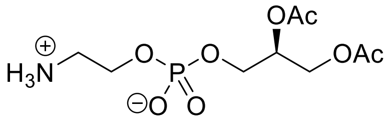

PE-Model

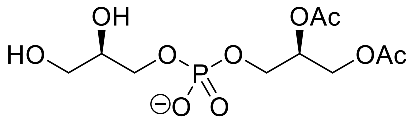

PG-Model

Figure S456 – Model phospholipid headgroups

The overall model we have selected simplifies the problem of SSA-bilayer interactions to the interactions between a single SSA and a single lipid headgroup. The simplified model operates at the assumption of high (infinite) dilution, and whilst it cannot provide detailed energetic information about interactions of an isolated SSA with a lipid bilayer, nor between a larger SSA aggregate with a lipid bilayer, the energies of diastereomeric interactions between SSA and lipid do allow insight into the extent to which lipid/SSA chirality may be of significance.

A conformation search for **7** about the 2 rotatable bonds of the urea moiety, and the C-N and C-C bonds in the backbone of the phenylalanine moiety was performed at the PM6 level using a COSMO(water) solvent model in Gaussian16;[8] the C-aryl and C-CO2 bonds were allowed to freely relax in each case. The geometry was then reoptimized at the M06-2X/6-311+g(d,p) level with a PCM(water) solvation model. The M06-2X functional was chosen it includes a dispersion consideration and has been shown to be effective at modelling hydrogen bonding to anions.[9] The linear, “open” form (as required for interaction with a phospholipid head group) was similarly optimised. The lowest energy isomer in the absence of an explicit cation was found to be, as expected, the one with intramolecular hydrogen bonding between the urea-moiety and the carboxylate group. The energy cost, ΔH, for unfolding to the linear form was $32.3\ \mathrm{kJ.mol^{-1}}$ ($\Delta G = 28.2\ \mathrm{kJ.mol^{-1}}$), indicating that even in the absence of an additional interaction at infinite dilution, the linear form is thermally accessible at ambient temperature and the SSA will be available for binding. The geometries were then inverted for subsequent calculations involving **9**. The geometry of the model phospholipid were optimised in the broadly linear geometry required for SSA/Lipid interactions at the M06-2X/ 6-311+g(d,p) level with a PCM(water) solvation model. All structures were confirmed as energy minima by the absence of any imaginary frequencies.

A range of SSA-headgroup interaction conformations were then optimised using the same computational parameters. The binding energy for each SSA-headgroup conformation was then calculated using Equation S1 – the energies were obtained from the output files and converted from Hartree to $kJ.mol^{-1}$. All calculated binding energies are shown in Table S24. Cartesian coordinates for the minimised binding conformations are given in Tables S25-35 and 3D visualisations of the structures are shown in Figures S457-467.

SSA-headgroup binding energy = SSA-headgroup energy – (SSA energy + headgroup energy)

Equation S1

Table S24 – Energies of interaction between **7** and **9**, and Phospholipid Models

	Binding Energy / $\mathbf{kJ.mol^{-1}}$	
**Interaction**	$\Delta\boldsymbol{H}$	$\Delta\boldsymbol{G}$
**7 / PC-Model**	-95.2	-30.7
**9 / PC-Model**	-110.8	-31.4
**7 / PE-Model**	-160.8	-83.1
**9 / PE-Model**	-133.2	-70.2
**7 / PG-Model**	-79.5	-17.0
**9 / PG-Model**	-102.1	-37.9

These results indicate that binding is exothermic in all cases, even for the SSA/PG-model interactions which are formally anion/anion events; all binding events are also exergonic, albeit to a lesser extent as expected for the decrease in entropy when two molecules come together. In each case, the binding geometry involves the two P-O groups oriented towards the urea N-H whilst the carboxylate group is closely associated with the cationic (PC, PE) or neutral (PG) head group. The significant increase in exergonicity between binding PC and PE can be attributed to not only the closer approach of carboxylate to ammonium centre permitted by the reduction in steric hindrance as methyl groups are replaced by protons but also the formation of O-HN hydrogen bonds. The differences in energy between diastereomeric model complexes range from $0.7\ \mathrm{kJ.mol^{-1}}$ to $20.9\ \mathrm{kJ.mol^{-1}}$, and show significant differences between phospholipid headgroups, indicating both that SSA chirality may induce significant changes in behaviour and that this may be used to enhance selectivity for different cell membrane compositions.

7 Favoured Geometry

Figure S457 – Optimised Geometry for lowest energy conformation of **7**

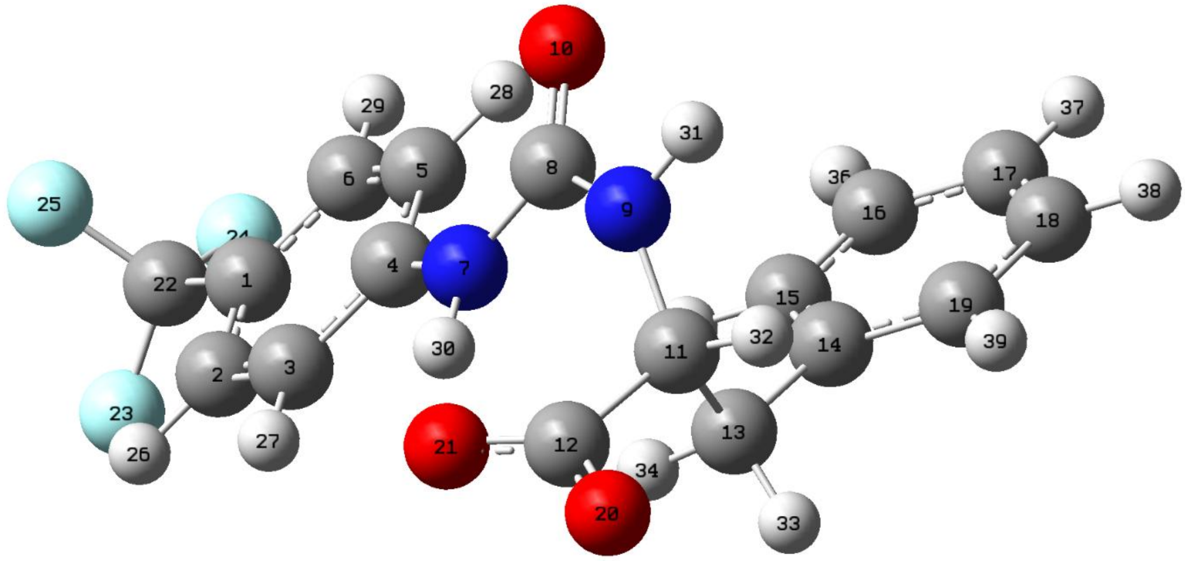

Table S25 – Cartesian Coordinates for **7** in its lowest energy conformation

Center Number	Atomic Number	Atomic Type	Coordinates (Angstroms) X	Y	Z
1	6	0	-5.502198	3.561507	-2.368342
2	6	0	-5.053466	4.286970	-1.266234
3	6	0	-3.954192	3.844744	-0.552780
4	6	0	-3.279036	2.671649	-0.928621
5	6	0	-3.739352	1.945235	-2.037368
6	6	0	-4.842876	2.396542	-2.747604
7	7	0	-2.216574	2.271164	-0.129987
8	6	0	-1.295487	1.291761	-0.413297
9	7	0	-0.509924	0.945605	0.657921
10	8	0	-1.133141	0.790907	-1.521839
11	6	0	-0.885746	0.961850	2.079756
12	6	0	-0.730838	2.345861	2.760751
13	6	0	-2.315221	0.439653	2.364373
14	6	0	-2.627636	-0.831566	1.617301
15	6	0	-3.522966	-0.826202	0.546754
16	6	0	-3.777819	-1.989686	-0.176339
17	6	0	-3.137534	-3.176895	0.164891
18	6	0	-2.244575	-3.194568	1.235345
19	6	0	-1.992627	-2.030416	1.953612
20	8	0	-0.182561	2.358427	3.875379
21	8	0	-1.223604	3.333851	2.149525
22	6	0	-6.651881	4.066720	-3.175053
23	9	0	-7.531097	4.764441	-2.435425
24	9	0	-7.338552	3.076087	-3.769627
25	9	0	-6.261391	4.896314	-4.168267
26	1	0	-5.564856	5.192448	-0.962528
27	1	0	-3.605882	4.401717	0.309706
28	1	0	-3.234921	1.038108	-2.333563
29	1	0	-5.195332	1.827404	-3.599682
30	1	0	-2.007080	2.811167	0.732183
31	1	0	0.138152	0.213460	0.404983
32	1	0	-0.177600	0.288268	2.561081

33	1	0	-2.380244	0.275530	3.443752
34	1	0	-3.049224	1.205706	2.107590
35	1	0	-4.023914	0.098561	0.275344
36	1	0	-4.475921	-1.966479	-1.005454
37	1	0	-3.333630	-4.083302	-0.395618
38	1	0	-1.746423	-4.116921	1.510955
39	1	0	-1.297302	-2.050926	2.787476

E(RM062X) = -1291.064783 Hartree

Sum of electronic and thermal Free Energies = -1290.828543 Hartree

7 Linear Geometry

Figure S458 – Optimised Geometry for linear conformation of **7**

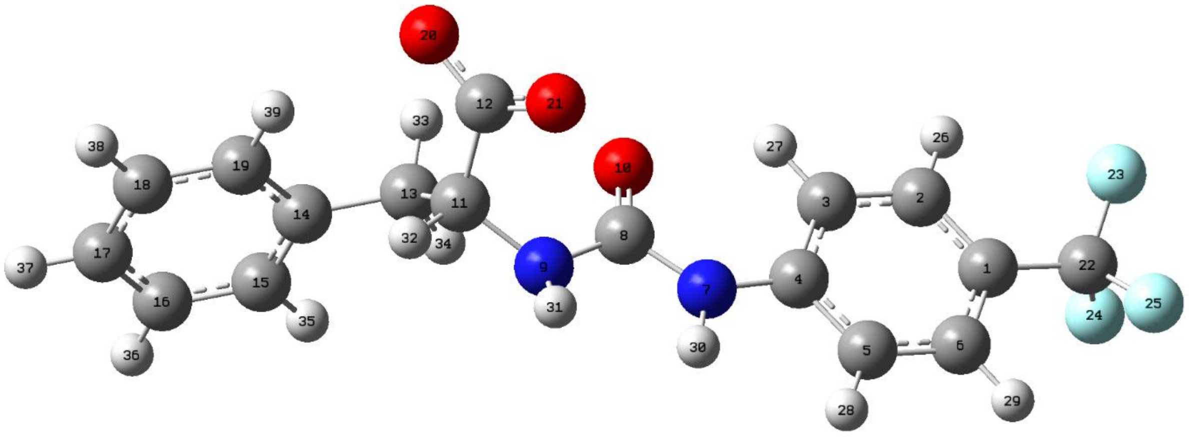

Table S26 – Cartesian Coordinates for **7** in its linear conformation, prior to binding

Center Number	Atomic Number	Atomic Type	Coordinates (Angstroms) X	Y	Z
1	6	0	-3.410901	5.632821	1.944699
2	6	0	-2.735162	4.470696	2.297227
3	6	0	-3.237126	3.223169	1.950355
4	6	0	-4.443815	3.133581	1.243141
5	6	0	-5.119197	4.313856	0.892053
6	6	0	-4.609850	5.551048	1.239401
7	7	0	-5.021271	1.931167	0.835316
8	6	0	-4.635552	0.641044	1.169819
9	7	0	-5.454222	-0.325913	0.659363
10	8	0	-3.680430	0.391697	1.888827
11	6	0	-4.974229	-1.705337	0.656840
12	6	0	-3.636343	-1.857910	-0.138812
13	6	0	-4.936150	-2.305358	2.080178
14	6	0	-5.665133	-3.623086	2.211059
15	6	0	-6.650072	-3.799101	3.185183
16	6	0	-7.323750	-5.012628	3.314642
17	6	0	-7.021717	-6.072227	2.465398
18	6	0	-6.041420	-5.908362	1.487736
19	6	0	-5.369538	-4.696430	1.362623
20	8	0	-2.949281	-2.866414	0.132832
21	8	0	-3.406908	-0.994474	-1.012117
22	6	0	-2.890372	6.965331	2.372336
23	9	0	-1.555866	6.972546	2.523891
24	9	0	-3.403635	7.361881	3.557684
25	9	0	-3.191820	7.938479	1.495217
26	1	0	-1.800243	4.531234	2.841293
27	1	0	-2.702472	2.327530	2.221152
28	1	0	-6.050029	4.253014	0.339768
29	1	0	-5.142929	6.450955	0.957416
30	1	0	-5.870872	2.024958	0.296749
31	1	0	-6.011026	-0.065600	-0.141618
32	1	0	-5.713094	-2.260784	0.071612
33	1	0	-3.893131	-2.439380	2.371392
34	1	0	-5.378471	-1.587506	2.772203
35	1	0	-6.893850	-2.976324	3.849716
36	1	0	-8.085766	-5.126912	4.077220

37	1	0	-7.544645	-7.016484	2.562175
38	1	0	-5.798754	-6.729462	0.822467
39	1	0	-4.598633	-4.566141	0.609914

E(RM062X) = -1291.052492 Hartree

Sum of electronic and thermal Free Energies = -1290.817817 Hartree

PC-Model

Figure S459 – Optimised Geometry for linear conformation of **PC-Model**

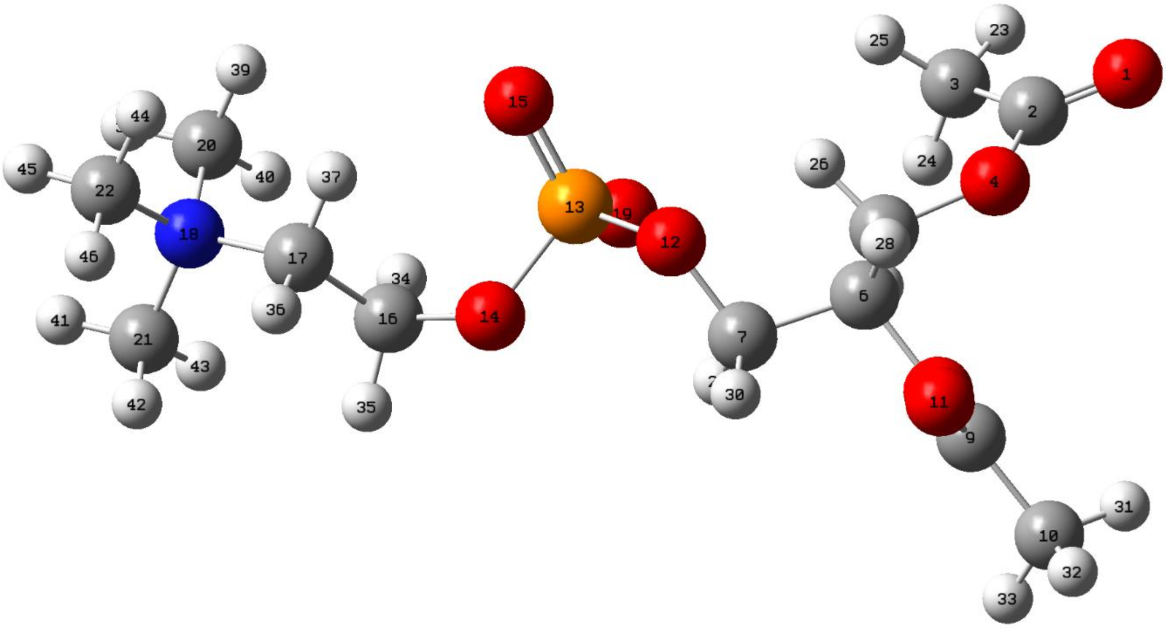

Table S27 – Cartesian Coordinates for **PC-Model** prior to binding

Center Number	Atomic Number	Atomic Type	Coordinates (Angstroms) X	Y	Z
1	8	0	-4.471236	2.317562	4.459282
2	6	0	-3.516342	1.715084	4.038081
3	6	0	-2.205052	1.606944	4.766649
4	8	0	-3.627315	1.108549	2.842998
5	6	0	-2.531759	0.363765	2.298256
6	6	0	-2.941855	-0.084467	0.917176
7	6	0	-1.779256	-0.718141	0.160631
8	8	0	-3.972899	-1.067272	1.087526
9	6	0	-4.854430	-1.228067	0.083508
10	6	0	-5.880016	-2.266522	0.425005
11	8	0	-4.800891	-0.599911	-0.943366
12	8	0	-0.844046	0.299184	-0.176848
13	15	0	0.660406	0.261186	0.441918
14	8	0	1.202561	-1.052356	-0.404980
15	8	0	1.376082	1.485529	-0.029531
16	6	0	2.552432	-1.434844	-0.203631
17	6	0	3.392944	-0.681658	-1.235643
18	7	0	4.882873	-0.773864	-1.026628
19	8	0	0.616519	-0.096853	1.895698
20	6	0	5.294963	-0.013554	0.195734
21	6	0	5.316338	-2.200089	-0.902897
22	6	0	5.552409	-0.161406	-2.217957
23	1	0	-2.299833	2.111441	5.723717
24	1	0	-1.934099	0.561960	4.924288
25	1	0	-1.408305	2.073901	4.184385
26	1	0	-1.635859	0.980175	2.225931
27	1	0	-2.319853	-0.507199	2.923666
28	1	0	-3.333678	0.759857	0.347196
29	1	0	-1.315601	-1.496767	0.772959
30	1	0	-2.151115	-1.162960	-0.763286

31	1	0	-6.429826	-1.952261	1.313179
32	1	0	-6.561510	-2.396147	-0.411102
33	1	0	-5.381760	-3.208655	0.655930
34	1	0	2.862616	-1.214516	0.822518
35	1	0	2.608426	-2.512377	-0.355944
36	1	0	3.189399	-1.076113	-2.231612
37	1	0	3.130707	0.376515	-1.205421
38	1	0	6.379356	-0.055967	0.274109
39	1	0	4.962656	1.016901	0.088886
40	1	0	4.846154	-0.467604	1.074644
41	1	0	6.403483	-2.226454	-0.871527
42	1	0	4.950513	-2.750556	-1.767807
43	1	0	4.911502	-2.618290	0.015833
44	1	0	5.200821	0.862968	-2.322509
45	1	0	6.628295	-0.179126	-2.057628
46	1	0	5.291481	-0.743623	-3.098987

E(RM062X) = -1469.620969 Hartree

Sum of electronic and thermal Free Energies = -1469.295737 Hartree

PE-Model

Figure S460 – Optimised Geometry for linear conformation of **PE-Model**

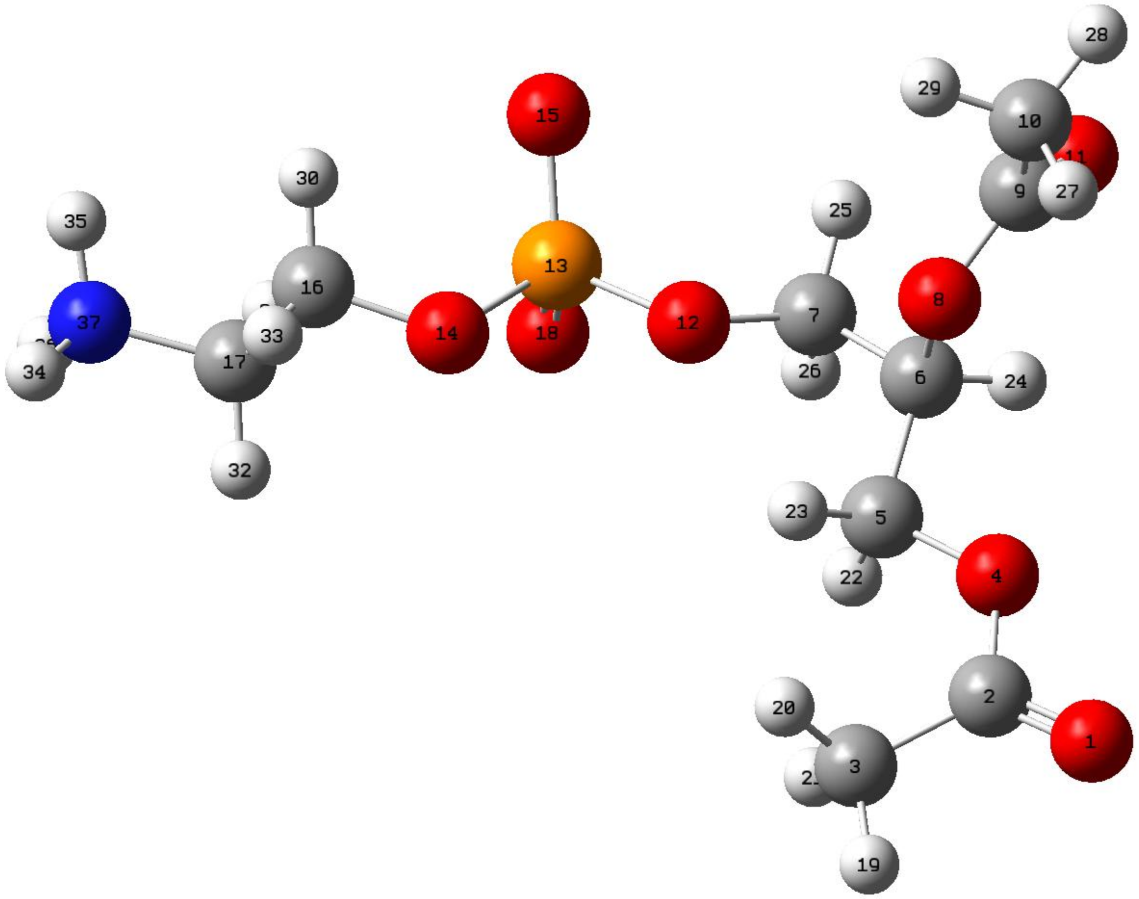

Table S28 – Cartesian Coordinates for **PE-Model** prior to binding

Center Number	Atomic Number	Atomic Type	Coordinates (Angstroms) X	Y	Z
1	8	0	-6.212381	-0.384589	0.400629
2	6	0	-5.221945	0.292684	0.508206
3	6	0	-5.242049	1.784424	0.695253
4	8	0	-4.027274	-0.324777	0.466402
5	6	0	-2.809408	0.420870	0.559561
6	6	0	-1.683762	-0.585210	0.631085
7	6	0	-0.331287	0.083059	0.487139
8	8	0	-1.802279	-1.233667	1.906371
9	6	0	-1.272048	-2.461886	2.029614
10	6	0	-1.485099	-3.009320	3.408831
11	8	0	-0.703017	-3.024918	1.127887
12	8	0	-0.250318	1.137899	1.437010
13	15	0	1.112676	2.039899	1.427336
14	8	0	0.487109	3.221472	2.385711
15	8	0	2.216404	1.346920	2.165570
16	6	0	1.409725	4.142473	2.931670
17	6	0	0.589324	5.087889	3.790161
18	8	0	1.356883	2.566745	0.046622
19	1	0	-6.270502	2.126270	0.625170
20	1	0	-4.838450	2.044112	1.675998
21	1	0	-4.633248	2.282804	-0.060177

22	1	0	-2.680280	1.043590	-0.328904
23	1	0	-2.803622	1.046563	1.452203
24	1	0	-1.796917	-1.329193	-0.159771
25	1	0	0.460563	-0.652262	0.656995
26	1	0	-0.233548	0.479453	-0.528325
27	1	0	-2.552977	-3.037814	3.628253
28	1	0	-1.059890	-4.006819	3.476271
29	1	0	-1.011361	-2.348694	4.136166
30	1	0	2.159243	3.620605	3.534769
31	1	0	1.920021	4.696265	2.135154
32	1	0	-0.163429	5.601698	3.196401
33	1	0	0.112559	4.557558	4.611462
34	1	0	0.940659	6.785797	4.967968
35	1	0	2.201036	5.726576	4.984589
36	1	0	1.947006	6.690702	3.668243
37	7	0	1.476736	6.136049	4.388557

E(RM062X) = -1351.72506 Hartree

Sum of electronic and thermal Free Energies = -1351.481686 Hartree

PG-Model

Figure S461 – Optimised Geometry for linear conformation of **PG-Model**

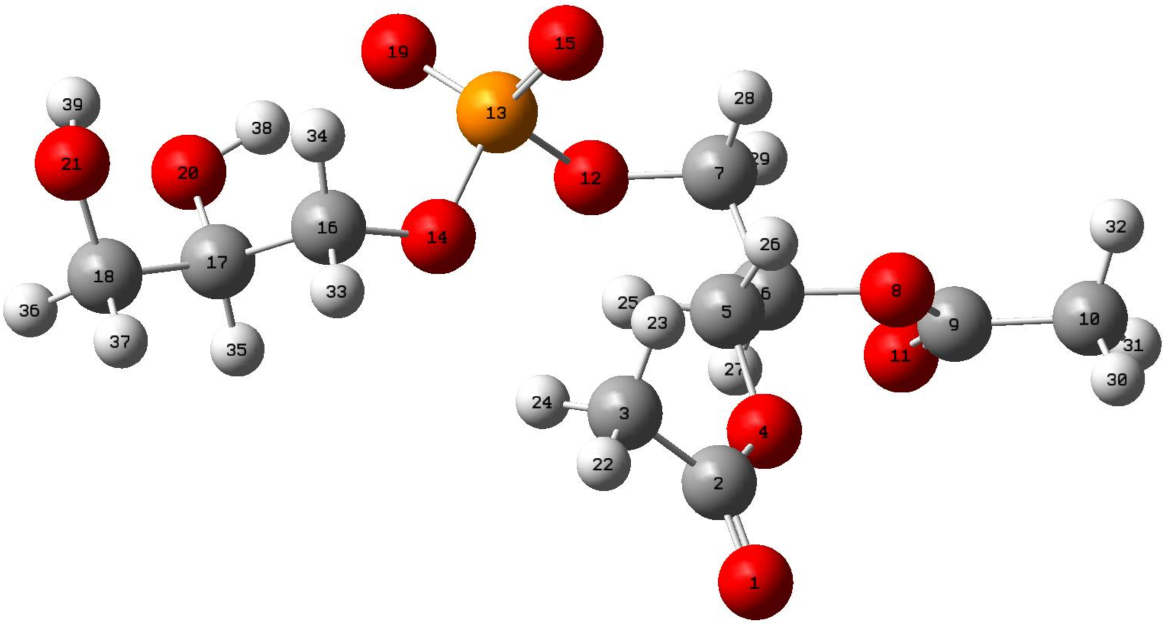

Table S29 – Cartesian Coordinates for **PG-Model** prior to binding

Center Number	Atomic Number	Atomic Type	Coordinates (Angstroms) X	Y	Z
1	8	0	-2.453885	0.472355	-4.209620
2	6	0	-2.458793	0.703012	-3.026886
3	6	0	-2.164111	2.055047	-2.436133
4	8	0	-2.729309	-0.310303	-2.185138
5	6	0	-2.794033	-0.083729	-0.772034
6	6	0	-2.754908	-1.430559	-0.094000
7	6	0	-2.669131	-1.297436	1.424403
8	8	0	-3.979996	-2.101996	-0.421249
9	6	0	-3.974464	-3.447607	-0.422604
10	6	0	-5.315947	-3.993545	-0.809110
11	8	0	-2.999924	-4.100392	-0.146688
12	8	0	-1.395472	-0.791827	1.801394
13	15	0	-1.163326	0.758793	2.241758
14	8	0	-0.276770	1.228452	0.944437
15	8	0	-2.469969	1.484549	2.272205
16	6	0	0.438828	2.468065	1.006812
17	6	0	1.906704	2.234967	1.338584
18	6	0	2.726832	3.502344	1.143615
19	8	0	-0.250424	0.776162	3.438850
20	8	0	2.104636	1.841904	2.686624
21	8	0	2.316873	4.513412	2.050588
22	1	0	-2.041952	2.765962	-3.247986
23	1	0	-2.969066	2.379045	-1.774749
24	1	0	-1.245252	2.011995	-1.847221
25	1	0	-1.938488	0.502770	-0.438419
26	1	0	-3.722441	0.437678	-0.523828
27	1	0	-1.910019	-2.013576	-0.465045
28	1	0	-3.467232	-0.646677	1.788960
29	1	0	-2.773502	-2.286354	1.871257
30	1	0	-5.573381	-3.644852	-1.810064

31	1	0	-5.289253	-5.079393	-0.785347
32	1	0	-6.073284	-3.619428	-0.119289
33	1	0	0.362191	2.925679	0.018841
34	1	0	-0.016395	3.144648	1.737053
35	1	0	2.285835	1.463414	0.655204
36	1	0	3.785190	3.260865	1.290821
37	1	0	2.598757	3.896345	0.134222
38	1	0	1.304886	1.380493	3.021192
39	1	0	2.282018	4.090828	2.917808

E(RM062X) = -1485.690506 Hartree

Sum of electronic and thermal Free Energies = -1485.441489 Hartree

7-PC-Model Binding

Figure S462 – Optimised geometry interaction between **7** and **PC-Model**

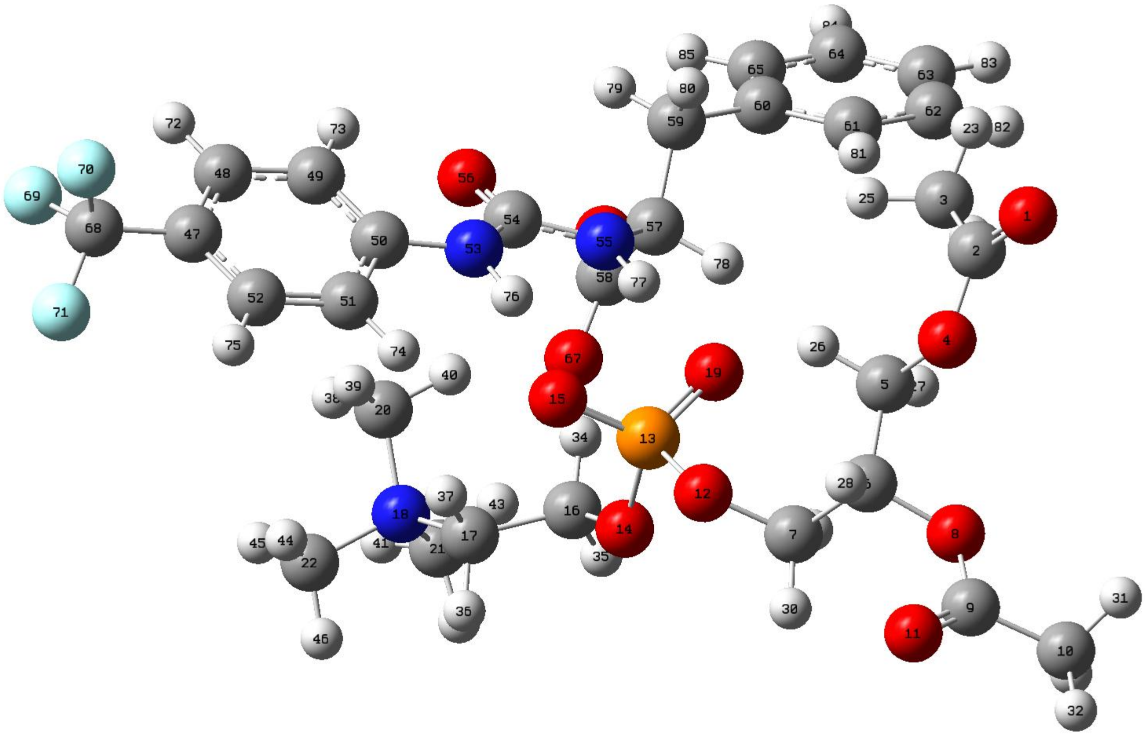

Table S30 – Cartesian Coordinates for the **7**/**PC-Model** binding interaction

Center Number	Atomic Number	Atomic Type	Coordinates (Angstroms) X	Y	Z
1	8	0	-4.519258	4.015743	1.900267
2	6	0	-3.723990	3.743243	1.036883
3	6	0	-2.875282	4.762517	0.327055
4	8	0	-3.581739	2.450958	0.692397
5	6	0	-2.663178	2.070369	-0.338025
6	6	0	-2.722034	0.566672	-0.451812
7	6	0	-1.904840	0.053487	-1.632680
8	8	0	-2.157204	0.036250	0.754828
9	6	0	-2.559299	-1.187140	1.147572
10	6	0	-1.901478	-1.585443	2.434023
11	8	0	-3.348581	-1.849262	0.522989
12	8	0	-2.555195	0.425263	-2.846269
13	15	0	-1.835747	1.459935	-3.859187
14	8	0	-0.597155	0.509611	-4.346678
15	8	0	-2.764237	1.713554	-5.011634
16	6	0	0.297293	1.059484	-5.313161
17	6	0	-0.140965	0.508836	-6.667968
18	7	0	0.623908	1.055155	-7.845858
19	8	0	-1.283345	2.641391	-3.111589
20	6	0	0.205628	2.463587	-8.139139
21	6	0	2.097535	1.032889	-7.580947
22	6	0	0.316381	0.200023	-9.032844
23	1	0	-3.147317	5.749537	0.689426
24	1	0	-1.817915	4.578611	0.527241
25	1	0	-3.024125	4.710715	-0.752623
26	1	0	-2.944558	2.517667	-1.291179
27	1	0	-1.648545	2.383063	-0.080200

28	1	0	-3.757292	0.234365	-0.547212
29	1	0	-0.888911	0.455871	-1.587990
30	1	0	-1.859547	-1.035148	-1.593908
31	1	0	-2.155531	-0.862008	3.209733
32	1	0	-2.234641	-2.578423	2.722650
33	1	0	-0.818358	-1.573126	2.306795
34	1	0	0.305124	2.154169	-5.289132
35	1	0	1.296821	0.713818	-5.050956
36	1	0	-0.008088	-0.573731	-6.678445
37	1	0	-1.195031	0.742178	-6.832075
38	1	0	0.774160	2.807736	-9.002112
39	1	0	-0.858271	2.462224	-8.369689
40	1	0	0.440614	3.084424	-7.274977
41	1	0	2.609121	1.270301	-8.511896
42	1	0	2.375431	0.034661	-7.245933
43	1	0	2.322463	1.789717	-6.829800
44	1	0	-0.763226	0.176784	-9.172386
45	1	0	0.799987	0.634363	-9.905336
46	1	0	0.697164	-0.802631	-8.849073
47	6	0	-4.223733	3.571058	-10.163283
48	6	0	-3.478072	4.727611	-9.963362
49	6	0	-2.802873	4.939051	-8.768429
50	6	0	-2.871363	3.976779	-7.750487
51	6	0	-3.637640	2.814620	-7.957839
52	6	0	-4.308691	2.616277	-9.150732
53	7	0	-2.231826	4.092606	-6.520453
54	6	0	-1.132206	4.895958	-6.233678
55	7	0	-0.669107	4.747237	-4.972401
56	8	0	-0.630766	5.665178	-7.049443
57	6	0	0.560005	5.390630	-4.535519
58	6	0	1.774086	5.018615	-5.445647
59	6	0	0.384403	6.904883	-4.320964
60	6	0	1.329917	7.478031	-3.292456
61	6	0	1.173732	7.153127	-1.941433
62	6	0	2.023775	7.679584	-0.972899
63	6	0	3.047709	8.548622	-1.342305
64	6	0	3.212359	8.882030	-2.684469
65	6	0	2.361609	8.347655	-3.648768
66	8	0	2.600844	5.916817	-5.689465
67	8	0	1.831178	3.817545	-5.810962
68	6	0	-4.986220	3.378044	-11.431449
69	9	0	-4.428228	4.019416	-12.472008
70	9	0	-6.254849	3.837572	-11.347254
71	9	0	-5.086725	2.082382	-11.777871
72	1	0	-3.416254	5.474213	-10.746247
73	1	0	-2.221510	5.835452	-8.624148
74	1	0	-3.683500	2.072821	-7.167715
75	1	0	-4.888437	1.712834	-9.298816
76	1	0	-2.437873	3.329114	-5.868348
77	1	0	-1.055473	4.035478	-4.352623
78	1	0	0.780198	4.931828	-3.565661
79	1	0	0.505951	7.420793	-5.273001
80	1	0	-0.641509	7.067282	-3.977324
81	1	0	0.370553	6.484482	-1.643941
82	1	0	1.883464	7.416499	0.069394
83	1	0	3.709706	8.963101	-0.590987
84	1	0	4.006945	9.557197	-2.981567
85	1	0	2.502180	8.591335	-4.695520

E(RM062X) = -2760.70971 Hartree
Sum of electronic and thermal Free Energies = -2760.125264 Hartree

9-PC-Model Binding

Figure S463 – Optimised geometry interaction between **9** and **PC-Model**

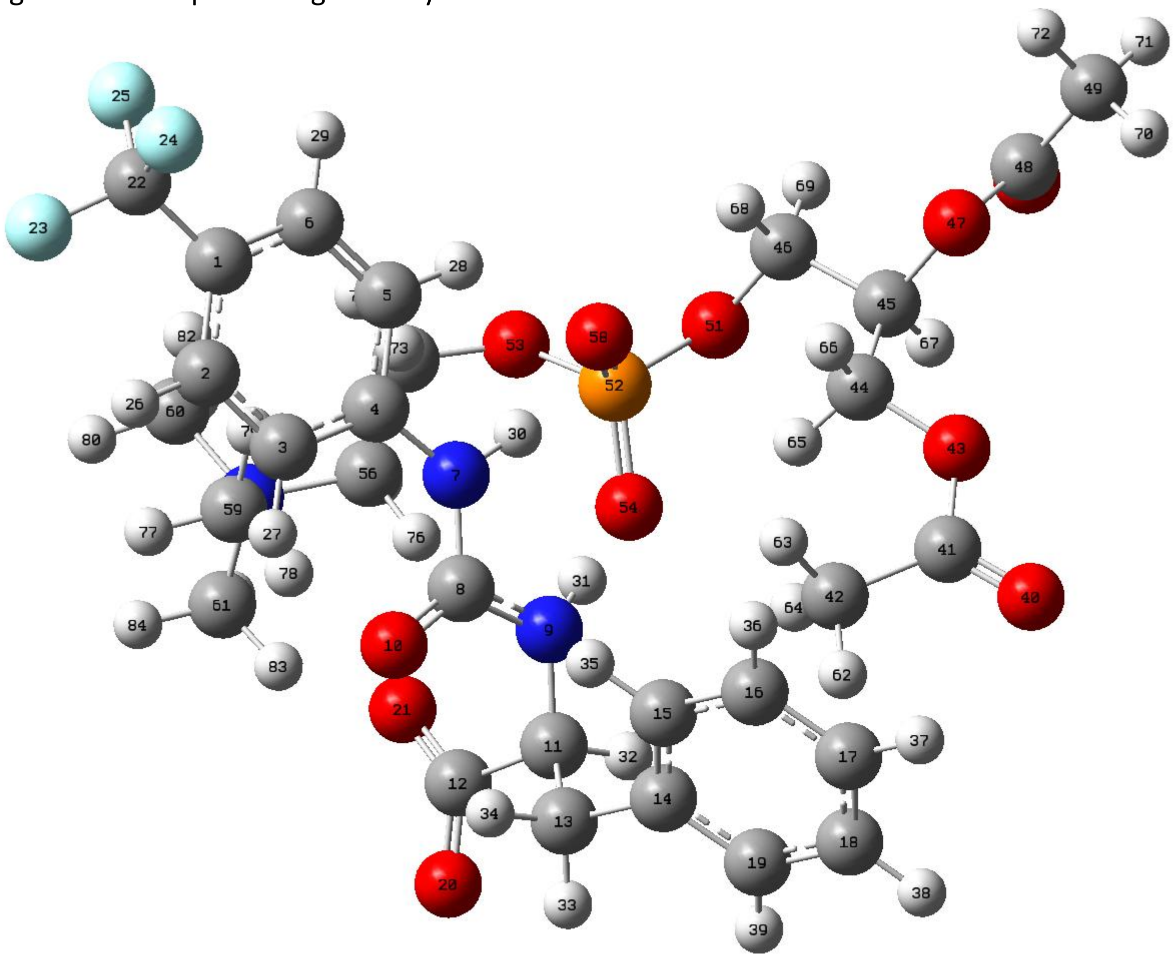

Table S31 – Cartesian Coordinates for the **9/PC-Model** binding interaction

Center Number	Atomic Number	Atomic Type	Coordinates (Angstroms) X	Y	Z
1	6	0	-10.665770	-3.212600	-4.484874
2	6	0	-11.727693	-3.330889	-3.596622
3	6	0	-11.620746	-2.864877	-2.292036
4	6	0	-10.427472	-2.263133	-1.862298
5	6	0	-9.360423	-2.139251	-2.774178
6	6	0	-9.479618	-2.608105	-4.068878
7	7	0	-10.191892	-1.800818	-0.571689
8	6	0	-11.058031	-1.848079	0.513375
9	7	0	-10.517806	-1.343360	1.656672
10	8	0	-12.208135	-2.264859	0.425384
11	6	0	-11.176489	-1.578460	2.932936
12	6	0	-11.274102	-3.095890	3.292537
13	6	0	-12.541072	-0.878202	3.075356
14	6	0	-12.483485	0.618177	2.910735
15	6	0	-12.457882	1.200899	1.639752
16	6	0	-12.403143	2.583165	1.488107
17	6	0	-12.378144	3.410851	2.609235
18	6	0	-12.411277	2.844530	3.880828
19	6	0	-12.461673	1.459554	4.026265
20	8	0	-11.991862	-3.379518	4.274597

21	8	0	-10.593009	-3.890733	2.604255
22	6	0	-10.803218	-3.670782	-5.898358
23	9	0	-11.753216	-4.607988	-6.048848
24	9	0	-11.132982	-2.664173	-6.738127
25	9	0	-9.659722	-4.190575	-6.381290
26	1	0	-12.651480	-3.798492	-3.915751
27	1	0	-12.447214	-2.970494	-1.608745
28	1	0	-8.438301	-1.678892	-2.436367
29	1	0	-8.646262	-2.510448	-4.754882
30	1	0	-9.264265	-1.389458	-0.429288
31	1	0	-9.498041	-1.350538	1.713826
32	1	0	-10.508084	-1.144927	3.686120
33	1	0	-12.912633	-1.125303	4.069433
34	1	0	-13.228223	-1.306192	2.345121
35	1	0	-12.488085	0.559627	0.764429
36	1	0	-12.383204	3.016448	0.494468
37	1	0	-12.337936	4.487502	2.492045
38	1	0	-12.397702	3.479555	4.759328
39	1	0	-12.488083	1.023360	5.020113
40	8	0	-8.269292	3.800508	3.491832
41	6	0	-8.090082	2.838851	2.787703
42	6	0	-9.101888	1.741976	2.593351
43	8	0	-6.908190	2.739213	2.145643
44	6	0	-6.678527	1.640054	1.260527
45	6	0	-5.191330	1.421367	1.134267
46	6	0	-4.885472	0.138346	0.360569
47	8	0	-4.651433	2.534082	0.409487
48	6	0	-3.355804	2.839739	0.610816
49	6	0	-2.953354	4.043403	-0.186387
50	8	0	-2.640687	2.209395	1.347586
51	8	0	-5.367390	-0.983511	1.093415
52	15	0	-6.813942	-1.651229	0.788566
53	8	0	-6.294479	-3.154497	0.419702
54	8	0	-7.620406	-1.675475	2.054358
55	6	0	-7.207374	-4.062880	-0.184524
56	6	0	-7.968379	-4.783455	0.926932
57	7	0	-8.910796	-5.854600	0.437179
58	8	0	-7.440510	-1.023896	-0.428535
59	6	0	-10.074577	-5.234940	-0.275147
60	6	0	-8.221404	-6.822599	-0.467651
61	6	0	-9.421681	-6.593987	1.637851
62	1	0	-10.011157	2.017158	3.118918
63	1	0	-9.324194	1.589626	1.535118
64	1	0	-8.722579	0.794690	2.986793
65	1	0	-7.122861	0.737657	1.673059
66	1	0	-7.121650	1.853473	0.284424
67	1	0	-4.735376	1.376550	2.125197
68	1	0	-5.346113	0.182134	-0.628828
69	1	0	-3.806586	0.025759	0.259679
70	1	0	-3.547816	4.900952	0.131812
71	1	0	-1.896315	4.244231	-0.035885
72	1	0	-3.158648	3.867310	-1.242732
73	1	0	-7.878304	-3.528761	-0.865109
74	1	0	-6.598391	-4.753175	-0.766230
75	1	0	-7.249635	-5.275417	1.584224
76	1	0	-8.575097	-4.084519	1.509065
77	1	0	-10.759117	-6.032790	-0.559608
78	1	0	-10.553247	-4.535800	0.412419
79	1	0	-9.715432	-4.729718	-1.169598
80	1	0	-8.902822	-7.648521	-0.661213
81	1	0	-7.322327	-7.185078	0.028477

82	1	0	-7.970360	-6.331681	-1.404519
83	1	0	-9.899388	-5.868025	2.294536
84	1	0	-10.135110	-7.341267	1.295030
85	1	0	-8.576823	-7.079021	2.123592

E(RM062X) = -2760.715654 Hartree

Sum of electronic and thermal Free Energies = -2760.125528 Hartree

7-PE-Model Binding

Figure S464 – Optimised geometry interaction between **7** and **PE-Model**

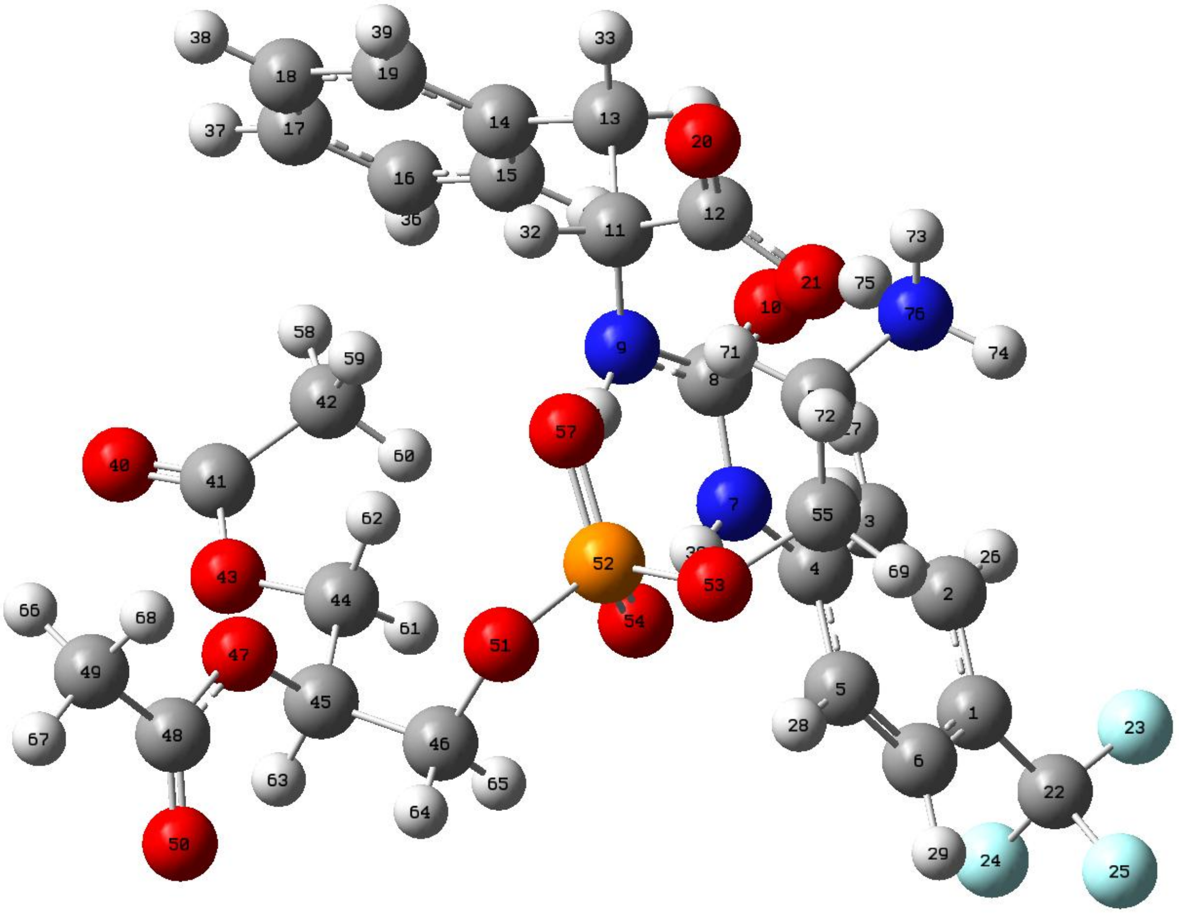

Table S32 – Cartesian Coordinates for the **7**/**PE-Model** binding interaction

Center Number	Atomic Number	Atomic Type	Coordinates (Angstroms) X	Y	Z
1	6	0	-12.454528	5.948147	0.898285
2	6	0	-12.872388	4.646648	1.152095
3	6	0	-11.957019	3.664280	1.506634
4	6	0	-10.596254	3.986170	1.608423
5	6	0	-10.183122	5.303735	1.342880
6	6	0	-11.101495	6.273946	0.988443
7	7	0	-9.597008	3.075741	1.931509
8	6	0	-9.757230	1.831287	2.512029
9	7	0	-8.565563	1.159736	2.617310
10	8	0	-10.838988	1.371554	2.851865
11	6	0	-8.299123	0.170274	3.643591
12	6	0	-8.194399	0.780131	5.044104
13	6	0	-9.244530	-1.044868	3.649970
14	6	0	-9.191140	-1.804825	2.347605
15	6	0	-10.075296	-1.507694	1.308113
16	6	0	-10.022643	-2.213191	0.108525
17	6	0	-9.080837	-3.224389	-0.069119
18	6	0	-8.188406	-3.521425	0.958196
19	6	0	-8.244711	-2.814002	2.156188
20	8	0	-7.590209	0.211674	5.932335
21	8	0	-8.803079	1.934882	5.189102

22	6	0	-13.436784	6.984387	0.464404
23	9	0	-14.683385	6.727075	0.893894
24	9	0	-13.519270	7.086960	-0.881192
25	9	0	-13.115202	8.213306	0.906876
26	1	0	-13.922550	4.390651	1.077697
27	1	0	-12.289844	2.658408	1.707374
28	1	0	-9.129663	5.548461	1.424330
29	1	0	-10.768489	7.286184	0.791887
30	1	0	-8.636679	3.441447	1.906743
31	1	0	-7.735754	1.722649	2.453402
32	1	0	-7.291370	-0.199627	3.438960
33	1	0	-8.916623	-1.692625	4.466331
34	1	0	-10.261804	-0.716097	3.855677
35	1	0	-10.805723	-0.718114	1.451359
36	1	0	-10.717997	-1.974639	-0.688212
37	1	0	-9.041711	-3.775840	-1.001241
38	1	0	-7.452142	-4.306326	0.828736
39	1	0	-7.551757	-3.053359	2.957288
40	8	0	-5.587077	-0.806906	-1.845472
41	6	0	-5.788479	-0.004655	-0.968569
42	6	0	-6.878771	-0.134782	0.057916
43	8	0	-4.988241	1.077369	-0.906885
44	6	0	-5.190473	2.058478	0.114439
45	6	0	-4.051474	3.048274	0.032815
46	6	0	-4.257358	4.227163	0.974266
47	8	0	-2.862085	2.322937	0.382147
48	6	0	-1.689150	2.801767	-0.062573
49	6	0	-0.547476	1.932097	0.371358
50	8	0	-1.597779	3.805151	-0.725590
51	8	0	-4.406603	3.792484	2.323324
52	15	0	-5.859574	3.507645	2.987162
53	8	0	-5.735056	4.527318	4.249074
54	8	0	-6.941505	3.974296	2.042163
55	6	0	-6.791108	4.472306	5.213274
56	6	0	-6.439245	3.504643	6.336280
57	8	0	-5.933854	2.087644	3.469213
58	1	0	-7.457172	-1.030654	-0.151703
59	1	0	-6.455231	-0.206248	1.062710
60	1	0	-7.534736	0.737594	0.042759
61	1	0	-6.134845	2.584991	-0.041881
62	1	0	-5.197055	1.584752	1.098453
63	1	0	-3.952933	3.426878	-0.986968
64	1	0	-3.380814	4.872985	0.941889
65	1	0	-5.136326	4.790275	0.653775
66	1	0	-0.694189	0.922198	-0.013538
67	1	0	0.387381	2.343408	0.000847
68	1	0	-0.528756	1.875523	1.460413
69	1	0	-6.920022	5.486229	5.595895
70	1	0	-7.727577	4.165121	4.735096
71	1	0	-6.110508	2.564443	5.889389
72	1	0	-5.623401	3.913628	6.938599
73	1	0	-7.417783	2.625538	7.919073
74	1	0	-8.031192	4.082215	7.525202
75	1	0	-8.499103	2.395450	6.065445
76	7	0	-7.648315	3.225823	7.132739

E(RM062X) = -2642.838783 Hartree

Sum of electronic and thermal Free Energies = -2642.331161 Hartree

9-PE-Model Binding

Figure S465 – Optimised geometry interaction between **9** and **PE-Model**

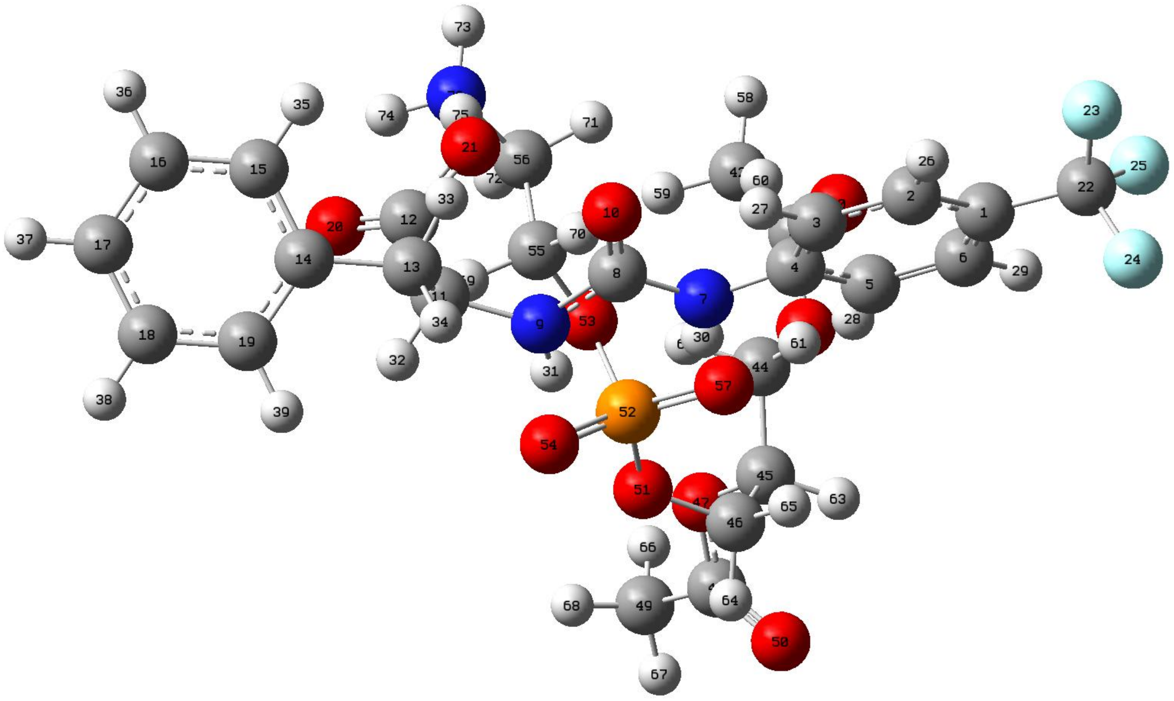

Table S33 – Cartesian Coordinates for the **9**/**PE-Model** binding interaction

Center Number	Atomic Number	Atomic Type	Coordinates (Angstroms) X	Y	Z
1	6	0	-3.775578	5.745861	-0.902481
2	6	0	-4.902285	4.960710	-1.119961
3	6	0	-4.813596	3.575081	-1.112800
4	6	0	-3.575356	2.957452	-0.883401
5	6	0	-2.443105	3.759905	-0.656525
6	6	0	-2.543708	5.139248	-0.663200
7	7	0	-3.384275	1.577045	-0.825802
8	6	0	-4.263985	0.605427	-1.277066
9	7	0	-3.873107	-0.663341	-0.948017
10	8	0	-5.295107	0.871144	-1.878714
11	6	0	-4.393235	-1.872449	-1.567787
12	6	0	-3.720394	-2.157561	-2.913142
13	6	0	-5.929385	-1.934847	-1.696371
14	6	0	-6.383198	-3.320995	-2.084171
15	6	0	-6.730363	-3.612298	-3.403821
16	6	0	-7.116850	-4.899092	-3.771594
17	6	0	-7.162034	-5.912420	-2.818959
18	6	0	-6.819737	-5.631934	-1.497529
19	6	0	-6.432218	-4.345905	-1.135887
20	8	0	-3.280692	-3.252870	-3.192922
21	8	0	-3.637391	-1.108397	-3.710254
22	6	0	-3.897405	7.232351	-0.856499
23	9	0	-4.883505	7.693760	-1.645006
24	9	0	-4.170799	7.690509	0.385884
25	9	0	-2.767708	7.853370	-1.238201
26	1	0	-5.862740	5.428862	-1.300683
27	1	0	-5.692764	2.976582	-1.288059
28	1	0	-1.489707	3.275359	-0.479561

29	1	0	-1.661440	5.744289	-0.491474
30	1	0	-2.439870	1.296993	-0.543197
31	1	0	-2.969208	-0.792795	-0.487056
32	1	0	-4.084436	-2.695352	-0.919904
33	1	0	-6.267759	-1.203082	-2.426422
34	1	0	-6.345835	-1.652550	-0.726049
35	1	0	-6.697355	-2.823944	-4.149534
36	1	0	-7.383867	-5.108551	-4.800920
37	1	0	-7.464957	-6.913555	-3.102078
38	1	0	-6.857593	-6.414814	-0.748957
39	1	0	-6.168736	-4.131370	-0.104466
40	8	0	4.864683	2.480388	-3.709202
41	6	0	3.894301	1.981035	-3.198532
42	6	0	2.676793	1.539685	-3.965237
43	8	0	3.902605	1.797202	-1.866159
44	6	0	2.761746	1.232804	-1.211737
45	6	0	3.162274	0.901429	0.205329
46	6	0	1.953919	0.527858	1.048098
47	8	0	4.085009	-0.196035	0.115463
48	6	0	4.936478	-0.376536	1.138374
49	6	0	5.838492	-1.548970	0.894577
50	8	0	4.948489	0.330584	2.115256
51	8	0	1.210937	-0.528622	0.439859
52	15	0	-0.188029	-0.282616	-0.336792
53	8	0	0.299851	-0.625732	-1.861932
54	8	0	-1.201064	-1.309281	0.081728
55	6	0	-0.771515	-0.941394	-2.749941
56	6	0	-0.282622	-0.933403	-4.186686
57	8	0	-0.581323	1.169135	-0.253713
58	1	0	2.787829	1.853689	-4.999118
59	1	0	2.585301	0.451902	-3.923099
60	1	0	1.766570	1.965922	-3.540132
61	1	0	1.940337	1.954626	-1.195464
62	1	0	2.433970	0.326524	-1.720085
63	1	0	3.664513	1.753000	0.669273
64	1	0	2.283414	0.172069	2.023656
65	1	0	1.323033	1.409383	1.177730
66	1	0	6.389399	-1.398066	-0.034384
67	1	0	6.527242	-1.660226	1.727427
68	1	0	5.235298	-2.451015	0.783131
69	1	0	-1.160397	-1.931537	-2.494632
70	1	0	-1.578808	-0.209767	-2.636617
71	1	0	-0.075367	0.089604	-4.503425
72	1	0	0.644385	-1.509486	-4.262966
73	1	0	-1.226170	-1.195883	-6.003051
74	1	0	-1.308370	-2.502338	-5.035527
75	1	0	-2.875818	-1.253670	-4.378037
76	7	0	-1.355235	-1.486210	-5.039383

E(RM062X) = -2642.828288 Hartree

Sum of electronic and thermal Free Energies = -2642.326248 Hartree

7-PG-Model Binding

Figure S466 – Optimised geometry interaction between **7** and **PG-Model**

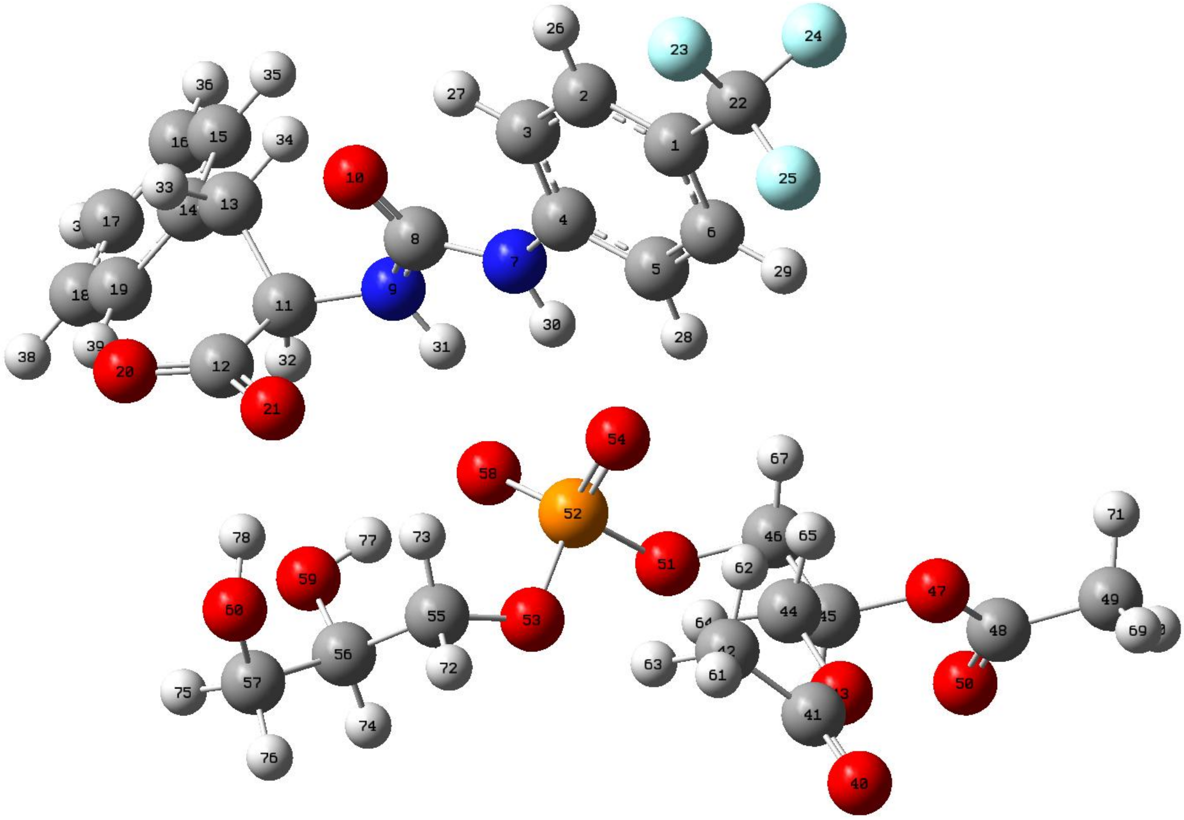

Table S34 – Cartesian Coordinates for the **7**/**PG-Model** binding interaction

Center Number	Atomic Number	Atomic Type	Coordinates (Angstroms) X	Y	Z
1	6	0	2.449041	4.886335	-0.277427
2	6	0	1.106506	5.244442	-0.326548
3	6	0	0.113887	4.275190	-0.391633
4	6	0	0.466687	2.917400	-0.411972
5	6	0	1.829438	2.565838	-0.365503
6	6	0	2.808502	3.538523	-0.301219
7	7	0	-0.437841	1.868579	-0.496678
8	6	0	-1.813691	1.923477	-0.353429
9	7	0	-2.423096	0.724937	-0.578065
10	8	0	-2.430597	2.949201	-0.086383
11	6	0	-3.734207	0.475550	-0.003746
12	6	0	-3.762229	0.642338	1.542573
13	6	0	-4.865683	1.248835	-0.724354
14	6	0	-5.927561	0.357047	-1.324637
15	6	0	-6.250592	0.431373	-2.680466
16	6	0	-7.231858	-0.393094	-3.230068
17	6	0	-7.905599	-1.307090	-2.426765
18	6	0	-7.590874	-1.390892	-1.070728
19	6	0	-6.611645	-0.566987	-0.526119
20	8	0	-4.890496	0.604810	2.073028
21	8	0	-2.657143	0.739129	2.133486
22	6	0	3.509434	5.935126	-0.267377
23	9	0	3.086927	7.094152	0.265303

24	9	0	3.952143	6.236072	-1.509546
25	9	0	4.598341	5.558706	0.427497
26	1	0	0.824591	6.290575	-0.309595
27	1	0	-0.925413	4.560945	-0.420422
28	1	0	2.097110	1.514623	-0.376757
29	1	0	3.852522	3.250624	-0.261181
30	1	0	-0.015082	0.933830	-0.533006
31	1	0	-1.841650	-0.105699	-0.650622
32	1	0	-3.902568	-0.596343	-0.149402
33	1	0	-5.335950	1.927454	-0.009576
34	1	0	-4.421006	1.860548	-1.509290
35	1	0	-5.727842	1.140897	-3.313954
36	1	0	-7.466172	-0.321344	-4.286143
37	1	0	-8.667759	-1.949976	-2.851370
38	1	0	-8.112099	-2.100095	-0.437439
39	1	0	-6.362487	-0.623314	0.529204
40	8	0	5.963921	-2.354863	2.652534
41	6	0	4.986543	-1.975550	2.058395
42	6	0	4.110124	-0.843880	2.521677
43	8	0	4.654752	-2.606325	0.917576
44	6	0	3.515791	-2.180436	0.160968
45	6	0	3.326754	-3.177316	-0.957460
46	6	0	2.081421	-2.885189	-1.791329
47	8	0	4.470112	-3.056110	-1.815663
48	6	0	4.834587	-4.137943	-2.528738
49	6	0	6.060380	-3.859516	-3.345158
50	8	0	4.234007	-5.181710	-2.490214
51	8	0	0.899152	-3.145655	-1.041854
52	15	0	0.089724	-1.972594	-0.272644
53	8	0	0.282648	-2.515930	1.244658
54	8	0	0.797721	-0.658245	-0.464199
55	6	0	-0.437271	-1.866623	2.309636
56	6	0	-1.706470	-2.630104	2.666102
57	6	0	-2.216230	-2.210115	4.039354
58	8	0	-1.373991	-2.041523	-0.626143
59	8	0	-2.749214	-2.430808	1.724771
60	8	0	-2.185084	-0.816423	4.265683
61	1	0	4.546674	-0.415314	3.419023
62	1	0	4.021049	-0.077439	1.750095
63	1	0	3.105977	-1.211056	2.744015
64	1	0	2.626689	-2.170625	0.791104
65	1	0	3.685994	-1.182398	-0.250191
66	1	0	3.278601	-4.190845	-0.555035
67	1	0	2.100991	-1.849624	-2.137251
68	1	0	2.067797	-3.556355	-2.649602
69	1	0	6.886815	-3.607456	-2.679204
70	1	0	6.311001	-4.734769	-3.937999
71	1	0	5.879751	-3.001958	-3.994035
72	1	0	0.234716	-1.854041	3.169021
73	1	0	-0.686756	-0.835491	2.037829
74	1	0	-1.450849	-3.698383	2.715628
75	1	0	-3.232215	-2.609496	4.152375
76	1	0	-1.587121	-2.670882	4.805878
77	1	0	-2.364011	-2.350181	0.831942
78	1	0	-2.474723	-0.317281	3.467135

E(RM062X) = -2776.773292 Hartree

Sum of electronic and thermal Free Energies = -2776.265795 Hartree

9-PG-Model Binding

Figure S467 – Optimised geometry interaction between **9** and **PG-Model**

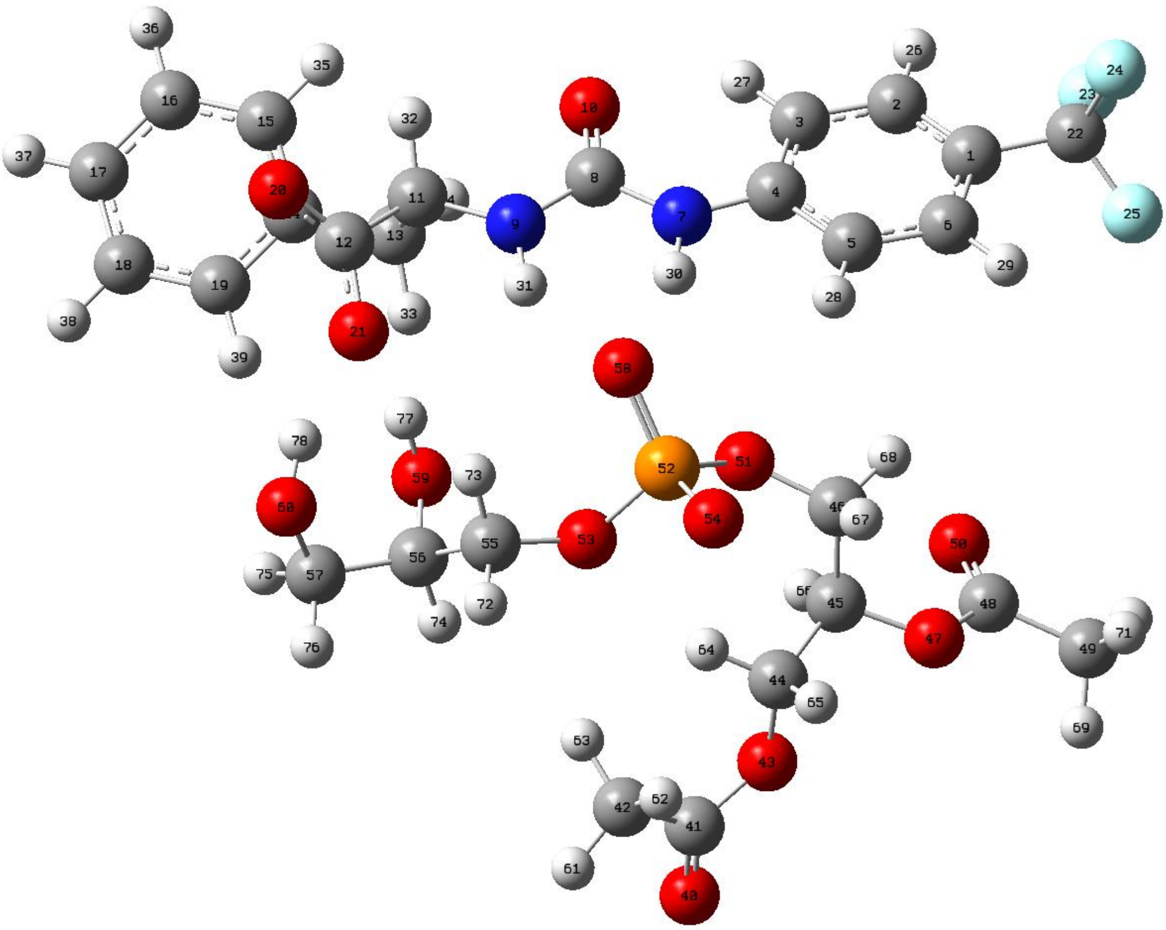

Table S35 – Cartesian Coordinates for the **9**/**PG-Model** binding interaction

Center Number	Atomic Number	Atomic Type	Coordinates (Angstroms) X	Y	Z
1	6	0	-5.699496	8.408306	-3.271232
2	6	0	-7.019018	8.146138	-3.620344
3	6	0	-7.751930	7.172756	-2.953155
4	6	0	-7.157935	6.445086	-1.911066
5	6	0	-5.824223	6.727331	-1.559338
6	6	0	-5.103114	7.695120	-2.230848
7	7	0	-7.786235	5.439528	-1.193264
8	6	0	-9.084098	4.978156	-1.324292
9	7	0	-9.339760	3.906136	-0.531167
10	8	0	-9.924214	5.489132	-2.060436
11	6	0	-10.622024	3.225336	-0.569659
12	6	0	-10.743454	2.329714	0.679042
13	6	0	-10.755629	2.371703	-1.852325
14	6	0	-12.042868	1.592089	-1.890466
15	6	0	-13.238369	2.206676	-2.271378
16	6	0	-14.437531	1.500650	-2.258078
17	6	0	-14.457043	0.164775	-1.862656

18	6	0	-13.271167	-0.457846	-1.481986
19	6	0	-12.073989	0.253178	-1.495154
20	8	0	-11.790479	2.364227	1.333824
21	8	0	-9.729525	1.600768	0.912839
22	6	0	-4.933773	9.489473	-3.956313
23	9	0	-5.403357	9.758793	-5.185626
24	9	0	-4.969582	10.658959	-3.278162
25	9	0	-3.629579	9.186540	-4.092902
26	1	0	-7.485333	8.698281	-4.427574
27	1	0	-8.772765	6.970272	-3.234895
28	1	0	-5.363798	6.164608	-0.754983
29	1	0	-4.075307	7.893691	-1.949656
30	1	0	-7.211005	4.928248	-0.515236
31	1	0	-8.575871	3.478370	-0.010281
32	1	0	-11.422868	3.965691	-0.546586
33	1	0	-9.902152	1.690782	-1.900149
34	1	0	-10.693240	3.051844	-2.704947
35	1	0	-13.225734	3.246798	-2.582954
36	1	0	-15.356219	1.991079	-2.558880
37	1	0	-15.389583	-0.387162	-1.854171
38	1	0	-13.278211	-1.497552	-1.175412
39	1	0	-11.149374	-0.230509	-1.193163
40	8	0	-1.662598	-1.594202	-2.118295
41	6	0	-2.116848	-0.704878	-1.443726
42	6	0	-2.785812	-0.923441	-0.112792
43	8	0	-2.029581	0.552722	-1.912971
44	6	0	-2.519108	1.652611	-1.136404
45	6	0	-2.796737	2.799948	-2.075081
46	6	0	-3.519818	3.943175	-1.365986
47	8	0	-1.528505	3.279175	-2.545446
48	6	0	-1.502870	3.915117	-3.731156
49	6	0	-0.110011	4.339267	-4.087848
50	8	0	-2.489935	4.103231	-4.396288
51	8	0	-4.858018	3.569099	-1.069467
52	15	0	-5.326234	3.059465	0.410063
53	8	0	-5.591089	1.492419	0.044077
54	8	0	-4.192869	3.188097	1.377273
55	6	0	-6.399452	0.709630	0.926467
56	6	0	-7.189611	-0.294294	0.101942
57	6	0	-7.936507	-1.271566	1.009822
58	8	0	-6.650567	3.719479	0.697405
59	8	0	-8.058226	0.363492	-0.802257
60	8	0	-8.659823	-0.645710	2.055370
61	1	0	-2.703847	-1.975953	0.142257
62	1	0	-2.319454	-0.314154	0.663411
63	1	0	-3.839694	-0.639821	-0.169625
64	1	0	-3.445819	1.381404	-0.636000
65	1	0	-1.768075	1.938842	-0.395193
66	1	0	-3.393319	2.455847	-2.922124
67	1	0	-2.984604	4.220405	-0.454233
68	1	0	-3.566539	4.802176	-2.036863
69	1	0	0.536948	3.462314	-4.130743
70	1	0	-0.117378	4.848531	-5.047498
71	1	0	0.276164	5.002301	-3.312667
72	1	0	-5.755504	0.190750	1.644434
73	1	0	-7.092855	1.357611	1.469377
74	1	0	-6.493578	-0.875243	-0.512353
75	1	0	-8.599375	-1.875809	0.378628
76	1	0	-7.217464	-1.942788	1.486535
77	1	0	-8.668408	0.921069	-0.274907
78	1	0	-9.194106	0.088945	1.697543

E(RM062X) = -2776.781901 Hartree

Sum of electronic and thermal Free Energies = -2776.273756 Hartree

Section S26: *In vitro* Drug Metabolism and Pharmacokinetics (DMPK) studies

Protein Binding Measurements in Human Plasma by Using Equilibrium Dialysis

Table S36 - Protein binding results of **9** and control compound in human plasma.

Compound	Species	% Bound	% Unbound	% Recovery	% Remaining at 6 hr
Ketoconazole	Human	99.14	0.86	96.60	101.17
**9**	Human	99.73	0.27	98.35	98.85

Kinetic Solubility Determination in PBS pH 7.4

Table S37 - The solubility data of **9** and control in PBS pH 7.4. The upper limit was set at 300 µM. Any value close to or above 300 µM indicates that the compound may have a solubility at or above 300 µM.

Compound	Solubility in PBS pH 7.4 (µM)
Progesterone	14.52
Diclofenac	280.79
**9**	271.13

Metabolic Stability in Rat and Mouse Liver Microsomes

Table S38 - Metabolic stability of **9** and control compound in rat and mouse liver microsomes.

Compound	Species	*in vitro* $t_{1/2}$ (min)	*in vitro* CL_{int} (µL/min/mg)	Scale-up CL_{int} (mL/min/Kg)	Predicted Hepatic CL_H (mL/min/kg)	Hepatic Extraction Ratio (ER)
Verapamil	Rat	2.40	577.23	1408.44	64.87	0.95
	Mouse	1.96	708.93	2932.13	87.32	0.97
SSA **9**	Rat	> 184.78	< 7.50	< 18.30	< 14.42	< 0.21
	Mouse	> 184.78	< 7.50	< 31.02	< 23.07	< 0.26

Table S39 - Remaining percentage of **9** and control compound in rat and mouse liver microsomes

Compound	Species	Assay format	Remaining percentage (%)				
			0.5 min	5 min	15 min	30 min	60 min
Verapamil	Rat	+Cofactors	100.00	27.30	3.04	BLOD	BLOD
		-Cofactors	100.00	-	-	-	99.51
	Mouse	+Cofactors	100.00	20.30	2.54	BLOD	BLOD
		-Cofactors	100.00	-	-	-	103.42
SSA **9**	Rat	+Cofactors	100.00	107.16	104.62	102.68	102.41
		-Cofactors	100.00	-	-	-	107.67
	Mouse	+Cofactors	100.00	101.43	97.80	99.51	99.55
		-Cofactors	100.00	-	-	-	101.52

Bidirectional Permeability in Caco-2 Cell Line

Table S40 - Permeability results of **9** and control compounds in the Caco-2 cell line.

Compound	$P_{app\ (A-B)}$ (10^{-6}, cm/s)	$P_{app\ (B-A)}$ (10^{-6}, cm/s)	Efflux Ratio	Recovery (%) AP-BL	Recovery (%) BL-AP
Metoprolol	28.43	26.41	0.93	107.79	98.96
Digoxin	0.42	18.74	45.59	95.92	99.20
SSA **9**	0.17	16.76	96.91	110.78	97.74

Table S41 - The assessment of Caco-2 cell monolayer integrity.

Compound	TEER A-B ($\Omega \times cm^2$)	TEER B-A ($\Omega \times cm^2$)	LY Leakage A-B (%)	LY Leakage B-A (%)
Metoprolol	751.82	821.75	0.13	0.15
Digoxin	732.02	768.48	0.14	0.15
SSA **9**	686.19	773.63	0.15	0.14

Section S27: SEM Images

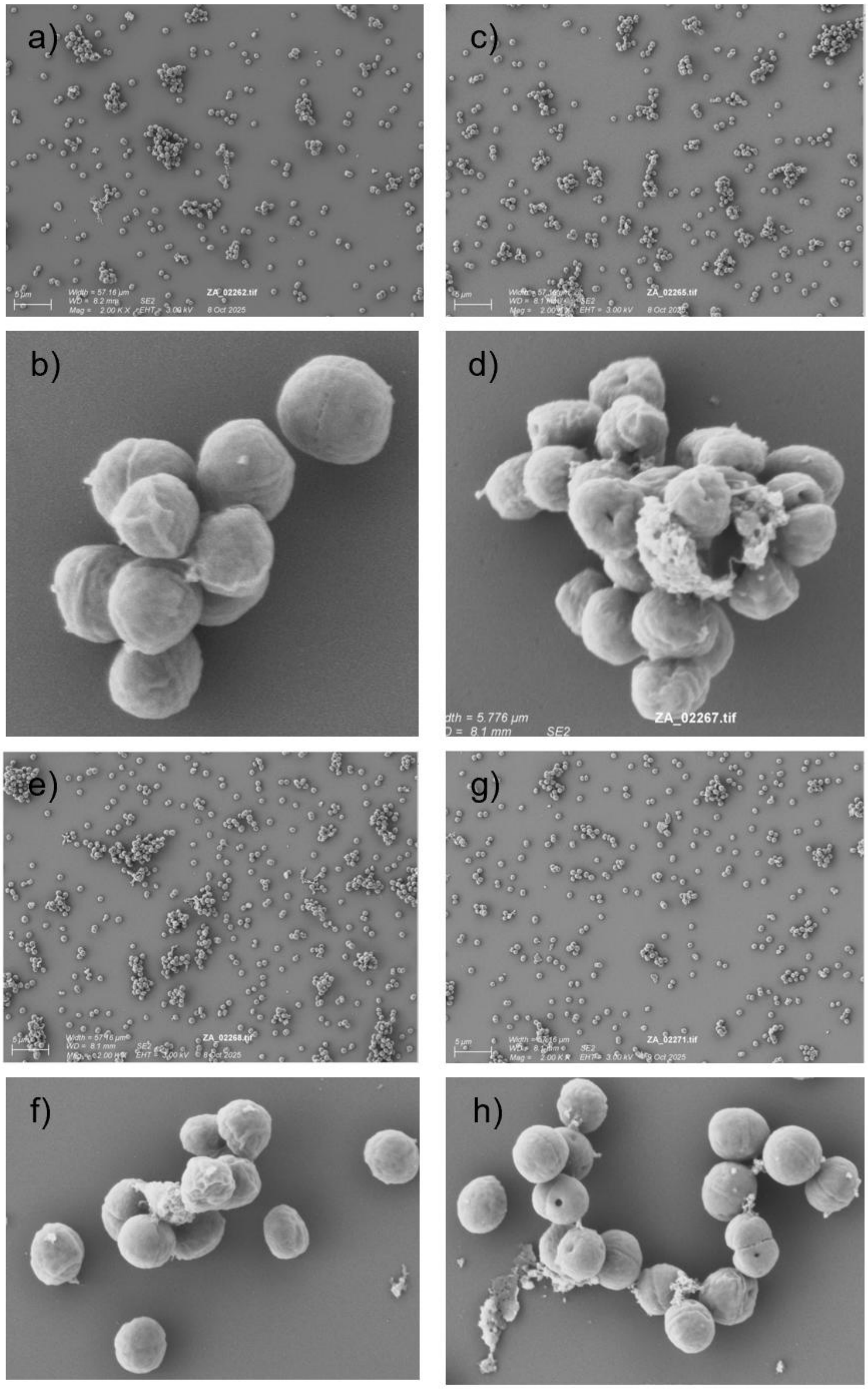

Figure S468 – SEM images of ATCC 9144 in the absence of SSA (a and b), in the presence of **7** (c and d), in the presence of **9** (e and f), in the presence of a 1:1 enantiomeric mixture of **9 + 7** (g and h).

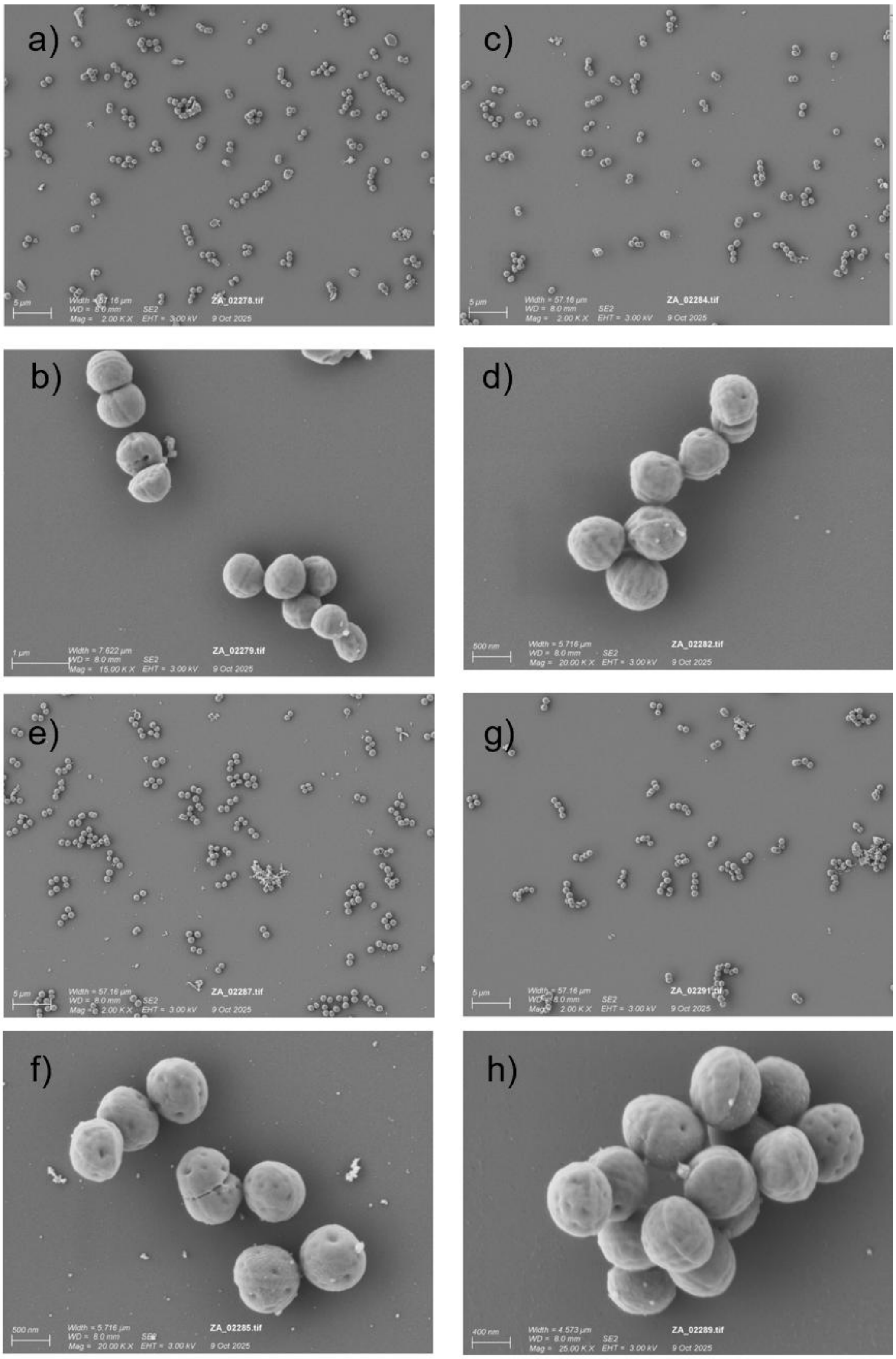

Figure S469 – SEM images of USA300 in the absence of SSA (a and b), in the presence of **7** (c and d), in the presence of **9** (e and f), in the presence of a 1:1 enantiomeric mixture of **9 + 7** (g and h).

Section S28: References

1 BindFit v0.5 | Supramolecular, http://app.supramolecular.org/bindfit/, (accessed 20 March 2024).

2 C. A. Hunter, *Angew. Chem. Int. Ed.*, 2004, **43**, 5310–5324.

3 J. J. P. Stewart, *J. Mol. Model*, 2007, **13**, 1173–1213.

4 S. M. Travis, N. N. Anderson, W. R. Forsyth, C. Espiritu, B. D. Conway, E. P. Greenberg, P. B. McCray, R. I. Lehrer, M. J. Welsh and B. F. Tack, *Infect. Immun.*, 2000, **68**, 2748–2755.

5 L. J. White, S. N. Tyuleva, B. Wilson, H. J. Shepherd, K. K. L. Ng, S. J. Holder, E. R. Clark and J. R. Hiscock, *Chem. Eur. J.*, 2018, **24**, 7761–7773.

6 A. Daina, O. Michielin and V. Zoete, *Scientific Reports 2017 7:1*, 2017, **7**, 1–13.

7 J. E. Boles, C. Bennett, J. Baker, K. L. F. Hilton, H. A. Kotak, E. R. Clark, Y. Long, L. J. White, H. Y. Lai, C. K. Hind, J. M. Sutton, M. D. Garrett, A. Cheasty, J. L. Ortega-Roldan, M. Charles, C. J. E. Haynes and J. R. Hiscock, *Chem. Sci.*, 2022,**13**, 9761-9773

8 Gaussian 16, Revision B.01, M. J. Frisch, G. W. Trucks, H. B. Schlegel, G. E. Scuseria, M. A. Robb, J. R. Cheeseman, G. Scalmani, V. Barone, G. A. Petersson, H. Nakatsuji, X. Li, M. Caricato, A. V. Marenich, J. Bloino, B. G. Janesko, R. Gomperts, B. Mennucci, H. P. Hratchian, J. V. Ortiz, A. F. Izmaylov, J. L. Sonnenberg, D. Williams-Young, F. Ding, F. Lipparini, F. Egidi, J. Goings, B. Peng, A. Petrone, T. Henderson, D. Ranasinghe, V. G. Zakrzewski, J. Gao, N. Rega, G. Zheng, W. Liang, M. Hada, M. Ehara, K. Toyota, R. Fukuda, J. Hasegawa, M. Ishida, T. Nakajima, Y. Honda, O. Kitao, H. Nakai, T. Vreven, K. Throssell, J. A. Montgomery, Jr., J. E. Peralta, F. Ogliaro, M. J. Bearpark, J. J. Heyd, E. N. Brothers, K. N. Kudin, V. N. Staroverov, T. A. Keith, R. Kobayashi, J. Normand, K. Raghavachari, A. P. Rendell, J. C. Burant, S. S. Iyengar, J. Tomasi, M. Cossi, J. M. Millam, M. Klene, C. Adamo, R. Cammi, J. W. Ochterski, R. L. Martin, K. Morokuma, O. Farkas, J. B. Foresman, and D. J. Fox, Gaussian, Inc., Wallingford CT, 2016.

9 M. Walker, A. J. A. Harvey, A. Sen and C. E. H. Dessent, *J. Phys. Chem. A*, 2013, **117**, 12590−12600.
